# Asymmetric α‐Alkylation With Activated and Unactivated Electrophiles by a Highly Productive and Recyclable Lewis Acid/Imidazolium Catalyst

**DOI:** 10.1002/anie.7069862

**Published:** 2026-05-11

**Authors:** Johanna Haußmann, Alexander Beck, Dominik Hornung, Michael Mistele, Alexander Allgaier, Wolfgang Frey, Joris van Slageren, Johannes Kästner, René Peters

**Affiliations:** ^1^ Institut für Organische Chemie Universität Stuttgart Stuttgart Germany; ^2^ Institut für Theoretische Chemie Universität Stuttgart Stuttgart Germany; ^3^ Institut für Physikalische Chemie Universität Stuttgart Stuttgart Germany

**Keywords:** catalyst design, cooperative catalysis, ion‐pairing, reaction mechanism continuum, structural anchoring

## Abstract

Asymmetric alkylation is widely used for the construction of α‐stereogenic carbonyl compounds, yet existing catalytic protocols typically suffer from several issues: (1) a limitation to π‐activated electrophiles, (2) the need for unsatisfying catalyst loadings, (3) a lack of catalyst recyclability, and (4) sophisticated catalyst structures requiring multi‐step syntheses. Herein, an efficiently accessible, air‐stable bifunctional Cu(II)/imidazolium catalyst (prepared over four steps without chromatographies in 74% yield) is reported that enables highly enantioselective α‐alkylations of 1,3‐dicarbonyls with unmet productivity (TON up to 1740). The catalyst exhibits broad electrophile compatibility, efficiently engaging π‐activated and non‐π‐activated alkylation agents. Remarkably, stereoretentive allylation with (*E*)‐ and (*Z*)‐configured allylbromides was achieved. The catalyst can be recycled over multiple cycles (10+) without loss of efficiency by a simple protocol. EPR proves formation of a Cu(II)‐enolate as resting state, for which detailed DFT calculations show that it is structurally anchored by hydrogen‐bonding to the imidazolium C(2)*H*. This feature is essential for stereodifferentiation of both enolate faces. A continuous mechanistic shift from S_N_1‐like to S_N_2‐type pathways is likely, depending on the electronic properties of the electrophile. This new alkylation concept allows for high practicality, combined with broad applicability and might serve as design prototype for future alkylation catalysts.

## Graphical abstract

An air‐stable, easily prepared, bifunctional Cu(II)/imidazolium complex enables highly enantioselective α‐alkylation of 1,3‐dicarbonyls with outstanding productivity (TON up to 1740). Highlights include broad electrophile scope spanning π‐activated and nonactivated reagents, stereo‐retentive allylation, simple catalyst synthesis, and efficient catalyst recycling. Mechanistic studies reveal a H‐bond‐anchored Cu(II) enolate enabling precise control.

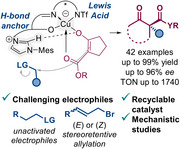

## 1 Introduction

**FIGURE 1:**
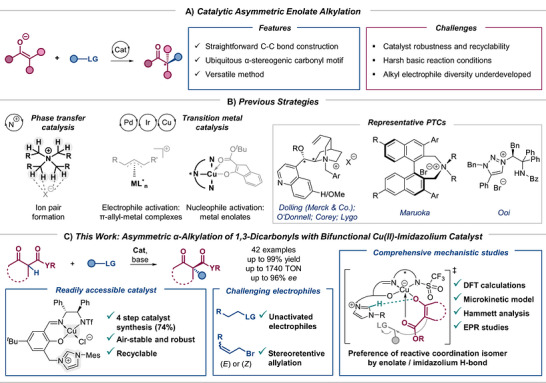
Context of this study. (A) Features and challenges of asymmetric enolate alkylation. (B) Phase transfer and transition metal catalysis and their mode of actions. Representative examples of established PTCs. (C) This work: asymmetric alkylation of 1,3‐dicarbonyls with a cooperative Cu(II)‐imidazolium catalyst.

The asymmetric enolate alkylation for the construction of C─C bonds is one of the most fundamental transformations in organic synthesis, providing direct access to ubiquitous α‐stereogenic carbonyl motifs (Figure 1A) found throughout natural products and pharmaceuticals [1, 2]. While different chiral auxiliaries initially provided reproducible access to highly enantioenriched products through stoichiometric asymmetric synthesis [3, 4, 5, 6, 7, 8, 9], a further breakthrough was achieved by asymmetric phase‐transfer catalysis (PTC), harnessing chiral ion‐pairing for stereocontrol [10]. The pioneering industrial work at Merck & Co., employing a cinchona alkaloid‐derived ammonium salt in the large‐scale technical synthesis of (+)‐indacrinone, proved a game changer [11] and inspired a number of applications of this catalyst type using different pronucleophile classes [12, 13, 14, 15, 16]. More advanced catalysts featuring axially chiral *C*_2_‐symmetric spiro‐ammonium salts were later developed by Maruoka et al. and demonstrated high efficiency for various pronucleophile types, such as α‐iminoesters [17, 18, 19]. Ooi et al. introduced chiral secondary amide/1,2,3‐triazolium salts, which were suggested to act as anion acceptor for structured ion pair formation [20].

Complementary to these developments, transition‐metal catalysis emerged as a powerful strategy. Next to classical catalytic asymmetric allylic alkylations (AAA), in which the electrophile activation occurs upon formation of electrophilic π‐allyl‐metal complexes [21, 22, 23, 24, 25], more general strategies in terms of transferrable alkyl moieties employ nucleophile activation through metal enolate formation [26, 27, 28]. Despite this significant progress, asymmetric enolate alkylation faces substantial challenges in terms of applicability. Catalysts’ recyclability was rarely accomplished [29], suggesting degradation under basic conditions. The more advanced reported systems are only accessible by multistep syntheses, making them costly and time‐consuming to prepare [10]. Furthermore, substrate scopes usually excluded low‐reactivity electrophiles and base‐sensitive substrates. Most methods are restricted to particularly reactive electrophiles, while approaches broadly applicable to diverse alkylation agents [26, 27] continue to be underdeveloped. These obstacles remain difficult to overcome [2].

Herein, we present a robust, highly productive (TON up to 1740), readily and rapidly accessible bifunctional Cu(II)/imidazolium catalyst for α‐alkylation of 1,3‐dicarbonyl pronucleophiles (Figure 1C). The catalyst tolerates a broad electrophile scope, efficiently not only engaging electrophiles activated by π‐systems (i.e., benzylic, allylic, and propargylic electrophiles) but also those without such activation. Particularly noteworthy is also that stereoretentive allylations with (*E*)‐ and (*Z*)‐configured allyl bromides are enabled, a task that has only recently been successfully addressed, mainly by electrophilic allyl metal complexes for few substrate classes [28, 30, 31, 32, 33, 34]. Moreover, both cyclic and acyclic pronucleophile classes could be employed. Due to its robustness, the air‐stable catalyst can be recycled multiple times with consistent performance applying an operationally simple protocol. Comprehensive mechanistic investigations suggest that a Cu(II) enolate is formed, which is structurally anchored and precisely preorganized via hydrogen bonding to the C(2)─H bond of the imidazolium moiety favoring the reactive coordination isomer. A continuous mechanistic shift from more S_N_1‐like to S_N_2 reaction pathways is likely, depending on the electrophile's electronic situation.

## 2 Results and Discussion

### 2.1 Catalyst Development and Optimization

The α‐alkylation of β‐ketoester **1a** and benzyl bromide was selected as model reaction, initially employing triazolium‐based catalyst **C1** (5 mol%) combined with diisopropylethylamine (DIPEA) as base (Table 1). An initial solvent screening identified EtOAc as suitable green solvent and allowed for product formation in quantitative yield with good enantioselectivity (#1). Subsequent investigation of azolium moieties showed that benzimidazolium derivative **C2** −useful in asymmetric 1,4‐additions [35]− provided diminished yield, while imidazolium variants displayed promising performance (#2–5) [36, 37]. Systematic variation of the *N*‐substituent revealed moderate performance for both small (Me) and sterically exceedingly demanding (2,6‐diisopropylphenyl, DIPP) groups, whereas *N*‐mesityl imidazolium matched the triazolium performance. We continued the optimization with the imidazolium catalyst **C5**, as it demonstrated superior performance at lower catalyst loadings (not shown) and is more efficiently accessible. At −20°C, the enantioselectivity increased to 95% *ee* with **C5**, and a quantitative yield was still achieved (#6). To allow for an efficient reaction outcome using a ten‐fold lower catalyst loading, that is, 0.5 mol% of **C5**, reactions were performed at 0°C under the optimized conditions, delivering **3aA** in quantitative yield and with 92% *ee* (#7). Notably, these results were fully reproducible also under atmospheric conditions, underscoring the robustness and operational simplicity of the catalytic protocol (#8).

**TABLE 1:** Optimization of the α‐alkylation of β‐ketoester **1a** with benzyl bromide^a^.

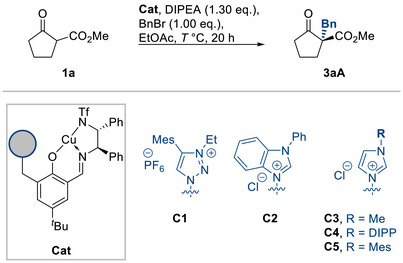				
#	Cat [mol%]	*T* [°C]	Yield^b^ [%]	ee^c^ [%]
1	**C1** (5.0 mol%)	25	>99	89
2	**C2** (5.0 mol%)	25	70	88
3	**C3** (5.0 mol%)	25	86	77
4	**C4** (5.0 mol%)	25	62	87
5	**C5** (5.0 mol%)	25	>99	90
6^d^	**C5** (5.0 mol%)	−20	>99	95
7^d^	**C5** (0.5 mol%)	0	>99	92
8^d^, ^e^	**C5** (0.5 mol%)	0	>99	92

^a^ The reactions were carried out at 0.10 mmol scale under nitrogen atmosphere.

^b^ Yield was determined by ^1^H‐NMR analysis of the crude product using mesitylene as internal standard.

^c^ Given ee‐values were determined by HPLC on chiral stationary phase.

^d^ 2.00 eq. of benzyl bromide were used.

^e^ Performed at 0.30 mmol scale under atmospheric conditions. DIPEA: diisopropylethylamine. DIPP: 2,6‐diisopropylphenyl. Mes: mesityl.

### 2.2 Scope

**SCHEME 1:**
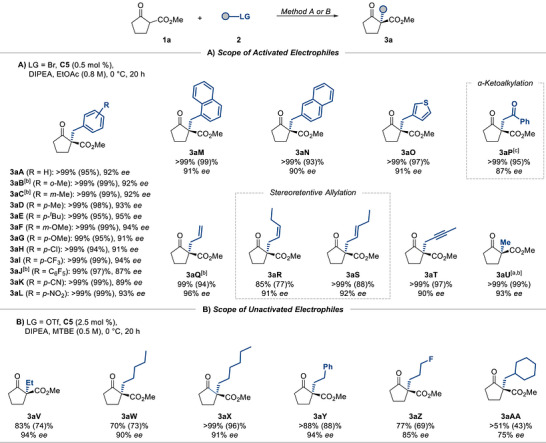
Scope of (A) activated electrophiles and (B) unactivated electrophiles. The yield was determined by ^1^H‐NMR analysis of the crude product using mesitylene as internal standard. The yield of isolated products after column chromatography is reported in parentheses. Given ee‐values were determined by HPLC or GC on chiral stationary phase. [A] Methyl iodide (4.00 equiv.) was used as alkylation agent. [B] 48 h reaction time. [C] Performed at −20°C.

With the optimized conditions established, we next explored the electrophile scope (Scheme 1). Electrophiles activated by π‐systems in α‐position to the electrophilic center, such as benzyl bromides bearing *o*‐, *m*‐, or *p*‐substituents −both electron‐donating and electron‐withdrawing− were uniformly well tolerated, affording products **3aA**–**3aL** in nearly quantitative yield with 87%–95% *ee* (Scheme 1A). Other aromatic systems, including 1‐ and 2‐naphthyl as well as thienyl groups, were also successfully employed (**3aM**–**3aO**, 93%–99%, 90%–91% *ee*). At −20°C, an α‐ketoalkylation furnished the 1,4‐diketone **3aP** (95%, 87% *ee*) without detectable side product formation. This transformation type has been rarely efficiently achieved [29] outside photochemical approaches [38, 39, 40], arguably due to product sensitivity and competing reaction pathways.

Of particular note is the finding that the method allows for complete stereoretention with geometrically pure allylic bromides, stereospecifically delivering products **3aR** and **3aS** (77%–94%, 91%–96% *ee*) from (*E*)‐ and (*Z*)‐configured olefins, respectively. Such stereospecificity is an ongoing challenge in allylation reactions, mainly making use of electrophilic allyl–metal complexes, for which only recently the first successes were reported for specific substrate classes [30, 31, 32, 33, 34]. Moreover, the reactions proceeded with full regioselectivity avoiding a competing γ‐substitution.

In addition, propargyl bromide was readily accommodated, regioselectively forming **3aT** avoiding any detectable allene side product. Moreover, methylation with methyl iodide afforded **3aU** in quantitative yield and 93% *ee*.

We next turned to the transfer of unactivated alkyl groups (Scheme 1B). Despite the high productivity with methyl iodide, with ethyl iodide, no product formation was observed, while alkyl triflates were identified as suitable in anhydrous MTBE. Under these conditions, linear alkyl triflates transferring ethyl‐, *n*‐pentyl, *n*‐hexyl, and 2‐phenylethyl moieties were well accepted, affording **3aV‐** **3aY** in 73%–96% yield and 90%–94% *ee*. With 3‐fluoropropyl triflate, enantioselectivity reduced to 85% *ee*. β‐Branched triflate provided **3aAA** in moderate yield and *ee* value of 75%, which is to our knowledge still the so far highest reported enantioselectivity for the alkylation of β‐ketoesters with this type of electrophile.

**SCHEME 2:**
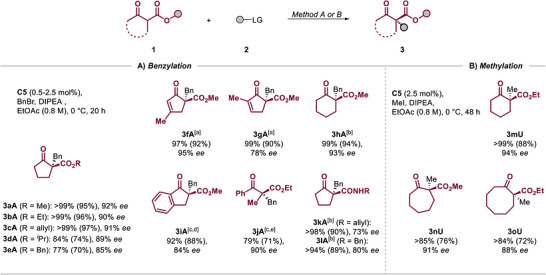
Scope of nucleophiles in (A) benzylations and (B) methylations. The yield was determined by ^1^H‐NMR analysis of the crude product using mesitylene as internal standard. The yield of isolated products after column chromatography is reported in parentheses. Given ee‐values were determined by HPLC or GC on chiral stationary phase. [A] 1 mol% of catalyst was used. [B] 2.5 mol% of catalyst was used. [C] 5.0 mol% of catalyst was used. [D] 30% aq. K_2_CO_3_ was used as base. [E] NaOH was used at base. Performed at −20°C under inert atmosphere.

The examination of various pronucleophiles (Scheme 2) showed that differences in ester groups have a relatively small influence on the reaction outcome. It was found that enantioselectivity is slightly decreasing as steric bulk increased (**3aA**–**3eA**, 85%–92% *ee*). For less reactive pronucleophiles, catalyst loadings were adjusted to 1–5 mol%. 3‐Methylcyclopentenone derivative **3fA** was smoothly obtained despite the presence of three π‐activated acidic positions. The 2‐methyl analogue **3gA** afforded a comparably high yield, but stereocontrol proved to be more difficult (*ee *= 78%), arguably for disturbing steric effects. The more readily enolizable indanone derivative required phase‐transfer conditions using aq. K_2_CO_3_ to suppress background reactivity (**3iA**, 88%, 84% *ee*), while an acyclic β‐ketoester, which is less acidic than cyclic ones, was successfully applied in the presence of solid NaOH at −20°C (**3jA**, 71%, 90% *ee*). In addition, six‐ to eight‐membered β‐ketoesters were effectively benzylated or methylated providing **3hA** and **3mU‐3nU** in 72%–94% and 88%–94% *ee*. Moreover, NH‐acidic secondary β‐ketoamides featuring *N*‐allyl and ‐benzyl protecting groups were found to be compatible, albeit with reduced enantioselectivity (**3kA–3lA**, 89%–90% yield, 73%–80% *ee*).

### 2.3 Catalyst Synthesis, Recycling, and Scale‐Up

**FIGURE 2:**
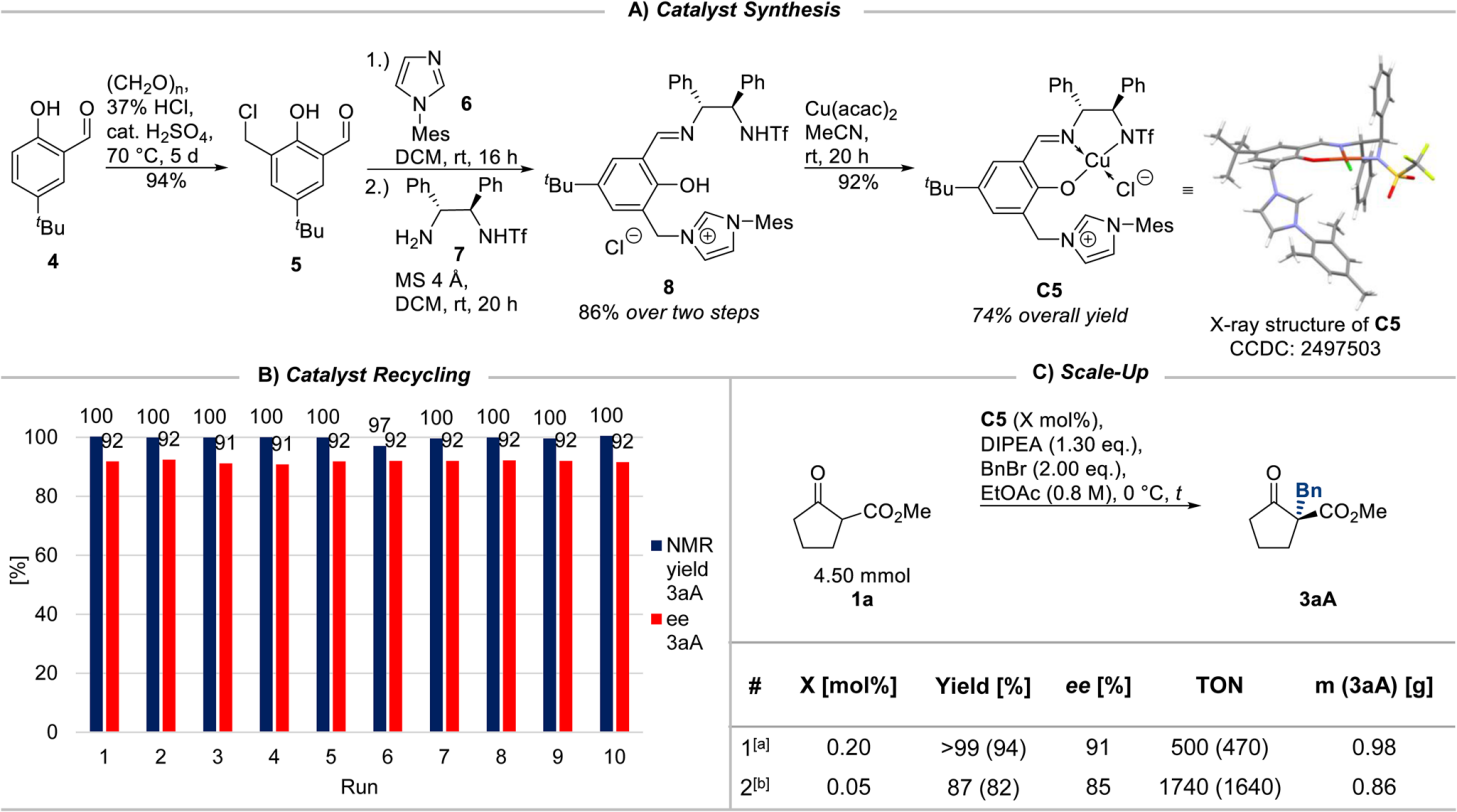
A) Catalyst Synthesis. (B) Catalyst recycling. The catalytic reactions were carried using **1a** (0.30 mmol), benzyl bromide (0.60 mmol), DIPEA (0.39 mmol), and C5 (0.5 mol%) in EtOAc (0.8 M) under atmospheric conditions. The yield was determined by ^1^H‐NMR analysis of the crude product using mesitylene as internal standard. Given ee‐values were determined by HPLC on chiral stationary phase. (C) Scale‐Up experiments. [A] 6 days reaction time. [B] 7 days reaction time.

The catalyst is readily accessible from commercial building blocks via a convenient four‐step sequence in analogy to our previously reported related benzimidazolium catalyst [41, 42], providing an overall yield of 74% (Figure 2A) [43]. Complexation with Cu(acac)_2_ afforded catalyst **C5**, whose structure was confirmed by x‐ray crystallography [43]. In this structure, the Cu(II) center adopts a slightly distorted square planar coordination geometry, in which chloride serves as fourth ligand, formally resulting in a negative charge on Cu, which is formally the counterion of the imidazolium residue. Notably, the purification in the entire synthesis relies solely on extractive work‐up or precipitation, eliminating the need for column chromatography.

Preliminary time‐course studies of conversion and *ee*, together with UV–vis experiments on the model reaction (see Supporting Information), provided initial insights into the stability of the catalyst and encouraged us to explore its recyclability. After its application to catalysis and removal of the side product DIPEA∙HBr by filtration over Celite, the catalyst was separated from the crude mixture by filtration over a small pad of silica gel using PE/EtOAc (2:1). The catalyst retained on the silica gel was subsequently recovered with more polar eluent DCM/MeOH (10:1), enabling reuse in further reactions.

Remarkably, with an initial catalyst loading of 0.5 mol%, ten consecutive runs were performed without any loss of productivity or enantioselectivity (Figure 2B) thus, demonstrating its high robustness. The overall turnover number (TON) thus accumulated to the theoretical maximum of nearly 2000, but the limits were not further probed.

Scale‐up experiments of the model reaction were performed using a reduced catalyst loading of 0.20 mol% **C5**, delivering 0.98 g of **3aA** (NMR yield >99%, yield of isolated product 94%, 91%*ee*, TON = 500 regarding NMR yield; TON = 470 based on isolated material, Figure 2C). A scale‐up experiment with as little as 0.05 mol% still afforded useful enantioselectivity (85% *ee*) and high efficiency (NMR yield 87%, yield of isolated product 82%). A TON of 1740 was thus obtained (TON = 1640 based on isolated material), demonstrating both the robustness and unique productivity of the catalyst.

### 2.4 Control Experiments

To probe the impact of the catalyst functionalities, a series of control experiments (Table 2) was conducted and compared with the performance of **C5** (#1+2). In the absence of base, no product formation was observed using standard catalyst **C5** (#3). In the presence of stoichiometric base, but without catalyst, only 3% yield was noted, indicating only minimal background reactivity (#4). Cu(II) complex **C6**, lacking the imidazolium moiety, and featuring just a Me substituent instead, gave drastically reduced yield and enantioselectivity (#5). The simple achiral imidazolium salt **C11** furnished product in low yield (15%, #6), while binary mixtures of both catalyst fragments failed to improve yield, though a slight increase in enantioselectivity was noted, pointing out the superiority of the olium moiety within a bifunctional catalyst system (#7).

**TABLE 2:** Control experiments with benzyl bromide^a^.

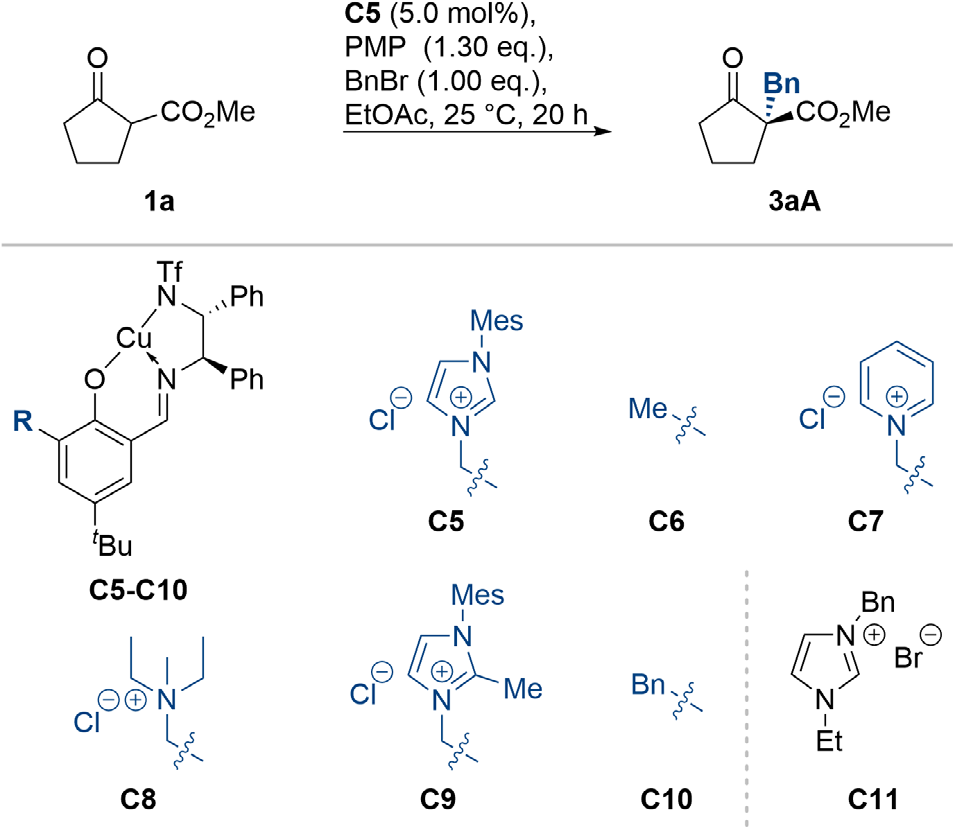			
#	Deviation from given conditions	Yield^b^ [%]	ee^c^ [%]
1	None	95	89
2	**C5** (0.5 mol%)	95	90
3	Without additional base	0	—
4	No catalyst	3	—
5	**C6**	45	13
6	**C11**	15	—
7	**C6** and **C11**	39	23
8	**C7** (5.0 mol%)	44	79
9	**C8** (5.0 mol%)	83	84
10	**C8** (0.5 mol%)	39	78
11	**C9** (5.0 mol%)	92	85
12	**C9** (0.5 mol%)	65	83
13	**C10** (5.0 mol%)	34	6

^a^ The reactions were carried out at 0.10 mmol scale under nitrogen atmosphere.

^b^ Yield was determined by ^1^H‐NMR analysis of the crude product using mesitylene as internal standard.

^c^ Given ee‐values were determined by HPLC on chiral stationary phase. PMP: 1,2,2,6,6‐Pentamethylpiperidine.

Using high catalyst loadings of 5 mol% at 25°C, pyridinium and ammonium scaffolds like in **C7** and **C8**, respectively, might be used as alternative catalysts as they provided moderate to good yields (44%–83%) and enantioselectivities (79%–84% *ee*) [44, 45, 46, 47, 48]. Nevertheless, the imidazolium‐based system is significantly superior at lower catalyst loading (compare #2 and #10).

**C9**, featuring a Me substituent at C(2) of the imidazolium fragment, performed comparably to ammonium catalyst **C8**, exhibiting decreased productivity at low loadings (#11–12) compared to **C5**. This again underscores the advantage of the C(2)‐H motif in the imidazolium system (#2). To probe steric effects, we also prepared the *ortho*‐benzyl substituted catalyst **C10**, which was slightly inferior compared to its Me analogue **C6**.

Additional control experiments were conducted using ethyl triflate (Table 3). The neutral Cu(II) complex **C6** provided a drastically reduced yield and almost no enantioselectivity (#2). Diminished performance was also observed for ammonium catalyst **C8** and the C(2)‐Me substituted imidazolium catalyst **C9**.

**TABLE 3:** Control experiments with ethyl triflate^a^.

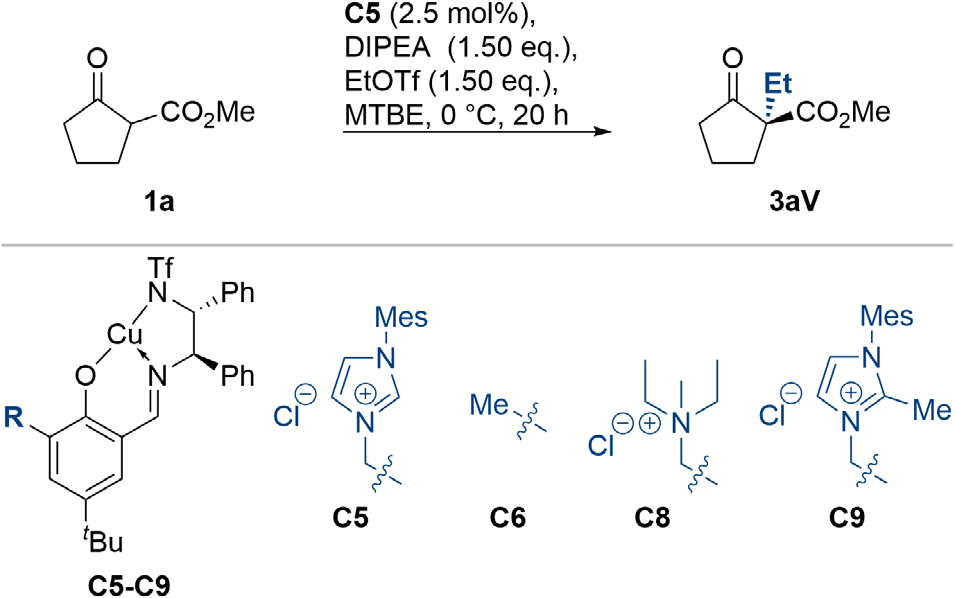			
#	Deviation from given conditions	Yield^b^ [%]	ee^c^ [%]
1	None	83	94
2	**C6**	27	5
3	**C8**	30	54
4	**C9**	52	87

^a^ The reactions were carried out at 0.10 mmol scale under nitrogen atmosphere.

^b^ Yield was determined by ^1^H‐NMR analysis of the crude product using mesitylene as internal standard.

^c^ Given ee‐values were determined by GC on chiral stationary phase.

The results of Tables 2 and 3 thus show that a positive charge within the appended side arms R is essential to achieve notable activity and good enantioselectivity. However, for an optimal performance the CH‐acidic azolium was found to be required, suggesting that H‐bonding plays a crucial role for the catalytic system. While quaternary ammonium salts as well as CH acidic moieties are also known to act as weak H‐bond donors by their polarized C─H bonds [10, 49, 50], the C(2)─H imidazolium bond is known as a very potent H‐bond donor, comparable to secondary amides [50, 51].

### 2.5 Hammett Analysis

**FIGURE 3:**
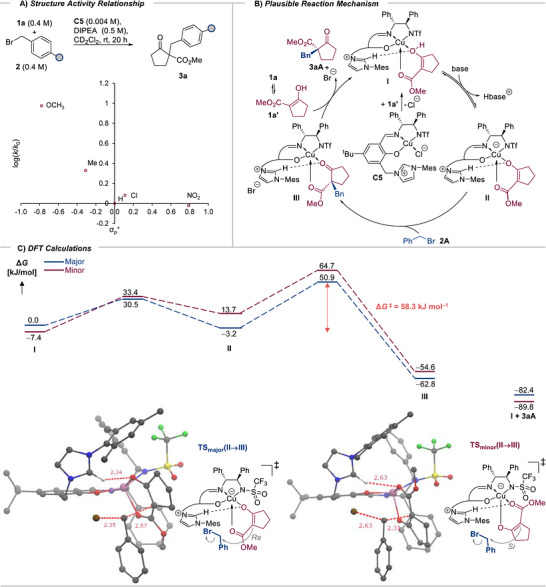
Mechanistic Investigations. (A) Structure activity relationship by Hammett Analysis. (B) Schematic representation of the proposed reaction mechanism leading to the formation of the main product. DIPEA is used as base. (C) Free energy profile of the investigated reaction mechanism leading to the major product (blue) and minor product (purple) using DCM as solvent. The energetic span of the reaction (Δ*G* ^‡^_major_ = 58.3 kJ mol^−1^) is depicted in red. The rate‐limiting transition state TS(**II** → **III**) is shown between structures **II** and **III** in the major and the minor product channel. The interaction between the imidazolium C(2)‐H and the β‐ketoester is indicated (Å). White: H, grey: C, red: O, blue: N, yellow: S, green: F and pink: Cu. Most hydrogens are omitted for visual clarity.

To gain insight into the mechanistic pathway of the alkylation, a Hammett analysis was conducted utilizing *para*‐substituted benzyl bromides (Figure 3A). In a typical S_N_2 process, electron‐deficient electrophiles are expected to react faster owing to stabilization of the associative, electron‐rich transition state. However, little substituent effect was observed for σ‐ and π‐acceptors (Cl, NO_2_), as reflected in the low log(*k*/*k*_0_) values, whereas an accelerating trend was obtained for σ‐ and π‐donor substituents (Me, OMe) with a negative Hammett reaction constant ρ. In this latter case, this suggests that stabilization of a positive partial charge in the rate limiting transition state in a more S_N_1‐like mechanism accelerates the C─C‐bond formation, with a more advanced C─Br bond cleavage as compared to the C─C bond formation. On the other hand, the nearly negligible effect of σ‐ and π‐acceptors indicate a mechanistic change, as positive charge stabilization in the limiting transition state is irrelevant. This may also at least in part rationalize the successful use of the unactivated primary alkylation agents as electrophiles, in which a positive charge upon leaving group release would be poorly stabilized [52]. The relatively high rate insensitivity for benzyl bromides in the absence of donor substituents suggests a nearly electron‐neutral reactive center in the limiting TS, which might be best explained by an asynchronous S_N_2‐ mechanism.

### 2.6 EPR Studies

The properties of **C5** and its interactions with the substrates **1a**, **2A,** and DIPEA were studied by X‐band electron paramagnetic resonance (EPR) measurements on frozen solutions, to gain insight into the electronic structure of the catalyst and the influence of substrates. The spectrum of **C5** is poorly resolved, and appears to contain contributions from several species, potentially due to distinct coordination isomers (see Supporting Information). The addition of **1a** (500 equiv.) or of **2A** (1000 equiv.) does not change the spectrum significantly. In contrast, the combination of **1a** and DIPEA (1:1.3) added to **C5** leads to a well resolved EPR spectrum with three well resolved peaks at low field and a strong derivative feature at higher fields, both of which are characteristic for Cu(II) EPR spectra at X‐band. Simulations of the spectrum give *g*‐tensor and *A*‐tensor (due to the hyperfine interaction of the electron spin to the copper nuclear spin) values of *g*_‖_ = 2.274(1) and *A*_‖_(Cu) = 485(2) MHz ( = 161·10^−4^ cm^−1^) [53]. The ratio *g*_‖_/*A*_‖_ = 141 cm indicates a five coordinate species with a strong axial contribution [54, 55]. Further addition of **2A** to arrive at catalytic conditions does not change the shape of the spectrum any further and is stable over the course of the reaction time. These results indicate that **1a** is the primary coordinating substrate, but coordination only takes place in the presence of DIPEA. The addition of the non‐nucleophilic base DIPEA enables the formation of the enolate, which is a more strongly coordinating species, and which is essential for the initiation of the catalytic cycle. This species represents the resting state in the catalytic cycle, since the spectrum does not change upon further addition of **2A**.

### 2.7 Mechanistic Investigation via DFT

Quantum chemical calculations were performed with density functional theory (DFT) at the B3LYP [56, 57, 58]‐D3(BJ) [59, 60]/def2‐TZVP [61, 62]/COSMO‐rs [63, 64] (DCM) level of theory on PBEh‐3c [65]‐D3(BJ) [59, 60]/def2‐mSVP [65] optimized geometries (for a comprehensive overview of the computational details see the Supporting Information).

The crystal structure of **C5** served as the starting point for computing a plausible reaction mechanism shown in Figure 3B. Substrate binding was modeled by replacing the chloride counterion with the enol form of the β‐ketoester, generating intermediate **I**, which is then deprotonated by DIPEA to form the catalyst–enolate complex **II**. The C─C bond‐forming step proceeds via nucleophilic attack of the enolate on the electrophile, yielding the catalyst–product adduct **III**. Subsequent product release and binding of the next β‐ketoester in its enol form close the cycle.

The barrier for the initial substrate binding from complex **C5** to give **I** is 75.1 kJ mol^−^^1^. The corresponding energy profile of the catalytic cycle starting from **I** is given in Figure 3C. Several coordination modes of β‐ketoester in **I**, including both keto and enol forms (**1a** and **1a′**), as well as mono‐ and bidentate coordination, were considered. Among these, bidentate coordination of the β‐ketoester **1a** in its keto form was found to be the most favorable. A similar coordination of the enol form within **I** was found to be energetically higher by 31.1 kJ mol^−1^. However, the deprotonation of the enol complex **I** has a significantly lower barrier than in its keto counterpart. Thus, we primarily focus on the enol form in the description here (for more details about the deprotonation of the non‐coordinated ketoester and the ketoester complex, see the Supporting Information).

We consider two pathways in Figure 3C, corresponding to the formation of the major and minor product enantiomers. For the major product channel, in catalyst‐substrate complex **I** the Cu(II) center adopts a distorted square–pyramidal geometry, with the oxygen of the former keto group coordinating in the equatorial position and the ester group in the axial position. In the minor channel the coordination geometry is inverted, that is, the position of the former keto oxygen and the ester is reversed (see Supporting Information). Coordination isomer **I_minor_** leading to the minor enantiomer has an energy 7.4 kJ mol^−1^ lower than **I** leading to the major product. However, the overall barrier for the major enantiomer channel is still lower.

For the deprotonation step (**I**→**II**) the reaction barrier was found to be 30.5 kJ mol^−1^ in the major product channel and 40.8 kJ mol^−1^ in the minor product channel with respect to **I**. The barrier of this reaction step is relatively small, as the hydrogen of the enol is readily accessible. By contrast, the deprotonation of the coordinated β‐ketoester in its keto form involves a substantial barrier of 95.6 kJ mol^−1^, which is significantly larger than the overall barrier of the mechanistic cycle involving the enol form. The higher activation barrier for deprotonation of the keto complex compared to that of the enol complex **I** or even the non‐coordinating ketoester **1a** (Δ*G* ^‡^ = 88.7 kJ mol^−1^ in DCM and 75.0 kJ mol^−1^ in EtOAc) can be attributed to the steric demand of DIPEA and the reduced steric accessibility of the relevant hydrogen atom in the keto complex form (see SI). The transition state from **I** to **II** of the major pathway is stabilized by a hydrogen‐bond interaction between C(2)─H of the imidazolium moiety serving as H‐bond donor and the nucleophilic carbon atom of the enol(ate), exhibiting a C_enol_^…^HC(2) distance of 2.55 Å. The energetic difference of intermediate **II** between the major channel (−3.2 kJ mol^−1^) and the minor channel (13.7 kJ mol^−1^) is partly explained by the different coordination modes of the respective enolates. While in the major channel the enolate oxygen coordinates to the metal center in the equatorial plane (calculated bond length Cu─O_enolate_: 1.94 Å), which allows for stronger binding than in the axial position (calculated bond length Cu─O_enolate_: 2.05 Å), in the minor channel the equatorial position is used for the weaker coordinating ester group (calculated bond lengths Cu─O_ester_: 1.96 Å, compared to 2.11 Å in the axial position). The barrier between **I** and **II** is small enough for fast switch between the major and minor channel to occur, resulting in a Curtin–Hammett situation. The energetic span is hence defined between **I** in the minor channel and the transition state **TS(II** **→** **III)** of the major channel.

Starting from **II**, the alkyl electrophile associates to the complex to undergo C─C bond formation via the rate‐determining transition state **TS(II** **→** **III)** to afford **III**, establishing the product configuration. DFT predicts that the overall barrier, that is, the energetic span of a catalytic cycle, in the major channel (Δ*G* ^‡^_major_ = 58.3 kJ mol^−1^) is significantly lower than the overall barrier in the minor channel (Δ*G* ^‡^_minor_ = 72.1 kJ mol^−1^). The transition states **TS(II** **→** **III)** again differ, like in **II**, in the enolate orientation. For further analysis we computed the CM5 partial charges [66] of the corresponding oxygen atoms for both channels. In the major channel, the enolate oxygen is bound in the equatorial position with a partial charge of O_major,eq._ = −0.374, while in the minor channel a partial charge of O_minor,eq._ = −0.356 was found with the equatorially binding ester O.

An inherent equatorial preference for the enolate oxygen favoring the major channel is attributed to its higher electron density, resulting in stronger ligand properties. In the Jahn–Teller‐like distortion in the square pyramidal Cu(II) complexes, the axial ligands are more weakly bound, as can be seen by the Cu─O bond lengths in both channels. In the major channel, where the enolate occupies the equatorial position, the Cu─O_eq_ distance was calculated to be 1.97 Å within **TS(II** **→** **III)**, while a Cu─O_ax_ distance of 2.13 Å was found for the ester group. For the minor channel, in which the ester is equatorially bound, a Cu─O_eq_ distance of 2.00 Å and a Cu─O_ax_ distance of 2.12 Å for the axially bound enolate were found. A coordination of the stronger ligand in the equatorial plane is apparently energetically preferred and thus crucial for the observed high enantioselectivity, because **TS(II** **→** **III)** is also the turnover‐determining transition state.

In the case reported here, the preference for the equatorially bound enolate‐O is further enhanced by more efficient H‐bonding, as judged from the calculated H‐bond distances between C(2)‐H of the imidazolium unit and the respective equatorial O‐atom (2.34 Å for the major path with the enolate‐O versus 2.63 Å for the minor path with the ester‐O) of both enolate coordination isomers. Also, this effect can be explained by the higher charge density at the enolate‐O as compared to the ester‐O.

The strongly dominating reactive allocation of **2A** is close to the imidazolium unit since the other enolate face is shielded by the ligand (see Figure 3C and Supporting Information). Both control over the geometry of the reactive Cu‐enolate coordination isomer and over the trajectory of the attacking electrophile is thus key to the observed enantioselectivity. Other conformations, involving different orientations of the imidazolium unit, were found to be energetically disfavored.

To further investigate the origin of enantioselectivity, a noncovalent interaction (NCI) analysis was performed. It reveals a higher prevalence of weak attractive interactions in the major channel. These include CH‐π interactions between the aromatic systems of the mesityl group as well as the diamino backbone and a respective hydrogen of the enolate, which are enhanced in the major pathway due to a more favorable geometry. Additional details of the NCI analysis are provided in the Supporting Information.

Splitting of the overall reaction barrier into its enthalpic and entropic components reveals that the activation entropy is strongly dominant. At 300 K the enthalpic contributions were even found to be negative for the major path with Δ*H* ^‡^_major_ = −2.1 kJ mol^−1^ (Δ*H* ^‡^_minor_ = 11.9 kJ mol^−1^), thus lowering the overall barrier. In contrast, the entropic term −*T*Δ*S* ^‡^_major_ = 60.4 kJ mol^−1^ and −*T*Δ*S* ^‡^_minor_ = 60.1 kJ mol^−1^ has the major impact on the reaction rates for both channels. The energy difference between the corresponding pathways (ΔΔ*G* ^‡^ = 13.8 kJ mol^−1^) is thus attributed to the difference in the enthalpic term and slightly higher than expected from the experimental enantiomeric excess but remains within the DFT accuracy.

The energy of catalyst‐product complex **III** was found to be −54.6 kJ mol^−1^ in the major channel and −62.8 kJ mol^−1^ in the minor channel. Product release and binding of the next β‐ketoester **1a′** substrate in its enol form restore **I**. The overall reaction is exergonic by −82.4 kJ mol^−1^, which is why **I** **+** **3aA** is indicated at that energy in Figure 3C.

### 2.8 Kinetic Investigations

The empirical rate law was determined from kinetic data using variable time normalization analysis (VTNA) [67, 68, 69]. The optimal fit for the normalized time scale was obtained using Equation (1):

(1) $$r=k_{\mathrm{obs}}{\left[2A\right]}^{1}{\left[\text{DIPEA}\right]}^{0.05}{\left[C5\right]}^{0.85}{\left[\text{DIPEA}\cdot \text{HBr}\right]}^{-0.3}$$

The zero‐order dependence on [**1a**] and nearly zero‐order dependence on [DIPEA] indicate substrate saturation and rapid deprotonation. A zero‐order dependence on [**3aA**] rules out product inhibition, whereas the negative order for DIPEA·HBr shows side product inhibition, attributable to the solvent CD_2_Cl_2_ used for kinetic measurements, in which the side product remains soluble to ensure homogeneous conditions for NMR analysis. In contrast, under the developed standard conditions using EtOAc as solvent, the ammonium salt precipitates and should thus inhibit less than in CH_2_Cl_2_. The reaction orders of 1 for both [**2A**] and [**C5**] suggest that C─C bond formation constitutes part of the energetic span, involving a catalyst–enolate complex and the alkylating agent, supporting the mechanistic proposal obtained from the DFT calculations.

## 3 Conclusion

The bifunctional catalyst reported here operates through a structurally precisely organized Cu(II)‐enolate/imidazolium ion‐pair assembly, which is structurally anchored by H‐bonding and enables asymmetric enolate alkylation across an unusually broad electrophile scope. As revealed by Hammett analysis of the structure–activity relationship, this can be explained by a mechanistic continuum between more S_N_1‐like and S_N_2‐type reaction pathways. Targeted control experiments demonstrate the importance of the imidazolium moiety as a key structural element for largely increased activity and stereocontrol within a bifunctional catalyst species. EPR spectroscopy identified the Cu(II)‐enolate as the resting state under catalytic conditions. Detailed DFT calculations provide a molecular‐level description of the dominating structurally anchored enolate assembly. The reaction pathway computed for benzyl bromide as model electrophile shows that the transition state of the C─C‐bond forming step is turnover‐determining. The calculated transition states reproduce the sense and level of enantioinduction. Based on the calculations and the structure/activity relationship, subtle changes in C─X bond activation and ion‐pair separation arguably govern whether C─C bond formation proceeds through tighter or more dissociative transition structures, which are both compatible with the reported catalyst type. Besides the broad substrate scope, the reported catalyst type offers several practical advantages, such as its ease of preparation, high robustness, the operationally simple catalyst recovery, and multiple catalyst recyclability (10+). The combined experimental and computational insights reveal how hydrogen‐bond‐supported metal enolate stabilization by a C─H‐acidic azolium unit integrated within the catalyst framework is offering a general design principle for exceptionally high efficiency in catalytic asymmetric C─C‐bond formation.

## Author Contributions

**Wolfgang Frey**: investigation. **Johanna Haußmann**: investigation, writing – original draft, methodology, validation, visualization, writing – review and editing. **Michael Mistele**: investigation. **Dominik Hornung**: investigation. **René Peters**: conceptualization, investigation, funding acquisition, writing – original draft, methodology, validation, writing – review and editing, project administration, supervision, resources. **Joris van Slageren**: investigation, funding acquisition, writing – review and editing, methodology, validation, supervision. **Alexander Allgaier**: investigation, writing – original draft, writing – review and editing. **Johannes Kästner**: supervision, investigation, funding acquisition, methodology, validation, writing – review and editing. **Alexander Beck**: investigation, writing– original draft, methodology, validation, visualization, writing – review and editing.

## Conflicts of Interest

The authors declare no conflicts of interest.

## Supporting information




**Supporting File 1**: The authors have cited additional references within the Supporting Information [70, 71, 72, 73, 74, 75, 76, 77, 78, 79, 80, 81, 82, 83, 84, 85, 86, 87, 88, 89, 90, 91, 92, 93, 94, 95, 96, 97, 98, 99, 100, 101, 102, 103, 104, 105, 106, 107, 108, 109, 110, 111, 112, 113, 114, 115, 116, 117, 118, 119].

**Supporting File 2**: anie72578‐sup‐0002‐cif.zip.

## Data Availability

The data that supports the findings of this study are available in the supplementary material of this article.
