# Supplementary material for: Asymmetric α‐Alkylation With Activated and Unactivated Electrophiles by a Highly Productive and Recyclable Lewis Acid/Imidazolium Catalyst
Source: Angew Chem Int Ed Engl. 2026 May 11;65(27):e7069862. doi: 10.1002/anie.7069862 (PMC13327639; doi:10.1002/anie.7069862)
Supplement: Supplementary file 1 — Supporting File 1: The authors have cited additional references within the Supporting Information [70, 71, 72, 73, 74, 75, 76, 77, 78, 79, 80, 81, 82, 83, 84, 85, 86, 87, 88, 89, 90, 91, 92, 93, 94, 95, 96, 97, 98, 99, 100, 101, 102, 103, 104, 105, 106, 107, 108, 109, 110, 111, 112, 113, 114, 115, 116, 117, 118, 119]. [file ANIE-65-e7069862-s002.pdf]

Supporting Information

Asymmetric α-Alkylation with Activated and Unactivated Electrophiles by a Highly Productive and Recyclable Lewis Acid / Imidazolium Catalyst

Johanna Haußmann, Alexander Beck, Dominik Hornung, Michael Mistele, Alexander Allgaier, Wolfgang Frey, Joris van Slageren, Johannes Kästner and René Peters*

Contents

1 General Remarks 1

2 General Procedures 2

3 Precursor Synthesis 9

4 Ligand Synthesis 25

5 Complex Synthesis 40

6 Synthesis of Asymmetric Alkylation Products 58

7 Reaction Parameter Screening 84

8 Catalyst Stability 87

9 EPR Studies 90

10 Kinetic Investigations 95

11 Computational Studies 110

12 References 126

13 NMR Spectra 133

14 HPLC Data 187

15 GC Data 218

1 General Remarks

Unless noted otherwise, reactions were carried out under ambient conditions with magnetic stirring. Oxygen- and moisture-sensitive reactions were performed under N_2 (≈0.1 bar overpressure) in pre-dried glassware (15 h at 150 °C in a drying oven or 5 min under high vacuum at 630 °C). Solvents were removed under reduced pressure (rotary evaporator, 40 °C, 600–10 mbar) or at room temperature under N_2 flow. Chemicals were purchased from *ABCR*, *Acros Organics*, *Fluka*, *Merck*, *Sigma-Aldrich*, *TCI*, or *Alfa Aesar* and used without further purification unless stated. Petroleum ether (PE), ethyl acetate (EtOAc), dichloromethane (DCM), and methanol (MeOH) were used for column chromatography after distillation; dry solvents (DCM, Et_2O, THF, toluene, acetonitrile) were dried using a *Siemens MBRAUN MB SPS-800* system. Molecular sieves (3 Å or 4 Å, *ROTH*) were activated at 300 °C under high vacuum for 12 h. Substrates for catalysis were purified according to their physical state: liquids by distillation and solids by column chromatography. Diisopropylethylamine (DIPEA) was purified by distillation. Silica gel plates (*Merck* 60 F254) were used for TLC; UV-active compounds were detected at 254 nm, non-UV-active compounds were visualized with basic $KMnO_4$ solution. Column chromatography employed *Merck* silica gel (0.040–0.063 nm), with eluent composition specified in the respective reaction instructions. NMR spectra were recorded on *Bruker* spectrometers (*Avance 300*, *Ascend 400*, *Avance 500*, *Avance III HD*) at 300–700 MHz (^{1}H), 175 MHz (^{13}C), and 376 MHz (^{19}F). Measurement frequencies and solvents are indicated in brackets. Chemical shifts δ (ppm) are referenced to the solvent signal and coupling constants J (Hz) were determined using *Topspin 4.1.4*. Signal multiplicities are abbreviated as *br* (broad), *s* (singlet), *d* (doublet), *t* (triplet), *q* (quartet), *p* (quintet), *h* (sextet), *sept* (septet), and *m* (multiplet). Mass spectrometry experiments were performed using *Thermo Scientific* mass spectrometers (*Exactive Plus*, *Exactive GC*); only the most intense signals were reported as m/z. Elemental (CHN) analysis was performed using a *PerkinElmer 240 Analyzer*. Enantiomeric excess values were determined by HPLC analysis on chiral stationary phases at room temperature (either *VWR Elite LaChrom* system with *HITACHI L-2200* autosampler, *HITACHI L-2130* pump, and *HITACHI L-2400* UV/VIS detector, or *Knauer Clarity Chrom* system with *AS 6.1L* autosampler, *P 6.1L* pump, and *DAD2.1L* UV/VIS detector); column type, mobile phase, flow rate, and detection wavelength are provided in the respective reaction instructions. Enantiomeric excess values were also determined by GC (*HRGC Mega 2*, *Thermo Scientific Trace GC Ultra*) on chiral columns; column type, carrier gas, flow rate, and temperature program are provided in the respective reaction instructions. The absolute configuration of the catalytic products was determined by comparison of optical rotation and

HPLC retention times with those of literature-known compounds. For products not previously reported, the absolute configuration was assigned by analogy based on structural similarity. Reported yields for preligands, ligands, complexes, and catalytic products refer to the isolated products after workup and purification, unless otherwise noted. Given yields determined by ^{1}H-NMR were measured using mesitylene as internal standard. Optical rotations were measured on a *Krüss P8000-T80* polarimeter at λ = 589 nm in a 50 mm glass tube, with details on concentration and solvent provided in the respective experiments. IR spectra were recorded on a *Bruker Alpha FT-IR* spectrometer, reporting only the most intense signals. UV/Vis spectra were measured with a *Perkin Elmer Lambda 365* using tungsten and deuterium lamps. Melting points were determined with a *Stuart SMP 40* at a heating rate of 5 °C/min in open glass capillaries. Elemental analysis was performed on an *Elementar Micro Cube*.

2 General Procedures

2.1 General Procedure for *N*-Alkylation of Heteroaromatic Compounds (GP 1)

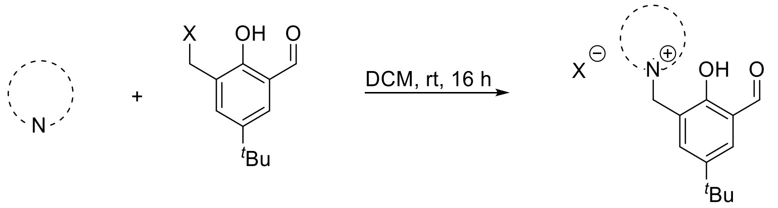

According to a literature procedure,[1] a solution of the respective halomethylated aldehyde (1.00 eq.) in DCM (20 mL/mmol) was added to a solution of the heteroaromatic compound (1.00 eq.) in DCM (10 mL/mmol) and the mixture was stirred at room temperature for 16 h. The reaction mixture was concentrated under reduced pressure, taken up in DCM (5 mL/mmol), and precipitated in Et_2O (100 mL/mmol). The resulting suspension was centrifuged, and the supernatant was decanted. After washing with Et_2O (100 mL/mmol), the product was dried under reduced pressure.

2.2 General Procedure for the Synthesis of Substituted Diamines (GP 2)

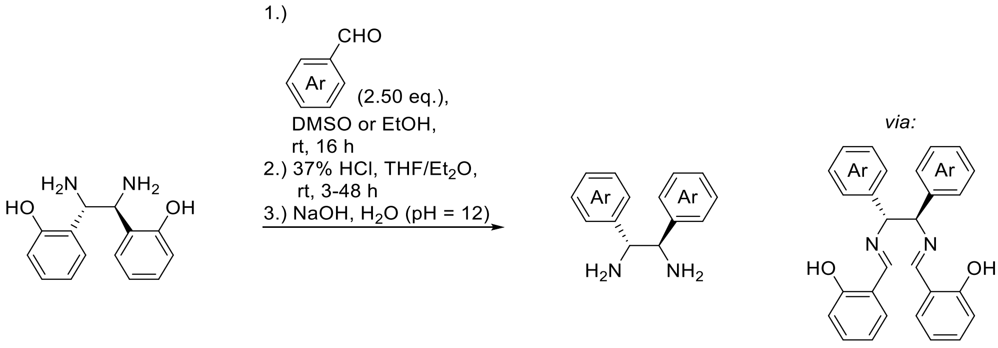

Step 1: Diaza-Cope Rearrangement

Method A: Following a literature procedure,[2] (*S*,*S*)-1,2-bis-(2-hydroxyphenyl)-1,2-diaminoethane (1.00 eq.) was dissolved in dry DMSO (5 mL/mmol). The corresponding aldehyde (2.50 eq.) was dissolved in dry DMSO (5 mL/mmol) and added dropwise to the diamine solution. The reaction mixture was stirred at room temperature overnight. Demineralized water was added (15 mL/mmol), and the aqueous phase was extracted with Et_2O (3 × 15 mL/mmol). The combined organic layers were washed with demineralized water (50 mL/mmol) and dried over Na_2SO_4. After removal of the solvent under reduced pressure, the diimine **DI** was dried under high vacuum.

Method B: Following a literature procedure,[2] a suspension of (*S*,*S*)-1,2-bis-(2-hydroxyphenyl)-1,2-diaminoethane (1.00 eq.) in dry ethanol (3 mL/mmol) was prepared, and the corresponding aldehyde (1 mL/mmol) was added. The resulting clear solution was stirred at room temperature until the diimine **DI** precipitated as a yellow solid. The solid was collected by filtration, washed with ethanol, and dried under high vacuum.

Step 2: Diimine Cleavage

Following a literature procedure,[2] the diimine was dissolved in THF/Et_2O (10 mL/mmol), and 37% HCl (0.3 mL/mmol) was added. The mixture was stirred at room temperature for 3-48 h, resulting in the precipitation of the dihydrochloride. The supernatant was decanted, and the solid was washed with THF/Et_2O (2 × 10 mL/mmol). For detailed solvent volumes and reaction times, see the respective literature procedures.

Step 3: Formation of the Free Amine

The dihydrochloride was dissolved in demineralized water (10 mL/mmol), following a literature procedure.[3] The pH was adjusted to 12 using 0.75 M NaOH solution. The aqueous phase was extracted with Et_2O (3 × 30 mL/mmol), and the combined organic layers were dried over Na_2SO_4, filtered, and concentrated under reduced pressure. The resulting diamine was dried under high vacuum.

2.3 General Procedure for the Synthesis of Chiral Sulfonamides (GP 3)

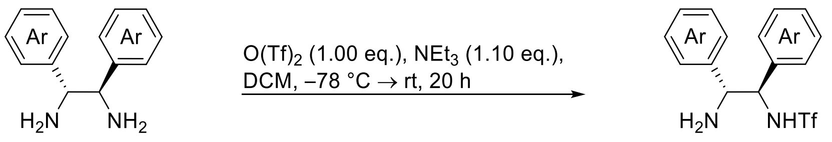

Following a literature procedure,[4] the diamine (1.00 eq.) was dissolved in dry DCM (50 mL/mmol) and cooled to −78 °C. A solution of trifluoromethanesulfonic anhydride (0.25–1.00 M in DCM, 1.00 eq.) was added dropwise over 2 h. The reaction mixture was stirred at room temperature overnight. Demineralized water (30 mL/mmol) and triethylamine (1.10 eq.) were added, the layers were separated, and the aqueous phase was extracted with DCM (3 × 30 mL/mmol). The combined organic layers were dried over $MgSO_4$, filtered, and concentrated under reduced pressure. The crude product was purified by column chromatography on silica gel (DCM/MeOH, 30:1).

2.4 General Procedure for Imine Condensation to Chiral Phenolimine Preligands (GP 4)

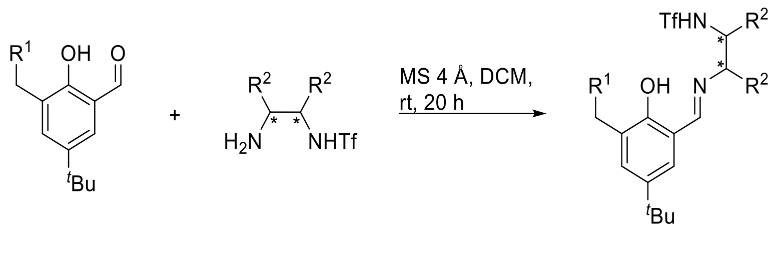

Following a literature procedure,[5] the corresponding aldehyde (1.00 eq.) and chiral amine (1.00-1.05 eq.) were placed in a pre-dried flask with MS 4 Å, dissolved in dry DCM (1 mL/mmol), and stirred overnight at room temperature under nitrogen atmosphere. The reaction solution was filtered through a pad of Celite® and the solvent was removed under reduced pressure. The solid was dissolved in DCM (0.2 mL/mmol) and precipitated in a solvent mixture of *n*-pentane/Et_2O (10:1, 5 mL/mmol) and washed twice with *n*-pentane/Et_2O (10:1,

5 mL/mmol). The phenolimine preligands **L** were dried under high vacuum and obtained as a fine powder. Deviating purification methods can be found in the corresponding synthesis procedures.

2.5 General Procedure for the Complexation (GP 5)[5]

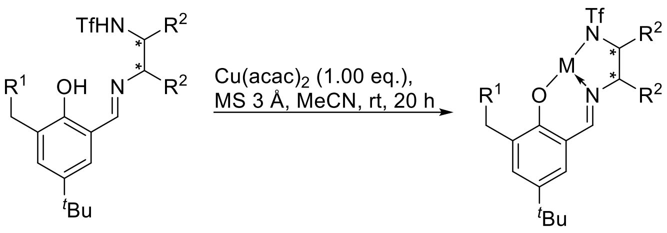

According to a literature procedure,[5] preligand (1.00 eq.), Cu(acac)$_2$ (1.00 eq.) and MS 3 Å were placed in a pre-dried flask and dissolved in dry MeCN (1 mL/mmol). The mixture was stirred for 20 h at room temperature and then filtered through a pad of Celite® and the filter cake was washed with DCM (3 × 1 mL/mmol). The solvent was removed under reduced pressure, the solid was dissolved in DCM (0.2 mL/mmol) and precipitated in *n*-pentane (5 mL/mmol). After decanting the supernatant, the solid was washed twice with *n*-pentane (5 mL/mmol) and the product was dried under high vacuum. The complexes were obtained as fine powders.

2.6 General Procedure for the Synthesis of Cyclopentenone Derived β-Ketoesters (GP 6)

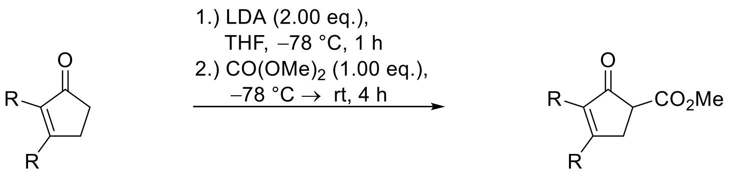

Diisopropylamine (2.00 eq.) was dissolved in dry THF (0.5 mL/mmol) and cooled to −78 °C. A solution of *n*-BuLi (2.5 M in hexane, 2.00 eq.) was added dropwise, and the mixture was stirred for 30 min at −78 °C. The respective cyclopentenone (1.00 eq.) was then added, and the mixture was stirred for 1 h at −78 °C. Dimethyl carbonate (1.00 eq.) was added, and the mixture was stirred for 4 h while gradually warming to room temperature. The reaction mixture was quenched with saturated NH_4Cl solution (10 mL/mmol) and extracted with DCM (3 × 10 mL/mmol), dried over Na_2SO_4, filtered, and concentrated under reduced pressure. Column chromatography on silica gel (PE/EtOAc, 5:1) afforded the desired products.

2.7 General Procedure for the Synthesis of Alkyl Bromides (GP 7)

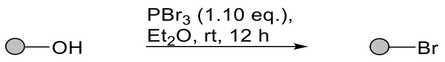

Following a literature procedure,[6] PBr_3 (1.10 eq.) was added dropwise at 0 °C to a solution of the corresponding primary alcohol (1.00 eq.) in Et_2O (2.5 mL/mmol). The reaction mixture was allowed to warm to room temperature and stirred overnight. The reaction mixture was diluted with Et_2O (10 mL/mmol) and quenched by careful addition of saturated $NaHCO_3$ solution (5 mL/mmol) at 0 °C. The layers were separated, and the aqueous phase was extracted with Et_2O (2 × 10 mL/mmol). The combined organic layers were dried over Na_2SO_4, and the solvent was removed under reduced pressure. The alkyl bromides were used directly in subsequent catalytic reactions without further purification.

2.8 General Procedure for the Synthesis of Alkyl Triflates (GP 8)

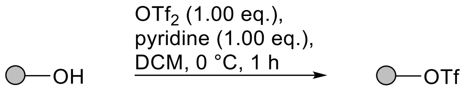

Following a literature procedure,[7] a solution of the corresponding primary alcohol (1.00 eq.) and pyridine (1.00 eq.) in dry DCM (3 mL/mmol) was added dropwise to a stirred solution of triflic anhydride (1.00 eq.) in dry DCM (3 mL/mmol) at 0 °C under inert atmosphere. The solution was maintained at 0 °C and stirred for 1 h. The reaction mixture was washed with ice-cold demineralized water and dried over Na_2SO_4. The solvent was removed under reduced pressure at room temperature. The alkyl triflates were used directly in subsequent catalytic reactions without further purification.

2.9 General Procedure for the Catalytic Asymmetric Alkylation using Alkyl Halides (GP 9)

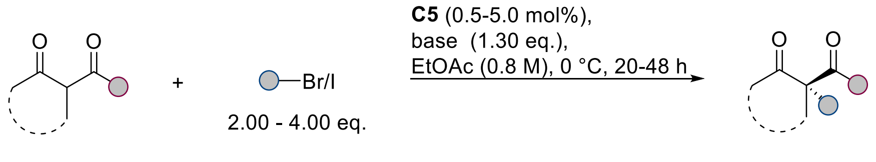

The 1,3-dicarbonyl substrate (1.00 eq.) and catalyst **C5** (Y mol%) were dissolved in EtOAc (0.8 M) and the solution was cooled to 0 °C. The base (1.30 eq.) was added, followed by the respective alkyl halide (2.00-4.00 eq.). The reaction was then stirred for 20-48 h at 0 °C. The reaction was quenched with 1 M HCl solution (0.3 mL), the layers were separated, and the

aqueous phase was extracted with DCM (3 × 1 mL). The combined organic layers were dried over Na_2SO_4, filtered, and the solvent was removed under reduced pressure. Purification by column chromatography on silica gel (PE/EtOAc 10:1) afforded the desired products. Alternative workup and purification procedures are described in the corresponding experimental sections.

2.10 General Procedure for the Catalytic Asymmetric Alkylation using Alkyl Triflates (GP 10)

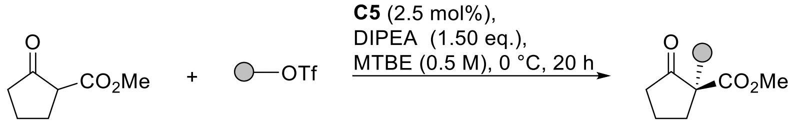

Catalyst **C5** (2.5 mol%) was placed in a Schlenk tube and dissolved in dry MTBE (0.25 mL). The β-ketoester (1.00 eq.) was added, and the solution was cooled to 0 °C. DIPEA (1.50 eq.) followed by a stock solution of the respective alkyl triflate in MTBE (0.2 mL, 1.50 eq.) were added. The reaction was then stirred for 20 h and quenched with 1 M HCl solution (0.3 mL), the layers were separated, and the aqueous phase was extracted with DCM (3 × 1 mL). The combined organic layers were dried over Na_2SO_4, filtered, and the solvent was removed under reduced pressure. Purification by column chromatography on silica gel (PE/EtOAc 10:1) afforded the desired products. Alternative workup and purification procedures are described in the corresponding experimental sections.

2.11 General Procedure for the Synthesis of Racemic Alkylated β-Ketoesters (GP 11)

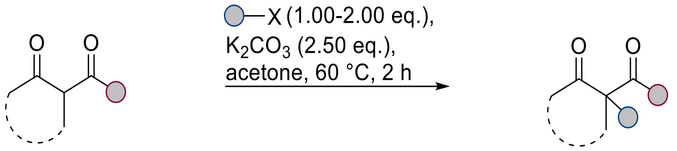

Following a modified literature procedure,[8] K_2CO_3 (2.50 eq.) was placed in a pressure tube and suspended in acetone (2 mL/mmol). The β-ketoester and the alkylating agent (2.00 eq. for low-boiling alkylating agents, otherwise 1.00 eq.) were then added, and the mixture was stirred at 60 °C overnight. The reaction mixture was diluted with ethyl acetate, filtered through Celite® and silica gel, and concentrated under reduced pressure.

2.12 General Procedure for Catalyst Recycling (GP 12)

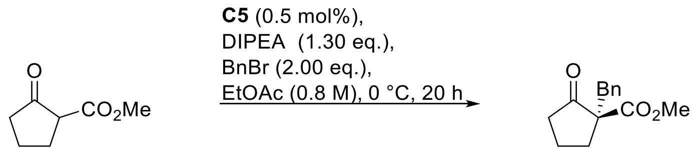

The β-ketoester (37.5 µL, 0.30 mmol, 1.00 eq.) and catalyst **C5** (1.21 mg, 1.5 µmol, 0.5 mol%) were dissolved in ethyl acetate (0.8 M) and the solution was cooled to 0 °C. DIPEA (68.5 µL, 0.39 mmol, 1.30 eq.) was added, followed by benzyl bromide (71.7 µL, 0.60 mmol, 2.00 eq.) and the reaction mixture was stirred at 0 °C for 20 h. The reaction mixture was diluted with PE/EtOAc (2:1, 2 mL) and filtered over Celite® to remove the precipitated DIPEA · HBr. The filtrate was diluted with more PE and filtered over silica to separate the catalyst from the product and unreacted starting materials. The yield was determined from the crude mixture by ^{1}H-NMR spectroscopy using mesitylene as an internal standard. The catalyst was then eluted from the silica using DCM/MeOH (10:1). The solvent was removed under reduced pressure, and the recovered catalyst dissolved in ethyl acetate (2 mL) and transferred to a reaction vial. The solvent was removed by using a stream of N_2, and the catalyst was further dried under high vacuum. The recycled catalyst was then reused by adding fresh substrates to perform subsequent reaction runs.

3 Precursor Synthesis

3.1 5-(*tert*-Butyl)-3-(chloromethyl)-2-hydroxybenzaldehyde 5

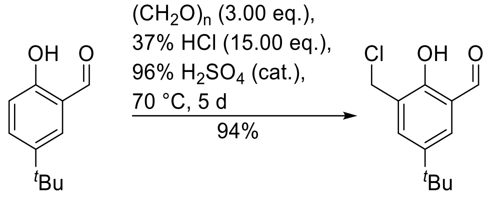

4 **5**

According to a literature procedure,[9] aldehyde **4** (9.40 g, 52.74 mmol, 1.00 eq.), paraformaldehyde (4.76 g, 158.22 mmol, 3.00 eq.), 37% HCl (65.8 mL, 791.1 mmol, 15.00 eq.), and 96% H_2SO_4 (16 drops) were heated under reflux for 5 d. After cooling to room temperature, the reaction mixture was dissolved in demineralized water (40 mL) and extracted with DCM (3 × 50 mL). The combined organic layers were dried over Na_2SO_4, and the solvent was removed under reduced pressure. The product (11.20 g, 49.40 mmol, 94%) was obtained as a brown oil.

$C_{12}H_{15}ClO_2$, MW: 226.70 g/mol. **^{1}H-NMR (400 MHz, $CDCl_3$)**: δ = 11.27 (*s*, 1 H, Ar-O*H*), 9.90 (*s*, 1 H, Ar-C*H*O), 7.68 (*d*, *J* = 2.3 Hz, 1 H, Ar-*H*), 7.52 (*d*, *J* = 2.4 Hz, 1 H, Ar-*H*), 4.70 (*s*, 2 H, Cl-C*H*$_2$-Ar), 1.34 (*s*, 9 H, Ar-C(C*H*$_3$)$_3$) ppm.

The analytical data are consistent with the literature.[9]

3.2 3-(Azidomethyl)-5-(*tert*-butyl)-2-hydroxybenzaldehyde S2

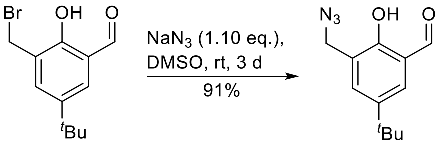

S1 **S2**

According to a literature procedure,[10] NaN_3 (1.88 g, 28.95 mmol, 1.10 eq.) was placed under a nitrogen atmosphere and dissolved in dry DMSO (50 mL). The bromomethylated aldehyde **S1** (7.14 g, 26.32 mmol, 1.00 eq.) was dissolved in dry DMSO (25 mL) and added dropwise to the NaN_3-solution. The reaction mixture was stirred at room temperature for 3 d. The mixture was then diluted with water (50 mL) and extracted with DCM (3 × 50 mL). The combined organic layers were washed with water (50 mL), dried over $MgSO_4$, filtered, and concentrated

under reduced pressure. Purification by column chromatography on silica gel (PE/Et_2O, 4:1) afforded **S2** (5.61 g, 24.04 mmol, 91%) as a light-brown solid.

$C_{12}H_{15}N_3O_2$, MW: 233.27 g/mol. **^{1}H-NMR (300 MHz, $CDCl_3$):** δ = 11.21 (*s*, 1 H, Ar-O*H*), 9.91 (*s*, 1 H, Ar-C*H*O), 7.55 (*dd*, *J* = 2.3, 15.1 Hz, 2 H, Ar-*H*), 4.45 (*s*, 2 H, Ar-CH_2-N_3), 1.34 (*s*, 9 H, Ar-C(CH_3)$_3$) ppm.

The analytical data are consistent with the literature.[10]

3.3 5-(*tert*-Butyl)-2-hydroxy-3-((4-mesityl-1*H*-1,2,3-triazol-1-yl)methyl)benzaldehyde S3

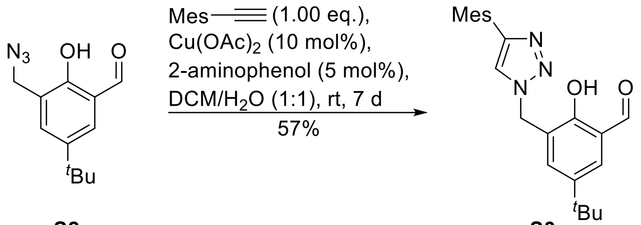

S2 **S3**

Following a literature procedure,[5] azide **S2** (1.00 g, 4.29 mmol, 1.00 eq.), copper(II) acetate (77.9 mg, 0.43 mmol, 10 mol%), and 2-aminophenol (23.4 mg, 0.21 mmol, 5 mol%) were placed in a flask and suspended in DCM/H_2O (1:1, 2.5 mL). Mesitylacetylene (0.70 mL, 4.29 mmol, 1.00 eq.) was then added, and the reaction mixture was stirred at room temperature for 7 d. The layers were separated, and the aqueous phase was extracted with DCM (3 × 10 mL). The combined organic layers were dried over Na_2SO_4, filtered, and concentrated under reduced pressure. Purification by column chromatography on silica gel (PE/Et_2O, 4:1) afforded triazole **S3** (0.93 g, 2.45 mmol, 57%) as a white solid.

$C_{23}H_{27}N_3O_2$, MW: 337.49 g/mol. **^{1}H-NMR (300 MHz, $CDCl_3$):** δ = 11.31 (*s*, 1 H, Ar-O*H*), 9.92 (*s*, 1 H, Ar-C*H*O), 7.57 (*s*, 1 H, H_{Trz}), 7.55 (*d*, *J* = 2.4 Hz, 1 H, Ar-*H*), 7.48 (*d*, *J* = 2.4 Hz, 1 H, Ar-*H*), 6.92 (*s*, 2 H, H_{Mes}), 5.68 (*s*, 2 H, Ar-CH_2-N_{Trz}), 2.30 (*s*, 3 H, *p*-Mes-CH_3), 2.09 (*s*, 6 H, *o*-Mes-(CH_3)$_2$), 1.29 (*s*, 9 H, Ar-C(CH_3)$_3$) ppm.

The analytical data are consistent with the literature.[5]

3.4 1-(2,6-Diisopropylphenyl)-1*H*-imidazole S5

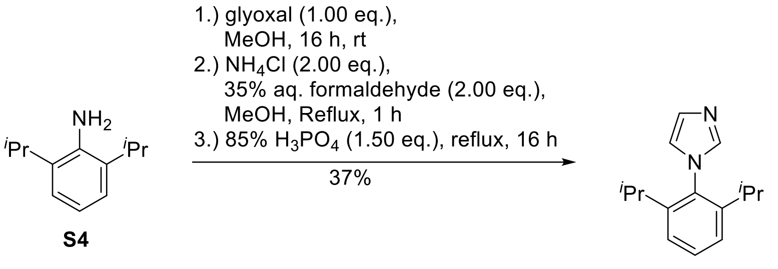

S4 **S5**

Following a literature procedure,[11] amine **S4** (1.60 g, 0.01 mol, 1.00 eq.) was dissolved in methanol (18 mL), and 40% aqueous glyoxal solution (1.04 mL, 0.02 mol, 1.00 eq.) was added. The mixture was stirred at room temperature overnight. Ammonium chloride (1.00 g, 0.02 mol, 2.00 eq.), 35% aqueous formaldehyde solution, and methanol (35 mL) were then added, and the mixture was stirred under reflux for 1 h. Subsequently, 85% phosphoric acid (0.83 mL, 0.02 mmol, 1.50 eq.) was added, and the mixture was stirred under reflux overnight. The solvent was removed under reduced pressure, and ice-cold water (300 mL) was added to the residue. The pH of the aqueous solution was adjusted to 9 using 40% aqueous KOH solution, and the mixture was extracted with Et_2O (5 × 100 mL). The combined organic layers were washed with water (200 mL) and saturated NaCl solution (200 mL), dried over Na_2SO_4, filtered, and concentrated under reduced pressure. The crude product was purified by column chromatography on silica gel (PE/Et_2O, 5:1). Imidazole **S5** was obtained as a beige solid (0.80 g, 3.30 mmol, 37%).

$C_{15}H_{20}N_2$, MW: 228.34 g/mol. **^{1}H-NMR (300 MHz, $CDCl_3$)**: δ = 7.44 (*br*, 1 H, NC*H*N), 7.40 (*d*, J = 7.7 Hz, 1 H, Ar-*H*), 7.40 (*d*, J = 7.5 Hz, 3 H, Ar-*H*), 6.91 (*br*, 1 H, Ar-*H*), 2.37 (*sept.* J = 6.7 Hz, 2 H, C*H*(CH_3)$_2$), 1.10 (*d*, J = 6.7 Hz, CH(C*H*$_3$)$_2$) ppm.

The analytical data are consistent with the literature.[11]

3.5 1-(5-(*tert*-Butyl)-3-formyl-2-hydroxybenzyl)-3-ethyl-4-mesityl-1*H*-1,2,3-triazol-3-ium hexafluorophosphate(V) S6

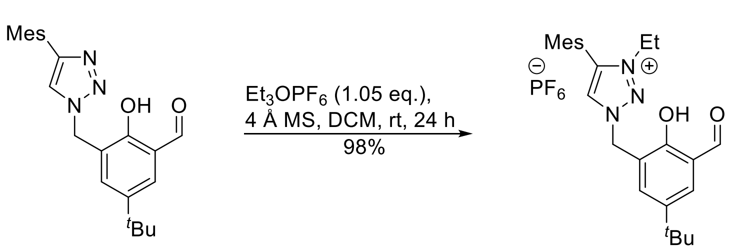

S3 **S6**

Following a literature procedure,[5] triazole **S3** (443.9 mg, 1.18 mmol, 1.00 eq.), the triethyloxonium hexafluorophosphate (206.4 mg, 1.24 mmol, 1.05 eq.), and 4 Å molecular sieves were placed in a pre-dried flask and dissolved in dry DCM (0.5 mLl). The reaction mixture was stirred at room temperature for 24 h. The mixture was filtered over Celite®, and the filtrate was washed with water (5 mL). The aqueous phase was extracted with DCM (3 × 5 mL/mmol), and the combined organic layers were dried over Na_2SO_4, filtered, and concentrated under reduced pressure. The residue was dissolved in DCM (1 mL) and precipitated in *n*-pentane (30 mL). The product **S6** (634.3 mg, 1.15 mmol, 98%) was dried under high vacuum and obtained as a fine powder.

$C_{25}H_{32}F_6N_3O_2P$, MW: 551.51 g/mol. **^{1}H-NMR (400 MHz, $CDCl_3$):** δ = 11.28 (*s*, 1 H, Ar-O*H*), 9.92 (*s*, 1 H, Ar-C*H*O), 8.19 (*s*, 1 H, H_{Trz}), 8.03 (*d*, *J* = 2.6 Hz, 1 H, Ar-*H*), 7.68 (*d*, *J* = 2.6 Hz, 1 H, Ar-*H*), 7.00 (*s*, 2 H, H_{Mes}), 5.97 (*s*, 2 H, Ar-CH_2-N_{Trz}), 4.21 (*q*, *J* = 7.4 Hz, 2 H, N-CH_2-CH_3), 2.34 (*s*, 3 H, *p*-Mes-CH_3), 2.02 (*s*, 6 H, *o*-Mes-(CH_3)$_2$), 1.50 (*t*, *J* = 7.4 Hz, 3 H, N-CH_2-CH_3), 1.38 (*s*, 9 H, Ar-C(CH_3)$_3$) ppm. **^{19}F-NMR (376 MHz, $CDCl_3$):** δ = 73.04 (*d*, *J* = 712.5 Hz, 6 F, $(PF_6)^-$) ppm. **^{31}P-NMR (162 MHz, $CDCl_3$):** δ = –144.53 (*sept*, *J* = 613.1 Hz, 1 P, $(PF_6)^-$) ppm.

The analytical data are consistent with the literature.[5]

3.6 3-(5-(*tert*-Butyl)-3-formyl-2-hydroxybenzyl)-1-methyl-1*H*-imidazol-3-ium chloride S7

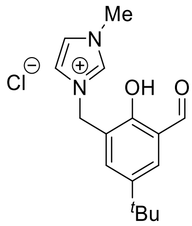

S7

The imidazolium salt **S7** was synthesized following **GP 1** using 1-methylimidazole (51.5 mg, 0.63 mmol, 1.00 eq.) and the chloromethylated aldehyde **5** (142.2 mg, 0.63 mmol, 1.00 eq.). After workup, the product **S7** (177.7 mg, 0.58 mmol, 92%) was obtained as a white solid.

$C_{16}H_{21}ClN_2O_2$, MW: 308.81 g/mol. **^{1}H-NMR (400 MHz, $CDCl_3$):** δ = 11.42 (*s*, 1 H, Ar-O*H*), 11.22 (*s*, 1 H, NC*H*N), 9.90 (*s*, 1 H, Ar-C*H*O), 8.36 (*d*, *J* = 2.3 Hz, 1 H, Ar-*H*), 7.60 (*d*, *J* = 2.3 Hz, 1 H, Ar-*H*), 7.53 (*t*, *J* = 1.7 Hz, 1 H, C$H_{imidazole}$), 7.12 (*t*, *J* = 1.6 Hz, 1 H, C$H_{imidazole}$), 5.68 (*s*, 2 H, Ar-CH_2-N), 4.01 (*s*, 3 H, N-CH_3), 1.36 (*s*, 9 H, Ar-C(CH_3)$_3$) ppm.

The analytical data are consistent with the literature.[12]

3.7 3-(5-(*tert*-Butyl)-3-formyl-2-hydroxybenzyl)-1-(2,6-diisopropylphenyl)-1*H*-imidazol-3-ium chloride S8

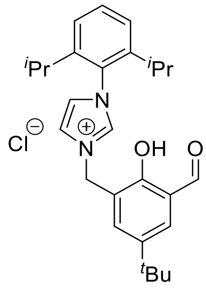

S8

The imidazolium salt **S8** was synthesized following **GP 1** using imidazole **S5** (69.1 mg, 0.30 mmol, 1.00 eq.) and the chloromethylated aldehyde **5** (68.6 mg, 0.30 mmol, 1.00 eq.). After workup, the product **S8** (108.4 mg, 0.24 mmol, 79%) was obtained as a white solid.

$C_{27}H_{35}ClN_2O_2$, MW: 455.04 g/mol. **MP:** 182 °C. **^{1}H-NMR (300 MHz, $CDCl_3$):** δ = 11.45 (*s*, 1 H, Ar-O*H*), 10.66 (*s*, 1 H, Ar-C*H*O), 9.92 (*s*, 1 H, NC*H*N), 8.65 (*d*, *J* = 1.5 Hz, 1 H, Ar-*H*), 8.03 (*s*, 1 H, Ar-*H*), 7.59 (*d*, *J* = 2.0 Hz, 1 H, Ar-*H*), 7.50 (*t*, *J* = 7.8 Hz, 1 H, Ar-*H*), 7.28 (*s*, 1 H, Ar-*H*), 7.25 (*s*, 1 H, Ar-*H*), 7.06 (*s*, 1 H, Ar-*H*), 6.13 (s, 2 H, *N*-CH_2-Ar), 2.21 (*sept*, *J* = 1.5 Hz, 2 H, C*H*(CH_3)$_2$), 1.34 (*s*, 9 H, Ar-C(CH_3)$_3$), 1.18 (*d*, *J* = 6.8 Hz, 6 H, CH(CH_3)$_2$), 1.09 (*d*, *J* = 6.8 Hz, 6 H, CH(CH_3)$_2$) ppm. **^{13}C-NMR (175 MHz, $CDCl_3$):** δ = 197.0, 157.5, 145.5, 144.7, 139.14, 139.11, 138.2, 132.0, 131.3, 130.4, 124.8, 123.7, 123.6, 122.5, 120.4, 47.6, 34.7, 31.4, 28.8, 24.5, 24.2 ppm. **IR ($CDCl_3$):** $\tilde{\nu}$ = 3872, 2964, 2870, 2177, 1676, 1651, 1619, 1560, 1543, 1464, 1386, 1366, 1314, 1277, 1220, 1200, 1182, 1116, 1069, 1010, 958, 926, 828, 805, 759, 729, 672, 645, 612, 456 cm^{-1}. **HRMS (ESI) *m/z*:** calculated for $C_{27}H_{35}N_2O_2$ [M-Cl]$^+$: 419.2693; measured 419.2681.

3.8 3-(5-(*tert*-Butyl)-3-formyl-2-hydroxybenzyl)-1-mesityl-1*H*-imidazol-3-ium chloride S9

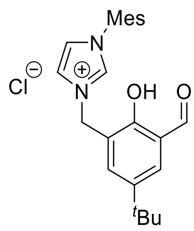

S9

The imidazolium salt **S9** was synthesized following **GP 1** using *N*-mesitylimidazole (99.3 mg, 0.53 mmol, 1.00 eq.) and the chloromethylated aldehyde **5** (120.9 mg, 0.53 mmol, 1.00 eq.). After workup, the product **S9** (194.2 mg, 0.47 mmol, 88%) was obtained as a white solid.

$C_{24}H_{29}ClN_2O_2$, MW: 412.92 g/mol. **^{1}H-NMR (400 MHz, $CDCl_3$):** δ = 11.45 (*s*, 1 H, NC*H*N), 10.87 (*s*, 1 H, Ar-O*H*), 9.92 (*s*, 1 H, Ar-C*H*O), 8.62 (*d*, *J* = 2.3 Hz, 1 H, Ar-*H*), 7.85 (*t*, *J* = 1.6 Hz, 1 H, $CH_{Imidazol}$), 7.60 (*d*, *J* = 2.5 Hz, 1 H, Ar-*H*), 7.03 (*t*, *J* = 1.6 Hz, 1 H, $CH_{Imidazol}$), 6.98 (*s*, 2 H, Ar-H_{Mes}), 6.06 (*s*, 2 H, Ar-CH_2-N), 2.32 (*s*, 3 H, *p*-Mes-CH_3), 2.03 (*s*, 6 H, *o*-Mes-CH_3), 1.35 (*s*, 9 H, Ar-C(CH_3)$_3$) ppm.

The analytical data are consistent with the literature.[12]

3.9 3-(5-(*tert*-Butyl)-3-formyl-2-hydroxybenzyl)-1-mesityl-1*H*-imidazol-3-ium bromide S10

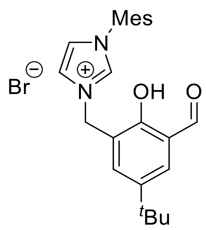

S10

The imidazolium salt **S10** was synthesized following **GP 1** using *N*-mesitylimidazole (60.0 mg, 0.32 mmol, 1.00 eq.) and the bromomethylated aldehyde **S1** (87.3 mg, 0.32 mmol, 1.00 eq.). After workup, the product **S10** (124.0 mg, 0.27 mmol, 84%) was obtained as a white solid.

$C_{24}H_{29}BrN_2O_2$, MW: 457.41 g/mol. **^{1}H-NMR (300 MHz, $CDCl_3$):** δ = 11.46 (*s*, 1 H, Ar-O*H*), 10.59 (*s*, 1 H, Ar-C*H*O), 9.91 (*s*, 1 H, NC*H*N), 8.66 (*d*, *J* = 2.2 Hz, 1 H, Ar-*H*), 7.88 (*t*, *J* = 1.2 Hz, 1 H, Ar-*H*), 7.61 (*d*, *J* = 2.2 Hz, 1 H, Ar-*H*), 7.05 (*t*, *J* = 0.8 Hz, 1 H, Ar-*H*), 6.98 (*s*, 2 H, Ar-*H*), 6.06 (*s*, 2 H, Ar-CH_2-N), 2.32 (s, 3 H, *p*-Mes-CH_3), 2.04 (*s*, 6 H, *o*-Mes-CH_3), 1.36 (*s*, 9 H, Ar-C(CH_3)$_3$) ppm.

The analytical data are consistent with the literature.[13]

3.10 3-(5-(*tert*-Butyl)-3-formyl-2-hydroxybenzyl)-1-mesityl-1*H*-imidazol-3-ium iodide S11

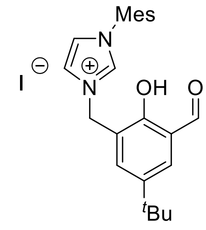

S11

The imidazolium salt **S11** was synthesized following **GP 1** using *N*-mesitylimidazole (50.7 mg, 0.27 mmol, 1.00 eq.) and 5-(*tert*-Butyl)-3-(bromomethyl)-2-hydroxybenzaldehyde (86.6 mg, 0.27 mmol, 1.00 eq.). After workup, the product **S11** (132.4 mg, 0.26 mmol, 96%) was obtained as a white solid.

$C_{24}H_{29}IN_2O_2$, MW: 504.41 g/mol. **MP:** 220-225 °C. **^{1}H-NMR (300 MHz, $CDCl_3$):** δ = 11.46 (*s*, 1 H, Ar-O*H*), 10.16 (*s*, 1 H, Ar-C*H*O), 9.92 (*s*, 1 H, NC*H*N), 8.61 (*d*, *J* = 2.3 Hz, 1 H, Ar-*H*), 7.88 (*s*, 1 H, Ar-*H*), 7.62 (*d*, *J* = 2.3 Hz, 1 H, Ar-*H*), 7.06 (*t*, *J* = 1.1 Hz, 1 H, Ar-*H*), 7.00 (*s*, 2 H, Ar-*H*), 6.03 (*s*, 2 H, *N*-CH_2-Ar), 2.34 (*s*, 3 H, *p*-Mes-CH_3), 2.07 (*s*, 6 H, *o*-Mes-CH_3), 1.37 (*s*, 9 H, Ar-C(CH_3)$_3$) ppm. **^{13}C-NMR (175 MHz, $CDCl_3$):** δ = 197.1, 157.7, 144.8, 141.7, 137.9, 137.7, 134.4, 131.7, 130.6, 130.1, 123.9, 122.7, 121.7, 120.3, 53.6, 48.1, 34.9, 31.5, 21.2, 17.9 cm^{-1}. **IR ($CDCl_3$):** $\tilde{\nu}$ = 3063, 3038, 2957, 2926, 2859, 2244, 2206, 2178, 2150, 2098, 2056, 1968, 1930, 1650, 1619, 1547, 1526, 1477, 1444, 1384, 1365, 1316, 1276, 1222, 1200, 1160, 1068, 1034, 1010, 919, 855, 828, 729, 669, 648, 631, 578, 517, 450, 411 cm^{-1}. **HRMS (ESI) *m/z*:** calculated for $C_{24}H_{29}N_2O_2$ [M-I]$^+$: 377.2224; measured 377.2209.

3.11 1-(5-(*tert*-Butyl)-3-formyl-2-hydroxybenzyl)pyridin-1-ium chloride S12

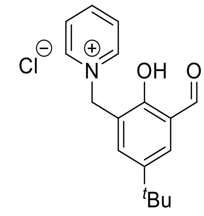

S12

The pyridinium salt **S12** was synthesized following **GP 1** using pyridine (0.15 mL, 0.81 mmol, 1.00 eq.) and the chloromethylated aldehyde **5** (150.6 mg, 0.81 mmol, 1.00 eq.). After workup, the product **S12** (253.3 mg, 0.57 mmol, 71%) was obtained as a white solid.

$C_{17}H_{20}ClNO_2$, MW: 305.80 g/mol. **MP:** 237 °C. **^{1}H-NMR (400 MHz, DMSO-d$_6$):** δ = 11.24 (*s*, 1 H, Ar-O*H*), 10.12 (*s*, 1 H, Ar-C*H*O), 9.19 (*d*, *J* = 6.0 Hz, 2 H, Ar-*H*), 8.61 (*t*, *J* = 7.8 Hz, 1 H, Ar-*H*), 8.16 (*t*, *J* = 7.1 Hz, 2 H, Ar-*H*), 8.11 (*d*, *J* = 2.3 Hz, 1 H, Ar-*H*), 7.86 (*d*, *J* = 2.3 Hz, 1 H, Ar-*H*), 5.93 (*s*, 2 H, Ar-CH_2-N), 1.31 (*s*, 9 H, Ar-C(CH_3)$_3$) ppm. **^{13}C-NMR (175 MHz, DMSO-d$_6$):** δ = 195.6, 156.8, 145.9, 145.0, 142.8, 136.2, 130.0, 128.1, 122.3, 121.8, 59.2, 34.1, 31.0 ppm. **IR (solid):** $\tilde{\nu}$ = 3023, 2992, 2953, 2868, 1650, 1632, 1620, 1502, 1484, 1426, 1384, 1362, 1272, 1225, 1203, 1161, 1148, 1064, 1026, 1012, 952, 928, 919, 863, 831, 820, 785, 760, 741, 717, 682, 661, 622, 569, 525, 516 cm^{-1}. **HRMS (ESI) *m/z*:** calculated for $C_{17}H_{20}NO_2$ [M-Cl]$^+$: 270.1489; measured 270.1476.

3.12 3-(5-(*tert*-Butyl)-3-formyl-2-hydroxybenzyl)-1-mesityl-2-methyl-1*H*-imidazol-3-ium chloride S42

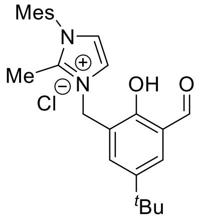

S42

The imidazolium salt **S42** was synthesized following **GP 1** using 1-mesityl-2-methyl-1*H*-imidazole (102.9 mg, 0.51 mmol, 1.00 eq.) and the chloromethylated aldehyde **5** (116.5 mg, 0.51 mmol, 1.00 eq.). After workup, the product **S42** (219.3 mg, 0.51 mmol, >99%) was obtained as a white solid.

$C_{25}H_{31}ClN_2O_2$, MW: 426.99 g/mol. **MP:** 199-206 °C. **^{1}H-NMR (300 MHz, CDCl$_3$):** δ = 11.34 (*s*, 1 H, Ar-O*H*), 9.92 (*s*, 1 H, Ar-C*H*O), 8.48 (*d*, *J* = 2.6 Hz, 1 H, Ar-*H*), 7.95 (*d*, *J* = 1.8 Hz, 1 H, Ar-*H*), 7.62 (*d*, *J* = 2.6 Hz, 1 H, Ar-*H*), 7.04 (*s*, 2 H, Ar-*H*), 6.98 (*d*, *J* = 2.6 Hz, 1 H, Ar-*H*), 5.92 (*s*, 2 H, Ar-CH_2-N), 2.68 (*s*, 3 H, C-CH_3), 2.36 (*s*, 3 H, *p*-Mes-CH_3), 2.00 (*s*, 6 H, *o*-Mes-CH_3), 1.39 (*s*, 9 H, Ar-C(CH_3)$_3$) ppm. **^{13}C-NMR (175 MHz, CDCl$_3$):** δ = 197.0, 157.8, 145.4, 144.4, 141.7, 138.0, 135.1, 131.1, 130.2, 130.1, 123.6, 121.6, 121.2, 120.4, 66.0, 48.5, 34.6, 31.4, 21.3, 17.7, 15.4, 10.7 ppm. **IR (CDCl$_3$):** $\tilde{\nu}$ = 3102, 2961, 2921, 2866, 1675, 1650, 1607, 1513, 1483, 1464, 1365, 1276, 1251, 1218, 1175, 1148, 1011, 973, 854, 832, 791, 728, 684, 665, 636, 608, 593, 578, 568, 547, 529, 519, 485, 475, 458, 448, 437, 424 cm^{-1}. **HRMS (ESI) *m/z*:** calculated for $C_{25}H_{31}N_2O_2$ [M-Cl]$^+$: 391.2380; measured 391.2369.

3.13 3-Benzyl-5-(*tert*-butyl)-2-hydroxybenzaldehyde S45

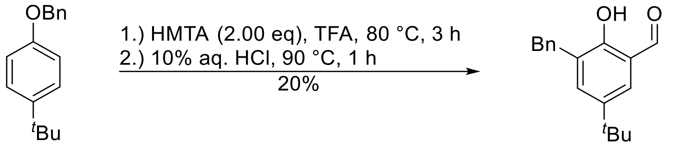

S44 **S45**

Following a literature procedure,[14] benzyl aryl ether **S44** (244.0 mg, 1.02 mmol, 1.00 eq.) was dissolved in trifluoroacetic acid (3.5 mL) under a nitrogen atmosphere, and hexamethylenetetramine (284.6 mg, 2.03 mmol, 2.00 eq.) was added. The reaction mixture was stirred at 80 °C for 3 h. After cooling to room temperature, aqueous HCl (10%, 3.5 mL) was added, and the mixture was stirred at 90 °C for 1 h. The reaction mixture was cooled to room temperature and extracted with EtOAc (2 × 10 mL). The combined organic layers were washed with saturated aqueous NaCl solution (10 mL), dried over anhydrous Na_2SO_4, filtered, and concentrated under reduced pressure. Purification by column chromatography on silica gel (PE/DCM 1:1) afforded product **S45** as a colorless oil (55.8 mg, 0.21 mmol 20%).

$C_{18}H_{20}O_2$, MW: 268.36 g/mol. **^{1}H-NMR (300 MHz, $CDCl_3$):** δ = 11.18 (*s*, 1 H, Ar-O*H*), 9.90 (*s*, 1 H, Ar-C*H*O), 7.48-7.38 (*m*, 2 H, Ar-*H*), 7.37-7.28 (*m*, 2 H, Ar-*H*), 7.28-7.16 (*m*, 3 H, Ar-*H*), 4.04 (*s*, 2 H, C*H*$_2$), 1.31 (*s*, 9 H, Ar-C(C*H*$_3$)$_3$) ppm.

The analytical data are consistent with the literature.[14]

3.14 3-Benzyl-1-ethyl-1*H*-imidazol-3-ium bromide C11

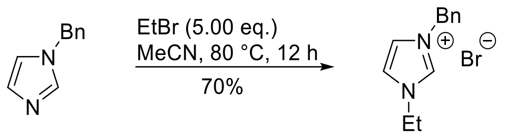

S13 **C11**

Following a literature procedure,[15] 1-benzylimidazole **S13** (100.0 mg, 0.63 mmol, 1.00 eq.) was placed in a pressure tube and dissolved in MeCN (2 mL). Ethyl bromide (136 µL, 3.16 mmol, 5.00 eq.) was added, and the reaction mixture was stirred at 80 °C for 12 h. After cooling to room temperature, the solvent was removed under reduced pressure, and the residue was washed with Et_2O. The product **C11** was obtained as a colorless oil (118.4 mg, 2.21 mmol, 70%).

$C_{12}H_{15}BrN_2$, **MW:** 267.17 g/mol. **^{1}H-NMR (300 MHz, $CDCl_3$):** δ = 10.89 (*s*, 1 H, H_{Imz}), 7.53-7.44 (*m*, 2 H, Ar-*H*), 7.44-7.35 (*m*, 3 H, Ar-H), 7.26 (*dt*, *J* = 23.0, 1.7 Hz, 2 H, H_{Imz}), 5.60 (*s*, 2 H, CH_2), 4.39 (*q*, *J* = 7.3 Hz, 2 H, N-CH_2-CH_3), 1.61 (*t*, *J* = 7.3 Hz, 3 H, N-CH_2-CH_3) ppm.

The analytical data are consistent with the literature.[15]

3.15 (1*R*,2*R*)-1,2-Di(naphthalen-1-yl)ethane-1,2-diamine S14

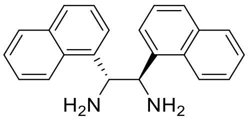

S14

Diamine **S14** was synthesized according to **GP 2-A** using (*S*,*S*)-1,2-bis-(2-hydroxyphenyl)-1,2-diaminoethane (301.9 mg, 1.17 mmol, 1.00 eq.) and 1-naphthaldehyde (0.40 mL, 2.94 mmol, 2.50 eq.). The diimine cleavage was performed in THF/Et_2O (2:1, 12 mL) over 2 d. After liberation of the free diamine, the crude product was purified by column chromatography on silica gel (DCM/MeOH/NEt_3, 80:1:1). Diamine **S14** was obtained as a beige solid (242.5 mg, 0.77 mmol, 66%).

$C_{22}H_{20}N_2$, MW: 312.42 g/mol. **^{1}H-NMR (400 MHz, $CDCl_3$):** δ = 8.24 (*d*, *J* = 8.5 Hz, 2 H, Ar-*H*), 7.81 (*d*, *J* = 7.8 Hz, 2 H, Ar-*H*), 7.76-7.66 (*m*, 4 H, Ar-*H*), 7.53-7.37 (*m*, 6 H, Ar-*H*), 5.05 (*s*, 2 H, Ar-C*H*-NH_2), 1.71 (*br*, 4 H, Ar-CH-NH_2) ppm.

The analytical data are consistent with the literature.[3]

3.16 (1*R*,2*R*)-1,2-Di(naphthalen-2-yl)ethane-1,2-diamine S15

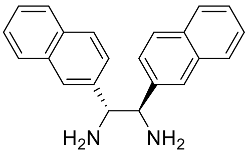

S15

Diamine **S15** was synthesized according to **GP 2-A** using (*S*,*S*)-1,2-bis-(2-hydroxyphenyl)-1,2-diaminoethane (202.1 mg, 0.79 mmol, 1.00 eq.) and 2-naphthaldehyde (306.9 mg, 1.97 mmol, 2.50 eq.). The diimine cleavage was carried out in THF (12 mL) over 3 h. After liberation of the free diamine, product **S15** was obtained without further purification as a beige solid (98.6 mg, 0.32 mmol, 40%).

$C_{22}H_{20}N_2$, **MW:** 312.42 g/mol. **^{1}H-NMR (400 MHz, $CDCl_3$):** δ = 7.88-7.75 (*m*, 8 H, Ar-*H*), 7.50-7.41 (*m*, 6 H, Ar-*H*), 4.43 (*s*, 2 H, Ar-C*H*-NH_2), 1.60 (*br*, 4 H, Ar-CH-NH_2) ppm.

The analytical data are consistent with the literature.[3]

3.17 (1R,2*R*)-1,2-Bis(4-fluorophenyl)ethane-1,2-diamine S16

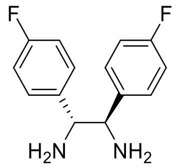

S16

Diamine **S16** was synthesized according to **GP 2-B** using (*S*,*S*)-1,2-bis-(2-hydroxyphenyl)-1,2-diaminoethane (326.1 mg, 1.26 mmol, 1.00 eq.) and *para*-fluorobenzaldehyde (0.41 mL, 3.17 mmol, 2.50 eq.). The diimine cleavage was carried out in THF/Et_2O (2:1, 12 mL) over 2 d. Product **S16** was obtained as a beige solid (297.0 mg, 1.19 mmol, 83%).

$C_{14}H_{14}F_2N_2$, **MW:** 248.28 g/mol. **^{1}H-NMR (300 MHz, $CDCl_3$):** δ = 7.21-7.11 (*m*, 4 H, Ar-*H*), 7.01-6.87 (*m*, 4 H, Ar-*H*), 4.00 (*s*, 2 H, Ar-C*H*-NH_2), 1.55 (*br*, 4 H, Ar-CH-NH_2) ppm.

The analytical data are consistent with the literature.[3]

3.18 (1*R*,2*R*)-1,2-Bis(4-nitrophenyl)ethane-1,2-diaminium chloride S17

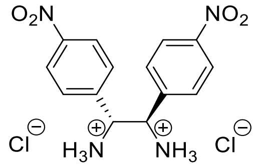

S17

Dihydrochloride **S17** was synthesized according to **GP 2-B** using (*S*,*S*)-1,2-bis-(2-hydroxyphenyl)-1,2-diaminoethane (319.7 mg, 1.24 mmol, 1.00 eq.) and *para*-nitrobenzaldehyde (469.7 mg, 3.10 mmol, 2.50 eq.). Product **S17** was obtained as a beige solid (316.6 mg, 0.84 mmol, 68%).

$C_{14}H_{16}Cl_2N_4O_4$, **MW:** 375.21 g/mol. **^{1}H-NMR (300 MHz, DMSO-d_6):** δ = 9.51 (*br*, 6 H, NH_3), 8.16 (*d*, *J* = 8.5 Hz, 4 H, Ar-*H*), 7.72 (*d, J* = 8.5 Hz, 4 H, Ar-*H*), 5.38 (*s*, 2 H, C*H*) ppm.

The analytical data are consistent with the literature.[2]

3.19 *N*-((1*R*,2*R*)-2-Amino-1,2-diphenylethyl)-1,1,1-trifluoromethanesulfonamide 7

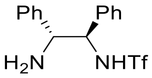

7

Sulfonamide **7** was synthesized according to **GP 3** using (*R*,*R*)-1,2-diphenylethylenediamine (0.25 g, 1.18 mmol, 1.00 eq.) and a solution of triflic anhydride (1 M in DCM, 1.2 mL, 1.18 mmol, 1.00 eq.). Product **7** was obtained as a white solid (0.34 g, 0.99 mmol, 84%).

$C_{15}H_{15}F_3N_2O_2S$, MW: 344.35 g/mol. **^{1}H-NMR (400 MHz, $CDCl_3$)**: δ = 7.49-7.27 (*m*, 10 H, Ar-*H*), 4.71 (*d*, *J* = 2.8 Hz, 1 H, Ph-C*H*-NHTf), 4.44 (*d*, *J* = 2.8 Hz, 1 H, Ph-C*H*-NH_2), 3.07 (*br*, 3 H, NH_2, N*H*Tf) ppm.

The analytical data are consistent with the literature.[4]

3.20 *N*-((1*R*,2*R*)-2-Amino-1,2-di(naphthalen-1-yl)ethyl)-1,1,1-trifluoromethanesulfonamide S18

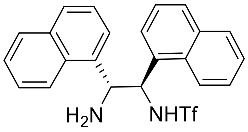

S18

Sulfonamide **S18** was synthesized according to **GP 3** using diamine **S14** (164.3 mg, 0.53 mmol, 1.00 eq.) and a solution of triflic anhydride (0.25 M in DCM, 2 mL, 0.53 mmol, 1.00 eq.). Product **S18** was obtained as a beige solid (112.5 mg, 0.25 mmol, 48%).

$C_{23}H_{19}F_3N_2O_2S$, MW: 444.47 g/mol. **MP:** 172 °C (decomposition). **$[\alpha]_D^{20}$ (*c* = 1.0 mg/mL, DCM)** = –48. **^{1}H-NMR (400 MHz, $CDCl_3$):** δ = 8.26-8.17 (*m*, 2 H, Ar-*H*), 7.97 (*d*, *J* = 8.2 Hz, 2 H, Ar-*H*), 7.90 (*d*, *J* = 8.2 Hz, 3 H, Ar-*H*), 7.77-7.53 (*m*, 7 H, Ar-*H*), 5.58 (*s*, 1 H, C*H*), 5.24 (*s*, 1 H, C*H*), 3.24 (*br*, 2 H, NH_2) ppm. **^{13}C-NMR (175 MHz, $CDCl_3$):** δ = 136.4, 136.0, 134.3, 134.2, 130.5, 129.9, 129.7, 129.5, 129.1, 129.0, 126.9, 126.6, 126.3, 126.0, 125.7, 125.5, 124.0, 123.8, 122.6, 122.3, 121.9, 120.1, 118.2, 116.4, 57.9, 52.6 ppm. **^{19}F-NMR (376 MHz, $CDCl_3$):** δ = –77.99 (*s*, 3 F, SO_2CF_3) ppm. **IR ($CDCl_3$):** $\tilde{\nu}$ = 3051, 2923, 1599, 1514, 1459, 1371, 1252, 1226, 1187, 1143, 1071, 1031, 1012, 927, 907, 859, 796, 773, 729, 641, 599, 574, 540, 518, 497, 475, 439 cm^{-1}. **HRMS (ESI) *m/z***: calculated for $C_{23}H_{20}F_3N_2O_2S$ $[M+H]^+$: 445.1192; measured 445.1189.

3.21 *N*-((1*R*,2*R*)-2-Amino-1,2-di(naphthalen-2-yl)ethyl)-1,1,1-trifluoromethanesulfonamide S19

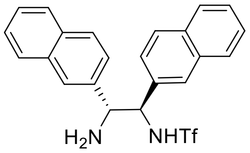

S19

Sulfonamide **S19** was synthesized according to **GP 3** using diamine **S15** (98.6 mg, 0.32 mmol, 1.00 eq.) and a solution of triflic anhydride (0.15 M in DCM, 2 mL, 0.32 mmol, 1.00 eq.). The crude product was purified by column chromatography on silica gel (DCM/MeOH, 30:1) and recrystallized from *n*-hexane/benzene (4:1). Product **S19** was obtained as a beige solid (79.4 mg, 0.18 mmol, 57%).

$C_{23}H_{19}F_3N_2O_2S$, MW: 444.47 g/mol. **MP:** 148 °C. **$[\alpha]_D^{20}$ (*c* = 1.0 mg/mL, DCM)** = 22. **^{1}H-NMR (400 MHz, MeOD-d_4):** δ = 7.79-7.65 (*m*, 5 H, Ar-*H*), 7.62-7.53 (*m*, 3 H, Ar-*H*), 7.45-7.37 (*m*, 2 H, Ar-*H*), 7.37-7.25 (*m*, 4 H, Ar-*H*), 4.91 (*d*, *J* = 9.2 Hz, 1 H, C*H*), 4.44 (*d*, *J* = 9.2 Hz, 1 H, C*H*) ppm. **^{13}C-NMR (175 MHz, MeOD-d_4):** δ = 139.6, 134.7, 134.5, 134.3, 134.2, 129.5, 129.0, 128.82, 128.76, 128.7, 128.6, 128.5, 127.9, 127.7, 127.5, 126.9, 126.7, 126.6, 126.2, 125.3, 123.5, 121.6, 119.8, 66.0, 63.9 ppm. **^{19}F-NMR (376 MHz MeOD-d_4):** δ = –78.96 (*s*, 3 F, SO_2CF_3) ppm. **IR ($CDCl_3$):** $\tilde{\nu}$ = 3058, 2906, 1602, 1509, 1371, 1258, 1194, 1073, 934, 909, 857, 817, 747, 667, 629, 609, 578, 516, 477, 410 cm^{-1}. **HRMS (ESI) *m/z*:** calculated for $C_{23}H_{20}F_3N_2O_2S$ $[M+H]^+$: 445.1192; measured 445.1182.

3.22 *N*-((1*R*,2*R*)-2-Amino-1,2-bis(4-fluorophenyl)ethyl)-1,1,1-trifluoromethanesulfonamide S20

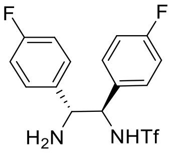

S20

Sulfonamide **S20** was synthesized according to **GP 3** using diamine **S16** (99.0 mg, 0.40 mmol, 1.00 eq.) and a solution of triflic anhydride (0.07 M in DCM, 5.7 mL, 0.40 mmol, 1.00 eq.). Product **S20** was obtained as a white solid (74.3 mg, 0.20 mmol, 49%).

$C_{15}H_{13}F_5N_2O_2S$, MW: 380.06 g/mol. **MP:** 82-87 °C. **$[\alpha]_D^{20}$ (*c* = 1.0 mg/mL, DCM)** = –3. **^{1}H-NMR (400 MHz, $CDCl_3$):** δ = 7.38-7.27 (*m*, 4 H, Ar-*H*), 7.14-7.04 (*m*, 4 H, Ar-*H*), 4.62 (*d*, *J* = 3.0 Hz, 1 H, C*H*), 4.32 (*d*, *J* = 3.0 Hz, 1 H, C*H*) ppm. **^{13}C-NMR (175 MHz, $CDCl_3$):** δ = 163.35, 163.31, 161.94, 161.90, 136.2, 134.91, 134.89, 128.11, 128.07, 127.83, 127.79, 121.9, 120.1, 118.3, 116.2, 116.1, 116.0, 115.9, 63.6, 59.7 ppm. **^{19}F-NMR (376 MHz $CDCl_3$):** δ = –77.78 (*s*, 3 F, SO_2CF_3), –113.45 (*sept.*, *J* = 4.5 Hz, 1 F, Ar-F), –113.63 (*sept.*, *J* = 4.5 Hz, 1 F, Ar-F) ppm. **IR ($CDCl_3$):** $\tilde{\nu}$ = 3081, 2930, 2854, 2018, 1608, 1514, 1469, 1424, 1373, 1229, 1196, 1158, 1098, 1073, 1015, 926, 862, 834, 794, 760, 724, 637, 614, 568, 549, 531, 482, 433 cm^{-1}. **HRMS (ESI) *m/z*:** calculated for $C_{15}H_{13}F_5N_2O_2S$ $[M+H]^+$: 381.0691; measured 381.0678.

3.23 *N*-((1*R*,2*R*)-2-Amino-1,2-bis(4-nitrophenyl)ethyl)-1,1,1-trifluoromethanesulfonamide S21

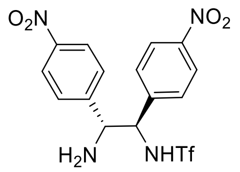

S21

Sulfonamide **S21** was synthesized according to **GP 3** using diamine **S17** (167.2 mg, 0.55 mmol, 1.00 eq.) and a solution of triflic anhydride (0.36 M in DCM, 1.5 mL, 0.55 mmol, 1.00 eq.). Product **S21** was obtained as a beige solid (26.0 mg, 0.05 mmol, 11%).

$C_{15}H_{13}F_3N_4O_6S$, MW: 434.35 g/mol. **MP:** 95 °C. **$[\alpha]_D^{20}$ (*c* = 1.0 mg/mL, DCM)** = 237. **^{1}H-NMR (400 MHz, $CDCl_3$):** δ = 8.38-8.24 (*m*, 4 H, Ar-*H*), 7.62-7.53 (*m*, 4 H, Ar-*H*), 4.78 (*d*, *J* = 3.4 Hz, 1 H, C*H*), 4.52 (*d*, *J* = 3.4 Hz, 1 H, C*H*) ppm. **^{13}C-NMR (175 MHz, $CDCl_3$):** δ = 148.1, 147.0, 145.7, 127.7, 127.3, 124.5, 124.3, 121.9, 120.1, 118.3, 116.5, 63.6, 59.8 ppm. **^{19}F-NMR (376 MHz $CDCl_3$):** δ = –77.34 (*s*, 3 F, SO_2CF_3) ppm. **IR ($CDCl_3$):** $\tilde{\nu}$ = 2925, 2855, 1660, 1607, 1523, 1461, 1377, 1348, 1262, 1227, 1196, 1146, 1109, 1078, 1015, 931, 860, 833, 749, 701, 608, 573, 510 cm^{-1}. **HRMS (ESI) *m/z*:** calculated for $C_{15}H_{13}F_3N_4O_6S$ $[M+H]^+$: 435.0581; measured 435.0572.

3.24 2-((1*R*,2*R*)-2-Aminocyclohexyl)isoindoline-1,3-dione S23

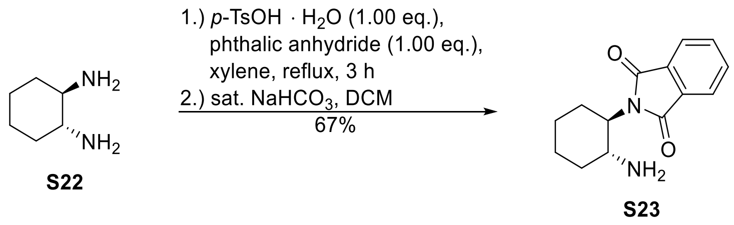

Following a literature procedure,[16] a solution of *p*-TsOH·H_2O (250 mg, 1.31 mmol, 1.00 eq.) in xylene (7 mL) was dehydrated by azeotropic distillation. *R*,*R*-DACH **S22** (150 mg, 1.31 mmol, 1.00 eq.) and phthalic anhydride (195 mg, 1.31 mmol, 1.00 eq.) were then added at room temperature. The reaction mixture was stirred under reflux for 3 h. After cooling to room temperature, the supernatant solution was removed, and the remaining solid was washed *n*-hexane/xylene (1:1; 3 × 10 mL). The resulting hydrotosylate was dried under high vacuum. To liberate the free amine, the solid was dissolved in DCM (30 mL) and stirred with saturated $NaHCO_3$ solution (7 mL) at room temperature overnight. The layers were separated, and the aqueous phase was extracted with DCM (3 × 5 mL). The combined organic phases were dried over Na_2SO_4 and concentrated under reduced pressure. Product **S23** was obtained as a beige solid (214 mg, 0.87 mmol, 67%).

$C_{14}H_{16}N_2O_2$, MW: 244.29 g/mol. **^{1}H-NMR (300 MHz, $CDCl_3$):** δ = 7.87-7.78 (*m*, 2 H, Ar-*H*), 7.75-7.66 (*m*, 2 H, Ar-*H*), 3.80 (*dt*, *J* = 11.6, 4.0 Hz, 1 H, C*H*N), 3.40 (*dt*, J = 11.0, 4.0 Hz, 1 H, C*H*N), 2.31-2.10 (*m*, 1 H, C*H*), 2.09-1.98 (*m*, 1 H, C*H*), 1.90-1.69 (*m*, 3 H, N*H_2*, C*H*), 1.52-1.07 (*m*, 5 H, C*H_2*, C*H*) ppm.

The analytical data are consistent with the literature.[16]

3.25 *N*-((1*R*,2*R*)-2-(1,3-Dioxoisoindolin-2-yl)cyclohexyl)-1,1,1-trifluoromethanesulfonamide S24

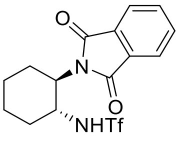

S24

Sulfonamide **S24** was synthesized according to **GP 3** using amine **S23** (51.7 mg, 0.21 mmol, 1.00 eq.) and a solution of triflic anhydride (0.21 M in DCM, 1.0 mL, 0.21 mmol, 1.00 eq.). The crude product was purified by column chromatography on silica gel (PE/EE, 4:1) to give **S24** as a white solid (52.4 mg, 0.13 mmol, 66%).

$C_{15}H_{15}F_3N_2O_4S$, **MW:** 376.35 g/mol. **MP:** 228-232 °C. $[\alpha]_D^{20}$ (*c* = 1.0 mg/mL, DCM) = –4. **^{1}H-NMR (400 MHz, CDCl$_3$):** δ = 7.88-7.81 (*m*, 2 H, Ar-*H*), 7.77-7.69 (*m*, 2 H, Ar-*H*), 5.14 (*s*, 1 H, N*H*Tf), 4.17 (*m*, 2 H, C*H*), 2.56 (*dq*, *J* = 13.0, 3.8, 1 H, C*H*), 2.36-2.23 (*m*, 1 H, C*H*), 1.95-1.77 (*m*, 3 H, C*H*, C*H*$_2$), 1.60-1.20 (*m*, 3 H, C*H*, C*H*$_2$) ppm. **^{13}C-NMR (175 MHz, CDCl$_3$):** δ = 168.7, 134.4, 131.7, 123.5, 122.2, 120.4, 118.5, 116.7, 56.3, 54.6, 34.9, 28.7, 25.2, 24.7 ppm. **^{19}F-NMR (376 MHz CDCl$_3$):** δ = –78.05 (*s*, 3 F, SO_2CF_3) ppm. **IR (CDCl$_3$):** $\tilde{\nu}$ = 3216, 2943, 2864, 1769, 1702, 1614, 1469, 1457, 1378, 1335, 1261, 1231, 1190, 1147, 1087, 1067, 1021, 948, 907, 721, 642, 610, 574, 531, 481 cm^{-1}. **HRMS (ESI) *m/z*:** calculated for $C_{15}H_{15}F_3N_2O_4SNa$ $[M+Na]^+$: 399.0597; measured 399.0585.

3.26 *N*-((1*R*,2*R*)-2-Aminocyclohexyl)-1,1,1-trifluoromethanesulfonamide S25

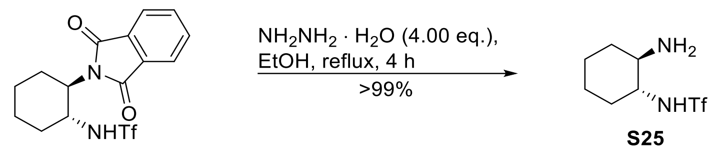

S24 **S25**

Following a literature procedure,[16] sulfonamide **S24** (42.3 mg, 0.11 mmol, 1.00 eq.) was dissolved in ethanol (1.5 mL), and hydrazine hydrate (28 µL, 0.44 mmol, 4.00 eq.) was added. The reaction mixture was stirred under reflux for 4 h. Et_2O (2 mL) was then added, and the resulting suspension was filtered through cotton. The solvent was removed under reduced pressure, and the product was dried under high vacuum. Product **S25** was obtained as a white solid without further purification (28.2 mg, 0.11 mmol, >99%).

$C_7H_{13}F_3N_2O_2S$, **MW:** 246.25 g/mol. **^{1}H-NMR (400 MHz, MeOD-d$_4$):** δ = 3.09-2.98 (*m*, 1 H, C*H*), 2.68-2.58 (*m*, 1 H, C*H*), 2.06-1.91 (*m*, 2 H, C*H*$_2$), 1.81-1.66 (*m*, 2 H, C*H*$_2$), 1.44-1.22 (*m*, 4 H, C*H*$_2$) ppm. **^{19}F-NMR (376 MHz, MeOD-d$_4$):** δ = –79.12 (*s*, 3 F, SO_2CF_3) ppm.

The analytical data are consistent with the literature.[17]

3.27 *N*-(2'-Amino-[1,1'-binaphthalen]-2-yl)-1,1,1-trifluoromethanesulfonamide S27

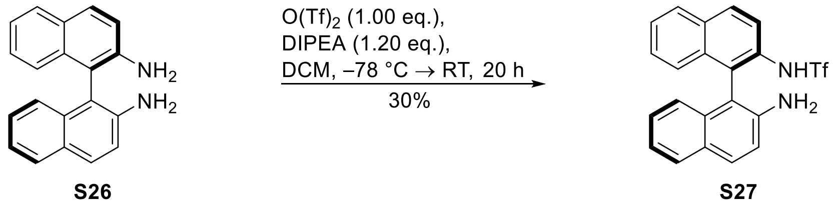

S26 **S27**

Following a literature procedure,[18] (*R*)-BINAM **S26** (102.4 mg, 0.36 mmol, 1.00 eq.) was dissolved under a nitrogen atmosphere in dry DCM (20 mL) and cooled to −78 °C. DIPEA (75 µL, 0.43 mmol, 1.20 eq.) was added, and a solution of triflic anhydride (0.36 M in DCM, 1.5 mL, 0.43 mmol, 1.00 eq.) was added over 1 h using a syringe pump. The reaction mixture was stirred for an additional hour at −78 °C, then allowed to warm to room temperature overnight. Water (5 mL) was added, and the layers were separated. The aqueous phase was extracted with DCM (3 × 5 mL), and the combined organic phases were washed with saturated NaCl solution, dried over Na_2SO_4, and concentrated under reduced pressure. The crude product was purified by preparative thin-layer chromatography on silica gel (DCM/MeOH, 50:1). Product **S27** was obtained as a white solid (45.0 mg, 0.11 mmol, 30%).

$C_{21}H_{15}F_3N_2O_2S$, MW: 416.42 g/mol. **^{1}H-NMR (400 MHz, $CDCl_3$):** δ = 8.03 (*d*, *J* = 9.0 Hz, 1 H, Ar-*H*), 8.00-7.93 (*m*, 2 H, Ar-*H*), 7.88 (*d*, *J* = 8.8 Hz, 1 H, Ar-*H*), 7.82 (*d*, *J* = 8.4 Hz, 1 H, Ar-*H*), 7.53-7.46 (*m*, 1 H, Ar-*H*), 7.37-7.19 (*m*, 4 H, Ar-*H*), 7.15 (*d*, *J* = 8.8 Hz, 1 H, Ar-*H*), 6.83 (*d*, *J* = 8.3 Hz, 1 H, Ar-*H*), 3.64 (*br*, 2 H, N*H*$_2$) ppm. **^{19}F-NMR (376 MHz, $CDCl_3$):** δ = –76.29 (*s*, 3 F, SO_2CF_3) ppm.

The analytical data are consistent with the literature.[18]

4 Ligand Synthesis

4.1 1-(5-(*tert*-Butyl)-3-((*E*)-(((1*R*,2*R*)-1,2-diphenyl-2-((trifluoromethyl)sulfonamido)ethyl)imino)methyl)-2-hydroxybenzyl)-3-ethyl-4-mesityl-1*H*-1,2,3-triazol-3-ium hexafluorophosphate(V) S28

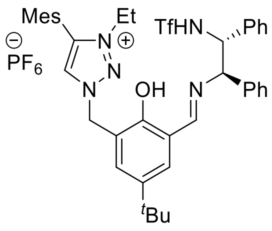

S28

Preligand **S28** was synthesized according to **GP 4** using aldehyde **S6** (35.0 mg, 63.5 µmol, 1.00 eq.) and sulfonamide **7** (22.3 mg, 64.7 µmol, 1.02 eq.). After workup, product **S28** was obtained as a yellow solid (51.2 mg, 58.3 µmol, 92%).

$C_{40}H_{45}F_9N_5O_3PS$, MW: 877.85 g/mol. **MP:** 133 °C (decomposition). **$[\alpha]_D^{20}$ (*c* = 1.0 mg/mL, DCM)** = +140.3. **^{1}H-NMR (400 MHz, $CDCl_3$):** δ = 13.61 (*s*, 1 H, Ar-O*H*), 8.59 (*s*, 1 H, Ar-C*H*N),

8.19 (*s*, 1 H, H_{Trz}), 7.60 (*d*, *J* = 2.6 Hz, 1 H, Ar-*H*), 7.46 (*d*, *J* = 2.6 Hz, 1 H, Ar-*H*), 7.23-7.14 (*m*, 6 H, H_{Ph}), 7.14-7.04 (*m*, 4 H, H_{Ph}) 6.98 (*s*, 2 H, H_{Mes}), 5.76 (*q*, *J* = 14.9 Hz, 2 H, Ar-CH_2-N_{Trz}), 4.86 (*dd*, *J* = 86.2, 9.0 Hz, 2 H, C*H*PhC*H*Ph), 4.20 (*qd*, *J* = 7.2, 3.3 Hz, 2 H, N-CH_2-CH_3), 2.34 (*s*, 3 H, *p*-Mes-CH_3), 2.02 (*s*, 6 H, *o*-Mes-(CH_3)$_2$), 1.50 (*t*, *J* = 7.4 Hz, 3 H, N-CH_2-CH_3), 1.38 (*s*, 9 H, Ar-C(CH_3)$_3$) ppm. **^{13}C-NMR (175 MHz, $CDCl_3$):** δ = 168.0, 158.0, 142.5, 142.5, 141.1, 138.6, 138.4, 137.1, 132.5, 131.6, 129.6, 129.4, 129.3, 128.7, 128.7, 128.3, 128.2, 127.9, 127.4, 118.6, 118.2, 117.8, 77.3, 77.2, 77.0, 65.3, 54.3, 46.7, 34.3, 31.4, 21.4, 20.1, 20.0, 14.2 ppm. **^{19}F-NMR (376 MHz, $CDCl_3$):** δ = –72.29 (*d*, *J* = 717.3 Hz, 6 F, $(PF_6)^-$), –77.88 (*s*, 3 F, SO_2CF_3) ppm. **^{31}P-NMR (162 MHz, $CDCl_3$):** δ = –144.53 (*sept*, *J* = 613.1 Hz, 1 P, $(PF_6)^-$) ppm. **IR ($CDCl_3$):** $\tilde{\nu}$ = 2962, 1632, 1481, 1456, 1377, 1229, 1195, 1147, 1047, 844, 737, 699, 597, 558 cm^{-1}. **HRMS (ESI) *m/z*:** calculated for $C_{40}H_{45}F_3N_5O_3S$ [M-PF_6]$^+$: 732.3190; measured 732.3177.

4.2 3-(5-(*tert*-Butyl)-3-((*E*)-(((1*R*,2*R*)-1,2-diphenyl-2-((trifluoromethyl)sulfonamido)ethyl)imino)methyl)-2-hydroxybenzyl)-1-methyl-1*H*-imidazol-3-ium chloride S29

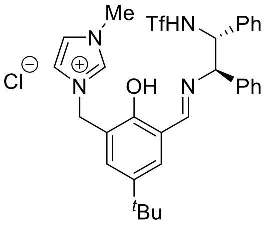

S29

Preligand **S29** was synthesized according to **GP 4** using aldehyde **S7** (36.7 mg, 118.8 µmol, 1.00 eq.) and sulfonamide **7** (40.9 mg, 118.8 µmol, 1.05 eq.). After workup, product **S29** was obtained as a yellow solid (62.3 mg, 98.1 µmol, 83%).

$C_{31}H_{34}ClF_3N_4O_3S$, MW: 635.14 g/mol. **^{1}H-NMR (400 MHz, MeOD-d_4):** δ = 8.98 (*s*, 1 H, NC*H*N), 8.59 (s, 1 H, Ar-C*H*N), 7.70-7.51 (*m*, 2 H, Ar-*H*), 7.56-7.49 (*m*, 2 H, Ar-*H*), 7.23-7.14 (*m*, 8 H, Ar-*H*), 7.12-7.06 (*m*, 2 H, Ar-*H*), 5.45 (*dd*, *J* = 63.5, 14.0 Hz, 2 H, Ar-CH_2-N), 4.94 (*d*, *J* = 9.8 Hz, 1 H, C*H*PhCHPh), 4.61 (*d*, *J* = 9.8 Hz, 1 H, CHPhC*H*Ph), 3.90 (*s*, 3 H. N-CH_3), 1.34 (*s*, 9 H, Ar-C(CH_3)$_3$) ppm. **^{19}F-NMR (376 MHz, MeOD-d_4):** δ = –80.01 (*s*, 3 F, SO_2CF_3) ppm.

The analytical data are consistent with the literature.[19]

4.3 3-(5-(*tert*-Butyl)-3-((*E*)-(((1*R*,2*R*)-1,2-diphenyl-2-((trifluoromethyl)sulfonamido)ethyl)imino)methyl)-2-hydroxybenzyl)-1-(2,6-diisopropylphenyl)-1*H*-imidazol-3-ium chloride S30

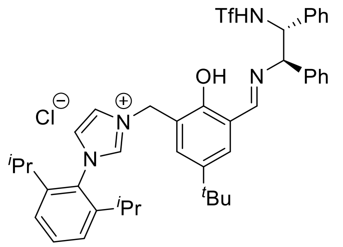

S30

Preligand **S30** was synthesized according to **GP 4** using aldehyde **S8** (28.1 mg, 61.8 µmol, 1.00 eq.) and sulfonamide **7** (21.27 mg, 61.8 µmol, 1.00 eq.). After workup, product **S30** was obtained as a yellow solid (38.1 mg, 53.4 µmol, 86%).

$C_{42}H_{48}ClF_3N_4O_3S$, MW: 781.37 g/mol. **MP:** 196 °C (decomposition). **$[\alpha]_D^{20}$(*c* = 1.0 mg/mL, DCM)** = +26. **^{1}H-NMR (400 MHz, $CDCl_3$):** *δ* = 14.37 (*s*, 1 H, Ar-O*H*), 11.64 (*br*, 1 H, N*H*Tf), 10.49 (*s*, 1 H, NC*H*N), 8.35 (*s*, 1 H, Ar-C*H*N), 8.23 (*s*, 1 H, Ar-*H*), 8.08 (*s*, 1 H, Ar-*H*), 7.50 (*t*, *J* = 7.8 Hz, 1 H, Ar-*H*), 7.44 (*d*, *J* = 6.5 Hz, 2 H, Ar-*H*), 7.31-7.26 (*m*, 2 H, Ar-*H*), 7.13-7.04 (*m*, 6 H, Ar-*H*), 7.01-6.96 (*m*, 3 H, Ar-*H*), 6.70 (*s*, 1 H, Ar-*H*), 6.32 (*d*, *J* = 13.3 Hz, 1 H, Ar-C*H*HN), 5.88 (*d*, *J* = 13.2 Hz, 1 H, Ar-CH*H*N), 5.60 (*br*, 1 H, C*H*PhCHPh), 4.98 (*d*, *J* = 10.7 Hz, 1 H, CHPhC*H*Ph), 2.38 (*sept.*, *J* = 6.7 Hz, 1 H, C*H*$(CH_3)_2$), 2.13 (*sept.*, *J* = 6.7 Hz, 1 H, C*H*$(CH_3)_2$), 1.21-1.16 (*m*, 9 H, CH(CH_3)$_2$), 1.14 (*s*, 9 H, Ar-C(CH_3)$_3$), 1.07 (*d*, *J* = 6.8 Hz, 3 H, CH(CH_3)$_2$) ppm. **^{13}C-NMR (175 MHz, $CDCl_3$):** *δ* = 168.2, 156.9, 145.9, 145.7, 141.6, 140.3, 138.9, 133.5, 131.7, 130.8, 129.3, 128.6, 128.5, 128.4, 128.1, 127.7, 127.4, 124.7, 124.6, 123.9, 123.2, 122.7, 117.9, 66.1, 48.4, 34.3, 31.5, 29.8, 28.8, 28.7, 24.7, 24.4, 24.0 ppm. **^{19}F-NMR (376 MHz, $CDCl_3$):** *δ* = –77.57 (*s*, 3 F, SO_2CF_3) ppm. **IR ($CDCl_3$):** $\tilde{\nu}$ = 3061, 3032, 2961, 2925, 2854, 2206, 1629, 1602, 1561, 1543, 1459, 1370, 1286, 1226, 1188, 1148, 1050, 956, 909, 843, 825, 805, 791, 774, 758, 730, 698, 671, 640, 627, 599, 571, 508, 458 cm^{-1}. **HRMS (ESI) *m/z*:** calculated for $C_{42}H_{48}F_3N_4O_3S$ [M-Cl]$^+$: 745.3394; measured 745.3366.

4.4 3-(5-(*tert*-Butyl)-3-((*E*)-(((1*R*,2*R*)-1,2-diphenyl-2-((trifluoromethyl)sulfonamido)ethyl)imino)methyl)-2-hydroxybenzyl)-1-mesityl-1*H*-imidazol-3-ium chloride 8

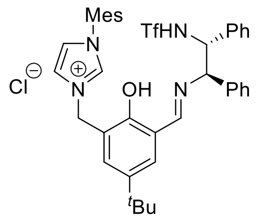

8

Preligand **8** was synthesized according to **GP 4** using aldehyde **S9** (20.7 mg, 50.1 µmol, 1.00 eq.) and sulfonamide **7** (18.2 mg, 52.6 µmol, 1.05 eq.). After workup, product **8** was obtained as a yellow solid (35.3 mg, 47.7 µmol, 95%).

$C_{39}H_{42}ClF_3N_4O_3S$, MW: 739.30 g/mol. **MP:** 185-197 °C. **$[\alpha]_D^{20}$ (*c* = 1.0 mg/mL, DCM)** = +70. **^{1}H-NMR (400 MHz, DMSO-d$_6$):** δ = 13.42 (*s*, 1 H, Ar-O*H*), 10.40 (*s*, 1 H, Ar-C*H*N), 9.57 (*s*, 1 H, NC*H*N), 8.72 (*s*, 1 H, *H*NTf), 7.95 (*d*, *J* = 27.5 Hz, 2 H, Ar-*H*), 7.63-7.50 (*m*, 2 H, Ar-*H*), 7.24-7.12 (*m*, 12 H, Ar-*H*), 5.54 (*dd*, *J* = 24.2, 15.2 Hz, 2 H, Ar-CH_2-N), 4.93 (*s*, 1 H, C*H*PhCHPh), 4.69 (*d*, *J* = 9.0 Hz, 1 H, CHPhC*H*Ph), 2.33 (*s*, 3 H, *p*-Mes-CH_3), 1.98 (*d*, *J* = 20.7 Hz, 6 H, *o*-Mes-(CH_3)$_2$), 1.28 (*s*, 9 H, Ar-C(CH_3)$_3$) ppm.**^{13}C-NMR (175 MHz, DMSO-d$_6$):** δ = 167.8, 156.6, 141.2, 140.3, 139.1, 138.1, 134.31, 134.30, 131.2, 130.8, 129.8, 129.3, 128.5, 128.2, 127.7, 127.5, 127.4, 124.0, 123.2, 121.0, 117.9, 77.4, 64.2, 48.6, 40.0, 33.9, 31.1, 20.6, 16.9, 16.8 ppm. **^{19}F-NMR (376 MHz, DMSO-d$_6$):** δ = –78.02 (*s*, 3 F, SO_2CF_3) ppm. **IR (CDCl$_3$):** $\tilde{\nu}$ **=** 2959, 1629, 1602, 1546, 1480, 1455, 1370, 1286, 1226, 1190, 1148, 1049, 955, 893, 854, 758, 698, 670, 639, 626, 598, 570, 511 cm^{-1}. **HRMS (ESI) *m/z*:** calculated for $C_{39}H_{42}F_3N_4O_3S$ [M-Cl]$^+$: 703.2924; measured 703.2927.

4.5 3-(5-(*tert*-Butyl)-3-((*E*)-(((1*R*,2*R*)-1,2-diphenyl-2-((trifluoromethyl)sulfonamido)ethyl)imino)methyl)-2-hydroxybenzyl)-1-mesityl-1*H*-imidazol-3-ium bromide S31

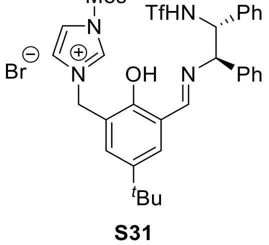

S31

Preligand **S31** was synthesized according to **GP 4** using aldehyde **S10** (23.8 mg, 51.9 µmol, 1.00 eq.) and sulfonamide **7** (17.9 mg, 51.9 µmol, 1.00 eq.). After workup, product **S31** was obtained as a yellow solid (32.9 mg, 42.0 µmol, 81%).

$C_{39}H_{42}BrF_3N_4O_3S$, MW: 783.75 g/mol. **MP:** 192-194 °C. **$[\alpha]_D^{20}$(*c* = 1.0 mg/mL, DCM)** = +50. **^{1}H-NMR (400 MHz, $CDCl_3$):** *δ* = 14.25 (*s*, 1 H, Ar-O*H*), 10.18 (*s*, 1 H, Ar-C*H*N), 8.26 (*s*, 1 H, NC*H*N), 8.23 (*d*, *J* = 1.7 Hz, 1 H, Ar-*H*), 8.13 (*s*, 1 H, Ar-*H*), 7.42 (*d*, *J* = 6.6 Hz, 2 H, Ar-*H*), 7.18-7.06 (*m*, 6 H, Ar-*H*), 7.05-6.93 (*m*, 5 H, Ar-*H*), 6.84 (*d*, *J* = 2.0 Hz, 1 H, Ar-*H*), 6.14 (*d*, *J* = 14.0 Hz, 1 H, Ar-CH_2-N), 5.73 (*d*, *J* = 14.0 Hz, 1 H, Ar-CH_2-N), 5.56 (*d*, *J* = 10.5 Hz, 1 H, C*H*PhCHPh), 5.05 (*d*, *J* = 10.5 Hz, 1 H, CHPhC*H*Ph), 2.32 (*s*, 3 H, *p*-Mes-CH_3), 2.08 (*s*, 3 H, *o*-Mes-CH_3), 1.98 (*s*, 3 H, *o*-Mes-CH_3), 1.13 (*s*, 9 H, Ar-C(CH_3)$_3$) ppm. **^{13}C-NMR (175 MHz, $CDCl_3$):** *δ* = 168.6, 157.2, 142.3, 141.3, 139.8, 137.7, 137.4, 134.6, 134.4, 132.9, 130.9, 129.9, 129.6, 128.6, 128.4, 128.3, 127.9, 127.9, 124.2, 122.2, 121.5, 118.3, 66.3, 48.7, 34.5, 31.5, 29.9, 22.8, 21.2, 17.8, 17.5, 14.3 ppm. **^{19}F-NMR (376 MHz, $CDCl_3$):** *δ* = –77.78 (*s*, 3 F, SO_2CF_3) ppm. **IR ($CDCl_3$):** $\tilde{\nu}$ = 3140, 3031, 2959, 2927, 2857, 1733, 1629, 1603, 1561, 1546, 1480, 1455, 1371, 1286, 1266, 1226, 1191, 1147, 1065, 1048, 984, 953, 909, 853, 826, 774, 758, 728, 698, 669, 640, 627, 598, 570, 515, 485, 441 cm^{-1}. **HRMS (ESI) *m/z*:** calculated for $C_{39}H_{42}F_3N_4O_3S$ [M-Br]$^+$: 703.2924; measured 703.2902.

4.6 3-(5-(*tert*-Butyl)-3-((*E*)-(((1*R*,2*R*)-1,2-diphenyl-2-((trifluoromethyl)sulfonamido)ethyl)imino)methyl)-2-hydroxybenzyl)-1-mesityl-1*H*-imidazol-3-ium iodide S32

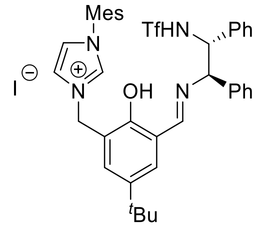

S32

Preligand **S32** was synthesized according to **GP 4** using aldehyde **S11** (22.1 mg, 43.9 µmol, 1.00 eq.) and sulfonamide **7** (15.1 mg, 43.9 µmol, 1.00 eq.). After workup, product **S32** was obtained as a yellow solid (32.8 mg, 39.5 µmol, 90%).

$C_{39}H_{42}F_3IN_4O_3S$, MW: 830.75 g/mol. **MP:** 147-155 °C. **$[\alpha]_D^{20}$ (*c* = 1.0 mg/mL, DCM)** = +55. **^{1}H-NMR (400 MHz, $CDCl_3$):** *δ* = 14.06 (*s*, 1 H, Ar-O*H*), 9.80 (*s*, 1 H, Ar-C*H*N), 8.45 (*s*, 1 H, NC*H*N), 8.10 (*d*, *J* = 2.2 Hz, 1 H, Ar-*H*), 8.03 (*t*, *J* = 1.0 Hz, 1 H, Ar-*H*), 7.37-7.33 (*m*, 2 H, Ar-

H), 7.19-7.10 (*m*, 6 H, Ar-*H*), 7.08-7.01 (*m*, 3 H, Ar-*H*), 7.01-6.95 (*m*, 3 H, Ar-*H*), 5.97 (*d*, *J* = 14.0 Hz, 1 H, Ar-CH_2-N), 5.66 (*d*, *J* = 14.0 Hz, 1 H, Ar-CH_2-N), 5.46 (*d*, *J* = 10.2 Hz, 1 H, C*H*PhCHPh), 5.09 (*d*, *J* = 10.2 Hz, 1 H, CHPhC*H*Ph), 2.33 (*s*, 3 H, *p*-Mes-CH_3), 2.06 (*s*, 3 H, *o*-Mes-(CH_3)$_2$), 2.00 (*s*, 3 H, *o*-Mes-(CH_3)$_2$), 1.16 (*s*, 9 H, Ar-C(CH_3)$_3$) ppm. **^{13}C-NMR (175 MHz, $CDCl_3$):** δ = 168.6, 157.2, 142.3, 141.3, 139.8, 137.7, 137.4, 134.6, 134.4, 132.9, 130.9, 129.9, 129.6, 128.6, 128.4, 128.3, 127.9, 127.9, 124.2, 122.2, 121.5, 118.3, 66.3, 48.7, 34.5, 31.5, 29.9, 22.8, 21.2, 17.8, 17.5, 14.3 ppm. **^{19}F-NMR (376 MHz, $CDCl_3$):** δ = –77.81 (*s*, 3 F, SO_2CF_3) ppm. **IR ($CDCl_3$):** $\tilde{\nu}$ = 3032, 2959, 2924, 2854, 1740, 1630, 1603, 1546, 1480, 1456, 1374, 1286, 1227, 1194, 1147, 1048, 951, 916, 854, 825, 774, 758, 731, 699, 669, 638, 627, 597, 570, 507, 484, 473 cm^{-1}. **HRMS (ESI) *m/z*:** calculated for $C_{39}H_{42}F_3N_4O_3S$ [M-Cl]$^+$: 703.2924; measured 703.2904.

4.7 *N*-((1*R*,2*R*)-2-(((*E*)-5-(*tert*-Butyl)-2-hydroxy-3-methylbenzylidene)amino)-1,2-diphenylethyl)-1,1,1-trifluoromethanesulfonamide S33

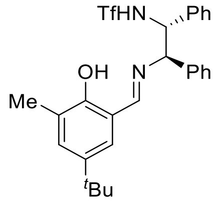

S33

Preligand **S33** was synthesized according to **GP 4** using aldehyde 5-(*tert*-butyl)-2-hydroxy-3-methylbenzaldehyde (32.3 mg, 168.0 µmol, 1.00 eq.) and sulfonamide **7** (60.7 mg, 176.4 µmol, 1.05 eq.). The crude product was washed with 1 M HCl solution (10 mL), and the aqueous phase was extracted with DCM (3 × 10 mL). The combined organic layers were dried over Na_2SO_4 and concentrated under reduced pressure. Product **S33** was obtained as a yellow solid (82.2 mg, 158.5 µmol, 94%).

$C_{27}H_{29}F_3N_2O_3S$, MW: 518.60 g/mol. **^{1}H-NMR (400 MHz, $CDCl_3$):** δ = 8.14 (*s*, 1 H, Ar-C*H*N), 7.41-7.26 (*m*, 9 H, Ar-*H*), 7.18-7.09 (*m*, 2 H, Ar-*H*), 6.94 (*d*, *J* = 2.5 Hz, 1 H, Ar-*H*), 5.79 (*br*, 1 H, Ar-O*H*), 5.06 (*d*, *J* = 4.4 Hz, 1 H, C*H*PhCHPh), 4.70 (*d*, *J* = 4.4 Hz, 1 H, CHPhC*H*Ph), 2.32 (*s*, 3 H, Ar-CH_3), 1.25 (*s*, 9 H, Ar-C(CH_3)$_3$) ppm.

The analytical data are consistent with the literature.[20]

4.8 *N*-(5-(*tert*-Butyl)-3-((*E*)-(((1*R*,2*R*)-1,2-diphenyl-2-((trifluoromethyl)sulfonamido)ethyl)imino)methyl)-2-hydroxybenzyl)-*N*-ethyl-*N*-methylethanaminium chloride S34

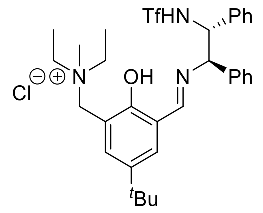

S34

Preligand **S34** was synthesized according to **GP 4** using *N*-(5-(*tert*-butyl)-3-formyl-2-hydroxybenzyl)-*N*-ethyl-*N*-methylethanaminium chloride (30.8 mg, 82.6 µmol, 1.00 eq.) and sulfonamide **7** (29.9 mg, 86.7 µmol, 1.05 eq.). For workup, methanol was added until the precipitate dissolved, and the resulting solution was filtered through Celite®. After removal of the solvent under reduced pressure, the solid was washed with a mixture of *n*-pentane/Et_2O (1:1, 5 mL/mmol) and subsequently dried under high vacuum to afford preligand **S34** as a yellow solid (51.3 mg, 80.1 µmol, 97%).

$C_{32}H_{41}ClF_3N_3O_3S$, MW: 640.20 g/mol. **^{1}H-NMR (400 MHz, DMSO-d$_6$):** δ = 13.98 (*s*, 1 H, Ar-O*H*), 10.48 (*s*, 1 H, Ar-C*H*N), 7.70-7.62 (*m*, 2 H, Ar-*H*), 7.29-7.10 (*m*, 10 H, Ar-*H*), 4.90 (*br*, 1 H, Ar-CH_2-N), 4.76 (*br*, 1 H, Ar-CH_2-N), 4.58 (*d*, *J* = 13.0 Hz, 1 H, C*H*PhCHPh), 4.45 (*d*, *J* = 13.0 Hz, 1 H, CHPhC*H*Ph), 3.44-3.20 (*m*, 4 H, N-CH_2-CH_3), 2.87 (*s*, 3 H, *N*-CH_3), 1.37-1.30 (*m*, 6 H, N-CH_2-CH_3), 1.30 (*s*, 9 H, Ar-C(CH_3)$_3$) ppm.

The analytical data are consistent with the literature.[21]

4.9 1-(5-(*tert*-Butyl)-3-((*E*)-(((1*R*,2*R*)-1,2-diphenyl-2-((trifluoromethyl)sulfonamido)ethyl)imino)methyl)-2-hydroxybenzyl)pyridin-1-ium chloride S35

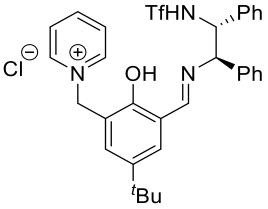

S35

The synthesis of preligand **S35** was carried out according to **GP 4** using aldehyde **S12** (26.0 mg, 85.1 µmol, 1.00 eq.) and sulfonamide **7** (29.3 mg, 85.1 µmol, 1.00 eq.). After workup, preligand **S35** was obtained as a yellow solid (51.7 mg, 81.8 µmol, 96%).

$C_{32}H_{33}ClF_3N_3O_3S$, MW: 632.14 g/mol. **MP:** 184 °C (decomposition). **$[\alpha]_D^{20}$ (*c* = 1.0 mg/mL, DCM)** = +88. **^{1}H-NMR (400 MHz, $CDCl_3$):** *δ* = 14.81 (*s*, 1 H, Ar-O*H*), 9.74 (*d*, *J* = 6.4 Hz, 2 H, Ar-*H*), 8.39 (*s*, 1 H, Ar-C*H*N), 8.25 (*d*, *J* = 1.7 Hz, 1 H, Ar-*H*), 8.20 (*t*, *J* = 7.4 Hz, 1 H, Ar-*H*), 7.79 (*t*, J = 6.9 Hz, 2 H, Ar-*H*), 7.44 (*d*, *J* = 6.8 Hz, 2 H, Ar-*H*), 7.17-7.09 (*m*, 6 H, Ar-*H*), 7.07-6.99 (*m*, 3 H, Ar-*H*), 6.39 (*d*, *J* = 13.3 Hz, 1 H, N-C*H*H-Ar), 5.89 (*d*, *J* = 13.3 Hz, 1 H, N-CH*H*-Ar), 5.49 (*d*, *J* = 10.5 Hz, 1 H, C*H*PhCHPh), 4.92 (*d*, *J* = 10.5 Hz, 1 H, CHPhC*H*Ph), 1.10 (*s*, 9 H, Ar-C(C*H*$_3$)$_3$) ppm. **^{13}C-NMR (175 MHz, $CDCl_3$):** *δ* = 167.4, 158.2, 146.0, 144.0, 142.0, 139.1, 138.3, 133.1, 130.0, 128.8, 128.6, 128.4, 128.2, 127.9, 127.7, 127.5, 122.2, 121.2, 120.4, 118.6, 118.3, 116.8, 73.7, 65.9, 60.0, 53.6, 34.3, 31.4 ppm. **^{19}F-NMR (376 MHz, $CDCl_3$):** *δ* = –78.01 (*s*, 3 F, SO_2CF_3) ppm. **IR ($CDCl_3$):** $\tilde{\nu}$ = 3032, 2960, 2866, 2779, 2685, 2192, 1630, 1601, 1480, 1369, 1286, 1262, 1225, 1189, 1147, 1049, 1030, 956, 908, 843, 819, 775, 761, 728, 698, 684, 636, 622, 599, 570, 513, 487 cm^{-1}. **HRMS (ESI) *m/z*:** calculated for $C_{32}H_{33}F_3N_3O_3S$ [M-Cl]$^+$: 596.2171; measured 596.2189.

4.10 3-(5-(*tert*-Butyl)-3-((*E*)-(((1*R*,2*R*)-1,2-diphenyl-2-((trifluoromethyl)sulfonamido)ethyl)imino)methyl)-2-hydroxybenzyl)-1-mesityl-2-methyl-1*H*-imidazol-3-ium chloride S43

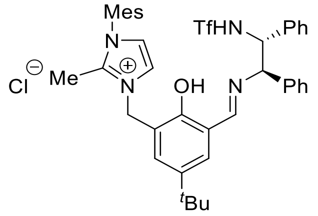

S43

Preligand **S43** was synthesized according to **GP 4** using aldehyde **S43** (27.6 mg, 64.5 µmol, 1.00 eq.) and sulfonamide **7** (22.3 mg, 64.5 µmol, 1.00 eq.). After workup, product **S43** was obtained as a yellow solid (44.5 mg, 59.1 µmol, 92%).

$C_{40}H_{44}ClF_3N_4O_3S$, MW: 753.32 g/mol. **MP:** 190-197 °C. **$[\alpha]_D^{20}$ (*c* = 1.0 mg/mL, DCM)** = +142. **^{1}H-NMR (400 MHz, $CDCl_3$):** *δ* = 14.37 (*s*, 1 H, Ar-O*H*), 8.40 (*s*, 1 H, Ar-C*H*N), 8.18 (*s*, 1 H, *H*NTf), 7.91 (*d*, *J* = 1.9 Hz, 1 H, Ar-*H*), 7.45 (*d*, *J* = 6.8 Hz, 2 H, Ar-*H*), 7.16-6.94 (*m*, 12 H, Ar-*H*), 6.86 (*d*, *J* = 2.3 Hz, 1 H, Ar-*H*), 5.99 (*d*, *J* = 14.2 Hz, 1 H, Ar-C*H*$_2$-N), 5.53 (*d*, *J* = 10.4 Hz, 1 H, C*H*PhCHPh), 5.44 (*d*, *J* = 14.2 Hz, 1 H, Ar-C*H*$_2$-N), 4.90 (*d*, *J* = 10.4 Hz, 1 H,

CHPhC*H*Ph), 2.71 (*s*, 3 H, C-CH_3), 2.35 (*s*, 3 H, *p*-Mes-CH_3), 1.97 (*d*, *J* = 8.5 Hz, 6 H, *o*-Mes-(CH_3)$_2$), 1.18 (*s*, 9 H, Ar-C(CH_3)$_3$) ppm. **^{13}C-NMR (175 MHz, $CDCl_3$):** δ = 168.0, 157.7, 145.1, 141.5, 141.3, 140.3, 135.5, 135.2, 133.2, 130.5, 130.0, 129.9, 128.9, 128.8, 128.5, 128.4, 128.1, 127.6, 127.4, 123.8, 121.6, 120.4, 118.0, 66.0, 48.2, 34.3, 31.5, 21.3, 17.4, 17.3, 10.2 ppm. **^{19}F-NMR (376 MHz, $CDCl_3$):** δ = –77.85 (*s*, 3 F, SO_2CF_3) ppm. **IR ($CDCl_3$):** $\tilde{\nu}$ **=** 3061, 3032, 2961, 2866, 2676, 1629, 1602, 1521, 1480, 1455, 1455, 1370, 1286, 1266, 1226, 1191, 1148, 1050, 1032, 957, 925, 911, 855, 827, 794, 774, 758, 729, 699, 640, 640, 640, 628, 599, 571, 511, 496, 473 cm^{-1}. **HRMS (ESI) *m/z*:** calculated for $C_{40}H_{44}F_3N_4O_3S$ [M-Cl]$^+$: 717.3081; measured 717.3068.

4.11 *N*-((1*R*,2*R*)-2-(((*E*)-3-Benzyl-5-(*tert*-butyl)-2-hydroxybenzylidene)amino)-1,2-diphenylethyl)-1,1,1-trifluoromethanesulfonamide S46

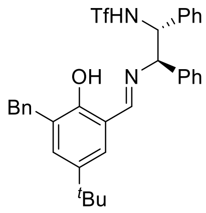

S46

Aldehyde **S45** (55.8 mg, 0.21 mmol, 1.00 eq.) and sulfonamide **7** (71.8 mg, 0.21 mmol, 1.00 eq.) were dissolved in dry ethanol (3 mL) and stirred with MS 3 Å under reflux for 3 h. The reaction mixture was filtered over Celite®, and the solvent was removed under reduced pressure. Preligand **S46** was dried under high vacuum and obtained as a yellow solid (123.7 mg, 0.21 mmol, >99%).

$C_{33}H_{33}F_3N_2O_3S$, MW: 594.69 g/mol. **MP:** 64-71 °C. **$[\alpha]_D^{20}$ (*c* = 1.0 mg/mL, DCM)** = +8. **^{1}H-NMR (400 MHz, $CDCl_3$):** δ = 12.52 (*s*, 1 H, Ar-O*H*), 8.15 (*s*, 1 H, Ar-C*H*N), 7.43-7.27 (*m*, 12 H, Ar-*H*), 7.24-7.17 (*m*, 2 H, Ar-*H*), 7.15-7.07 (*m*, 2 H, Ar-*H*), 6.97 (*d*, *J* = 2.5 Hz, 1 H, Ar-*H*), 5.04 (*d*, *J* = 4.5 Hz, 1 H, C*H*PhCHPh), 4.67 (*d*, *J* = 4.6 Hz, 1 H, CHPhC*H*Ph), 4.07 (*s*, 2 H, Ar-CH_2), 1.21 (*s*, 9 H, Ar-C(CH_3)$_3$) ppm. **^{13}C-NMR (175 MHz, $CDCl_3$):** δ = 168.7, 156.5, 141.6, 140.6, 138.1, 137.4, 131.9, 129.0, 128.9, 128.8, 128.7, 128.6, 128.5, 128.4, 128.4, 128.4, 127.5, 126.9, 126.8, 126.3, 126.0, 125.9, 121.9, 120.0, 118.2, 117.2, 116.4, 64.6, 35.5, 33.9, 31.3, 31.3 ppm. **^{19}F-NMR (376 MHz, $CDCl_3$):** δ = –77.51 (*s*, 3 F, SO_2CF_3) ppm. **IR ($CDCl_3$):** $\tilde{\nu}$ = 3304, 3064, 3031, 2961, 2905, 2869, 1627, 1602, 1494, 1455, 1376, 1271, 1228, 1198, 1144, 1051, 1029, 950, 911, 825, 772, 751, 733, 698, 604, 571, 510, 466 cm^{-1}. **HRMS (ESI) *m/z*:** calculated for $C_{33}H_{34}F_3N_2O_3S$ [M+H]$^+$: 595.2237; measured 595.2231.

4.12 3-(5-(*tert*-Butyl)-3-((*E*)-(((1*R*,2*R*)-1,2-di(naphthalen-1-yl)-2-((trifluoromethyl)sulfonamido)ethyl)imino)methyl)-2-hydroxybenzyl)-1-mesityl-1*H*-imidazol-3-ium chloride S36

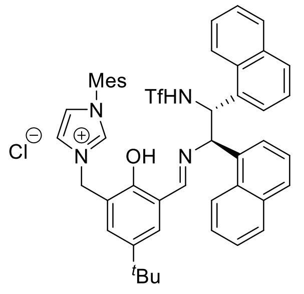

S36

The synthesis of preligand **S36** was carried out according to **GP 4** using aldehyde **S9** (28.4 mg, 68.8 µmol, 1.00 eq.) and sulfonamide **S18** (30.6 mg, 68.8 µmol, 1.00 eq.). After workup, preligand **S36** was obtained as a yellow solid (35.7 mg, 42.5 µmol, 92%).

$C_{47}H_{46}ClF_3N_4O_3S$, MW: 839.42 g/mol. **MP:** 199 °C (decomposition). **$[\alpha]_D^{20}$ (*c* = 1.0 mg/mL, DCM)** = +142. **^{1}H-NMR (400 MHz, $CDCl_3$):** δ = 14.47 (*s*, 1 H, Ar-O*H*), 11.62 (*br*, 1 H, Ar-C*H*N), 10.40 (*s*, 1 H, NC*H*N), 8.80 (*d*, *J* = 8.80 Hz, 1 H, Ar-*H*), 8.44-8.22 (*m*, 3 H, Ar-*H*), 7.58-7.27 (*m*, 9 H, Ar-*H*), 7.17-6.91 (*m*, 6 H, Ar-*H*), 6.65 (*s*, 1 H, Ar-C*H*HN), 6.42 (*d*, *J* = 13.6 Hz, 2 H, Ar-CH*H*N, C*H*PhCHPh), 5.90 (*d*, *J* = 14.0 Hz, 1 H, CHPhC*H*Ph), 2.34 (*s*, 3 H, *p*-Mes-C*H*₃), 2.06 (*d*, *J* = 38.1 Hz, 6 H, *o*-Mes-$(CH_3)_2$), 1.08 (*s*, 9 H, Ar-$C(CH_3)_3$) ppm. **^{13}C-NMR (175 MHz, $CDCl_3$):** δ = 167.7 156.8 141.7 141.1 138.4 134.9 134.5 133.2 132.8 131.3 130.5 129.95 129.86 128.9 128.4 128.1 127.9 127.2 126.4 126.0 125.5 124.8 124.6 124.3 122.45 122.16 120.5 118.7 118.1 48.6 34.2 31.5 21.2 17.6 17.3 ppm. **^{19}F-NMR (376 MHz, $CDCl_3$):** δ = –78.09 (*s*, 3 F, SO_2CF_3) ppm. **IR ($CDCl_3$):** $\tilde{\nu}$ = 2961, 2864, 1627, 1599, 1545, 1513, 1478, 1461, 1366, 1281, 1263, 1225, 1187, 1148, 1066, 1035, 950, 909, 855, 826, 800, 778, 728, 670, 637, 598, 574, 552, 516, 505, 482, 445 cm^{-1}. **HRMS (ESI) *m/z*:** calculated for $C_{47}H_{46}F_3N_4O_3S$ $[M-Cl]^+$: 803.3237; measured 803.3222.

4.13 3-(5-(*tert*-Butyl)-3-((*E*)-(((1*R*,2*R*)-1,2-di(naphthalen-2-yl)-2-((trifluoromethyl)sulfonamido)ethyl)imino)methyl)-2-hydroxybenzyl)-1-mesityl-1*H*-imidazol-3-ium chloride S37

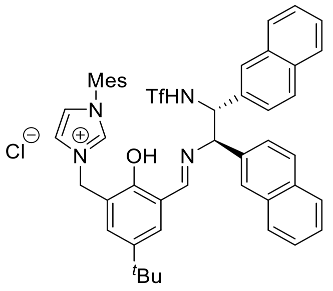

S37

The synthesis of preligand **S37**was carried out according to **GP 4** using aldehyde **S9** (19.1 mg, 46.3 µmol, 1.00 eq.) and sulfonamide **S19** (20.6 mg, 46.3 µmol, 1.00 eq.). After workup, preligand **S37** was obtained as a yellow solid (35.7 mg, 42.5 µmol, 92%).

$C_{47}H_{46}ClF_3N_4O_3S$, MW: 839.42 g/mol. **MP:** 192 °C (decomposition). **$[\alpha]_D^{20}$ (*c* = 1.0 mg/mL, DCM)** = +128. **^{1}H-NMR (400 MHz, $CDCl_3$):** *δ* = 14.38 (*br*, 1 H, Ar-O*H*), 11.57 (*br*, 1 H, N*H*Tf), 10.28 (*s*, 1 H, Ar-C*H*N), 8.34-8.18 (*m*, 2 H, Ar-*H*), 8.14 (*s*, 1 H, Ar-*H*), 7.78 (*m*, 2 H, Ar-*H*), 7.68-7.62 (*m*, 3 H, Ar-*H*), 7.61-7.56 (*m*, 3 H, Ar-*H*), 7.51 (*s*, 1 H, Ar-*H*), 7.38-7.27 (*m*, 4 H, Ar-*H*), 7.25-7.20 (*m*, 1 H, Ar-*H*), 6.98 (*s*, 3 H, Ar-*H*), 6.73 (*s*, 1 H, Ar-*H*), 6.32 (*d*, *J* = 14.0 Hz, 1 H, Ar-C*H*H-N), 5.91 (*br*, 1 H, C*H*), 5.82 (*d*, *J* = 14.0 Hz, 1 H, Ar-CH*H*-N), 5.35 (*d*, *J* = 10.7 Hz, 1 H, C*H*), 2.34 (*s*, 3 H, *p*-Mes-CH_3), 2.03 (*d*, *J* = 35.8 Hz, 6 H, *o*-Mes-($CH_3)_2$), 1.11 (*s*, 9 H, Ar-C($CH_3)_3$) ppm**^{13}C-NMR (175 MHz, $CDCl_3$):** *δ* = 168.1 156.9 141.5 140.9 138.1 137.0 134.8 134.3 133.03 132.96 132.9 132.7 132.6 131.1 129.8 129.7 129.1 128.8 128.4 128.3 128.1 127.9 127.52 127.47 126.1 126.0 125.74 125.67 125.5 124.3 122.3 122.0 120.4 118.6 117.9 65.8 48.5 34.1 31.3 21.1 17.5 17.2 ppm. **^{19}F-NMR (376 MHz, $CDCl_3$):** *δ* = –77.73 (*s*, 3 F, SO_2CF_3) ppm. **IR ($CDCl_3$):** $\tilde{\nu}$ = 2961, 2862, 1629, 1601, 1546, 1509, 1480, 1368, 1262, 1226, 1190, 1149, 1127, 1066, 948, 909, 857, 819, 731, 667, 647, 628, 607, 577, 516, 478, 442, 418, 3142, 3053, 2807 cm^{-1}. **HRMS (ESI) *m/z*:** calculated for $C_{47}H_{46}F_3N_4O_3S$ [M-Cl]$^+$: 803.3237; measured 803.3202.

4.14 3-(3-((*E*)-(((1*R*,2*R*)-1,2-Bis(4-fluorophenyl)-2-((trifluoromethyl)sulfonamido)ethyl)imino)methyl)-5-(*tert*-butyl)-2-hydroxybenzyl)-1-mesityl-1*H*-imidazol-3-ium chloride S38

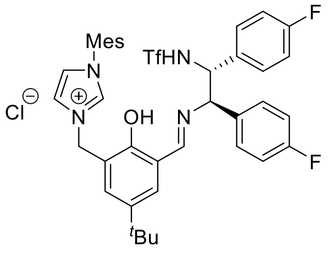

S38

The synthesis of preligand **S38** was carried out according to **GP 4** using aldehyde **S9** (28.1 mg, 67.9 µmol, 1.00 eq.) and sulfonamide **S20** (25.8 mg, 67.9 µmol, 1.00 eq.). After workup, preligand **S38** was obtained as a yellow solid (58.1 mg, 66.8 µmol, 98%).

$C_{39}H_{40}ClF_5N_4O_3S$, MW: 775.28 g/mol. **MP:** 186-188 °C. **$[\alpha]_D^{20}$ (*c* = 1.0 mg/mL, DCM)** = +29. **^{1}H-NMR (400 MHz, $CDCl_3$):** *δ* = 14.12 (*s*, 1 H, Ar-O*H*), 11.47 (*br*, 1 H, N*H*Tf), 10.19 (*s*, 1 H, NC*H*N), 8.25-8.02 (*m*, 3 H, Ar-*H*), 7.40 (*t*, *J* = 3.9 Hz, 2 H, Ar-*H*), 7.00-6.94 (*m*, 5 H, Ar-*H*), 6.86-6.79 (*m*, 5 H, Ar-*H*), 6.15 (*d*, *J* = 13.3 Hz, 1 H, Ar-C*H*HN), 5.69 (*d*, *J* = 13.3 Hz, 1 H, Ar-CH*H*N), 5.50 (*br*, 1 H, C*H*PhCHPh), 4.92 (*d*, *J* = 10.6 Hz, 1 H, CHPhC*H*Ph), 2.33 (*s*, 3 H, *p*-Mes-CH_3), 2.06 (*s*, 3 H, *o*-Mes-CH_3), 1.95 (*s*, 3 H, *o*-Mes-CH_3), 1.13 (*s*, 9 H, Ar-C(CH_3)$_3$) ppm. **^{13}C-NMR (175 MHz, $CDCl_3$):** *δ* = 168.4, 162.9, 162.8, 161.5, 161.4, 156.8, 141.9, 141.2, 138.2, 136.0, 134.8, 134.3, 133.2, 131.1, 130.6, 130.11, 130.06, 130.0, 129.9, 129.0, 124.1, 122.3, 122.2, 118.0, 115.7, 115.6, 115.3, 115.2, 65.4, 48.5, 34.3, 31.5, 21.2, 17.6, 17.3 ppm. **^{19}F-NMR (376 MHz, $CDCl_3$):** *δ* = −77.77 (*s*, 3 F, SO_2CF_3), −113.74 (*br*, 1 F, Ar-F), −114.56 (*br*, 1 F, Ar-F) ppm. **IR ($CDCl_3$):** $\tilde{\nu}$ = 2958, 2927, 2857, 1629, 1604, 1546, 1511, 1480, 1371, 1285, 1225, 1192, 1150, 1099, 1058, 1016, 957, 911, 856, 840, 791, 732, 670, 641, 610, 579, 570, 532, 507, 480 cm^{-1}. **HRMS (ESI) *m/z*:** calculated for $C_{39}H_{40}F_5N_4O_3S$ [M-Cl]$^+$: 739.2736; measured 739.2711.

4.15 3-(3-((*E*)-(((1*R*,2*R*)-1,2-Bis(4-nitrophenyl)-2-((trifluoromethyl)sulfonamido)ethyl)imino)methyl)-5-(*tert*-butyl)-2-hydroxybenzyl)-1-mesityl-1*H*-imidazol-3-ium chloride S39

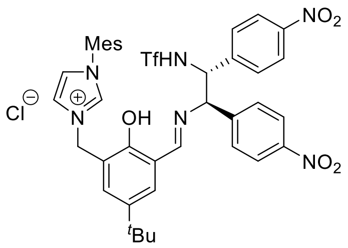

S39

The synthesis of preligand **S39** was carried out according to **GP 4** using aldehyde **S9** (9.6 mg, 23.3 µmol, 1.00 eq.) and sulfonamide **S21** (8.4 mg, 23.3 µmol, 1.00 eq.). After purification by column chromatography on silica gel (DCM/MeOH, 10:1), preligand **S39** was obtained as a yellow solid (15.0 mg, 18.1 µmol, 77%).

$C_{39}H_{40}ClF_3N_6O_7S$, MW: 829.29 g/mol. **MP:** 172-176 °C. **$[\alpha]_D^{20}$ (*c* = 1.0 mg/mL, DCM)** = +275. **^{1}H-NMR (400 MHz, CDCl$_3$):** *δ* = 14.25 (*br*, 1 H, Ar-O*H*), 9.02 (*s*, 1 H, NC*H*N), 8.54 (*s*, 1 H, Ar-C*H*N), 8.01 (*d*, *J* = 8.6 Hz, 2 H, Ar-*H*), 7.95 (*d*, *J* = 8.6 Hz, 2 H, Ar-*H*), 7.72 (*s*, 1 H, Ar-*H*), 7.51 (*s*, 1 H, Ar-*H*), 7.38-7.26 (*m*, 5 H, Ar-*H*), 7.03 (*br*, 1 H, Ar-*H*), 6.98 (*br*, 2 H, Ar-*H*), 5.70 (*d*, *J* = 13.9 Hz, 1 H, Ar-C*H*HN), 5.30 (*d*, *J* = 13.9 Hz, 1 H, Ar-CH*H*N), 4.88 (*d*, *J* = 8.6 Hz, 1 H, C*H*PhCHPh), 4.46 (*br*, 1 H, CHPhC*H*Ph), 2.33 (*s*, 3 H, *p*-Mes-C*H*$_3$), 1.99 (*s*, 3 H, *o*-Mes-C*H*$_3$), 1.90 (*s*, 3 H, *o*-Mes-C*H*$_3$), 1.15 (*s*, 9 H, Ar-C(C*H*$_3$)$_3$) ppm. **^{13}C-NMR (175 MHz, CDCl$_3$):** *δ* = 157.6, 148.0, 147.2, 146.8, 142.7, 141.7, 137.1, 134.2, 131.5, 130.6, 130.6, 130.2, 130.0, 130.0, 129.9, 129.0, 128.9, 123.6, 123.5, 123.2, 123.1, 120.5, 118.8, 49.4, 34.2, 31.4, 29.9, 22.8, 21.2, 17.4, 17.3, 14.3 ppm. **^{19}F-NMR (376 MHz, CDCl$_3$):** *δ* = –78.13 (*s*, 3 F, SO_2CF_3) ppm. **IR (CDCl$_3$):** $\tilde{\nu}$ = 2955, 2924, 2855, 1632, 1599, 1520, 1481, 1463, 1345, 1270, 1222, 1196, 1152, 1107, 1086, 1068, 933, 910, 857, 829, 788, 730, 699, 648, 607, 574 cm^{-1}. **HRMS (ESI) *m/z*:** calculated for $C_{39}H_{40}F_3N_6O_7S$ [M-Cl]$^+$: 793.2626; measured 793.2626.

4.16 3-(5-(*tert*-Butyl)-2-hydroxy-3-((*E*)-(((1*R*,2*R*)-2-((trifluoromethyl)sulfonamido)cyclohexyl)imino)methyl)benzyl)-1-mesityl-1*H*-imidazol-3-ium chloride S40

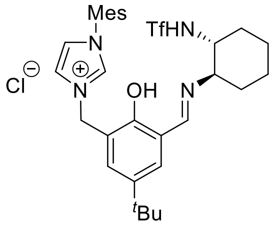

S40

The synthesis of preligand **S40** was carried out according to **GP 4** using aldehyde **S9** (29.4 mg, 71.1 µmol, 1.00 eq.) and sulfonamide **S25** (17.5 mg, 71.1 µmol, 1.00 eq.). After purification by column chromatography on silica gel (DCM/MeOH, 10:1) and subsequent precipitation from a solution in DCM into *n*-pentane (20 mL), preligand **S40** was obtained as a yellow solid (39.2 mg, 61.1 µmol, 86%).

$C_{31}H_{40}ClF_3N_4O_3S$, MG: 641.19 g/mol. **MP:** 175-180 °C. **$[\alpha]_D^{20}$ (*c* = 1.0 mg/mL, DCM)** = −16. **^{1}H-NMR (400 MHz, $CDCl_3$):** *δ* = 14.36 (*s*, 1 H, Ar-O*H*), 9.80 (*s*, 1 H, Ar-C*H*N), 8.56 (*s*, 1 H, NC*H*N), 8.05 (*s*, 1 H, Ar-*H*), 7.82 (*d*, *J* = 2.1 Hz, 1 H, Ar-*H*), 7.18 (*d*, *J* = 2.1Hz, 1 H, Ar-*H*), 6.97-6.90 (*m*, 3 H, Ar-*H*), 5.76 (*d*, *J* = 13.9 Hz, 1 H, Ar-C*H*HN), 5.64 (*d*, *J* = 13.9 Hz, 1 H, Ar-CH*H*N), 3.96-3.79 (*m*, 1 H, C*H*), 3.52-3.36 (*m*, 1 H, C*H*), 2.30 (*s*, 3 H, *p*-Mes-CH_3), 2.13-2.02 (*m*, 1 H, C*H*), 2.00 (*s*, 3 H, *o*-Mes-CH_3), 1.91 (*s*, 3 H, *o*-Mes-CH_3), 1.92-1.65 (*m*, 3 H, CH_2, C*H*), 1.55-1.39 (*m*, 2 H, CH_2), 1.37-1.24 (*m*, 2 H, CH_2), 1.19 (*s*, 9 H, Ar-C(CH_3)$_3$) ppm. **^{13}C-NMR (175 MHz, $CDCl_3$):** *δ* = 165.9, 159.4, 141.1, 141.1, 137.5, 134.5, 134.4, 132.3, 131.0, 129.9, 129.8, 124.2, 122.6, 121.3, 120.8, 119.0, 118.1, 117.1, 68.2, 59.9, 49.3, 34.2, 33.8, 32.8, 31.4, 25.2, 23.9, 21.2, 17.6, 17.5 ppm. **^{19}F-NMR (376 MHz, $CDCl_3$):** *δ* = −78.13 (*s*, 3 F, SO_2CF_3) ppm. **IR ($CDCl_3$):** $\tilde{\nu}$ = 3146, 2952, 2862, 2801, 2739, 2692, 2197, 1631, 1605, 1546, 1481, 1449, 1368, 1285, 1227, 1186, 1153, 1142, 1091, 1066, 1038, 971, 909, 854, 826, 790, 728, 670, 643, 607, 574, 517, 472, 442 cm^{-1}. **HRMS (ESI) *m/z*:** calculated for $C_{31}H_{40}F_3N_4O_3S$ $[M-Cl]^+$: 605.2774; measured 605.2768.

4.17 (*R,E*)-3-(5-(*tert*-Butyl)-2-hydroxy-3-(((2'-((trifluoromethyl)sulfonamido)-[1,1'-binaphthalen]-2-yl)imino)methyl)benzyl)-1-mesityl-1*H*-imidazol-3-ium chloride S41

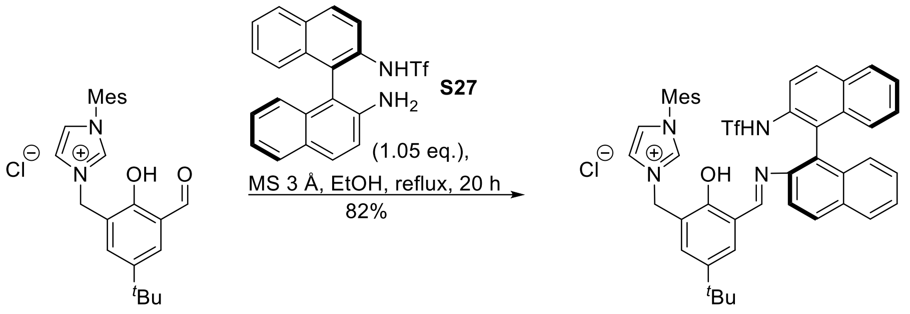

S9 **S41**

Aldehyde **S9** (18.9 mg, 45.8 µmol, 1.00 eq.) and sulfonamide **S27** (20.0 mg, 48.1 µmol, 1.05 eq.) were placed with MS 3 Å in a pre-dried flask, dissolved in dry ethanol (3 mL), and stirred under reflux overnight. The reaction mixture was filtered over Celite®, and the solvent was removed under reduced pressure. The resulting solid was redissolved in DCM (0.4 mL) and precipitated from *n*-pentane/Et_2O (10:1, 10 mL), followed by washing with *n*-pentane/Et_2O (10:1, 10 mL). Preligand **S41** was dried under high vacuum and obtained as an orange solid (30.4 mg, 37.5 µmol, 82%).

$C_{45}H_{42}ClF_3N_4O_3S$, MW: 811.36 g/mol. **MP:** 195-200 °C. **$[\alpha]_D^{20}$ (*c* = 1.0 mg/mL, DCM)** = +775. **^{1}H-NMR (400 MHz, CD_2Cl_2):** δ = 13.65 (*br*, 1 H, Ar-O*H*), 8.84 (*br*, 1 H, Ar-C*H*N), 8.73 (*s*, 1 H, NC*H*N), 8.04 (*d*, *J* = 8.0 Hz, 1 H, Ar-*H*), 7.93 (*d*, *J* = 7.5 Hz, 1 H, Ar-*H*), 7.86 (*d*, *J* = 8.9 Hz, 1 H, Ar-*H*), 7.70 (*t*, *J* = 9.0 Hz, 2 H, Ar-*H*), 7.65-7.38 (*m*, 4 H, Ar-*H*), 7.31 (*s*, 1 H, Ar-*H*), 7.25 (*d*, *J* = 2.8 Hz, 2 H, Ar-*H*), 7.17-6.98 (*m*, 5 H, Ar-*H*), 6.89 (*d*, *J* = 8.0 Hz, 1 H, Ar-*H*), 5.14 (*s*, 2 H, Ar-CH_2-N), 2.39 (*s*, 3 H, *p*-Mes-CH_3), 1.95 (*s*, 6 H, *o*-Mes-CH_3), 1.23 (*s*, 9 H, Ar-C(CH_3)$_3$) ppm. **^{13}C-NMR (175 MHz, CD_2Cl_2):** δ = 197.0, 160.0, 158.1, 157.4, 144.1, 142.8, 141.8, 141.5, 141.44, 141.36, 138.4, 137.4, 136.8, 134.6, 134.3, 133.7, 133.7, 133.6, 132.9, 132.8, 132.1, 131.6, 131.3, 130.9, 130.8, 130.7, 130.1, 130.0, 129.8, 129.7, 129.2, 128.4, 128.3, 128.3, 128.1, 127.8, 127.4, 127.3, 127.1, 126.6, 126.1, 126.1, 126.0, 125.7, 125.0, 124.9, 124.2, 123.9, 123.5, 123.1, 122.8, 121.9, 120.7, 120.6, 120.5, 118.9, 118.3, 116.1, 49.2, 47.6, 34.4, 34.0, 31.1, 31.0, 20.94, 20.88, 17.33, 17.26 ppm (rotameric mixture). **^{19}F-NMR (376 MHz, CD_2Cl_2):** δ = –77.36 (*s*, 3 F, SO_2CF_3) ppm. **IR (CD_2Cl_2):** $\tilde{\nu}$ = 3133, 3055, 2957, 2867, 1618, 1585, 1561, 1546, 1503, 1470, 1425, 1363, 1345, 1291, 1196, 1162, 1068, 1040, 1018, 967, 878, 853, 819, 789, 749, 714, 632, 598, 555, 510 cm^{-1}. **HRMS (ESI) *m/z*:** calculated for $C_{45}H_{42}F_3N_4O_3S$ [M-Cl]$^+$: 775.2924; measured 775.2917.

5 Complex Synthesis

5.1 1-(5-(*tert*-Butyl)-3-((*E*)-(((1*R*,2*R*)-1,2-diphenyl-2-((trifluoromethyl)sulfonamido-κ*N*-)ethyl)imino-κ*N*-)methyl)-2-hydroxy-κ*O*-benzyl)-3-ethyl-4-mesityl-1*H*-1,2,3-triazol-3-ium aqua-κ*O* copper(II) hexafluorophosphate(V) C1

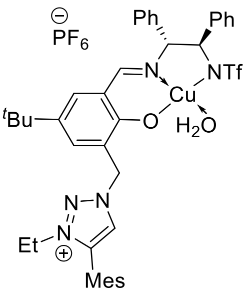

C1

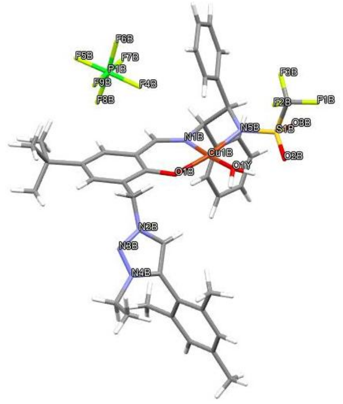

Complex **C1** was synthesized according to **GP 5** using preligand **S28** (30.0 mg, 34.2 µmol, 1.00 eq.) and $Cu(acac)_2$ (9.0 mg, 34.2 µmol, 1.00 eq.). After workup, complex **C1** was obtained as a green solid (31.4 mg, 33.4 µmol, 98%).

$C_{40}H_{43}CuF_9N_5O_3PS$, MW: 939.38 g/mol. **MP:** 177 °C (decomposition). **$[\alpha]_D^{20}$ (*c* = 1.0 mg/mL, DCM)** = +81.6. **NMR:** paramagnetic species. **IR ($CDCl_3$):** $\tilde{\nu}$ = 2963, 1628, 1550, 1452, 1321, 1212, 1187, 1149, 1072, 843, 700, 613, 558 cm^{-1}. **HRMS (ESI) *m/z*:** calculated for $C_{40}H_{43}CuN_5O_3S$ $[M-PF_6]^+$: 793.2315; measured 793.2329.

UV-Vis (DCM, *c* = 2 · 10^{-5} M):

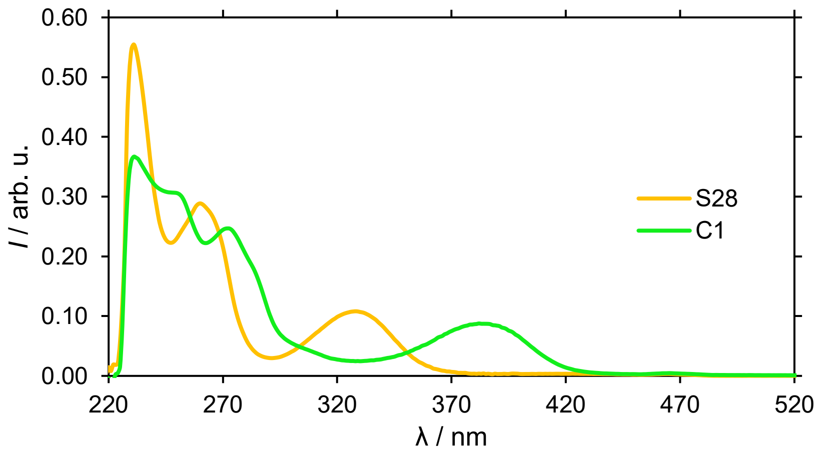

CCDC 2497500 contains supplementary crystallographic data for compound **C1**. These data are provided free of charge by the joint Cambridge Crystallographic Data Centre and Fachinformationszentrum Karlsruhe www.ccdc.cam.ac.uk/structures/.

5.2 3-(5-(*tert*-Butyl)-3-((*E*)-(((1*R*,2*R*)-1,2-diphenyl-2-((trifluoromethyl)sulfonamido-κ*N*-)ethyl)imino-κ*N*-)methyl)-2-hydroxybenzyl-κ*O*)-1-phenyl-1*H*-benzo[*d*]imidazol-3-ium copper(II) chloride C2

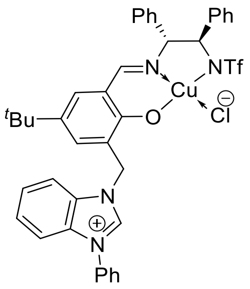

C2

Complex **C2** was synthesized according to **GP 5** using previously reported preligand[1] (24.4 mg, 32.7 μmol, 1.00 eq.) and Cu(acac)$_2$ (8.5 mg, 32.7 μmol, 1.00 eq.). After workup, complex **C2** was obtained as a green solid (24.4 mg, 30.2 μmol, 92%).

$C_{40}H_{36}ClCuF_3N_4O_3S$, MW: 808.80 g/mol. **NMR:** paramagnetic species. **HRMS (ESI) *m/z*:** calculated for $C_{40}H_{36}CuF_3N_4O_3S$ [M-Cl]$^+$: 772.1751; measured 772.1746.

UV-Vis (DCM, *c* = 2 · 10^{-5} M):

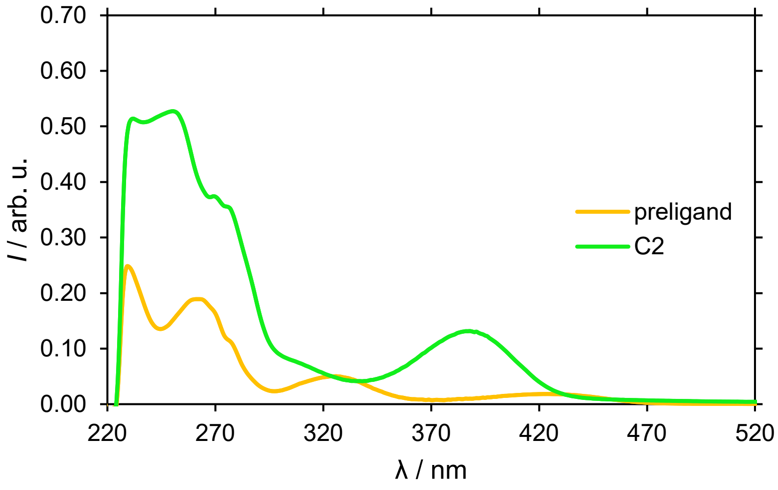

The analytical data are consistent with the literature.[1]

5.3 3-(5-(*tert*-Butyl)-3-((*E*)-(((1*R*,2*R*)-1,2-diphenyl-2-((trifluormethyl)sulfonamido-κ*N*-)ethyl)imino-κ*N*-)methyl)-2-hydroxy-κ*O*-benzyl)-1-methyl-1*H*-imidazol-3-ium copper(II) chloride C3

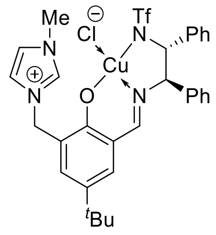

C3

Complex **C3** was synthesized according to **GP 5** using preligand **S29** (38.7 mg, 60.9 µmol, 1.00 eq.) and $Cu(acac)_2$ (16.0 mg, 60.9 µmol, 1.00 eq.). After workup, complex **C3** was obtained as a green solid (40.0 mg, 57.4 µmol, 94%).

$C_{31}H_{32}ClCuF_3N_4O_3S$, MW: 696.67 g/mol. **NMR:** paramagnetic species. **HRMS (ESI) *m/z*:** calculated for $C_{31}H_{32}CuF_3N_4O_3S$ $[M\text{-}Cl]^+$: 660.1438; measured 660.1431.

UV-Vis (DCM, *c* = 2 · 10^{-5} M):

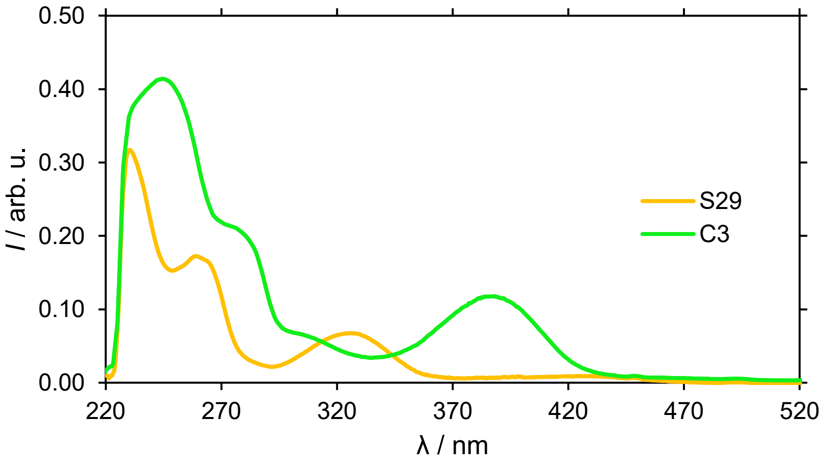

The analytical data are consistent with the literature.[19]

5.4 3-(5-(*tert*-Butyl)-3-((*E*)-(((1*R*,2*R*)-1,2-diphenyl-2-((trifluormethyl)sulfonamido-κ*N*-)ethyl)imino-κ*N*-)methyl)-2-hydroxy-κ*O*-benzyl)-1-(2,6-diisopropylphenyl)-1*H*-imidazol-3-ium copper(II) chloride C4

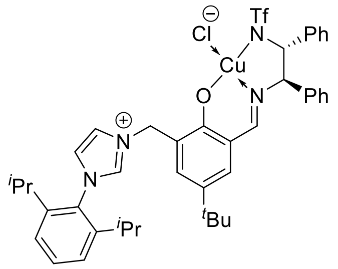

C4

Complex **C4** was synthesized according to **GP 5** using preligand **S30** (16.0 mg, 20.5 µmol, 1.00 eq.) and Cu(acac)$_2$ (5.4 mg, 20.5 µmol, 1.00 eq.). After workup, complex **C4** was obtained as a green solid (12.4 mg, 14.7 µmol, 72%).

$C_{44}H_{46}ClCuF_3N_4O_3S$, MW: 842.91 g/mol. **MP:** 207 °C (decomposition). **$[\alpha]_D^{20}$ (*c* = 1.0 mg/mL, DCM)** = +290. **NMR:** paramagnetic species. **IR (CDCl$_3$):** $\tilde{\nu}$ = 2965, 1626, 1543, 1453, 1392, 1324, 1212, 1185, 1072, 997, 943, 765, 700, 610, 513, 411 cm^{-1}. **HRMS (ESI) *m/z*:** calculated for $C_{42}H_{46}CuF_3N_4O_3S$ [M-Cl]$^+$: 806.2533; measured 806.2514.

UV-Vis (DCM, *c* = 2 · 10^{-5} M):

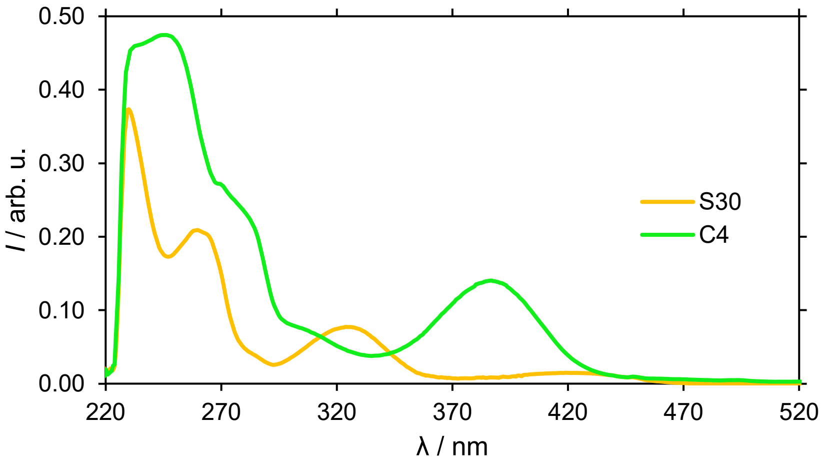

5.5 3-(5-(*tert*-Butyl)-3-((*E*)-(((1*R*,2*R*)-1,2-diphenyl-2-((trifluoromethyl)sulfonamido-*κN*-)ethyl)imino-*κN*-)methyl)-2-hydroxybenzyl-*κO*)-1-mesityl-1*H*-imidazol-3-ium copper(II) chloride C5

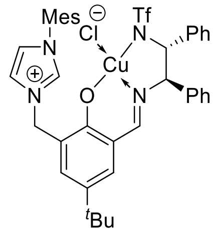

C5

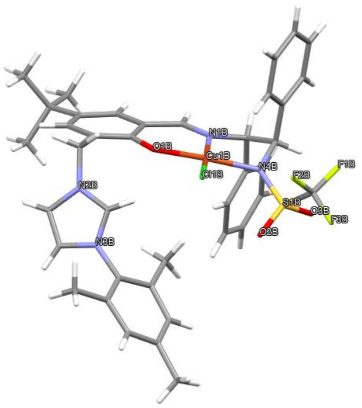

Complex **C5** was synthesized according to **GP 5** using preligand **8** (13.0 mg, 17.6 µmol, 1.00 eq.) and $Cu(acac)_2$ (4.6 mg, 17.6 µmol, 1.00 eq.). After workup, complex **C5** was obtained as a green solid (13.0 mg, 16.2 µmol, 92%).

$C_{39}H_{40}ClCuF_3N_4O_3S$, MW: 800.83 g/mol. **MP:** 208 °C (decomposition). **$[\alpha]_D^{20}$ (*c* = 1.0 mg/mL, DCM)** = +442. **NMR:** paramagnetic species. **IR ($CDCl_3$):** $\tilde{\nu}$ = 3455, 3026, 2957, 1626, 1544, 1494, 1452, 1393, 1365, 1323, 1275, 1211, 1184, 1094, 1071, 1030, 997, 943, 911, 854, 839, 799, 785, 766, 731, 701, 671, 631, 610, 588 cm^{-1}. **HRMS (ESI) *m/z*:** calculated for $C_{39}H_{40}CuF_3N_4O_3S$ [M-Cl]$^+$: 764.2064; measured 764.2066. **EA (%):** calculated for $C_{39}H_{40}ClCuF_3N_4O_3S+H_2O$: C 57.21, H 5.17, N 6.84; found: C 57.50, H 5.23, N 6.86.

UV-Vis (DCM, *c* = 2 · 10^{-5} M):

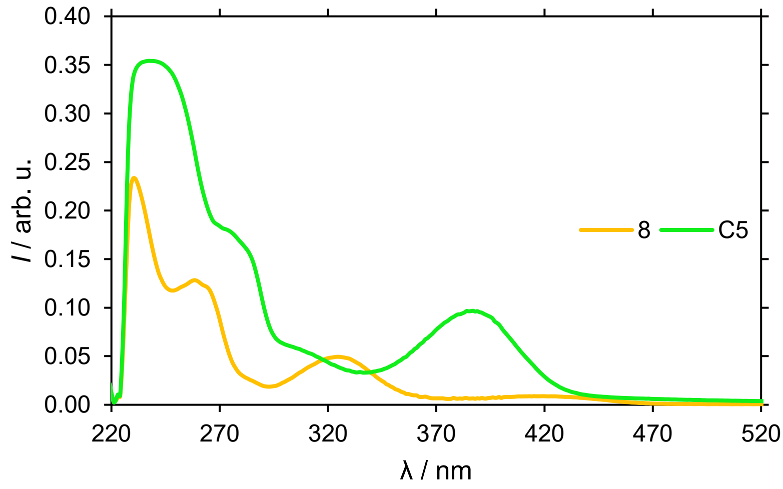

CCDC 2497503 contains supplementary crystallographic data for compound **C5**. These data are provided free of charge by the joint Cambridge Crystallographic Data Centre and Fachinformationszentrum Karlsruhe www.ccdc.cam.ac.uk/structures/.

5.6 3-(5-(*tert*-Butyl)-3-((*E*)-(((1*R*,2*R*)-1,2-diphenyl-2-((trifluoromethyl)sulfonamido-*κN*-)ethyl)imino-*κN*-)methyl)-2-hydroxy-*κO*-benzyl)-1-mesityl-1*H*-imidazol-3-ium copper(II) bromide C12

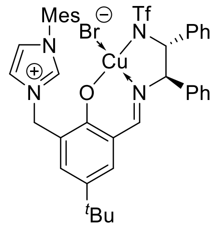

C12

Complex **C12** was synthesized according to **GP 5** using preligand **S31** (13.5 mg, 17.2 µmol, 1.00 eq.) and $Cu(acac)_2$ (4.5 mg, 17.2 µmol, 1.00 eq.). After workup, complex **C12** was obtained as a green solid (12.6 mg, 14.9 µmol, 87%).

$C_{39}H_{40}BrCuF_3N_4O_3S$, MW: 845.28 g/mol. **MP:** 205-210 °C (decomposition). **$[\alpha]_D^{20}$ (*c* = 1.0 mg/mL, DCM)** = +161. **NMR:** paramagnetic species. **IR (DCM):** $\tilde{\nu}$ = 3031, 2956, 1626, 1544, 1452, 1393, 1365, 1323, 1275, 1186, 1071, 1030, 996, 942, 839, 766, 701, 612, 517 cm^{-1}. **HRMS (ESI) *m/z*:** calculated for $C_{39}H_{40}CuF_3N_4O_3S$ $[M-Br]^+$: 764.2064; measured 764.2058.

UV-Vis (DCM, *c* = 2 · 10^{-5} M):

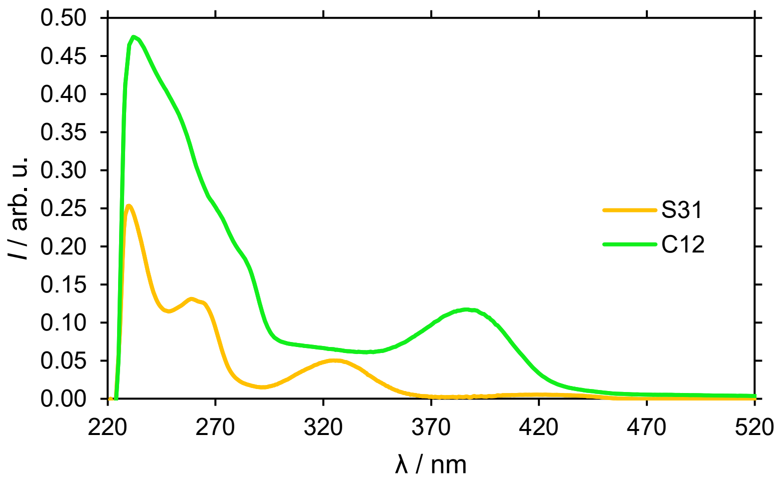

5.7 3-(5-(*tert*-Butyl)-3-((*E*)-(((1*R*,2*R*)-1,2-diphenyl-2-((trifluoromethyl)sulfonamido-κ*N*-)ethyl)imino-κ*N*-)methyl)-2-hydroxy-κ*O*-benzyl)-1-mesityl-1*H*-imidazol-3-ium copper(II) iodide C13

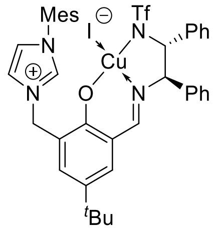

C13

Complex **C13** was synthesized according to **GP 5** using preligand **S32** (22.6 mg, 27.2 µmol, 1.00 eq.) and Cu(acac)$_2$ (7.1 mg, 27.2 µmol, 1.00 eq.). After workup, complex **C13** was obtained as a green solid (19.5 mg, 21.9 µmol, 80%).

$C_{39}H_{40}CuF_3IN_4O_3S$, MW: 892.28 g/mol. **MP:** 269 °C (decomposition). **$[\alpha]_D^{20}$ (*c* = 1.0 mg/mL, DCM)** = +193. **NMR:** paramagnetic species. **IR (DCM):** $\tilde{\nu}$ = 3028, 2958, 1626, 1544, 1494, 1451, 1393, 1365, 1321, 1275, 1211, 1185, 1070, 1029, 997, 942, 839, 766, 701, 633, 612, 515 cm^{-1}. **HRMS (ESI) *m/z*:** calculated for $C_{39}H_{40}CuF_3N_4O_3S$ [M-I]$^+$: 764.2064; measured 764.2054.

UV-Vis (DCM, *c* = 2 · 10^{-5} M):

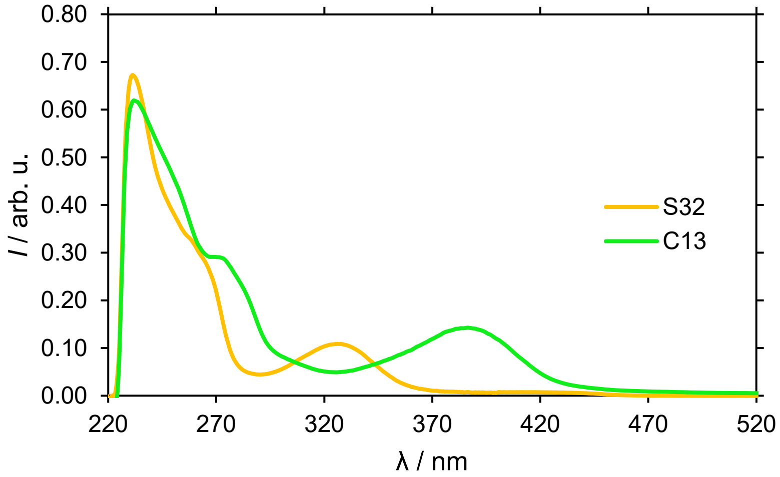

5.8 *N*-((1*R*,2*R*)-2-(((*E*)-5-(*tert*-Butyl)-2-hydroxy-*κO*-3-methylbenzylidene)amino-*κN*)-1,2-diphenylethyl)-1,1,1-trifluoromethanesulfonamide-*κN* copper(II) C6

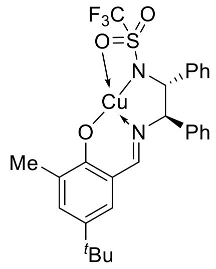

C6

Complex **C6** was synthesized according to **GP 5** using preligand **S33** (21.3 mg, 41.1 μmol, 1.00 eq.) and $Cu(acac)_2$ (10.8 mg, 41.1 μmol, 1.00 eq.). After filtration over Celite®, complex **C6** was obtained without further purification as a green solid (23.8 mg, 41.0 μmol, >99%).

$C_{27}H_{27}CuF_3N_2O_3S$, MW: 580.13 g/mol. **NMR:** paramagnetic species. **HRMS (ESI)** ***m/z*:** calculated for $C_{27}H_{28}CuF_3N_2O_3S$ $[M+H]^+$: 580.1063; measured 580.1067.

UV-Vis (DCM, $c = 2 \cdot 10^{-5}$ M):

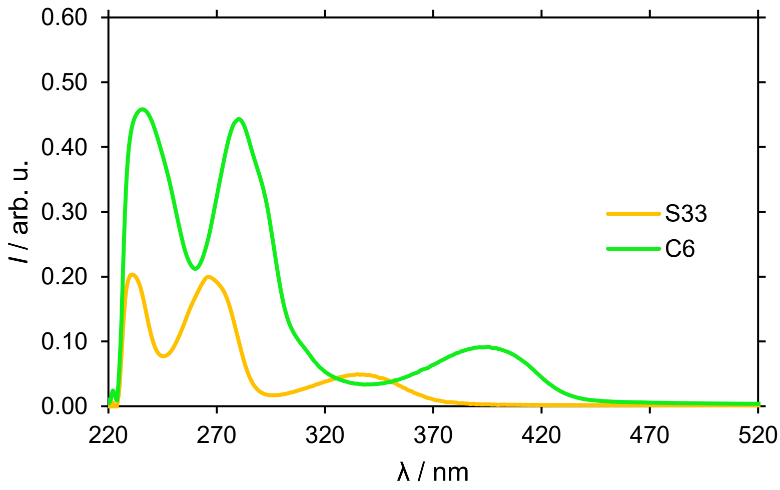

The analytical data are consistent with the literature.[20]

5.9 1-(5-(*tert*-Butyl)-3-((*E*)-(((1*R*,2*R*)-1,2-diphenyl-2-((trifluormethyl)sulfonamido-*κN*-)ethyl)imino-*κN*-)methyl)-2-hydroxy-*κO*-benzyl)pyridin-1-ium copper(II) chloride C7

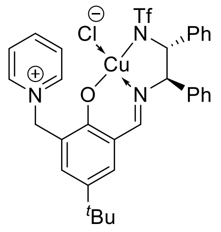

C7

Complex **C7** was synthesized according to **GP 5** using preligand **S35** (12.4 mg, 19.6 µmol, 1.00 eq.) and Cu(acac)$_2$ (5.1 mg, 19.6 µmol, 1.00 eq.). After workup, complex **C7** was obtained as a green solid (11.1 mg, 16.0 µmol, 82%).

$C_{32}H_{31}ClCuF_3N_3O_3S$, MW: 693.67 g/mol. **MP:** 216 °C (decomposition). **$[\alpha]_D^{20}$ (*c* = 1.0 mg/mL, DCM)** = +220. **NMR:** paramagnetic species. **IR (CDCl$_3$):** $\tilde{\nu}$ = 3062, 2961, 1626, 1544, 1486, 1452, 1392, 1321, 1212, 1187, 1073, 1029, 997, 944, 821, 773, 728, 693, 614, 513, 485 cm^{-1}. **HRMS (ESI) *m/z*:** calculated for $C_{32}H_{31}CuF_3N_3O_3S$ [M-Cl]$^+$: 657.1329; measured 657.1300.

UV-Vis (DCM, *c* = 2 · 10^{-5} M):

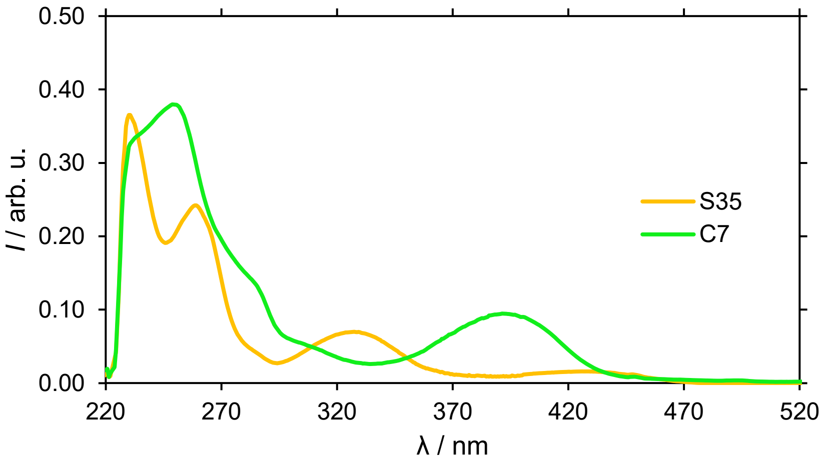

5.10 *N*-(5-(*tert*-Butyl)-3-((*E*)-(((1*R*,2*R*)-1,2-diphenyl-2-((trifluormethyl)sulfonamido-κ*N*-)ethyl)imino-κ*N*-)methyl)-2-hydroxy-κ*O*-benzyl)-*N*-ethyl-*N*-methylethaneaminium copper(II) chloride C8

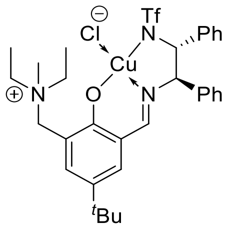

C8

Complex **C8** was synthesized according to **GP 5** using preligand **S34** (24.9 mg, 38.9 µmol, 1.00 eq.) and $Cu(acac)_2$ (10.2 mg, 38.9 µmol, 1.00 eq.). After workup, complex **C8** was obtained as a green solid (25.5 mg, 36.3 µmol, 93%).

$C_{32}H_{39}ClCuF_3N_3O_3S$, MW: 701.73 g/mol. **NMR:** paramagnetic species. **HRMS (ESI) *m/z*:** calculated for $C_{32}H_{39}CuF_3N_3O_3S$ $[M\text{-}Cl]^+$: 665.1955; measured 665.1946.

UV-Vis (DCM, $c = 2 \cdot 10^{-5}$ M):

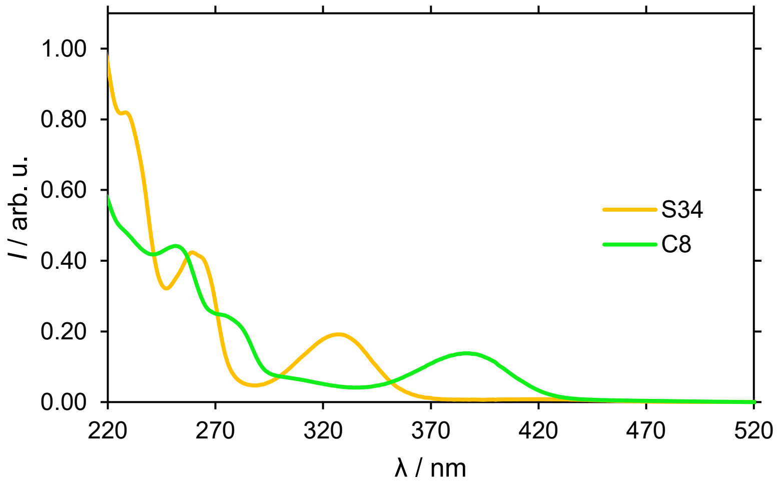

The analytical data are consistent with the literature.[21]

5.11 3-(5-(*tert*-Butyl)-3-((*E*)-(((1*R*,2*R*)-1,2-diphenyl-2-((trifluoromethyl)sulfonamido-κ*N*-)ethyl)imino-κ*N*-)methyl)-2-hydroxybenzyl-κ*O*)-1-mesityl-2-methyl-1*H*-imidazol-3-ium copper(II) chloride C9

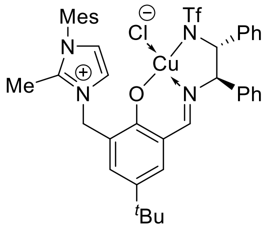

C9

Complex **C9** was synthesized according to **GP 5** using preligand **S43** (42.1 mg, 32.0 µmol, 1.00 eq.) and $Cu(acac)_2$ (8.4 mg, 32.0 µmol, 1.00 eq.). After workup, complex **C9** was obtained as a green solid (22.4 mg, 27.5 µmol, 86%).

$C_{40}H_{42}ClCuF_3N_4O_3S$, MW: 814.85 g/mol. **MP:** 203-210 °C (decomposition). **$[\alpha]_D^{20}$ (*c* = 1.0 mg/mL, DCM)** = +680. **NMR:** paramagnetic species. **IR (DCM):** $\tilde{\nu}$ = 3061, 3026, 2954, 1627, 1544, 1521, 1495, 1451, 1392, 1365, 1325, 1273, 1245, 1212, 1186, 1073, 1030, 995, 942, 766, 702, 658, 615, 564, 513, 471, 446, 429 cm^{-1}. **HRMS (ESI) *m/z*:** calculated for $C_{40}H_{42}CuF_3N_4O_3S$ $[M-Cl]^+$: 778.2220; measured 778.2199.

UV-Vis (DCM, *c* = 2 · 10^{-5} M):

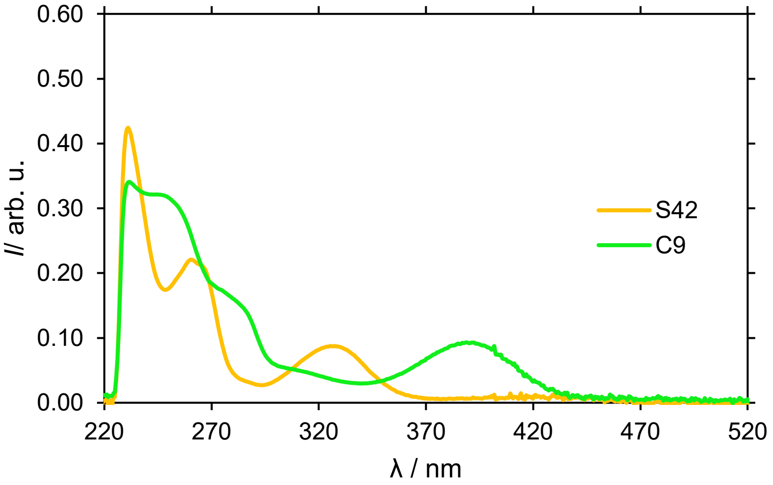

5.12 *N*-((1*R*,2*R*)-2-(((*E*)-3-Benzyl-5-(*tert*-butyl)-2-hydroxy-κ*O*-benzylidene)amino-κ*N*)-1,2-diphenylethyl)-1,1,1-trifluoromethanesulfonamide-κ*N* C10

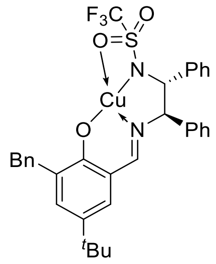

C10

Complex **C10** was synthesized according to **GP 5** using preligand **S46** (55.8 mg, 93.8 µmol, 1.00 eq.) and $Cu(acac)_2$ (26.6 mg, 93.8 µmol, 1.00 eq.). The reaction mixture was filtered over Celite®, and the solvent was removed under reduced pressure. Complex **C10** was dried under high vacuum and obtained as a green solid (61.6 mg, 93.8 µmol, >99%).

$C_{33}H_{31}CuF_3N_2O_3S$, MW: 656.22 g/mol. **MP:** 169-178 °C (decomposition). **$[\alpha]_D^{20}$ (*c* = 1.0 mg/mL, DCM)** = +120. **NMR:** paramagnetic species. **IR (DCM):** $\tilde{\nu}$ = 3061, 3028, 2960, 2927, 2866, 1621, 1583, 1540, 1524, 1495, 1450, 1392, 1364, 1320, 1270, 1212, 1185, 1149, 1086, 1072, 1029, 995, 944, 775, 764, 743, 699, 634, 617, 595, 567, 514, 489, 462, 445 cm^{-1}. **HRMS (ESI) *m/z*:** calculated for $C_{33}H_{32}CuF_3N_2O_3S$ [M+H]$^+$: 656.1376; measured 656.1378.

UV-Vis (DCM, *c* = 2 · 10^{-5} M):

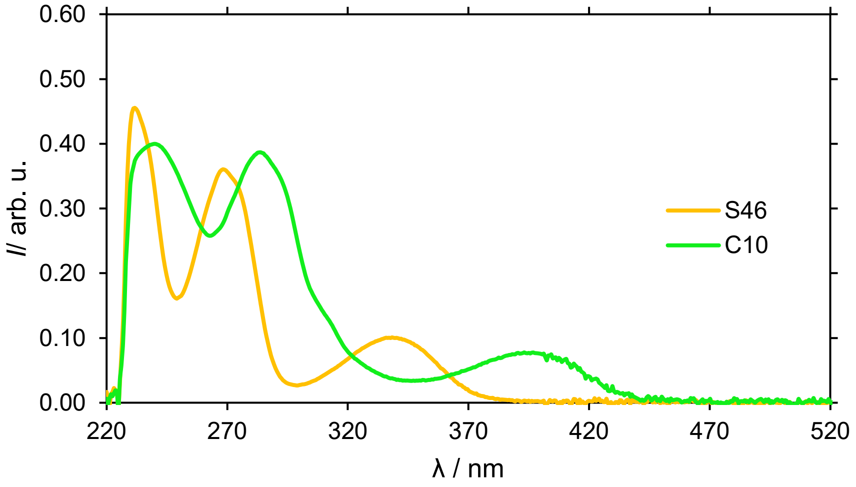

5.13 3-(5-(*tert*-Butyl)-3-((*E*)-(((1*R*,2*R*)-1,2-di(naphthalene-1-yl)-2-((trifluormethyl)sulfonamido-*κN*-)ethyl)imino-*κN*-)methyl)-2-hydroxy-*κO*-benzyl)-1-mesityl-1*H*-imidazol-3-ium copper(II) chloride C14

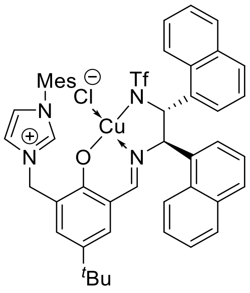

C14

Complex **C14** was synthesized according to **GP 5** using preligand **S36** (13.1 mg, 15.6 µmol, 1.00 eq.) and Cu(acac)$_2$ (4.1 mg, 15.6 µmol, 1.00 eq.). After workup, complex **C14** was obtained as a green solid (12.7 mg, 14.1 µmol, 90%).

$C_{47}H_{44}ClCuF_3N_4O_3S$, MW: 900.95 g/mol. **MP:** 212 °C (decomposition). **$[\alpha]_D^{20}$ (*c* = 1.0 mg/mL, DCM)** = +222. **NMR:** paramagnetic species. **IR (DCM):** $\tilde{\nu}$ = 2956, 1624, 1543, 1455, 1394, 1323, 1271, 1184, 1068, 1016, 938, 856, 781, 633, 593, 528, 503, 473, 449, 423 cm^{-1}. **HRMS (ESI) *m/z*:** calculated for $C_{47}H_{44}CuF_3N_4O_3S$ [M-Cl]$^+$: 864.2377; measured 864.2362.

UV-Vis (DCM, *c* = 2 · 10^{-5} M):

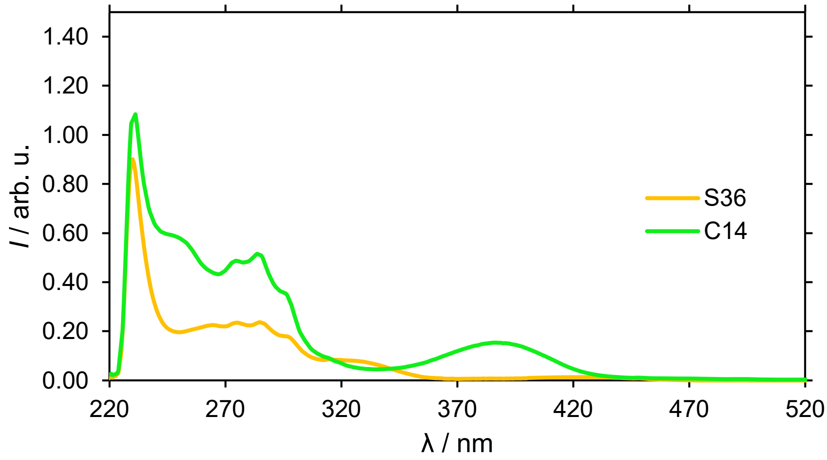

5.14 3-(5-(*tert*-Butyl)-3-((*E*)-(((1*R*,2*R*)-1,2-di(naphthalen-2-yl)-2-((trifluormethyl)sulfonamido-*κN*-)ethyl)imino-*κN*-)methyl)-2-hydroxy-*κO*-benzyl)-1-mesityl-1*H*-imidazol-3-ium copper(II) chloride C15

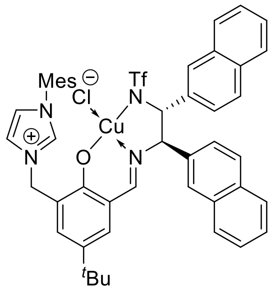

C15

Complex **C15** was synthesized according to **GP 5** using preligand **S37** (12.3 mg, 14.7 µmol, 1.00 eq.) and Cu(acac)$_2$ (3.8 mg, 14.7 µmol, 1.00 eq.). After workup, complex **C15** was obtained as a green solid (12.9 mg, 14.3 µmol, 98%).

$C_{47}H_{44}ClCuF_3N_4O_3S$, MW: 900.95 g/mol. **MP:** 215 °C (decomposition). **$[\alpha]_D^{20}$ (*c* = 1.0 mg/mL, DCM)** = +150. **NMR:** paramagnetic species. **IR ($CDCl_3$):** $\tilde{\nu}$ = 2924, 2853, 1734, 1625, 1543, 1508, 1455, 1434, 1391, 1365, 1332, 1274, 1186, 1120, 1089, 1068, 1001, 941, 858, 820, 799, 752, 600, 579, 478 cm^{-1}. **HRMS (ESI) *m/z*:** calculated for $C_{47}H_{44}CuF_3N_4O_3S$ [M-Cl]$^+$: 864.2377; measured 864.2350.

UV-Vis (DCM, *c* = 2 · 10^{-5} M):

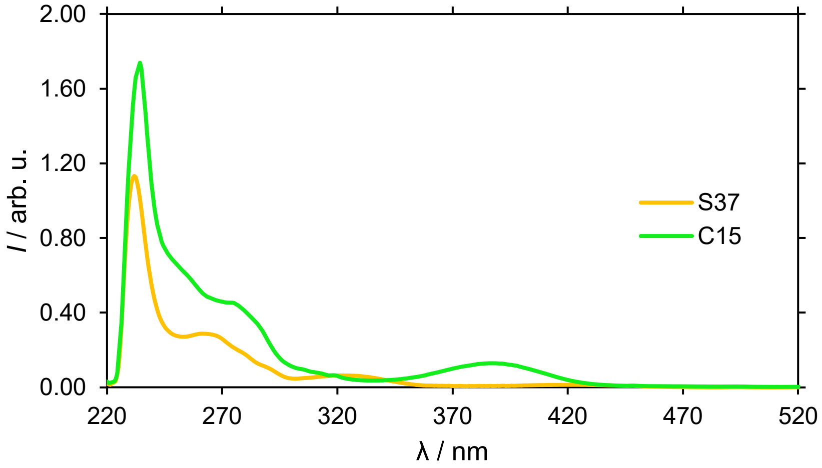

5.15 3-(3-((*E*)-(((1*R*,2*R*)-1,2-Bis(4-fluorophenyl)-2-((trifluormethyl)sulfonamido-*κN*-)ethyl)imino-*κN*-)methyl)-5-(*tert*-butyl)-2-hydroxy-*κO*-benzyl)-1-mesityl-1*H*-imidazol-3-ium copper(II) chloride C16

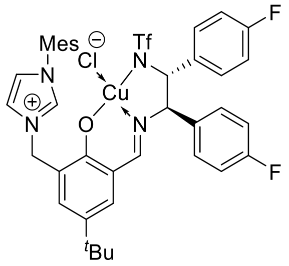

C16

Complex **C16** was synthesized according to **GP 5** using preligand **S38** (25.8 mg, 33.3 μmol, 1.00 eq.) and $Cu(acac)_2$ (8.7 mg, 33.3 μmol, 1.00 eq.). After workup, complex **C16** was obtained as a green solid (25.0 mg, 29.9 μmol, 90%).

$C_{39}H_{38}ClCuF_5N_4O_3S$, MW: 836.81 g/mol. **MP:** 226-230 °C (decomposition). **$[\alpha]_D^{20}$ (*c* = 1.0 mg/mL, DCM)** = +405. **NMR:** paramagnetic species. **IR (DCM):** $\tilde{\nu}$ = 2955, 2924, 2854, 1626, 1544, 1509, 1456, 1324, 1213, 1185, 1159, 1083, 998, 935, 840, 764, 621, 583, 523, 465 cm^{-1}. **HRMS (ESI) *m/z*:** calculated for $C_{39}H_{38}CuF_5N_4O_3S$ $[M-Cl]^+$: 800.1875; measured 800.1867.

UV-Vis (DCM, *c* = 2 · 10^{-5} M):

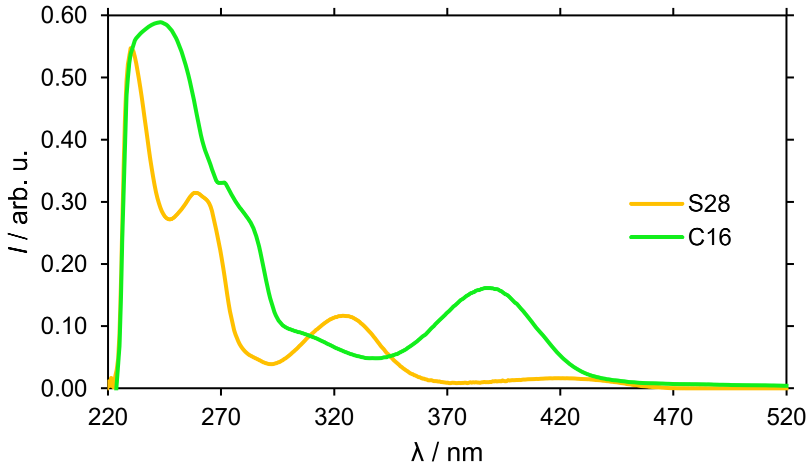

5.16 3-(3-((*E*)-(((1*R*,2*R*)-1,2-Bis(4-nitrophenyl)-2-((trifluoromethyl)sulfonamido-*κN*-)ethyl)imino-*κN*-)methyl)-5-(*tert*-butyl)-2-hydroxy-*κO*-benzyl)-1-mesityl-1*H*-imidazol-3-ium copper(II) chloride C17

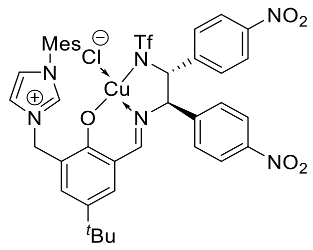

C17

Complex **C17** was synthesized according to **GP 5** using preligand **S39** (13.3 mg, 16.0 µmol, 1.00 eq.) and Cu(acac)$_2$ (4.2 mg, 16.0 µmol, 1.00 eq.). After workup, complex **C17** was obtained as a green solid (14.3 mg, 16.0 µmol, >99%).

$C_{39}H_{38}ClCuF_3N_6O_7S$, MW: 890.82 g/mol. **MP:** 185 °C (decomposition). **$[\alpha]_D^{20}$ (*c* = 1.0 mg/mL, DCM)** = +19. **NMR:** paramagnetic species. **IR (DCM):** $\tilde{\nu}$ = 3085, 2958, 2923, 2866, 1627, 1604, 1518, 1458, 1402, 1344, 1275, 1249, 1211, 1183, 1109, 1086, 1067, 1014, 997, 936, 859, 837, 798, 761, 736, 706, 680, 652, 613, 597, 517 cm^{-1}. **HRMS (ESI) *m/z*:** calculated for $C_{39}H_{38}CuF_3N_6O_7S$ [M-Cl]$^+$: 854.1765; measured 854.1759.

UV-Vis (DCM, *c* = 2 · 10^{-5} M):

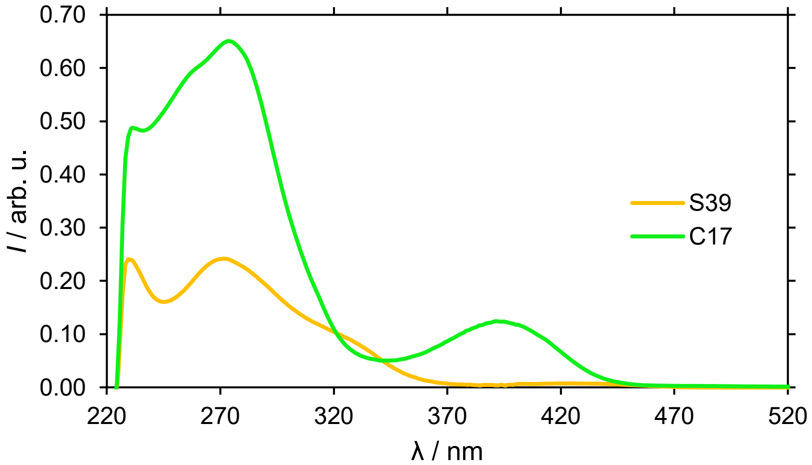

5.17 3-(5-(*tert*-Butyl)-2-hydroxy-*κO*-3-((*E*)-(((1*R*,2*R*)-2-((trifluoromethyl)sulfonamido-*κN*-)cyclohexyl)imino-*κN*-)methyl)benzyl)-1-mesityl-1*H*-imidazol-3-ium copper(II) chloride C18

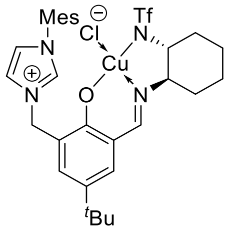

C18

Complex **C18** was synthesized according to **GP 5** using preligand **S40** (15.2 mg, 23.7 µmol, 1.00 eq.) and $Cu(acac)_2$ (6.2 mg, 23.7 µmol, 1.00 eq.). After workup, complex **C18** was obtained as a green solid (11.6 mg, 16.5 µmol, 70%).

$C_{28}H_{32}ClCuF_3N_4O_3S$, MW: 660.64 g/mol. **MP:** 197-200 °C (decomposition). **$[\alpha]_D^{20}$ (*c* = 1.0 mg/mL, DCM)** = +508. **NMR:** paramagnetic species. **IR (DCM):** $\tilde{\nu}$ = 3080, 2961, 2929, 2860, 1634, 1543, 1458, 1390, 1364, 1324, 1308, 1261, 1209, 1172, 1083, 1062, 1037, 1020, 962, 935, 906, 852, 796, 732, 700, 669, 654, 619, 578, 538, 480, 423 cm^{-1}. **HRMS (ESI) *m/z*:** calculated for $C_{28}H_{32}CuF_3N_4O_3S$ $[M-Cl]^+$: 666.1907; measured 666.1900.

UV-Vis (DCM, *c* = 2 · 10^{-5} M):

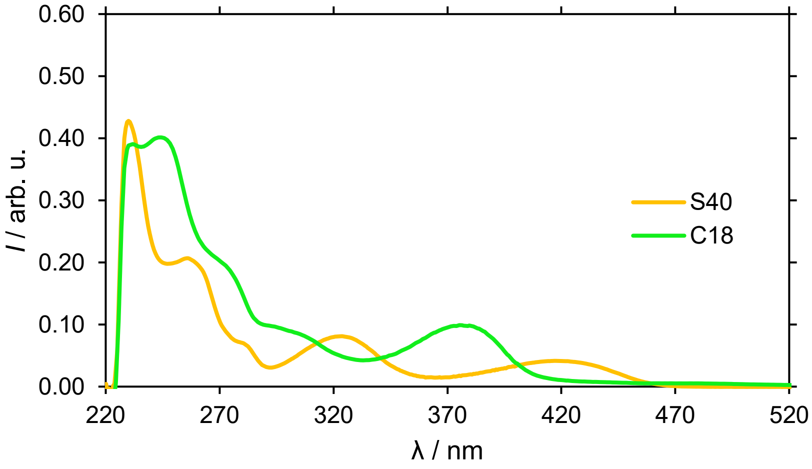

5.18 (*R*,*E*)-3-(5-(*tert*-Butyl)-2-hydroxy-*κO*-3-(((2'-((trifluoromethyl)sulfonamido-*κN*-)-[1,1'-binaphthalen]-2-yl)imino-*κN*-)methyl)benzyl)-1-mesityl-1*H*-imidazol-3-ium copper(II) chloride C19

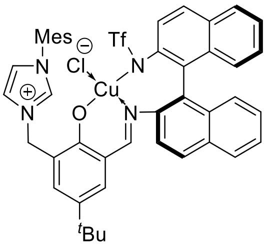

C19

Complex **C19** was synthesized according to **GP 5** using preligand **S41** (20.4 mg, 25.1 µmol, 1.00 eq.) and Cu(acac)$_2$ (6.6 mg, 25.1 µmol, 1.00 eq.). After workup, complex **C19** was obtained as a green solid (15.5 mg, 17.8 µmol, 71%).

$C_{45}H_{40}ClCuF_3N_4O_3S$, MW: 872.89 g/mol. **MP:** 210 °C (decomposition). **$[\alpha]_D^{20}$ (*c* = 1.0 mg/mL, DCM)** = +379. **NMR:** paramagnetic species. **IR (DCM):** $\tilde{\nu}$ = 3130, 3099, 3099, 3081, 3052, 2955, 2922, 2863, 1619, 1585, 1550, 1522, 1458, 1386, 1339, 1279, 1197, 1160, 1068, 1042, 1019, 933, 878, 852, 842, 822, 796, 796, 775, 748, 716, 623, 599, 551, 511 cm^{-1}. **HRMS (ESI) *m/z*:** calculated for $C_{45}H_{40}CuF_3N_4O_3S$ $[M\text{-}Cl]^+$: 836.2064; measured 836.2060.

UV-Vis (DCM, *c* = 2 · 10^{-5} M):

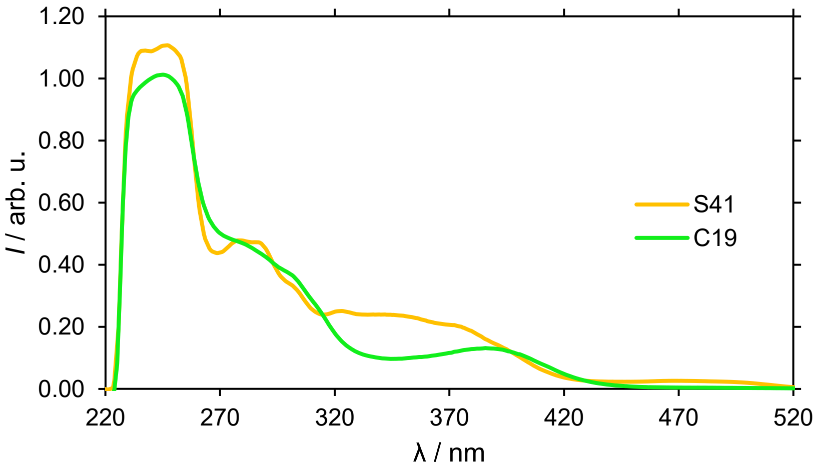

6 Synthesis of Asymmetric Alkylation Products

6.1 Methyl (*R*)-1-benzyl-2-oxocyclopentane-1-carboxylate 3aA

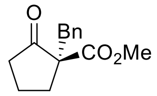

3aA

Compound **3aA** was synthesized according to **GP 9** using catalyst **C5** (1.21 mg, 1.5 µmol, 0.5 mol%), β-ketoester **1a** (37.5 µL, 0.30 mmol, 1.00 eq.), benzyl bromide **2A** (71.7 µL, 0.60 mmol, 2.00 eq.) and DIPEA (68.5 µL, 0.39 mmol, 1.30 eq.). The reaction was stirred for 20 h. The product **3aA** (66.6 mg, 0.29 mmol, 95%, 92% *ee*) was obtained as a colorless oil. The enantiomeric excess was determined by HPLC on chiral stationary phase. CHIRALPAK® *IA*, *n*-hexane/iPrOH = 97/3, 0.5 mL/min, λ = 215 nm, t_{R1} = 14.6 min, t_{R2} = 15.9 min. $[\alpha]_D^{20}$ = –41.1 (*c* = 1.0 mg/mL, DCM, 86% *ee*); Lit.[22] $[\alpha]_D^{20}$ = +58.0 (*c* = 0.4 mg/mL, DCM, 86% $ee_{(S)}$).

$C_{14}H_{16}O_3$, MW: 232.28 g/mol. **^{1}H-NMR (400 MHz, $CDCl_3$):** δ = 7.20-7.17 (*m*, 3 H, *o*- und *p*-Ar-*H*), 7.15-7.08 (*m*, 2 H, *m*-Ar-*H*), 3.72 (*s*, 3 H, OCH_3), 3.16 (*dd*, *J* = 22.9, 13.5 Hz, 2 H, CH_2-Ph), 2.48-2.29 (*m*, 2 H, C*H*), 2.13-1.97 (*m*, 3 H, C*H*), 1.69-1.51 (*m*, 1 H, C*H*) ppm.

The analytical data are consistent with the literature.[23]

6.2 Methyl (*R*)-1-(2-methylbenzyl)-2-oxocyclopentane-1-carboxylate 3aB

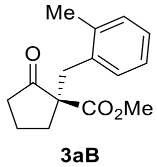

3aB

Compound **3aB** was synthesized according to **GP 9** using catalyst **C5** (1.21 mg, 1.5 µmol, 0.5 mol%), β-ketoester **1a** (37.5 µL, 0.30 mmol, 1.00 eq.), 1-(bromomethyl)-2-methylbenzene **2B** (111.7 mg, 0.60 mmol, 2.00 eq.) and DIPEA (68.5 µL, 0.39 mmol, 1.30 eq.). The reaction was stirred for 48 h. The product **3aB** (74.3 mg, 0.30 mmol, 99%, 92% *ee*) was obtained as a colorless oil. The enantiomeric excess was determined by HPLC on chiral stationary phase. CHIRALPAK® *IJ*, cyclohexane/*i*PrOH = 97/3, 0.5 mL/min, λ = 215 nm, t_{R1} = 15.7 min, t_{R2} = 18.8 min.

$C_{15}H_{18}O_3$, **MW:** 246.30 g/mol. $[\alpha]_D^{20}$(c = 1.0 mg/mL, $CHCl_3$, 92% *ee*) = –56. **^{1}H-NMR (300 MHz, $CDCl_3$):** δ = 7.19 – 6.96 (*m*, 4 H, Ar-*H*), 3.73 (*s*, 3 H, CO_2CH_3), 3.31 (*d*, J = 14.4 Hz, 1 H, Ar-CH_2), 3.20 (*d*, J = 14.4 Hz, 1 H, Ar-CH_2), 2.55–2.32 (*m*, 2 H, C*H*), 2.30 (*s*, 3 H, CH_3), 2.11–1.62 (*m*, 4 H, C*H*) ppm. **^{13}C-NMR (175 MHz, $CDCl_3$):** δ = 215.4, 171.9, 137.4, 135.4, 130.6, 130.0, 127.0, 126.2, 61.7, 52.9, 38.5, 35.2, 31.6, 20.3, 19.8 ppm. **IR ($CDCl_3$):** $\tilde{\nu}$ = 2953, 1753, 1729, 1494, 1450, 1228, 1144, 745 cm $^{-1}$. **HRMS (ESI) *m/z*:** calculated for $C_{15}H_{19}O_3$ $[M+H]^+$: 247.1329; measured 247.1323.

6.3 Methyl (*R*)-1-(3-methylbenzyl)-2-oxocyclopentane-1-carboxylate 3aC

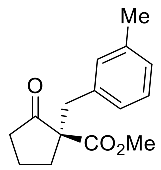

3aC

Compound **3aC** was synthesized according to **GP 9** using catalyst **C5** (1.2 mg, 1.5 µmol, 0.5 mol%), β-ketoester **1a** (37.5 µL, 0.30 mmol, 1.00 eq.), 1-(bromomethyl)-3-methylbenzene **2C** (81.5µL, 0.60 mmol, 2.00 eq.) and DIPEA (68.5 µL, 0.39 mmol, 1.30 eq.). The reaction was stirred for 48 h. The product **3aC** (74.3 mg, 0.30 mmol, >99%, 92% *ee*) was obtained as a colorless oil. The enantiomeric excess was determined by HPLC on chiral stationary phase. CHIRALPAK® *IJ*, cyclohexane/*i*PrOH = 97/3, 0.5 mL/min, λ = 215 nm, t_{R1} = 17.1 min, t_{R2} = 21.9 min. $[\alpha]_D^{20}$ (c = 1.0 mg/mL, $CHCl_3$, 92% *ee*) = –46.

$C_{15}H_{18}O_3$, **MW:** 246.30 g/mol. **^{1}H-NMR (300 MHz, $CDCl_3$):** δ = 7.14 (*t*, J = 7.5 Hz, 1 H, Ar-*H*), 7.03 (*d*, J = 7.5 Hz, 1 H, Ar-*H*), 6.91 (*d*, J = 7.8 Hz, 2 H, Ar-*H*), 3.73 (*s*, 3 H, CO_2CH_3), 3.17 (*d*, J = 13.7 Hz, 1 H, Ar-CH_2), 3.07 (*d*, J = 13.7 Hz, 1 H, Ar-CH_2), 2.49–2.32 (*m*, 2 H, C*H*), 2.30 (*s*, 3 H, CH_3), 2.14–1.78 (*m*, 3 H, C*H*), 1.72–1.54 (*m*, 1 H, C*H*) ppm.

The analytical data are consistent with the literature.[24]

6.4 Methyl (*R*)-1-(4-methylbenzyl)-2-oxocyclopentane-1-carboxylate 3aD

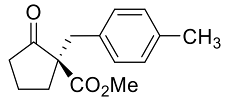

3aD

Compound **3aD** was synthesized according to **GP 9** using catalyst **C5** (1.21 mg, 1.5 µmol, 0.5 mol%), β-ketoester **1a** (37.5 µL, 0.30 mmol, 1.00 eq.), 4-methylbenzyl bromide (72.3 µL,

0.60 mmol, 2.00 eq.) **2D** and DIPEA (68.5 µL, 0.39 mmol, 1.30 eq.). The reaction was stirred for 20 h. The product **3aD** (73.1 mg, 0.29 mmol, 98%, 93% *ee*) was obtained as a colorless oil. The enantiomeric excess was determined by HPLC on chiral stationary phase. CHIRALPAK® *OD-H*, *n*-hexane/*i*PrOH = 97/3, 0.5 mL/min, λ = 215 nm, t_{R1} = 19.3 min, t_{R2} = 21.1 min.

$C_{15}H_{18}O_3$, MW: 246.31 g/mol. **$[\alpha]_D^{20}$** (c = 1.0 mg/mL, $CHCl_3$, 93% *ee*) = –64. **^{1}H-NMR (300 MHz, $CDCl_3$):** δ = 7.10-6.96 (*m*, 4 H, Ar-*H*), 3.72 (*s*, 3 H, OCH_3), 3.16 (*d*, *J* = 13.7 Hz, 1 H, Ar-CH_2), 3.07 (*d*, *J* = 13.7 Hz, 1 H, Ar-CH_2), 2.48-2.32 (*m*, 2 H, C*H*), 2.30 (*s*, 3 H, Ar-CH_3), 2.12-1.79 (*m*, 3 H, C*H*), 1.79-1.55 (*m*, 1 H, C*H*) ppm. **^{13}C-NMR (175 MHz, $CDCl_3$):** δ = 215.1, 171.6, 136.6, 133.5, 130.1, 129.2, 61.7, 52.8, 38.9, 38.6, 31.8, 21.2, 19.6 ppm. **IR ($CDCl_3$):** $\tilde{\nu}$ = 2954, 2923, 1752, 1728, 1515, 1435, 1405, 1265, 1235, 1143, 1116, 1017, 825 cm^{-1}. **HRMS (EI) *m/z*:** calculated for $C_{15}H_{18}O_3$ $[M]^+$: 246.1250; measured 246.1248.

6.5 Methyl (*R*)-1-(4-(*tert*-butyl)benzyl)-2-oxocyclopentane-1-carboxylate 3aE

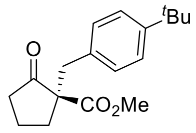

3aE

Compound **3aE** was synthesized according to **GP 9** using catalyst **C5** (1.21 mg, 1.5 µmol, 0.5 mol%), β-ketoester **1a** (37.5 µL, 0.30 mmol, 1.00 eq.), 1-(bromomethyl)-4-(*tert*-butyl)benzene **2E** (110.9 mg, 0.60 mmol, 2.00 eq.) and DIPEA (68.5 µL, 0.39 mmol, 1.30 eq.). The reaction was stirred for 20 h. The product **3aE** (82.1 mg, 0.29 mmol, 98%, 95% *ee*) was obtained as a colorless oil. The enantiomeric excess was determined by HPLC on chiral stationary phase. CHIRALPAK® *OD-H*, *n*-hexane/*i*PrOH = 97/3, 0.5 mL/min, λ = 215 nm, t_{R1} = 13.3 min, t_{R2} = 14.3 min.

$C_{18}H_{24}O_3$, MW: 288.38 g/mol. **$[\alpha]_D^{20}$**(c = 1.0 mg/mL, $CHCl_3$, 95% *ee*) = –44. **^{1}H-NMR (300 MHz, $CDCl_3$):** δ = 7.26 (*d*, *J* = 8.2 Hz, 2 H, Ar-*H*), 7.05 (*d*, *J* = 8.2 Hz, 2 H, Ar-*H*), 3.73 (*s*, 3 H, CH_3), 3.19 (*d*, *J* = 13.8 Hz, 1 H, Ar-CH_2), 3.06 (*d*, *J* = 13.8 Hz, 1 H, Ar-CH_2), 2.50–2.29 (*m*, 2 H, C*H*), 2.16–1.81 (*m*, 3 H, C*H*), 1.74-1.54 (*m*, 1 H, C*H*), 1.29 (*s*, 9 H, C(CH_3)$_3$) ppm. **^{13}C-NMR (175 MHz, $CDCl_3$):** δ = 215.1, 171.6, 149.8, 133.5, 129.9, 125.4, 61.7, 52.8, 38.7, 38.5, 34.5, 31.8, 31.5, 19.6 ppm. **IR ($CDCl_3$):** $\tilde{\nu}$ = 2960, 2869, 1753, 1728, 1514, 1435, 1407, 1365, 1313, 1268, 1224, 1183, 1162, 1144, 1124, 1110, 1039, 1020, 971, 924, 838, 583, 560, 509 cm^{-1}. **HRMS (ESI) *m/z*:** calculated for **$C_{18}H_{25}O_3$** $[M+H]^+$: 289.1798; measured 289.1794.

6.6 Methyl (*R*)-1-(3-methoxybenzyl)-2-oxocyclopentane-1-carboxylate 3aF

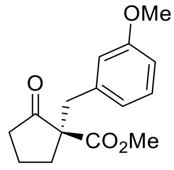

3aF

Compound **3aF** was synthesized according to **GP 9** using catalyst **C5** (1.2 mg, 1.5 µmol, 0.5 mol%), β-ketoester **1a** (37.5 µL, 0.30 mmol, 1.00 eq.), 1-(bromomethyl)-3-methoxybenzene **2F** (88.5 µL, 0.60 mmol, 2.00 eq.) and DIPEA (68.5 µL, 0.39 mmol, 1.30 eq.). The reaction was stirred for 20 h. The product **3aF** (79.2 mg, 0.30 mmol, >99%, 94% *ee*) was obtained as a colorless oil. The enantiomeric excess was determined by HPLC on chiral stationary phase. CHIRALPAK® *IJ*, cyclohexane/*i*PrOH = 97/3, 0.5 mL/min, λ = 215 nm, t_{R1} = 28.9 min, t_{R2} = 32.5 min. $[\alpha]_D^{20}$ (*c* = 1.0 mg/mL, $CHCl_3$, 94% *ee*) = –34.

$C_{15}H_{18}O_4$, MW: 262.30 g/mol. **^{1}H-NMR (300 MHz, $CDCl_3$):** δ = 7.17 (*t*, *J* = 7.9, 1 H, Ar-*H*), 6.80–6.73 (*m*, 1 H, Ar-*H*), 6.73–6.65 (*m*, 2 H, Ar-*H*), 3.77 (*s*, 3 H, C*H*$_3$), 3.73 (*s*, 3 H, C*H*$_3$), 3.19 (*d*, *J* = 13.7 Hz, 1 H, Ar-C*H*$_2$), 3.09 (*d*, *J* = 13.7 Hz, 1 H, Ar-C*H*$_2$), 2.48–2.31 (*m*, 2 H, C*H*), 2.15–1.80 (*m*, 3 H, C*H*), 1.71–1.55 (*m*, 1 H, C*H*) ppm.

The analytical data are consistent with the literature.[25]

6.7 Methyl (*R*)-1-(4-methoxybenzyl)-2-oxocyclopentane-1-carboxylate 3aG

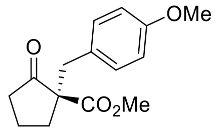

3aG

Compound **3aG** was synthesized according to **GP 9** using catalyst **C5** (1.2 mg, 1.5 µmol, 0.5 mol%), β-ketoester **1a** (37.5 µL, 0.30 mmol, 1.00 eq.), 1-(bromomethyl)-4-methoxybenzene **2G** (98.9 µL, 0.60 mmol, 2.00 eq.) and DIPEA (68.5 µL, 0.39 mmol, 1.30 eq.). The reaction was stirred for 20 h. The product **3aG** (75.2 mg, 0.28 mmol, 95%, 91% *ee*) was obtained as a colorless oil. The enantiomeric excess was determined by HPLC on chiral stationary phase. CHIRALPAK® *IJ*, *n*-hexane/*i*PrOH = 90/10, 0.5 mL/min, λ = 215 nm, t_{R1} = 38.2 min, t_{R2} = 49.6 min. $[\alpha]_D^{20}$ (*c* = 1.0 mg/mL, $CHCl_3$, 91% *ee*) = –50.

$C_{15}H_{18}O_4$, MW: 262.30 g/mol. **^{1}H-NMR (300 MHz, $CDCl_3$):** δ = 7.10–6.99 (*m*, 2 H, Ar-*H*), 6.85–6.74 (*m*, 2 H, Ar-*H*), 3.78 (*s*, 3 H, C*H*$_3$), 3.72 (*s*, 3 H, C*H*$_3$), 3.12 (*d*, *J* = 3.1, 1 H, Ar-C*H*$_2$), 3.09 (*d*, *J* = 3.1, 1 H, Ar-C*H*$_2$), 2.48–2.28 (*m*, 2 H, C*H*), 2.12–1.80 (*m*, 3 H, C*H*), 1.72–1.48 (*m*, 1 H, C*H*) ppm.

The analytical data are consistent with the literature.[24]

6.8 Methyl (*R*)-1-(4-chlorobenzyl)-2-oxocyclopentane-1-carboxylate 3aH

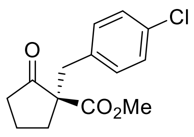

3aH

Compound **3aH** was synthesized according to **GP 9** using catalyst **C5** (1.2 mg, 1.5 µmol, 0.5 mol%), β-ketoester **1a** (37.5 µL, 0.30 mmol, 1.00 eq.), 1-(bromomethyl)-4-chlorobenzene **2H** (124.0 mg, 0.60 mmol, 2.00 eq.) and DIPEA (68.5 µL, 0.39 mmol, 1.30 eq.). The reaction was stirred for 20 h. The product **3aH** (76.0 mg, 0.28 mmol, 94%, 91% *ee*) was obtained as a colorless oil. The enantiomeric excess was determined by HPLC on chiral stationary phase. CHIRALPAK® *IC*, *n*-hexane/*i*PrOH = 97/3, 0.5 mL/min, λ = 215 nm, t_{R1} = 29.6 min, t_{R2} = 31.8 min. $[\alpha]_D^{20}$ (*c* = 1.0 mg/mL, $CHCl_3$, 91% *ee*) = –40.

$C_{14}H_{15}ClO_3$, MW: 266.72 g/mol. **^{1}H-NMR (300 MHz, $CDCl_3$):** δ = 7.25–7.20 (*m*, 2 H, Ar-*H*), 7.12–7.01 (*m*, 2 H, Ar-*H*), 3.72 (*s*, 3 H, C*H*$_3$), 3.18 (*d*, *J* = 13.8 Hz, 1 H, Ar-C*H*$_2$), 3.07 (*d*, *J* = 13.8 Hz, 1 H, Ar-C*H*$_2$), 2.50–2.31 (*m*, 2 H, C*H*), 2.14–1.96 (*m*, 1 H, C*H*), 1.94–1.80 (*m*, 2 H, C*H*), 1.74–1.56 (*m*, 1 H, C*H*) ppm.

The analytical data are consistent with the literature.[26]

6.9 Methyl (*R*)-2-oxo-1-(4-(trifluoromethyl)benzyl)cyclopentane-1-carboxylate 3aI

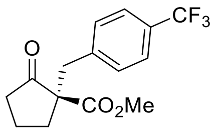

3aI

Compound **3aI** was synthesized according to **GP 9** using catalyst **C5** (1.21 mg, 1.5 µmol, 0.5 mol%), β-ketoester **1a** (37.5 µL, 0.30 mmol, 1.00 eq.), 1-(bromomethyl)-4-

(trifluoromethyl)benzene **2l** (144.3 mg, 0.60 mmol, 2.00 eq.) and DIPEA (68.5 µL, 0.39 mmol, 1.30 eq.). The reaction was stirred for 20 h. The product **3al** (82.5 mg, 0.29 mmol, 99%, 94% *ee*) was obtained as a colorless oil. The enantiomeric excess was determined by HPLC on chiral stationary phase. CHIRALPAK® *OD-H*, *n*-hexane/*i*PrOH = 99/1, 0.5 mL/min, λ = 215 nm, t_{R1} = 49.5 min, t_{R2} = 53.5 min.

$C_{15}H_{15}F_3O_3$, MW: 300.27 g/mol. **$[\alpha]_D^{20}$** (c = 1.0 mg/mL, $CHCl_3$, 94% *ee*) = –32. **^{1}H-NMR (300 MHz, $CDCl_3$):** δ = 7.25 (*d*, J = 3.0 Hz, 2 H, Ar-*H*), 7.26 (*d*, J = 3.0 Hz, 2 H, Ar-*H*), 3.73 (*s*, 3 H, CH_3), 3.28 (*d*, J = 13.8 Hz, 1 H, Ar-CH_2), 3.14 (*d*, J = 13.8 Hz, 1 H, Ar-CH_2), 2.50–2.35 (*m*, 2 H, C*H*), 2.14–2.06 (*m*, 1 H, C*H*), 1.99–1.82 (*m*, 2 H, C*H*), 1.73–1.61 (*m*, 1 H, C*H*) ppm. **^{19}F-NMR (376 MHz, $CDCl_3$):** δ = –62.53 (*s*, 3 F, CF_3) ppm. **^{13}C-NMR (175 MHz, $CDCl_3$):** δ = 214.4, 171.2, 140.9, 130.7, 129.7, 129.5, 129.3, 129.1, 126.6, 125.5, 125.5, 125.4, 125.4, 125.0, 123.5, 122.0, 61.5, 53.0, 38.8, 38.3, 31.9, 19.6 ppm. **IR ($CDCl_3$):** $\tilde{\nu}$ = 2958, 1754, 1729, 1619, 1450, 1436, 1420, 1326, 1269, 1238, 1164, 1116, 1068, 1041, 1020, 974, 958, 924, 854, 774, 523 cm^{-1}. **HRMS (ESI) *m/z*:** calculated for **$C_{15}H_{16}F_3O_3$** $[M+H]^+$: 301.1046; measured 301.1041.

6.10 Methyl (*R*)-2-oxo-1-((perfluorophenyl)methyl)cyclopentane-1-carboxylate 3aJ

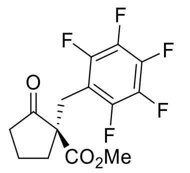

3aJ

Compound **3aJ** was synthesized according to **GP 9** using catalyst **C5** (1.21 mg, 1.5 µmol, 0.5 mol%), β-ketoester **1a** (37.5 µL, 0.30 mmol, 1.00 eq.), 1-(bromomethyl)-2,3,4,5,6-pentafluorobenzene **2J** (84.5µL, 0.60 mmol, 2.00 eq.) and DIPEA (68.5 µL, 0.39 mmol, 1.30 eq.). The reaction was stirred for 20 h. The product **3aJ** (94.3 mg, 0.29 mmol, 97%, 87% *ee*) was obtained as a white solid. The enantiomeric excess was determined by HPLC on chiral stationary phase. CHIRALPAK® *IC*, *n*-hexane/*i*PrOH = 97/3, 0.5 mL/min, λ = 208 nm, t_{R1} = 14.5 min, t_{R2} = 22.1 min.

$C_{14}H_{11}F_5O_3$, MW: 322.23 g/mol. **MP:** 79 °C. **$[\alpha]_D^{20}$** (c = 1.0 mg/mL, $CHCl_3$, 87% *ee*) = –10. **^{1}H-NMR (300 MHz, $CDCl_3$):** δ = 3.72 (*s*, 3 H, CH_3), 3.45 (*dt*, J = 14.5, 1.9 Hz, 1 H, Ar-CH_2), 2.98 (*dt*, J = 14.4, 1.9 Hz, 1 H, Ar-CH_2), 2.56–2.40 (*m*, 2 H, C*H*), 2.35–2.19 (*m*, 1 H, C*H*), 1.99–

1.77 (*m*, 3 H, C*H*). **^{19}F-NMR (376 MHz, $CDCl_3$):** δ = –140.27 (*dd*, *J* = 22.3, 8.0 Hz, 2 F, C_6F_5), –155.18 (*t*, *J* = 20.9 Hz, 1 F, C_6F_5), –161.97 – –162.18 (*m*, 2 F, C_6F_5) ppm. **^{13}C-NMR (175 MHz, $CDCl_3$):** δ = 212.4, 170.0, 146.40, 146.38, 146.36, 146.34, 146.32, 146.30, 146.27, 146.25, 145.00, 144.98, 144.96, 144.93, 144.92, 144.89, 144.87, 144.8, 141.3, 141.22, 141.19, 141.18, 141.15, 141.12, 141.10, 141.07, 141.0, 139.81, 139.78, 139.76, 139.74, 139.71, 139.68, 139.66, 139.63, 139.60, 138.38, 138.37, 138.36, 138.35, 138.31, 138.30, 138.28, 138.27, 138.26, 138.24, 138.21, 138.20, 138.18, 138.17, 137.0, 136.95, 136.93, 136.92, 136.89, 136.88, 136.86, 136.85, 136.83, 136.82, 136.79, 136.78, 136.76, 136.7, 110.69, 110.66, 110.58, 110.56, 110.5, 110.4, 60.6, 53.2, 37.3, 32.9, 26.8, 19.3 ppm. **IR ($CDCl_3$):** $\tilde{\nu}$ = 2959, 2896, 1725, 1657, 1521, 1501, 1447, 1407, 1317, 1301, 1262, 1212, 1109, 1044, 1022, 986, 934, 858, 612, 521, 458 cm^{-1}. **HRMS (ESI) *m/z*:** calculated for $C_{14}H_{12}F_5O_3$ $[M+H]^+$: 323.0701; measured 323.0693.

6.11 Methyl (*R*)-1-(4-cyanobenzyl)-2-oxocyclopentane-1-carboxylate 3aK

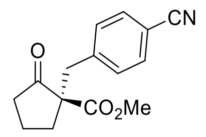

3aK

Compound **3aK** was synthesized according to **GP 9** using catalyst **C5** (1.2 mg, 1.5 µmol, 0.5 mol%), β-ketoester **1a** (37.5 µL, 0.30 mmol, 1.00 eq.), 4-(bromomethyl)benzonitrile **2K** (118.3 mg, 0.60 mmol, 2.00 eq.) and DIPEA (68.5 µL, 0.39 mmol, 1.30 eq.). The reaction was stirred for 20 h. The product **3aK** (77.1 mg, 0.29 mmol, 99%, 89% *ee*) was obtained as a yellow solid. The enantiomeric excess was determined by HPLC on chiral stationary phase. CHIRALPAK® *IJ*, cyclohexane/*i*PrOH = 90/10, 0.5 mL/min, λ = 215 nm, t_{R1} = 42.6 min, t_{R2} = 46.1 min. $[\alpha]_D^{20}$ (*c* = 1.0 mg/mL, $CHCl_3$, 89% *ee*) = –46.

$C_{15}H_{15}NO_3$, MW: 257.28 g/mol. **^{1}H-NMR (300 MHz, $CDCl_3$):** δ = 7.59–7.52 (*m*, 2 H, Ar-*H*), 7.30–7.24 (*m*, 2 H, Ar-*H*), 3.72 (*s*, 3 H, CH_3), 3.28 (*d*, *J* = 13.7 Hz, 1 H, Ar-CH_2), 3.12 (*d*, *J* = 13.7 Hz, 1 H, Ar-CH_2), 2.51–2.35 (*m*, 2 H, C*H*), 2.17–1.79 (*m*, 3 H, C*H*), 1.78–1.63 (*m*, 1 H, C*H*) ppm.

The analytical data are consistent with the literature.[26]

6.12 Methyl (*R*)-1-(4-nitrobenzyl)-2-oxocyclopentane-1-carboxylate 3aL

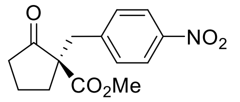

3aL

Compound **3aL** was synthesized according to **GP 9** using catalyst **C5** (1.2 mg, 1.5 µmol, 0.5 mol%), β-ketoester **1a** (37.5 µL, 0.30 mmol, 1.00 eq.), 4-Nitrobenzyl bromide (130.4 mg, 0.60 mmol, 2.00 eq.) **2L** and DIPEA (68.5 µL, 0.39 mmol, 1.30 eq.). The reaction was stirred for 20 h. The product **3aL** (83.5 mg, 0.30 mmol, 99%, 93% *ee*) was obtained as a colorless oil. The enantiomeric excess was determined by HPLC on chiral stationary phase. CHIRALPAK® *AS-H*, cyclohexane/*i*PrOH = 80/20, 0.2 mL/min, λ = 254 nm, t_{R1} = 36.9 min, t_{R2} = 40.9 min.

$C_{14}H_{15}NO_5$, MW: 277.28 g/mol. **$[\alpha]_D^{20}$** (c = 1.0 mg/mL, $CHCl_3$, 93% *ee*) = –48. **^{1}H-NMR (300 MHz, $CDCl_3$):** δ = 8.17-8.08 (*m*, 2 H, Ar-*H*), 7.36-7.28 (*m*, 2 H, Ar-*H*), 3.72 (*s*, 3 H, OCH_3), 3.33 (*d*, *J* = 13.7 Hz, 1 H, Ar-CH_2), 3.15 (*d*, *J* = 13.7 Hz, 1 H, Ar-CH_2), 2.55-2.37 (*m*, 2 H, C*H*), 2.16-1.80 (*m*, 3 H, C*H*), 1.79-1.64 (*m*, 1 H, C*H*) ppm. **^{13}C-NMR (175 MHz, $CDCl_3$):** δ = 213.9, 170.9, 147.2, 144.6, 131.2, 123.7, 61.4, 53.0, 38.8, 38.2, 32.1, 19.6 ppm. **IR ($CDCl_3$):** $\tilde{\nu}$ = 2956, 1751, 1725, 1604, 1518, 1493, 1435, 1404, 1346, 1267, 1224, 1182, 1162, 1144, 1111, 1016, 858, 748, 703 cm^{-1}. **HRMS (EI) *m/z*:** calculated for **$C_{14}H_{15}NO_5$** $[M]^+$: 277.0945; measured 277.0943.

6.13 Methyl (*R*)-1-(naphthalen-1-ylmethyl)-2-oxocyclopentane-1-carboxylate 3aM

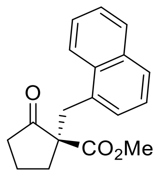

3aM

Compound **3aM** was synthesized according to **GP 9** using catalyst **C5** (1.2 mg, 1.5 µmol, 0.5 mol%), β-ketoester **1a** (37.5 µL, 0.30 mmol, 1.00 eq.), 1-(bromomethyl)naphthalene **2M** (133.4 mg, 0.60 mmol, 2.00 eq.) and DIPEA (68.5 µL, 0.39 mmol, 1.30 eq.). The reaction was stirred for 20 h. The product **3aM** (85.2 mg, 0.30 mmol, >99%, 91% *ee*) was obtained as a colorless oil. The enantiomeric excess was determined by HPLC on chiral stationary phase. CHIRALPAK® *IJ*, cyclohexane/*i*PrOH = 97/3, 0.5 mL/min, λ = 215 nm, t_{R1} = 25.8 min, t_{R2} = 38.6 min. $[\alpha]_D^{20}$ (c = 1.0 mg/mL, $CHCl_3$, 91% *ee*) = –78.

$C_{18}H_{18}O_3$, MW: 282.33 g/mol. **^{1}H-NMR (300 MHz, $CDCl_3$):** δ = 8.05 (*d*, *J* = 8.3 Hz, 1 H, Ar-*H*), 7.89–7.80 (*m*, 1 H, Ar-*H*), 7.74 (*d*, *J* = 8.2, 1 H, Ar-*H*), 7.58–7.42 (*m*, 2 H, Ar-*H*), 7.41–7.32 (*m*, 1 H, Ar-*H*), 7.30–7.26 (*m*, 1 H, Ar-*H*), 3.86 (*d*, *J* = 14.5 Hz, 1 H, Ar-C*H*$_2$), 3.73 (*s*, 3 H, C*H*$_3$), 3.59 (*d*, *J* = 14.5 Hz, 1 H, Ar-C*H*$_2$), 2.51–2.31 (*m*, 2 H, C*H*), 2.01–1.67 (*m*, 3 H, C*H*), 1.62–1.41 (*m*, 1 H, C*H*) ppm.

The analytical data are consistent with the literature.[24]

6.14 Methyl (*R*)-1-(naphthalen-2-ylmethyl)-2-oxocyclopentane-1-carboxylate 3aN

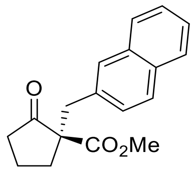

3aN

Compound **3aN** was synthesized according to **GP 9** using catalyst **C5** (1.21 mg, 1.5 µmol, 0.5 mol%), β-ketoester **1a** (37.5 µL, 0.30 mmol, 1.00 eq.), 2-(bromomethyl)naphthalene **2N** (133.4 mg, 0.60 mmol, 2.00 eq.) and DIPEA (68.5 µL, 0.39 mmol, 1.30 eq.). The reaction was stirred for 20 h. The product **3aN** (77.8 mg, 0.28 mmol, 93%, 90% *ee*) was obtained as a colorless oil. The enantiomeric excess was determined by HPLC on chiral stationary phase. CHIRALPAK® *OD-H*, *n*-hexane/*i*PrOH = 99/1, 0.5 mL/min, λ = 215 nm, t_{R1} = 26.1 min, t_{R2} = 34.6 min.

$C_{18}H_{18}O_3$, MW: 282.33 g/mol. $[\alpha]_D^{20}$ (*c* = 1.0 mg/mL, $CHCl_3$, 90% *ee*) = –68. **^{1}H-NMR (300 MHz, $CDCl_3$):** δ = 7.84–7.70 (*m*, 3 H, Ar-*H*), 7.59 (*br*, 1 H, Ar-*H*), 7.52–7.39 (*m*, 2 H, Ar-*H*), 7.27–7.22 (*m*, 1 H, Ar-*H*), 3.75 (*s*, 3 H, C*H*$_3$), 3.37 (*d*, *J* = 13.7 Hz, 1 H, Ar-C*H*$_2$), 3.30 (*d*, *J* = 13.7 Hz, 1 H, Ar-C*H*$_2$), 2.52–2.30 (*m*, 2 H, C*H*), 2.12–1.78 (*m*, 3 H, C*H*), 1.70–1.54 (*m*, 1 H, C*H*) ppm. **^{13}C-NMR (175 MHz, $CDCl_3$):** δ = 215.1, 171.6, 134.3, 133.5, 132.5, 129.1, 128.4, 128.1, 127.8, 127.7, 126.2, 125.9, 61.8, 52.9, 39.4, 38.5, 31.9, 19.7 ppm. **IR ($CDCl_3$):** $\tilde{\nu}$ = 3053, 3019, 2953, 2924, 2890, 2855, 1750, 1725, 1433, 1225, 1142, 824, 751, 478 cm^{-1}. **HRMS (ESI) *m/z*:** calculated for **$C_{18}H_{19}O_3$** $[M+H]^+$: 283.1329; measured 283.1332.

6.15 Methyl (*R*)-2-oxo-1-(thiophen-3-ylmethyl)cyclopentane-1-carboxylate 3aO

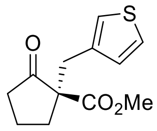

3aO

Compound **3aO** was synthesized according to **GP 9** using catalyst **C5** (1.2 mg, 1.5 µmol, 0.5 mol%), β-ketoester **1a** (37.5 µL, 0.30 mmol, 1.00 eq.), 2-(bromomethyl)thiophene **2O** (65.4 µL, 0.60 mmol, 2.00 eq.) and DIPEA (68.5 µL, 0.39 mmol, 1.30 eq.). The reaction was stirred for 20 h. The product **3aO** (69.6 mg, 0.29 mmol, 97%, 91% *ee*) was obtained as a colorless oil. The enantiomeric excess was determined by HPLC on chiral stationary phase. CHIRALPAK® *IA*, *n*-hexane/*i*PrOH = 97/3, 0.5 mL/min, λ = 215 nm, t_{R1} = 18.2 min, t_{R2} = 20.9 min.

$C_{12}H_{14}O_3S$, MW: 238.30 g/mol. **$[\alpha]_D^{20}$** (c = 1.0 mg/mL, $CHCl_3$, 91% *ee*) = –44. **^{1}H-NMR (300 MHz, $CDCl_3$):** δ = 7.23 (*dd*, J = 4.9, 3.0 Hz, 1 H, Ar-*H*), 7.02–6.94 (*m*, 1 H Ar-*H*), 6.86 (*dd*, J = 4.9, 1.3 Hz, 1 H, Ar-*H*), 3.72 (*s*, 3 H, C*H*$_3$), 3.24 (*d*, J = 14.2 Hz, 1 H, Ar-C*H*$_2$), 3.11 (*d*, J = 14.2 Hz, 1 H, Ar-C*H*$_2$), 2.50–2.31 (*m*, 2 H, C*H*), 2.17–1.83 (*m*, 3 H, C*H*), 1.76–1.55 (*m*, 1 H, C*H*) ppm. **^{13}C-NMR (175 MHz, $CDCl_3$):** δ = 215.1, 171.6, 136.7, 129.4, 125.7, 123.6, 61.2, 52.8, 38.5, 33.7, 32.1, 19.6 ppm. **IR ($CDCl_3$):** $\tilde{\nu}$ = 3105, 2953, 1750, 1726, 1435, 1405, 1317, 1232, 1143, 1097, 1009, 921, 835, 793, 692, 648 cm^{-1}. **HRMS (EI) *m/z*:** calculated for $C_{12}H_{14}O_3SNa$ [M+Na]$^+$: 261.0556; measured 261.0548.

6.16 Methyl (*R*)-2-oxo-1-(2-oxo-2-phenylethyl)cyclopentane-1-carboxylate 3aP

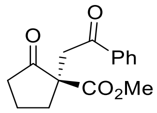

3aP

Compound **3aP** was synthesized according to **GP 9** using catalyst **C5** (1.21 mg, 1.5 µmol, 0.5 mol%), β-ketoester **1a** (37.5 µL, 0.30 mmol, 1.00 eq.), 2-bromo-1-phenylethan-1-one **2P** (119.5 mg, 0.60 mmol, 2.00 eq.) and DIPEA (68.5 µL, 0.39 mmol, 1.30 eq.). The reaction was stirred for 20 h at –20 °C. The product **3aP** (74.1 mg, 0.28 mmol, 95%, 87% *ee*) was obtained as a colorless oil. The enantiomeric excess was determined by HPLC on chiral stationary phase. CHIRALPAK® *IJ*, *n*-hexane/*i*PrOH = 90/10, 0.5 mL/min, λ = 215 nm, t_{R1} = 27.4 min, t_{R2} = 35.3 min.

$C_{15}H_{16}O_4$, MW: 260.28 g/mol. **$[\alpha]_D^{20}$** (*c* = 1.0 mg/mL, $CHCl_3$, 87% *ee*) = –32. **^{1}H-NMR (300 MHz, $CDCl_3$):** δ = 7.99–7.90 (*m*, 2 H, Ar-*H*), 7.63–7.52 (*m*, 1 H, Ar-*H*), 7.52–7.40 (*m*, 2 H, Ar-*H*), 3.86 (*d*, *J* = 18.5 Hz, 1 H, Ar-C*H*$_2$), 3.72 (*s*, 3 H, C*H*$_3$), 3.49 (*d*, *J* = 18.6 Hz, 1 H, Ar-C*H*$_2$), 2.74–2.44 (*m*, 3 H, C*H*), 2.34–2.00 (*m*, 3 H, C*H*) ppm. **^{13}C-NMR (175 MHz, $CDCl_3$):** δ = 215.1, 196.8, 171.3, 136.4, 133.7, 128.8, 128.2, 57.6, 53.0, 43.7, 37.9, 33.5, 20.0 ppm. **IR ($CDCl_3$):** $\tilde{\nu}$ = 2955, 2915, 1751, 1723, 1685, 1597, 1580, 1449, 1434, 1402, 1354, 1323, 1263, 1221, 1106, 1078, 1044, 1029, 1000, 923, 880, 852, 799, 754, 691, 616, 596, 566, 509, 486 cm^{-1}. **HRMS (ESI) *m/z*:** calculated for **$C_{15}H_{17}O_4$** $[M+H]^+$: 261.1121; measured 261.1116.

6.17 Methyl (*R*)-1-allyl-2-oxocyclopentane-1-carboxylate 3aQ

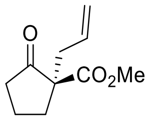

3aQ

Compound **3aQ** was synthesized according to **GP 9** using catalyst **C5** (1.21 mg, 1.5 µmol, 0.5 mol%), β-ketoester **1a** (37.5 µL, 0.30 mmol, 1.00 eq.), allyl bromide **2Q** (52.2 µL, 0.60 mol, 2.00 eq.) and DIPEA (68.5 µL, 0.39 mmol, 1.30 eq.). The reaction mixture was stirred for 48 h. Column chromatography on silica gel (*n*-pentane/Et_2O, 2:1) afforded product **3aQ** (51.6 mg, 0.28 mmol, 94%, 96% *ee*) as a colorless oil. The enantiomeric excess was determined by GC using the *Bondex un ß* column with H_2 carrier gas (40 °C for 1 min then with 0.5 °C/min to 80 °C), t_{R1} = 85.06 min, t_{R2} = 87.54 min. $[\alpha]_D^{20}$ = –28 (*c* = 1.0 mg/mL, $CHCl_3$, 96% *ee*); Lit.[27] $[\alpha]_D^{25}$ = –51.9 (*c* = 1.0 mg/mL, $CHCl_3$, 97% $ee_{(R)}$).

$C_{10}H_{14}O_3$, MW: 182.20 g/mol. **^{1}H-NMR (300 MHz, $CDCl_3$):** δ = 5.75-5.55 (*m*, 1 H, C=C*H*), 5.16-5.02 (*m*, 2 H, C=C*H*$_2$), 3.67 (*s*, 3 H, CO_2C*H*$_3$), 2.70-2.58 (*m*, 1 H, C*H*), 2.51-2.12 (*m*, 4 H, C*H*), 1.99-1.77 (*m*, 3 H, C*H*) ppm.

The analytical data are consistent with the literature.[27]

6.18 Methyl (*R*,*Z*)-2-oxo-1-(pent-2-en-1-yl)cyclopentane-1-carboxylat 3aR

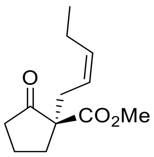

3aR

Compound **3aR** was synthesized according to **GP 9** using catalyst **C5** (1.2 mg, 1.5 μmol, 0.5 mol%), β-ketoester **1a** (37.5 μL, 0.30 mmol, 1.00 eq.), (*Z*)-1-bromopent-2-ene **2R** (71.3 μL, 0.60 mmol, 2.00 eq.) and DIPEA (68.5 μL, 0.39 mmol, 1.30 eq.). The reaction was stirred for 20 h. The product **3aR** (44.9 mg, 0.21 mmol, 71%, 91% *ee*) was obtained as a colorless oil. The enantiomeric excess was determined by HPLC on chiral stationary phase. CHIRALPAK® *OD-H*, *n*-hexane/*i*PrOH = 97/3, 0.5 mL/min, λ = 202 nm, t_{R1} = 11.3 min, t_{R2} = 13.0 min. $[\alpha]_D^{20}$ (*c* = 1.0 mg/mL, $CHCl_3$, 91% *ee*) = –6.

$C_{12}H_{18}O_3$, MW: 210.27 g/mol. **^{1}H-NMR (300 MHz, $CDCl_3$):** δ = 5.62–5.44 (*m*, 1 H, C=C*H*), 5.27–5.12 (*m*, 1 H, C=C*H*), 3.70 (*s*, 3 H, CO_2CH_3), 2.74–2.60 (*m*, 1 H, C*H*), 2.57–2.33 (*m*, 3 H, C*H*), 2.33–2.15 (*m*, 1 H, C*H*), 2.13–1.82 (*m*, 5 H, C*H*), 0.95 (*t*, *J* = 7.5 Hz, 3 H, CH_3) ppm.

The analytical data are consistent with the literature.[28]

6.19 Methyl (*R*,*E*)-2-oxo-1-(pent-2-en-1-yl)cyclopentane-1-carboxylate 3aS

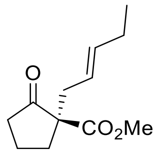

3aS

Compound **3aS** was synthesized according to **GP 9** using catalyst **C5** (1.2 mg, 1.5 μmol, 0.5 mol%), β-ketoester **1a** (37.5 μL, 0.30 mmol, 1.00 eq.), (*E*)-1-bromopent-2-ene **2S** (71.3 μL, 0.60 mmol, 2.00 eq.) and DIPEA (68.5 μL, 0.39 mmol, 1.30 eq.). The reaction was stirred for 20 h. The product **3aS** (55.2 mg, 0.26 mmol, 88%, 92% *ee*) was obtained as a colorless oil. The enantiomeric excess was determined by HPLC on chiral stationary phase. CHIRALPAK® *OD-H*, *n*-hexane/*i*PrOH = 97/3, 0.5 mL/min, λ = 215 nm, t_{R1} = 11.1 min, t_{R2} = 12.2 min.

$C_{12}H_{18}O_3$, MW: 210.27 g/mol. **$[\alpha]_D^{20}$** (*c* = 1.0 mg/mL, $CHCl_3$, 92% *ee*) = –112. **^{1}H-NMR (300 MHz, $CDCl_3$):** δ = 5.55 (*dtt*, *J* = 15.2, 6.4, 1.2 Hz, 1 H, C=C*H*), 5.26 (*dtt*, *J* = 15.0, 7.3, 1.5 Hz, 1 H, C=C*H*), 3.70 (*s*, 3 H, CH_3), 2.67–2.53 (*m*, 1 H, C*H*), 2.53–2.12 (*m*, 4 H, C*H*), 2.11–1.79 (*m*, 5 H, C*H*), 0.95 (*t*, *J* = 7.5 Hz, 3 H, CH_3) ppm. **^{13}C-NMR (175 MHz, $CDCl_3$):** δ = 215.0, 171.6, 137.3, 123.1, 60.5, 52.7, 38.4, 36.9, 32.0, 25.8, 19.6, 13.9 ppm. **IR ($CDCl_3$):** $\tilde{\nu}$ = 2961, 1752, 1728, 1434, 1406, 1317, 1276, 1224, 1162, 1078, 1016, 972, 924, 841 cm^{-1}. **HRMS (ESI) *m/z*:** calculated for $C_{12}H_{18}O_4$ $[M+H]^+$: 211.1329; measured 211.1324.

6.20 Methyl (*R*)-1-(but-2-yn-1-yl)-2-oxocyclopentane-1-carboxylate 3aT

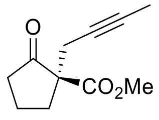

3aT

Compound **3aT** was synthesized according to **GP 9** using catalyst **C5** (1.2 mg, 1.5 µmol, 0.5 mol%), β-ketoester **1a** (37.5 µL, 0.30 mmol, 1.00 eq.), 1-bromobut-2-yne **2T** (52.8 µL, 0.60 mmol, 2.00 eq.) and DIPEA (68.5 µL, 0.39 mmol, 1.30 eq.). The reaction was stirred for 20 h. The product **3aT** (56.6 mg, 0.29 mmol, 97%, 90% *ee*) was obtained as a colorless oil. The enantiomeric excess was determined by HPLC on chiral stationary phase. CHIRALPAK® *OD-H*, *n*-hexane/*i*PrOH = 97/3, 0.5 mL/min, λ = 204 nm, t_{R1} = 16.1 min, t_{R2} = 20.3 min. $[\alpha]_D^{20}$ (*c* = 1.0 mg/mL, $CHCl_3$, 90% *ee*) = −50.

$C_{11}H_{14}O_3$, MW: 194.23 g/mol. **^{1}H-NMR (300 MHz, $CDCl_3$):** δ = 3.71 (*s*, 3 H, CO_2CH_3), 2.66 (*q*, *J* = 2.5 Hz, 2 H, CCH_2), 2.56–2.39 (*m*, 2 H, C*H*), 2.38–2.19 (*m*, 2 H, C*H*), 2.14–1.94 (*m*, 2 H, C*H*), 1.75 (*t*, *J* = 2.6, 3 H, CCH_3) ppm.

The analytical data are consistent with the literature.[28]

6.21 Methyl (*S*)-1-methyl-2-oxocyclopentane-1-carboxylate 3aU

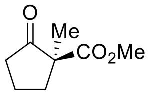

3aU

Compound **3aU** was synthesized according to **GP 9** using catalyst **C5** (1.21 mg, 1.5 µmol, 0.5 mol%), β-ketoester **1a** (37.5 µL, 0.30 mmol, 1.00 eq.), methyl iodide **2U** (75.2 µL, 1.20 mmol, 4.00 eq.), DIPEA (68.5 µL, 0.39 mmol, 1.30 eq.). The reaction was stirred for 48 h. Column chromatography on silica gel (*n*-pentane/Et_2O, 2:1) afforded product **3aU** (46.9 mg, 0.30 mmol, >99%, 93% *ee*) as a colorless oil. The enantiomeric excess was determined by GC using the *ChiralDex-B-DM* column with H_2 carrier gas (40 °C for 1 min then with 2.5 °C/min to 200 °C), t_{R1} = 21.33 min, t_{R2} = 22.49 min. $[\alpha]_D^{20}$ = +10 (*c* = 1.0 mg/mL, $CHCl_3$, 93% *ee*); Lit.[29] $[\alpha]_D^{20}$ = +11.1 (*c* = 1.0 mg/mL, $CHCl_3$, >99% $ee_{(S)}$).

$C_8H_{12}O_3$, MW: 156.18 g/mol. **^{1}H-NMR (300 MHz, $CDCl_3$):** δ = 3.70 (*s*, 3 H, CO_2CH_3), 2.59-2.23 (*m*, 3 H, C*H*), 2.13-1.78 (*m*, 3 H, C*H*), 1.31 (*s*, 3 H, CH_3) ppm.

The analytical data are consistent with the literature.[8]

6.22 Methyl (*S*)-1-ethyl-2-oxocyclopentane-1-carboxylate 3aV

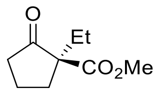

3aV

Compound **3aV** was synthesized according to **GP 10** using catalyst **C5** (6.0 mg, 7.5 μmol, 2.5 mol%), β-ketoester **1a** (37.5 μL, 0.30 mmol, 1.00 eq.), ethyl trifluoromethanesulfonate **2V** (58.5 μL, 0.45 mmol, 1.50 eq.) and DIPEA (78.6 μL, 0.15 mmol, 1.50 eq.). The product **3aV** (37.8 mg, 0.22 mmol, 74%, 94% *ee*) was isolated after column chromatography on silica gel (*n*-pentane/Et_2O, 2:1) as a colorless oil. The enantiomeric excess was determined by chiral GC analysis using ChiralDex-B-DM column with H_2 carrier gas 40 °C for 1 min then with 0.5 °C/min to 200 °C, t_{R1} = 67.33 min, t_{R2} = 69.05 min. $[\alpha]_D^{20}$ (*c* = 1.0 mg/mL, $CHCl_3$, 89% *ee*) = 10.

$C_9H_{14}O_3$, MW: 170.20 g/mol. **^{1}H-NMR (300 MHz, $CDCl_3$):** δ = 3.70 (*s*, 3 H, CH_3), 2.66–2.11 (*m*, 3 H CH_2CH_3, C*H*), 2.10–1.77 (*m*, 4 H, C*H*), 1.75–1.39 (*m*, 1 H, C*H*), 0.95–0.81 (*m*, 3 H, CH_2CH_3) ppm.

The analytical data are consistent with the literature.[30]

6.23 Methyl (*S*)-2-oxo-1-pentylcyclopentane-1-carboxylate 3aW

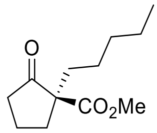

3aW

Compound **3aW** was synthesized according to **GP 10** using catalyst **C5** (6.0 mg, 7.5 μmol, 2.5 mol%), β-ketoester **1a** (37.5 μL, 0.30 mmol, 1.00 eq.), pentyl trifluoromethanesulfonate **2W** (99.1 mg, 0.45 mmol, 1.50 eq.) and DIPEA (78.6 μL, 0.15 mmol, 1.50 eq.). Column chromatography on silica gel (DCM//PE, 5:1) afforded product **3aW** (46.4 mg, 0.22 mmol, 73%, 90% *ee*) as a colorless oil. The enantiomeric excess was determined by chiral GC analysis using ChiralDex-B-DM column with H_2 carrier gas 40 °C for 1 min then with 1°C/min to 200 °C, t_{R1} = 75.06 min, t_{R2} = 76.03 min.

$C_{12}H_{20}O_3$, MW: 212.14 g/mol. **$[\alpha]_D^{20}$** (c = 1.0 mg/mL, $CHCl_3$, 90% *ee*) = –30. **^{1}H-NMR (300 MHz, $CDCl_3$):** δ = 3.73–3.66 (*m*, 3 H, CO_2CH_3), 2.61–2.16 (*m*, 3 H, C*H*), 2.09–1.81 (*m*, 4 H, C*H*), 1.62–1.46 (*m*, 1 H, C*H*), 1.36–1.24 (*m*, 6 H, C*H*), 0.92–0.81 (*m*, 3 H, CH_3) ppm. **^{13}C-NMR (175 MHz, $CDCl_3$):** δ = 215.2, 171.7, 60.8, 52.6, 38.2, 34.1, 32.8, 32.1, 24.7, 22.5, 19.8, 14.1 ppm. **IR ($CDCl_3$):** $\tilde{\nu}$ = 2955, 2929, 2860, 1752, 1725, 1458, 1435, 1407, 1379, 1318, 1257, 1231, 1202, 1156, 1110, 1005, 917, 843 cm^{-1}. **HRMS (ESI) *m/z*:** calculated for $C_{12}H_{21}O_3$ $[M+H]^+$: 213.1485; measured 213.1480.

6.24 Methyl (*S*)-1-hexyl-2-oxocyclopentane-1-carboxylate 3aX

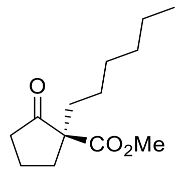

3aX

Compound **3aX** was synthesized according to **GP 10** using catalyst **C5** (6.0 mg, 7.5 µmol, 2.5 mol%), β-ketoester **1a** (37.5 µL, 0.30 mmol, 1.00 eq.), hexyl trifluoromethanesulfonate **2X** (105.4 mg, 0.45 mmol, 1.50 eq.) and DIPEA (78.6 µL, 0.15 mmol, 1.50 eq.). The product **3aX** (65.1 mg, 0.29 mmol, 96%, 91% *ee*) was obtained as a colorless oil. The enantiomeric excess was determined by chiral GC analysis using Bondex un Beta column with H_2 carrier gas 40 °C for 1 min then with 1 °C/min to 200 °C, t_{R1} = 86.38 min, t_{R2} = 87.25 min. $[\alpha]_D^{20}$ = –18 (c = 1.0 mg/mL, DCM, 91% *ee*); Lit.[31] $[\alpha]_D^{25}$ = +21.1 (c = 0.54 mg/mL, DCM, 85% $ee_{(R)}$).

$C_{13}H_{22}O_3$, MW: 226.31 g/mol. **^{1}H-NMR (300 MHz, $CDCl_3$):** δ = 3.70 (*s*, 3 H, CO_2CH_3), 2.68–2.16 (*m*, 3 H, C*H*), 2.08–1.80 (*m*, 3 H, C*H*), 1.76–1.64 (*m*, 1 H, C*H*), 1.63–1.47 (*m*, 1 H, C*H*), 1.46–1.07 (*m*, 8 H, C*H*), 0.92–0.81 (*m*, 3 H, CH_3).

The analytical data are consistent with the literature.[31]

6.25 Methyl (*R*)-2-oxo-1-phenethylcyclopentane-1-carboxylate 3aY

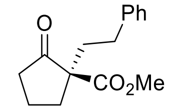

3aY

Compound **3aY** was synthesized according to **GP 10** using catalyst **C5** (6.0 mg, 7.5 µmol, 2.5 mol%), β-ketoester **1a** (37.5 µL, 0.30 mmol, 1.00 eq.), phenethyl trifluoromethanesulfonate

2Y (114.4 mg, 0.45 mmol, 1.50 eq.) and DIPEA (78.6 µL, 0.15 mmol, 1.50 eq.). Column chromatography on silica gel (DCM//PE, 5:1) afforded product **3aY** (65.7 mg, 0.27 mmol, 89%, 94% *ee*) as a colorless oil. The enantiomeric excess was determined by chiral GC analysis using Bondex un beta column with H_2 carrier gas 40 °C for 1 min then with 1 °C/min to 100 °C for 3 min then with 1.0 °C min to 130 °C for 3 min then with 0.5 °C/min to 200 °C, t_{R1} = 93.73 min, t_{R2} = 94.70 min.

$C_{15}H_{18}O_3$, MW: 246.30 g/mol. $[\alpha]_D^{20}$ (*c* = 1.0 mg/mL, $CHCl_3$, 94% *ee*) = –24. **^{1}H-NMR (300 MHz, $CDCl_3$):** δ = 7.33–7.23 (*m*, 2 H, Ar-*H*), 7.23–7.11 (*m*, 3 H, Ar-*H*), 3.71 (*s*, 3 H, C*H*$_3$), 2.76–2.36 (*m*, 4 H, C*H*), 2.36–2.15 (*m*, 2 H, C*H*), 2.14–1.71 (*m*, 4 H, C*H*) ppm. **^{13}C-NMR (175 MHz, $CDCl_3$):** δ = 213.7, 170.4, 140.4, 127.5, 127.5, 125.2, 59.6, 51.7, 37.1, 35.0, 32.1, 30.4, 18.8 ppm. **IR ($CDCl_3$):** $\tilde{\nu}$ = 3062, 3026, 2954, 2927, 2863, 1748, 1719, 1603, 1497, 1454, 1434, 1405, 1318, 1255, 1231, 1198, 1143, 1113, 1075, 1028, 953, 916, 841, 814, 791, 750, 700, 598, 495 cm^{-1}. **HRMS (EI) *m/z*:** calculated for $C_{15}H_{18}O_3$ $[M]^+$: 245.1183; measured 245.1185.

6.26 Methyl (*R*)-1-(3-fluoropropyl)-2-oxocyclopentane-1-carboxylate 3aZ

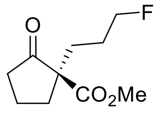

3aZ

Compound **3aZ** was synthesized according to **GP 10** using catalyst **C5** (6.0 mg, 7.5 µmol, 2.5 mol%), β-ketoester **1a** (37.5 µL, 0.30 mmol, 1.00 eq.), 3-fluoropropyl trifluoromethanesulfonate **2Z** (94.6 mg, 0.45 mmol, 1.50 eq.) and DIPEA (78.6 µL, 0.15 mmol, 1.50 eq.). Column chromatography on silica gel (DCM//PE, 5:1) afforded product **3aZ** (41.8 mg, 0.20 mmol, 69%, 85% *ee*) as a colorless oil. The enantiomeric excess was determined by chiral GC analysis using Beta-DexTM-225 column with H_2 carrier gas 40 °C for 1 min then with 2.5 °C/min to 200 °C, t_{R1} = 144.18 min, t_{R2} = 145.39 min.

$C_{10}H_{15}FO_3$, MW: 202.23 g/mol. $[\alpha]_D^{20}$ (*c* = 1.0 mg/mL, $CHCl_3$, 85% *ee*) = –14. **^{1}H-NMR (300 MHz, $CDCl_3$):** δ = 4.66–4.41 (*m*, 1 H, C*H*$_2$F), 4.41–4.26 (*m*, 1 H, C*H*$_2$F), 3.71 (*s*, 3 H, C*H*$_3$), 2.62–2.36 (*m*, 2 H, C*H*), 2.36–2.18 (*m*, 1 H, C*H*), 2.14–1.60 (*m*, 7 H, C*H*) ppm. **^{13}C-NMR (175 MHz, $CDCl_3$):** δ = 214.7, 171.6, 84.4, 83.4, 60.0, 52.8, 38.0, 33.2, 29.8, 29.8, 26.2, 26.1, 19.7 ppm. **IR ($CDCl_3$):** $\tilde{\nu}$ = 2958, 1750, 1725, 1451, 1435, 1405, 1321, 1264, 1234, 1220, 1205, 1155, 1045, 997, 971, 920, 880, 839, 617, 534, 454, 410 cm^{-1}. **HRMS (ESI) *m/z*:** calculated for **$C_{10}H_{16}FO_3$** $[M+H]^+$: 203.1078; measured 203.1072.

6.27 Methyl (*R*)-1-(cyclohexylmethyl)-2-oxocyclopentane-1-carboxylate 3aAA

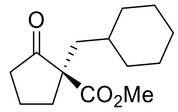

3aAA

Compound **3aAA** was synthesized according to **GP 10** using catalyst **C5** (6.0 mg, 7.5 µmol, 2.5 mol%), β-ketoester **1a** (37.5 µL, 0.30 mmol, 1.00 eq.), cyclohexylmethyl trifluoromethanesulfonate **2AA** (121.8 mg, 0.45 mmol, 1.50 eq.) and DIPEA (78.6 µL, 0.15 mmol, 1.50 eq.). The product **3aAA** (31.1 mg, 0.13 mmol, 43%, 75% *ee*) was obtained as a colorless oil. The enantiomeric excess was determined by HPLC on chiral stationary phase. CHIRALPAK® *IA*, *n*-hexane/iPrOH = 97/3, 0.5 mL/min, λ = 215 nm, t_{R1} = 10.9 min, t_{R2} = 11.7 min.

$C_{14}H_{22}O_3$, MW: 238.32 g/mol. **MP:** 50 °C $[\alpha]_D^{20}$ (*c* = 1.0 mg/mL, $CHCl_3$, 75% *ee*) = –18. **^{1}H-NMR (300 MHz, $CDCl_3$):** δ = 3.70 (*s,* 3 H, C*H*$_3$), 2.69–2.57 (*m*, 1 H, C*H*), 2.47–2.18 (*m*, 2 H, C*H*), 2.08–1.79 (*m*, 4 H, C*H*), 1.63 (*t*, *J* = 14.4 Hz, 4 H, C*H*), 1.47–0.78 (*m*, 8 H, C*H*) ppm. **^{13}C-NMR (175 MHz, $CDCl_3$):** δ = 214.9, 171.6, 60.7, 52.7, 41.4, 37.7, 35.0, 34.4, 33.5, 32.7, 26.4, 26.3, 19.7 ppm. **IR ($CDCl_3$):** $\tilde{\nu}$ = 2924, 2851, 1753, 1722, 1449, 1406, 1250, 1212, 1152, 1006, 945, 895, 847, 518, 445 cm^{-1}. **HRMS (ESI) *m/z*:** calculated for $C_{14}H_{23}O_3$ $[M+H]^+$: 239.1642; measured 239.1639.

6.28 Ethyl (*R*)-1-benzyl-2-oxocyclopentane-1-carboxylate 3bA

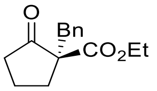

3bA

Compound **3bA** was synthesized according to **GP 9** using catalyst **C5** (1.21 mg, 1.5 µmol, 0.5 mol%), ethyl 2-oxocyclopentane-1-carboxylate **1b** (44.7 µL, 0.30 mmol, 1.00 eq.), benzyl bromide **2A** (71.7 µL, 0.60 mmol, 2.00 eq.) and DIPEA (68.5 µL, 0.39 mmol, 1.30 eq.). The reaction was stirred for 20 h. The product **3bA** (71.1 mg, 0.29 mmol, 96%, 90% *ee*) was obtained as a colorless oil. The enantiomeric excess was determined by HPLC on chiral stationary phase. CHIRALPAK® *IA*, *n*-hexane/*i*PrOH = 98/2, 0.5 mL/min, λ = 215 nm, t_{R1} = 16.3 min, t_{R2} = 18.63 min. $[\alpha]_D^{20}$ = –22 (*c* = 1.0 mg/mL, $CHCl_3$, 90% *ee*); Lit.[32] $[\alpha]_D^{20}$ = –56.6 (*c* = 0.79 mg/mL, $CHCl_3$, 81% *ee*$_{(R)}$).

$C_{15}H_{18}O_3$, MW: 246.12 g/mol. **^{1}H-NMR (300 MHz, $CDCl_3$):** δ = 7.30-7.08 (*m*, 5 H, Ar-*H*), 4.18 (*q*, *J* = 7.2 Hz, 2 H, $CO_2CH_2CH_3$), 3.20 (*d*, *J* = 13.6 Hz, 1 H, Ar-CH_2), 3.12 (*d*, *J* = 13.6 Hz, 1 H, Ar-CH_2), 2.47-2.29 (*m*, 2 H, C*H*), 2.09-1.79 (*m*, 3 H, C*H*), 1.67-1.56 (*m*, 1 H, C*H*), 1.25 (*t*, *J* = 6.9 Hz, 3 H, $CO_2CH_2CH_3$) ppm.

The analytical data are consistent with the literature.[32]

6.29 Allyl (*R*)-1-benzyl-2-oxocyclopentane-1-carboxylate 3cA

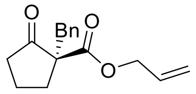

3cA

Compound **3cA** was synthesized according to **GP 9** using catalyst **C5** (1.21 mg, 1.5 µmol, 0.5 mol%), allyl 2-oxocyclopentane-1-carboxylate **1c** (51.0 mg, 0.30 mmol, 1.00 eq.), benzyl bromide **2A** (71.7 µL, 0.60 mmol, 2.00 eq.) and DIPEA (68.5 µL, 0.39 mmol, 1.30 eq.). The reaction was stirred for 20 h. The product **3cA** (76.0 mg, 0.29 mmol, 97%, 91% *ee*) was obtained as a colorless oil. The enantiomeric excess was determined by HPLC on chiral stationary phase. CHIRALPAK® *IA*, *n*-hexane/iPrOH = 99/1, 0.4 mL/min, λ = 215 nm, t_{R1} = 49.4 min, t_{R2} = 58.1 min. $[\alpha]_D^{20}$ = –32 (*c* = 1.0 mg/mL, $CHCl_3$, 91% *ee*); Lit.[33] $[\alpha]_D^{27}$ = +17.01 (*c* = 0.2 mg/mL, $CHCl_3$, 98% $ee_{(S)}$).

$C_{16}H_{18}O_3$, MW: 258.31 g/mol. **^{1}H-NMR (400 MHz, $CDCl_3$):** δ = 7.30-7.20 (*m*, 3 H, Ar-*H*), 7.17-7.09 (*m*, 2 H, Ar-*H*), 5.96-5.80 (*m*, 1 H, CH_2CHCH_2), 5.35-5.20 (*m*, 2 H, CH_2CHCH_2), 4.61 (*dq*, *J* = 5.6, 0.75 Hz, 2 H, CH_2CHCH_2), 3.21 (*d*, *J* = 13.7 Hz, 1 H, Ar-CH_2), 3.14 (*d*, *J* = 14.0 Hz, 1 H, Ar-CH_2), 2.48-2.30 (*m*, 2 H, C*H*), 2.13-1.80 (*m*, 3 H, C*H*), 1.68-1.55 (*m*, 1 H, C*H*) ppm.

The analytical data are consistent with the literature.[33]

6.30 Isopropyl (*R*)-1-benzyl-2-oxocyclopentane-1-carboxylate 3dA

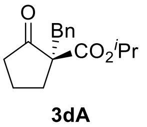

3dA

Compound **3dA** was synthesized according to **GP 9** using catalyst **C5** (1.21 mg, 1.5 µmol, 0.5 mol%), isopropyl 2-oxocyclopentane-1-carboxylate **1d** (48.6 µL, 0.30 mmol, 1.00 eq.), benzyl bromide **2A** (107.6 µL, 0.90 mmol, 3.00 eq.) and DIPEA (68.5 µL, 0.39 mmol, 1.30 eq.). The reaction was stirred for 20 h. The product **3dA** (58.3 mg, 0.22 mmol, 74%, 89% *ee*) was

obtained as a colorless oil. The enantiomeric excess was determined by HPLC on chiral stationary phase. CHIRALPAK® *IA*, *n*-hexane/*i*PrOH = 98/2, 0.5 mL/min, λ = 215 nm, t_{R1} = 13.6 min, t_{R2} = 16.9 min.

$C_{16}H_{20}O_3$, MW: 260.33 g/mol. **$[\alpha]_D^{20}$**(c = 1.0 mg/mL, $CHCl_3$, 89% *ee*) = –26. **^{1}H-NMR (300 MHz, $CDCl_3$):** δ = 7.29-7.17 (*m*, 3 H, Ar-*H*), 7.16-7.09 (*m*, 2 H, Ar-*H*), 5.01 (*sept.*, 1 H, C*H*(CH_3)$_2$), 3.15 (*dd*, *J* = 18.9, 13.7 Hz, 2 H, Ar-C*H*$_2$), 2.45-2.29 (*m*, 2 H, C*H*), 2.07-1.08 (*m*, 3 H, C*H*), 1.67-1.50 (*m*, 1 H, C*H*), 1.22 (*dd*, *J* = 6.2, 2.7 Hz, 6 H, CH(C*H*$_3$)$_2$) ppm. **^{13}C-NMR (175 MHz, $CDCl_3$):** δ = 215.3, 170.8, 136.9, 130.4, 128.5, 126.9, 69.3, 61.5, 38.9, 38.5, 31.9, 21.8, 21.7, 19.6 ppm. **IR ($CDCl_3$):** $\tilde{\nu}$ = 3063, 3030, 2979, 2938, 2890, 1749, 1721, 1604, 1496, 1467, 1454, 1405, 1387, 1375, 1316, 1266, 1236, 1181, 1163, 1145, 1103, 1076, 1033, 1003, 921, 853, 827, 747, 703, 579, 509, 419 cm^{-1}. **HRMS (ESI) *m/z*:** calculated for $C_{16}H_{20}O_3NH_4$ $[M+NH_4]^+$: 278.1751; measured 278.1747.

6.31 Benzyl (*R*)-1-benzyl-2-oxocyclopentane-1-carboxylate 3eA

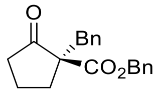

3eA

Compound **3eA** was synthesized according to **GP 9** using catalyst **C5** (1.21 mg, 1.5 µmol, 0.5 mol%), benzyl 2-oxocyclopentane-1-carboxylate **1e** (66.0 mg, 0.30 mmol, 1.00 eq.), benzyl bromide **2A** (107.7 µL, 0.90 mmol, 3.00 eq.) and DIPEA (68.5 µL, 0.39 mmol, 1.30 eq.). The reaction was stirred for 20 h. The product **3eA** (65.7 mg, 0.21 mmol, 70%, 85% *ee*) was obtained as a colorless oil. The enantiomeric excess was determined by HPLC on chiral stationary phase. CHIRALPAK® *IA*, *n*-hexane/*i*PrOH = 97/3, 0.5 mL/min, λ = 215 nm, t_{R1} = 18.2 min, t_{R2} = 21.0 min.

$C_{20}H_{20}O_3$, MW: 308.37 g/mol. **$[\alpha]_D^{20}$**(c = 1.0 mg/mL, $CHCl_3$, 85% *ee*) = –6. **^{1}H-NMR (300 MHz, $CDCl_3$):** δ = 7.41-7.27 (*m*, 5 H, Ar-*H*), 7.24-7.18 (*m*, 3 H, Ar-*H*), 7.13-7.04 (*m*, 2 H, Ar-*H*), 5.15 (*dd*, *J* = 13.0, 13.0 Hz, 2 H, Ar-C*H*$_2$), 3.22 (*d*, *J* = 13.4 Hz, 1 H, Ar-C*H*$_2$), 3.13 (*d*, *J* = 14.0 Hz, 1 H, Ar-C*H*$_2$), 2.47-2.28 (*m*, 2 H, C*H*), 2.12-1.77 (*m*, 3 H, C*H*), 1.64-1.51 (*m*, 1 H, C*H*) ppm. **^{13}C-NMR (175 MHz, $CDCl_3$):** δ = 214.9, 171.0, 136.6, 135.6, 130.3, 128.7, 128.5, 128.5, 128.2, 127.0, 67.3, 61.6, 39.2, 38.5, 31.8, 19.6 ppm. **IR ($CDCl_3$):** $\tilde{\nu}$ = 3031, 2962, 2890, 1751, 1726, 1604, 1497, 1454, 1403, 1374, 1314, 1264, 1218, 1157, 1140, 1102, 1076, 1031, 1004, 923, 746, 700, 602, 498, 409 cm^{-1}. **HRMS (EI) *m/z*:** calculated for $C_{20}H_{20}O_3Na$ $[M+Na]^+$: 331.1305; measured 331.1299.

6.32 Methyl (*R*)-1-benzyl-4-methyl-2-oxocyclopent-3-ene-1-carboxylate 3fA

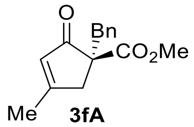

3fA

Compound **3fA** was synthesized according to **GP 9** using catalyst **C5** (2.42 mg, 3.0 µmol, 1.0 mol%), methyl 4-methyl-2-oxocyclopent-3-ene-1-carboxylate **1f** (46.6 mg, 0.30 mmol, 1.00 eq.), benzyl bromide **2A** (71.7 µL, 0.60 mmol, 2.00 eq.) and DIPEA (68.5 µL, 0.39 mmol, 1.30 eq.). The reaction was stirred for 20 h. The product **3fA** (67.9 mg, 0.28 mmol, 92%, 95% *ee*) was obtained as a colorless oil. The enantiomeric excess was determined by HPLC on chiral stationary phase. CHIRALPAK® *AD-H*, *n*-hexane/*i*PrOH = 97/3, 0.8 mL/min, λ = 215 nm, t_{R1} = 22.7 min, t_{R2} = 27.1 min.

$C_{15}H_{16}O_3$, MW: 244.29 g/mol. **$[\alpha]_D^{20}$**(c = 1.0 mg/mL, $CHCl_3$, 95% *ee*) = –138. **^{1}H-NMR (300 MHz, $CDCl_3$):** δ = 7.24-7.04 (*m*, 5 H, Ar-*H*), 5.82-5.74 (*m*, 1 H, C=C*H*), 3.73 (*s*, 3 H, CO_3CH_3), 3.29 (*d*, *J* = 13.9 Hz, 1 H, Ar-CH_2), 3.20 (*d*, *J* = 13.9 Hz, 1 H, Ar-CH_2), 3.06 (*d*, *J* = 19.0 Hz, 1 H, C*H*), 2.57 (*d*, *J* = 19.0 Hz, 1 H, C*H*), 2.00 (*d*, *J* = 0.6 Hz, 3 H, C=CH_3) ppm. **^{13}C-NMR (175 MHz, $CDCl_3$):** δ = 204.9, 178.9, 171.3, 136.4, 130.1, 128.5, 128.4, 127.0, 60.8, 53.0, 41.9, 39.5, 19.4 ppm. **IR ($CDCl_3$):** $\tilde{\nu}$ = 3085, 3062, 3029, 3004, 2981, 2952, 2920, 2846, 1740, 1700, 1624, 1604, 1584, 1496, 1454, 1433, 1378, 1323, 1287, 1253, 1172, 1145, 1121, 1086, 1068, 1030, 977, 952, 920, 902, 846, 816, 789, 774, 752, 705, 656, 605, 579, 542, 518, 488, 462, 435 cm^{-1}. **HRMS (EI) *m/z*:** calculated for $C_{15}H_{16}O_3$ $[M]^+$: 244.1094; measured 244.1094.

6.33 Methyl (*R*)-1-benzyl-3-methyl-2-oxocyclopent-3-ene-1-carboxylate 3gA

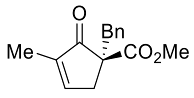

3gA

Compound **3gA** was synthesized according to **GP 9** using catalyst **C5** (2.4 mg, 3.0 µmol, 1.0 mol%), methyl 3-methyl-2-oxocyclopent-3-ene-1-carboxylate **1g** (46.6 mg, 0.30 mmol, 1.00 eq.), benzyl bromide **2A** (71.7 µL, 0.60 mmol, 2.00 eq.) and DIPEA (68.5 µL, 0.39 mmol, 1.30 eq.). The reaction was stirred for 20 h. The product **3gA** (77.0 mg, 0.27 mmol, 90%, 78% *ee*) was obtained as a colorless oil. The enantiomeric excess was determined by HPLC on chiral stationary phase. CHIRALPAK® *AD-H*, *n*-hexane/*i*PrOH = 97/3, 0.5 mL/min, λ = 215 nm, t_{R1} = 14.6 min, t_{R2} = 18.3 min.

$C_{15}H_{16}O_3$, MW: 244.29 g/mol. **$[\alpha]_D^{20}$**(c = 1.0 mg/mL, $CHCl_3$, 78% *ee*) = –124. **^{1}H-NMR (300 MHz, $CDCl_3$):** δ = 7.25–7.13 (*m*, 4 H, Ar-*H*), 7.11–7.02 (*m*, 2 H, Ar-*H,* C=C*H*), 3.72 (*s*, 3 H, CH_3), 3.25 (*s*, 2 H, CH_2), 3.02 (*dt*, *J* = 19.0, 2.4 Hz, 1 H, C*H*), 2.57 (*dt*, *J* = 18.9, 2.4 Hz, 1 H, C*H*), 1.70 (*q*, *J* = 2.0 Hz, 3 H, C=CCH_3) ppm. **^{13}C-NMR (175 MHz, $CDCl_3$):** δ = 205.6, 171.5, 157.7, 140.3, 136.3, 130.1, 128.3, 127.0, 58.9, 52.9, 39.7, 36.1, 10.5 ppm. **IR ($CDCl_3$):** $\tilde{\nu}$ = 2952, 2923, 2852, 2360, 1740, 1704, 1679, 1603, 1513, 1413, 1254, 1240, 1177, 1122, 703 cm^{-1}. **HRMS (ESI) *m/z*:** calculated for $C_{15}H_{17}O_3$ $[M+H]^+$: 245.1172; measured 245.1168.

6.34 Methyl (*R*)-1-benzyl-2-oxocyclohexane-1-carboxylate 3hA

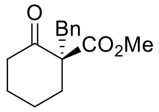

3hA

Compound **3hA** was synthesized according to **GP 9** using catalyst **C5** (4.0 mg, 5.0 µmol, 2.5 mol%), methyl 2-oxocyclohexane-1-carboxylate **1h** (28.5 µL, 0.20 mmol, 1.00 eq.), benzyl bromide **2A** (47.6 µL, 0.40 mmol, 2.00 eq.) and DIPEA (45.5 µL, 0.26 mmol, 1.30 eq.). The reaction was stirred for 20 h. The product **3hA** (46.2 mg, 0.18 mmol, 94%, 93% *ee*) was obtained as a colorless oil. The enantiomeric excess was determined by HPLC on chiral stationary phase. CHIRALPAK® *IC*, *n*-hexane/iPrOH = 97/3, 0.5 mL/min, λ = 215 nm, t_{R1} = 29.4 min, t_{R2} = 31.9 min. $[\alpha]_D^{20}$ = 90 (c = 1.0 mg/mL, EtOH, 93% *ee*); Lit.[34] $[\alpha]_D^{26}$ = –110.5 (c = 0.42 mg/mL, EtOH, 99% $ee_{(S)}$).

$C_{15}H_{18}O_3$, MW: 246.30 g/mol. **^{1}H-NMR (300 MHz, $CDCl_3$):** δ = 7.31–7.14 (*m*, 3 H, Ar-*H*), 7.09 (*dd*, *J* = 7.7, 1.8 Hz, 2 H, Ar-*H*), 3.64 (*s*, 3 H, CH_3), 3.32 (*d*, *J* = 13.7 Hz, 1 H, Ar-CH_2), 2.87 (*d*, *J* = 13.7 Hz, 1 H, Ar-CH_2), 2.55 – 2.31 (*m*, 3 H, C*H*), 2.00 (*dq*, *J* = 9.4, 3.1 Hz, 1 H, C*H*), 1.83–1.34 (*m*, 4 H, C*H*) ppm.

The analytical data are consistent with the literature.[34]

6.35 Methyl (*S*)-2-benzyl-1-oxo-2,3-dihydro-1*H*-indene-2-carboxylate 3iA

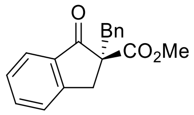

3iA

Compound **3iA** was synthesized according to **GP 9** using catalyst **C5** (4.0 mg, 5.0 µmol, 5.0 mol%), methyl 1-oxo-2,3-dihydro-1H-indene-2-carboxylate **1i** (19.0 mg, 0.10 mmol, 1.00 eq.), benzyl bromide **2A** (23.8 µL, 0.20 mmol, 2.00 eq.) and aq. 30% K_2CO_3 solution (100 µL, 0.28 mmol, 2.80 eq.). The reaction was stirred for 20 h. The reaction mixture was diluted with demineralized water (2 mL), the phases were separated, and the aqueous phase was extracted with DCM (3 × 1 mL). The combined organic phases were dried over Na_2SO_4, filtered, and the solvent was removed under reduced pressure. Purification by preparative thin-layer chromatography on silica gel (PE/EtOAc 10:1) afforded product **3iA** (24.6 mg, 87.8 µmol, 88%, 84% *ee*) as a colorless oil. The enantiomeric excess was determined by HPLC on chiral stationary phase. CHIRALPAK® *AD-H*, *n*-hexane/iPrOH = 98/2, 0.8 mL/min, λ = 215 nm, t_{R1} = 32.4 min, t_{R2} = 43.8 min. $[\alpha]_D^{20}$ = –130.0 (*c* = 1.0 mg/mL, EtOH, 84% *ee*); Lit.[35] $[\alpha]_D^{20}$= +144 (*c* = 1.0 mg/mL, EtOH, *(R)*).

$C_{18}H_{16}O_3$, MW: 280.32 g/mol. **^{1}H-NMR (300 MHz, $CDCl_3$):** δ = 7.73 (*d*, *J* = 7.6 Hz, 1 H, Ar-*H*), 7.58-7.49 (*m*, 1 H, Ar-*H*), 7.37-7.28 (*m*, 2 H, Ar-*H*), 7.22-7.07 (*m*, 5 H, Ar-*H*), 3.71 (*s*, 3 H, CH_3), 3.62 (*d*, *J* = 17.7, 1 H, C*H*), 3.49 (*d*, *J* = 13.9, 1 H, Ar-CH_2), 3.28 (*d*, *J* = 13.9, 1 H, Ar-CH_2), 3.17 (*d*, *J* = 17.7, 1 H, C*H*) ppm.

The analytical data are consistent with the literature.[36]

6.36 Ethyl (*R*)-2-benzyl-2-methyl-3-oxo-3-phenylpropanoat 3jA

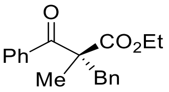

3jA

Compound **3jA** was synthesized according to **GP 9** using catalyst **C5** (4.0 mg, 5.0 µmol, 5.0 mol%), ethyl 2-methyl-3-oxo-3-phenylpropanoate **1j** (19.1 µL, 0.10 mmol, 1.00 eq.), benzyl bromide **2A** (23.8 µL, 0.20 mmol, 2.00 eq.) and NaOH (5.2 mg, 0.13 mmol, 1.30 eq.) under nitrogen atmosphere and dry solvent. The reaction was stirred for 20 h. The reaction mixture was diluted with demineralized water (2 mL), the phases were separated, and the aqueous phase was extracted with DCM (3 × 1 mL). The combined organic phases were dried over Na_2SO_4, filtered, and the solvent was removed under reduced pressure. Purification by preparative thin-layer chromatography on silica gel (PE/EtOAc 10:1) afforded product **3jA** (20.9 mg, 70.5 µmol, 71%, 90% *ee*) as a colorless oil. The enantiomeric excess was determined by HPLC on chiral stationary phase. CHIRALPAK® *IJ*, *n*-hexane/iPrOH = 97/3,

0.5 mL/min, λ = 215 nm, t_{R1} = 29.1 min, t_{R2} = 33.2 min. $[\alpha]_D^{20}$(c = 1.0 mg/mL, $CHCl_3$, 90% *ee*) = –30.

$C_{19}H_{20}O_3$, MW: 296.36 g/mol. **^{1}H-NMR (300 MHz, $CDCl_3$):** δ = 7.91–7.77 (*m,* 2 H, Ar-*H*), 7.59–7.48 (*m*, 1 H, Ar-*H*), 7.48–7.35 (*m*, 2 H, Ar-*H*), 7.26–7.14 (*m*, 3 H, Ar-*H*), 7.09–6.97 (*m*, 2 H, Ar-*H*), 4.09 (*qq*, *J* = 7.0, 3.6 Hz, 2 H, $CO_2CH_2CH_3$), 3.42 (*d*, *J* = 13.8 Hz, 1 H, Ar-CH_2), 3.33 (*d*, *J* = 13.8 Hz, 1 H, Ar-CH_2), 1.48 (*s*, 3 H, CH_3), 1.03 (*t*, *J* = 7.1 Hz, 3 H, $CO_2CH_2CH_3$) ppm.

The analytical data are consistent with the literature.[37]

6.37 (*R*)-*N*-Allyl-1-benzyl-2-oxocyclopentane-1-carboxamide 3kA

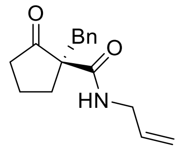

3kA

Compound **3kA** was synthesized according to **GP 9** using catalyst **C5** (4.0 mg, 5.0 µmol, 2.5 mol%), *N*-allyl-2-oxocyclopentane-1-carboxamide **1k** (33.4 mg, 0.20 mmol, 1.00 eq.), benzyl bromide **2A** (47.5µL, 0.40 mmol, 2.00 eq.) and DIPEA (45.4 µL, 0.26 mmol, 1.30 eq.). The reaction was stirred for 20 h. The product **3kA** (46.1 mg, 0.18 mmol, 90%, 73% *ee*) was obtained as a colorless oil. The enantiomeric excess was determined by HPLC on chiral stationary phase. CHIRALPAK® *OD-H*, *n*-hexane/*i*PrOH = 90/10, 1.0 mL/min, λ = 215 nm, t_{R1} = 9.1 min, t_{R2} = 9.7 min.

$C_{16}H_{19}NO_2$, MW: 257.33 g/mol. **$[\alpha]_D^{20}$** (c = 1.0 mg/mL, $CHCl_3$, 73% *ee*) = –6. **^{1}H-NMR (300 MHz, $CDCl_3$):** δ = 7.33–7.17 (*m*, 3 H, Ar-*H*), 7.12–7.03 (*m*, 2 H, Ar-*H*), 6.78 (*br*, 1 H, N*H*), 5.80 (*ddd*, *J* = 22.5, 10.8, 5.6 Hz, 1 H, *H*C=CH_2), 5.21 – 5.07 (*m*, 2 H, HC=CH_2), 3.98–3.72 (*m*, 2 H, CH_2), 3.15 (*d*, *J* = 13.5 Hz, 1 H, C*H*), 2.96 (*d*, *J* = 13.5 Hz, 1 H, C*H*), 2.70–2.55 (*m*, 1 H, C*H*), 2.38–2.22 (*m*, 1 H, C*H*), 2.22–2.05 (*m*, 1 H, C*H*), 2.04–1.86 (*m*, 1 H, C*H*), 1.83–1.67 (*m*, 1 H, C*H*), 1.65–1.53 (*m*, 1 H, C*H*) ppm. **^{13}C-NMR (175 MHz, $CDCl_3$):** δ = 169.0, 136.2, 134.0, 130.0, 128.5, 127.3, 116.6, 62.0, 43.3, 42.3, 39.5, 30.9, 19.0 ppm. **IR ($CDCl_3$):** $\tilde{\nu}$ = 3372, 3062, 3030, 2923, 1726, 1669, 1522, 1454, 1404, 1272, 1151, 1001, 923, 702 cm^{-1}. **HRMS (ESI) *m/z***: calculated for $C_{16}H_{19}NO_2Na$ [M+Na]$^+$: 280.1308; measured 280.1307.

6.38 (*R*)-*N*,1-Dibenzyl-2-oxocyclopentane-1-carboxamide 3lA

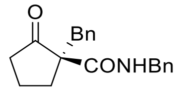

3lA

Compound **3lA** was synthesized according to **GP 9** using catalyst **C5** (4.0 mg, 5.0 µmol, 2.5 mol%), *N*-benzyl-2-oxocyclopentane-1-carboxamide **1l** (43.5 mg, 0.20 mmol, 1.00 eq.), benzyl bromide **2A** (47.5µL, 0.40 mmol, 2.00 eq.) and DIPEA (45.4 µL, 0.26 mmol, 1.30 eq.). The reaction was stirred for 20 h. The product **3lA** (54.6 mg, 0.18 mmol, 89%, 80% *ee*) was obtained as a colorless oil. The enantiomeric excess was determined by HPLC on chiral stationary phase. CHIRALPAK® *OD-H*, *n*-hexane/*i*PrOH = 90/10, 1.0 mL/min, λ = 215 nm, t_{R1} = 14.1 min, t_{R2} = 15.9 min.

$C_{20}H_{21}NO_2$, MW: 307.39 g/mol. **$[\alpha]_D^{20}$** (c = 1.0 mg/mL, $CHCl_3$, 80% *ee*) = +8. **^{1}H-NMR (300 MHz, $CDCl_3$):** δ = 7.41–7.19 (*m*, 8 H, Ar-*H*), 7.11–7.00 (*m*, 2 H, Ar-*H*), 4.50 (*dd*, *J* = 14.8, 6.0 Hz, 1 H, Ar-C*H*$_2$), 4.35 (*dd*, *J* = 14.8, 5.5 , 1 H, Ar-C*H*$_2$), 3.16 (*d*, *J* = 13.5 Hz, 1 H, Ar-C*H*$_2$), 2.99 (*d*, *J* = 13.5 Hz, 1 H, Ar-C*H*$_2$), 2.67 (*dt*, *J* = 13.5, 6.8 Hz, 1 H, C*H*), 2.43–2.26 (*m*, 1 H, C*H*), 2.25–2.08 (*m*, 1 H, C*H*), 2.00 (*dt*, *J* = 13.7, 7.0 Hz, 1 H, C*H*), 1.85–1.46 (*m*, 2 H, C*H*) ppm. **^{13}C-NMR (175 MHz, $CDCl_3$):** δ = 169.1, 138.1, 136.1, 130.0, 128.8, 128.5, 127.9, 127.6, 127.3, 61.9, 44.0, 43.3, 39.4, 31.0, 19.0 ppm. **IR ($CDCl_3$):** $\tilde{\nu}$ = 3376, 3061, 3029, 2956, 2925, 1726, 1668, 1604, 1585, 1523, 1497, 1454, 1403, 1360, 1240, 1151, 1078, 1030, 1004, 924, 847, 820, 737, 700, 602, 508, 452, 425 cm^{-1}. **HRMS (EI) *m/z*:** calculated for $C_{20}H_{21}NO_2$ $[M]^+$: 307.1567; measured 307.1566.

6.39 Ethyl (*S*)-1-methyl-2-oxocyclohexane-1-carboxylate 3mU

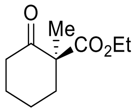

3mU

Compound **3mU** was synthesized according to **GP 9** using catalyst **C5** (4.0 mg, 5.0 µmol, 2.5 mol%), ethyl 2-oxocyclohexane-1-carboxylate **1m** (32.1 µL, 0.20 mmol, 1.00 eq.), methyl iodide **2U** (50.2 µL, 0.80 mmol, 4.00 eq.) and DIPEA (45.7 µL, 0.26 mmol, 1.30 eq.). The reaction was stirred for 48 h. Column chromatography on silica gel (*n*-pentane/Et_2O, 2:1) afforded product **3mU** (32.6 mg, 0.18 mmol, 88%, 94% *ee*) as a colorless oil. The enantiomeric excess was by determined by GC using the Bondex un alpha+beta column with H_2 carrier gas

40 °C for 1 min then with 0.5 °C/min to 200 °C, t_{R1} = 93.98 min, t_{R2} = 96.10 min. $[\alpha]_D^{20}$ = 13 (*c* = 1.0 mg/mL, DCM, 94% *ee*); Lit.[38] $[\alpha]_D^{23}$ = −101 (*c* = 2.92 mg/mL, DCM, 96% $ee_{(R)}$).

$C_{10}H_{16}O_3$, MW: 184.23 g/mol. **^{1}H-NMR (300 MHz, $CDCl_3$):** δ = 4.19 (*qd*, *J* = 7.1, 1.9 Hz, 2 H, $CO_2CH_2CH_3$), 2.58–2.42 (*m*, 3 H, C*H*), 2.11–1.93 (*m*, 1 H, C*H*), 1.77–1.57 (*m*, 3 H, C*H*), 1.52–1.38 (*m*, 1 H, C*H*), 1.29 (*s*, 3 H, CH_3), 1.28–1.23 (*m*, 3 H, CO_2CH_3) ppm.

The analytical data are consistent with the literature.[38]

6.40 Methyl (*S*)-1-methyl-2-oxocycloheptane-1-carboxylate 3nU

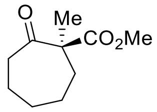

3nU

Compound **3nU** was synthesized according to **GP 9** using catalyst **C5** (4.0 mg, 5.0 µmol, 2.5 mol%), methyl 2-oxocycloheptane-1-carboxylate **1n** (32.0 µL, 0.20 mmol, 1.00 eq.), methyl iodide **2U** (50.2 µL, 0.80 mmol, 4.00 eq.) and DIPEA (45.7 µL, 0.26 mmol, 1.30 eq.). The reaction was stirred for 48 h. Column chromatography on silica gel (*n*-pentane/Et_2O, 2:1) afforded product **3nU** (26.8 mg, 0.15 mmol, 76%, 91% *ee*) as a colorless oil. The enantiomeric excess was by determined by chiral GC analysis using ChiralDex-B-DM column with H_2 carrier gas 40 °C for 1 min then with 1 °C/min to 200 °C, t_{R1} = 56.95 min, t_{R2} = 58.16 min. $[\alpha]_D^{20}$ = −12 (*c* = 1.0 mg/mL, $CHCl_3$, 91% *ee*); Lit.[39] $[\alpha]_D^{26}$ = +10.5 (*c* = 1.0 mg/mL, $CHCl_3$, 14% $ee_{(R)}$).

$C_{10}H_{16}O_3$, MW: 184.23 g/mol. **^{1}H-NMR (300 MHz, $CDCl_3$):** δ = 3.71 (*s*, 3 H, CO_2CH_3), 2.80–2.34 (*m*, 2 H, C*H*), 2.25–2.03 (*m*, 1 H, C*H*), 1.99–1.47 (*m*, 6 H, C*H*), 1.48–1.36 (*m*, 1 H, C*H*), 1.34 (*s*, 3 H, CH_3) ppm.

The analytical data are consistent with the literature.[39]

6.41 Ethyl (*R*)-1-methyl-2-oxocyclooctane-1-carboxylate 3oU

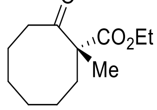

3oU

Compound **3oU** was synthesized according to **GP 9** using catalyst **C5** (4.0 mg, 5.0 µmol, 2.5 mol%), ethyl 2-oxocyclooctane-1-carboxylate **1o** (40.0 mg, 0.20 mmol, 1.00 eq.), methyl iodide **2U** (50.2 µL, 0.80 mmol, 4.00 eq.) and DIPEA (45.5 µL, 0.26 mmol, 1.30 eq.). The reaction was stirred for 48 h. Column chromatography on silica gel (*n*-pentane/Et_2O, 2:1) afforded product **3oU** (31.0 mg, 0.14 mmol, 72%, 88% *ee*) as a colorless oil. The enantiomeric excess was determined by GC analysis using ChiralDex-B-DM column with H_2 carrier gas 40 °C for 1 min then with 1 °C/min to 80 °C for 3 min then with 0.5 °C/min 200 °C, t_{R1} = 89.86 min, t_{R2} = 91.31 min. $[\alpha]_D^{20}$ (*c* = 1.0 mg/mL, $CHCl_3$, 88% *ee*) = 26.

$C_{12}H_{20}O_3$, MW: 212.28 g/mol. **^{1}H-NMR (300 MHz, $CDCl_3$):** *δ* = 4.15 (*qq*, *J* = 10.8, 7.1 Hz, 2 H, $CO_2CH_2CH_3$), 2.82 (*td*, *J* = 12.2, 3.7 , 1 H, C*H*), 2.56 (*ddd*, *J* = 15.5, 12.0, 4.1 Hz, 1 H, C*H*), 2.30 (*ddd*, *J* = 12.2, 6.1, 3.9 Hz, 1 H, C*H*), 1.94 (*dt*, *J* = 15.0, 4.5 Hz, 1 H, C*H*), 1.88–1.45 (*m*, 6 H, C*H*), 1.41–1.29 (*m*, 4 H, C*H*), 1.23 (*t*, *J* = 7.1 Hz, 3 H, $CO_2CH_2CH_3$), 1.04–0.90 (*m*, 1 H, C*H*) ppm.

The analytical data are consistent with the literature.[40]

7 Reaction Parameter Screening

Leaving group

Table S1: Leaving group screening.

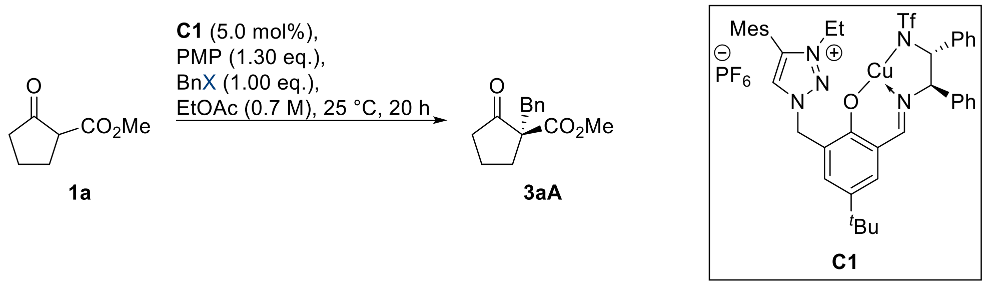

#	BnX	Yield[a] [%]	*ee*[b] [%]
**1**	BnCl	4	83
**2**	BnBr	>99	89
**3**	BnOMs	80	91
**4**	BnOTs	83	92
**5a**	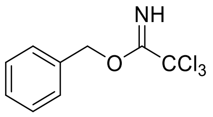	0	-
**5b**[c]		0	-

Catalytic reactions were conducted at a 0.10 mmol scale under nitrogen atmosphere. [a]Determined by ^{1}H-NMR analysis of the crude product using mesitylene as internal standard. [b]Determined by chiral HPLC analysis. [c]PMP (10 mol%) was added.

Catalyst counter ion

Table S2: Catalyst counter ion screening.

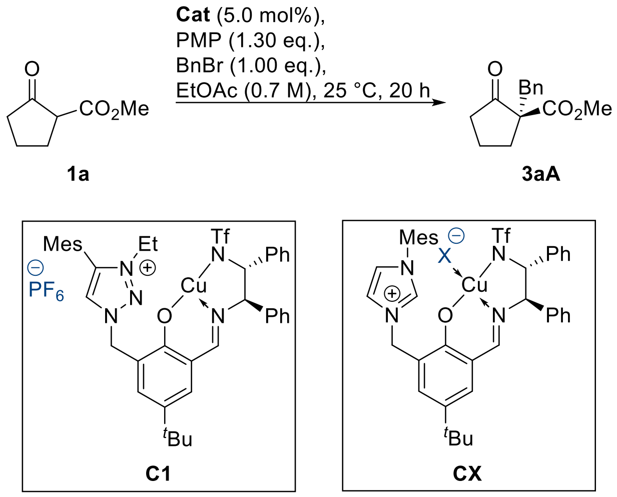

#	Cat	X	Yield[a] [%]	*ee*[b] [%]
**1**	**C1**	PF_6	>99	89
**2**	**C5**	Cl	95	89
**3**	**C12**	Br	94	89
**4**	**C13**	I	90	89

Catalytic reactions were conducted at a 0.10 mmol scale under nitrogen atmosphere. [a]Determined by ^{1}H-NMR analysis of the crude product using mesitylene as internal standard. [b]Determined by chiral HPLC analysis.

Chiral Diamine

Table S3: Screening of chiral diamine moiety.

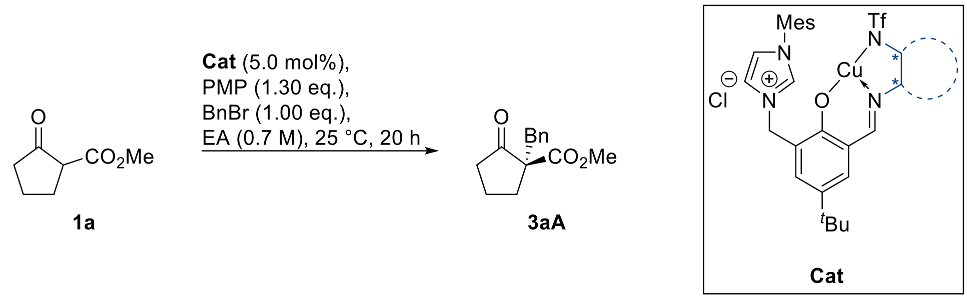

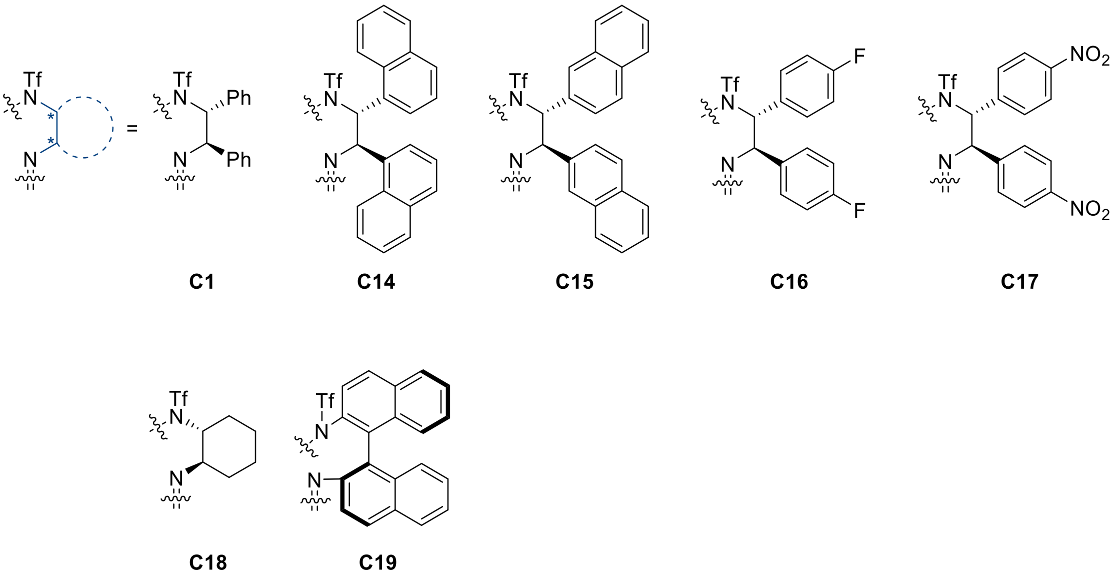

#	Cat	Yield[a] [%]	*ee*[b] [%]
1	**C1**	95	89
2	**C14**	94	85
3	**C15**	95	82
4	**C16**	93	88
5	**C17**	94	88
6	**C18**	83	–46
7	**C19**	65	–23

Catalytic reactions were conducted at a 0.10 mmol scale under nitrogen atmosphere. [a]Determined by ^{1}H-NMR analysis of the crude product using mesitylene as internal standard. [b]Determined by chiral HPLC analysis.

8 Catalyst Stability

Same Excess Protocol

A

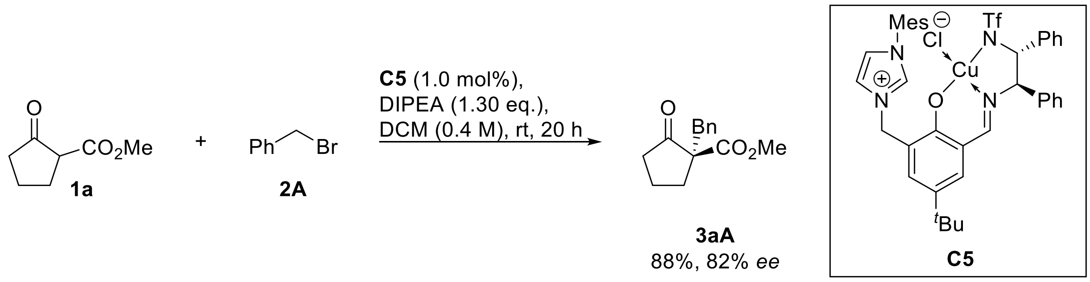

B

#	Experiment	1a	2A	DIPEA	C5	3aA	DIPEA·HBr
		**Initial concentration *c* [M]**					
**1**	*Standard*	0.4	0.4	0.5	0.004	0.0	0
**2**	*Same* [xs]	0.2	0.2	0.3	0.004	0.0	0
**3**	*Same* [xs] + ***3aA** addition*	0.2	0.2	0.3	0.004	0.2	0
**4**	*Same [xs] +* ***3aA*** *+ DIPEA · HBr addition*	0.2	0.2	0.3	0.004	0.2	0.2

C

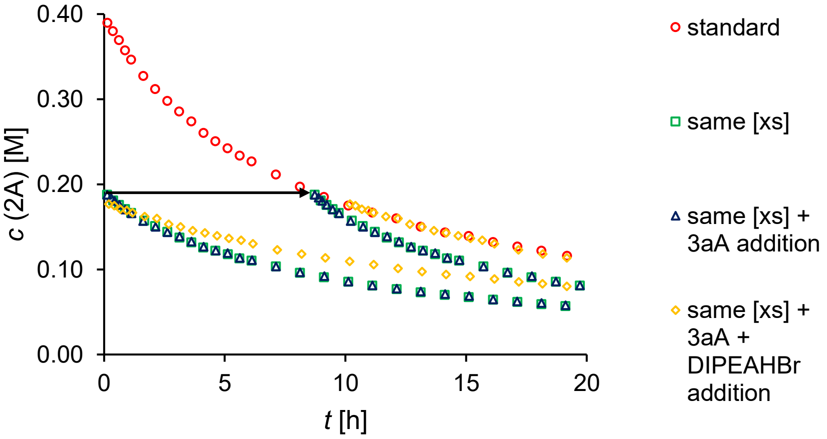

Figure S1: RPKA method: *Same Excess* protocol for the investigation of catalyst robustness and product influence.[41,42] **A:** Reaction conditions for the standard experiment. The catalytic reaction was carried out in a Schlenk tube in dry DCM (0.4 M) at room temperature, furnishing the product in 88% yield and 82% *ee*. **B:** Initial concentrations of the reaction components for each experiment. The experiments were carried out in borosilicate glass NMR tubes with screw caps in dry CD_2Cl_2 and at room temperature. The reaction was monitored using ^{1}H-NMR spectroscopy (400 MHz) with 10 number of scans and an interscan delay of 1 s. **C:** Benzyl bromide

concentration c(2A) vs. time t for each experiment. Time adjustment has been performed to correspond the initial concentration to the standard experiment.

ee and yield vs. time

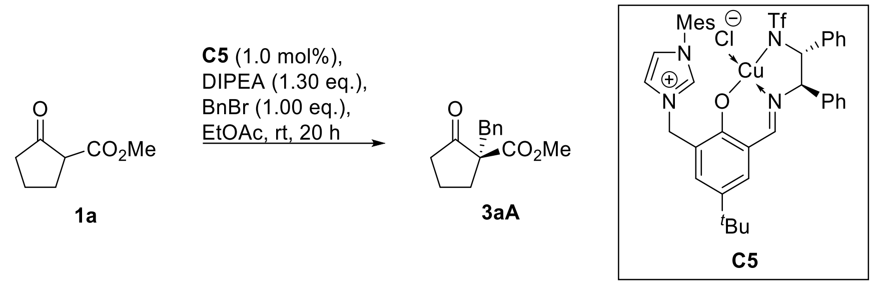

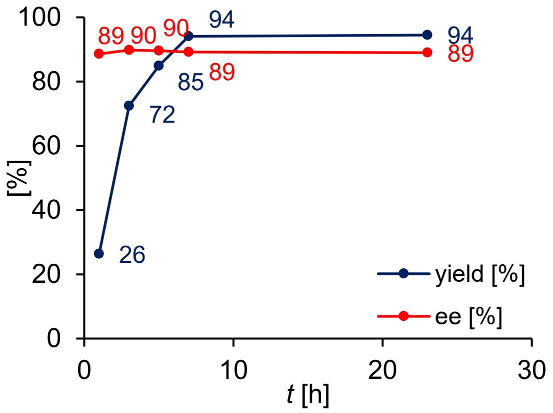

Figure S2: Yield and *ee* of the model reaction over time. Five catalytic reactions were set up simultaneously and the work up was carried out after the respective reaction time.

UV-Vis of reisolated catalyst

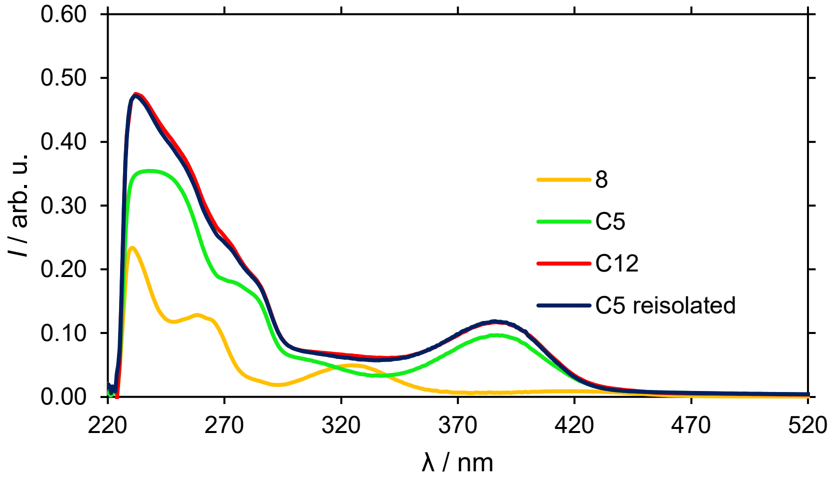

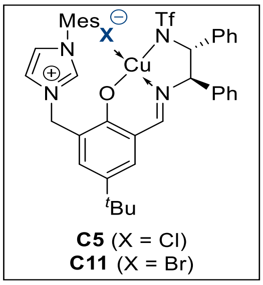

Figure S3: UV-VIS spectra of preligand **8** (yellow), the complexes **C5** (light green), **C12** (red) and the complex **C5** reisolated after catalysis (blue) in DCM at $c = 2 \cdot 10^{-5}$ M. The complex was isolated by precipitation of the supernatant of the catalytic reaction in *n*-pentane.

9 EPR Studies

All EPR Measurements were conducted with a Bruker EMX X-Band EPR spectrometer with an ER 4102ST standard rectangular resonator (TE_{102} mode) equipped with a liquid nitrogen resonator. All measurements were done in frozen solutions at 110 K in standard 3 mm i.d. quarz EPR tubes. Measurements of the catalyst were taken in EtOAc with the addition of various components of the overall reaction.

Measurements of the pure catalyst in EtOAc do not show a well resolved EPR spectrum, that still looks like a characteristic Cu(II) signal. For reference the catalyst was also measured in Me-THF to address the impact of the solvent on the catalyst. Right after dissolving the catalyst the spectra of both samples look very similar, but after 24 h in solution the signal of the Me-THF sample clears up to a well resolved spectrum that is characteristic for Cu(II). This is an indicator for a slow but quantitative coordination of the solvent. In EtOAc this effect was not observed in this timescale.

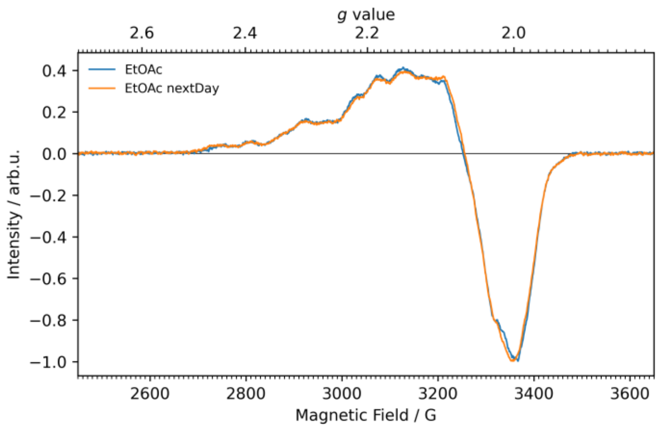

Figure S4: X-Band EPR spectrum of the catalyst in frozen solution of EtOAc (110 K) measured after few minutes in solution (blue) and after 24 h (orange).

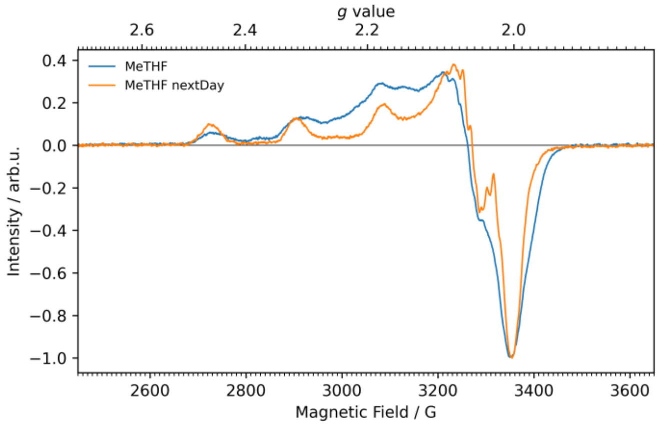

Figure S5: X-Band EPR spectrum of the catalyst in frozen solution of Me-THF(110 K) measured after few minutes in solution (blue) and after 24 h (orange).

The individual addition of the substrates to the catalyst is shown in the following figures.

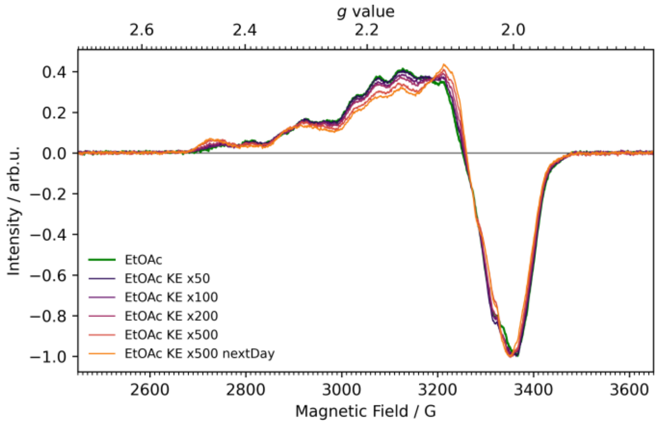

Figure S6: X-Band EPR spectrum of the catalyst in frozen solution of EtOAc (110 K) with the addition of increasing equivalents of β-ketoester (KE, **1a**) above the ratio of catalytic reactions and after 24 h in the highest ratio.

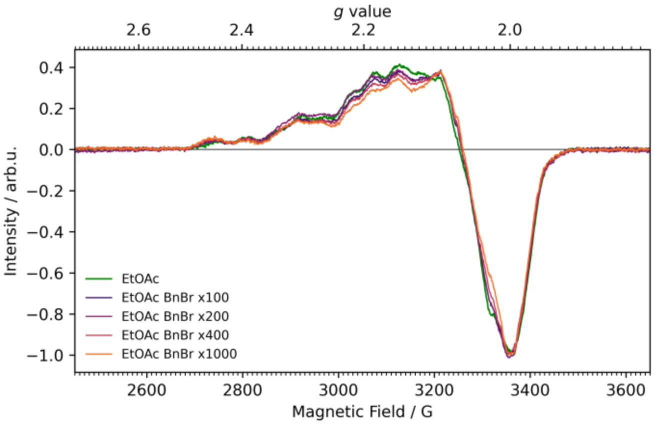

Figure S7: X-Band EPR spectrum of the catalyst in frozen solution of EtOAc (110 K) with the addition of increasing equivalents of benzylbromide (BnBr, **2A**) above the ratio of catalytic reactions and after 24 h in the highest ratio.

In both experiments the signal of the catalyst did not change over the course of the substrate addition. An extended time in solution also did not change the shape of the EPR spectrum. These results indicate that both substrates do not interact with the substrate individually.

The addition of both **1a** and DIPEA in catalytic ratios breaks up the shape of the unresolved signal clearly and shows a singular Cu(II) EPR spectrum with three well resolved peaks at low field and a derivative shaped signal at 3300 G as shown in the following figure.

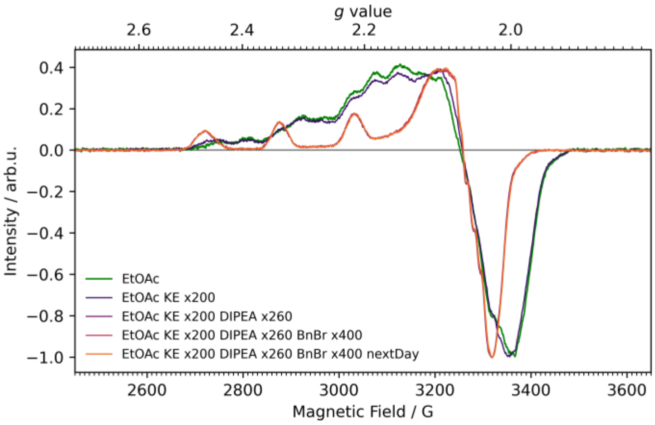

Figure S8: X-Band EPR spectrum of the catalyst in frozen solution of EtOAc (110 K) with the subsequent addition of **1a** (KE), DIPEA and BnBr (**2A**).

The spectra of the catalyst in Me-THF and the full reaction mixture in EtOAc are well resolved and can be modelled by a simulation of the spin Hamiltonian.[43] In the following the simulated spectra are plotted in red over the respective measurements.

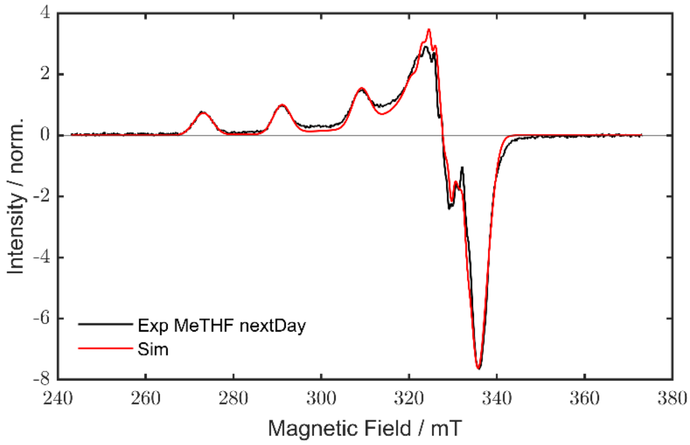

Figure S9: X-Band EPR spectrum of the catalyst in frozen solution of Me-THF (110 K) with a spin Hamiltonian simulation based on a Cu(II) center with additional hyperfines on the coordinating nitrogen atoms.

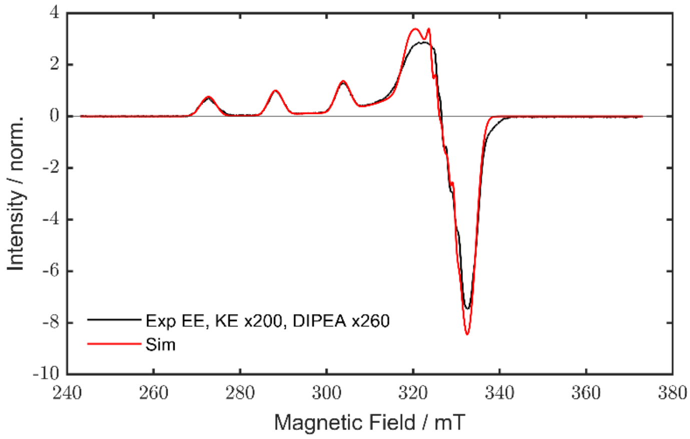

Figure S10: X-Band EPR spectrum of the catalyst with KE (**1a**) and DIPEA in frozen solution of EtOAc (110 K) with a spin Hamiltonian simulation based on a Cu(II) center with additional hyperfines on the coordinating nitrogen atoms.

The parameters for the simulations of both systems are shown in the following tables.

Table S4: Simulation parameters for X-Band EPR of the catalyst in Me-THF after 24 h. The nitrogen hyperfine values are set constant based on calculations on an optimized geometry of the catalyst.

Parameter	Value		
g_x, g_y, g_z	2.040(1)	2.066(1)	2.242(1)
Cu: A_x, A_y, A_z [MHz]	45(2)	47(2)	552(2)
AStrain [MHz]	70(5)	20(3)	0
gStrain	0.0011(1)	0.011(1)	0.025(2)
Gaussian LW /mT		0.65(2)	

Table S5: Simulation parameters for X-Band EPR of the catalyst in EtOAc after 24 h. The nitrogen hyperfine values are set constant based on calculations on an optimized geometry of the catalyst.

Parameter	Value		
g_x, g_y, g_z	2.061(1)	2.061(1)	2.274(1)
Cu: A_x, A_y, A_z [MHz]	45(2)	47(2)	485(2)
AStrain [MHz]	70(5)	20(3)	0
gStrain	0.0033(1)	0.026(1)	0.015(2)
Gaussian LW /mT		0.68(2)	

10 Kinetic Investigations

10.1 Hammett Analysis

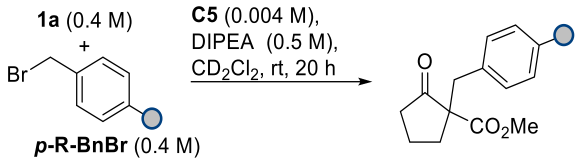

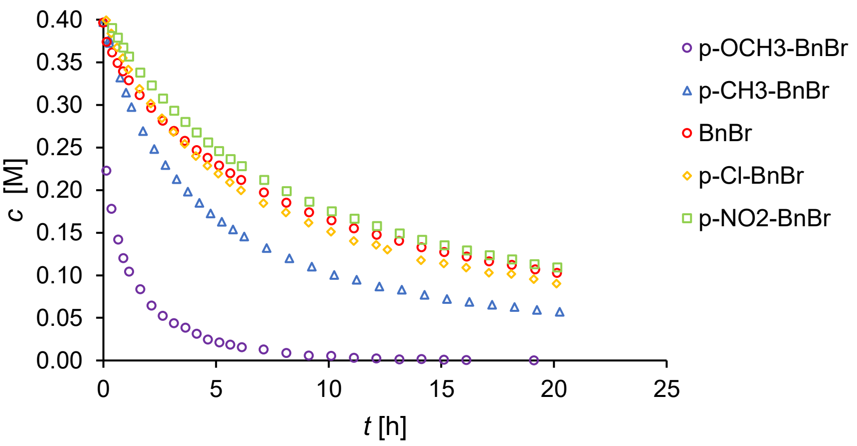

Figure S11: Influence of different *para*-substituents of the respective benzyl bromide on reaction rate. The reactions were conducted using c_0(**1a**) = 0.4 M, c_0(***p*-R-BnBr**) = 0.4 M, c_0(DIPEA) = 0.5 M, c_0(**C5**) = 0.004 M.

Linearization of concentration profiles: second order rate law (A = respective benzyl bromide)

$$[\mathrm{A}] = \frac{1}{\frac{1}{[\mathrm{A}]_0}} - \nu_{\mathrm{A}} k t \qquad (1)$$

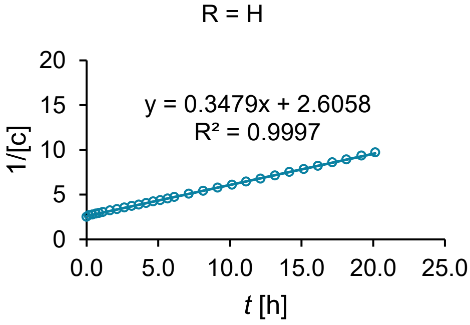

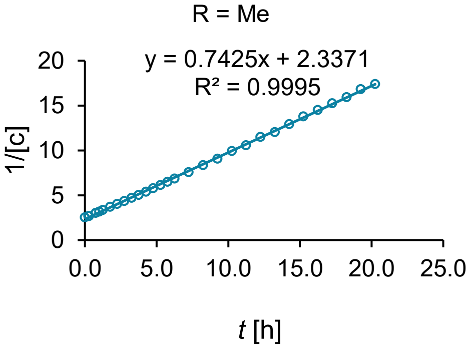

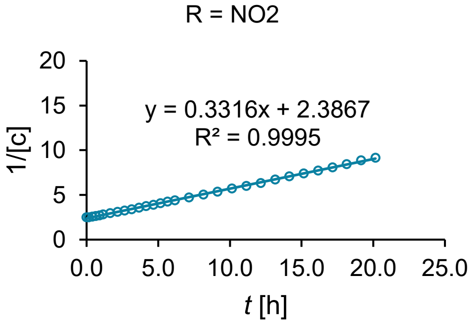

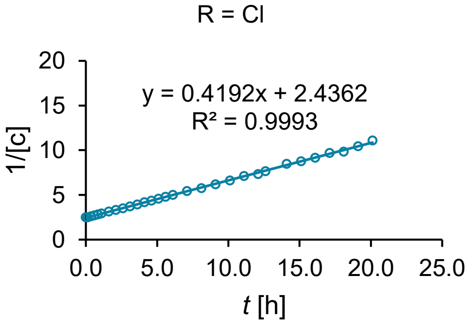

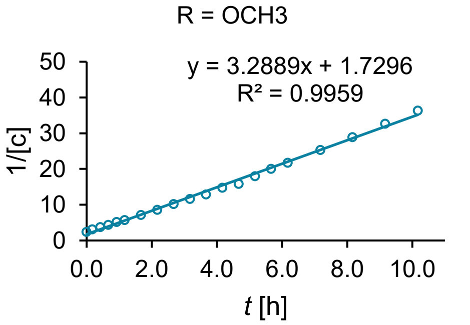

R	k $(\mathrm{mol/l{\cdot}h})^{-1}$	$\log(k/k_0)$	σ_p^+
**H**	0.3479	0	0
**Me**	0.7425	0.329188	-0.31
**NO_2**	0.3316	-0.02082	0.79
**Cl**	0.4192	0.080919	0.11
**OCH_3**	3.2889	0.975574	-0.78

Figure S12: Linearization of concentration profiles according to a second order rate law for the corresponding *para*-substituent.

10.2 Rate Law by VTNA Analysis

Variable Time Normalization Analysis (VTNA) was used to determine reaction orders. Following *Burés'* method, concentration profiles with varying amounts of a component align when the time axis is replaced by the integral of concentration raised to the order α, calculated via the trapezoid rule in Equation (2).[44–46]

$$\int_{t=0}^{t=n} [\mathrm{A}]^{\alpha} \mathrm{d}t = \sum_{i=1}^{n} \left(\frac{[\mathrm{A}]_i + [\mathrm{A}]_{i-1}}{2} \right)^{\alpha} (t_i - t_{i-1}) \tag{2}$$

As the catalyst concentration remains constant during the catalytic cycle, the normalized time scale for the catalyst is determined using the initial concentration of the catalyst $[\mathrm{cat}]_0$ (3).

$$\sum_{i=1}^{n} (cat)^{\alpha} (t_i - t_{i-1}) = t[cat]_0^{\alpha} \tag{3}$$

The starting concentrations of the respective experiments are given in the following table.

Table S6: Variation of initial concentration of each reaction component **B1**-**B7**. The experiments were carried out in borosilicate glass NMR tubes with screw caps in dry CD_2Cl_2 and at room temperature. The reaction was monitored using ^{1}H-NMR spectroscopy (400 MHz) with 10 number of scans and an interscan delay of 1 s.

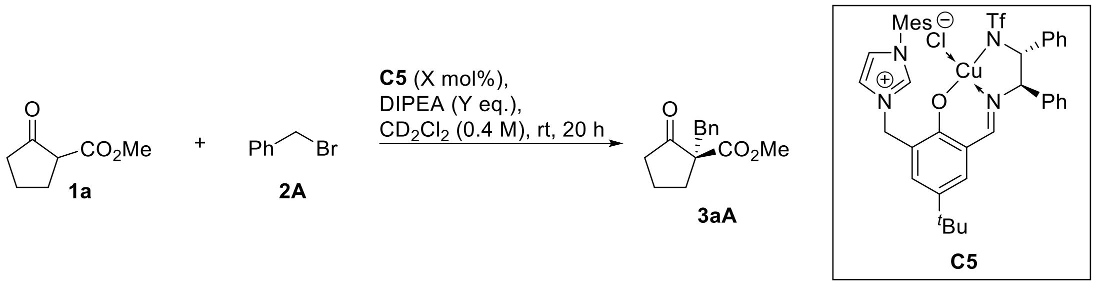

#	Varied parameter	Initial concentration *c* [M]					
		**1a**	**DIPEA**	**2A**	**C5**	**3aA**	**DIPEA · HBr**
**B1**	Standard	0.4	0.5	0.4	0.004	0.0	0.0
**B2**	c_0(**1a**)	0.6	0.5	0.4	0.004	0.0	0.0
**B3**	c_0(**2A**)	0.4	0.5	0.6	0.004	0.0	0.0
**B4**	c_0(**DIPEA**)	0.4	1.2	0.4	0.004	0.0	0.0
**B5**	c_0(**C5**)	0.4	0.5	0.4	0.008	0.0	0.0
**B6**	c_0(**DIPEA · HBr**)	0.4	0.5	0.4	0.004	0.0	0.2
**B7**	c_0(**3aA**)	0.4	0.5	0.4	0.004	0.2	0.0

The concentration profiles of experiments **B2-B7** compared to standard experiment **B1** are shown in Figure S13.

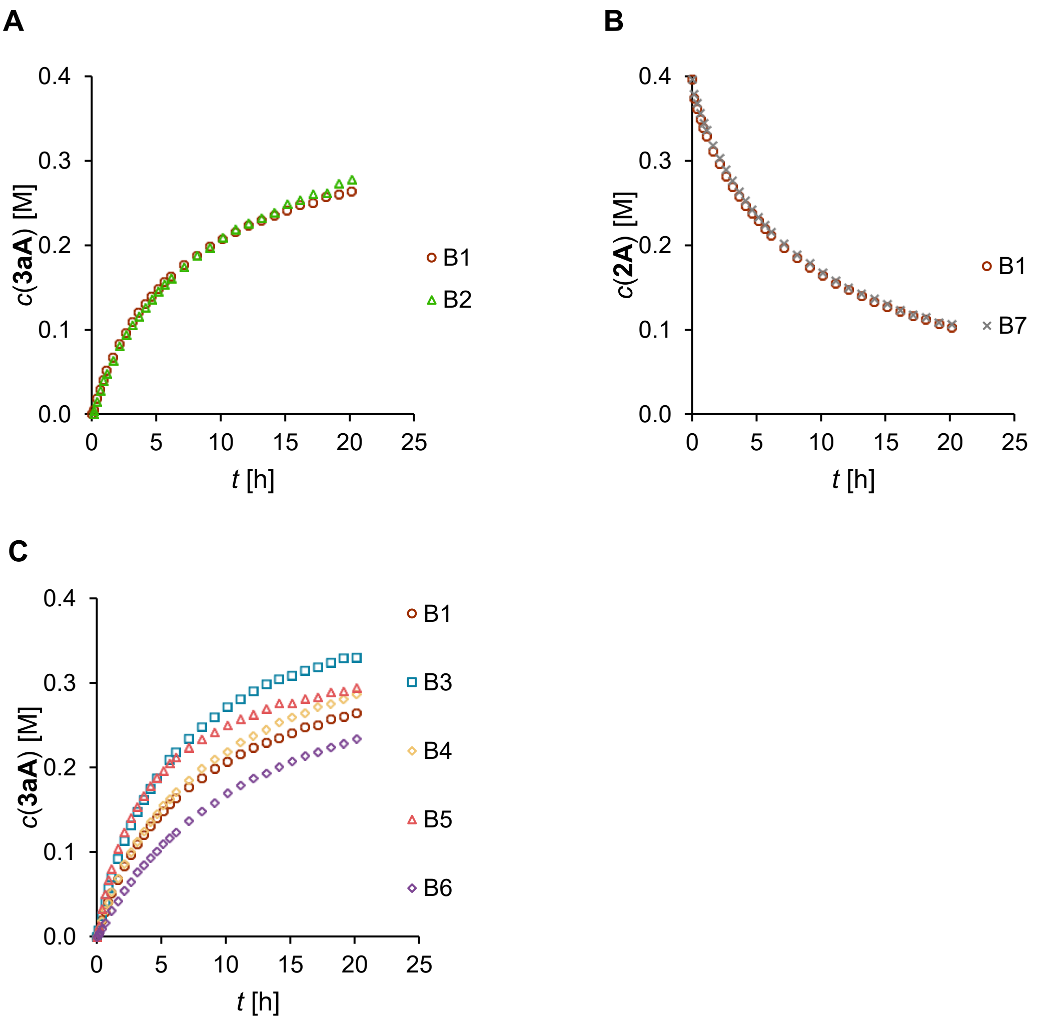

Figure S13: Concentration profiles of experiments **B2**-**B7** compared to the standard experiment **B1** monitored by ^{1}H-NMR spectroscopy. **A:** Comparison of **B1** and **B2** shows no influence of *c*(**1a**) on the product formation rate. **B:** Comparison of **B1** and **B7** shows no influence of *c*(**3aA**) on the reaction rate. **C:** All other varied starting concentrations **B3**-**B6** ([**2A**], [**DIPEA**], [**C5**], and [**DIPEA · HBr**]) showed deviating curves from the standard experiment, therefore influencing the reaction rate.

Since not all reaction components could be monitored by ^{1}H-NMR spectroscopy of the reaction mixture, the following assumptions were made to estimate the time-dependent concentrations. The concentration of DIPEA was determined by subtracting the amount of formed alkylated β-ketoester from the initial concentration (4). The concentration of DIPEA·HBr was calculated by adding the amount of alkylation product formed to the initial concentration (5).

$$[\mathrm{DIPEA}] = [\mathrm{DIPEA}]_0 - [3\mathrm{aA}] \tag{4}$$

$$[\mathrm{DIPEAHBr}] = [\mathrm{DIPEA}\cdot\ \mathrm{HBr}]_0 + [3\mathrm{aA}] \tag{5}$$

The concentration profiles with the normalized time scales for experiments **B3-B6** are depicted in Figure S14.

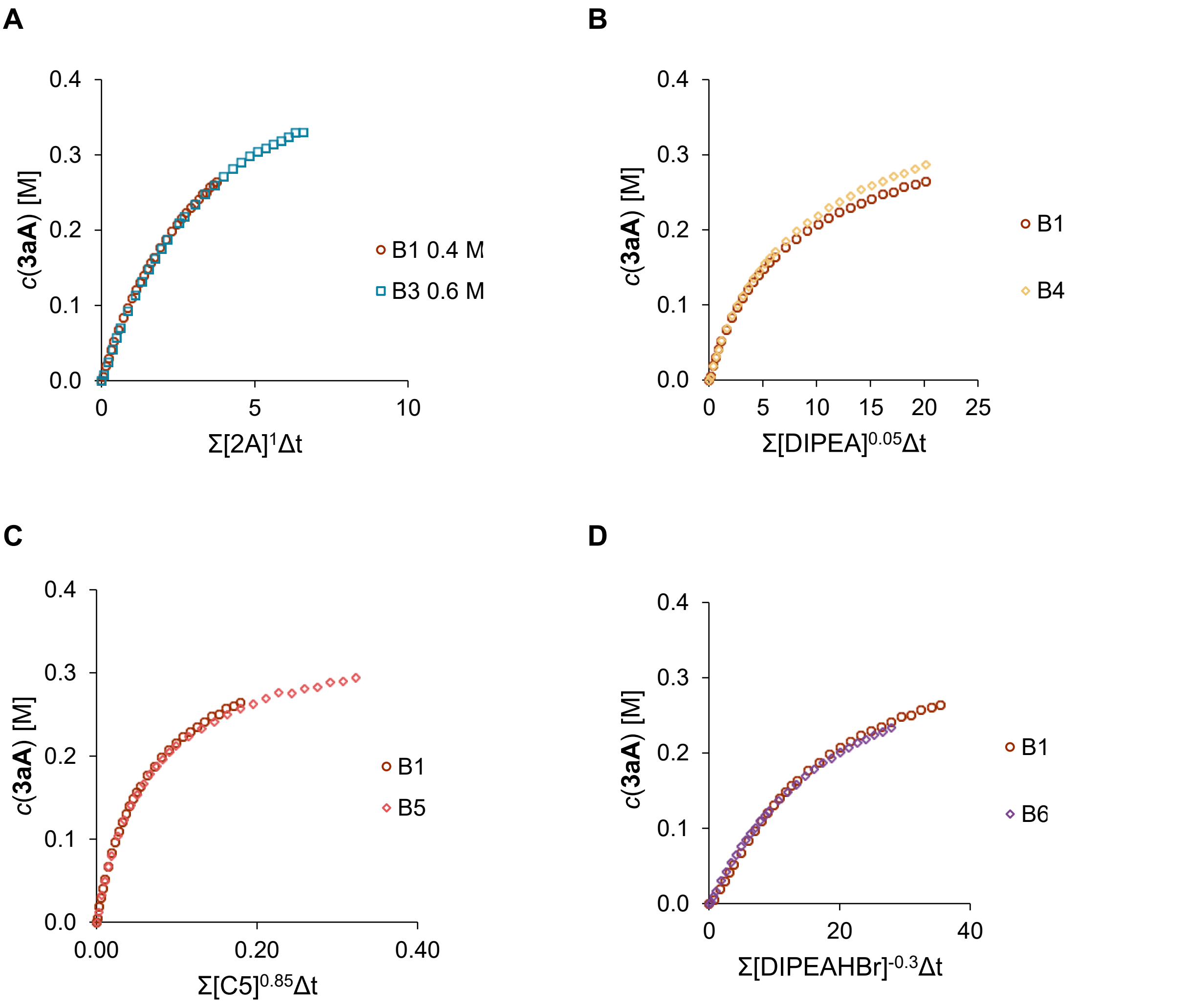

Figure S14: Variable time normalization analysis to determine the order of each component. **A:** order of **2A** (1). **B:** order of DIPEA (0.05). **C:** order of **C5** (0.85). **D:** order of DIPEA · HBr (-0.3).

Sequential normalization of the time scale was applied to linearize the concentration profiles using data points up to <50% conversion.

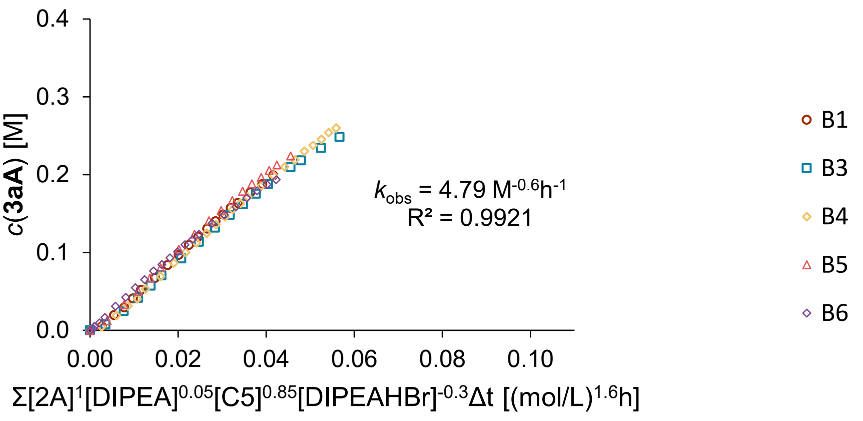

Figure S15: Sequential normalization of the time scale.

The following rate law was derived from the VTNA analysis (6).

$$\frac{d[3aA]}{d[t]} = 4.79\ \mathrm{M}^{-0.6}\mathrm{h}^{-1}[2A]^{1}[DIPEA]^{0.05}[C5]^{0.85}[DIPEA \cdot HBr]^{-0.3} \qquad (6)$$

Inhibiting Effect of DIPEA · HBr

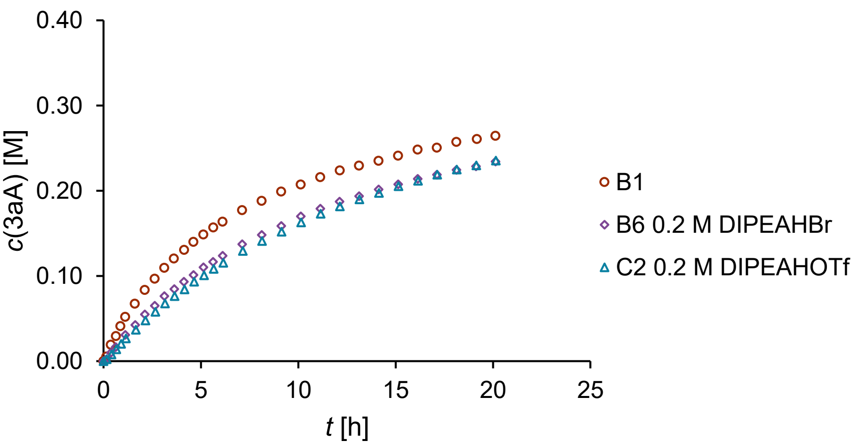

Figure S16: Influence of different DIPEA · HX salts on reaction rate. The reactions were conducted using c_0(KE) = 0.4 M, c_0(BnBr) = 0.4 M, c_0(DIPEA) = 0.5 M, c_0(cat) = 0.004 M and c_0(DIPEA · HX) = 0.2 M. **B1** (red curve) represents the standard experiment where no additional salt is added. Addition of DIPEA · HX salts (X = Br, OTf) slowed down the reaction rate.

Measured Concentration Profiles

Table S7: Experiment B1.

t [h]	[**1a**] [M]	[**2A**] [M]	[**3aA**] [M]
0.00	0.4005	0.3964	0.0000
0.14	0.3691	0.3738	0.0050
0.37	0.3554	0.3615	0.0191
0.62	0.3447	0.3493	0.0293
0.86	0.3336	0.3392	0.0409
1.12	0.3226	0.3290	0.0517
1.62	0.3070	0.3115	0.0672
2.12	0.2913	0.2963	0.0833
2.61	0.2783	0.2814	0.0965
3.12	0.2650	0.2693	0.1093
3.61	0.2532	0.2579	0.1203
4.12	0.2430	0.2467	0.1305
4.61	0.2347	0.2377	0.1398
5.12	0.2263	0.2287	0.1485
5.61	0.2170	0.2198	0.1567
6.12	0.2097	0.2117	0.1635
7.11	0.1956	0.1973	0.1770
8.12	0.1855	0.1852	0.1878
9.11	0.1726	0.1740	0.1988
10.12	0.1669	0.1646	0.2072
11.11	0.1553	0.1550	0.2157
12.11	0.1481	0.1477	0.2235
13.12	0.1418	0.1406	0.2295
14.12	0.1376	0.1331	0.2351
15.11	0.1306	0.1273	0.2411
16.11	0.1241	0.1220	0.2479
17.12	0.1198	0.1163	0.2504
18.13	0.1186	0.1123	0.2572
19.18	0.1117	0.1070	0.2605
20.13	0.1072	0.1030	0.2643

Table S8: Experiment B2.

t [h]	[**1a**] [M]	[**2A**] [M]	[**3aA**] [M]
0.00	0.5853	0.3964	0.0000
0.16	0.5681	0.3708	0.0042
0.40	0.5581	0.3598	0.0154
0.65	0.5445	0.3479	0.0280
0.90	0.5330	0.3377	0.0398
1.15	0.5245	0.3274	0.0484
1.65	0.5081	0.3092	0.0637
2.15	0.4915	0.2944	0.0809
2.66	0.4778	0.2795	0.0941
3.15	0.4663	0.2675	0.1059
3.65	0.4531	0.2536	0.1161
4.15	0.4441	0.2444	0.1267
4.65	0.4332	0.2338	0.1364
5.15	0.4228	0.2237	0.1458
5.65	0.4129	0.2146	0.1541
6.15	0.4052	0.2051	0.1610
7.15	0.3920	0.1903	0.1742
8.15	0.3784	0.1786	0.1879
9.15	0.3681	0.1656	0.1973
10.15	0.3560	0.1554	0.2092
11.15	0.3458	0.1454	0.2192
12.15	0.3382	0.1365	0.2261
13.15	0.3354	0.1299	0.2325
14.15	0.3276	0.1210	0.2389
15.15	0.3185	0.1160	0.2490
16.15	0.3126	0.1087	0.2537
17.15	0.3062	0.1030	0.2607
18.23	0.3058	0.0970	0.2618
19.15	0.2968	0.0946	0.2731
20.15	0.2954	0.0918	0.2780

Table S9: Experiment B3.

t [h]	[**1a**] [M]	[**2A**] [M]	[**3aA**] [M]
0	0.4005	0.5832	0.0000
0.13	0.3687	0.5716	0.0075
0.38	0.3518	0.5536	0.0245
0.63	0.3351	0.5377	0.0415
0.88	0.3182	0.5215	0.0572
1.13	0.3055	0.5080	0.0701
1.63	0.2830	0.4844	0.0923
2.13	0.2622	0.4633	0.1136
2.63	0.2428	0.4438	0.1316
3.13	0.2259	0.4263	0.1480
3.63	0.2115	0.4112	0.1623
4.13	0.1982	0.3974	0.1753
4.63	0.1852	0.3842	0.1878
5.63	0.1679	0.3642	0.2096
6.13	0.1572	0.3529	0.2182
7.13	0.1394	0.3345	0.2343
8.13	0.1243	0.3186	0.2480
9.13	0.1123	0.3052	0.2595
10.13	0.1031	0.2950	0.2718
11.13	0.0921	0.2841	0.2813
12.13	0.0857	0.2762	0.2902
13.13	0.0793	0.2691	0.2987
14.13	0.0741	0.2620	0.3045
15.13	0.0654	0.2541	0.3090
16.13	0.0605	0.2485	0.3146
17.13	0.0560	0.2438	0.3190
18.13	0.0506	0.2398	0.3241
19.14	0.0496	0.2373	0.3297
20.13	0.0482	0.2333	0.3303

Table S10: Experiment B4.

t [h]	[**1a**] [M]	[**2A**] [M]	[**3aA**] [M]
0.00	0.4005	0.3964	0.0000
0.12	0.3611	0.3857	0.0044
0.37	0.3473	0.3727	0.0187
0.64	0.3344	0.3589	0.0313
0.88	0.3242	0.3477	0.0404
1.13	0.3123	0.3377	0.0530
1.63	0.2966	0.3203	0.0693
2.13	0.2790	0.3037	0.0862
2.63	0.2647	0.2885	0.1002
3.13	0.2518	0.2749	0.1125
3.64	0.2402	0.2627	0.1247
4.15	0.2296	0.2510	0.1359
4.65	0.2193	0.2402	0.1456
5.13	0.2110	0.2315	0.1552
5.64	0.2020	0.2212	0.1628
6.13	0.1955	0.2137	0.1713
7.12	0.1802	0.1977	0.1850
8.12	0.1677	0.1844	0.1989
9.11	0.1556	0.1718	0.2099
10.11	0.1457	0.1596	0.2190
11.11	0.1358	0.1502	0.2301
12.11	0.1272	0.1404	0.2376
13.12	0.1202	0.1323	0.2454
14.12	0.1121	0.1251	0.2540
15.12	0.1087	0.1186	0.2595
16.12	0.1011	0.1114	0.2648
17.12	0.0949	0.1062	0.2719
18.12	0.0907	0.0995	0.2756
19.11	0.0857	0.0950	0.2813
20.12	0.0819	0.0917	0.2870

Table S11: Experiment B5.

t [h]	[**1a**] [M]	[**2A**] [M]	[**3aA**] [M]
0	0.4005	0.3964	0.0000
0.15	0.3498	0.3450	0.0124
0.38	0.3293	0.3248	0.0328
0.63	0.3129	0.3070	0.0498
0.88	0.2946	0.2899	0.0672
1.13	0.2839	0.2771	0.0801
1.63	0.2596	0.2528	0.1040
2.13	0.2424	0.2334	0.1233
2.64	0.2233	0.2147	0.1408
3.13	0.2095	0.2001	0.1538
3.63	0.1980	0.1874	0.1668
4.14	0.1859	0.1754	0.1786
4.63	0.1783	0.1660	0.1881
5.14	0.1705	0.1561	0.1963
5.63	0.1605	0.1477	0.2052
6.13	0.1519	0.1395	0.2124
7.13	0.1422	0.1270	0.2239
8.13	0.1298	0.1143	0.2335
9.13	0.1214	0.1045	0.2418
10.13	0.1143	0.0966	0.2502
11.13	0.1046	0.0897	0.2578
12.13	0.1003	0.0834	0.2630
13.13	0.0965	0.0775	0.2695
14.13	0.0930	0.0751	0.2766
15.13	0.0878	0.0680	0.2760
16.14	0.0838	0.0646	0.2816
17.13	0.0828	0.0607	0.2833
18.13	0.0761	0.0579	0.2891
19.13	0.0753	0.0542	0.2903
20.15	0.0698	0.0515	0.2944

Table S12: Experiment B6.

t [h]	[**1a**] [M]	[**2A**] [M]	[**3aA**] [M]
0.00	0.4005	0.3964	0.0000
0.14	0.3774	0.3850	0.0018
0.39	0.3703	0.3771	0.0095
0.63	0.3634	0.3697	0.0163
0.17	0.3746	0.3837	0.0047
1.13	0.3485	0.3556	0.0309
1.63	0.3366	0.3432	0.0423
2.13	0.3238	0.3312	0.0547
2.63	0.3126	0.3194	0.0648
3.13	0.3020	0.3093	0.0763
3.63	0.2942	0.2998	0.0845
4.13	0.2853	0.2902	0.0931
4.63	0.2777	0.2817	0.1009
5.13	0.2670	0.2722	0.1099
5.63	0.2605	0.2644	0.1166
6.13	0.2532	0.2564	0.1235
7.13	0.2401	0.2433	0.1373
8.13	0.2295	0.2309	0.1481
9.13	0.2178	0.2193	0.1585
10.13	0.2095	0.2104	0.1700
11.13	0.2038	0.2026	0.1791
12.13	0.1940	0.1932	0.1871
13.14	0.1858	0.1836	0.1935
14.13	0.1774	0.1761	0.2013
15.13	0.1683	0.1677	0.2074
16.14	0.1622	0.1616	0.2139
17.13	0.1583	0.1554	0.2186
18.13	0.1529	0.1501	0.2243
19.13	0.1483	0.1446	0.2285
20.13	0.1427	0.1400	0.2342

Table S13: Experiment B7.

t [h]	[**1a**] [M]	[**2A**] [M]	[**3aA**] [M]
0.00	0.4005	0.3964	0.1656
0.13	0.3690	0.3792	0.1756
0.36	0.3586	0.3682	0.1870
0.61	0.3467	0.3567	0.1984
0.89	0.3360	0.3447	0.2084
1.11	0.3273	0.3362	0.2178
1.61	0.3085	0.3183	0.2355
2.11	0.2953	0.3035	0.2495
2.61	0.2824	0.2896	0.2628
3.12	0.2699	0.2765	0.2747
3.63	0.2566	0.2636	0.2861
4.13	0.2457	0.2529	0.2976
4.61	0.2360	0.2424	0.3065
5.12	0.2270	0.2332	0.3152
5.61	0.2187	0.2243	0.3228
6.11	0.2112	0.2162	0.3297
7.11	0.1971	0.2020	0.3450
8.12	0.1841	0.1888	0.3565
9.11	0.1749	0.1790	0.3680
10.11	0.1653	0.1674	0.3757
11.12	0.1551	0.1582	0.3855
12.11	0.1491	0.1497	0.3908
13.12	0.1407	0.1425	0.3996
14.12	0.1384	0.1371	0.4058
15.11	0.1300	0.1307	0.4134
16.12	0.1220	0.1231	0.4179
17.11	0.1190	0.1179	0.4215
18.11	0.1172	0.1147	0.4266
19.12	0.1110	0.1089	0.4317
20.12	0.1090	0.1067	0.4378

Table S14: Experiment C2.

t [h]	[**1a**] [M]	[**2A**] [M]	[**3aA**] [M]
0.00	0.4005	0.3964	0
0.17	0.3699	0.3812	0.0022
0.41	0.3644	0.3750	0.0079
0.66	0.3587	0.3692	0.0140
0.91	0.3530	0.3633	0.0204
1.16	0.3467	0.3568	0.0266
1.67	0.3366	0.3462	0.0367
2.16	0.3246	0.3343	0.0475
2.66	0.3128	0.3227	0.0578
3.17	0.3039	0.3138	0.0675
3.66	0.2931	0.3032	0.0762
4.16	0.2846	0.2938	0.0842
4.67	0.2757	0.2852	0.0931
5.16	0.2681	0.2769	0.1008
5.66	0.2623	0.2704	0.1080
6.16	0.2543	0.2624	0.1154
7.17	0.2409	0.2495	0.1294
8.15	0.2302	0.2392	0.1409
9.15	0.2186	0.2285	0.1520
10.15	0.2057	0.2169	0.1628
11.15	0.1954	0.2066	0.1729
12.15	0.1861	0.1961	0.1814
13.15	0.1791	0.1886	0.1897
14.14	0.1736	0.1816	0.1973
15.15	0.1635	0.1732	0.2052
16.15	0.1561	0.1653	0.2118
17.15	0.1490	0.1591	0.2188
18.15	0.1430	0.1525	0.2248
19.14	0.1382	0.1476	0.2296
20.15	0.1334	0.1422	0.2353

11 Computational Studies

11.1 Computational Details

All structures relevant to the reaction mechanism were first built manually and explored in their conformational space using metadynamics simulations, based on tight-binding quantum chemical calculations as implemented in CREST.[47,48] All minimum structures and transition states were pre-optimized using GFN2-xTB[49] with the Turbomole V7.8.1 program package[50,51] and subsequently re-optimized at the DFT level using the PBEh[52]-3c functional including the Becke-Johnson damping scheme D3(BJ)[53,54] and the def2-mSVP[52] basis set. The verification of the optimized structures as local minima was carried out by analytical frequency analysis. The electronic energies for all obtained geometries were calculated using the B3LYP[55–57] functional with D3(BJ)[53,54] dispersion correction and the def2-TZVP[58,59] basis set. The free energy G was calculated at 298.15 K within the rigid rotor harmonic oscillator (RRHO) approximation at a reference state of 1 mol/L. Solvent effects were treated with the conductor-like screening model for real solvents (COSMO-RS)[60], where the dielectric constant was set to $\varepsilon = 8.93$ for ethyl acetate (experimental conditions) and $\varepsilon = 6.02$ for DCM (NMR reaction monitoring conditions). The DCOSMO-RS,[60] model was employed as it is implemented in the Turbomole V7.8.1 program package,[50,51] and the standard potential file for pure solvents was used. Transition state structures were searched for using bond length scans or the nudged elastic band (NEB)[61] method, and possible transition state structures were then subsequently investigated via the dimer method[62,63]. The nature of the found transition states was verified by confirming that they possess only a single mode with an imaginary frequency. For the NCI Analysis we used the NCIPlot 4.3 software[64,65] VMD 19.3[66] as well as gnuplot 6.0[67] for visualization purposes.

An additional xyz-file has been provided, which contains Cartesian coordinates for all calculated species.

11.2 Keto-Enol Tautomerism and Background Reaction

Keto-enol tautomerism of the β-ketoester was investigated to assess whether a sufficient quantity of the enol form can be formed under the reaction conditions (Figure S17, blue). We calculated the mechanism with DIPEA acting as the catalytic base for both solvents EtOAc and DCM. Depending on the solvent the energetic difference between the keto and enol form was calculated to be 7.0 kJ/mol in EtOAc and 8.0 kJ/mol in DCM, which corresponds to a ratio of about 94% keto- and 6% enol-tautomer. Furthermore, the barrier of the initial deprotonation of

the keto form was determined to be $\Delta G^{\ddagger}_{\text{keto-enol,EtOAc}}$ = 75.0 kJ mol^{-1} in EtOAc and $\Delta G^{\ddagger}_{\text{keto-enol,DCM}}$ = 88.7 kJ mol^{-1} in DCM. On this basis, a continuous formation of the enol tautomer *via* the keto-enol equilibrium is assumed under the reaction conditions.

Additionally, the uncatalyzed background reaction leading to racemic product formation was investigated to enable direct comparison with the catalytic pathway (Figure S17, red). For the background reaction, the activation barrier of the rate-determining C-C bond formation is significantly higher ($\Delta G^{\ddagger}_{\text{background}}$ = 105.9 kJ mol^{-1}) than that of the corresponding step in the catalytic mechanism ($\Delta G^{\ddagger}_{\text{cat}}$ = 58.3 kJ mol^{-1}), indicating that this transformation is disfavored by 47.6 kJ mol^{-1} relative to the catalyzed pathway.

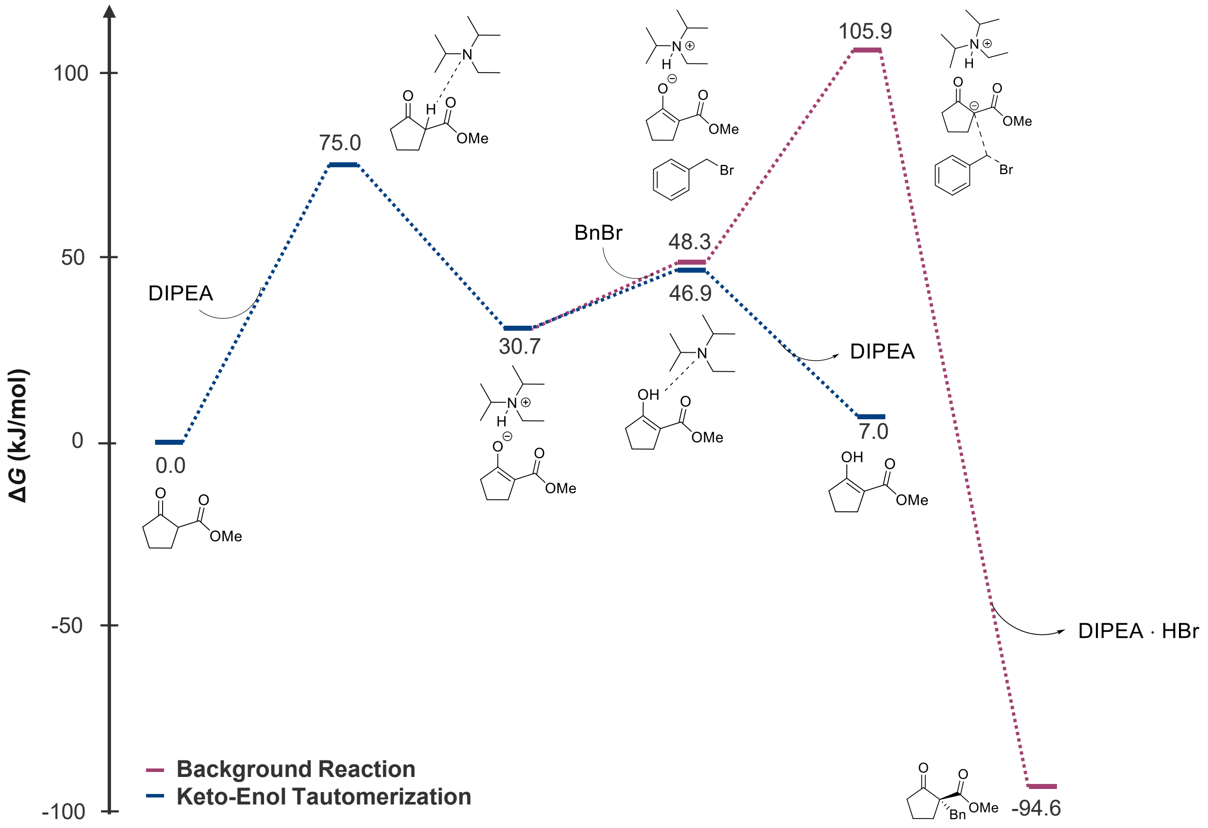

Figure S17: Free energy profile of the keto-enol tautomerization (blue) and the background reaction, i.e., the alkylation of the β-ketoester without any catalyst (red) in EtOAc. The deprotonation of the β-ketoester is promoted by DIPEA in both cases. The keto form was set as the reference (ΔG = 0 kJ mol^{-1}).

11.3 Mechanistic Discussions on the Asymmetric Catalytic Reaction Mechanism

Figure S18 provides an overview of the investigated reaction mechanism for the major (blue) and minor (red) product channels. The structures of all intermediates and transition states are

shown for the major pathway. The major channel proceeds with an overall barrier (energetic span) of $\Delta G^{\ddagger}_{major}$ = 58.3 kJ mol^{-1}, whereas the barrier for the minor channel is significantly higher, $\Delta G^{\ddagger}_{minor}$ = 72.1 kJ mol^{-1}.

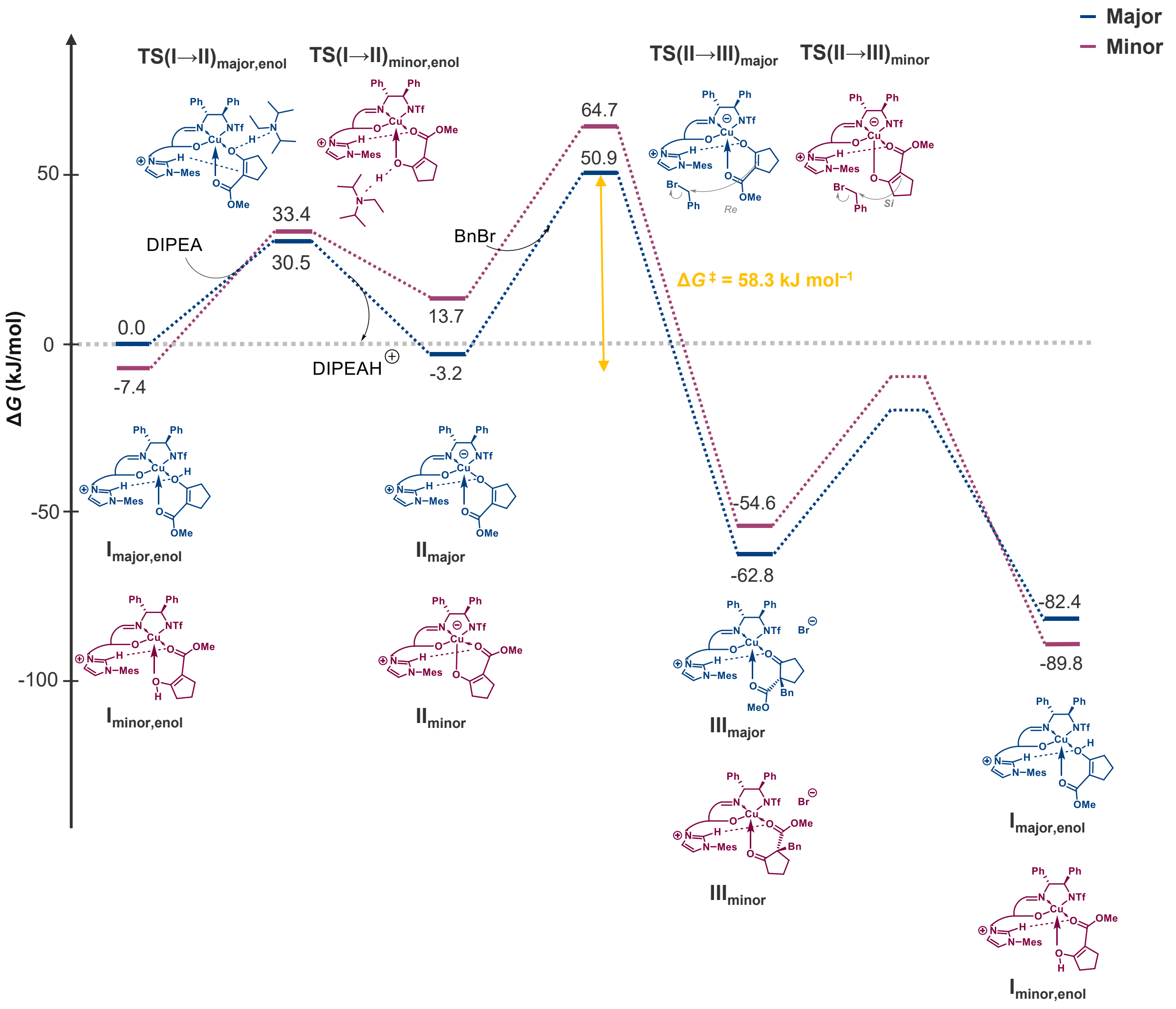

Figure S18: Free energy profile of the investigated reaction mechanism leading to the major enantiomer (blue) and minor enantiomer (purple) using DCM as solvent. The energetic span of the reaction ($\Delta G^{\ddagger}_{major}$ = 58.3 kJ mol^{-1}) is depicted in yellow. Similar to Figure 3C in the main text.

In the following sections a mechanistic analysis of the reaction pathway is provided, including a step-by-step description of all elementary reactions and a detailed characterization of the corresponding intermediates and transition-state structures.

11.3.1 Initial Coordination of the Substrate

To identify the most favorable binding mode, a range of coordination motifs of the β-ketoester were systematically investigated, including both keto and enol tautomers as well as mono- and bidentate binding modes. Among the conformers examined, the four structures shown were

determined to be the energetically most relevant (Figure S19) and no additional structures of comparable stability were identified. In structures $\mathbf{I}_{\mathbf{major,keto}}$ and $\mathbf{I}_{\mathbf{major,enol}}$ of the major reaction channel, the keto or enol oxygen respectively is bound in the equatorial position while the ester group is in axial position. In contrast, the minor channel structures $\mathbf{I}_{\mathbf{minor,keto}}$ and $\mathbf{I}_{\mathbf{minor,enol}}$ exhibit the reversed binding mode, the ester oxygen thus adopting the equatorial position.

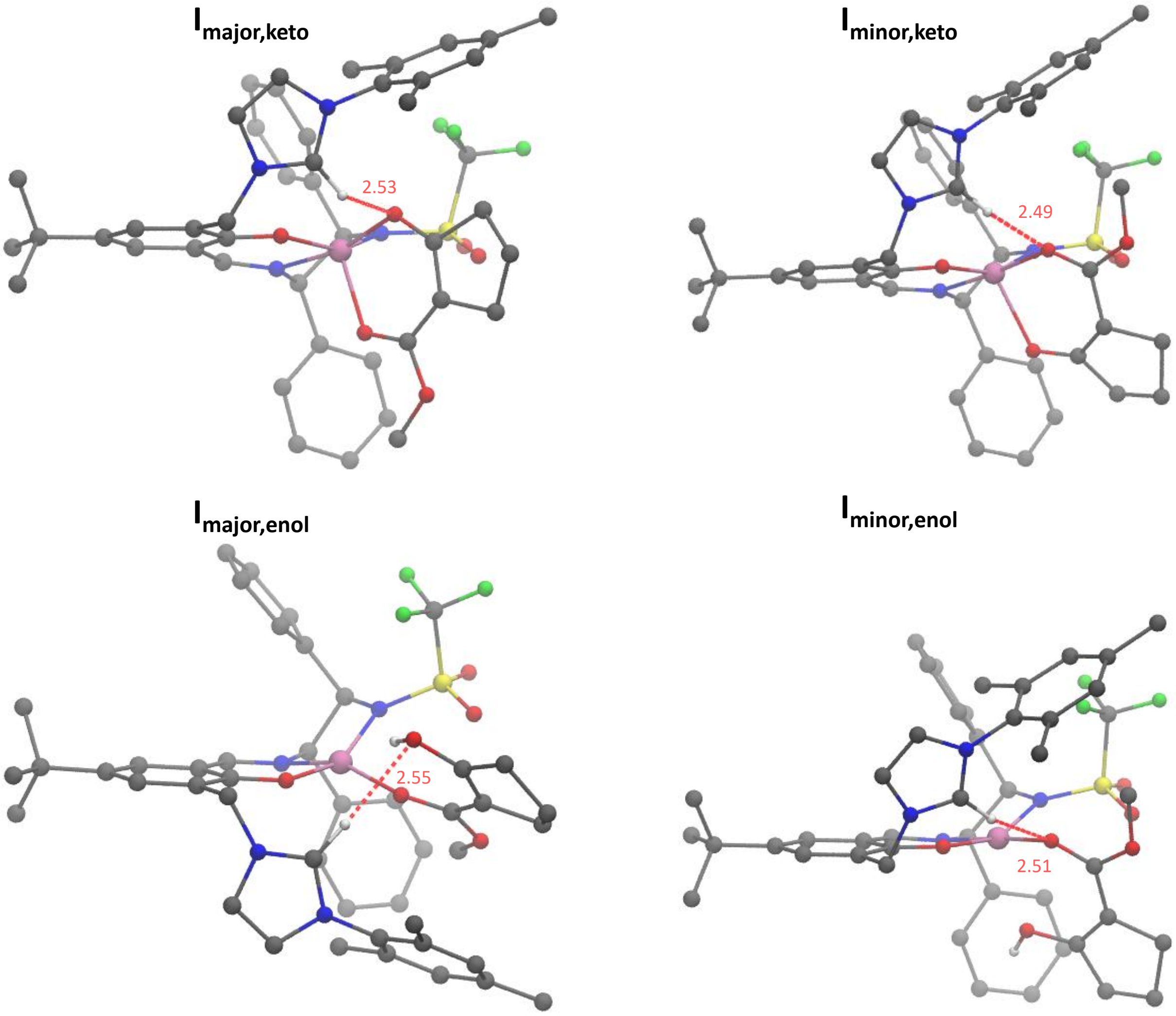

Figure S19: Initial coordination modes of the β-ketoester for the major and minor product channels, shown for both the keto ($\mathbf{I}_{\mathbf{major,keto}}$ and $\mathbf{I}_{\mathbf{minor,keto}}$) and enol ($\mathbf{I}_{\mathbf{major,enol}}$ and $\mathbf{I}_{\mathbf{minor,enol}}$) tautomers. The interaction between the imidazolium C(2)-H and the β-ketoester is indicated (Å). White: H, grey: C, red: O, blue: N, yellow: S, green: F, and pink: Cu. Non-relevant hydrogens are omitted for visual clarity.

11.3.2 Deprotonation Step: Evaluation of Possible Deprotonation Pathways

The deprotonation step was investigated for both the major and minor reaction channels, considering the coordinated β-ketoester in its keto ($\mathbf{I}_{\mathbf{major,keto}}$ and $\mathbf{I}_{\mathbf{minor,keto}}$) and enol ($\mathbf{I}_{\mathbf{major,enol}}$ and $\mathbf{I}_{\mathbf{minor,enol}}$) forms (Figure S20). For the major channel *via* the keto form, the DFT-calculated

deprotonation barrier was determined to be $\Delta G^{\ddagger}_{TS,major,keto}$ = 95.6 kJ mol^{-1}. In contrast, the deprotonation barrier of the corresponding enol complex **I**$_{\mathbf{major,enol}}$ was found to be $\Delta G^{\ddagger}_{TS,major,enol}$ = 30.5 kJ mol^{-1}. The difference of both transition state energies was calculated to be 34.0 kJ/mol favoring the enol path. A similar trend was observed for the deprotonation step of the minor channels, with barriers of $\Delta G^{\ddagger}_{TS,minor,keto}$ = 82.9 kJ mol^{-1} and $\Delta G^{\ddagger}_{TS,minor,enol}$ = 40.8 kJ mol^{-1} for the keto and enol complexes, respectively.

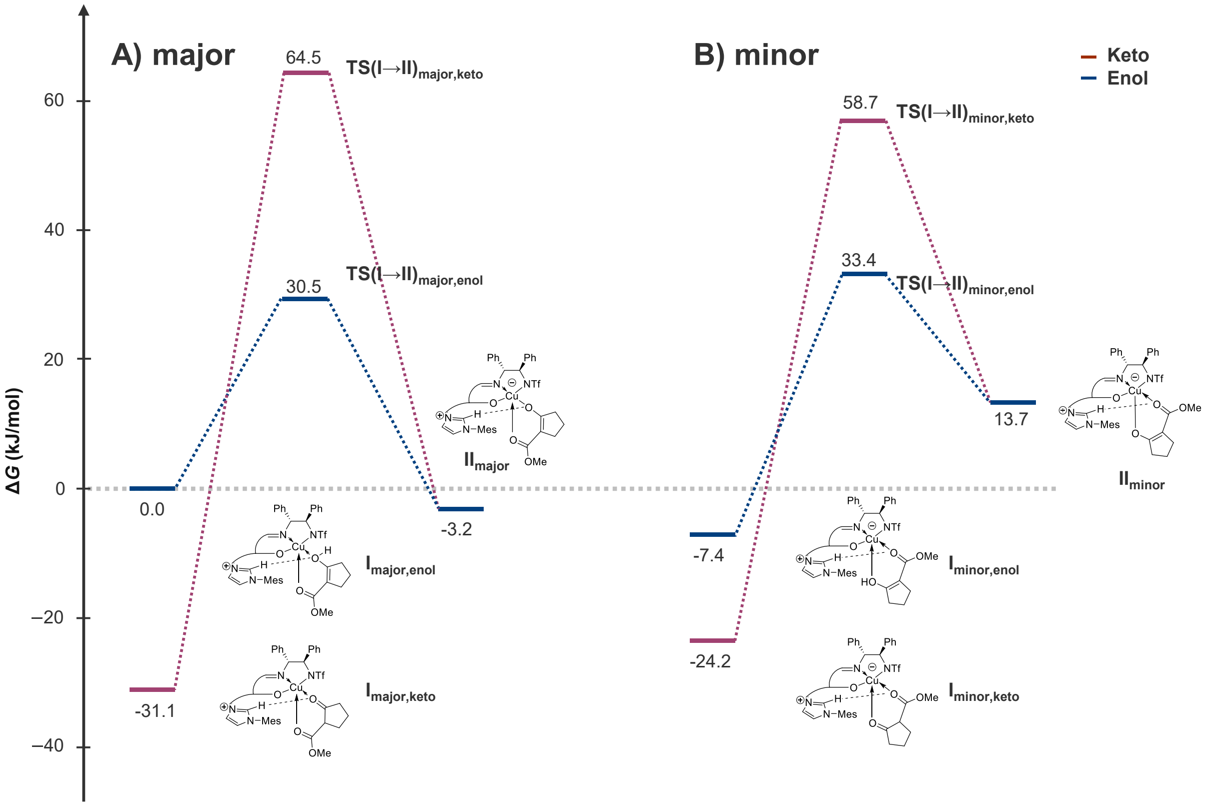

Figure S20: Free energy profile of the deprotonation step for the A) major and B) minor product channels, shown for both the keto (red) and enol tautomers (blue) using DCM as solvent.

The substantial energetic difference can at least be partly attributed to the different steric accessibility of the respective hydrogens (**TS(I→II)**$_{\mathbf{major/minor,keto}}$ vs. **TS(I→II)**$_{\mathbf{major/minor,enol}}$) (see Figure S21).

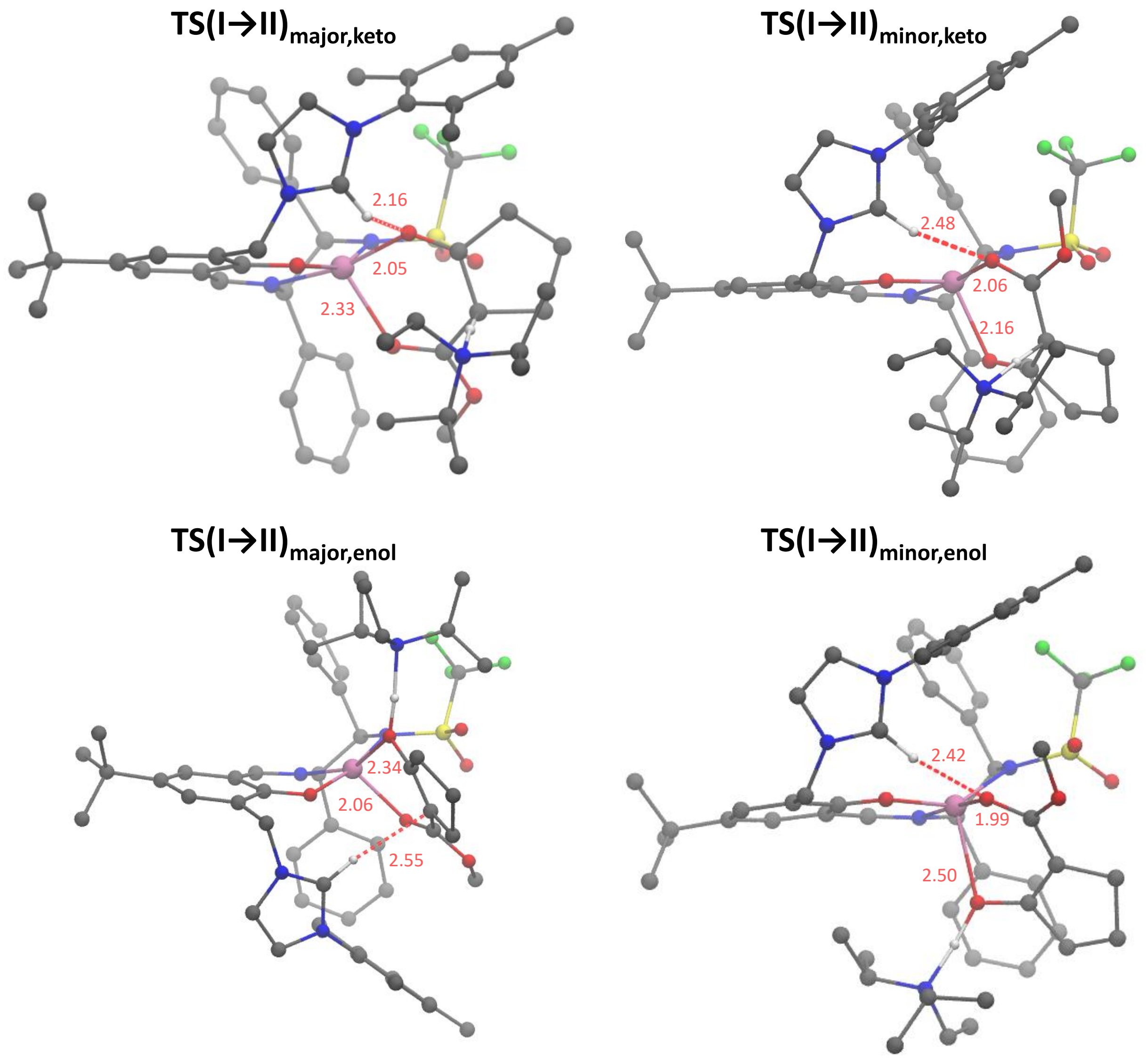

Figure S21: Deprotonation transition states the major and minor product channels, shown for both the keto (**$TS(I \rightarrow II)_{major,keto}$** and **$TS(I \rightarrow II)_{minor,keto}$**) and enol (**$TS(I \rightarrow II)_{major,enol}$** and **$TS(I \rightarrow II)_{minor,enol}$**) tautomers. The interaction between the imidazolium C(2)-H and the β-ketoester is indicated (Å). White: H, grey: C, red: O, blue: N, yellow: S, green: F, and pink: Cu. Non-relevant hydrogens are omitted for visual clarity.

As described above, the deprotonation barrier of the non-coordinated β-ketoester in DCM was determined to be $\Delta G^{\ddagger}_{\text{keto-enol,DCM}}$ = 88.7 kJ mol^{-1} (see section 11.2), which is higher than the barrier of the Cu enol complexes. In contrast, the deprotonation of the coordinated keto tautomer $\mathbf{I}_{\mathbf{major,keto}}$ ($\Delta G^{\ddagger}_{\text{major,keto}}$ = 95.6 kJ mol^{-1}) is higher than for the non-coordinated β-ketoester. For the coordinated enol tautomer an $\underline{O}$···H···$\underline{N}_{base}$ distance of 2.60 Å was found, for the non-coordinated β-ketoester a $\underline{C}$···H···$\underline{N}_{base}$ distance of 2.79 Å, whereas for the coordinated keto form a $\underline{C}$···H···$\underline{N}_{base}$ distance of 2.83 Å was calculated. The increased distance in the latter case is explained by the large steric demand of DIPEA, as the C–H bond of the coordinated

β-ketoester is sterically less accessible (also in comparison to the O–H bond of the coordinated enol). Besides the DFT analysis, we additionally performed microkinetic modelling (see section 11.5), which independently supports this conclusion by showing good agreement only for the deprotonation *via* the enol form.

11.3.3 Coordination of the Enolate

The coordination of the enolate to the catalyst in the major channel exhibits a lower free energy ΔG_{major} = -3.2 kJ mol^{-1}, compared to the minor channel ΔG_{minor} = 13.7 kJ mol^{-1}. This energetic preference is reflected in the shorter bond distances between the enolate and NHC of 2.33 Å for the major channel and 2.43 Å in the minor channel (Figure S22).

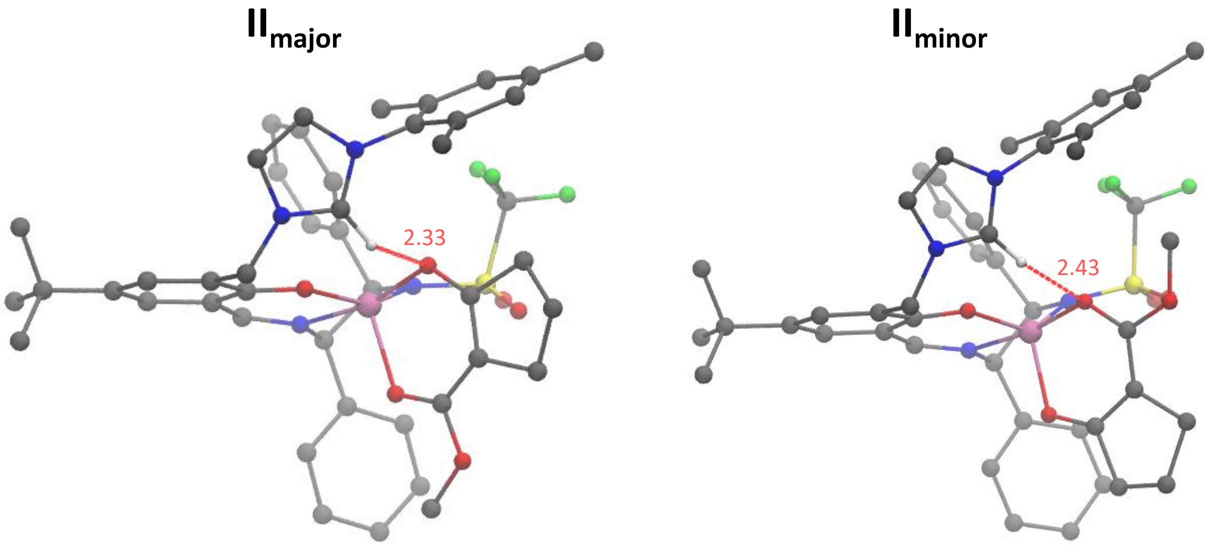

Figure S22: Structure of **II** for the major product channel (left) and the minor product channel (right). The interaction between the imidazolium C(2)-H and the enolate is indicated (Å). White: H, grey: C, red: O, blue: N, yellow: S, green: F and pink: Cu. Non-relevant hydrogens are omitted for visual clarity.

11.3.4 Rate-Determining Transition State

In the following step of the mechanism, benzyl bromide associates to the enolate complex and subsequently the C-C bond is formed between the nucleophilic carbon of the enolate and the electrophilic carbon of benzyl bromide. This step corresponds to the rate-determining transition state, which exhibits a barrier of $\Delta G^{\ddagger}_{TS,major}$ = 58.3 kJ mol^{-1} for the major channel and $\Delta G^{\ddagger}_{TS,minor}$ = 72.1 kJ mol^{-1} for the minor channel. Consequently, the difference in the transition-state free-energy barriers is higher than expected but remains within the expected DFT accuracy.

The significant difference between the respective channels originates from the distinct orientation of the enolate relative to the metal center, which leads to different steric interactions.

These effects are reflected in the varying distances between the hydrogen atom of the imidazolium unit and the respective oxygen atom of the enolate, measuring 2.34 Å in the major transition state and 2.63 Å in the minor transition state (Figure S23). In the minor channel, the steric repulsion involving the methoxy substituent is larger, as it is oriented toward the ligand framework of the catalyst. In contrast, in the major channel, the methoxy group is directed away from the ligand environment, thereby minimizing steric repulsion.

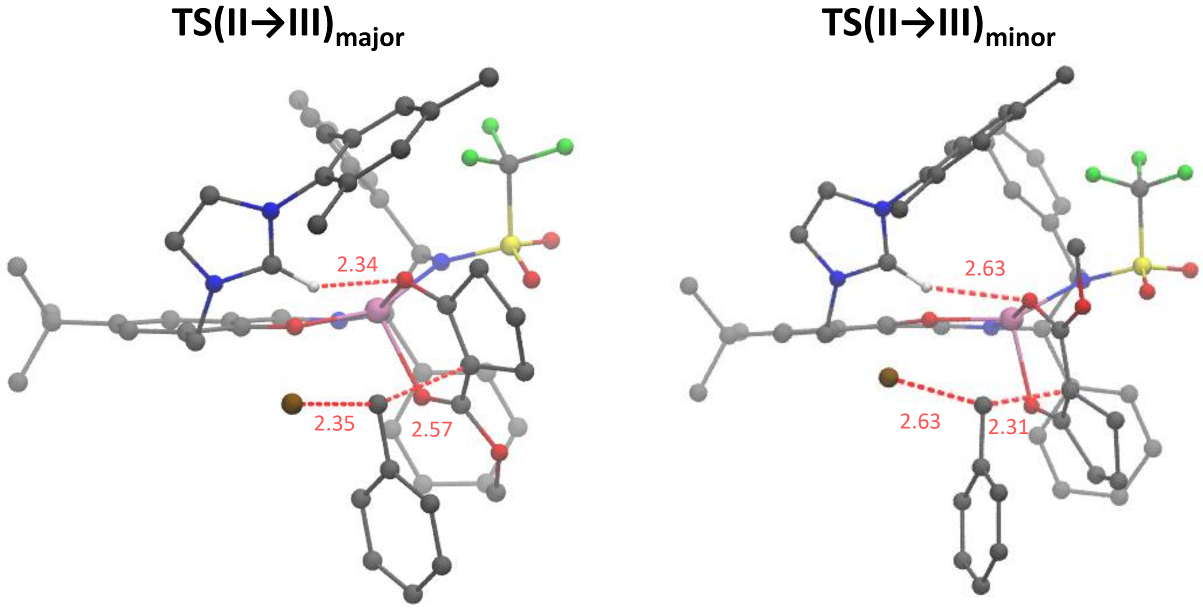

Figure S23: The rate-limiting transition state (**TS(II→III)**) in the major product channel (left) and the minor product channel(right). The interaction between the imidazolium C(2)-H and the enolate is indicated (Å). White: H, grey: C, red: O, blue: N, yellow: S, green: F and pink: Cu. Non-relevant hydrogens are omitted for visual clarity.

11.3.5 Coordination of Product

The final step of the reaction constitutes the product coordinating to the catalyst (Figure S24). As observed in earlier intermediates, the main difference between the major and minor channel is the orientation of the enolate unit relative to the catalyst, which lead to the respective *R* and *S* product. Although the corresponding bond lengths are comparable, the free energies of the two product–catalyst complexes differ with $\Delta G_{\text{Prod,major}}$ = -62.8 kJ mol^{-1} and $\Delta G_{\text{Prod,minor}}$ = -54.6 kJ mol^{-1}. Subsequent product dissociation of the product and reassociation of a new substrate regenerate the active catalyst and complete the catalytic cycle.

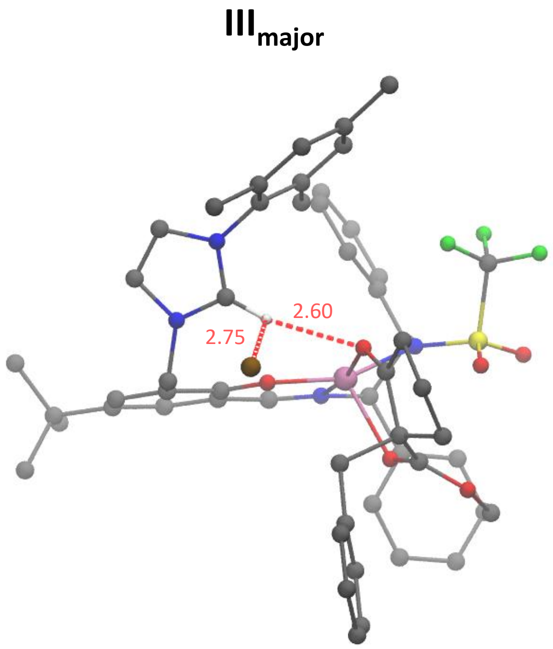

Figure S24: The product coordinated to the complex (**III**) in the major product channel (left) and the minor product channel(right). The interaction between the imidazolium C(2)-H and the alkylated β-ketoester is indicated (Å). White: H, grey: C, red: O, blue: N, yellow: S, green: F, and pink: Cu. Non-relevant hydrogens are omitted for visual clarity.

11.4 Non-Covalent Interaction Analysis

For further investigation on the enantioselectivity of the obtained products we examined the relevant transition states with an additional focus on the non-covalent interactions (NCIs). These weak interactions, like van der Waals forces, π-π stacking or steric repulsion, may explain many mechanistic features such as enantioselectivity and rate differences. NCI analysis[64,65] provides a powerful framework to directly visualize and compare these interactions and thereby understand the mechanistic nature of the transition state.

The NCI approach is based on the reduced electron density gradient $s(\mathrm{r})$, which is defined as the inhomogeneity of the electron density at a point r, or in other words, the ratio of which the density changes with respect to the uniform electron gas (7).[64]

$$s(\mathrm{r}) = \frac{1}{2(3\pi^2)^{1/3}} \frac{|\nabla\rho(r)|}{\rho(r)^{4/3}} \quad (7)$$

In order to visualize the NCI, the reduced density gradient can be plotted by low valued isosurfaces of $s(\mathrm{r}) = 0.3 - 0.5$ in combination with a low electron density cut-off of $\rho(r) = 0.05$ to exclude visualization of interactions associated with covalent bonding. To qualitatively determine whether any interaction is attractive or repulsive, the sign of the second eigenvalue $\mathrm{sign}(\lambda_2)$ of the Hessian is used. Specifically, $\mathrm{sign}(\lambda_2) < 0$ corresponds to strong attractive interactions (e.g. hydrogen bonds), $\mathrm{sign}(\lambda_2) \approx 0$ indicates weak attractive interactions (e.g. van der Waals forces) and repulsive interactions (e.g. steric clashes) are characterized by $\mathrm{sign}(\lambda_2) > 0$. This classification is especially useful for complex transition states where multiple weak interactions act cooperatively. Consequently, NCI analysis provides a robust basis for rationalizing differences between competing transition states.

For the NCI Analysis we used the NCIPlot 4.3 software[64,65] VMD 19.3[66] as well as gnuplot 6.0[67] for visualization purposes.

Investigation of the Rate Determining Transitions States TS(II→III) by NCI Analysis

In addition to our DFT calculations, NCI analysis was performed to elucidate the structural differences of the transition state geometries.

The results of the NCI analysis are presented in Figures S27-29. A color code based on $\mathrm{sign}(\lambda_2)\rho$ is used to distinguish different types of interactions, where strongly attractive interactions (e.g. hydrogen bonds) are displayed in blue, weak attractive interactions (e.g. van der Waals forces) in green and strong repulsive interactions (e.g. steric clashes) in red. Figure

S25 and Figure S26 depict the NCIs as three-dimensional isosurfaces from different perspectives, which is well suited for identifying specific interactions important for the transition state geometries. To enable quantitative comparison between the interactions of the respective transition state, Figure S27 describes the same interactions, plotting $s(\mathrm{r})$ against $\mathrm{sign}(\lambda_2)\rho$.

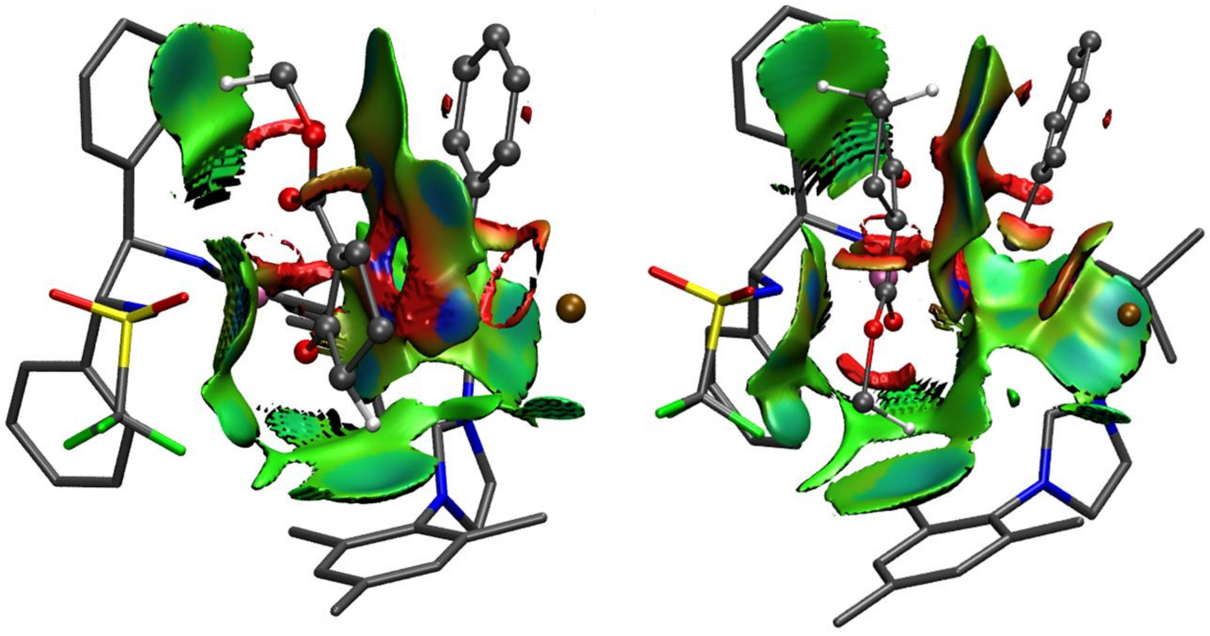

Figure S25: NCI analysis using promolecular approximations[64,65] of the rate determining transition state **TS(II→III)** for the major channel (left) and the minor channel (right). The isosurface value was set to 0.5. Most hydrogens are omitted for visual clarity. Attractive interactions are indicated in blue, weak attractive interactions in green and steric clashes in red.

The NCI analysis reveals multiple NCIs that contribute to the stabilization of the investigated transition state for both major and minor channel. Among these the van der Waals interactions between the enolate and the benzyl bromide appear to be particularly significant. From visual inspection the isosurfaces suggests a slightly larger interaction region for the major channel, hence favouring this pathway. In addition to that, several CH-π interactions could be identified. In the major channel these include interactions between a CH_2 unit of the enolate and the aromatic ring of the mesityl unit, as well as between the CH_3 unit of the enolates methoxy substituent and the aromatic system of the diamino backbone. For the minor channel analogous interactions are present, but due to the reversed geometry of the enolate their spatial arrangement is also reversed. In this case, the interaction with the imidazolium unit goes *via* the methoxy group, whereas the interaction with the aromatic systems of the diamino backbone and benzyl bromide occurs *via* the CH_2 unit. Finally, in both channels a weak interaction between the bromide anion and imidazolium unit can be observed, indicating that

the transition state structures are not purely governed by π-interactions or hydrogen bonding but also additionally *via* electrostatic interactions.[64]

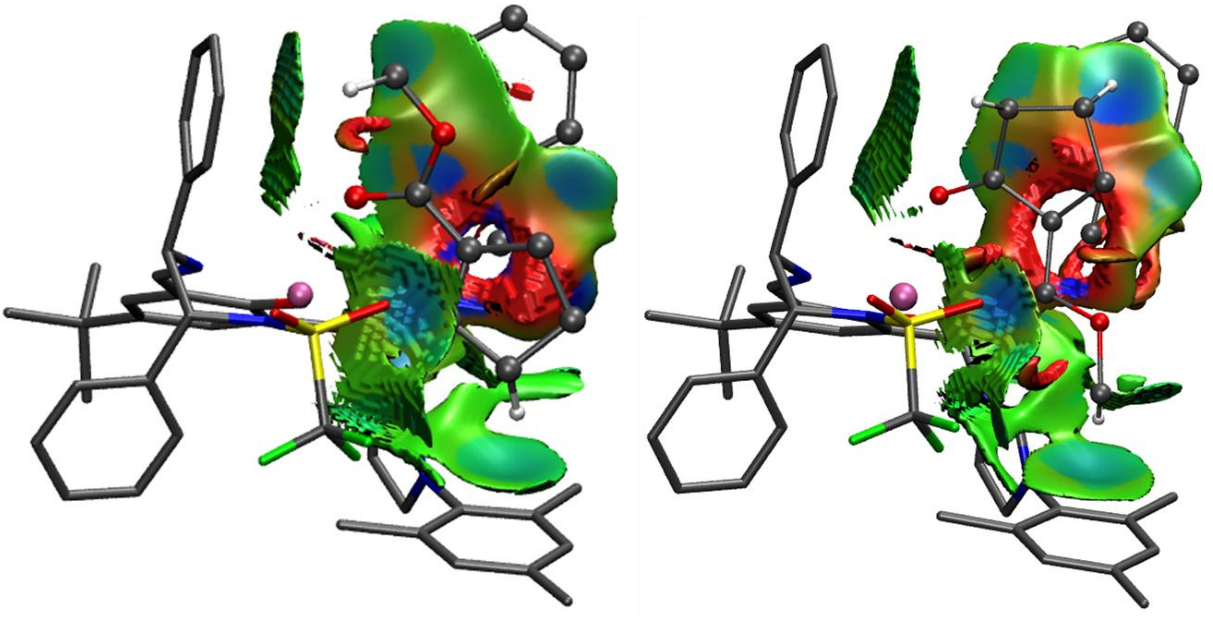

Figure S26: Different perspective of the NCI analysis using promolecular approximations[64,65] of the rate determining transition state **TS(II→III)** for the major channel (left) and the minor channel (right). The isosurface value was set to 0.5. Most hydrogens are omitted for visual clarity. Attractive interactions are indicated in blue, weak attractive interactions in green and steric clashes in red.

Figure S26 presents the NCI analysis from an alternative perspective, facilitating a clearer comparison of the differences in the isosurfaces between the enolate and benzyl bromide. Also, from this viewpoint, an additional interaction can be observed here, namely an electrostatic interaction between an oxygen atom of the triflate group and the α-C, which is more pronounced for the major channel.

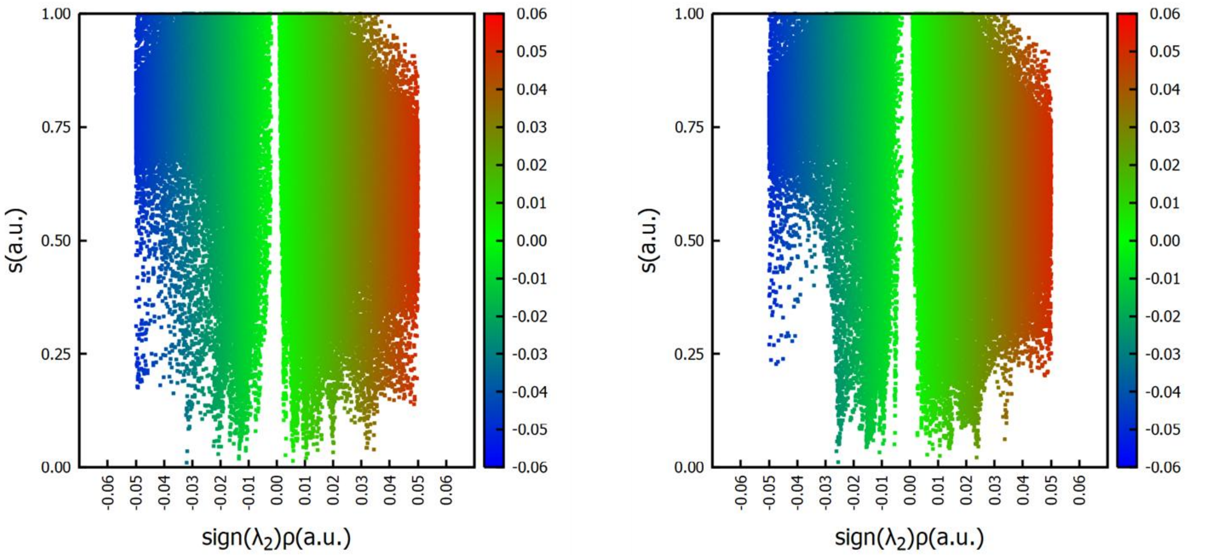

Figure S27: Reduced density gradient $s(\mathbf{r})$ with respect to the sign of the second eigenvalue of the Hessian $\mathbf{sign}(\boldsymbol{\lambda}_2)\boldsymbol{\rho}$ for major (left) and the minor channel (right). Strong attractive interactions (e.g. hydrogen bonds) are represented in blue, weak attractive interactions (e.g. van der Waals forces) in green and repulsive interactions (e.g. steric clashes) in red.

As mentioned above quantitative comparison of the three-dimensional isosurfaces obtained by NCI analysis is not straightforward. Therefore, we also computed two-dimensional plots of $s(\mathrm{r})$ with respect to $\mathrm{sign}(\lambda_2)\rho$, which are better suited for a quantitative comparison between major and minor channel. Comparing these results for both channels indicates that the attractive interactions are more prevalent in the major channel.

11.5 Influence of the Solvent on the Reaction Mechanism

The reaction was investigated using two different solvent systems. As mentioned above, an important driving force of the catalysis is that the side product HDIPEA · Br precipitates in ethyl acetate (EtOAc), thereby promoting product formation and increasing the overall yield. In order to carry out kinetic investigations, the concentration of each species in the reaction mixture has to be monitored. This is only possible if all of these species are present in solution. Therefore, the solvent was changed to DCM, which does not lead to HDIPEA · Br precipitation.

The choice of solvent influences the computed potential energy surfaces, which can be seen in Figure S28, where the surfaces using EtOAc and DCM as solvents are compared. The most notable difference is that the energy of all stationary points is about 25-30 kJ mol^{-1} higher in energy when DCM is used. This shift is consistent with the lower dielectric constant of DCM ($\varepsilon = 6.02$) relative to EtOAc ($\varepsilon = 8.93$), which provides less stabilization of the polar catalyst–substrate complex. However, the relative energy differences remain essentially unchanged

regardless of the solvent chosen. In both cases, the reaction is characterized by a lower barrier for the deprotonation of the β-ketoester and a higher barrier for the carbon-carbon bond formation. The overall determining driving force is in both cases similarly exergonic with ΔG_{DCM} = 82.4 kJ mol^{-1} and ΔG_{EtOAc} = 94.6 kJ mol^{-1}.

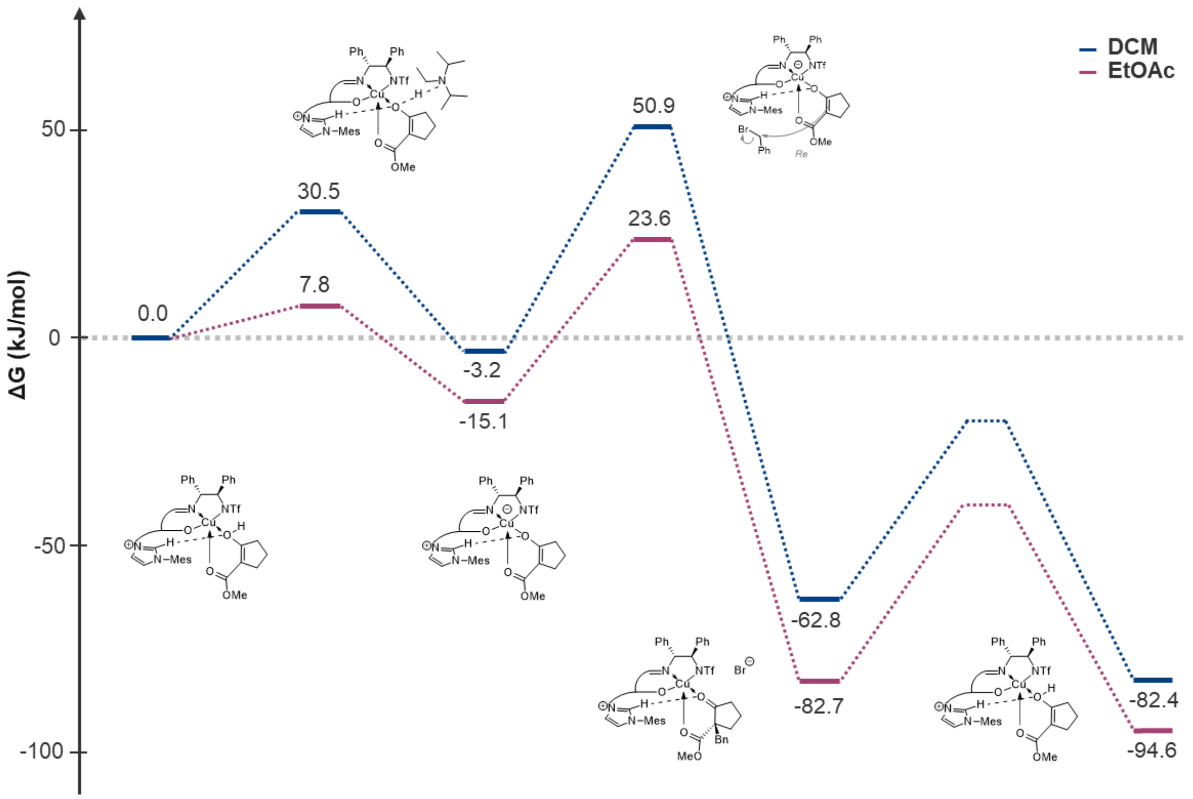

Figure S28: Free energy profile of the investigated mechanism for different solvents (red: EtOAc, blue: DCM). The solvent effects were treated implicitly using COSMO for EtOAc (ε = 6.02) and COSMO-rs for DCM (ε = 8.93). Structure **I** was chosen as the reference (ΔG = 0 kJ/mol) of the catalyst for both solvents.

11.6 Microkinetic Modelling

Microkinetic modelling allows a direct comparison between a DFT energy profile and experimentally observed kinetic data. The catalytic reaction mechanism is divided into elementary reaction steps, each described by a rate equation and a rate constant. Solving the coupled rate equations provides the time-dependent evolution of reactant and product concentrations. Rate constants are adjusted to fit the experimentally measured concentration profiles. Since rate constants can be directly converted to barriers using transition-state theory, they can be compared to DFT results.[68] However, it must be noted that the assignment is by no means unique: several sets of rate constants can typically fit the experimental data equally well. Here, we have fitted five representative sets of rate constants to the experimental data and report the corresponding energy ranges.

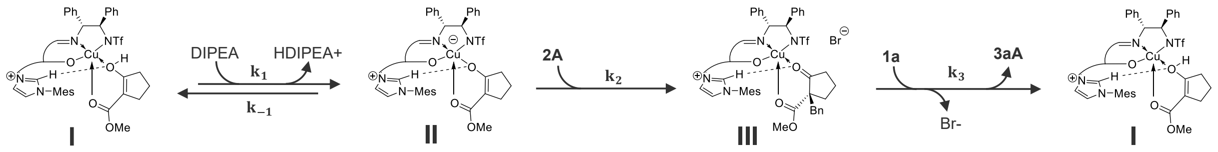

Figure S29: Description of the elementary reaction steps postulated for the microkinetic modelling. Rate constants for the backward reaction are indicated by a negative sign.

All kinetic data are obtained from NMR-monitored catalytic reactions performed under systematically varied initial conditions (Figure S30).[44–46]

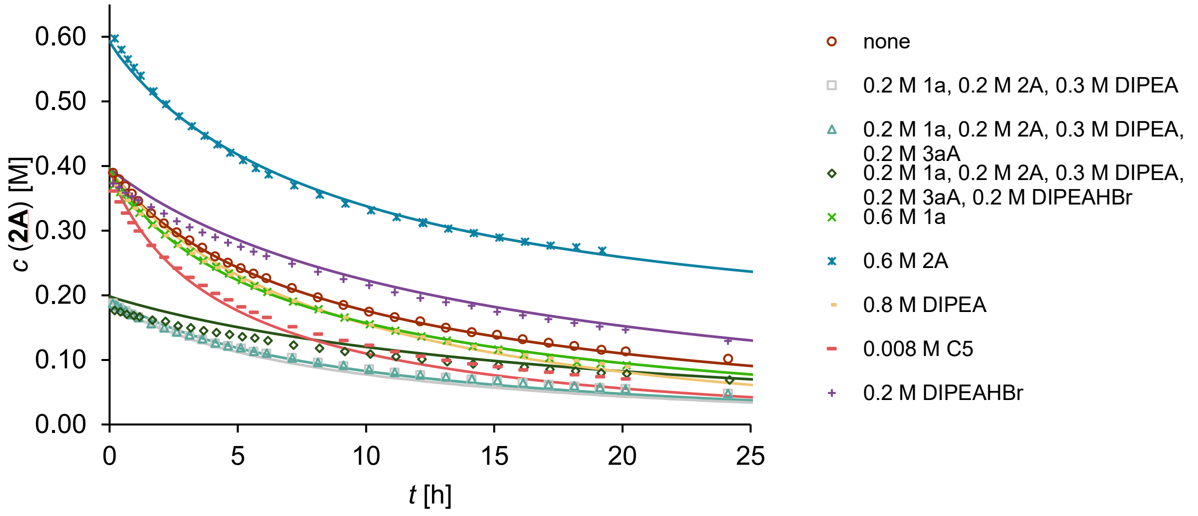

Figure S30: Experimentally obtained concentrations (symbols) and simulated concentration profiles (lines) obtained by our microkinetic model. All experimental data is classified by the change compared to the initial experiment (specified as none), which are: 0.4 M **1a** (1 equiv.), 0.4 M **2A** (1 equiv.), 0.5 M DIPEA, 0.0 M **3aA**, 0.0 M DIPEAHBr, and 0.04 M **C5**. All reactions were carried out at room temperature and using CD_2Cl_2 as a solvent.

Comparison of the free energies calculated *via* DFT and obtained by microkinetic modelling shows a degree of consistency that can be expected from such simulations. We fitted five kinetic models to the experimental data. Starting from different initial conditions, we obtained equivalent agreement with the experimental data for all of them. While, for example, the barriers from **I** to **II** differed significantly between the models, the energy difference between **I** and **II** is, with 0–4 kJ mol^{-1}, very similar between them. That agrees well with the -3.2 kJ mol^{-1} we found from DFT. The barrier for the C-C bond formation is with 83–85 kJ mol^{-1}, again very narrowly constrained by the models. The barrier is somewhat higher than found by DFT (54.1 kJ mol^{-1}), which can be attributed to DFT errors. After all, a microkinetic model is a very stringent test for DFT. The remaining energies, the barrier and energy difference between **III** and **I**, are, again, hardly constrained by the microkinetic model. Any values for the energy of

III higher than that of **I** after the cycle are consistent with the experimental data. All that indicates that the reaction mechanism as proposed is reasonable.

Table S15: Comparison between the free Gibbs energies $\Delta G^{‡}$ for each elementary reaction used to model the catalytic cycle (**k_1 - k_3**) and the keto-enol-tautomerization (**k_{taut1}** and **k_{taut-1}**) determined by both DFT ($\Delta G^{‡}_{DFT}$) in DCM and microkinetic modelling ($\Delta G^{‡}_{MKM}$).

label	reaction	$\Delta G^{‡}_{DFT}$ (kJ mol^{-1})	$\Delta G^{‡}_{MKM}$ (kJ mol^{-1})
k_1	**I** + DIPEA → **II** + $HDIPEA^+$	30.5	40-80
k_{-1}	**II** + $HDIPEA^+$ → **I** + DIPEA	33.7	40-76
k_2	**2A** + **II** → **III** + Br^-	54.1	83-85
k_3	**III** + **1a'** (enol) → **I** + **3aA**	-	74-75
k_{-3}	**I** + **3aA** → **III** + **1a'** (enol)	-	82-87
k_{taut1}	**1a** (keto) → **1a'** (enol)	88.7	93-78
k_{taut-1}	**1a'** (enol) → **1a** (keto)	80.7	86-71

We also examined the keto-enol tautomerization and the background reaction leading to the major product. The results indicate that DFT and the microkinetic model are in good agreement about the energetic difference between the keto and enol form of about 8.0 kJ mol^{-1}.

Using a microkinetic model comprising the recorded concentration profiles, we achieved excellent fits of all nine kinetic measurements (Figure S30). Various barrier combinations can achieve similarly good fits, consistently giving a barrier for the C-C bond formation of 83–85 kJ mol^{-1}. This value is higher than the one found by DFT (54.1 kJ mol^{-1}), which can probably be attributed to errors in the DFT method. All good fits also show that there is an equilibrium between **I** and **II** with very similar Gibbs energy of the two states, being consistent with DFT (3.2 kJ mol^{-1}) and our determined rate law.

12 References

[1] A. C. Hans, P. M. Becker, J. Haußmann, S. Suhr, D. M. Wanner, V. Lederer, F. Willig, W. Frey, B. Sarkar, J. Kästner, R. Peters, "A Practical and Robust Zwitterionic Cooperative Lewis Acid / Acetate / Benzimidazolium Catalyst for Direct 1,4-Additions" *Angew. Chem. Int. Ed.* **2023**, *62*, e202217519.

[2] H. Kim, Y. Nguyen, C. P. H. Yen, L. Chagal, A. J. Lough, B. M. Kim, J. Chin, "Stereospecific synthesis of C2 symmetric diamines from the mother diamine by resonance-assisted hydrogen-bond directed diaza-cope rearrangement" *J. Am. Chem. Soc.* **2008**, *130*, 12184–12191.

[3] M. Zhou, K. Li, D. Chen, R. Xu, G. Xu, W. Tang, "Enantioselective Reductive Coupling of Imines Templated by Chiral Diboron" *J. Am. Chem. Soc.* **2020**, *142*, 10337–10342.

[4] J. E. D. Martins, M. Wills, "Ir(III) complexes of diamine ligands for asymmetric ketone hydrogenation" *Tetrahedron* **2009**, *65*, 5782–5786.

[5] J. Schmid, T. Junge, J. Lang, W. Frey, R. Peters, "Polyfunctional Bis-Lewis-Acid-/Bis-Triazolium Catalysts for Stereoselective 1,4-Additions of 2-Oxindoles to Maleimides" *Angew. Chem. Int. Ed.* **2019**, *58*, 5447–5451.

[6] B. Peng, D. Geerdink, N. Maulide, "Electrophilic rearrangements of chiral amides: A traceless asymmetric α-allylation" *J. Am. Chem. Soc.* **2013**, *135*, 14968–14971.

[7] J. Pecyna, D. Pociecha, P. Kaszyński, "Zwitterionic pyridinium derivatives of [closo-1-CB9H10]− and [closo-1-CB11H12]− as high Δε additives to a nematic host" *J. Mater. Chem. C* **2014**, *2*, 1585–1591.

[8] E. Abraham, T. D. W. Claridge, S. G. Davies, B. Odell, P. M. Roberts, A. J. Russell, A. D. Smith, L. J. Smith, H. R. Storr, M. J. Sweet, A. L. Thompson, J. E. Thomson, G. E. Tranter, D. J. Watkin, "A systematic study of the solid state and solution phase conformational preferences of β-peptides derived from C(3)-alkyl substituted transpentacin derivatives" *Tetrahedron Asymmetry* **2011**, *22*, 69–100.

[9] Q. Wang, C. Wilson, A. J. Blake, S. R. Collinson, P. A. Tasker, M. Schröder, "The one-pot halomethylation of 5-substituted salicylaldehydes as convenient precursors for the preparation of heteroditopic ligands for the binding of metal salts" *Tetrahedron Lett.* **2006**, *47*, 8983–8987.

[10] J. Schmid, W. Frey, R. Peters, "Polynuclear Enantiopure Salen−Mesoionic Carbene Hybrid Complexes" *Organometallics* **2017**, *36*, 4313–4324.

[11] J. Liu, J. Chen, J. Zhao, Y. Zhao, L. Li, H. Zhang, “A Modified Procedure for the Synthesis of 1-Arylimidazoles” *Synthesis (Stuttg).* **2003**, 2661–2666.

[12] M. Mechler, K. Latendorf, W. Frey, R. Peters, “Homo- and heterobimetallic Pd-, Ag-, and Ni-hybrid salen-bis-NHC complexes” *Organometallics* **2013**, *32*, 112–130.

[13] K. Latendorf, M. Mechler, I. Schamne, D. Mack, W. Frey, R. Peters, “Titanium Salen Complexes with Appended Silver NHC Groups as Nucleophilic Carbene Reservoir for Cooperative Asymmetric Lewis Acid/NHC Catalysis” *Eur. J. Org. Chem.* **2017**, *2017*, 4140–4167.

[14] S. P. Chavan, P. B. Lasonkar, “One-pot migration–formylation of benzyl aryl ethers under Duff reaction condition” *Tetrahedron Lett.* **2013**, *54*, 4789–4792.

[15] S. Bauri, A. Ramachandran, A. Rit, “Base-catalyzed Effective C2-Amidation of Azolium Salts Using Isocyanates under Mild Conditions” *Chem Asian J.* **2023**, *18*, e202201301.

[16] M. Kaik, J. Gawroński, “Facile monoprotection of trans-1,2-diaminocyclohexane” *Tetrahedron Asymmetry* **2003**, *14*, 1559–1563.

[17] F. Xue, S. Zhang, W. Duan, W. Wang, “A Novel Bifunctional Sulfonamide Primary Amine-Catalyzed Enantioselective Conjugate Addition of Ketones to Nitroolefins” *Adv. Synth. Catal.* **2008**, *350*, 2194–2198.

[18] T. Baba, J. Yamamoto, K. Hayashi, M. Sato, M. Yamanaka, T. Kawabata, T. Furuta, “Catalytic discrimination between formyl groups in regio- and stereoselective intramolecular cross-aldol reactions” *Chem. Sci.* **2016**, *7*, 3791–3797.

[19] F. Willig, J. Lang, A. C. Hans, M. R. Ringenberg, D. Pfeffer, W. Frey, R. Peters, “Polyfunctional Imidazolium Aryloxide Betaine/Lewis Acid Catalysts as Tools for the Asymmetric Synthesis of Disfavored Diastereomers” *J. Am. Chem. Soc.* **2019**, *141*, 12029–12043.

[20] D. M. Wanner, P. M. Becker, S. Suhr, N. Wannenmacher, S. Ziegler, J. Herrmann, F. Willig, J. Gabler, K. Jangid, J. Schmid, A. C. Hans, W. Frey, B. Sarkar, J. Kästner, R. Peters, “Cooperative Lewis Acid-1,2,3-Triazolium-Aryloxide Catalysis: Pyrazolone Addition to Nitroolefins as Entry to Diaminoamides” *Angew. Chem. Int. Ed.* **2023**, *62*, e202307317.

[21] A. C. Hans, *Dissertation*, Institute for Organic Chemistry, University of Stuttgart, **2023**.

[22] F. Tan, M. Pu, J. He, J. Li, J. Yang, S. Dong, X. Liu, Y. D. Wu, X. Feng, "Catalytic Asymmetric Homologation of Ketones with α-Alkyl α-Diazo Esters" *J. Am. Chem. Soc.* **2021**, *143*, 2394–2402.

[23] L. H. P. Teixeira, E. J. Barreiro, C. A. M. Fraga, "Reduction of 2-Alkyl-2-carbomethoxy-cyclopentanone Derivatives with Sodium Borohydride. II. The Elucidation of the Diastereoselective Control" *Synth. Commun.* **1997**, *27*, 3241–3257.

[24] J. Le Nôtre, D. Van Mele, C. G. Frost, "A New Method for Constructing Quaternary Carbon Centres: Tandem Rhodium-Catalysed 1,4-Addition/Intramolecular Cyclisation" *Adv. Synth. Catal.* **2007**, *349*, 432–440.

[25] K. Ohkata, "Substituent Effects for the Benzene Ring on Solvolysis of 3,4-Benzotricyclo[4.3.1.01,6]dec-3-en-2-yl p-Nitrobenzoate" *BCSJ* **1976**, *49*, 235–244.

[26] A. Kato, Y. Ikeda, N. Sugita, T. Nitta, H. Enari, K. Niimura, A. Kashima, M. Konno, "Aromatase Inhibitors: Synthesis, Biological Activity, and Structure of 1,2-Imidazolylmethylcyclopentanol Derivatives" *Chem. Pharm. Bull.* **1995**, *43*, 2152–2158.

[27] M. Yoshida, "Asymmetric α-Allylation of α-Substituted β-Ketoesters with Allyl Alcohols" *J. Org. Chem.* **2017**, *82*, 12821–12826.

[28] C. Chapuis, E. Walther, F. Robvieux, C. A. Richard, L. Goumaz, J. Y. De Saint Laumer, "Route Scouting towards a Methyl Jasmonate Precursor" *Helv. Chim. Acta* **2016**, *99*, 95–109.

[29] J. R. Dehli, V. Gotor, "Preparation of enantiopure ketones and alcohols containing a quaternary stereocenter through parallel kinetic resolution of β-keto nitriles" *J. Org. Chem.* **2002**, *67*, 1716–1718.

[30] Z. Protich, L. L. Lowder, R. P. Hughes, J. Wu, "Regiodivergent (3 + 2) annulation reactions of oxyallyl cations" *Chem. Sci.* **2023**, *14*, 5196–5203.

[31] W. Li, F. Tan, X. Hao, G. Wang, Y. Tang, X. Liu, L. Lin, X. Feng, "Catalytic Asymmetric Intramolecular Homologation of Ketones with α-Diazoesters: Synthesis of Cyclic α-Aryl/Alkyl β-Ketoesters" *Angew. Chem. Int. Ed.* **2015**, *54*, 1608–1611.

[32] Q. H. Deng, H. Wadepohl, L. H. Gade, "Highly enantioselective copper-catalyzed alkylation of β-ketoesters and subsequent cyclization to spirolactones/bi-spirolactones" *J. Am. Chem. Soc.* **2012**, *134*, 2946–2949.

[33] S. Qin, S. Liu, Y. Cao, J. Li, C. Chong, T. Liu, Y. Luo, J. Hu, S. Jiang, H. Zhou, G. Yang, C. Yang, "α-Alkylation of Chiral Sulfinimines for Constructing Quaternary Chiral Carbons by Introducing Removable Directing Groups" *Org. Lett.* **2018**, *20*, 1350–1354.

[34] K. Kato, H. Suemune, K. Sakai, "Asymmetric alkylation using chiral cyclic diols to prepare a quaternary carbon" *Tetrahedron* **1994**, *50*, 3315–3326.

[35] H. Falk, W. Fröstl, K. Schlögl, "Darstellung und absolute Konfiguration von optisch aktivem 2,2′-spiro-biindanon-1" *Tetrahedron Lett.* **1974**, *15*, 217–220.

[36] P. Niedbała, M. Majdecki, P. Grodek, J. Jurczak, "H-Bond Mediated Phase-Transfer Catalysis: Enantioselective Generating of Quaternary Stereogenic Centers in β-Keto Esters" *Molecules* **2022**, *27*, 2508.

[37] W. Chen, Z. Liu, J. Tian, J. Li, J. Ma, X. Cheng, G. Li, "Building Congested Ketone: Substituted Hantzsch Ester and Nitrile as Alkylation Reagents in Photoredox Catalysis" *J. Am. Chem. Soc.* **2016**, *138*, 12312–12315.

[38] K. M. Peese, D. Y. Gin, "Enantioselective approach to the hetisine alkaloids. Synthesis of the 3-methyl-1-aza-tricyclo[5.2.1.03,8]decane core via intramolecular dipolar cycloaddition" *Org. Lett.* **2005**, *7*, 3323–3325.

[39] T. Hashimoto, Y. Naganawa, K. Maruoka, "Desymmetrizing asymmetric ring expansion of cyclohexanones with α-diazoacetates catalyzed by chiral aluminum Lewis acid" *J. Am. Chem. Soc.* **2011**, *133*, 8834–8837.

[40] B. Milenkov, M. Hesse, "Eine neue Synthese von (±)-Dihydrorecifeiolid" *Helv. Chim. Acta* **1986**, *69*, 1323–1330.

[41] D. G. Blackmond, "Kinetic Profiling of Catalytic Organic Reactions as a Mechanistic Tool" *J. Am. Chem. Soc.* **2015**, *137*, 10852–10866.

[42] R. D. Baxter, D. Sale, K. M. Engle, J. Q. Yu, D. G. Blackmond, "Mechanistic rationalization of unusual kinetics in Pd-catalyzed C-H olefination" *J. Am. Chem. Soc.* **2012**, *134*, 4600–4606.

[43] S. Stoll, A. Schweiger, "EasySpin, a comprehensive software package for spectral simulation and analysis in EPR" *J. Magn. Reson.* **2006**, *178*, 42–55.

[44] C. D. T. Nielsen, J. Burés, “Visual kinetic analysis” *Chem. Sci.* **2019**, *10*, 348–353.

[45] J. Burés, “Variable Time Normalization Analysis: General Graphical Elucidation of Reaction Orders from Concentration Profiles” *Angew. Chem. Int. Ed.* **2016**, *55*, 16084–16087.

[46] J. Burés, “A Simple Graphical Method to Determine the Order in Catalyst” *Angew. Chem. Int. Ed.* **2016**, *55*, 2028–2031.

[47] P. Pracht, F. Bohle, S. Grimme, “Automated exploration of the low-energy chemical space with fast quantum chemical methods” *Phys. Chem. Chem. Phys.* **2020**, *22*, 7169–7192.

[48] S. Grimme, “Exploration of Chemical Compound, Conformer, and Reaction Space with Meta-Dynamics Simulations Based on Tight-Binding Quantum Chemical Calculations” *J. Chem. Theory Comput.* **2019**, *15*, 2847–2862.

[49] C. Bannwarth, S. Ehlert, S. Grimme, “GFN2-xTB—An Accurate and Broadly Parametrized Self-Consistent Tight-Binding Quantum Chemical Method with Multipole Electrostatics and Density-Dependent Dispersion Contributions” *J. Chem. Theory Comput.* **2019**, *15*, 1652–1671.

[50] Y. J. Franzke, C. Holzer, J. H. Andersen, T. Begušić, F. Bruder, S. Coriani, F. Della Sala, E. Fabiano, D. A. Fedotov, S. Fürst, S. Gillhuber, R. Grotjahn, M. Kaupp, M. Kehry, M. Krstić, F. Mack, S. Majumdar, B. D. Nguyen, S. M. Parker, F. Pauly, A. Pausch, E. Perlt, G. S. Phun, A. Rajabi, D. Rappoport, B. Samal, T. Schrader, M. Sharma, E. Tapavicza, R. S. Treß, V. Voora, A. Wodyński, J. M. Yu, B. Zerulla, F. Furche, C. Hättig, M. Sierka, D. P. Tew, F. Weigend, “TURBOMOLE: Today and Tomorrow” *J. Chem. Theory Comput.* **2023**, *19*, 6859–6890.

[51] S. G. Balasubramani, G. P. Chen, S. Coriani, M. Diedenhofen, M. S. Frank, Y. J. Franzke, F. Furche, R. Grotjahn, M. E. Harding, C. Hättig, A. Hellweg, B. Helmich-Paris, C. Holzer, U. Huniar, M. Kaupp, A. Marefat Khah, S. Karbalaei Khani, T. Müller, F. Mack, B. D. Nguyen, S. M. Parker, E. Perlt, D. Rappoport, K. Reiter, S. Roy, M. Rückert, G. Schmitz, M. Sierka, E. Tapavicza, D. P. Tew, C. van Wüllen, V. K. Voora, F. Weigend, A. Wodyński, J. M. Yu, “TURBOMOLE: Modular program suite for ab initio quantum-chemical and condensed-matter simulations” *J. Chem. Phys.* **2020**, *152*, 184107.

[52] S. Grimme, J. G. Brandenburg, C. Bannwarth, A. Hansen, “Consistent structures and interactions by density functional theory with small atomic orbital basis sets” *J. Chem. Phys.* **2015**, *143*, 54107.

[53] S. Grimme, S. Ehrlich, L. Goerigk, "Effect of the damping function in dispersion corrected density functional theory" *J. Comput. Chem.* **2011**, *32*, 1456–1465.

[54] S. Grimme, J. Antony, S. Ehrlich, H. Krieg, "A consistent and accurate ab initio parametrization of density functional dispersion correction (DFT-D) for the 94 elements H-Pu" *J. Chem. Phys.* **2010**, *132*, 154104.

[55] A. D. Becke, "Density-functional exchange-energy approximation with correct asymptotic behavior" *Phys. Rev. A* **1988**, *38*, 3098–3100.

[56] Q. Wu, W. Yang, "Empirical correction to density functional theory for van der Waals interactions" *J. Chem. Phys.* **2002**, *116*, 515–524.

[57] A. D. Becke, "Density-functional thermochemistry. III. The role of exact exchange" *J. Chem. Phys.* **1993**, *98*, 5648–5652.

[58] F. Weigend, R. Ahlrichs, "Balanced basis sets of split valence, triple zeta valence and quadruple zeta valence quality for H to Rn: Design and assessment of accuracy" *Phys. Chem. Chem. Phys.* **2005**, *7*, 3297–3305.

[59] F. Weigend, "Accurate Coulomb-fitting basis sets for H to Rn" *Phys. Chem. Chem. Phys.* **2006**, *8*, 1057–1065.

[60] A. Klamt, "The COSMO and COSMO-RS solvation models" *WIREs Comput. Mol. Sci.* **2011**, *1*, 699–709.

[61] G. Henkelman, H. Jónsson, B. P. Uberuaga, "A climbing image nudged elastic band method for finding saddle points and minimum energy paths" *J. Chem. Phys.* **2000**, *113*, 9901–9904.

[62] G. Henkelman, H. Jónsson, "A dimer method for finding saddle points on high dimensional potential surfaces using only first derivatives" *J. Chem. Phys.* **1999**, *111*, 7010–7022.

[63] J. Kästner, P. Sherwood, "Superlinearly converging dimer method for transition state search" *J. Chem. Phys.* **2008**, *128*, 14106.

[64] J. Contreras-García, E. R. Johnson, S. Keinan, R. Chaudret, J. P. Piquemal, D. N. Beratan, W. Yang, "NCIPLOT: A program for plotting noncovalent interaction regions" *J. Chem. Theory Comput.* **2011**, *7*, 625–632.

[65] R. A. Boto, F. Peccati, R. Laplaza, C. Quan, A. Carbone, J. P. Piquemal, Y. Maday, J. Contreras-García, “NCIPLOT4: Fast, Robust, and Quantitative Analysis of Noncovalent Interactions” *J. Chem. Theory Comput.* **2020**, *16*, 4150–4158.

[66] W. Humphrey, A. Dalke, K. Schulten, “VMD: Visual molecular dynamics” *J. Mol. Graph.* **1996**, *14*, 33–38.

[67] T. Williams, C. Kelley, C. Bersch, H.-B. Bröker, J. Campbell, R. Cunningham, D. Denholm, G. Elber, R. Fearick, C. Grammes, L. Hart, L. Hecking, P. Juhász, T. Koenig, D. Kotz, E. Kubaitis, R. Lang, T. Lecomte, A. Lehmann, J. Lodewyck, A. Mai, B. Märkisch, T. Matsuoka, E. A. Merritt, P. Mikulík, H. Motoyoshi, D. Sebald, C. Steger, S. Takeno, T. Tkacik, J. Van Der Woude, J. R. Van Zandt, A. Woo, J. Zellner, “gnuplot 6.0 An Interactive Plotting Program” **1986**.

[68] J. R. Murdoch, “What is the rate-limiting step of a multistep reaction?” *J. Chem. Educ.* **1981**, *58*, 32–36.

13 NMR Spectra

S8

^{1}H

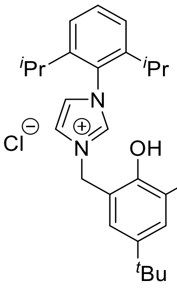

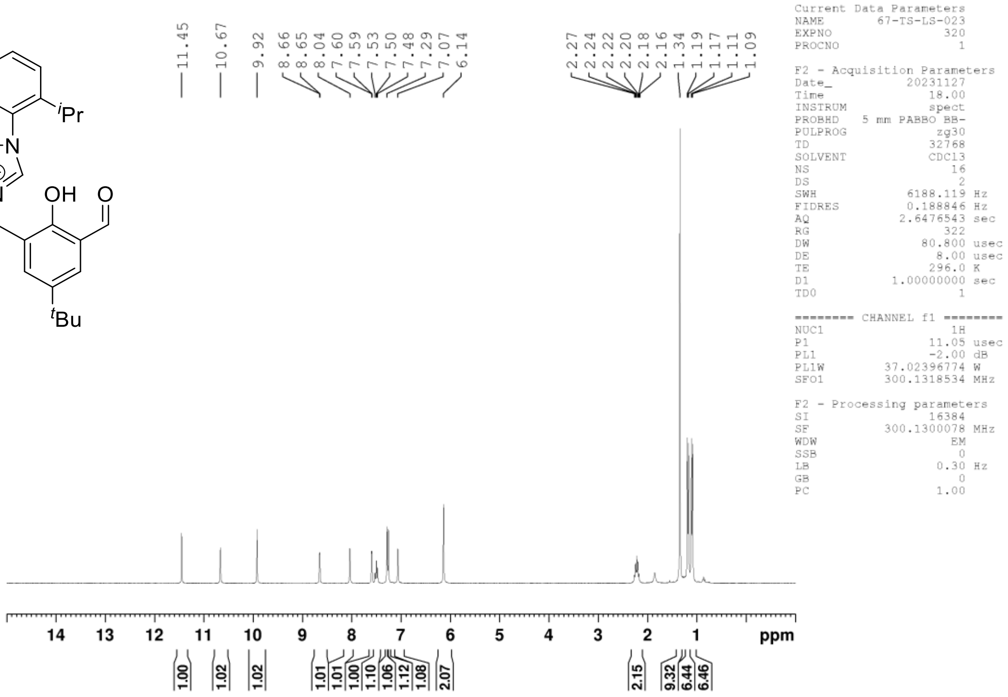

^{13}C

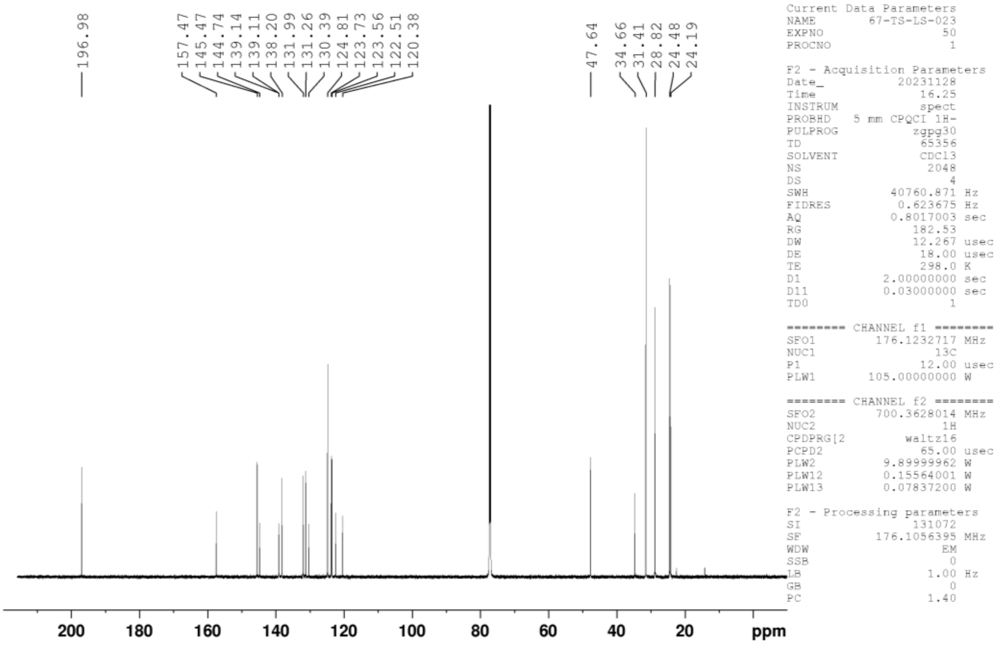

S11

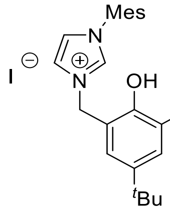
Mes
I
OH
O
tBu

¹H

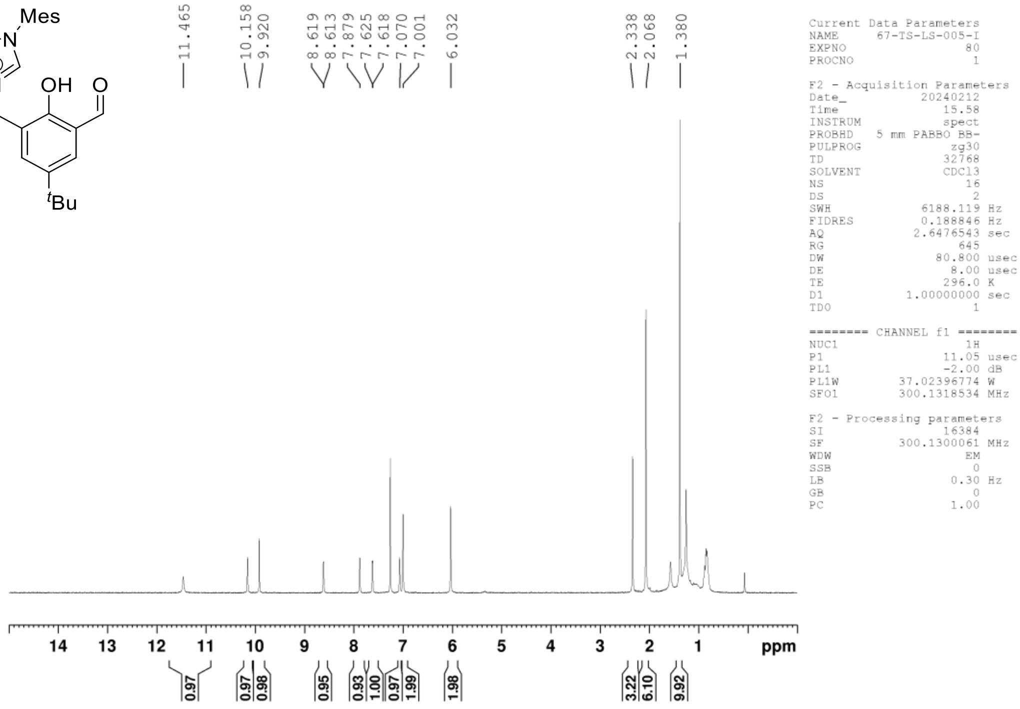
11.465
10.158
9.920
8.619
8.613
7.879
7.625
7.618
7.070
7.001
6.032
2.338
2.068
1.380
0.97
0.97
0.98
0.95
0.93
1.00
0.97
1.99
1.98
3.22
6.10
9.92
14 13 12 11 10 9 8 7 6 5 4 3 2 1 ppm
Current Data Parameters
NAME 67-TS-LS-005-I
EXPNO 80
PROCNO 1
F2 - Acquisition Parameters
Date_ 20240212
Time 15.58
INSTRUM spect
PROBHD 5 mm PABBO BB-
PULPROG zg30
TD 32768
SOLVENT CDCl3
NS 16
DS 2
SWH 6188.119 Hz
FIDRES 0.188846 Hz
AQ 2.6476543 sec
RG 645
DW 80.800 usec
DE 8.00 usec
TE 296.0 K
D1 1.00000000 sec
TD0 1
======== CHANNEL f1 ========
NUC1 1H
P1 11.05 usec
PL1 -2.00 dB
PL1W 37.02396774 W
SFO1 300.1318534 MHz
F2 - Processing parameters
SI 16384
SF 300.1300061 MHz
WDW EM
SSB 0
LB 0.30 Hz
GB 0
PC 1.00

¹³C

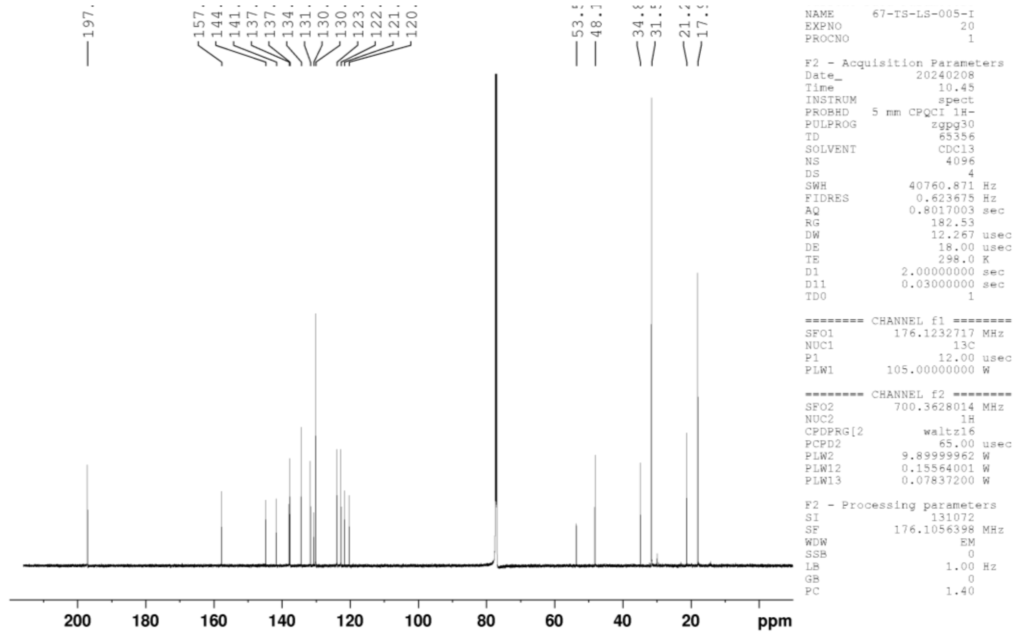
197.
157.
144.
141.
137.
137.
134.
131.
130.
130.
123.
122.
121.
120.
53.
48.
34.
31.
21.
17.
200 180 160 140 120 100 80 60 40 20 ppm
NAME 67-TS-LS-005-I
EXPNO 20
PROCNO 1
F2 - Acquisition Parameters
Date_ 20240208
Time 10.45
INSTRUM spect
PROBHD 5 mm CPQCI 1H-
PULPROG zgpg30
TD 65356
SOLVENT CDCl3
NS 4096
DS 4
SWH 40760.871 Hz
FIDRES 0.623675 Hz
AQ 0.8017003 sec
RG 182.53
DW 12.267 usec
DE 18.00 usec
TE 298.0 K
D1 2.00000000 sec
D11 0.03000000 sec
TD0 1
======== CHANNEL f1 ========
SFO1 176.1232717 MHz
NUC1 13C
P1 12.00 usec
PLW1 105.00000000 W
======== CHANNEL f2 ========
SFO2 700.3628014 MHz
NUC2 1H
CPDPRG[2 waltz16
PCPD2 65.00 usec
PLW2 9.89999962 W
PLW12 0.15564001 W
PLW13 0.07837200 W
F2 - Processing parameters
SI 131072
SF 176.1056398 MHz
WDW EM
SSB 0
LB 1.00 Hz
GB 0
PC 1.40

S12

^{1}H

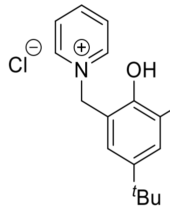
Cl
N
OH
O
tBu

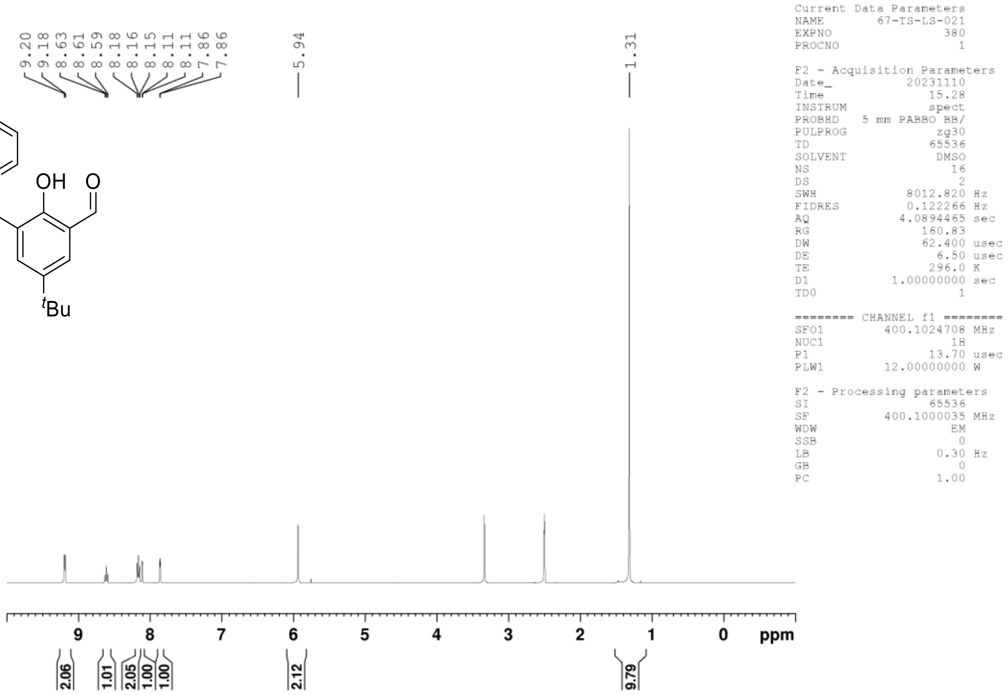
9.20
9.18
8.63
8.61
8.59
8.18
8.16
8.15
8.11
8.11
7.86
7.86
5.94
1.31
Current Data Parameters
NAME 67-TS-LS-021
EXPNO 380
PROCNO 1
F2 - Acquisition Parameters
Date_ 20231110
Time 15.28
INSTRUM spect
PROBHD 5 mm PABBO BB/
PULPROG zg30
TD 65536
SOLVENT DMSO
NS 16
DS 2
SWH 8012.820 Hz
FIDRES 0.122266 Hz
AQ 4.0894465 sec
RG 160.83
DW 62.400 usec
DE 6.50 usec
TE 296.0 K
D1 1.00000000 sec
TD0 1
======== CHANNEL f1 ========
SFO1 400.1024708 MHz
NUC1 1H
P1 13.70 usec
PLW1 12.00000000 W
F2 - Processing parameters
SI 65536
SF 400.1000035 MHz
WDW EM
SSB 0
LB 0.30 Hz
GB 0
PC 1.00
9
8
7
6
5
4
3
2
1
0
ppm
2.06
1.01
2.05
1.00
1.00
2.12
9.79

^{13}C

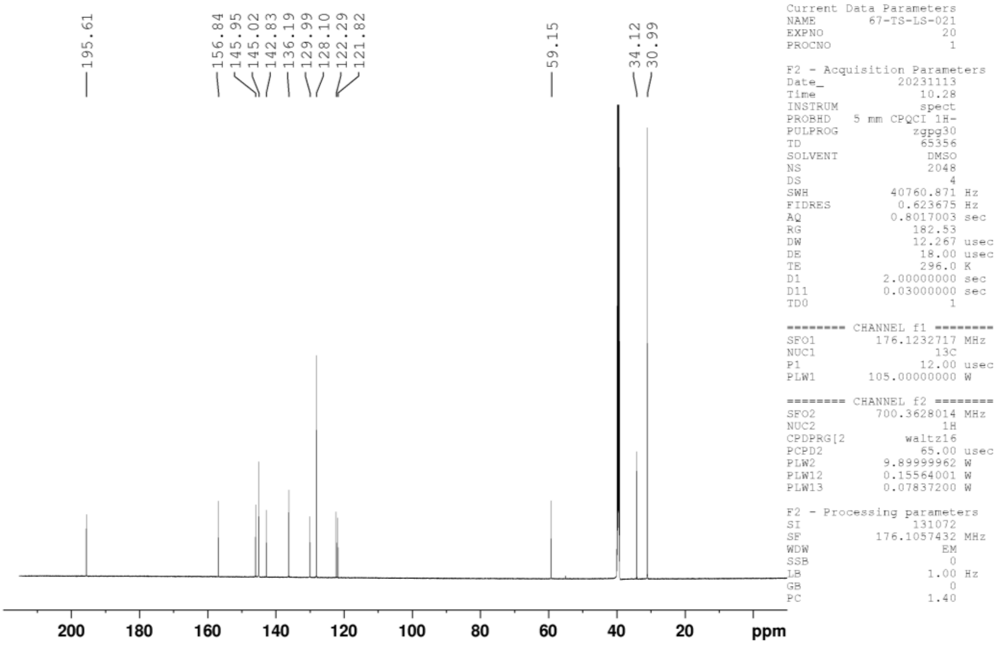
195.61
156.84
145.95
145.02
142.83
136.19
129.99
128.10
122.29
121.82
59.15
34.12
30.99
Current Data Parameters
NAME 67-TS-LS-021
EXPNO 20
PROCNO 1
F2 - Acquisition Parameters
Date_ 20231113
Time 10.28
INSTRUM spect
PROBHD 5 mm CPQCI 1H-
PULPROG zgpg30
TD 65356
SOLVENT DMSO
NS 2048
DS 4
SWH 40760.871 Hz
FIDRES 0.623675 Hz
AQ 0.8017003 sec
RG 182.53
DW 12.267 usec
DE 18.00 usec
TE 296.0 K
D1 2.00000000 sec
D11 0.03000000 sec
TD0 1
======== CHANNEL f1 ========
SFO1 176.1232717 MHz
NUC1 13C
P1 12.00 usec
PLW1 105.00000000 W
======== CHANNEL f2 ========
SFO2 700.3628014 MHz
NUC2 1H
CPDPRG[2 waltz16
PCPD2 65.00 usec
PLW2 9.89999962 W
PLW12 0.15564001 W
PLW13 0.07837200 W
F2 - Processing parameters
SI 131072
SF 176.1057432 MHz
WDW EM
SSB 0
LB 1.00 Hz
GB 0
PC 1.40
200
180
160
140
120
100
80
60
40
20
ppm

S42

^{1}H

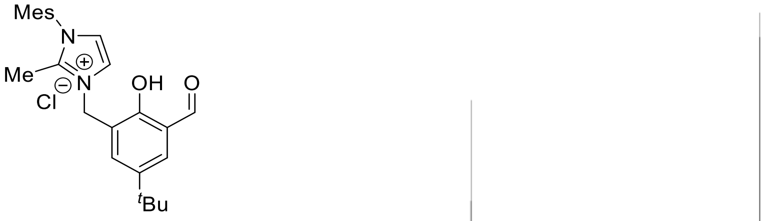

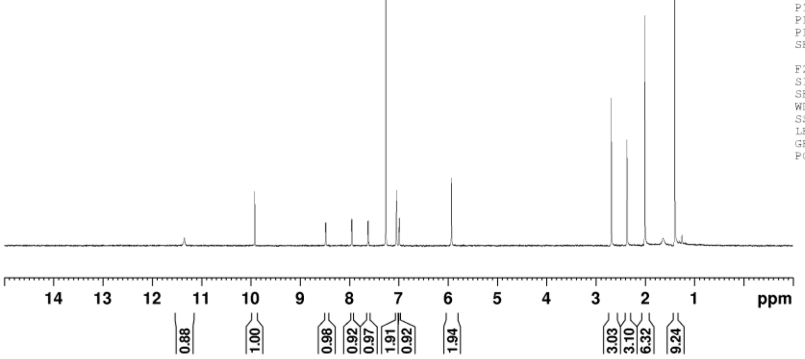


```
Current Data Parameters
NAME            45-LS020
EXPNO                 60
PROCNO                 1

F2 - Acquisition Parameters
Date_           20260317
Time               10.29
INSTRUM            spect
PROBHD   5 mm PABBO BB-
PULPROG             zg30
TD                 32768
SOLVENT            CDCl3
NS                    16
DS                     2
SWH             6188.119 Hz
FIDRES          0.188846 Hz
AQ             2.6476543 sec
RG                   812
DW                80.800 usec
DE                  8.00 usec
TE                 296.0 K
D1            1.00000000 sec
TD0                    1

======== CHANNEL f1 ========
NUC1                  1H
P1                 11.05 usec
PL1                -2.00 dB
PL1W         37.02396774 W
SFO1         300.1318534 MHz

F2 - Processing parameters
SI                 16384
SF           300.1300061 MHz
WDW                   EM
SSB                    0
LB                  0.30 Hz
GB                     0
PC                  1.00
```

^{13}C

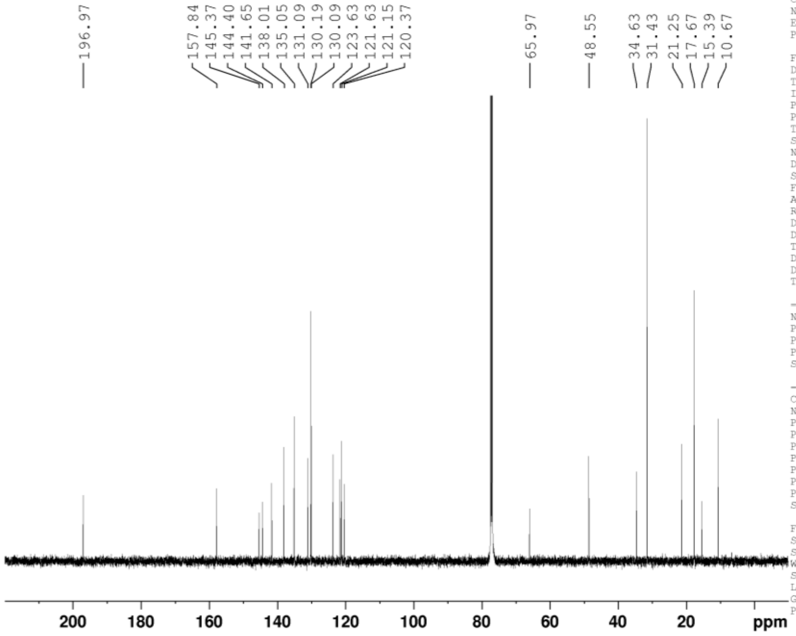


```
Current Data Parameters
NAME           45-LS-020
EXPNO                 40
PROCNO                 1

F2 - Acquisition Parameters
Date_           20160128
Time               17.28
INSTRUM            spect
PROBHD   5 mm PABBO BB-
PULPROG           zgpg30
TD                 65536
SOLVENT            CDCl3
NS                  2048
DS                     4
SWH            32894.738 Hz
FIDRES          0.501934 Hz
AQ             0.9961472 sec
RG                  2580
DW                15.200 usec
DE                 10.00 usec
TE                 296.8 K
D1            2.00000000 sec
D11           0.03000000 sec
TD0                    1

======== CHANNEL f1 ========
NUC1                 13C
P1                 10.20 usec
PL1                 1.50 dB
PL1W         51.74793243 W
SFO1         125.7761482 MHz

======== CHANNEL f2 ========
CPDPRG[2         waltz16
NUC2                  1H
PCPD2             100.00 usec
PL2                 1.00 dB
PL12               19.99 dB
PL13               21.00 dB
PL2W         19.75309753 W
PL12W         0.24925002 W
PL13W         0.19753097 W
SFO2         500.1550006 MHz

F2 - Processing parameters
SI                 32768
SF           125.7635582 MHz
WDW                   EM
SSB                    0
LB                  1.00 Hz
GB                     0
PC                  1.40
```

S18

^{1}H

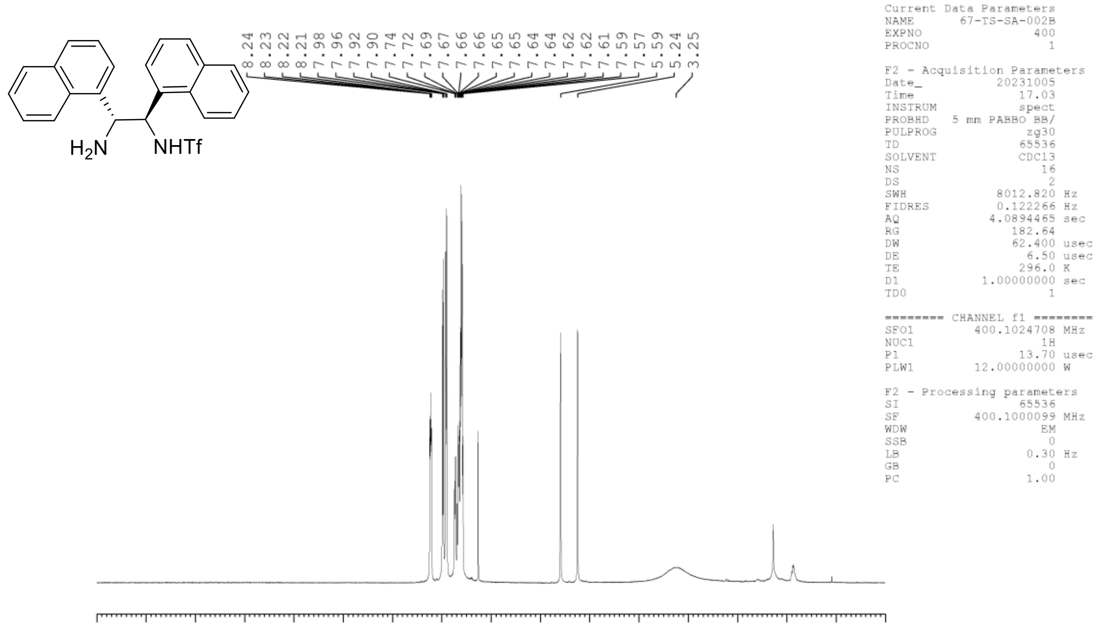
8.24
8.23
8.22
8.21
7.98
7.96
7.92
7.90
7.74
7.72
7.69
7.67
7.66
7.66
7.65
7.65
7.64
7.64
7.62
7.62
7.61
7.59
7.57
5.59
5.24
3.25
H2N
NHTf
Current Data Parameters
NAME 67-TS-SA-002B
EXPNO 400
PROCNO 1
F2 - Acquisition Parameters
Date_ 20231005
Time 17.03
INSTRUM spect
PROBHD 5 mm PABBO BB/
PULPROG zg30
TD 65536
SOLVENT CDCl3
NS 16
DS 2
SWH 8012.820 Hz
FIDRES 0.122266 Hz
AQ 4.0894465 sec
RG 182.64
DW 62.400 usec
DE 6.50 usec
TE 296.0 K
D1 1.00000000 sec
TD0 1
======== CHANNEL f1 ========
SFO1 400.1024708 MHz
NUC1 1H
P1 13.70 usec
PLW1 12.00000000 W
F2 - Processing parameters
SI 65536
SF 400.1000099 MHz
WDW EM
SSB 0
LB 0.30 Hz
GB 0
PC 1.00

^{13}C

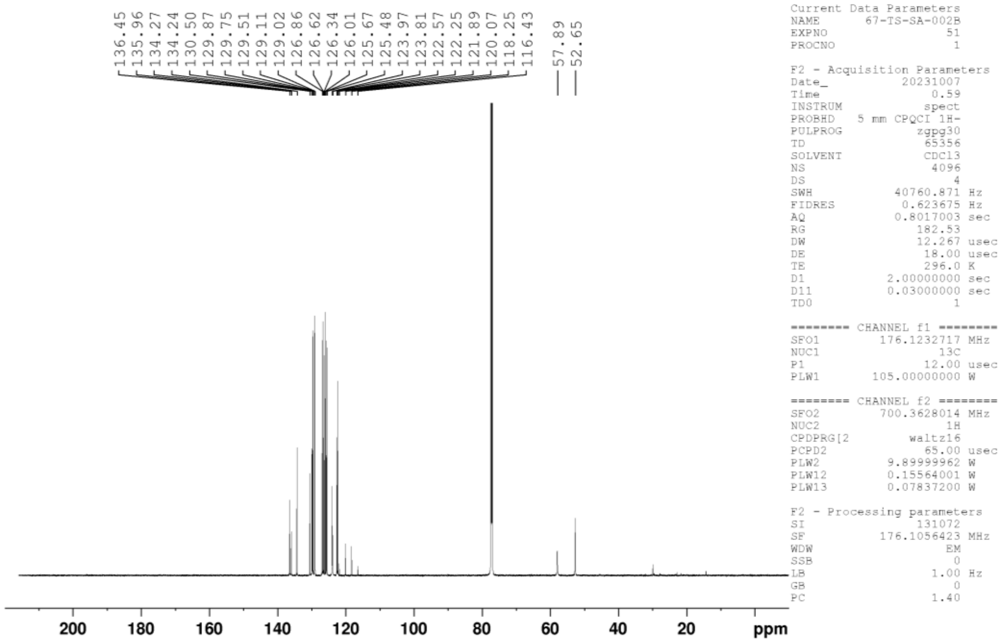
136.45
135.96
134.27
134.24
130.50
129.87
129.75
129.51
129.11
129.02
126.86
126.62
126.34
126.01
125.67
125.48
123.97
123.81
122.57
122.25
121.89
120.07
118.25
116.43
57.89
52.65
200
180
160
140
120
100
80
60
40
20
ppm
Current Data Parameters
NAME 67-TS-SA-002B
EXPNO 51
PROCNO 1
F2 - Acquisition Parameters
Date_ 20231007
Time 0.59
INSTRUM spect
PROBHD 5 mm CPQCI 1H-
PULPROG zgpg30
TD 65356
SOLVENT CDCl3
NS 4096
DS 4
SWH 40760.871 Hz
FIDRES 0.623675 Hz
AQ 0.8017003 sec
RG 182.53
DW 12.267 usec
DE 18.00 usec
TE 296.0 K
D1 2.00000000 sec
D11 0.03000000 sec
TD0 1
======== CHANNEL f1 ========
SFO1 176.1232717 MHz
NUC1 13C
P1 12.00 usec
PLW1 105.00000000 W
======== CHANNEL f2 ========
SFO2 700.3628014 MHz
NUC2 1H
CPDPRG[2 waltz16
PCPD2 65.00 usec
PLW2 9.89999962 W
PLW12 0.15564001 W
PLW13 0.07837200 W
F2 - Processing parameters
SI 131072
SF 176.1056423 MHz
WDW EM
SSB 0
LB 1.00 Hz
GB 0
PC 1.40

^{19}F

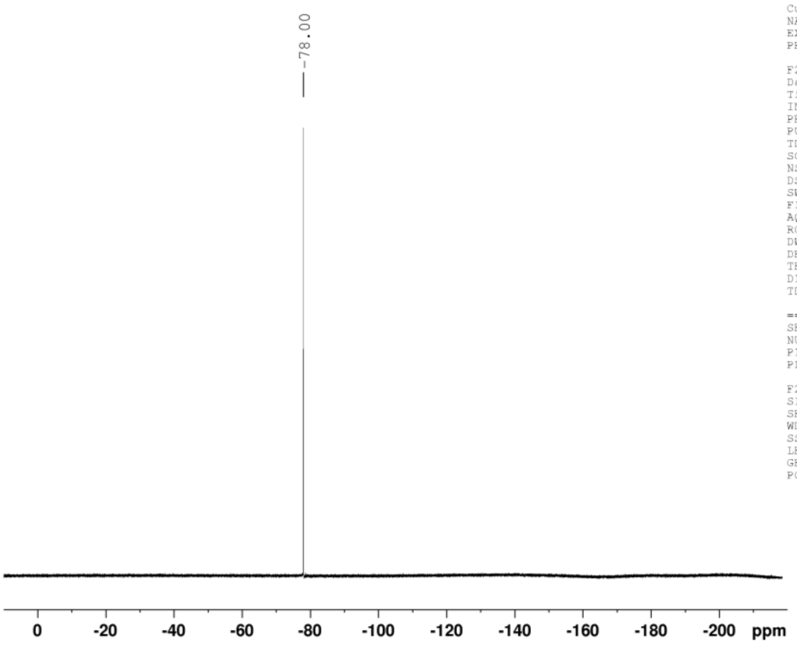


```
Current Data Parameters
NAME       67-TS-SA-002B
EXPNO                301
PROCNO                 1

F2 - Acquisition Parameters
Date_           20231005
Time               14.01
INSTRUM            spect
PROBHD   5 mm PABBO BB/
PULPROG          zgflqn
TD                131072
SOLVENT            CDCl3
NS                    16
DS                     4
SWH            89285.711 Hz
FIDRES          0.681196 Hz
AQ             0.7340032 sec
RG                205.35
DW                 5.600 usec
DE                  6.50 usec
TE                 296.0 K
D1            1.00000000 sec
TD0                    1

======== CHANNEL f1 ========
SFO1         376.4324910 MHz
NUC1                 19F
P1                 14.65 usec
PLW1         20.00000000 W

F2 - Processing parameters
SI                 65536
SF           376.4701380 MHz
WDW                   EM
SSB                    0
LB                  0.30 Hz
GB                     0
PC                  1.00
```

S19

^{1}H

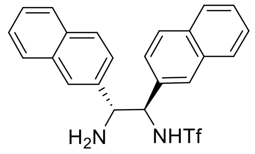

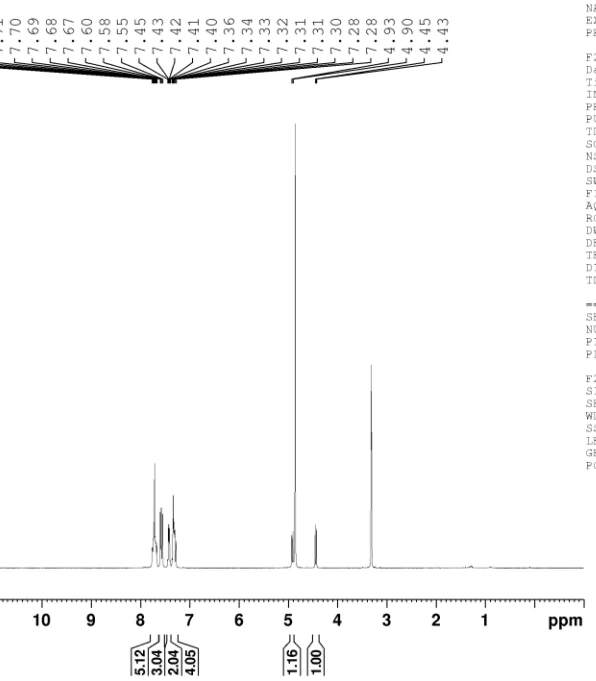


```
Current Data Parameters
NAME        67-TS-SA-003
EXPNO                410
PROCNO                 1

F2 - Acquisition Parameters
Date_           20231005
Time               17.22
INSTRUM            spect
PROBHD   5 mm PABBO BB/
PULPROG             zg30
TD                 65536
SOLVENT             MeOD
NS                    16
DS                     2
SWH             8012.820 Hz
FIDRES          0.122266 Hz
AQ             4.0894465 sec
RG                205.35
DW                62.400 usec
DE                  6.50 usec
TE                 296.0 K
D1            1.00000000 sec
TD0                    1

======== CHANNEL f1 ========
SFO1         400.1024708 MHz
NUC1                  1H
P1                 13.70 usec
PLW1         12.00000000 W

F2 - Processing parameters
SI                 65536
SF           400.1000078 MHz
WDW                   EM
SSB                    0
LB                  0.30 Hz
GB                     0
PC                  1.00
```

^{13}C

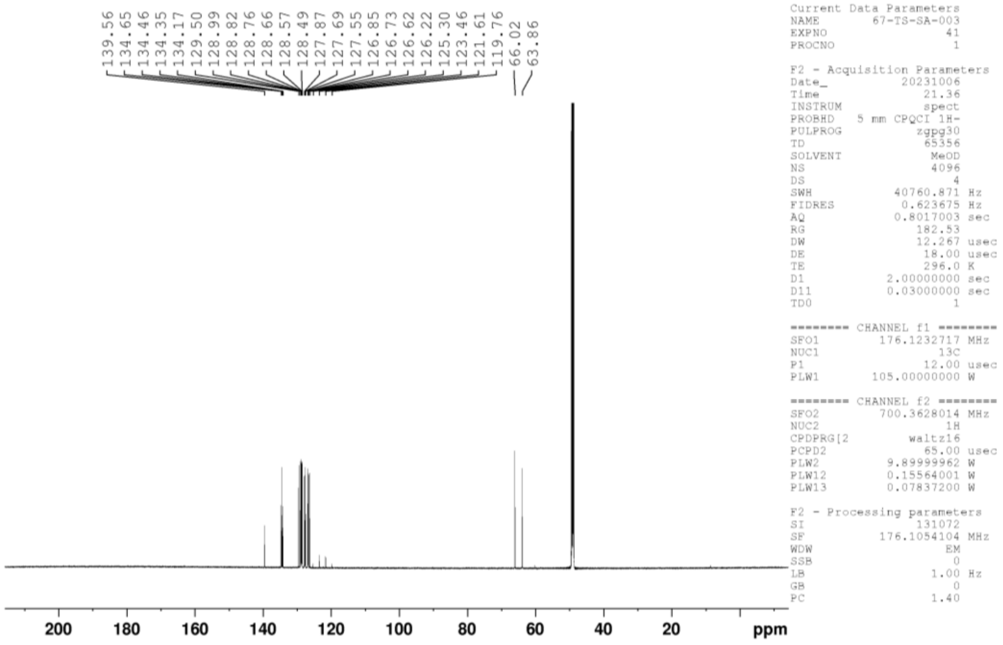
139.56
134.65
134.46
134.35
134.17
129.50
128.99
128.82
128.76
128.66
128.57
128.49
127.87
127.69
127.55
126.85
126.73
126.62
126.22
125.30
123.46
121.61
119.76
66.02
63.86
200 180 160 140 120 100 80 60 40 20 ppm
Current Data Parameters
NAME 67-TS-SA-003
EXPNO 41
PROCNO 1
F2 - Acquisition Parameters
Date_ 20231006
Time 21.36
INSTRUM spect
PROBHD 5 mm CPQCI 1H-
PULPROG zgpg30
TD 65356
SOLVENT MeOD
NS 4096
DS 4
SWH 40760.871 Hz
FIDRES 0.623675 Hz
AQ 0.8017003 sec
RG 182.53
DW 12.267 usec
DE 18.00 usec
TE 296.0 K
D1 2.00000000 sec
D11 0.03000000 sec
TD0 1
======== CHANNEL f1 ========
SFO1 176.1232717 MHz
NUC1 13C
P1 12.00 usec
PLW1 105.00000000 W
======== CHANNEL f2 ========
SFO2 700.3628014 MHz
NUC2 1H
CPDPRG[2 waltz16
PCPD2 65.00 usec
PLW2 9.89999962 W
PLW12 0.15564001 W
PLW13 0.07837200 W
F2 - Processing parameters
SI 131072
SF 176.1054104 MHz
WDW EM
SSB 0
LB 1.00 Hz
GB 0
PC 1.40

^{19}F

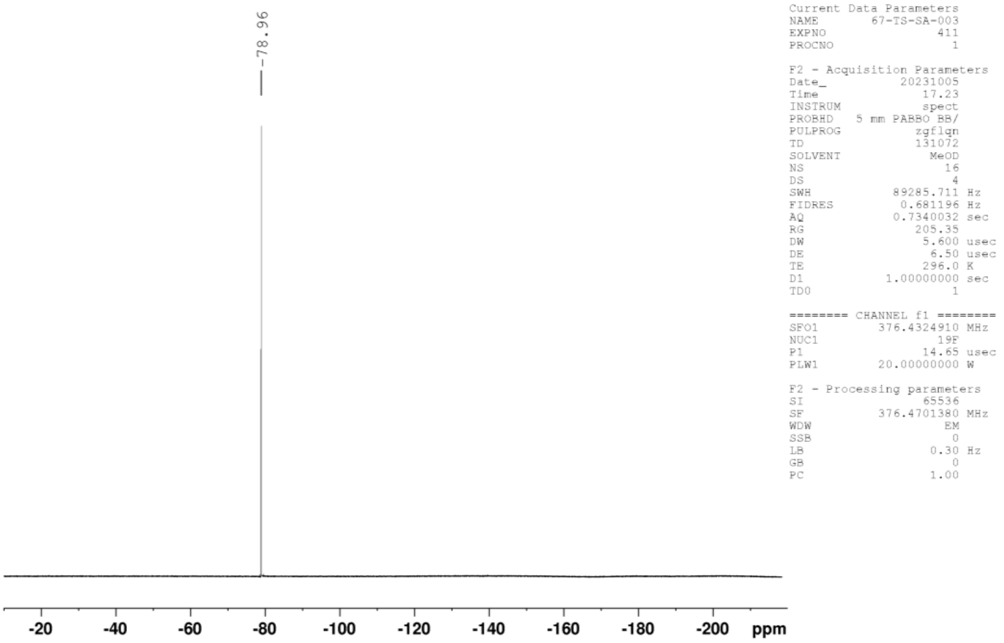
-78.96
-20 -40 -60 -80 -100 -120 -140 -160 -180 -200 ppm
Current Data Parameters
NAME 67-TS-SA-003
EXPNO 411
PROCNO 1
F2 - Acquisition Parameters
Date_ 20231005
Time 17.23
INSTRUM spect
PROBHD 5 mm PABBO BB/
PULPROG zgflqn
TD 131072
SOLVENT MeOD
NS 16
DS 4
SWH 89285.711 Hz
FIDRES 0.681196 Hz
AQ 0.7340032 sec
RG 205.35
DW 5.600 usec
DE 6.50 usec
TE 296.0 K
D1 1.00000000 sec
TD0 1
======== CHANNEL f1 ========
SFO1 376.4324910 MHz
NUC1 19F
P1 14.65 usec
PLW1 20.00000000 W
F2 - Processing parameters
SI 65536
SF 376.4701380 MHz
WDW EM
SSB 0
LB 0.30 Hz
GB 0
PC 1.00

S20

^{1}H

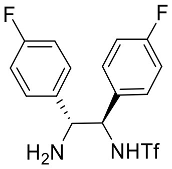

F
F
H_2N
NHTf

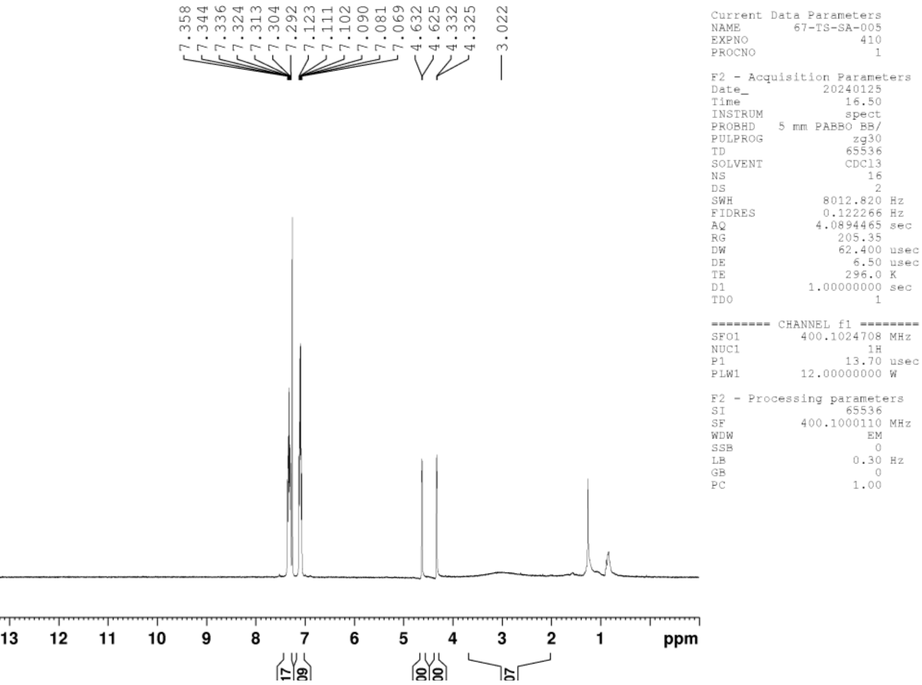

7.358
7.344
7.336
7.324
7.313
7.304
7.292
7.123
7.111
7.102
7.090
7.081
7.069
4.632
4.625
4.332
4.325
3.022
14 13 12 11 10 9 8 7 6 5 4 3 2 1 ppm
Current Data Parameters
NAME 67-TS-SA-005
EXPNO 410
PROCNO 1
F2 - Acquisition Parameters
Date_ 20240125
Time 16.50
INSTRUM spect
PROBHD 5 mm PABBO BB/
PULPROG zg30
TD 65536
SOLVENT CDCl3
NS 16
DS 2
SWH 8012.820 Hz
FIDRES 0.122266 Hz
AQ 4.0894465 sec
RG 205.35
DW 62.400 usec
DE 6.50 usec
TE 296.0 K
D1 1.00000000 sec
TD0 1
======== CHANNEL f1 ========
SFO1 400.1024708 MHz
NUC1 1H
P1 13.70 usec
PLW1 12.00000000 W
F2 - Processing parameters
SI 65536
SF 400.1000110 MHz
WDW EM
SSB 0
LB 0.30 Hz
GB 0
PC 1.00

^{13}C

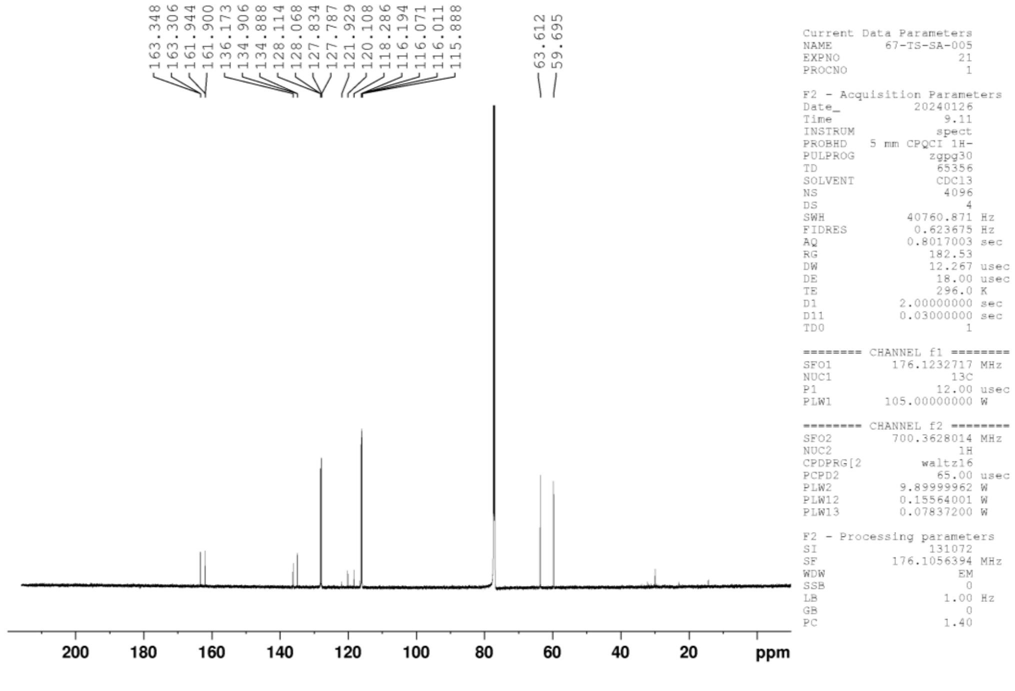

163.348
163.306
161.944
161.900
136.173
134.906
134.888
128.114
128.068
127.834
127.787
121.929
120.108
118.286
116.194
116.071
116.011
115.888
63.612
59.695
200 180 160 140 120 100 80 60 40 20 ppm
Current Data Parameters
NAME 67-TS-SA-005
EXPNO 21
PROCNO 1
F2 - Acquisition Parameters
Date_ 20240126
Time 9.11
INSTRUM spect
PROBHD 5 mm CPQCI 1H-
PULPROG zgpg30
TD 65356
SOLVENT CDCl3
NS 4096
DS 4
SWH 40760.871 Hz
FIDRES 0.623675 Hz
AQ 0.8017003 sec
RG 182.53
DW 12.267 usec
DE 18.00 usec
TE 296.0 K
D1 2.00000000 sec
D11 0.03000000 sec
TD0 1
======== CHANNEL f1 ========
SFO1 176.1232717 MHz
NUC1 13C
P1 12.00 usec
PLW1 105.00000000 W
======== CHANNEL f2 ========
SFO2 700.3628014 MHz
NUC2 1H
CPDPRG[2 waltz16
PCPD2 65.00 usec
PLW2 9.89999962 W
PLW12 0.15564001 W
PLW13 0.07837200 W
F2 - Processing parameters
SI 131072
SF 176.1056394 MHz
WDW EM
SSB 0
LB 1.00 Hz
GB 0
PC 1.40

^{19}F

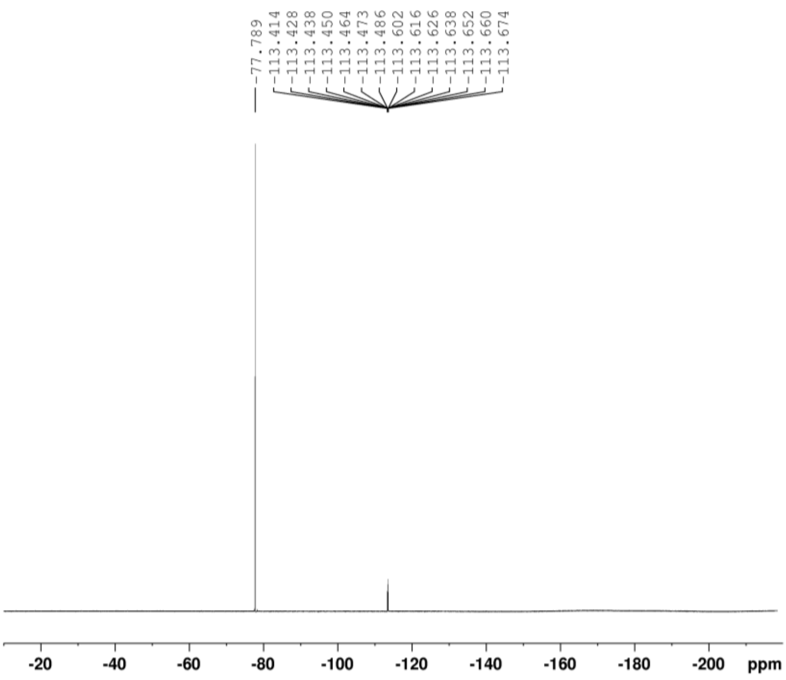


```
Current Data Parameters
NAME        67-TS-SA-005
EXPNO                231
PROCNO                 1

F2 - Acquisition Parameters
Date_           20240125
Time               13.46
INSTRUM            spect
PROBHD   5 mm PABBO BB/
PULPROG          zgflqn
TD                131072
SOLVENT            CDCl3
NS                    16
DS                     4
SWH            89285.711 Hz
FIDRES          0.681196 Hz
AQ             0.7340032 sec
RG                205.35
DW                 5.600 usec
DE                  6.50 usec
TE                 296.0 K
D1            1.00000000 sec
TD0                    1

======== CHANNEL f1 ========
SFO1         376.4324910 MHz
NUC1                 19F
P1                 14.65 usec
PLW1         20.00000000 W

F2 - Processing parameters
SI                 65536
SF           376.4701380 MHz
WDW                   EM
SSB                    0
LB                  0.30 Hz
GB                     0
PC                  1.00
```

S21

^{1}H

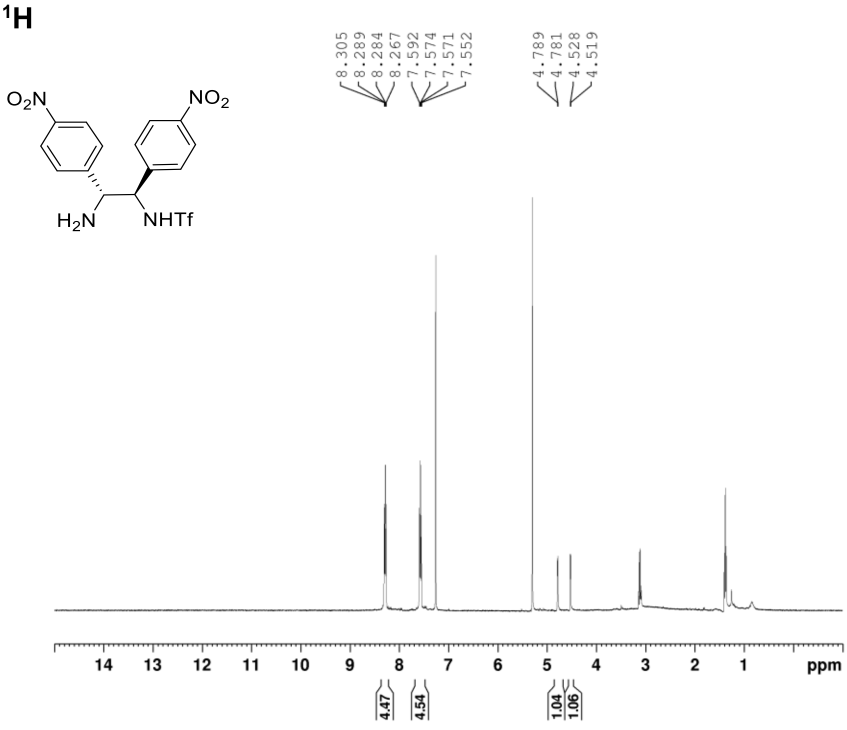


```
Current Data Parameters
NAME        67-TS-SA-010
EXPNO                430
PROCNO                 1

F2 - Acquisition Parameters
Date_           20240416
Time               15.13
INSTRUM            spect
PROBHD   5 mm PABBO BB/
PULPROG             zg30
TD                 65536
SOLVENT            CDCl3
NS                    16
DS                     2
SWH             8012.820 Hz
FIDRES          0.122266 Hz
AQ             4.0894465 sec
RG                205.35
DW                62.400 usec
DE                  6.50 usec
TE                 296.0 K
D1            1.00000000 sec
TD0                    1

======== CHANNEL f1 ========
SFO1         400.1024708 MHz
NUC1                  1H
P1                 13.70 usec
PLW1         12.00000000 W

F2 - Processing parameters
SI                 65536
SF           400.1000090 MHz
WDW                   EM
SSB                    0
LB                  0.30 Hz
GB                     0
PC                  1.00
```

^{13}C

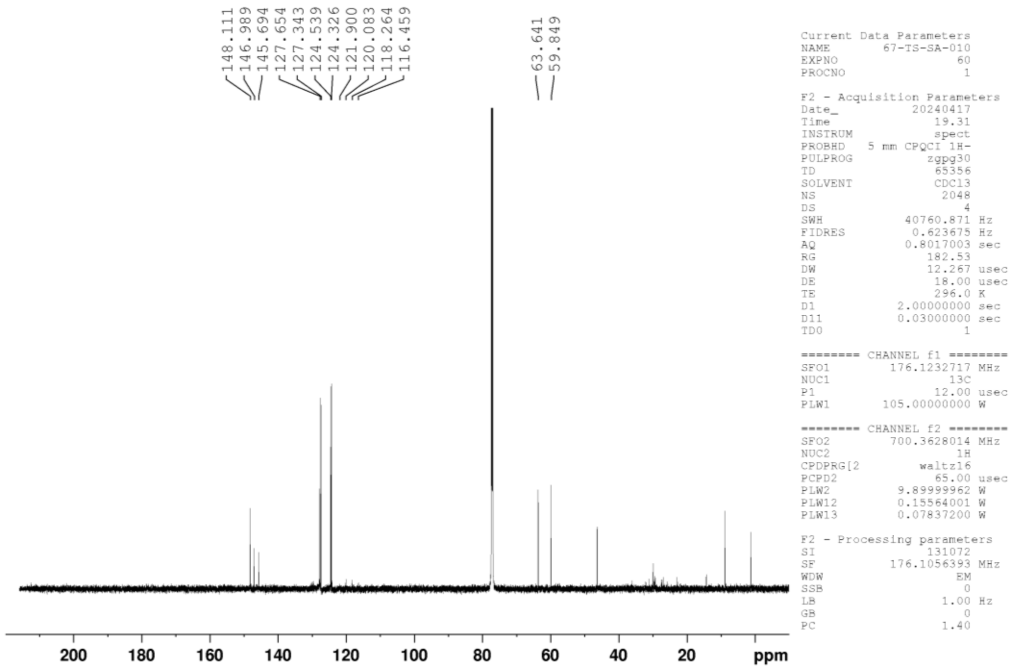
148.111
146.989
145.694
127.654
127.343
124.539
124.326
121.900
120.083
118.264
116.459
63.641
59.849
Current Data Parameters
NAME 67-TS-SA-010
EXPNO 60
PROCNO 1
F2 - Acquisition Parameters
Date_ 20240417
Time 19.31
INSTRUM spect
PROBHD 5 mm CPQCI 1H-
PULPROG zgpg30
TD 65356
SOLVENT CDCl3
NS 2048
DS 4
SWH 40760.871 Hz
FIDRES 0.623675 Hz
AQ 0.8017003 sec
RG 182.53
DW 12.267 usec
DE 18.00 usec
TE 296.0 K
D1 2.00000000 sec
D11 0.03000000 sec
TD0 1
======== CHANNEL f1 ========
SFO1 176.1232717 MHz
NUC1 13C
P1 12.00 usec
PLW1 105.00000000 W
======== CHANNEL f2 ========
SFO2 700.3628014 MHz
NUC2 1H
CPDPRG[2 waltz16
PCPD2 65.00 usec
PLW2 9.89999962 W
PLW12 0.15564001 W
PLW13 0.07837200 W
F2 - Processing parameters
SI 131072
SF 176.1056393 MHz
WDW EM
SSB 0
LB 1.00 Hz
GB 0
PC 1.40
200 180 160 140 120 100 80 60 40 20 ppm

^{19}F

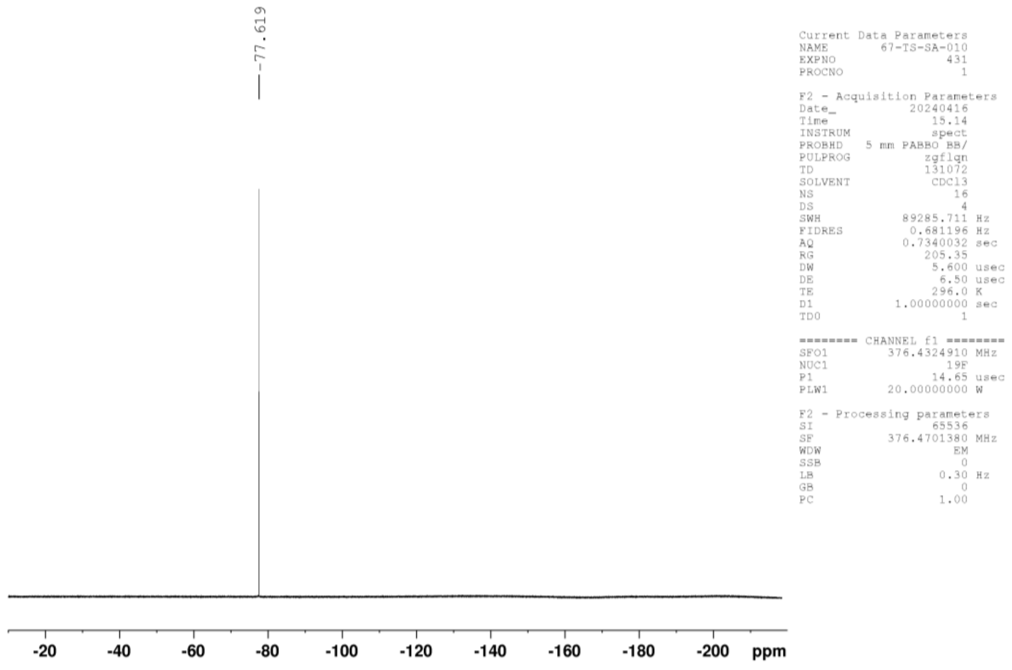
-77.619
Current Data Parameters
NAME 67-TS-SA-010
EXPNO 431
PROCNO 1
F2 - Acquisition Parameters
Date_ 20240416
Time 15.14
INSTRUM spect
PROBHD 5 mm PABBO BB/
PULPROG zgflqn
TD 131072
SOLVENT CDCl3
NS 16
DS 4
SWH 89285.711 Hz
FIDRES 0.681196 Hz
AQ 0.7340032 sec
RG 205.35
DW 5.600 usec
DE 6.50 usec
TE 296.0 K
D1 1.00000000 sec
TD0 1
======== CHANNEL f1 ========
SFO1 376.4324910 MHz
NUC1 19F
P1 14.65 usec
PLW1 20.00000000 W
F2 - Processing parameters
SI 65536
SF 376.4701380 MHz
WDW EM
SSB 0
LB 0.30 Hz
GB 0
PC 1.00
-20 -40 -60 -80 -100 -120 -140 -160 -180 -200 ppm

S24

^{1}H

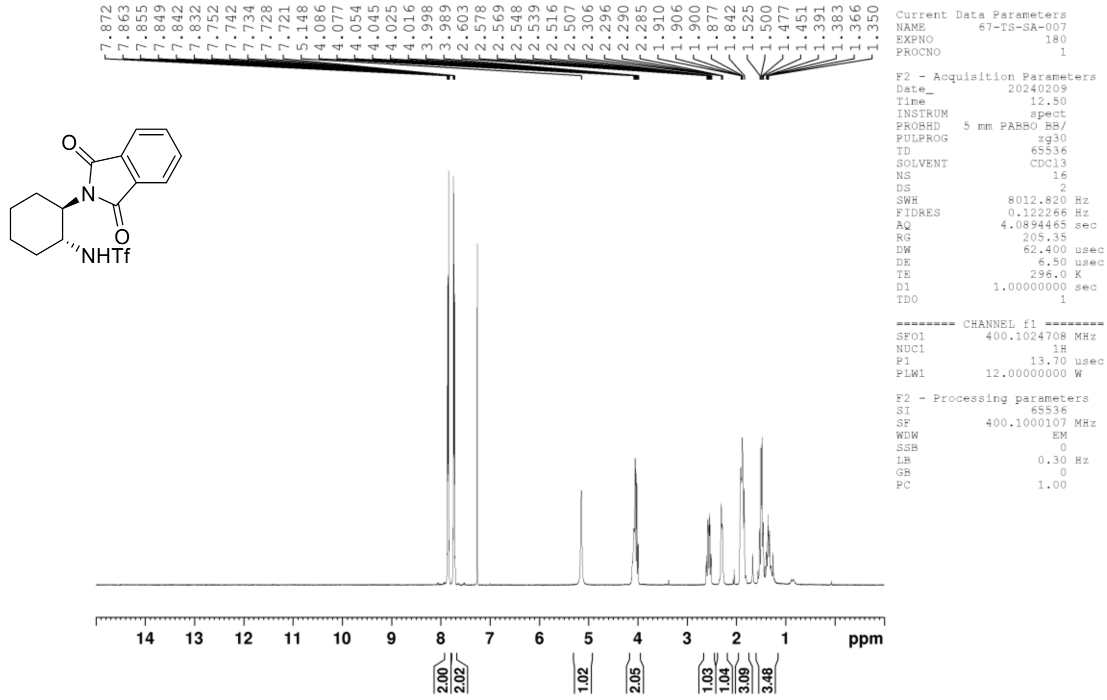

O
N
O
NHTf
7.872 7.863 7.855 7.849 7.842 7.832 7.752 7.742 7.734 7.728 7.721 5.148 4.086 4.077 4.054 4.045 4.025 4.016 3.998 3.989 2.603 2.578 2.569 2.548 2.539 2.516 2.507 2.306 2.296 2.290 2.285 1.910 1.906 1.900 1.877 1.842 1.525 1.500 1.477 1.451 1.391 1.383 1.366 1.350
14 13 12 11 10 9 8 7 6 5 4 3 2 1 ppm
2.00 2.02 1.02 2.05 1.03 1.04 3.09 3.48
Current Data Parameters
NAME 67-TS-SA-007
EXPNO 180
PROCNO 1
F2 - Acquisition Parameters
Date_ 20240209
Time 12.50
INSTRUM spect
PROBHD 5 mm PABBO BB/
PULPROG zg30
TD 65536
SOLVENT CDCl3
NS 16
DS 2
SWH 8012.820 Hz
FIDRES 0.122266 Hz
AQ 4.0894465 sec
RG 205.35
DW 62.400 usec
DE 6.50 usec
TE 296.0 K
D1 1.00000000 sec
TD0 1
======== CHANNEL f1 ========
SFO1 400.1024708 MHz
NUC1 1H
P1 13.70 usec
PLW1 12.00000000 W
F2 - Processing parameters
SI 65536
SF 400.1000107 MHz
WDW EM
SSB 0
LB 0.30 Hz
GB 0
PC 1.00

^{13}C

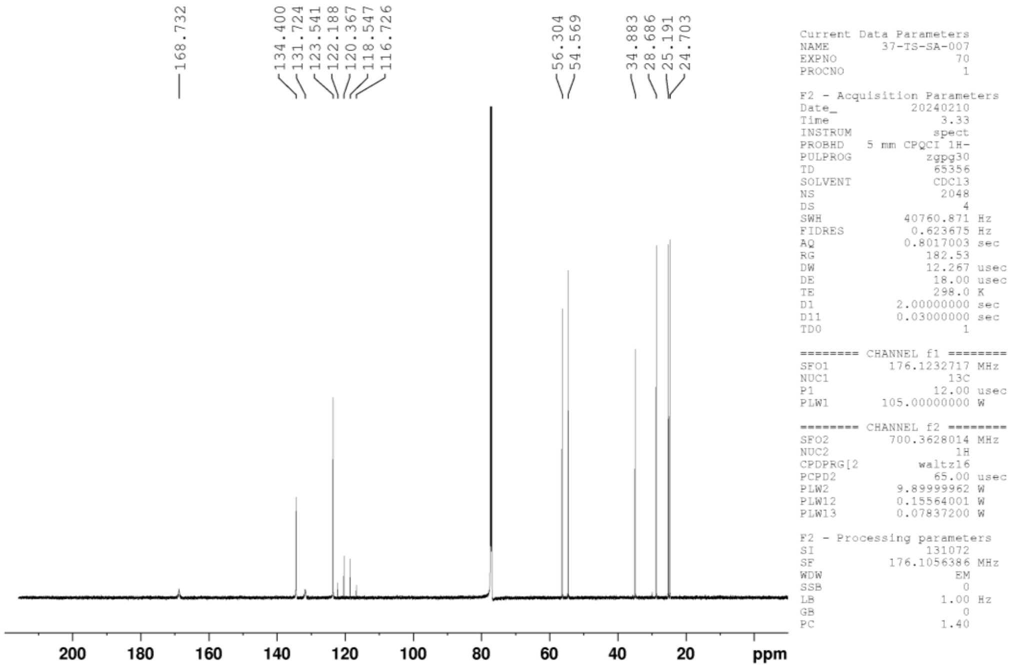

168.732
134.400 131.724 123.541 122.188 120.367 118.547 116.726
56.304 54.569
34.883 28.686 25.191 24.703
200 180 160 140 120 100 80 60 40 20 ppm
Current Data Parameters
NAME 37-TS-SA-007
EXPNO 70
PROCNO 1
F2 - Acquisition Parameters
Date_ 20240210
Time 3.33
INSTRUM spect
PROBHD 5 mm CPQCI 1H-
PULPROG zgpg30
TD 65356
SOLVENT CDCl3
NS 2048
DS 4
SWH 40760.871 Hz
FIDRES 0.623675 Hz
AQ 0.8017003 sec
RG 182.53
DW 12.267 usec
DE 18.00 usec
TE 298.0 K
D1 2.00000000 sec
D11 0.03000000 sec
TD0 1
======== CHANNEL f1 ========
SFO1 176.1232717 MHz
NUC1 13C
P1 12.00 usec
PLW1 105.00000000 W
======== CHANNEL f2 ========
SFO2 700.3628014 MHz
NUC2 1H
CPDPRG[2 waltz16
PCPD2 65.00 usec
PLW2 9.89999962 W
PLW12 0.15564001 W
PLW13 0.07837200 W
F2 - Processing parameters
SI 131072
SF 176.1056386 MHz
WDW EM
SSB 0
LB 1.00 Hz
GB 0
PC 1.40

^{19}F

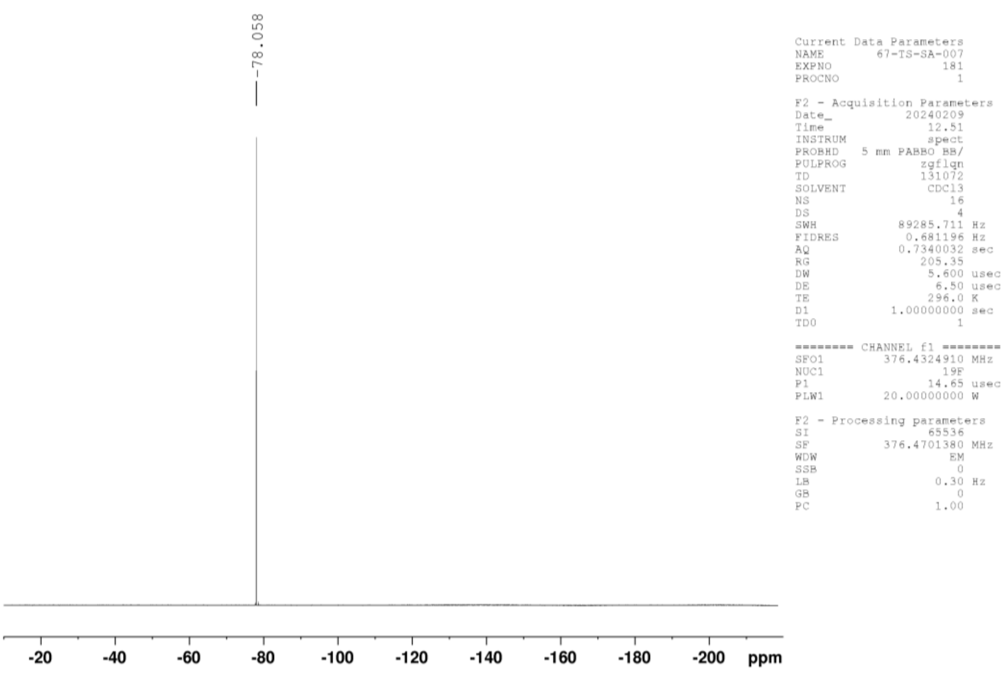

S28

^{1}H

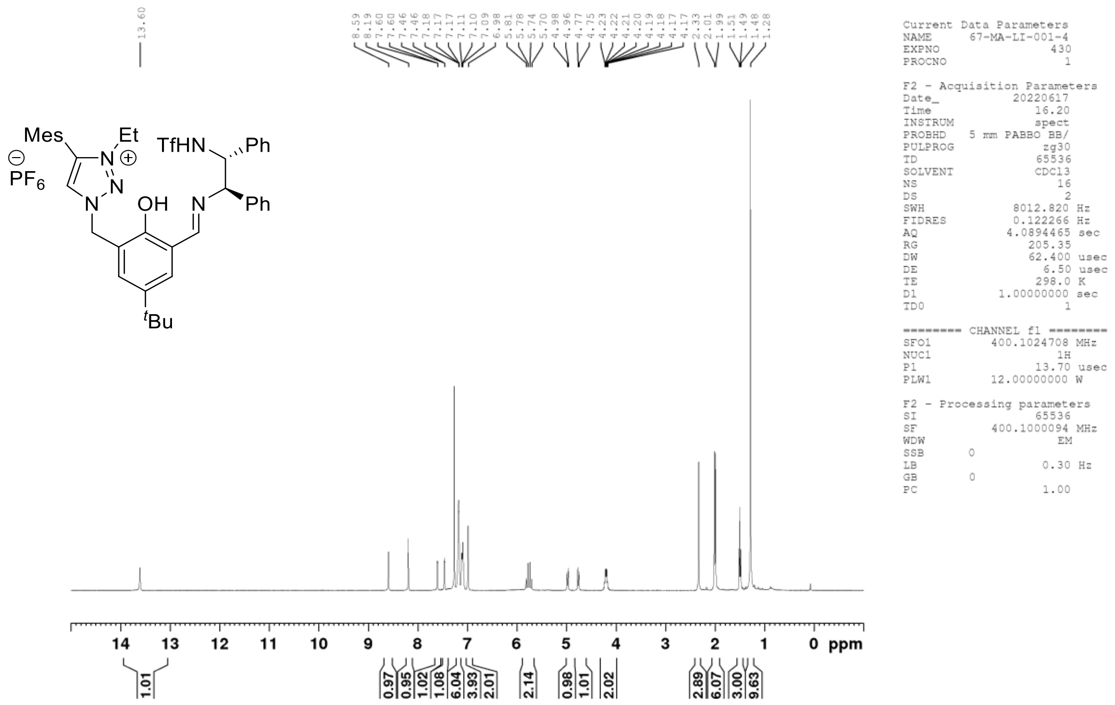

^{13}C

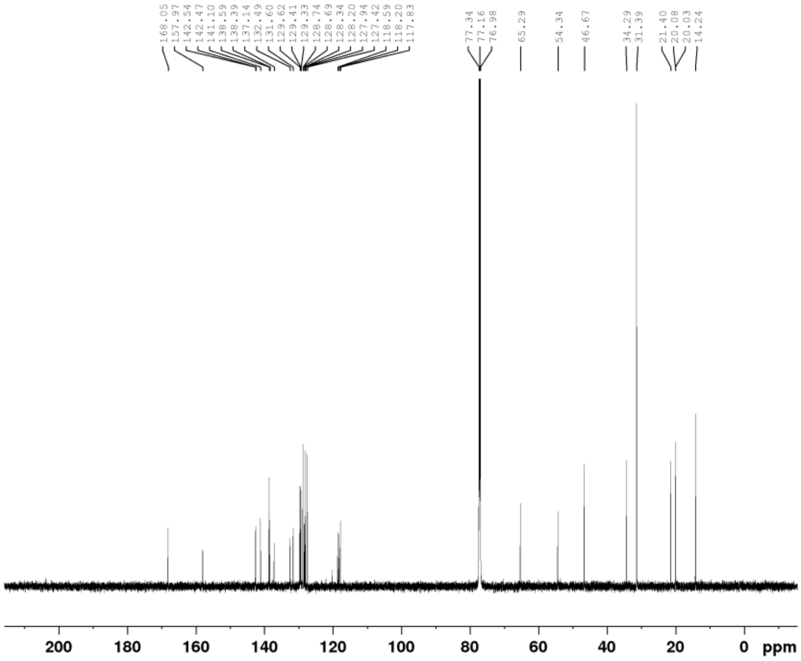


```
Current Data Parameters
NAME      67-MA-LI-001
EXPNO              140
PROCNO               1

F2 - Acquisition Parameters
Date_         20220513
Time              3.02
INSTRUM          spect
PROBHD   5 mm CPQCI 1H-
PULPROG         zgpg30
TD               65356
SOLVENT          CDCl3
NS                1024
DS                   4
SWH          40760.871 Hz
FIDRES        0.623675 Hz
AQ           0.8017003 sec
RG              182.53
DW              12.267 usec
DE               18.00 usec
TE               296.0 K
D1          2.00000000 sec
D11         0.03000000 sec
TD0                  1

======== CHANNEL f1 ========
SFO1       176.1232717 MHz
NUC1               13C
P1               12.00 usec
PLW1      105.00000000 W

======== CHANNEL f2 ========
SFO2       700.3628014 MHz
NUC2                1H
CPDPRG[2        waltz16
PCPD2            65.00 usec
PLW2        9.89999962 W
PLW12       0.15564001 W
PLW13       0.07837200 W

F2 - Processing parameters
SI              131072
SF         176.1056397 MHz
WDW                 EM
SSB      0
LB                1.00 Hz
GB       0
PC                1.40
```

^{19}F

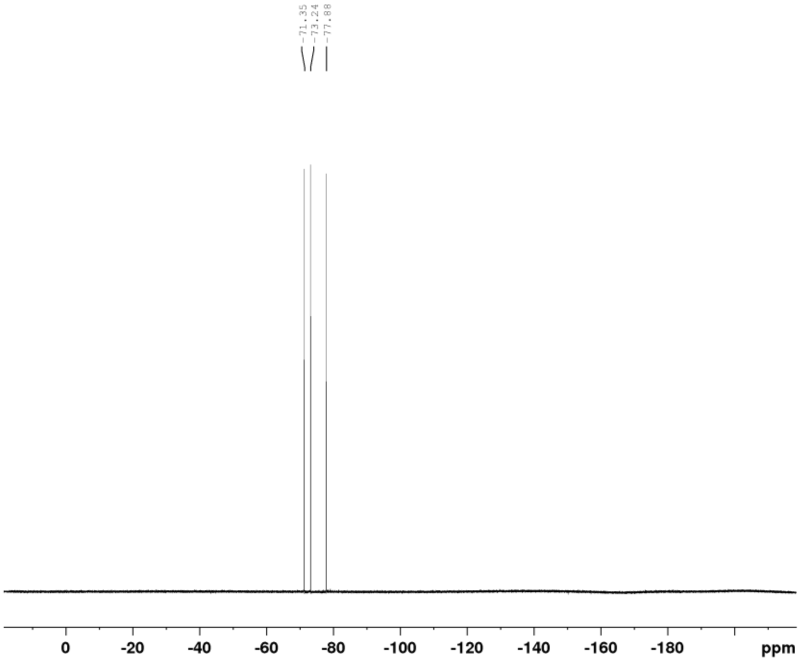


```
Current Data Parameters
NAME    67-MA-LI-001-2
EXPNO              291
PROCNO               1

F2 - Acquisition Parameters
Date_         20220427
Time             13.44
INSTRUM          spect
PROBHD   5 mm PABBO BB/
PULPROG         zgflqn
TD              131072
SOLVENT          CDCl3
NS                  16
DS                   4
SWH          89285.711 Hz
FIDRES        0.681196 Hz
AQ           0.7340032 sec
RG              205.35
DW               5.600 usec
DE                6.50 usec
TE               298.0 K
D1          1.00000000 sec
TD0                  1

======== CHANNEL f1 ========
SFO1       376.4324910 MHz
NUC1               19F
P1               14.65 usec
PLW1       20.00000000 W

F2 - Processing parameters
SI               65536
SF         376.4701380 MHz
WDW                 EM
SSB      0
LB                0.30 Hz
GB       0
PC                1.00
```

S30

^{1}H

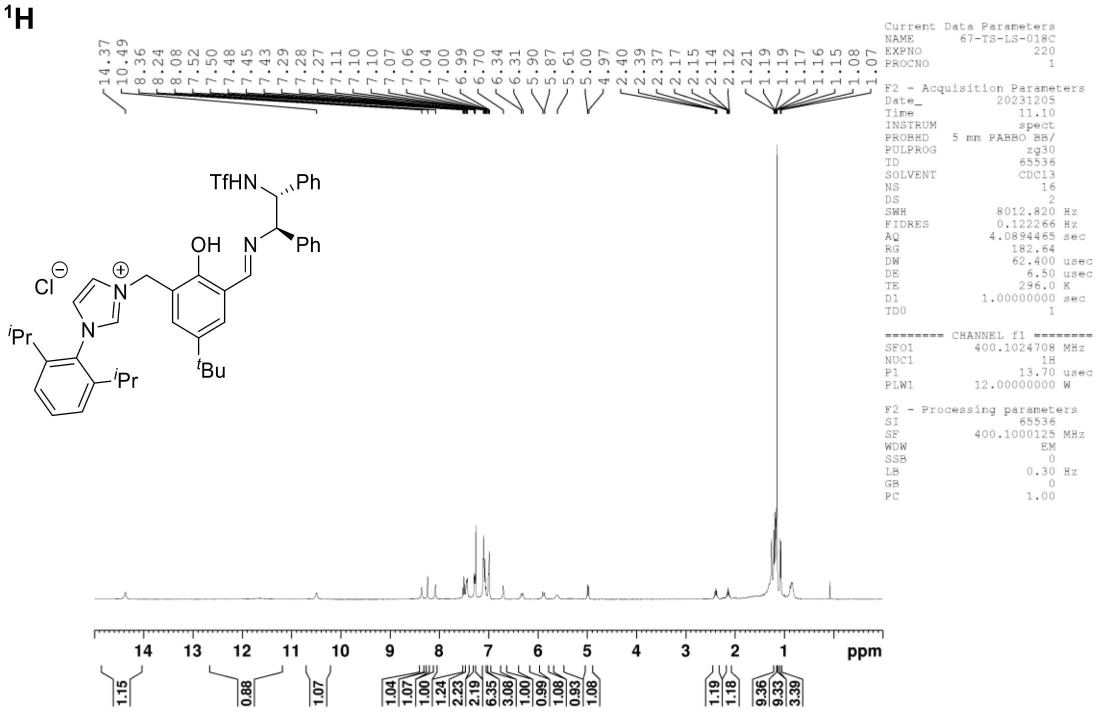

14.37
10.49
8.36
8.24
8.08
7.52
7.50
7.48
7.45
7.43
7.29
7.28
7.27
7.11
7.10
7.10
7.07
7.06
7.04
7.00
6.99
6.70
6.34
6.31
5.90
5.87
5.61
5.00
4.97
2.40
2.39
2.37
2.17
2.15
2.14
2.12
1.21
1.19
1.19
1.17
1.16
1.15
1.08
1.07
TfHN
Ph
OH
N
Ph
Cl
N
N
iPr
iPr
tBu
Current Data Parameters
NAME 67-TS-LS-018C
EXPNO 220
PROCNO 1
F2 - Acquisition Parameters
Date_ 20231205
Time 11.10
INSTRUM spect
PROBHD 5 mm PABBO BB/
PULPROG zg30
TD 65536
SOLVENT CDCl3
NS 16
DS 2
SWH 8012.820 Hz
FIDRES 0.122266 Hz
AQ 4.0894465 sec
RG 182.64
DW 62.400 usec
DE 6.50 usec
TE 296.0 K
D1 1.00000000 sec
TD0 1
======== CHANNEL f1 ========
SFO1 400.1024708 MHz
NUC1 1H
P1 13.70 usec
PLW1 12.00000000 W
F2 - Processing parameters
SI 65536
SF 400.1000125 MHz
WDW EM
SSB 0
LB 0.30 Hz
GB 0
PC 1.00
14 13 12 11 10 9 8 7 6 5 4 3 2 1 ppm
1.15
0.88
1.07
1.04
1.07
1.00
1.24
2.23
2.19
6.35
3.08
1.00
0.99
1.08
0.93
1.08
1.19
1.18
9.36
9.33
3.39

^{13}C

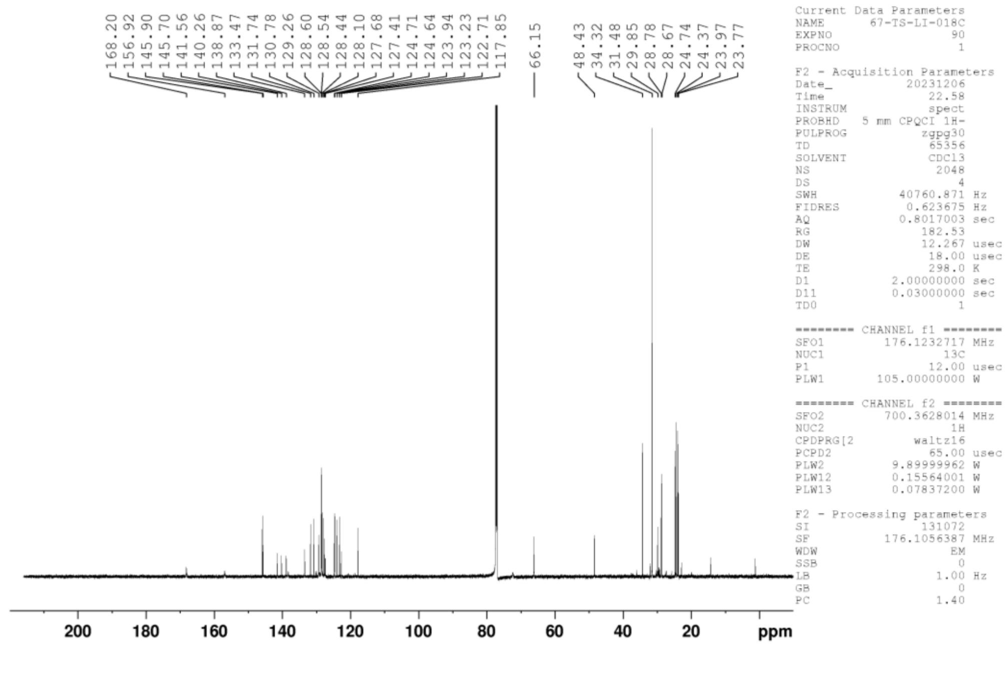

168.20
156.92
145.90
145.70
141.56
140.26
138.87
133.47
131.74
130.78
129.26
128.60
128.54
128.44
128.10
127.68
127.41
124.71
124.64
123.94
123.23
122.71
117.85
66.15
48.43
34.32
31.48
29.85
28.78
28.67
24.74
24.37
23.97
23.77
Current Data Parameters
NAME 67-TS-LI-018C
EXPNO 90
PROCNO 1
F2 - Acquisition Parameters
Date_ 20231206
Time 22.58
INSTRUM spect
PROBHD 5 mm CPQCI 1H-
PULPROG zgpg30
TD 65356
SOLVENT CDCl3
NS 2048
DS 4
SWH 40760.871 Hz
FIDRES 0.623675 Hz
AQ 0.8017003 sec
RG 182.53
DW 12.267 usec
DE 18.00 usec
TE 298.0 K
D1 2.00000000 sec
D11 0.03000000 sec
TD0 1
======== CHANNEL f1 ========
SFO1 176.1232717 MHz
NUC1 13C
P1 12.00 usec
PLW1 105.00000000 W
======== CHANNEL f2 ========
SFO2 700.3628014 MHz
NUC2 1H
CPDPRG[2 waltz16
PCPD2 65.00 usec
PLW2 9.89999962 W
PLW12 0.15564001 W
PLW13 0.07837200 W
F2 - Processing parameters
SI 131072
SF 176.1056387 MHz
WDW EM
SSB 0
LB 1.00 Hz
GB 0
PC 1.40
200 180 160 140 120 100 80 60 40 20 ppm

^{19}F

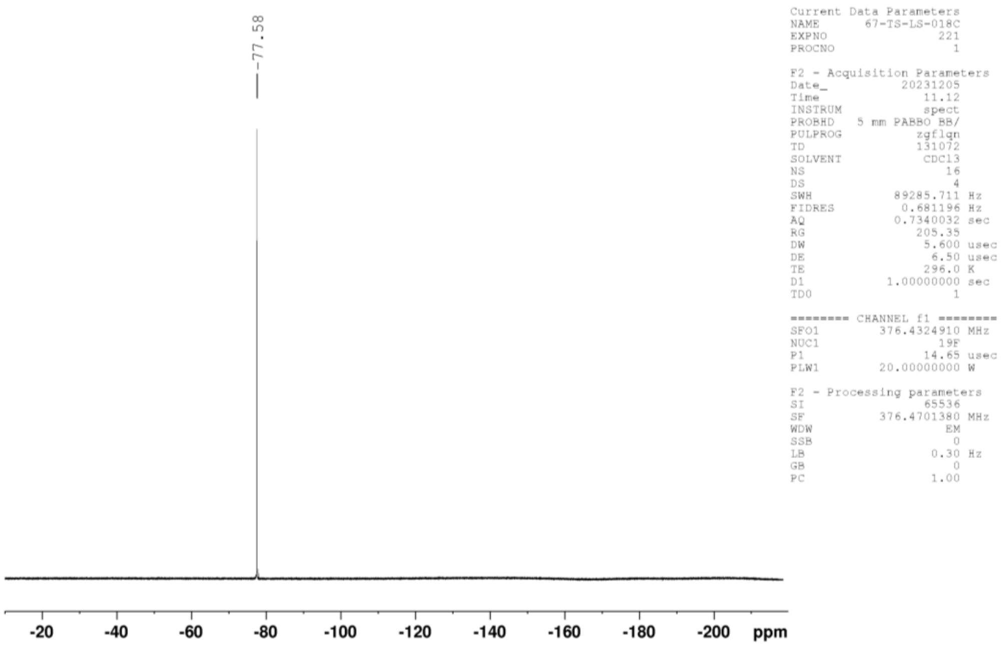
-77.58
-20
-40
-60
-80
-100
-120
-140
-160
-180
-200
ppm
Current Data Parameters
NAME 67-TS-LS-018C
EXPNO 221
PROCNO 1
F2 - Acquisition Parameters
Date_ 20231205
Time 11.12
INSTRUM spect
PROBHD 5 mm PABBO BB/
PULPROG zgflqn
TD 131072
SOLVENT CDCl3
NS 16
DS 4
SWH 89285.711 Hz
FIDRES 0.681196 Hz
AQ 0.7340032 sec
RG 205.35
DW 5.600 usec
DE 6.50 usec
TE 296.0 K
D1 1.00000000 sec
TD0 1
======== CHANNEL f1 ========
SFO1 376.4324910 MHz
NUC1 19F
P1 14.65 usec
PLW1 20.00000000 W
F2 - Processing parameters
SI 65536
SF 376.4701380 MHz
WDW EM
SSB 0
LB 0.30 Hz
GB 0
PC 1.00

8

^{1}H

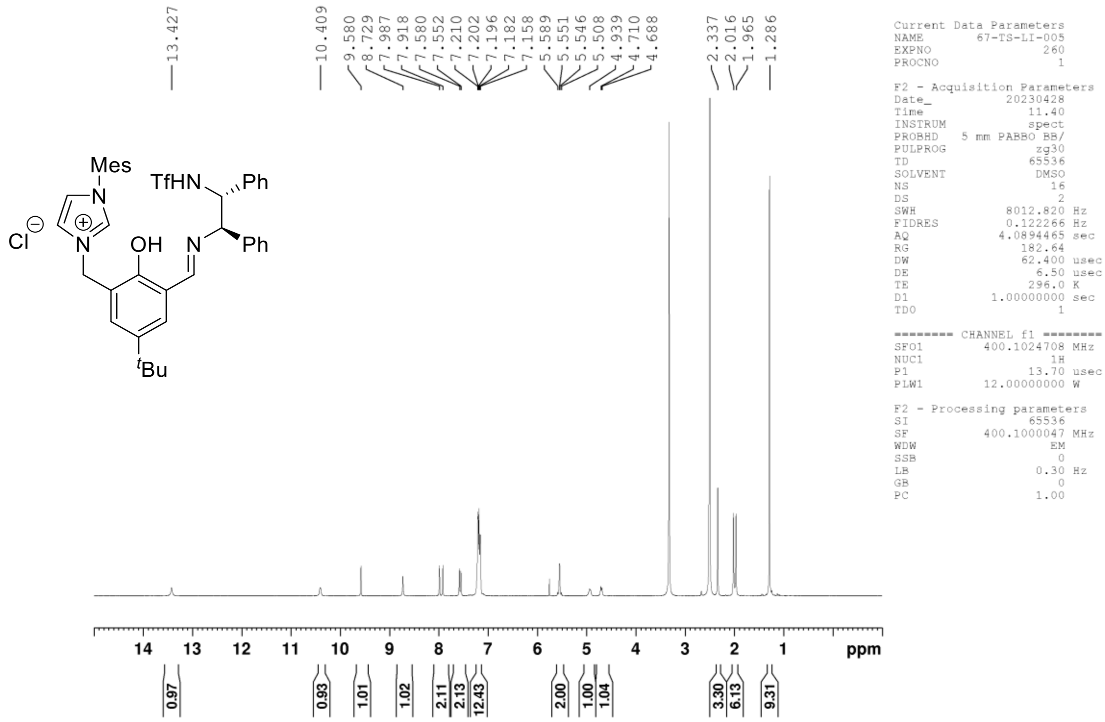
13.427
10.409
9.580
8.729
7.987
7.918
7.580
7.552
7.210
7.202
7.196
7.182
7.158
5.589
5.551
5.546
5.508
4.939
4.710
4.688
2.337
2.016
1.965
1.286
Mes
TfHN
Ph
Cl
OH
N
Ph
tBu
14
13
12
11
10
9
8
7
6
5
4
3
2
1
ppm
0.97
0.93
1.01
1.02
2.11
2.13
12.43
2.00
1.00
1.04
3.30
6.13
9.31
Current Data Parameters
NAME 67-TS-LI-005
EXPNO 260
PROCNO 1
F2 - Acquisition Parameters
Date_ 20230428
Time 11.40
INSTRUM spect
PROBHD 5 mm PABBO BB/
PULPROG zg30
TD 65536
SOLVENT DMSO
NS 16
DS 2
SWH 8012.820 Hz
FIDRES 0.122266 Hz
AQ 4.0894465 sec
RG 182.64
DW 62.400 usec
DE 6.50 usec
TE 296.0 K
D1 1.00000000 sec
TD0 1
======== CHANNEL f1 ========
SFO1 400.1024708 MHz
NUC1 1H
P1 13.70 usec
PLW1 12.00000000 W
F2 - Processing parameters
SI 65536
SF 400.1000047 MHz
WDW EM
SSB 0
LB 0.30 Hz
GB 0
PC 1.00

^{13}C

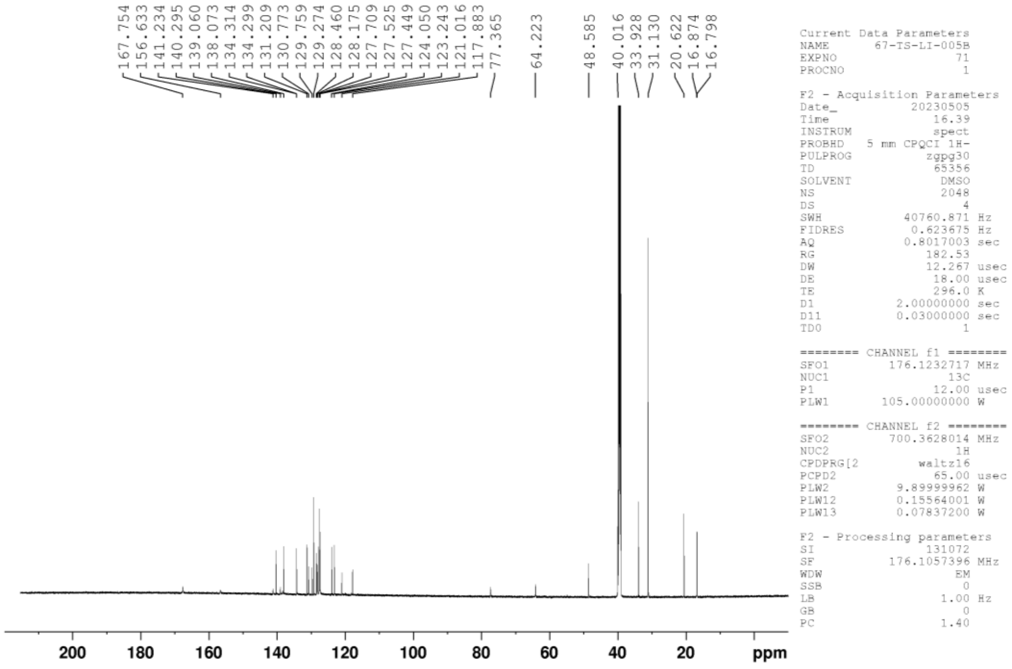
167.754
156.633
141.234
140.295
139.060
138.073
134.314
134.299
131.209
130.773
129.759
129.274
128.460
128.175
127.709
127.525
127.449
124.050
123.243
121.016
117.883
77.365
64.223
48.585
40.016
33.928
31.130
20.622
16.874
16.798
Current Data Parameters
NAME 67-TS-LI-005B
EXPNO 71
PROCNO 1
F2 - Acquisition Parameters
Date_ 20230505
Time 16.39
INSTRUM spect
PROBHD 5 mm CPQCI 1H-
PULPROG zgpg30
TD 65356
SOLVENT DMSO
NS 2048
DS 4
SWH 40760.871 Hz
FIDRES 0.623675 Hz
AQ 0.8017003 sec
RG 182.53
DW 12.267 usec
DE 18.00 usec
TE 296.0 K
D1 2.00000000 sec
D11 0.03000000 sec
TD0 1
======== CHANNEL f1 ========
SFO1 176.1232717 MHz
NUC1 13C
P1 12.00 usec
PLW1 105.00000000 W
======== CHANNEL f2 ========
SFO2 700.3628014 MHz
NUC2 1H
CPDPRG[2 waltz16
PCPD2 65.00 usec
PLW2 9.89999962 W
PLW12 0.15564001 W
PLW13 0.07837200 W
F2 - Processing parameters
SI 131072
SF 176.1057396 MHz
WDW EM
SSB 0
LB 1.00 Hz
GB 0
PC 1.40
200 180 160 140 120 100 80 60 40 20 ppm

^{19}F

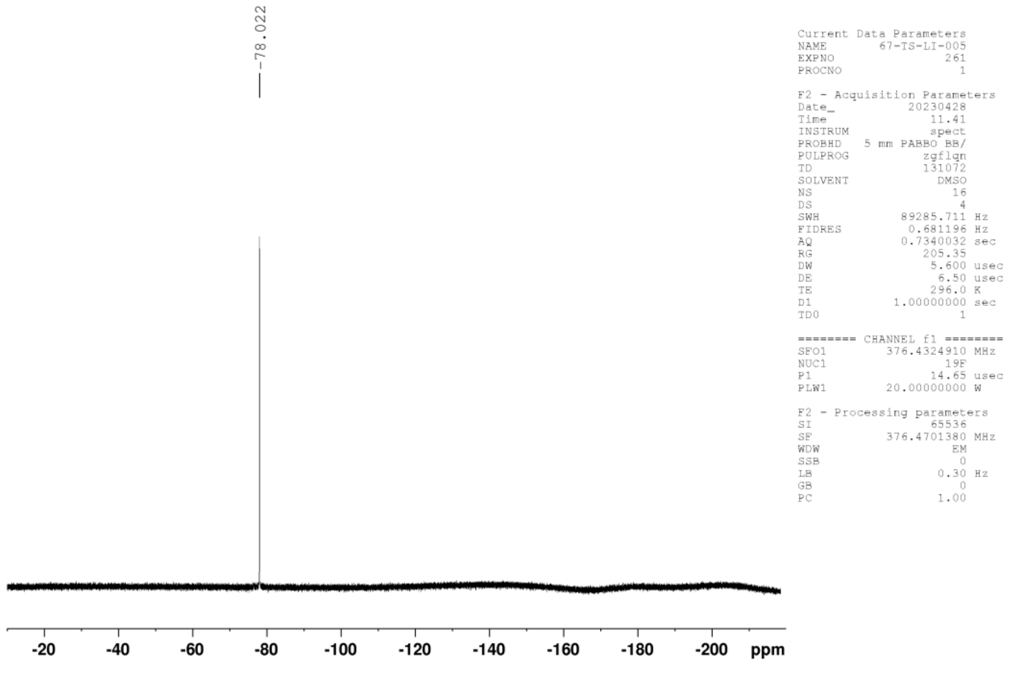
-78.022
Current Data Parameters
NAME 67-TS-LI-005
EXPNO 261
PROCNO 1
F2 - Acquisition Parameters
Date_ 20230428
Time 11.41
INSTRUM spect
PROBHD 5 mm PABBO BB/
PULPROG zgflqn
TD 131072
SOLVENT DMSO
NS 16
DS 4
SWH 89285.711 Hz
FIDRES 0.681196 Hz
AQ 0.7340032 sec
RG 205.35
DW 5.600 usec
DE 6.50 usec
TE 296.0 K
D1 1.00000000 sec
TD0 1
======== CHANNEL f1 ========
SFO1 376.4324910 MHz
NUC1 19F
P1 14.65 usec
PLW1 20.00000000 W
F2 - Processing parameters
SI 65536
SF 376.4701380 MHz
WDW EM
SSB 0
LB 0.30 Hz
GB 0
PC 1.00
-20 -40 -60 -80 -100 -120 -140 -160 -180 -200 ppm

S31

^{1}H

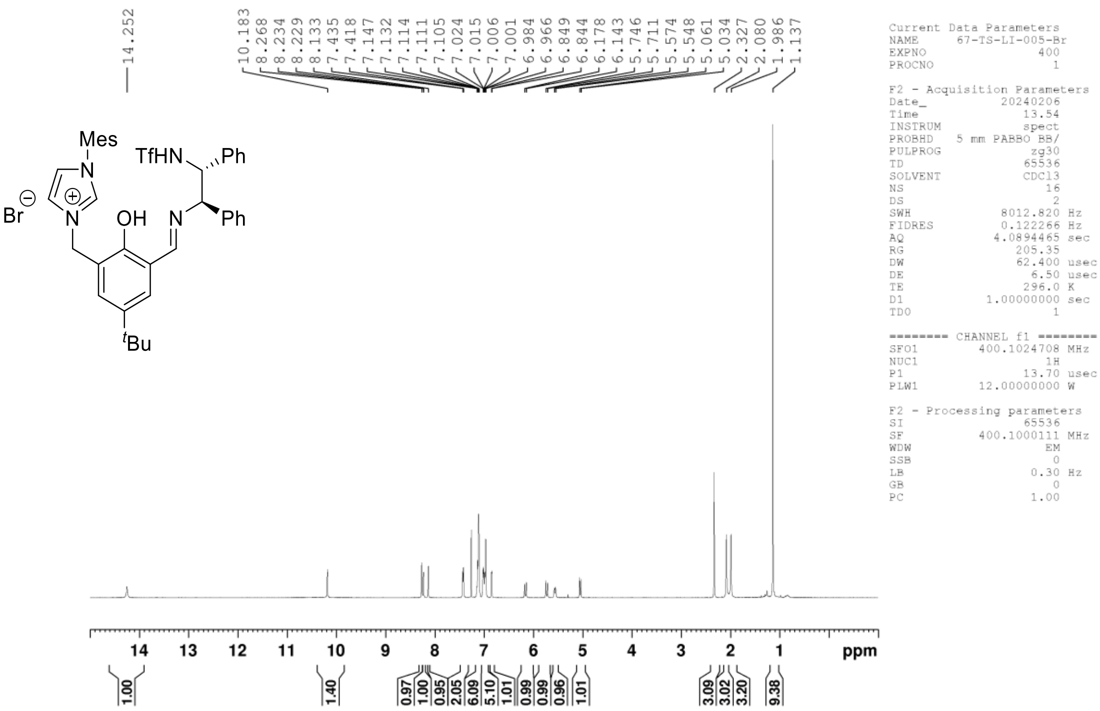

Mes
TfHN
Ph
Br
OH
N
Ph
tBu
14.252
10.183
8.268
8.234
8.229
8.133
7.435
7.418
7.147
7.132
7.114
7.111
7.105
7.024
7.015
7.006
7.001
6.984
6.966
6.849
6.844
6.178
6.143
5.746
5.711
5.574
5.548
5.061
5.034
2.327
2.080
1.986
1.137
14 13 12 11 10 9 8 7 6 5 4 3 2 1 ppm
1.00
1.40
0.97
1.00
0.95
2.05
6.09
5.10
1.01
0.99
0.99
0.96
1.01
3.09
3.02
3.20
9.38
Current Data Parameters
NAME 67-TS-LI-005-Br
EXPNO 400
PROCNO 1
F2 - Acquisition Parameters
Date_ 20240206
Time 13.54
INSTRUM spect
PROBHD 5 mm PABBO BB/
PULPROG zg30
TD 65536
SOLVENT CDCl3
NS 16
DS 2
SWH 8012.820 Hz
FIDRES 0.122266 Hz
AQ 4.0894465 sec
RG 205.35
DW 62.400 usec
DE 6.50 usec
TE 296.0 K
D1 1.00000000 sec
TD0 1
======== CHANNEL f1 ========
SFO1 400.1024708 MHz
NUC1 1H
P1 13.70 usec
PLW1 12.00000000 W
F2 - Processing parameters
SI 65536
SF 400.1000111 MHz
WDW EM
SSB 0
LB 0.30 Hz
GB 0
PC 1.00

^{13}C

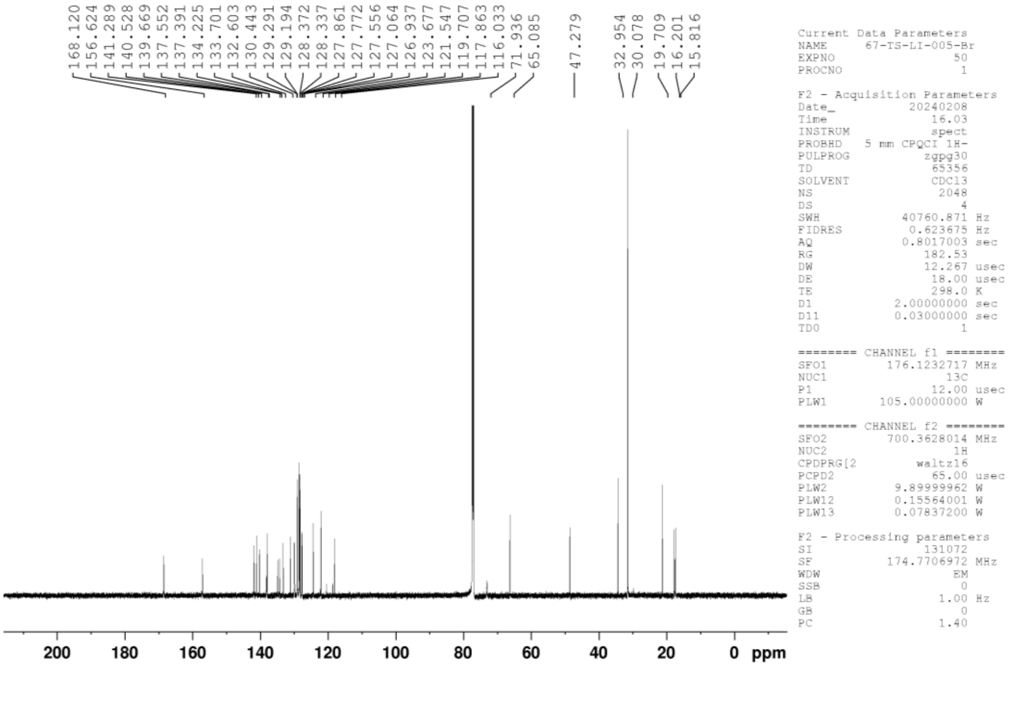

168.120
156.624
141.289
140.528
139.669
137.552
137.391
134.225
133.701
132.603
130.443
129.291
129.194
128.372
128.337
127.861
127.772
127.556
127.064
126.937
123.677
121.547
119.707
117.863
116.033
71.936
65.085
47.279
32.954
30.078
19.709
16.201
15.816
200 180 160 140 120 100 80 60 40 20 0 ppm
Current Data Parameters
NAME 67-TS-LI-005-Br
EXPNO 50
PROCNO 1
F2 - Acquisition Parameters
Date_ 20240208
Time 16.03
INSTRUM spect
PROBHD 5 mm CPQCI 1H-
PULPROG zgpg30
TD 65356
SOLVENT CDCl3
NS 2048
DS 4
SWH 40760.871 Hz
FIDRES 0.623675 Hz
AQ 0.8017003 sec
RG 182.53
DW 12.267 usec
DE 18.00 usec
TE 298.0 K
D1 2.00000000 sec
D11 0.03000000 sec
TD0 1
======== CHANNEL f1 ========
SFO1 176.1232717 MHz
NUC1 13C
P1 12.00 usec
PLW1 105.00000000 W
======== CHANNEL f2 ========
SFO2 700.3628014 MHz
NUC2 1H
CPDPRG[2 waltz16
PCPD2 65.00 usec
PLW2 9.89999962 W
PLW12 0.15564001 W
PLW13 0.07837200 W
F2 - Processing parameters
SI 131072
SF 174.7706972 MHz
WDW EM
SSB 0
LB 1.00 Hz
GB 0
PC 1.40

^{19}F

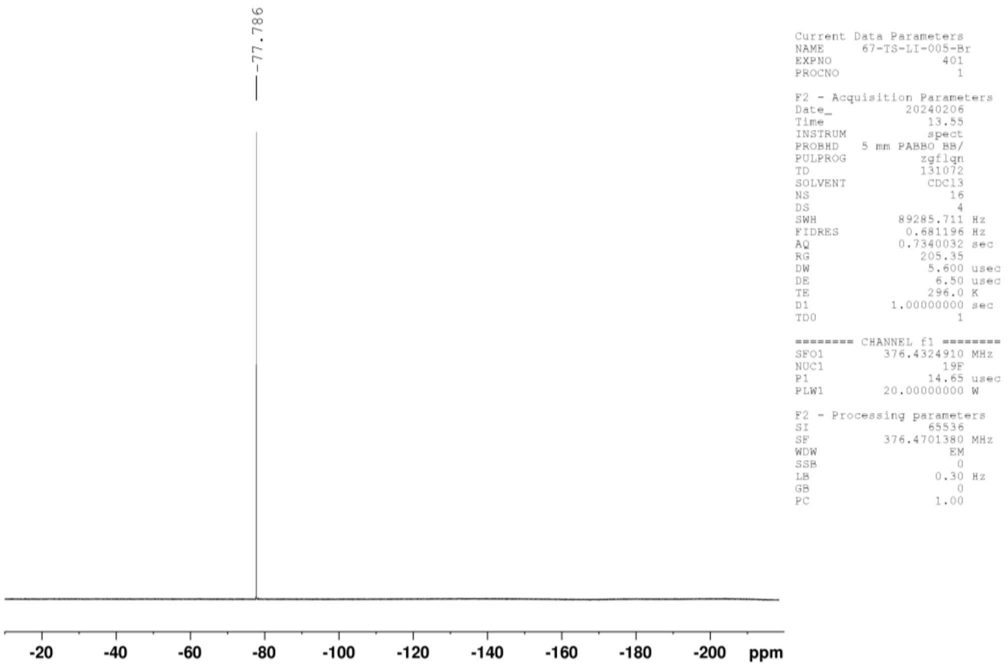

-77.786
Current Data Parameters
NAME 67-TS-LI-005-Br
EXPNO 401
PROCNO 1
F2 - Acquisition Parameters
Date_ 20240206
Time 13.55
INSTRUM spect
PROBHD 5 mm PABBO BB/
PULPROG zgflqn
TD 131072
SOLVENT CDCl3
NS 16
DS 4
SWH 89285.711 Hz
FIDRES 0.681196 Hz
AQ 0.7340032 sec
RG 205.35
DW 5.600 usec
DE 6.50 usec
TE 296.0 K
D1 1.00000000 sec
TD0 1
======== CHANNEL f1 ========
SFO1 376.4324910 MHz
NUC1 19F
P1 14.65 usec
PLW1 20.00000000 W
F2 - Processing parameters
SI 65536
SF 376.4701380 MHz
WDW EM
SSB 0
LB 0.30 Hz
GB 0
PC 1.00
-20 -40 -60 -80 -100 -120 -140 -160 -180 -200 ppm

S32

^{1}H

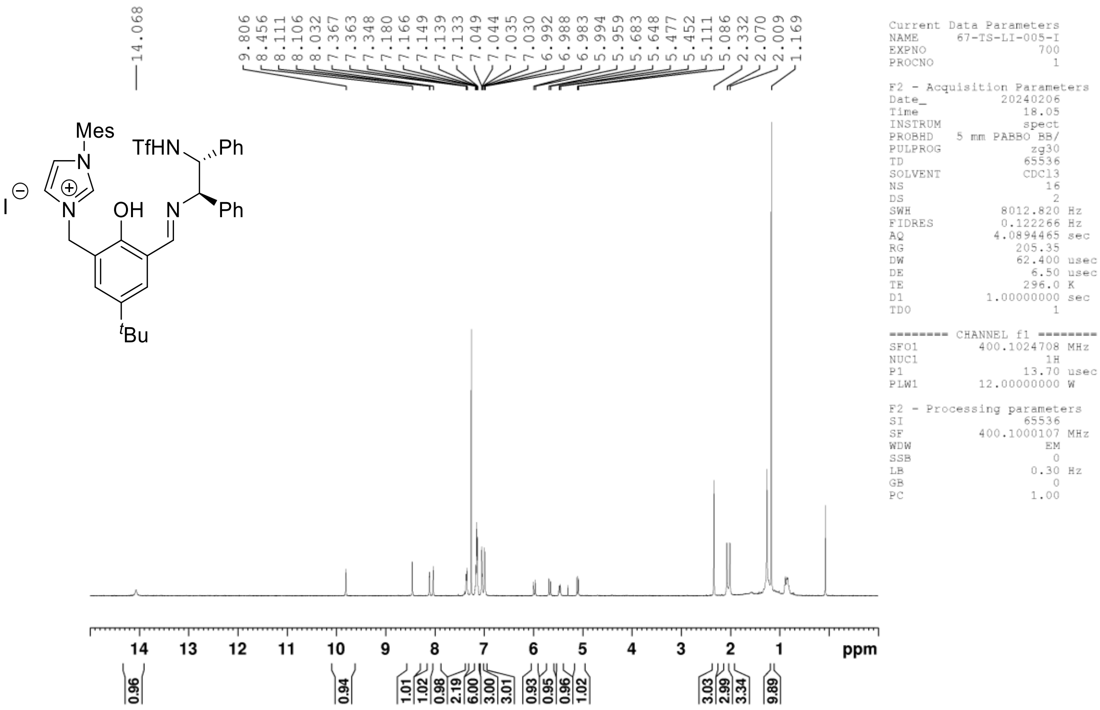

14.068
9.806 8.456 8.111 8.106 8.032 7.367 7.363 7.348 7.180 7.166 7.149 7.139 7.133 7.049 7.044 7.035 7.030 6.992 6.988 6.983 5.994 5.959 5.683 5.648 5.477 5.452 5.111 5.086 2.332 2.070 2.009 1.169
Mes
TfHN
Ph
OH
N
Ph
I
tBu
Current Data Parameters
NAME 67-TS-LI-005-I
EXPNO 700
PROCNO 1
F2 - Acquisition Parameters
Date_ 20240206
Time 18.05
INSTRUM spect
PROBHD 5 mm PABBO BB/
PULPROG zg30
TD 65536
SOLVENT CDCl3
NS 16
DS 2
SWH 8012.820 Hz
FIDRES 0.122266 Hz
AQ 4.0894465 sec
RG 205.35
DW 62.400 usec
DE 6.50 usec
TE 296.0 K
D1 1.00000000 sec
TD0 1
======== CHANNEL f1 ========
SFO1 400.1024708 MHz
NUC1 1H
P1 13.70 usec
PLW1 12.00000000 W
F2 - Processing parameters
SI 65536
SF 400.1000107 MHz
WDW EM
SSB 0
LB 0.30 Hz
GB 0
PC 1.00
14 13 12 11 10 9 8 7 6 5 4 3 2 1 ppm
0.96 0.94 1.01 1.02 0.98 2.19 6.00 3.00 3.01 0.93 0.95 0.96 1.02 3.03 2.99 3.34 9.89

^{13}C

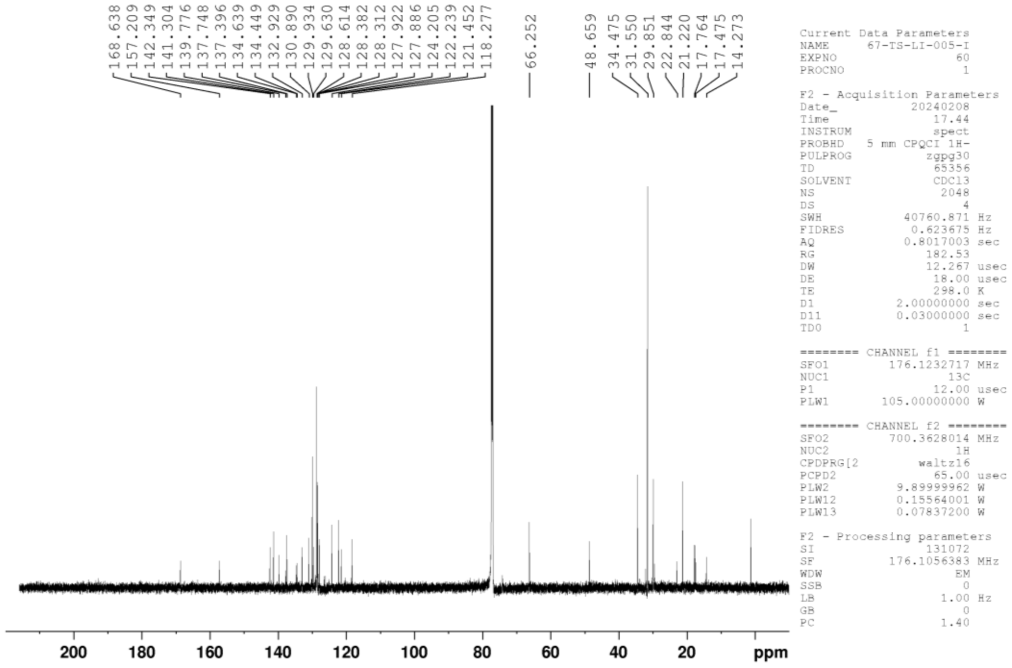
168.638
157.209
142.349
141.304
139.776
137.748
137.396
134.639
134.449
132.929
130.890
129.934
129.630
128.614
128.382
128.312
127.922
127.886
124.205
122.239
121.452
118.277
66.252
48.659
34.475
31.550
29.851
22.844
21.220
17.764
17.475
14.273
200 180 160 140 120 100 80 60 40 20 ppm
Current Data Parameters
NAME 67-TS-LI-005-I
EXPNO 60
PROCNO 1
F2 - Acquisition Parameters
Date_ 20240208
Time 17.44
INSTRUM spect
PROBHD 5 mm CPQCI 1H-
PULPROG zgpg30
TD 65356
SOLVENT CDCl3
NS 2048
DS 4
SWH 40760.871 Hz
FIDRES 0.623675 Hz
AQ 0.8017003 sec
RG 182.53
DW 12.267 usec
DE 18.00 usec
TE 298.0 K
D1 2.00000000 sec
D11 0.03000000 sec
TD0 1
======== CHANNEL f1 ========
SFO1 176.1232717 MHz
NUC1 13C
P1 12.00 usec
PLW1 105.00000000 W
======== CHANNEL f2 ========
SFO2 700.3628014 MHz
NUC2 1H
CPDPRG[2 waltz16
PCPD2 65.00 usec
PLW2 9.89999962 W
PLW12 0.15564001 W
PLW13 0.07837200 W
F2 - Processing parameters
SI 131072
SF 176.1056383 MHz
WDW EM
SSB 0
LB 1.00 Hz
GB 0
PC 1.40

^{19}F

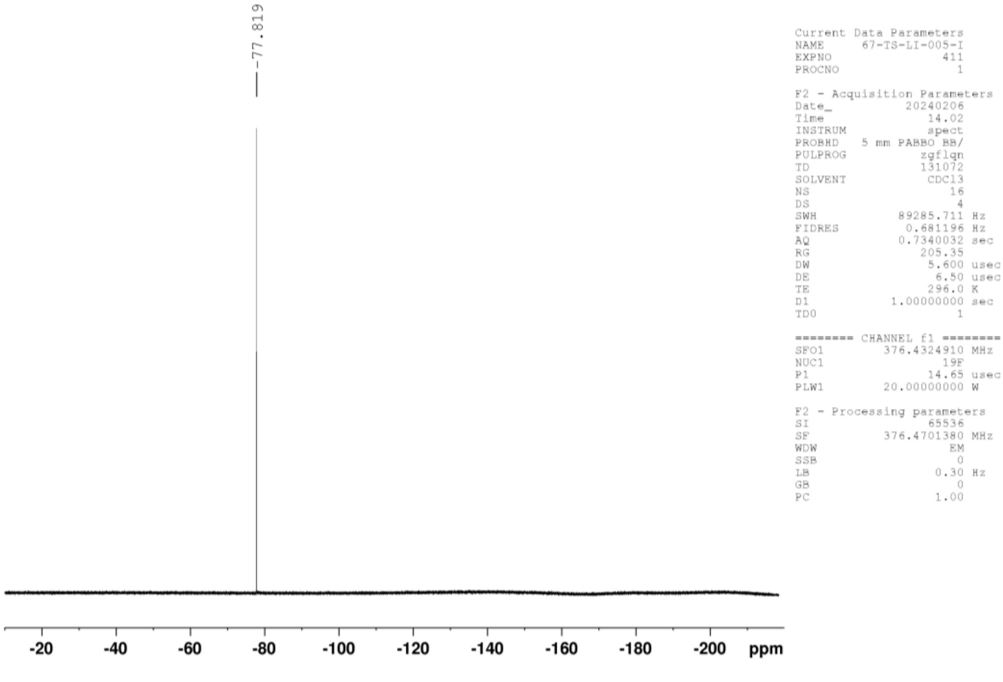
-77.819
-20 -40 -60 -80 -100 -120 -140 -160 -180 -200 ppm
Current Data Parameters
NAME 67-TS-LI-005-I
EXPNO 411
PROCNO 1
F2 - Acquisition Parameters
Date_ 20240206
Time 14.02
INSTRUM spect
PROBHD 5 mm PABBO BB/
PULPROG zgflqn
TD 131072
SOLVENT CDCl3
NS 16
DS 4
SWH 89285.711 Hz
FIDRES 0.681196 Hz
AQ 0.7340032 sec
RG 205.35
DW 5.600 usec
DE 6.50 usec
TE 296.0 K
D1 1.00000000 sec
TD0 1
======== CHANNEL f1 ========
SFO1 376.4324910 MHz
NUC1 19F
P1 14.65 usec
PLW1 20.00000000 W
F2 - Processing parameters
SI 65536
SF 376.4701380 MHz
WDW EM
SSB 0
LB 0.30 Hz
GB 0
PC 1.00

S35

^{1}H

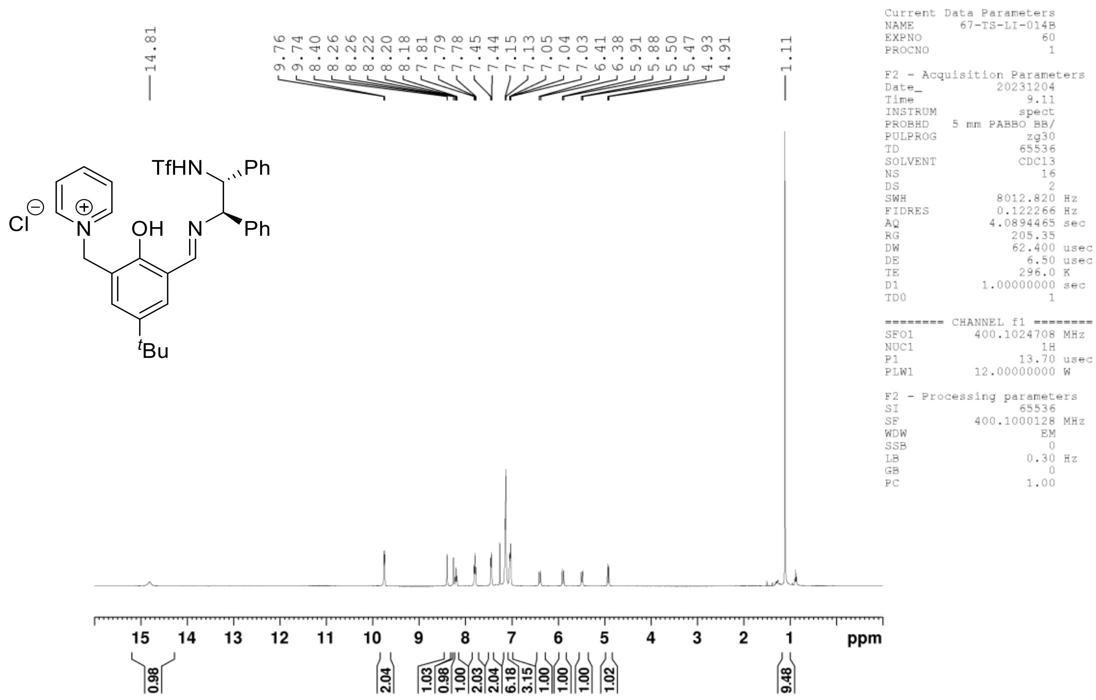
TfHN
Ph
Cl
N
OH
N
Ph
tBu
14.81
9.76 9.74 8.40 8.26 8.26 8.22 8.20 8.18 7.81 7.79 7.78 7.45 7.44 7.15 7.13 7.05 7.04 7.03 6.41 6.38 5.91 5.88 5.50 5.47 4.93 4.91
1.11
Current Data Parameters
NAME 67-TS-LI-014B
EXPNO 60
PROCNO 1
F2 - Acquisition Parameters
Date_ 20231204
Time 9.11
INSTRUM spect
PROBHD 5 mm PABBO BB/
PULPROG zg30
TD 65536
SOLVENT CDCl3
NS 16
DS 2
SWH 8012.820 Hz
FIDRES 0.122266 Hz
AQ 4.0894465 sec
RG 205.35
DW 62.400 usec
DE 6.50 usec
TE 296.0 K
D1 1.00000000 sec
TD0 1
======== CHANNEL f1 ========
SFO1 400.1024708 MHz
NUC1 1H
P1 13.70 usec
PLW1 12.00000000 W
F2 - Processing parameters
SI 65536
SF 400.1000128 MHz
WDW EM
SSB 0
LB 0.30 Hz
GB 0
PC 1.00
15 14 13 12 11 10 9 8 7 6 5 4 3 2 1 ppm
0.98 2.04 1.03 0.98 1.00 2.03 2.04 6.18 3.15 1.00 1.00 1.00 1.02 9.48

^{13}C

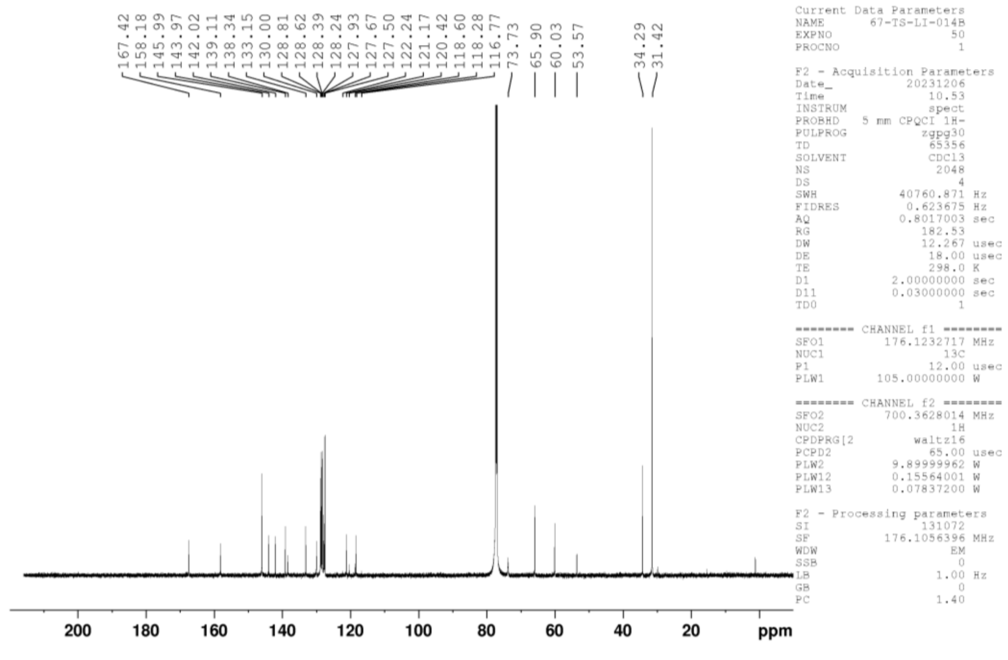
167.42 158.18 145.99 143.97 142.02 139.11 138.34 133.15 130.00 128.81 128.62 128.39 128.24 127.93 127.67 127.50 122.24 121.17 120.42 118.60 118.28 116.77
73.73 65.90 60.03 53.57
34.29 31.42
Current Data Parameters
NAME 67-TS-LI-014B
EXPNO 50
PROCNO 1
F2 - Acquisition Parameters
Date_ 20231206
Time 10.53
INSTRUM spect
PROBHD 5 mm CPQCI 1H-
PULPROG zgpg30
TD 65356
SOLVENT CDCl3
NS 2048
DS 4
SWH 40760.871 Hz
FIDRES 0.623675 Hz
AQ 0.8017003 sec
RG 182.53
DW 12.267 usec
DE 18.00 usec
TE 298.0 K
D1 2.00000000 sec
D11 0.03000000 sec
TD0 1
======== CHANNEL f1 ========
SFO1 176.1232717 MHz
NUC1 13C
P1 12.00 usec
PLW1 105.00000000 W
======== CHANNEL f2 ========
SFO2 700.3628014 MHz
NUC2 1H
CPDPRG[2 waltz16
PCPD2 65.00 usec
PLW2 9.89999962 W
PLW12 0.15564001 W
PLW13 0.07837200 W
F2 - Processing parameters
SI 131072
SF 176.1056396 MHz
WDW EM
SSB 0
LB 1.00 Hz
GB 0
PC 1.40
200 180 160 140 120 100 80 60 40 20 ppm

[19]F

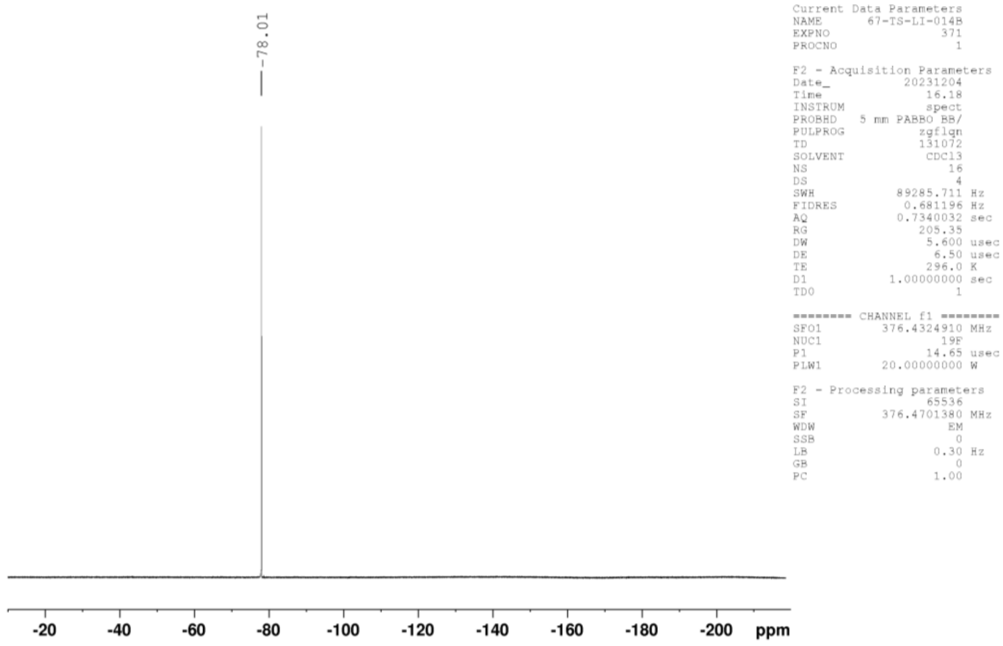
-78.01
Current Data Parameters
NAME 67-TS-LI-014B
EXPNO 371
PROCNO 1
F2 - Acquisition Parameters
Date_ 20231204
Time 16.18
INSTRUM spect
PROBHD 5 mm PABBO BB/
PULPROG zgflqn
TD 131072
SOLVENT CDCl3
NS 16
DS 4
SWH 89285.711 Hz
FIDRES 0.681196 Hz
AQ 0.7340032 sec
RG 205.35
DW 5.600 usec
DE 6.50 usec
TE 296.0 K
D1 1.00000000 sec
TD0 1
======== CHANNEL f1 ========
SFO1 376.4324910 MHz
NUC1 19F
P1 14.65 usec
PLW1 20.00000000 W
F2 - Processing parameters
SI 65536
SF 376.4701380 MHz
WDW EM
SSB 0
LB 0.30 Hz
GB 0
PC 1.00
-20 -40 -60 -80 -100 -120 -140 -160 -180 -200 ppm

S36

[1]H

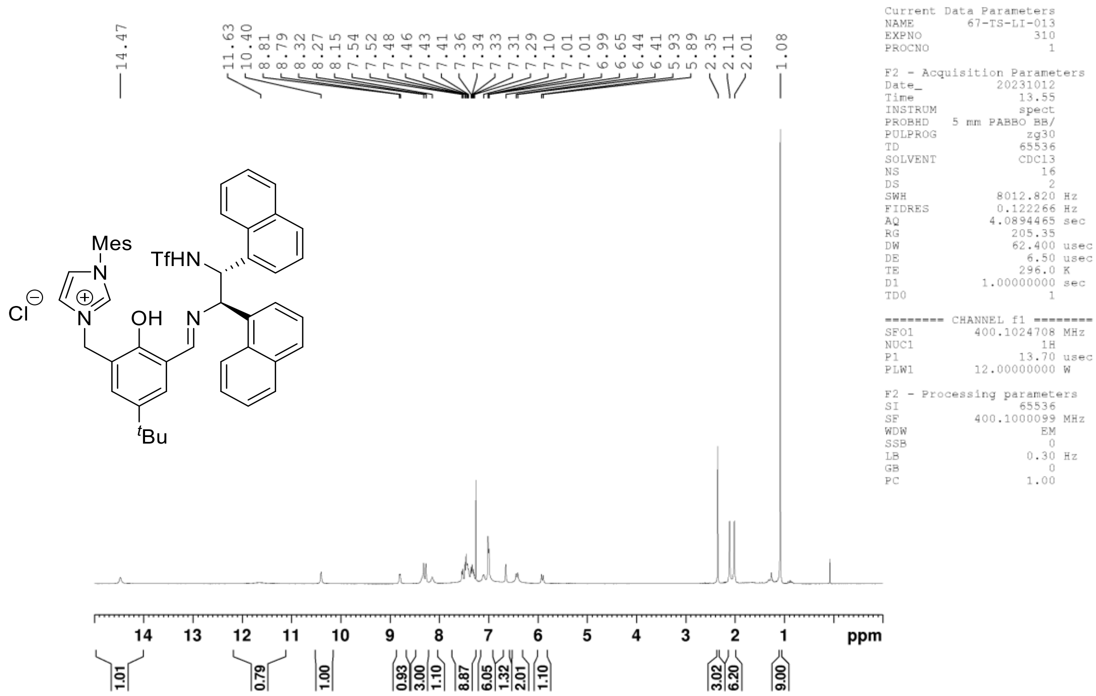
14.47
11.63
10.40
8.81
8.79
8.32
8.27
8.15
7.54
7.52
7.48
7.46
7.43
7.41
7.36
7.34
7.33
7.31
7.29
7.10
7.01
7.01
6.99
6.65
6.44
6.41
5.93
5.89
2.35
2.11
2.01
1.08
Mes
TfHN
Cl
OH
N
tBu
Current Data Parameters
NAME 67-TS-LI-013
EXPNO 310
PROCNO 1
F2 - Acquisition Parameters
Date_ 20231012
Time 13.55
INSTRUM spect
PROBHD 5 mm PABBO BB/
PULPROG zg30
TD 65536
SOLVENT CDCl3
NS 16
DS 2
SWH 8012.820 Hz
FIDRES 0.122266 Hz
AQ 4.0894465 sec
RG 205.35
DW 62.400 usec
DE 6.50 usec
TE 296.0 K
D1 1.00000000 sec
TD0 1
======== CHANNEL f1 ========
SFO1 400.1024708 MHz
NUC1 1H
P1 13.70 usec
PLW1 12.00000000 W
F2 - Processing parameters
SI 65536
SF 400.1000099 MHz
WDW EM
SSB 0
LB 0.30 Hz
GB 0
PC 1.00
14 13 12 11 10 9 8 7 6 5 4 3 2 1 ppm
1.01
0.79
1.00
0.93
3.00
1.10
8.87
6.05
1.32
2.01
1.10
3.02
6.20
9.00

^{13}C

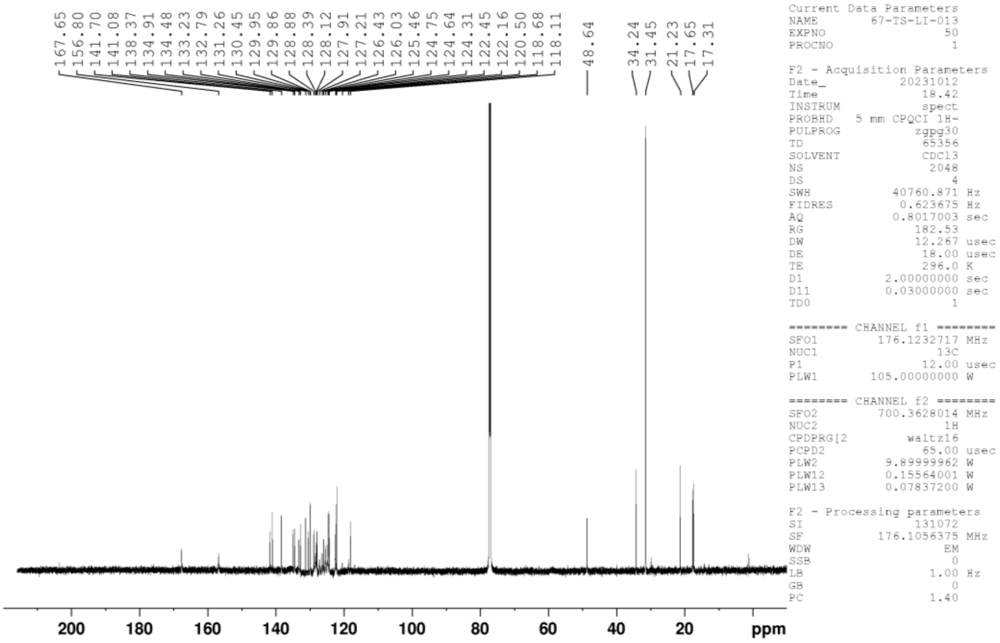
167.65
156.80
141.70
141.08
138.37
134.91
134.48
133.23
132.79
131.26
130.45
129.95
129.86
128.88
128.39
128.12
127.91
127.21
126.43
126.03
125.46
124.75
124.64
124.31
122.45
122.16
120.50
118.68
118.11
48.64
34.24
31.45
21.23
17.65
17.31
Current Data Parameters
NAME 67-TS-LI-013
EXPNO 50
PROCNO 1
F2 - Acquisition Parameters
Date_ 20231012
Time 18.42
INSTRUM spect
PROBHD 5 mm CPQCI 1H-
PULPROG zgpg30
TD 65356
SOLVENT CDCl3
NS 2048
DS 4
SWH 40760.871 Hz
FIDRES 0.623675 Hz
AQ 0.8017003 sec
RG 182.53
DW 12.267 usec
DE 18.00 usec
TE 296.0 K
D1 2.00000000 sec
D11 0.03000000 sec
TD0 1
======== CHANNEL f1 ========
SFO1 176.1232717 MHz
NUC1 13C
P1 12.00 usec
PLW1 105.00000000 W
======== CHANNEL f2 ========
SFO2 700.3628014 MHz
NUC2 1H
CPDPRG[2 waltz16
PCPD2 65.00 usec
PLW2 9.89999962 W
PLW12 0.15564001 W
PLW13 0.07837200 W
F2 - Processing parameters
SI 131072
SF 176.1056375 MHz
WDW EM
SSB 0
LB 1.00 Hz
GB 0
PC 1.40
200
180
160
140
120
100
80
60
40
20
ppm

^{19}F

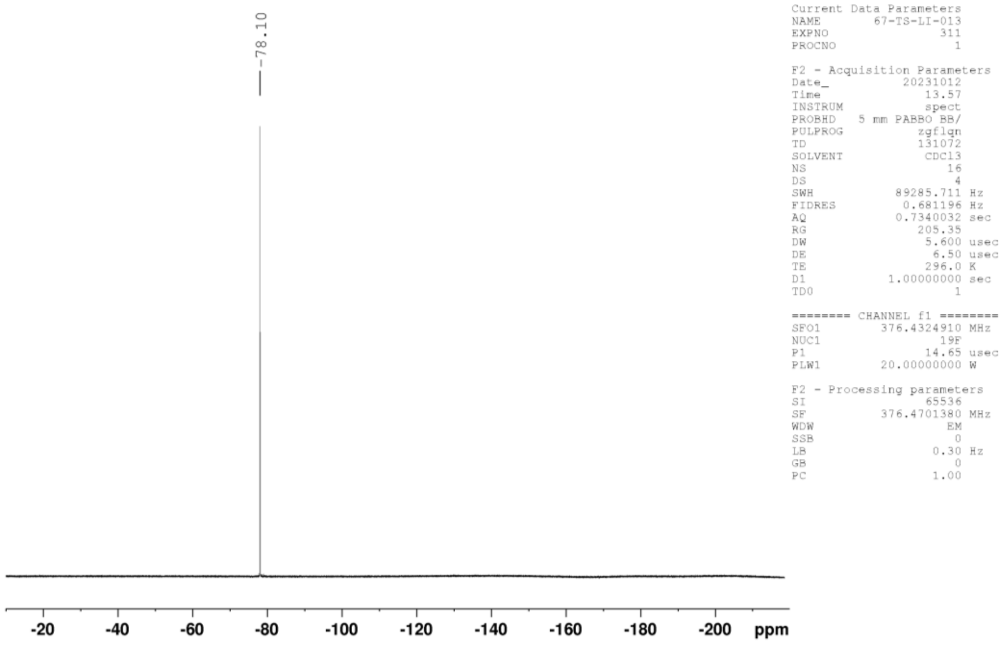
-78.10
Current Data Parameters
NAME 67-TS-LI-013
EXPNO 311
PROCNO 1
F2 - Acquisition Parameters
Date_ 20231012
Time 13.57
INSTRUM spect
PROBHD 5 mm PABBO BB/
PULPROG zgflqn
TD 131072
SOLVENT CDCl3
NS 16
DS 4
SWH 89285.711 Hz
FIDRES 0.681196 Hz
AQ 0.7340032 sec
RG 205.35
DW 5.600 usec
DE 6.50 usec
TE 296.0 K
D1 1.00000000 sec
TD0 1
======== CHANNEL f1 ========
SFO1 376.4324910 MHz
NUC1 19F
P1 14.65 usec
PLW1 20.00000000 W
F2 - Processing parameters
SI 65536
SF 376.4701380 MHz
WDW EM
SSB 0
LB 0.30 Hz
GB 0
PC 1.00
-20
-40
-60
-80
-100
-120
-140
-160
-180
-200
ppm

S37

^{1}H

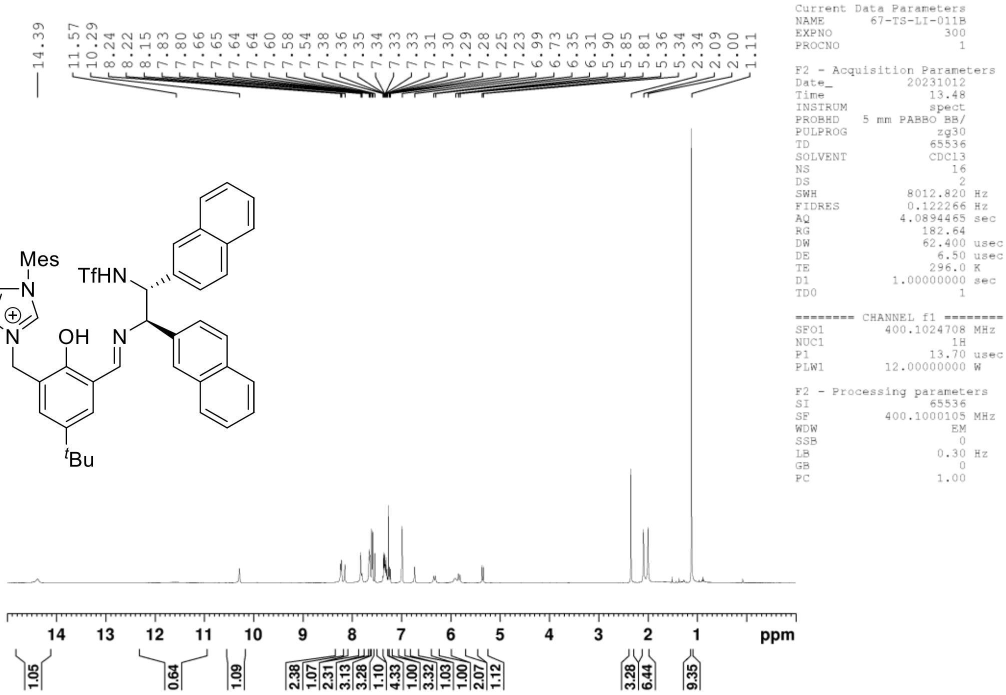
14.39
11.57 10.29 8.24 8.22 8.15 7.83 7.80 7.66 7.65 7.64 7.60 7.58 7.54 7.38 7.36 7.35 7.34 7.33 7.33 7.31 7.30 7.29 7.28 7.25 7.23 6.99 6.73 6.35 6.31 5.90 5.85 5.81 5.36 5.34 2.34 2.09 2.00 1.11
Mes
TfHN
Cl
OH
N
tBu
Current Data Parameters
NAME 67-TS-LI-011B
EXPNO 300
PROCNO 1
F2 - Acquisition Parameters
Date_ 20231012
Time 13.48
INSTRUM spect
PROBHD 5 mm PABBO BB/
PULPROG zg30
TD 65536
SOLVENT CDCl3
NS 16
DS 2
SWH 8012.820 Hz
FIDRES 0.122266 Hz
AQ 4.0894465 sec
RG 182.64
DW 62.400 usec
DE 6.50 usec
TE 296.0 K
D1 1.00000000 sec
TD0 1
======== CHANNEL f1 ========
SFO1 400.1024708 MHz
NUC1 1H
P1 13.70 usec
PLW1 12.00000000 W
F2 - Processing parameters
SI 65536
SF 400.1000105 MHz
WDW EM
SSB 0
LB 0.30 Hz
GB 0
PC 1.00
14 13 12 11 10 9 8 7 6 5 4 3 2 1 ppm
1.05 0.64 1.09 2.38 1.07 2.31 3.13 3.28 1.10 4.33 1.00 3.32 1.03 1.00 2.07 1.12 3.28 6.44 9.35

^{13}C

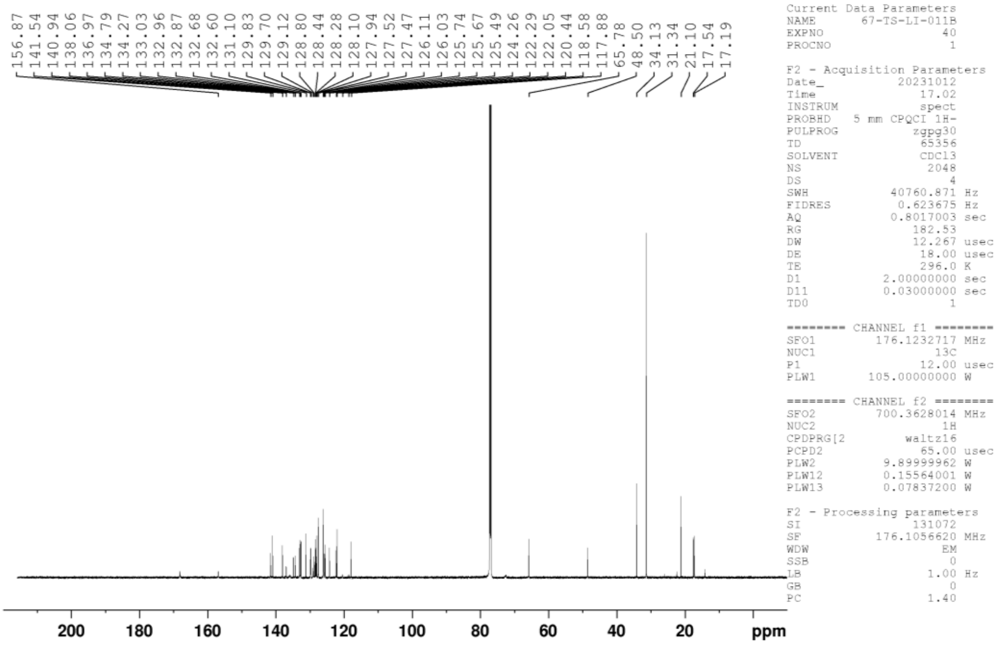
156.87 141.54 140.94 138.06 136.97 134.79 134.27 133.03 132.96 132.87 132.68 132.60 131.10 129.83 129.70 129.12 128.80 128.44 128.28 128.10 127.94 127.52 127.47 126.11 126.03 125.74 125.67 125.49 124.26 122.29 122.05 120.44 118.58 117.88 65.78 48.50 34.13 31.34 21.10 17.54 17.19
Current Data Parameters
NAME 67-TS-LI-011B
EXPNO 40
PROCNO 1
F2 - Acquisition Parameters
Date_ 20231012
Time 17.02
INSTRUM spect
PROBHD 5 mm CPQCI 1H-
PULPROG zgpg30
TD 65356
SOLVENT CDCl3
NS 2048
DS 4
SWH 40760.871 Hz
FIDRES 0.623675 Hz
AQ 0.8017003 sec
RG 182.53
DW 12.267 usec
DE 18.00 usec
TE 296.0 K
D1 2.00000000 sec
D11 0.03000000 sec
TD0 1
======== CHANNEL f1 ========
SFO1 176.1232717 MHz
NUC1 13C
P1 12.00 usec
PLW1 105.00000000 W
======== CHANNEL f2 ========
SFO2 700.3628014 MHz
NUC2 1H
CPDPRG[2 waltz16
PCPD2 65.00 usec
PLW2 9.89999962 W
PLW12 0.15564001 W
PLW13 0.07837200 W
F2 - Processing parameters
SI 131072
SF 176.1056620 MHz
WDW EM
SSB 0
LB 1.00 Hz
GB 0
PC 1.40
200 180 160 140 120 100 80 60 40 20 ppm

^{19}F

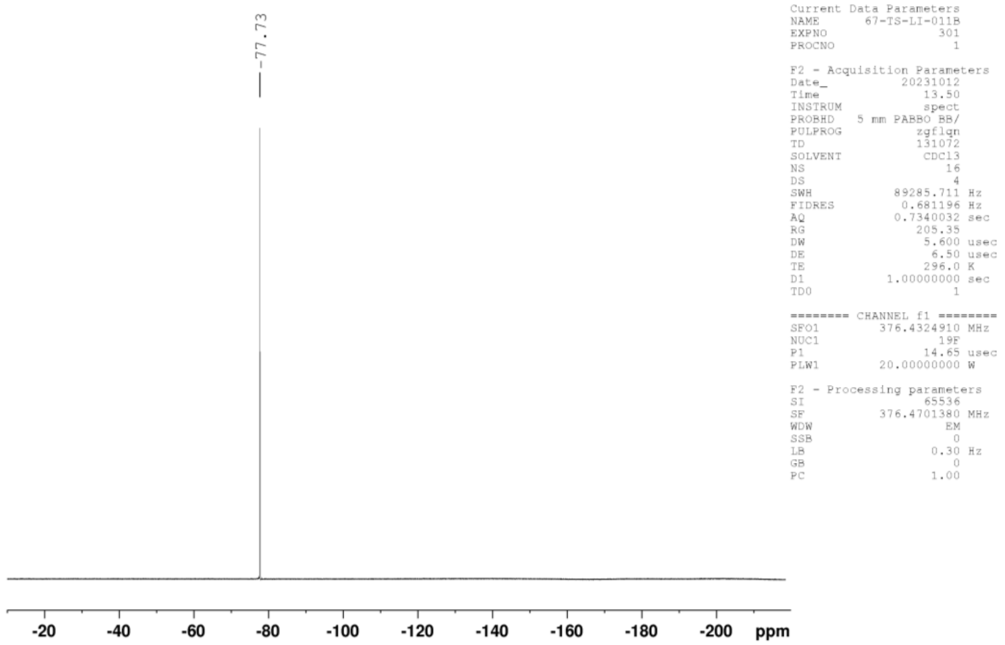
-77.73
Current Data Parameters
NAME 67-TS-LI-011B
EXPNO 301
PROCNO 1
F2 - Acquisition Parameters
Date_ 20231012
Time 13.50
INSTRUM spect
PROBHD 5 mm PABBO BB/
PULPROG zgflqn
TD 131072
SOLVENT CDCl3
NS 16
DS 4
SWH 89285.711 Hz
FIDRES 0.681196 Hz
AQ 0.7340032 sec
RG 205.35
DW 5.600 usec
DE 6.50 usec
TE 296.0 K
D1 1.00000000 sec
TD0 1
======== CHANNEL f1 ========
SFO1 376.4324910 MHz
NUC1 19F
P1 14.65 usec
PLW1 20.00000000 W
F2 - Processing parameters
SI 65536
SF 376.4701380 MHz
WDW EM
SSB 0
LB 0.30 Hz
GB 0
PC 1.00
-20 -40 -60 -80 -100 -120 -140 -160 -180 -200 ppm

S38

^{1}H

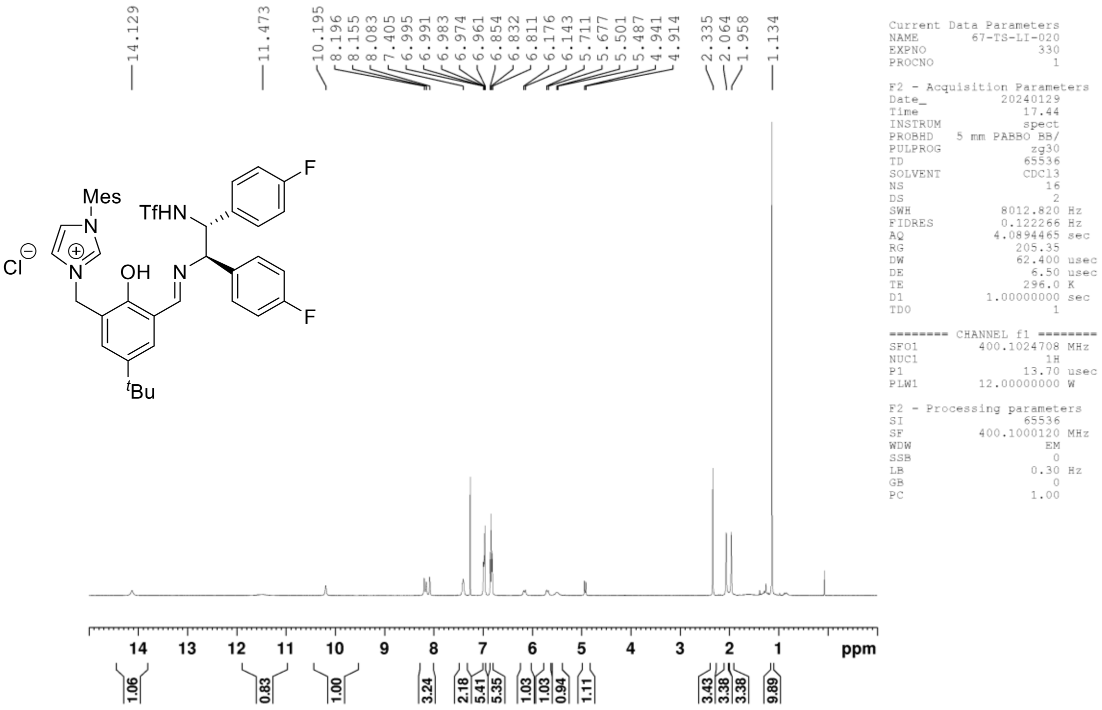
Mes
N
N
Cl
OH
N
TfHN
F
F
tBu
14.129
11.473
10.195
8.196
8.155
8.083
7.405
6.995
6.991
6.983
6.974
6.961
6.854
6.832
6.811
6.176
6.143
5.711
5.677
5.501
5.487
4.941
4.914
2.335
2.064
1.958
1.134
Current Data Parameters
NAME 67-TS-LI-020
EXPNO 330
PROCNO 1
F2 - Acquisition Parameters
Date_ 20240129
Time 17.44
INSTRUM spect
PROBHD 5 mm PABBO BB/
PULPROG zg30
TD 65536
SOLVENT CDCl3
NS 16
DS 2
SWH 8012.820 Hz
FIDRES 0.122266 Hz
AQ 4.0894465 sec
RG 205.35
DW 62.400 usec
DE 6.50 usec
TE 296.0 K
D1 1.00000000 sec
TD0 1
======== CHANNEL f1 ========
SFO1 400.1024708 MHz
NUC1 1H
P1 13.70 usec
PLW1 12.00000000 W
F2 - Processing parameters
SI 65536
SF 400.1000120 MHz
WDW EM
SSB 0
LB 0.30 Hz
GB 0
PC 1.00
14 13 12 11 10 9 8 7 6 5 4 3 2 1 ppm
1.06
0.83
1.00
3.24
2.18
5.41
5.35
1.03
1.03
0.94
1.11
3.43
3.38
3.38
9.89

^{13}C

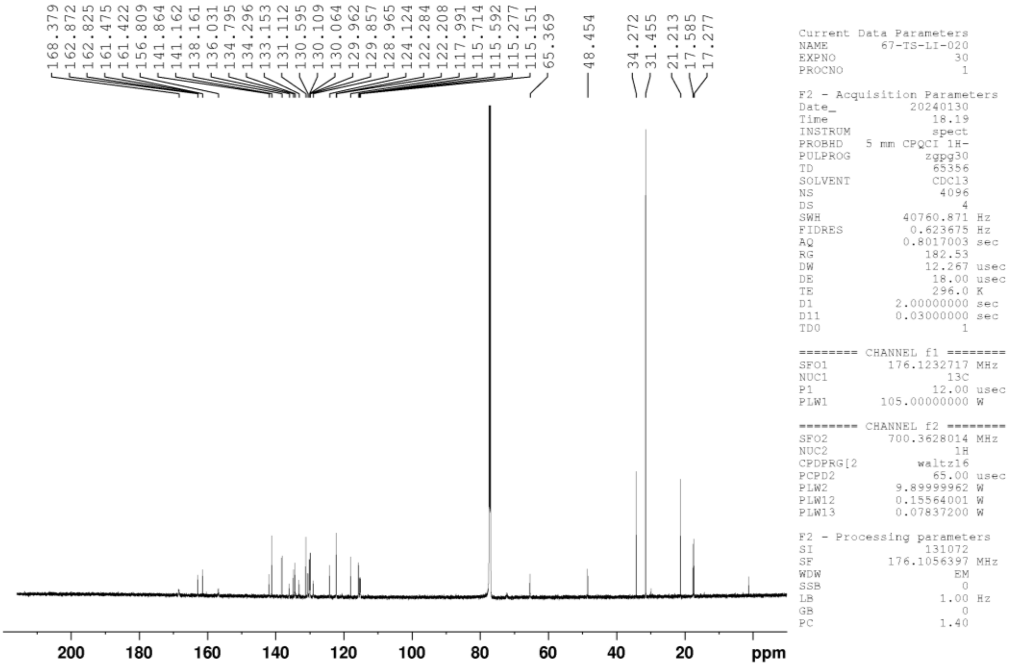
168.379
162.872
162.825
161.475
161.422
156.809
141.864
141.162
138.161
136.031
134.795
134.296
133.153
131.112
130.595
130.109
130.064
129.962
129.857
128.965
124.124
122.284
122.208
117.991
115.714
115.592
115.277
115.151
65.369
48.454
34.272
31.455
21.213
17.585
17.277
Current Data Parameters
NAME 67-TS-LI-020
EXPNO 30
PROCNO 1
F2 - Acquisition Parameters
Date_ 20240130
Time 18.19
INSTRUM spect
PROBHD 5 mm CPQCI 1H-
PULPROG zgpg30
TD 65356
SOLVENT CDCl3
NS 4096
DS 4
SWH 40760.871 Hz
FIDRES 0.623675 Hz
AQ 0.8017003 sec
RG 182.53
DW 12.267 usec
DE 18.00 usec
TE 296.0 K
D1 2.00000000 sec
D11 0.03000000 sec
TD0 1
======== CHANNEL f1 ========
SFO1 176.1232717 MHz
NUC1 13C
P1 12.00 usec
PLW1 105.00000000 W
======== CHANNEL f2 ========
SFO2 700.3628014 MHz
NUC2 1H
CPDPRG[2 waltz16
PCPD2 65.00 usec
PLW2 9.89999962 W
PLW12 0.15564001 W
PLW13 0.07837200 W
F2 - Processing parameters
SI 131072
SF 176.1056397 MHz
WDW EM
SSB 0
LB 1.00 Hz
GB 0
PC 1.40
200 180 160 140 120 100 80 60 40 20 ppm

^{19}F

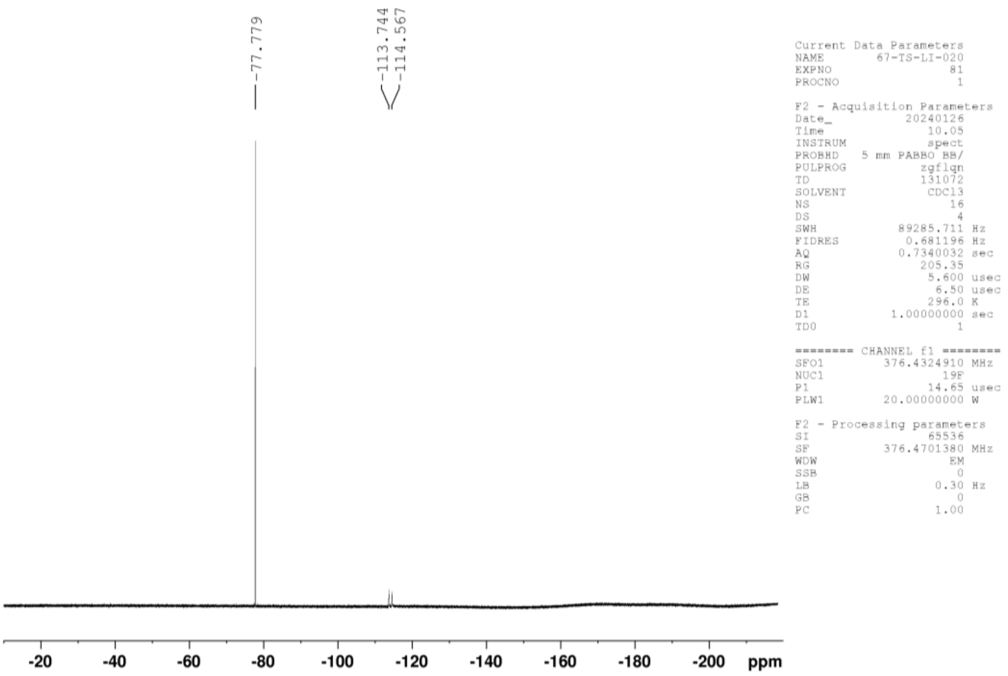
-77.779
-113.744
-114.567
Current Data Parameters
NAME 67-TS-LI-020
EXPNO 81
PROCNO 1
F2 - Acquisition Parameters
Date_ 20240126
Time 10.05
INSTRUM spect
PROBHD 5 mm PABBO BB/
PULPROG zgflqn
TD 131072
SOLVENT CDCl3
NS 16
DS 4
SWH 89285.711 Hz
FIDRES 0.681196 Hz
AQ 0.7340032 sec
RG 205.35
DW 5.600 usec
DE 6.50 usec
TE 296.0 K
D1 1.00000000 sec
TD0 1
======== CHANNEL f1 ========
SFO1 376.4324910 MHz
NUC1 19F
P1 14.65 usec
PLW1 20.00000000 W
F2 - Processing parameters
SI 65536
SF 376.4701380 MHz
WDW EM
SSB 0
LB 0.30 Hz
GB 0
PC 1.00
-20 -40 -60 -80 -100 -120 -140 -160 -180 -200 ppm

S39

^1H

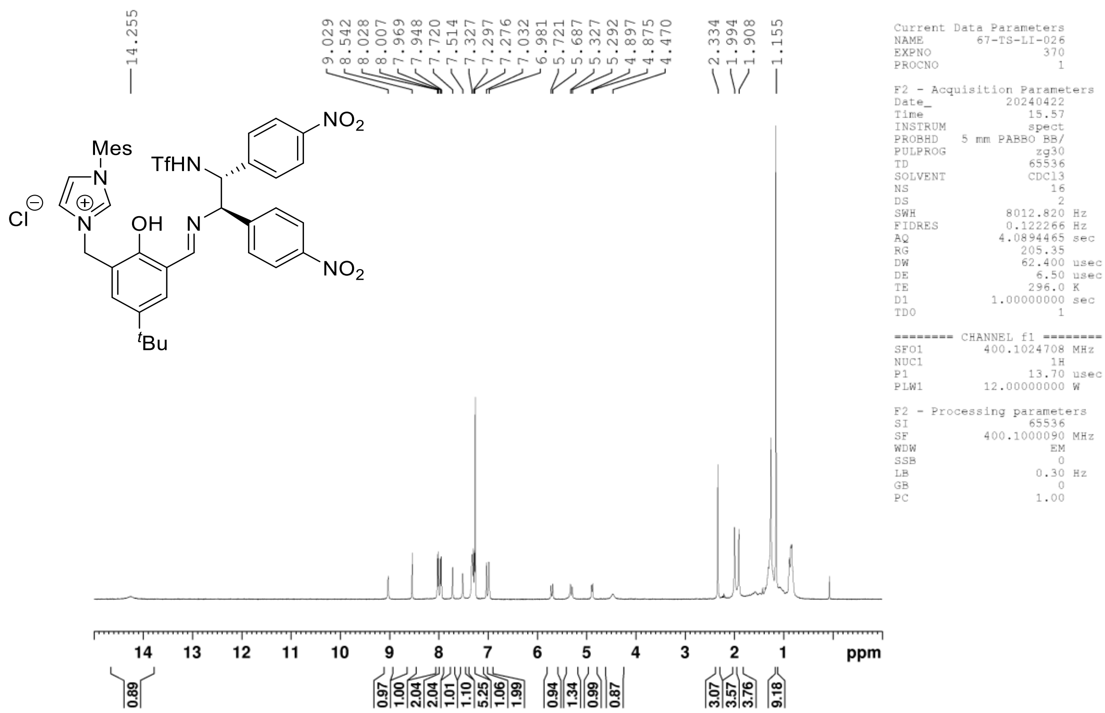

14.255
9.029 8.542 8.028 8.007 7.969 7.948 7.720 7.514 7.327 7.297 7.276 7.032 6.981 5.721 5.687 5.327 5.292 4.897 4.875 4.470
2.334 1.994 1.908 1.155
Mes
TfHN
NO2
Cl
OH
N
NO2
tBu
14 13 12 11 10 9 8 7 6 5 4 3 2 1 ppm
0.89
0.97 1.00 2.04 2.04 1.01 1.10 5.25 1.06 1.99
0.94 1.34 0.99 0.87
3.07 3.57 3.76 9.18
Current Data Parameters
NAME 67-TS-LI-026
EXPNO 370
PROCNO 1
F2 - Acquisition Parameters
Date_ 20240422
Time 15.57
INSTRUM spect
PROBHD 5 mm PABBO BB/
PULPROG zg30
TD 65536
SOLVENT CDCl3
NS 16
DS 2
SWH 8012.820 Hz
FIDRES 0.122266 Hz
AQ 4.0894465 sec
RG 205.35
DW 62.400 usec
DE 6.50 usec
TE 296.0 K
D1 1.00000000 sec
TD0 1
======== CHANNEL f1 ========
SFO1 400.1024708 MHz
NUC1 1H
P1 13.70 usec
PLW1 12.00000000 W
F2 - Processing parameters
SI 65536
SF 400.1000090 MHz
WDW EM
SSB 0
LB 0.30 Hz
GB 0
PC 1.00

^{13}C

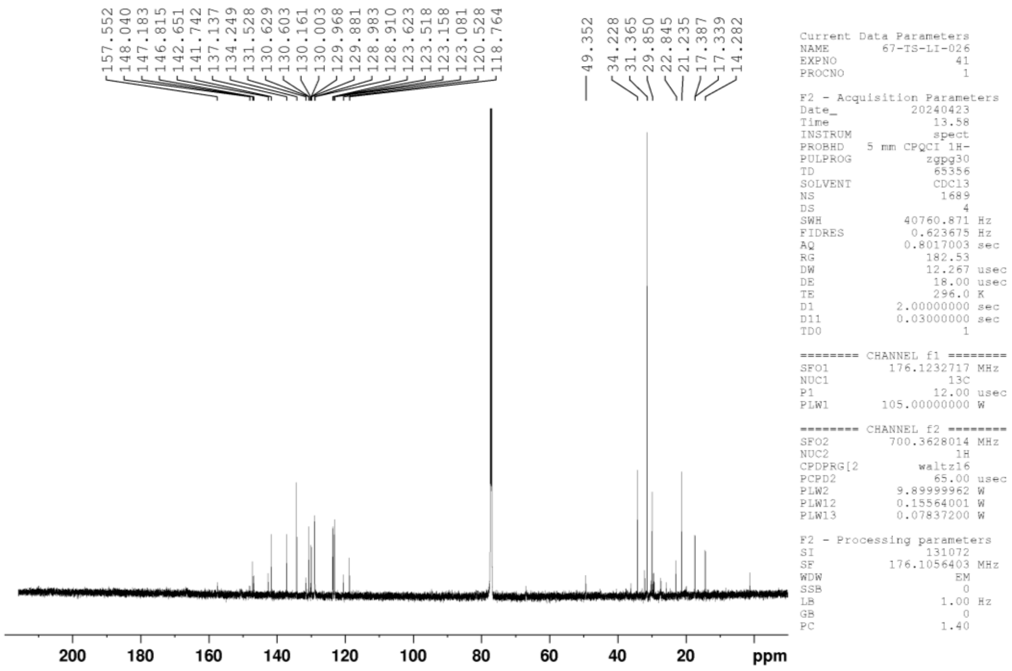

157.552 148.040 147.183 146.815 142.651 141.742 137.137 134.249 131.528 130.629 130.603 130.161 130.003 129.968 129.881 128.983 128.910 123.623 123.518 123.158 123.081 120.528 118.764
49.352 34.228 31.365 29.850 22.845 21.235 17.387 17.339 14.282
200 180 160 140 120 100 80 60 40 20 ppm
Current Data Parameters
NAME 67-TS-LI-026
EXPNO 41
PROCNO 1
F2 - Acquisition Parameters
Date_ 20240423
Time 13.58
INSTRUM spect
PROBHD 5 mm CPQCI 1H-
PULPROG zgpg30
TD 65356
SOLVENT CDCl3
NS 1689
DS 4
SWH 40760.871 Hz
FIDRES 0.623675 Hz
AQ 0.8017003 sec
RG 182.53
DW 12.267 usec
DE 18.00 usec
TE 296.0 K
D1 2.00000000 sec
D11 0.03000000 sec
TD0 1
======== CHANNEL f1 ========
SFO1 176.1232717 MHz
NUC1 13C
P1 12.00 usec
PLW1 105.00000000 W
======== CHANNEL f2 ========
SFO2 700.3628014 MHz
NUC2 1H
CPDPRG[2 waltz16
PCPD2 65.00 usec
PLW2 9.89999962 W
PLW12 0.15564001 W
PLW13 0.07837200 W
F2 - Processing parameters
SI 131072
SF 176.1056403 MHz
WDW EM
SSB 0
LB 1.00 Hz
GB 0
PC 1.40

^{19}F

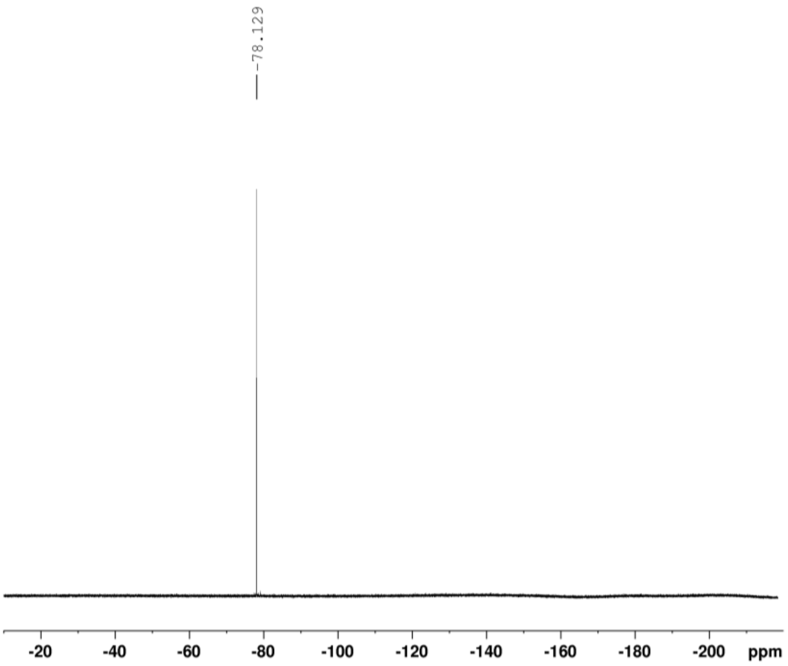


```
Current Data Parameters
NAME        67-TS-LI-026
EXPNO                191
PROCNO                 1

F2 - Acquisition Parameters
Date_           20240422
Time               12.59
INSTRUM            spect
PROBHD   5 mm PABBO BB/
PULPROG           zgflqn
TD                131072
SOLVENT            CDCl3
NS                    16
DS                     4
SWH            89285.711 Hz
FIDRES          0.681196 Hz
AQ             0.7340032 sec
RG                205.35
DW                 5.600 usec
DE                  6.50 usec
TE                 296.0 K
D1            1.00000000 sec
TD0                    1

======== CHANNEL f1 ========
SFO1         376.4324910 MHz
NUC1                 19F
P1                 14.65 usec
PLW1         20.00000000 W

F2 - Processing parameters
SI                 65536
SF           376.4701380 MHz
WDW                   EM
SSB                    0
LB                  0.30 Hz
GB                     0
PC                  1.00
```

S40

^{1}H

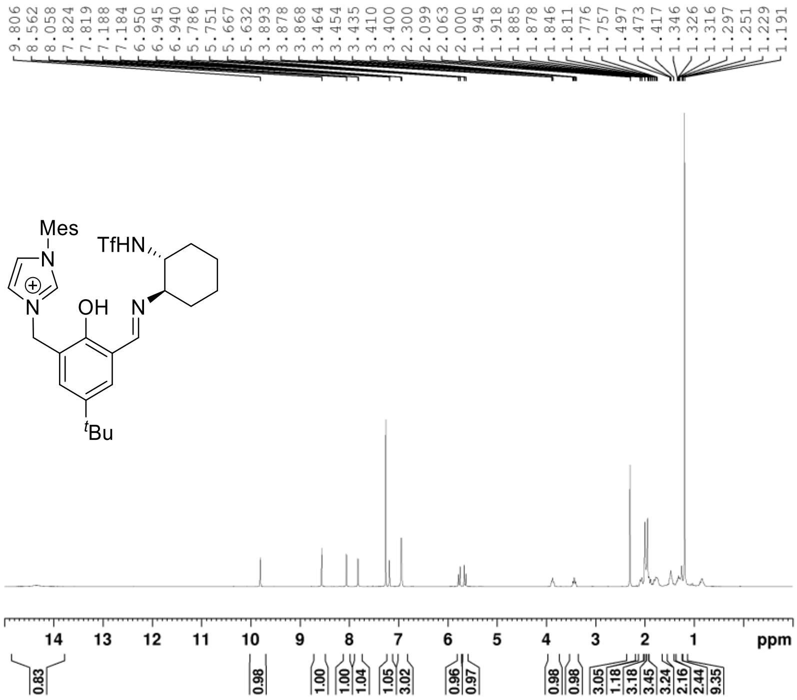


```
Current Data Parameters
NAME        67-TS-LI-022
EXPNO                630
PROCNO                 1

F2 - Acquisition Parameters
Date_           20240215
Time               17.48
INSTRUM            spect
PROBHD   5 mm PABBO BB/
PULPROG             zg30
TD                 65536
SOLVENT            CDCl3
NS                    16
DS                     2
SWH             8012.820 Hz
FIDRES          0.122266 Hz
AQ             4.0894465 sec
RG                182.64
DW                62.400 usec
DE                  6.50 usec
TE                 296.0 K
D1            1.00000000 sec
TD0                    1

======== CHANNEL f1 ========
SFO1         400.1024708 MHz
NUC1                  1H
P1                 13.70 usec
PLW1         12.00000000 W

F2 - Processing parameters
SI                 65536
SF           400.1000111 MHz
WDW                   EM
SSB                    0
LB                  0.30 Hz
GB                     0
PC                  1.00
```

^{13}C

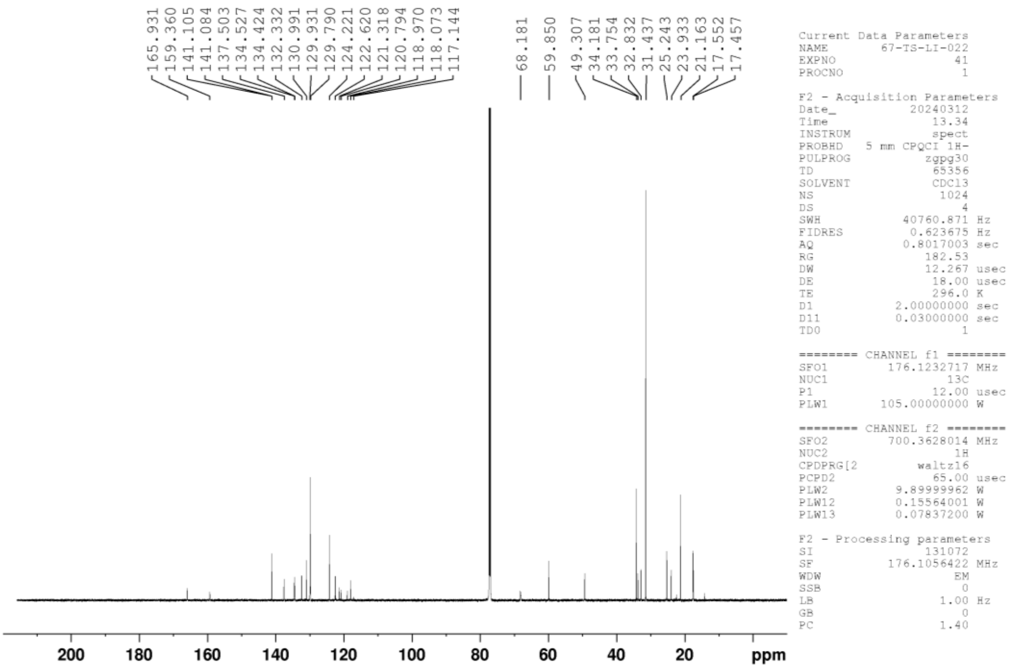
165.931
159.360
141.105
141.084
137.503
134.527
134.424
132.332
130.991
129.931
129.790
124.221
122.620
121.318
120.794
118.970
118.073
117.144
68.181
59.850
49.307
34.181
33.754
32.832
31.437
25.243
23.933
21.163
17.552
17.457
200 180 160 140 120 100 80 60 40 20 ppm
Current Data Parameters
NAME 67-TS-LI-022
EXPNO 41
PROCNO 1
F2 - Acquisition Parameters
Date_ 20240312
Time 13.34
INSTRUM spect
PROBHD 5 mm CPQCI 1H-
PULPROG zgpg30
TD 65356
SOLVENT CDCl3
NS 1024
DS 4
SWH 40760.871 Hz
FIDRES 0.623675 Hz
AQ 0.8017003 sec
RG 182.53
DW 12.267 usec
DE 18.00 usec
TE 296.0 K
D1 2.00000000 sec
D11 0.03000000 sec
TD0 1
======== CHANNEL f1 ========
SFO1 176.1232717 MHz
NUC1 13C
P1 12.00 usec
PLW1 105.00000000 W
======== CHANNEL f2 ========
SFO2 700.3628014 MHz
NUC2 1H
CPDPRG[2 waltz16
PCPD2 65.00 usec
PLW2 9.89999962 W
PLW12 0.15564001 W
PLW13 0.07837200 W
F2 - Processing parameters
SI 131072
SF 176.1056422 MHz
WDW EM
SSB 0
LB 1.00 Hz
GB 0
PC 1.40

^{19}F

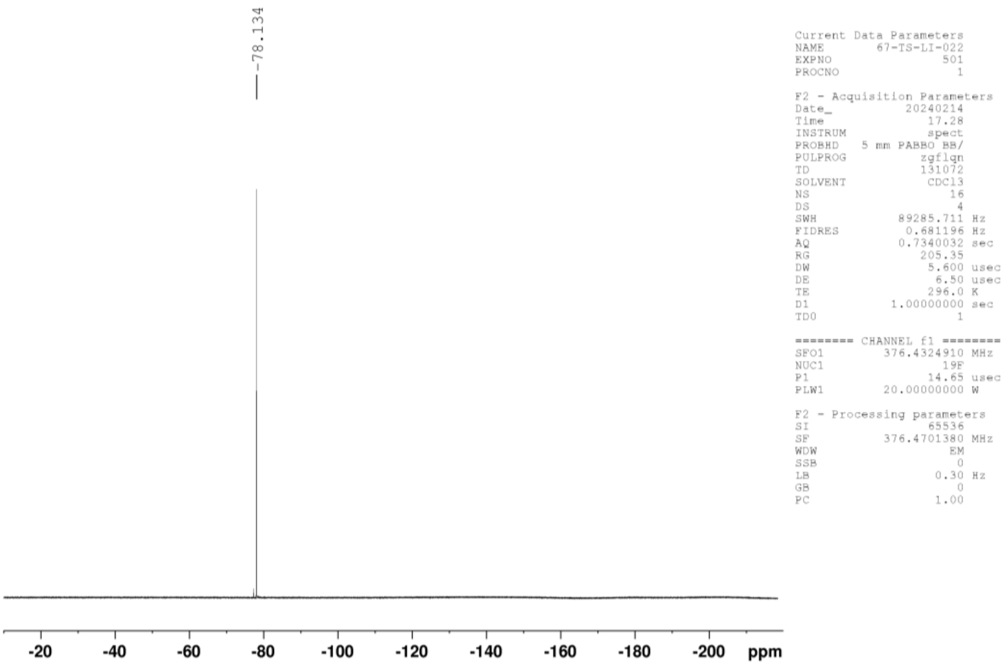
-78.134
-20 -40 -60 -80 -100 -120 -140 -160 -180 -200 ppm
Current Data Parameters
NAME 67-TS-LI-022
EXPNO 501
PROCNO 1
F2 - Acquisition Parameters
Date_ 20240214
Time 17.28
INSTRUM spect
PROBHD 5 mm PABBO BB/
PULPROG zgflqn
TD 131072
SOLVENT CDCl3
NS 16
DS 4
SWH 89285.711 Hz
FIDRES 0.681196 Hz
AQ 0.7340032 sec
RG 205.35
DW 5.600 usec
DE 6.50 usec
TE 296.0 K
D1 1.00000000 sec
TD0 1
======== CHANNEL f1 ========
SFO1 376.4324910 MHz
NUC1 19F
P1 14.65 usec
PLW1 20.00000000 W
F2 - Processing parameters
SI 65536
SF 376.4701380 MHz
WDW EM
SSB 0
LB 0.30 Hz
GB 0
PC 1.00

S41

^{1}H

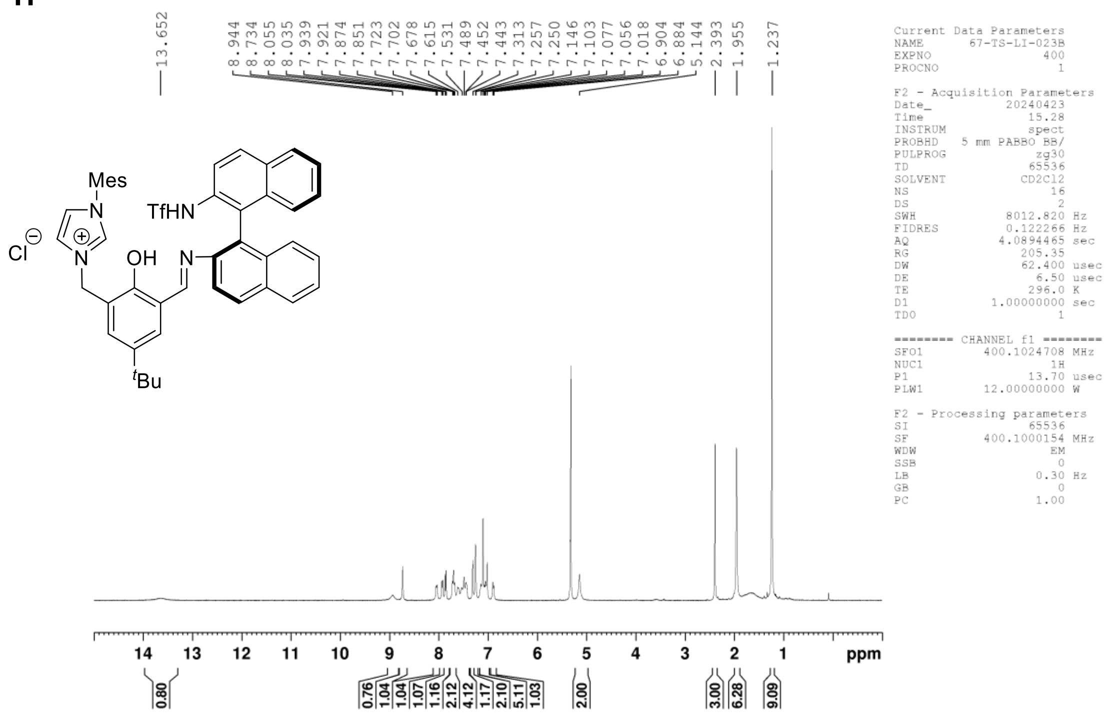

13.652
8.944
8.734
8.055
8.035
7.939
7.921
7.874
7.851
7.723
7.702
7.678
7.615
7.531
7.489
7.452
7.443
7.313
7.257
7.250
7.146
7.103
7.077
7.056
7.018
6.904
6.884
5.144
2.393
1.955
1.237
Mes
TfHN
Cl
OH
N
tBu
Current Data Parameters
NAME 67-TS-LI-023B
EXPNO 400
PROCNO 1
F2 - Acquisition Parameters
Date_ 20240423
Time 15.28
INSTRUM spect
PROBHD 5 mm PABBO BB/
PULPROG zg30
TD 65536
SOLVENT CD2Cl2
NS 16
DS 2
SWH 8012.820 Hz
FIDRES 0.122266 Hz
AQ 4.0894465 sec
RG 205.35
DW 62.400 usec
DE 6.50 usec
TE 296.0 K
D1 1.00000000 sec
TD0 1
======== CHANNEL f1 ========
SFO1 400.1024708 MHz
NUC1 1H
P1 13.70 usec
PLW1 12.00000000 W
F2 - Processing parameters
SI 65536
SF 400.1000154 MHz
WDW EM
SSB 0
LB 0.30 Hz
GB 0
PC 1.00
14 13 12 11 10 9 8 7 6 5 4 3 2 1 ppm
0.80
0.76
1.04
1.04
1.07
1.16
2.12
4.12
1.17
2.10
5.11
1.03
2.00
3.00
6.28
9.09

^{13}C

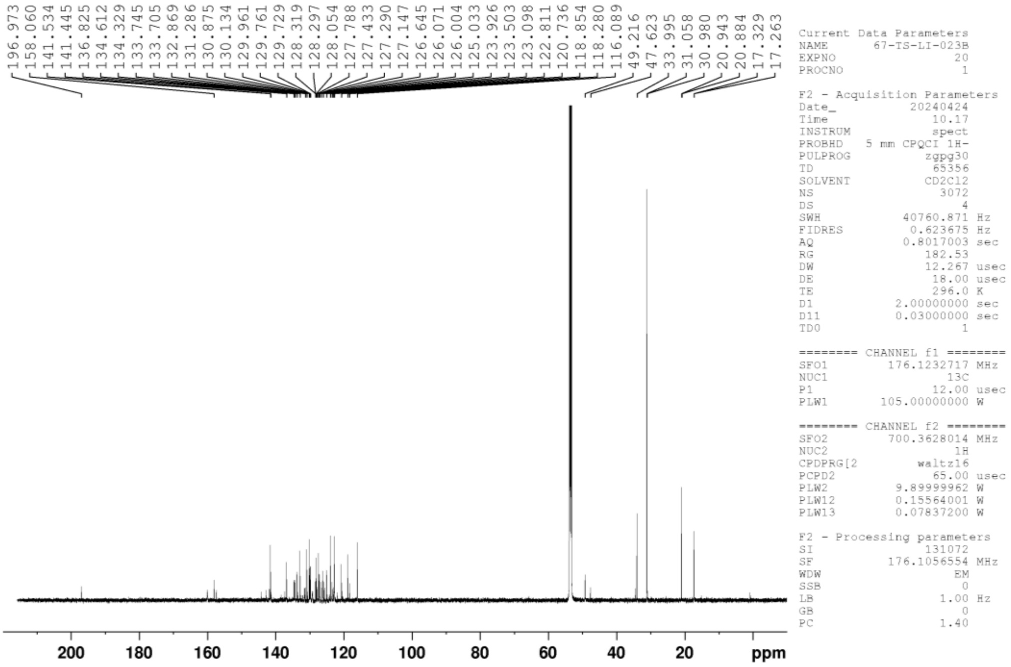

196.973
158.060
141.534
141.445
136.825
134.612
134.329
133.745
133.705
132.869
131.286
130.875
130.134
129.961
129.761
129.729
128.319
128.297
128.054
127.788
127.433
127.290
127.147
126.645
126.071
126.004
125.033
123.926
123.503
123.098
122.811
120.736
118.854
118.280
116.089
49.216
47.623
33.995
31.058
30.980
20.943
20.884
17.329
17.263
Current Data Parameters
NAME 67-TS-LI-023B
EXPNO 20
PROCNO 1
F2 - Acquisition Parameters
Date_ 20240424
Time 10.17
INSTRUM spect
PROBHD 5 mm CPQCI 1H-
PULPROG zgpg30
TD 65356
SOLVENT CD2Cl2
NS 3072
DS 4
SWH 40760.871 Hz
FIDRES 0.623675 Hz
AQ 0.8017003 sec
RG 182.53
DW 12.267 usec
DE 18.00 usec
TE 296.0 K
D1 2.00000000 sec
D11 0.03000000 sec
TD0 1
======== CHANNEL f1 ========
SFO1 176.1232717 MHz
NUC1 13C
P1 12.00 usec
PLW1 105.00000000 W
======== CHANNEL f2 ========
SFO2 700.3628014 MHz
NUC2 1H
CPDPRG[2 waltz16
PCPD2 65.00 usec
PLW2 9.89999962 W
PLW12 0.15564001 W
PLW13 0.07837200 W
F2 - Processing parameters
SI 131072
SF 176.1056554 MHz
WDW EM
SSB 0
LB 1.00 Hz
GB 0
PC 1.40
200 180 160 140 120 100 80 60 40 20 ppm

^{19}F

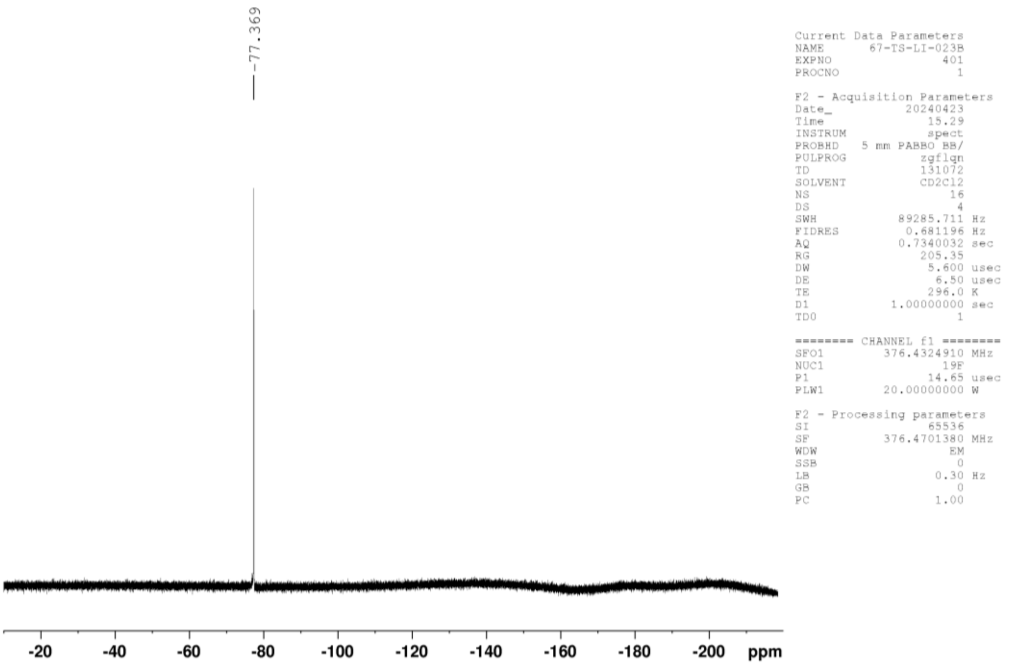
-77.369
Current Data Parameters
NAME 67-TS-LI-023B
EXPNO 401
PROCNO 1
F2 - Acquisition Parameters
Date_ 20240423
Time 15.29
INSTRUM spect
PROBHD 5 mm PABBO BB/
PULPROG zgflqn
TD 131072
SOLVENT CD2Cl2
NS 16
DS 4
SWH 89285.711 Hz
FIDRES 0.681196 Hz
AQ 0.7340032 sec
RG 205.35
DW 5.600 usec
DE 6.50 usec
TE 296.0 K
D1 1.00000000 sec
TD0 1
======== CHANNEL f1 ========
SFO1 376.4324910 MHz
NUC1 19F
P1 14.65 usec
PLW1 20.00000000 W
F2 - Processing parameters
SI 65536
SF 376.4701380 MHz
WDW EM
SSB 0
LB 0.30 Hz
GB 0
PC 1.00
-20 -40 -60 -80 -100 -120 -140 -160 -180 -200 ppm

S43

^{1}H

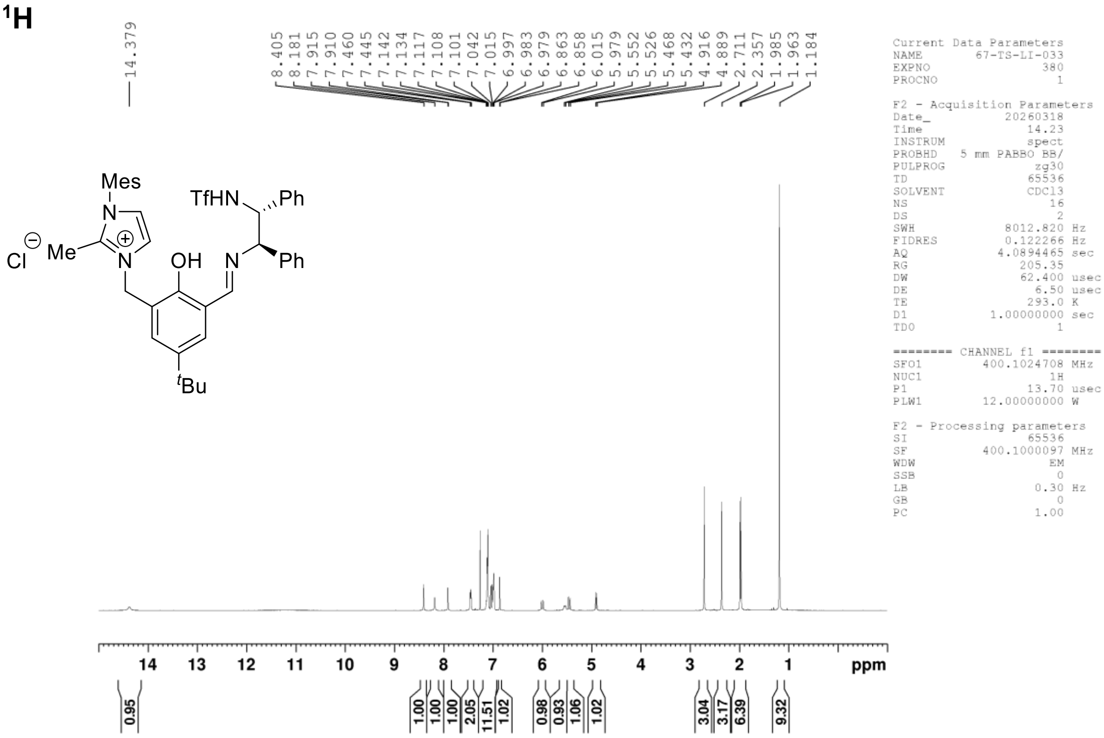
14.379
8.405 8.181 7.915 7.910 7.460 7.445 7.142 7.134 7.117 7.108 7.101 7.042 7.015 6.997 6.983 6.979 6.863 6.858 6.015 5.979 5.552 5.526 5.468 5.432 4.916 4.889 2.711 2.357 1.985 1.963 1.184
Mes
TfHN
Ph
Cl
Me
N
OH
N
Ph
tBu
Current Data Parameters
NAME 67-TS-LI-033
EXPNO 380
PROCNO 1
F2 - Acquisition Parameters
Date_ 20260318
Time 14.23
INSTRUM spect
PROBHD 5 mm PABBO BB/
PULPROG zg30
TD 65536
SOLVENT CDCl3
NS 16
DS 2
SWH 8012.820 Hz
FIDRES 0.122266 Hz
AQ 4.0894465 sec
RG 205.35
DW 62.400 usec
DE 6.50 usec
TE 293.0 K
D1 1.00000000 sec
TD0 1
======== CHANNEL f1 ========
SFO1 400.1024708 MHz
NUC1 1H
P1 13.70 usec
PLW1 12.00000000 W
F2 - Processing parameters
SI 65536
SF 400.1000097 MHz
WDW EM
SSB 0
LB 0.30 Hz
GB 0
PC 1.00
14 13 12 11 10 9 8 7 6 5 4 3 2 1 ppm
0.95 1.00 1.00 1.00 2.05 11.51 1.02 0.98 0.93 1.06 1.02 3.04 3.17 6.39 9.32

^{13}C

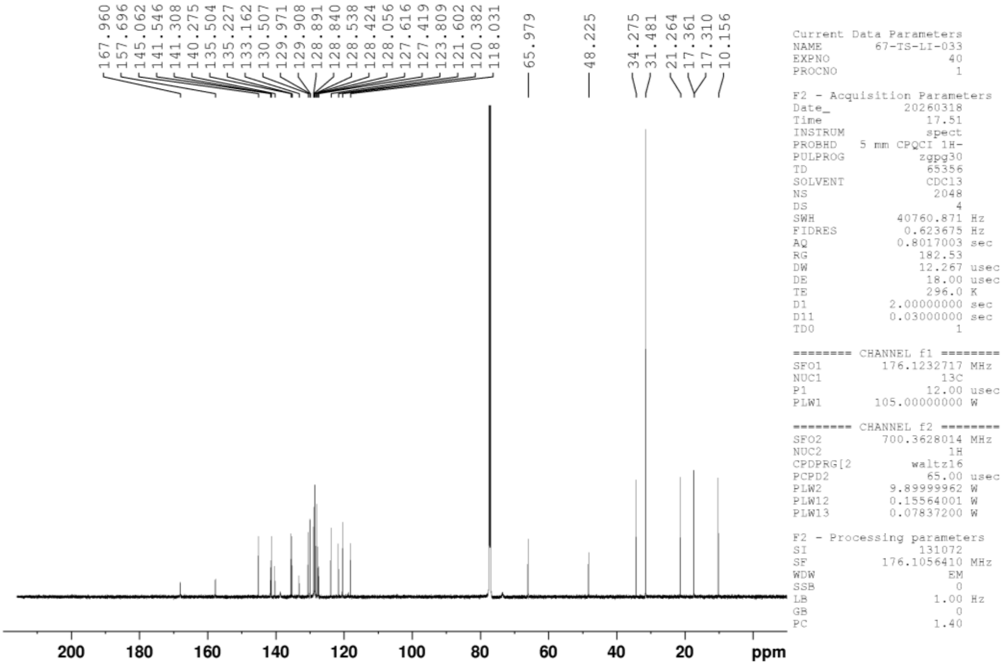
167.960
157.696
145.062
141.546
141.308
140.275
135.504
135.227
133.162
130.507
129.971
129.908
128.891
128.840
128.538
128.424
128.056
127.616
127.419
123.809
121.602
120.382
118.031
65.979
48.225
34.275
31.481
21.264
17.361
17.310
10.156
Current Data Parameters
NAME 67-TS-LI-033
EXPNO 40
PROCNO 1
F2 - Acquisition Parameters
Date_ 20260318
Time 17.51
INSTRUM spect
PROBHD 5 mm CPQCI 1H-
PULPROG zgpg30
TD 65356
SOLVENT CDCl3
NS 2048
DS 4
SWH 40760.871 Hz
FIDRES 0.623675 Hz
AQ 0.8017003 sec
RG 182.53
DW 12.267 usec
DE 18.00 usec
TE 296.0 K
D1 2.00000000 sec
D11 0.03000000 sec
TD0 1
======== CHANNEL f1 ========
SFO1 176.1232717 MHz
NUC1 13C
P1 12.00 usec
PLW1 105.00000000 W
======== CHANNEL f2 ========
SFO2 700.3628014 MHz
NUC2 1H
CPDPRG[2 waltz16
PCPD2 65.00 usec
PLW2 9.89999962 W
PLW12 0.15564001 W
PLW13 0.07837200 W
F2 - Processing parameters
SI 131072
SF 176.1056410 MHz
WDW EM
SSB 0
LB 1.00 Hz
GB 0
PC 1.40
200 180 160 140 120 100 80 60 40 20 ppm

^{19}F

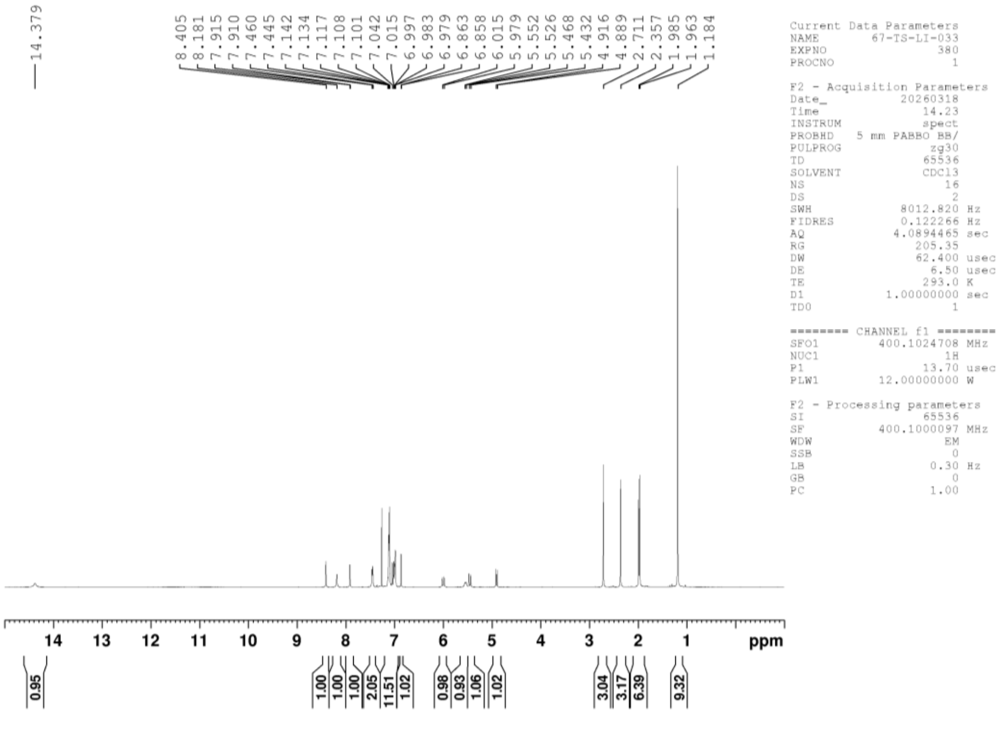
14.379
8.405
8.181
7.915
7.910
7.460
7.445
7.142
7.134
7.117
7.108
7.101
7.042
7.015
6.997
6.983
6.979
6.863
6.858
6.015
5.979
5.552
5.526
5.468
5.432
4.916
4.889
2.711
2.357
1.985
1.963
1.184
Current Data Parameters
NAME 67-TS-LI-033
EXPNO 380
PROCNO 1
F2 - Acquisition Parameters
Date_ 20260318
Time 14.23
INSTRUM spect
PROBHD 5 mm PABBO BB/
PULPROG zg30
TD 65536
SOLVENT CDCl3
NS 16
DS 2
SWH 8012.820 Hz
FIDRES 0.122266 Hz
AQ 4.0894465 sec
RG 205.35
DW 62.400 usec
DE 6.50 usec
TE 293.0 K
D1 1.00000000 sec
TD0 1
======== CHANNEL f1 ========
SFO1 400.1024708 MHz
NUC1 1H
P1 13.70 usec
PLW1 12.00000000 W
F2 - Processing parameters
SI 65536
SF 400.1000097 MHz
WDW EM
SSB 0
LB 0.30 Hz
GB 0
PC 1.00
14 13 12 11 10 9 8 7 6 5 4 3 2 1 ppm
0.95
1.00
1.00
1.00
2.05
11.51
1.02
0.98
0.93
1.06
1.02
3.04
3.17
6.39
9.32

S46

^{1}H

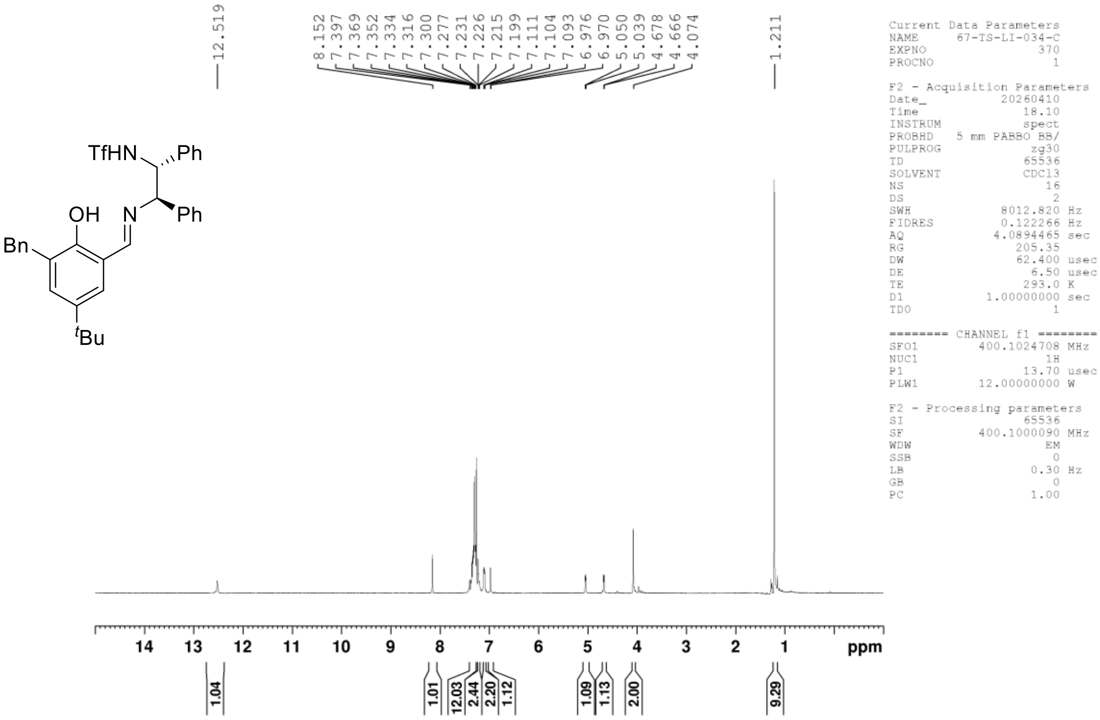
TfHN
Ph
OH
N
Ph
Bn
tBu
12.519
8.152
7.397
7.369
7.352
7.334
7.316
7.300
7.277
7.231
7.226
7.215
7.199
7.111
7.104
7.093
6.976
6.970
5.050
5.039
4.678
4.666
4.074
1.211
14 13 12 11 10 9 8 7 6 5 4 3 2 1 ppm
1.04
1.01
12.03
2.44
2.20
1.12
1.09
1.13
2.00
9.29
Current Data Parameters
NAME 67-TS-LI-034-C
EXPNO 370
PROCNO 1
F2 - Acquisition Parameters
Date_ 20260410
Time 18.10
INSTRUM spect
PROBHD 5 mm PABBO BB/
PULPROG zg30
TD 65536
SOLVENT CDCl3
NS 16
DS 2
SWH 8012.820 Hz
FIDRES 0.122266 Hz
AQ 4.0894465 sec
RG 205.35
DW 62.400 usec
DE 6.50 usec
TE 293.0 K
D1 1.00000000 sec
TD0 1
======== CHANNEL f1 ========
SFO1 400.1024708 MHz
NUC1 1H
P1 13.70 usec
PLW1 12.00000000 W
F2 - Processing parameters
SI 65536
SF 400.1000090 MHz
WDW EM
SSB 0
LB 0.30 Hz
GB 0
PC 1.00

^{13}C

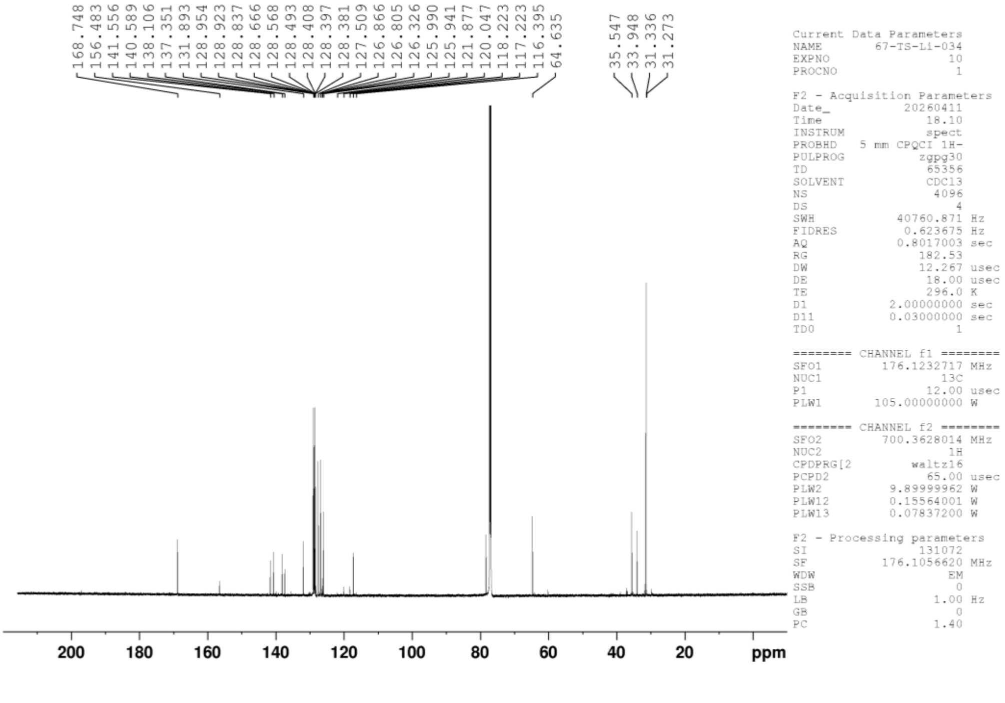
168.748
156.483
141.556
140.589
138.106
137.351
131.893
128.954
128.923
128.837
128.666
128.568
128.493
128.408
128.397
128.381
127.509
126.866
126.805
126.326
125.990
125.941
121.877
120.047
118.223
117.223
116.395
64.635
35.547
33.948
31.336
31.273
200 180 160 140 120 100 80 60 40 20 ppm
Current Data Parameters
NAME 67-TS-LI-034
EXPNO 10
PROCNO 1
F2 - Acquisition Parameters
Date_ 20260411
Time 18.10
INSTRUM spect
PROBHD 5 mm CPQCI 1H-
PULPROG zgpg30
TD 65356
SOLVENT CDCl3
NS 4096
DS 4
SWH 40760.871 Hz
FIDRES 0.623675 Hz
AQ 0.8017003 sec
RG 182.53
DW 12.267 usec
DE 18.00 usec
TE 296.0 K
D1 2.00000000 sec
D11 0.03000000 sec
TD0 1
======== CHANNEL f1 ========
SFO1 176.1232717 MHz
NUC1 13C
P1 12.00 usec
PLW1 105.00000000 W
======== CHANNEL f2 ========
SFO2 700.3628014 MHz
NUC2 1H
CPDPRG[2 waltz16
PCPD2 65.00 usec
PLW2 9.89999962 W
PLW12 0.15564001 W
PLW13 0.07837200 W
F2 - Processing parameters
SI 131072
SF 176.1056620 MHz
WDW EM
SSB 0
LB 1.00 Hz
GB 0
PC 1.40

^{19}F

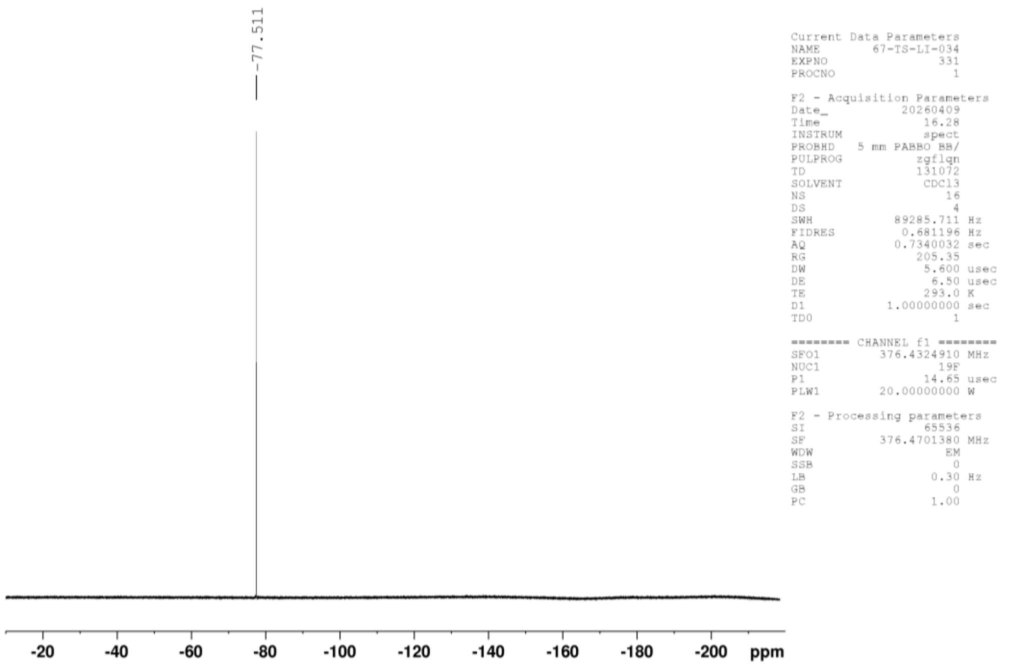

-77.511
Current Data Parameters
NAME 67-TS-LI-034
EXPNO 331
PROCNO 1
F2 - Acquisition Parameters
Date_ 20260409
Time 16.28
INSTRUM spect
PROBHD 5 mm PABBO BB/
PULPROG zgflqn
TD 131072
SOLVENT CDCl3
NS 16
DS 4
SWH 89285.711 Hz
FIDRES 0.681196 Hz
AQ 0.7340032 sec
RG 205.35
DW 5.600 usec
DE 6.50 usec
TE 293.0 K
D1 1.00000000 sec
TD0 1
======== CHANNEL f1 ========
SFO1 376.4324910 MHz
NUC1 19F
P1 14.65 usec
PLW1 20.00000000 W
F2 - Processing parameters
SI 65536
SF 376.4701380 MHz
WDW EM
SSB 0
LB 0.30 Hz
GB 0
PC 1.00
-20 -40 -60 -80 -100 -120 -140 -160 -180 -200 ppm

3aB

^{1}H

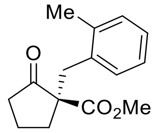

Me
O
CO2Me

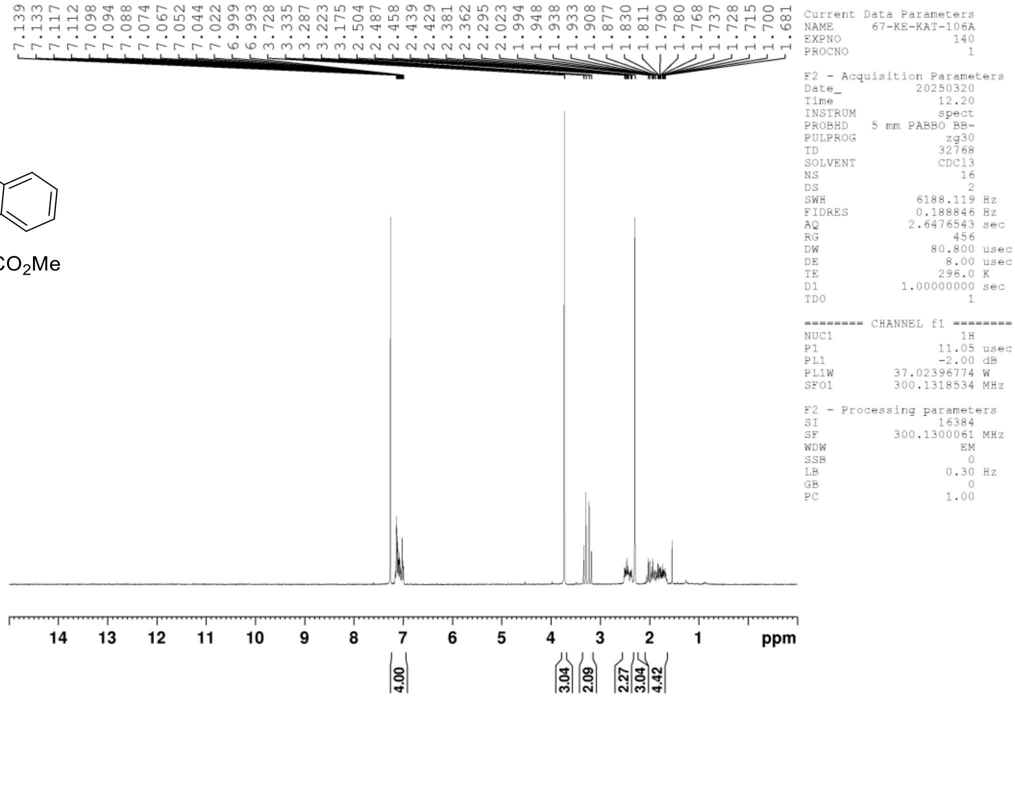

7.139 7.133 7.117 7.112 7.098 7.094 7.088 7.074 7.067 7.052 7.044 7.022 6.999 6.993 3.728 3.335 3.287 3.223 3.175 2.504 2.487 2.458 2.439 2.429 2.381 2.362 2.295 2.023 1.994 1.948 1.938 1.933 1.908 1.877 1.830 1.811 1.790 1.780 1.768 1.737 1.728 1.715 1.700 1.681
Current Data Parameters
NAME 67-KE-KAT-106A
EXPNO 140
PROCNO 1
F2 - Acquisition Parameters
Date_ 20250320
Time 12.20
INSTRUM spect
PROBHD 5 mm PABBO BB-
PULPROG zg30
TD 32768
SOLVENT CDCl3
NS 16
DS 2
SWH 6188.119 Hz
FIDRES 0.188846 Hz
AQ 2.6476543 sec
RG 456
DW 80.800 usec
DE 8.00 usec
TE 296.0 K
D1 1.00000000 sec
TD0 1
======== CHANNEL f1 ========
NUC1 1H
P1 11.05 usec
PL1 -2.00 dB
PL1W 37.02396774 W
SFO1 300.1318534 MHz
F2 - Processing parameters
SI 16384
SF 300.1300061 MHz
WDW EM
SSB 0
LB 0.30 Hz
GB 0
PC 1.00
14 13 12 11 10 9 8 7 6 5 4 3 2 1 ppm
4.00
3.04
2.09
2.27
3.04
4.42

[13]C

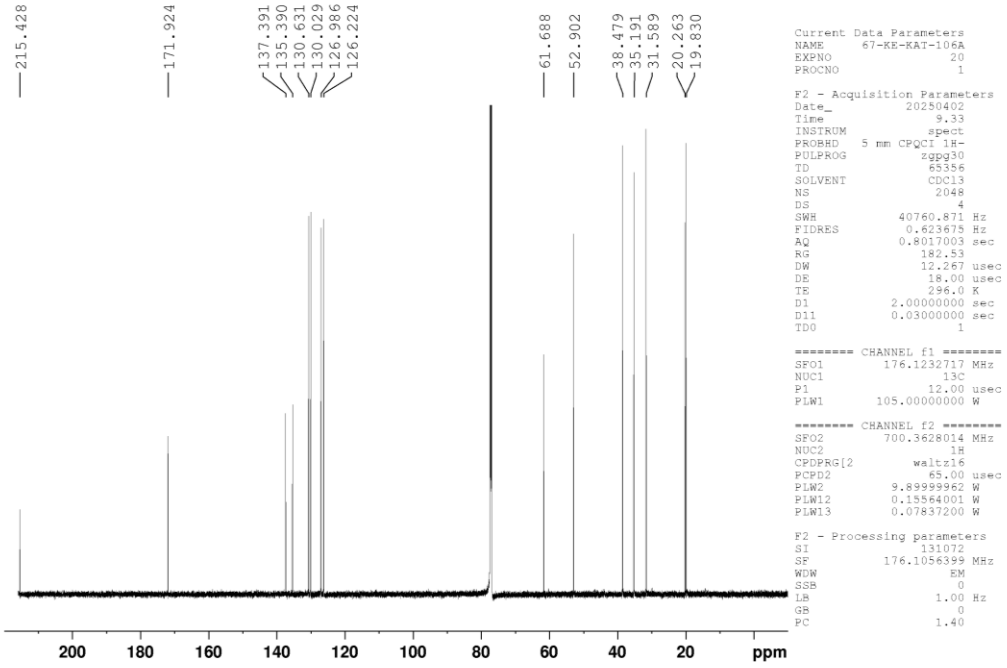
215.428
171.924
137.391
135.390
130.631
130.029
126.986
126.224
61.688
52.902
38.479
35.191
31.589
20.263
19.830
Current Data Parameters
NAME 67-KE-KAT-106A
EXPNO 20
PROCNO 1
F2 - Acquisition Parameters
Date_ 20250402
Time 9.33
INSTRUM spect
PROBHD 5 mm CPQCI 1H-
PULPROG zgpg30
TD 65356
SOLVENT CDCl3
NS 2048
DS 4
SWH 40760.871 Hz
FIDRES 0.623675 Hz
AQ 0.8017003 sec
RG 182.53
DW 12.267 usec
DE 18.00 usec
TE 296.0 K
D1 2.00000000 sec
D11 0.03000000 sec
TD0 1
======== CHANNEL f1 ========
SFO1 176.1232717 MHz
NUC1 13C
P1 12.00 usec
PLW1 105.00000000 W
======== CHANNEL f2 ========
SFO2 700.3628014 MHz
NUC2 1H
CPDPRG[2 waltz16
PCPD2 65.00 usec
PLW2 9.89999962 W
PLW12 0.15564001 W
PLW13 0.07837200 W
F2 - Processing parameters
SI 131072
SF 176.1056399 MHz
WDW EM
SSB 0
LB 1.00 Hz
GB 0
PC 1.40
200 180 160 140 120 100 80 60 40 20 ppm

3aD

[1]H

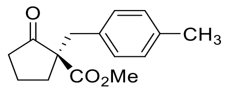
O
CH3
CO2Me

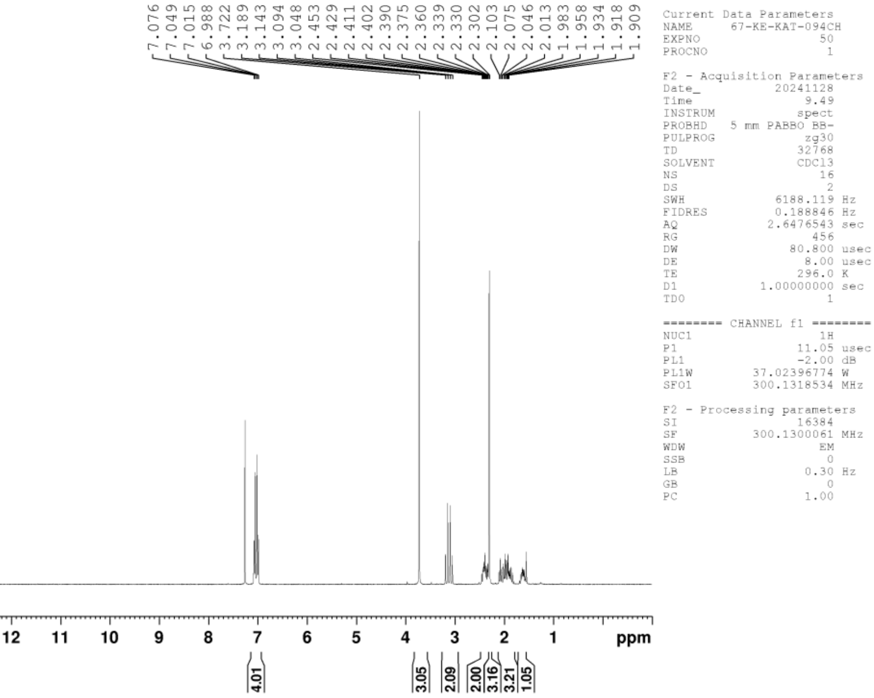
7.076
7.049
7.015
6.988
3.722
3.189
3.143
3.094
3.048
2.453
2.429
2.411
2.402
2.390
2.375
2.360
2.339
2.330
2.302
2.103
2.075
2.046
2.013
1.983
1.958
1.934
1.918
1.909
Current Data Parameters
NAME 67-KE-KAT-094CH
EXPNO 50
PROCNO 1
F2 - Acquisition Parameters
Date_ 20241128
Time 9.49
INSTRUM spect
PROBHD 5 mm PABBO BB-
PULPROG zg30
TD 32768
SOLVENT CDCl3
NS 16
DS 2
SWH 6188.119 Hz
FIDRES 0.188846 Hz
AQ 2.6476543 sec
RG 456
DW 80.800 usec
DE 8.00 usec
TE 296.0 K
D1 1.00000000 sec
TD0 1
======== CHANNEL f1 ========
NUC1 1H
P1 11.05 usec
PL1 -2.00 dB
PL1W 37.02396774 W
SFO1 300.1318534 MHz
F2 - Processing parameters
SI 16384
SF 300.1300061 MHz
WDW EM
SSB 0
LB 0.30 Hz
GB 0
PC 1.00
14 13 12 11 10 9 8 7 6 5 4 3 2 1 ppm
4.01
3.05
2.09
2.00
3.16
3.21
1.05

^{13}C

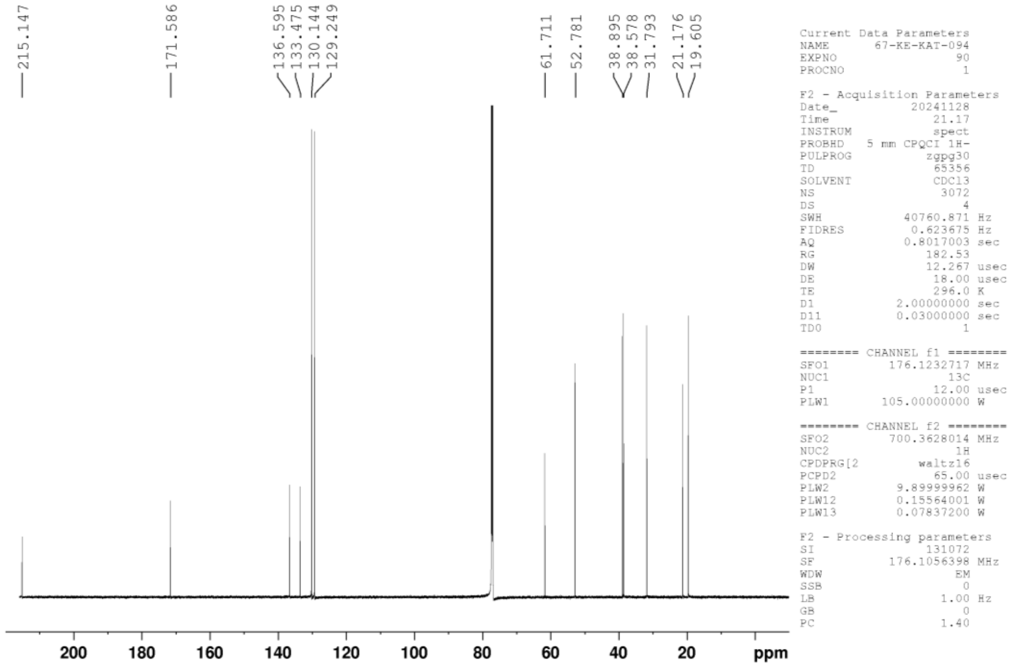
215.147
171.586
136.595
133.475
130.144
129.249
61.711
52.781
38.895
38.578
31.793
21.176
19.605
Current Data Parameters
NAME 67-KE-KAT-094
EXPNO 90
PROCNO 1
F2 - Acquisition Parameters
Date_ 20241128
Time 21.17
INSTRUM spect
PROBHD 5 mm CPQCI 1H-
PULPROG zgpg30
TD 65356
SOLVENT CDCl3
NS 3072
DS 4
SWH 40760.871 Hz
FIDRES 0.623675 Hz
AQ 0.8017003 sec
RG 182.53
DW 12.267 usec
DE 18.00 usec
TE 296.0 K
D1 2.00000000 sec
D11 0.03000000 sec
TD0 1
======== CHANNEL f1 ========
SFO1 176.1232717 MHz
NUC1 13C
P1 12.00 usec
PLW1 105.00000000 W
======== CHANNEL f2 ========
SFO2 700.3628014 MHz
NUC2 1H
CPDPRG[2 waltz16
PCPD2 65.00 usec
PLW2 9.89999962 W
PLW12 0.15564001 W
PLW13 0.07837200 W
F2 - Processing parameters
SI 131072
SF 176.1056398 MHz
WDW EM
SSB 0
LB 1.00 Hz
GB 0
PC 1.40
200
180
160
140
120
100
80
60
40
20
ppm

3aE

^{1}H

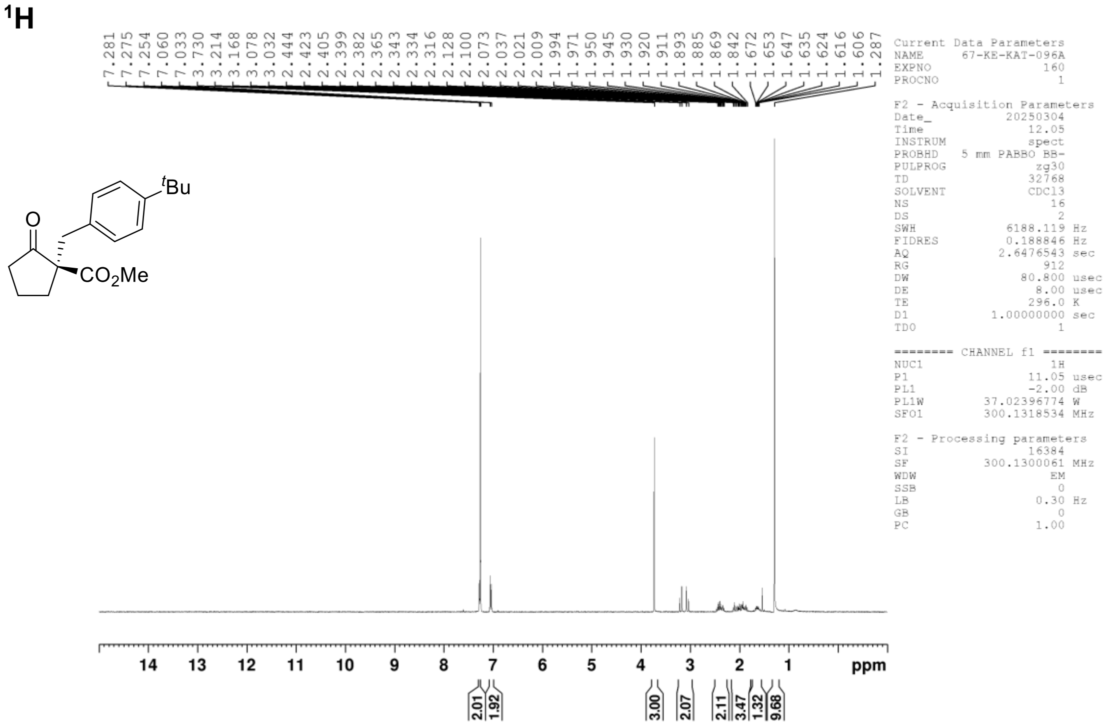
tBu
O
CO2Me
7.281
7.275
7.254
7.060
7.033
3.730
3.214
3.168
3.078
3.032
2.444
2.423
2.405
2.399
2.382
2.365
2.343
2.334
2.316
2.128
2.100
2.073
2.037
2.021
2.009
1.994
1.971
1.950
1.945
1.930
1.920
1.911
1.893
1.885
1.869
1.842
1.672
1.653
1.647
1.635
1.624
1.616
1.606
1.287
Current Data Parameters
NAME 67-KE-KAT-096A
EXPNO 160
PROCNO 1
F2 - Acquisition Parameters
Date_ 20250304
Time 12.05
INSTRUM spect
PROBHD 5 mm PABBO BB-
PULPROG zg30
TD 32768
SOLVENT CDCl3
NS 16
DS 2
SWH 6188.119 Hz
FIDRES 0.188846 Hz
AQ 2.6476543 sec
RG 912
DW 80.800 usec
DE 8.00 usec
TE 296.0 K
D1 1.00000000 sec
TD0 1
======== CHANNEL f1 ========
NUC1 1H
P1 11.05 usec
PL1 -2.00 dB
PL1W 37.02396774 W
SFO1 300.1318534 MHz
F2 - Processing parameters
SI 16384
SF 300.1300061 MHz
WDW EM
SSB 0
LB 0.30 Hz
GB 0
PC 1.00
14
13
12
11
10
9
8
7
6
5
4
3
2
1
ppm
2.01
1.92
3.00
2.07
2.11
3.47
1.32
9.68

^{13}C

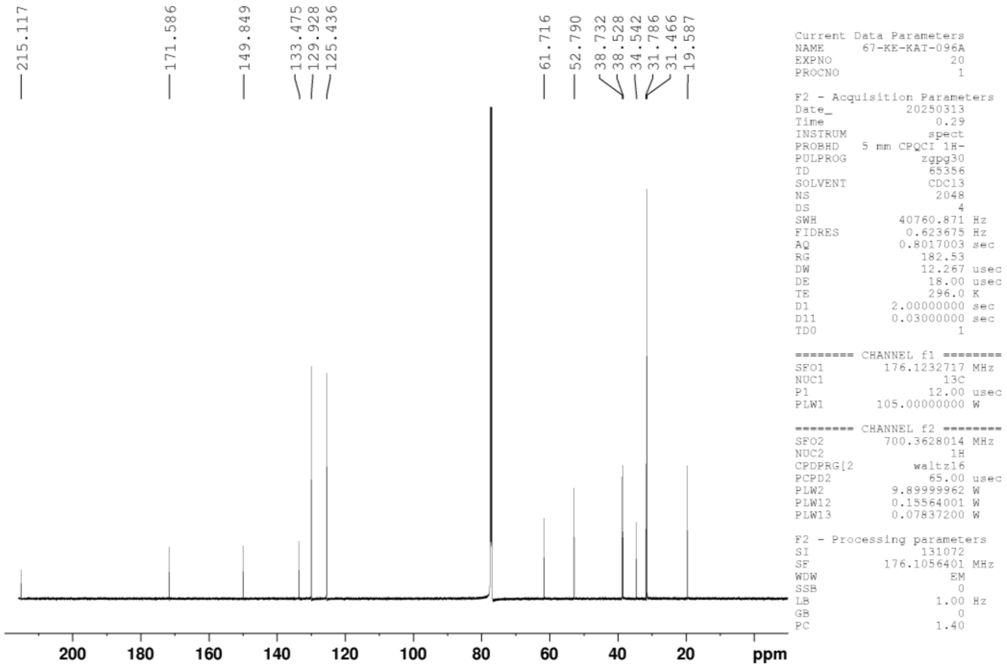
215.117
171.586
149.849
133.475
129.928
125.436
61.716
52.790
38.732
38.528
34.542
31.786
31.466
19.587
Current Data Parameters
NAME 67-KE-KAT-096A
EXPNO 20
PROCNO 1
F2 - Acquisition Parameters
Date_ 20250313
Time 0.29
INSTRUM spect
PROBHD 5 mm CPQCI 1H-
PULPROG zgpg30
TD 65356
SOLVENT CDCl3
NS 2048
DS 4
SWH 40760.871 Hz
FIDRES 0.623675 Hz
AQ 0.8017003 sec
RG 182.53
DW 12.267 usec
DE 18.00 usec
TE 296.0 K
D1 2.00000000 sec
D11 0.03000000 sec
TD0 1
======== CHANNEL f1 ========
SFO1 176.1232717 MHz
NUC1 13C
P1 12.00 usec
PLW1 105.00000000 W
======== CHANNEL f2 ========
SFO2 700.3628014 MHz
NUC2 1H
CPDPRG[2 waltz16
PCPD2 65.00 usec
PLW2 9.89999962 W
PLW12 0.15564001 W
PLW13 0.07837200 W
F2 - Processing parameters
SI 131072
SF 176.1056401 MHz
WDW EM
SSB 0
LB 1.00 Hz
GB 0
PC 1.40
200
180
160
140
120
100
80
60
40
20
ppm

3al

^{1}H

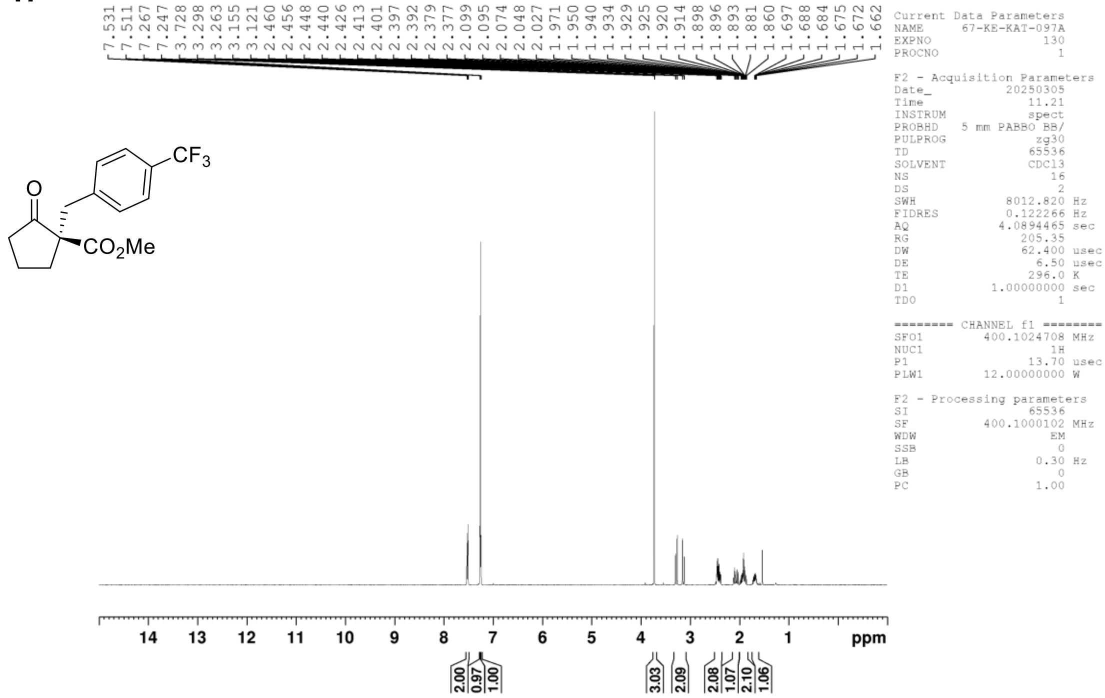
7.531
7.511
7.267
7.247
3.728
3.298
3.263
3.155
3.121
2.460
2.456
2.448
2.440
2.426
2.413
2.401
2.397
2.392
2.379
2.377
2.099
2.095
2.074
2.048
2.027
1.971
1.950
1.940
1.934
1.929
1.925
1.920
1.914
1.898
1.896
1.893
1.881
1.860
1.697
1.688
1.684
1.675
1.672
1.662
Current Data Parameters
NAME 67-KE-KAT-097A
EXPNO 130
PROCNO 1
F2 - Acquisition Parameters
Date_ 20250305
Time 11.21
INSTRUM spect
PROBHD 5 mm PABBO BB/
PULPROG zg30
TD 65536
SOLVENT CDCl3
NS 16
DS 2
SWH 8012.820 Hz
FIDRES 0.122266 Hz
AQ 4.0894465 sec
RG 205.35
DW 62.400 usec
DE 6.50 usec
TE 296.0 K
D1 1.00000000 sec
TD0 1
======== CHANNEL f1 ========
SFO1 400.1024708 MHz
NUC1 1H
P1 13.70 usec
PLW1 12.00000000 W
F2 - Processing parameters
SI 65536
SF 400.1000102 MHz
WDW EM
SSB 0
LB 0.30 Hz
GB 0
PC 1.00
O
CF3
CO2Me
14
13
12
11
10
9
8
7
6
5
4
3
2
1
ppm
2.00
0.97
1.00
3.03
2.09
2.08
1.07
2.10
1.06

^{13}C

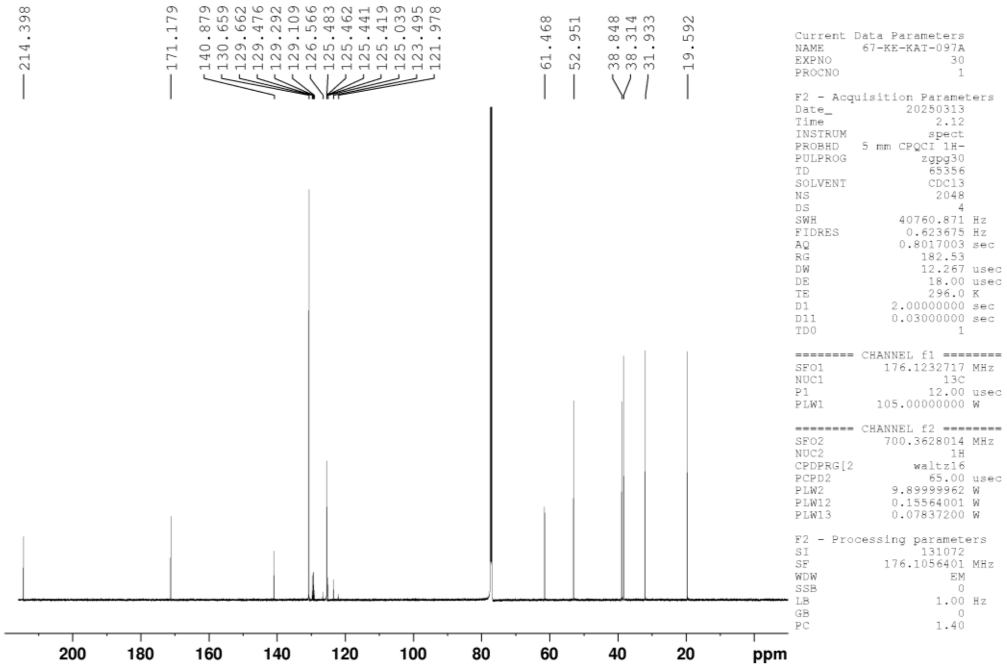

214.398
171.179
140.879
130.659
129.662
129.476
129.292
129.109
126.566
125.483
125.462
125.441
125.419
125.039
123.495
121.978
61.468
52.951
38.848
38.314
31.933
19.592
200
180
160
140
120
100
80
60
40
20
ppm
Current Data Parameters
NAME 67-KE-KAT-097A
EXPNO 30
PROCNO 1
F2 - Acquisition Parameters
Date_ 20250313
Time 2.12
INSTRUM spect
PROBHD 5 mm CPQCI 1H-
PULPROG zgpg30
TD 65356
SOLVENT CDCl3
NS 2048
DS 4
SWH 40760.871 Hz
FIDRES 0.623675 Hz
AQ 0.8017003 sec
RG 182.53
DW 12.267 usec
DE 18.00 usec
TE 296.0 K
D1 2.00000000 sec
D11 0.03000000 sec
TD0 1
======== CHANNEL f1 ========
SFO1 176.1232717 MHz
NUC1 13C
P1 12.00 usec
PLW1 105.00000000 W
======== CHANNEL f2 ========
SFO2 700.3628014 MHz
NUC2 1H
CPDPRG[2 waltz16
PCPD2 65.00 usec
PLW2 9.89999962 W
PLW12 0.15564001 W
PLW13 0.07837200 W
F2 - Processing parameters
SI 131072
SF 176.1056401 MHz
WDW EM
SSB 0
LB 1.00 Hz
GB 0
PC 1.40

^{19}F

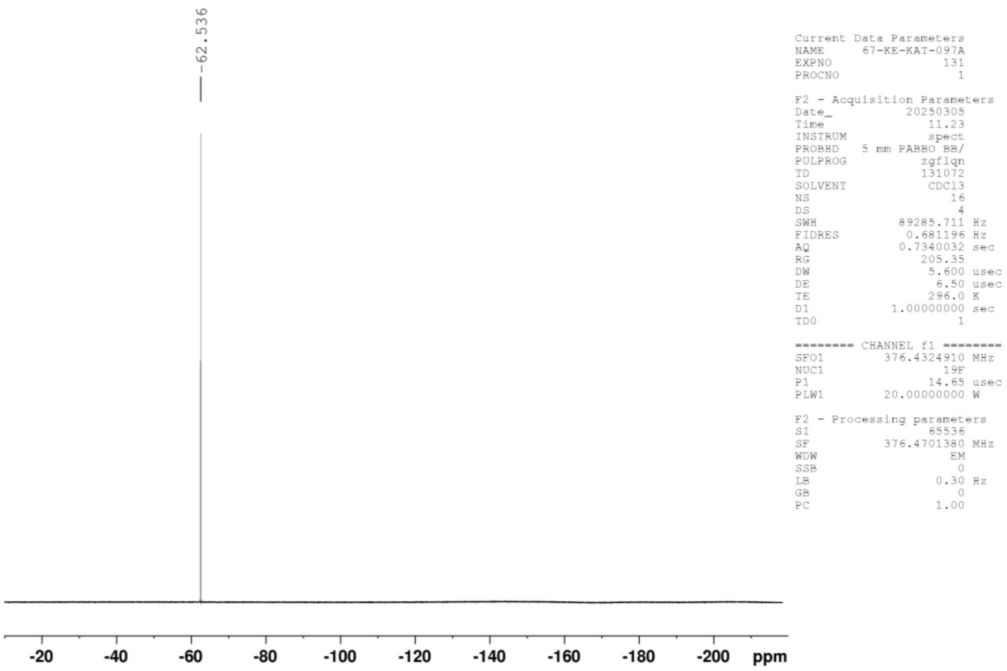

-62.536
-20
-40
-60
-80
-100
-120
-140
-160
-180
-200
ppm
Current Data Parameters
NAME 67-KE-KAT-097A
EXPNO 131
PROCNO 1
F2 - Acquisition Parameters
Date_ 20250305
Time 11.23
INSTRUM spect
PROBHD 5 mm PABBO BB/
PULPROG zgflqn
TD 131072
SOLVENT CDCl3
NS 16
DS 4
SWH 89285.711 Hz
FIDRES 0.681196 Hz
AQ 0.7340032 sec
RG 205.35
DW 5.600 usec
DE 6.50 usec
TE 296.0 K
D1 1.00000000 sec
TD0 1
======== CHANNEL f1 ========
SFO1 376.4324910 MHz
NUC1 19F
P1 14.65 usec
PLW1 20.00000000 W
F2 - Processing parameters
SI 65536
SF 376.4701380 MHz
WDW EM
SSB 0
LB 0.30 Hz
GB 0
PC 1.00

3aL

^{1}H

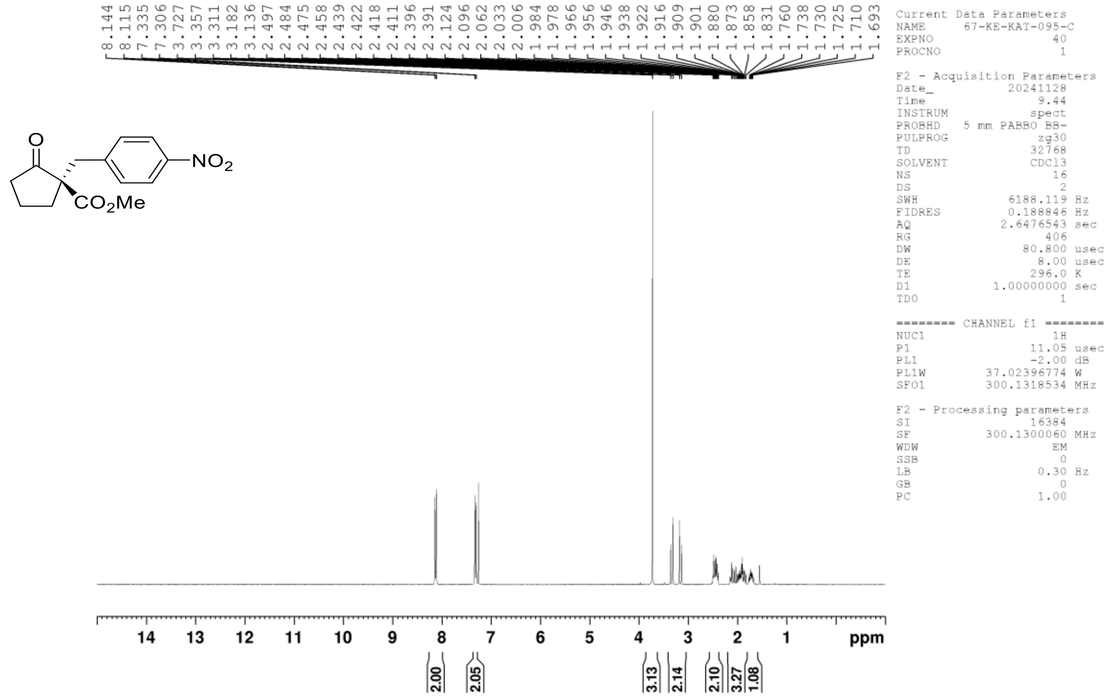
8.144
8.115
7.335
7.306
3.727
3.357
3.311
3.182
3.136
2.497
2.484
2.475
2.458
2.439
2.422
2.418
2.411
2.396
2.391
2.124
2.096
2.062
2.033
2.006
1.984
1.978
1.966
1.956
1.946
1.938
1.922
1.916
1.909
1.901
1.880
1.873
1.858
1.831
1.760
1.738
1.730
1.725
1.710
1.693
Current Data Parameters
NAME 67-KE-KAT-095-C
EXPNO 40
PROCNO 1
F2 - Acquisition Parameters
Date_ 20241128
Time 9.44
INSTRUM spect
PROBHD 5 mm PABBO BB-
PULPROG zg30
TD 32768
SOLVENT CDCl3
NS 16
DS 2
SWH 6188.119 Hz
FIDRES 0.188846 Hz
AQ 2.6476543 sec
RG 406
DW 80.800 usec
DE 8.00 usec
TE 296.0 K
D1 1.00000000 sec
TD0 1
======== CHANNEL f1 ========
NUC1 1H
P1 11.05 usec
PL1 -2.00 dB
PL1W 37.02396774 W
SFO1 300.1318534 MHz
F2 - Processing parameters
SI 16384
SF 300.1300060 MHz
WDW EM
SSB 0
LB 0.30 Hz
GB 0
PC 1.00
14 13 12 11 10 9 8 7 6 5 4 3 2 1 ppm
2.00
2.05
3.13
2.14
2.10
3.27
1.08

^{13}C

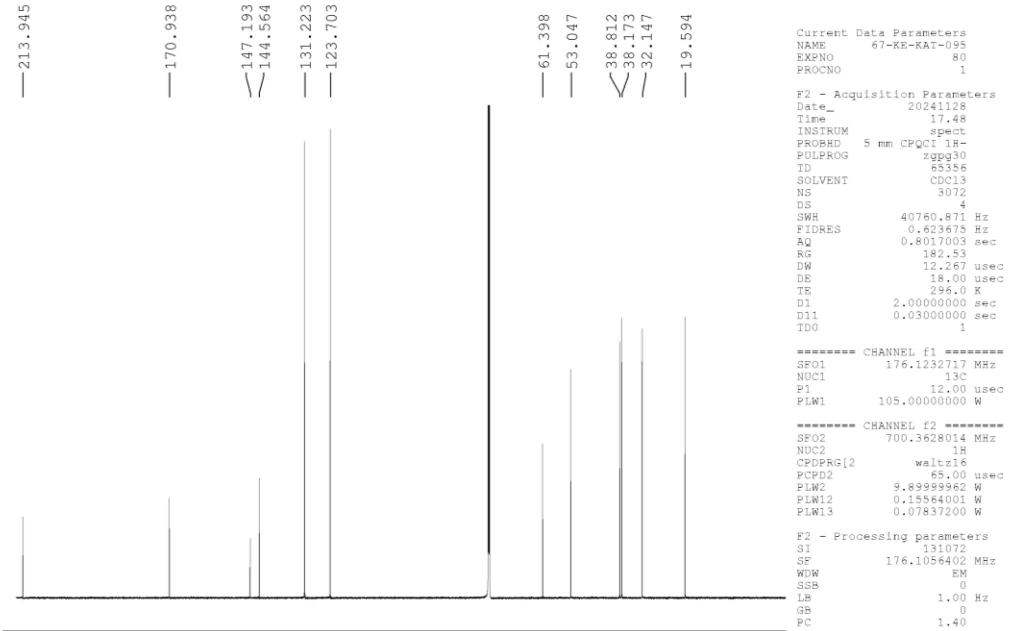
213.945
170.938
147.193
144.564
131.223
123.703
61.398
53.047
38.812
38.173
32.147
19.594
Current Data Parameters
NAME 67-KE-KAT-095
EXPNO 80
PROCNO 1
F2 - Acquisition Parameters
Date_ 20241128
Time 17.48
INSTRUM spect
PROBHD 5 mm CPQCI 1H-
PULPROG zgpg30
TD 65356
SOLVENT CDCl3
NS 3072
DS 4
SWH 40760.871 Hz
FIDRES 0.623675 Hz
AQ 0.8017003 sec
RG 182.53
DW 12.267 usec
DE 18.00 usec
TE 296.0 K
D1 2.00000000 sec
D11 0.03000000 sec
TD0 1
======== CHANNEL f1 ========
SFO1 176.1232717 MHz
NUC1 13C
P1 12.00 usec
PLW1 105.00000000 W
======== CHANNEL f2 ========
SFO2 700.3628014 MHz
NUC2 1H
CPDPRG[2 waltz16
PCPD2 65.00 usec
PLW2 9.89999962 W
PLW12 0.15564001 W
PLW13 0.07837200 W
F2 - Processing parameters
SI 131072
SF 176.1056402 MHz
WDW EM
SSB 0
LB 1.00 Hz
GB 0
PC 1.40
200 180 160 140 120 100 80 60 40 20 ppm

3aN

^{1}H

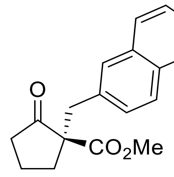

O
CO2Me

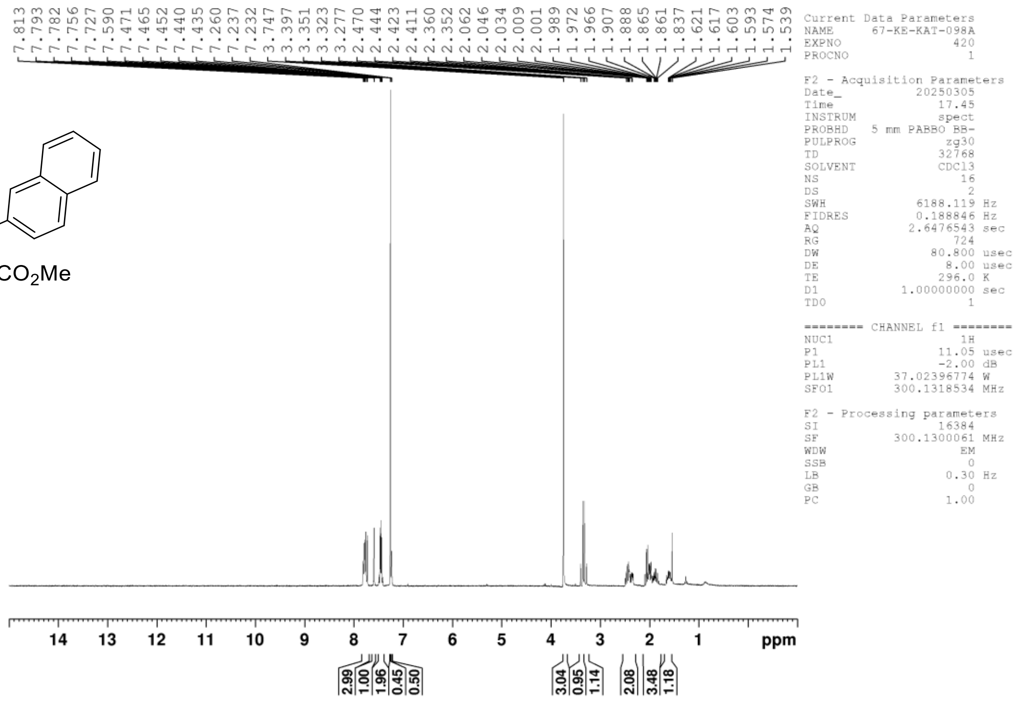

7.813 7.793 7.782 7.756 7.727 7.590 7.471 7.465 7.452 7.440 7.435 7.260 7.237 7.232 3.747 3.397 3.351 3.323 3.277 2.470 2.444 2.423 2.411 2.360 2.352 2.062 2.046 2.034 2.009 2.001 1.989 1.972 1.966 1.907 1.888 1.865 1.861 1.837 1.621 1.617 1.603 1.593 1.574 1.539
Current Data Parameters
NAME 67-KE-KAT-098A
EXPNO 420
PROCNO 1
F2 - Acquisition Parameters
Date_ 20250305
Time 17.45
INSTRUM spect
PROBHD 5 mm PABBO BB-
PULPROG zg30
TD 32768
SOLVENT CDCl3
NS 16
DS 2
SWH 6188.119 Hz
FIDRES 0.188846 Hz
AQ 2.6476543 sec
RG 724
DW 80.800 usec
DE 8.00 usec
TE 296.0 K
D1 1.00000000 sec
TD0 1
======== CHANNEL f1 ========
NUC1 1H
P1 11.05 usec
PL1 -2.00 dB
PL1W 37.02396774 W
SFO1 300.1318534 MHz
F2 - Processing parameters
SI 16384
SF 300.1300061 MHz
WDW EM
SSB 0
LB 0.30 Hz
GB 0
PC 1.00
14 13 12 11 10 9 8 7 6 5 4 3 2 1 ppm
2.99 1.00 1.96 0.45 0.50 3.04 0.95 1.14 2.08 3.48 1.18

^{13}C

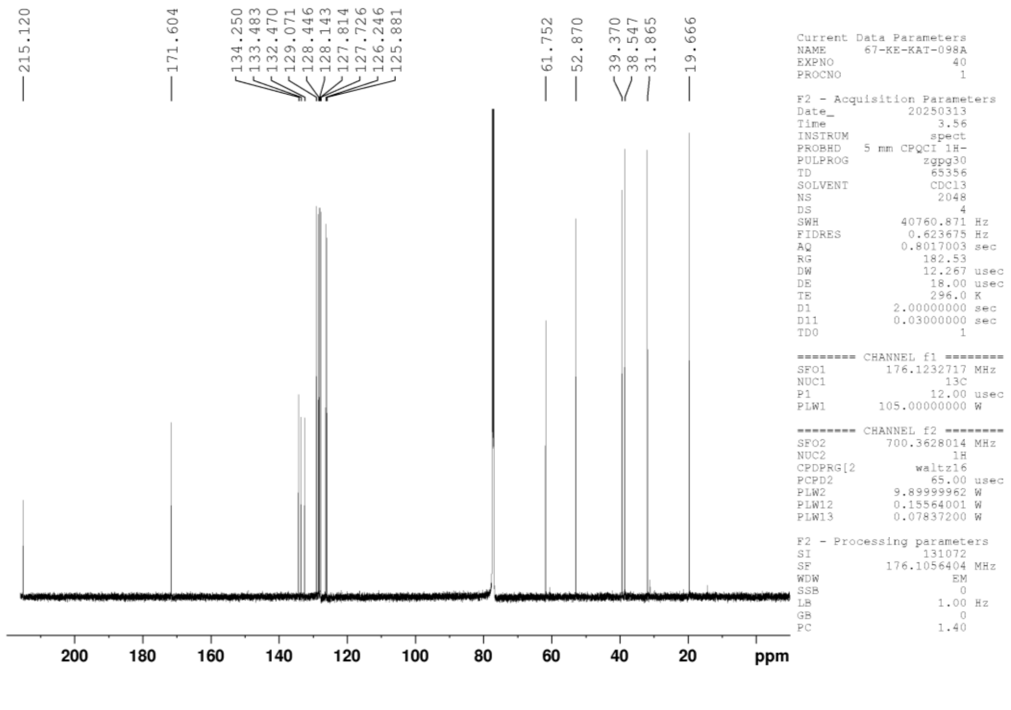

215.120 171.604 134.250 133.483 132.470 129.071 128.446 128.143 127.814 127.726 126.246 125.881 61.752 52.870 39.370 38.547 31.865 19.666
Current Data Parameters
NAME 67-KE-KAT-098A
EXPNO 40
PROCNO 1
F2 - Acquisition Parameters
Date_ 20250313
Time 3.56
INSTRUM spect
PROBHD 5 mm CPQCI 1H-
PULPROG zgpg30
TD 65356
SOLVENT CDCl3
NS 2048
DS 4
SWH 40760.871 Hz
FIDRES 0.623675 Hz
AQ 0.8017003 sec
RG 182.53
DW 12.267 usec
DE 18.00 usec
TE 296.0 K
D1 2.00000000 sec
D11 0.03000000 sec
TD0 1
======== CHANNEL f1 ========
SFO1 176.1232717 MHz
NUC1 13C
P1 12.00 usec
PLW1 105.00000000 W
======== CHANNEL f2 ========
SFO2 700.3628014 MHz
NUC2 1H
CPDPRG[2 waltz16
PCPD2 65.00 usec
PLW2 9.89999962 W
PLW12 0.15564001 W
PLW13 0.07837200 W
F2 - Processing parameters
SI 131072
SF 176.1056404 MHz
WDW EM
SSB 0
LB 1.00 Hz
GB 0
PC 1.40
200 180 160 140 120 100 80 60 40 20 ppm

3aJ

^{1}H

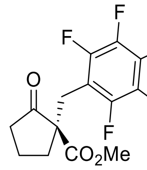
F
F
F
F
F
O
CO2Me

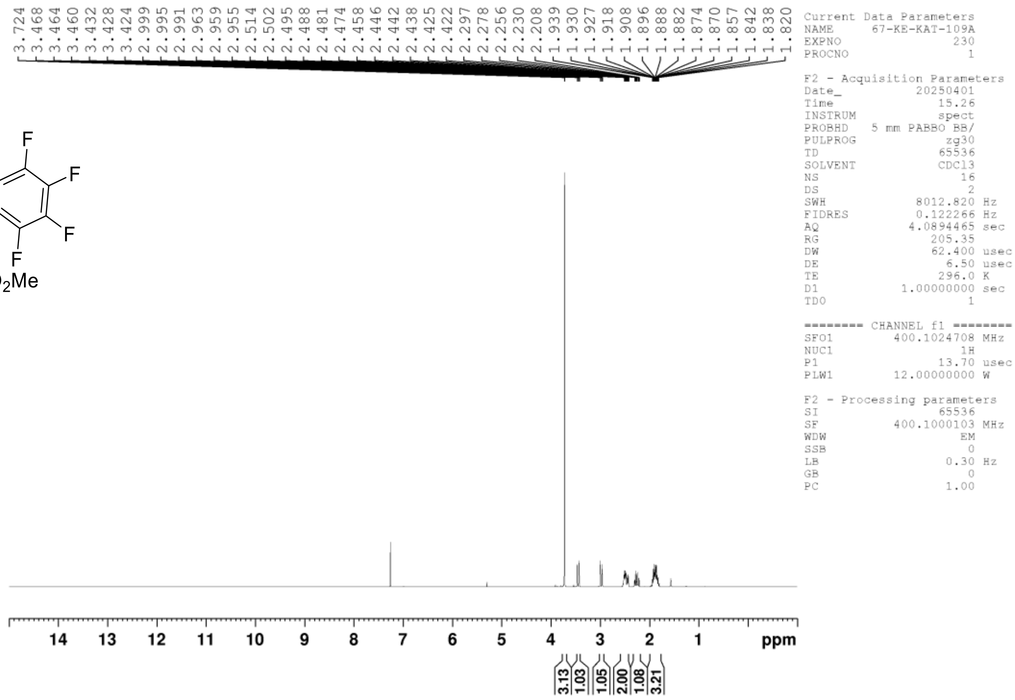
3.724 3.468 3.464 3.460 3.432 3.428 3.424 2.999 2.995 2.991 2.963 2.959 2.955 2.514 2.502 2.495 2.488 2.481 2.474 2.458 2.446 2.442 2.438 2.425 2.422 2.297 2.278 2.256 2.230 2.208 1.939 1.930 1.927 1.918 1.908 1.896 1.888 1.882 1.874 1.870 1.857 1.842 1.838 1.820
14 13 12 11 10 9 8 7 6 5 4 3 2 1 ppm
3.13 1.03 1.05 2.00 1.08 3.21
Current Data Parameters
NAME 67-KE-KAT-109A
EXPNO 230
PROCNO 1
F2 - Acquisition Parameters
Date_ 20250401
Time 15.26
INSTRUM spect
PROBHD 5 mm PABBO BB/
PULPROG zg30
TD 65536
SOLVENT CDCl3
NS 16
DS 2
SWH 8012.820 Hz
FIDRES 0.122266 Hz
AQ 4.0894465 sec
RG 205.35
DW 62.400 usec
DE 6.50 usec
TE 296.0 K
D1 1.00000000 sec
TD0 1
======== CHANNEL f1 ========
SFO1 400.1024708 MHz
NUC1 1H
P1 13.70 usec
PLW1 12.00000000 W
F2 - Processing parameters
SI 65536
SF 400.1000103 MHz
WDW EM
SSB 0
LB 0.30 Hz
GB 0
PC 1.00

^{13}C

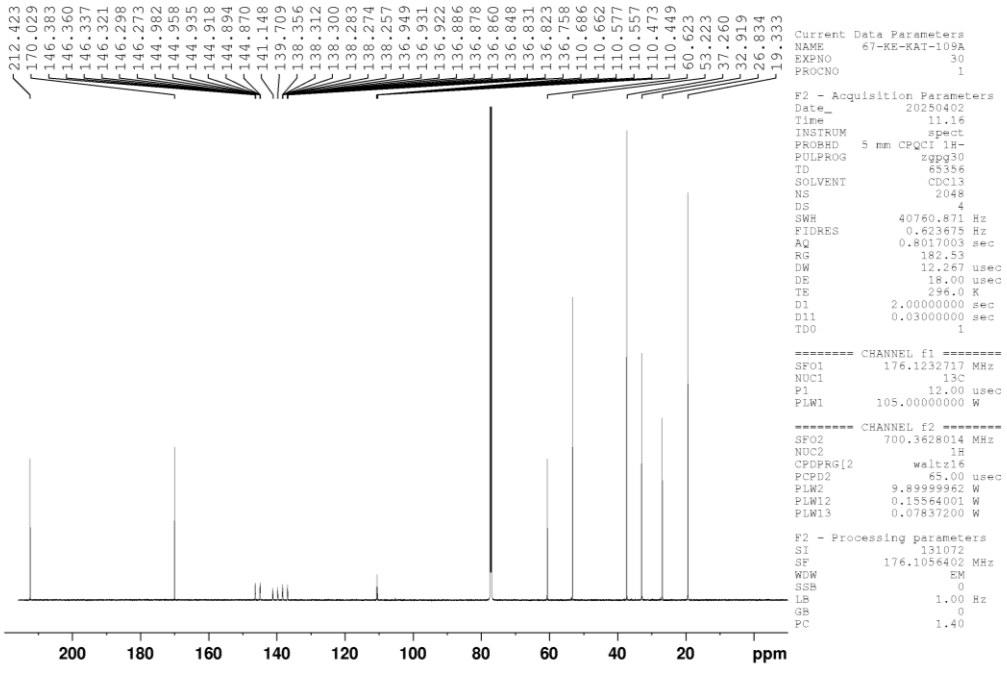
212.423 170.029 146.383 146.360 146.337 146.321 146.298 146.273 144.982 144.958 144.935 144.918 144.894 144.870 141.148 139.709 138.356 138.312 138.300 138.283 138.274 138.257 136.949 136.931 136.922 136.886 136.878 136.860 136.848 136.831 136.823 136.758 110.686 110.662 110.577 110.557 110.473 110.449 60.623 53.223 37.260 32.919 26.834 19.333
200 180 160 140 120 100 80 60 40 20 ppm
Current Data Parameters
NAME 67-KE-KAT-109A
EXPNO 30
PROCNO 1
F2 - Acquisition Parameters
Date_ 20250402
Time 11.16
INSTRUM spect
PROBHD 5 mm CPQCI 1H-
PULPROG zgpg30
TD 65356
SOLVENT CDCl3
NS 2048
DS 4
SWH 40760.871 Hz
FIDRES 0.623675 Hz
AQ 0.8017003 sec
RG 182.53
DW 12.267 usec
DE 18.00 usec
TE 296.0 K
D1 2.00000000 sec
D11 0.03000000 sec
TD0 1
======== CHANNEL f1 ========
SFO1 176.1232717 MHz
NUC1 13C
P1 12.00 usec
PLW1 105.00000000 W
======== CHANNEL f2 ========
SFO2 700.3628014 MHz
NUC2 1H
CPDPRG[2 waltz16
PCPD2 65.00 usec
PLW2 9.89999962 W
PLW12 0.15564001 W
PLW13 0.07837200 W
F2 - Processing parameters
SI 131072
SF 176.1056402 MHz
WDW EM
SSB 0
LB 1.00 Hz
GB 0
PC 1.40

^{19}F

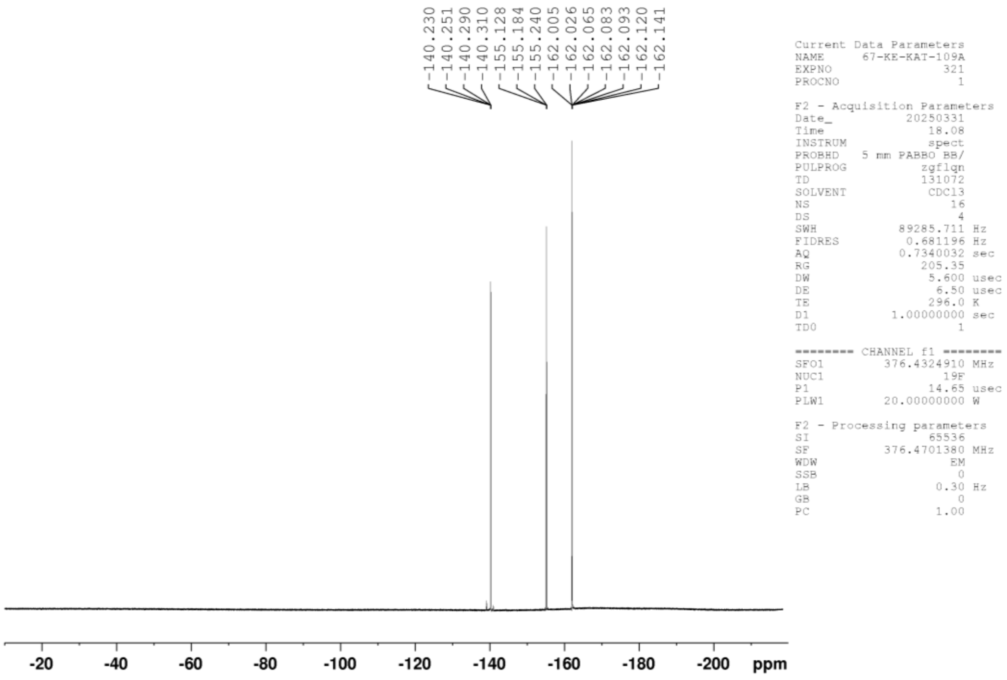

-140.230
-140.251
-140.290
-140.310
-155.128
-155.184
-155.240
-162.005
-162.026
-162.065
-162.083
-162.093
-162.120
-162.141
Current Data Parameters
NAME 67-KE-KAT-109A
EXPNO 321
PROCNO 1
F2 - Acquisition Parameters
Date_ 20250331
Time 18.08
INSTRUM spect
PROBHD 5 mm PABBO BB/
PULPROG zgflqn
TD 131072
SOLVENT CDCl3
NS 16
DS 4
SWH 89285.711 Hz
FIDRES 0.681196 Hz
AQ 0.7340032 sec
RG 205.35
DW 5.600 usec
DE 6.50 usec
TE 296.0 K
D1 1.00000000 sec
TD0 1
======== CHANNEL f1 ========
SFO1 376.4324910 MHz
NUC1 19F
P1 14.65 usec
PLW1 20.00000000 W
F2 - Processing parameters
SI 65536
SF 376.4701380 MHz
WDW EM
SSB 0
LB 0.30 Hz
GB 0
PC 1.00
-20 -40 -60 -80 -100 -120 -140 -160 -180 -200 ppm

3aO

^{1}H

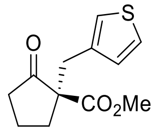

O
S
CO2Me

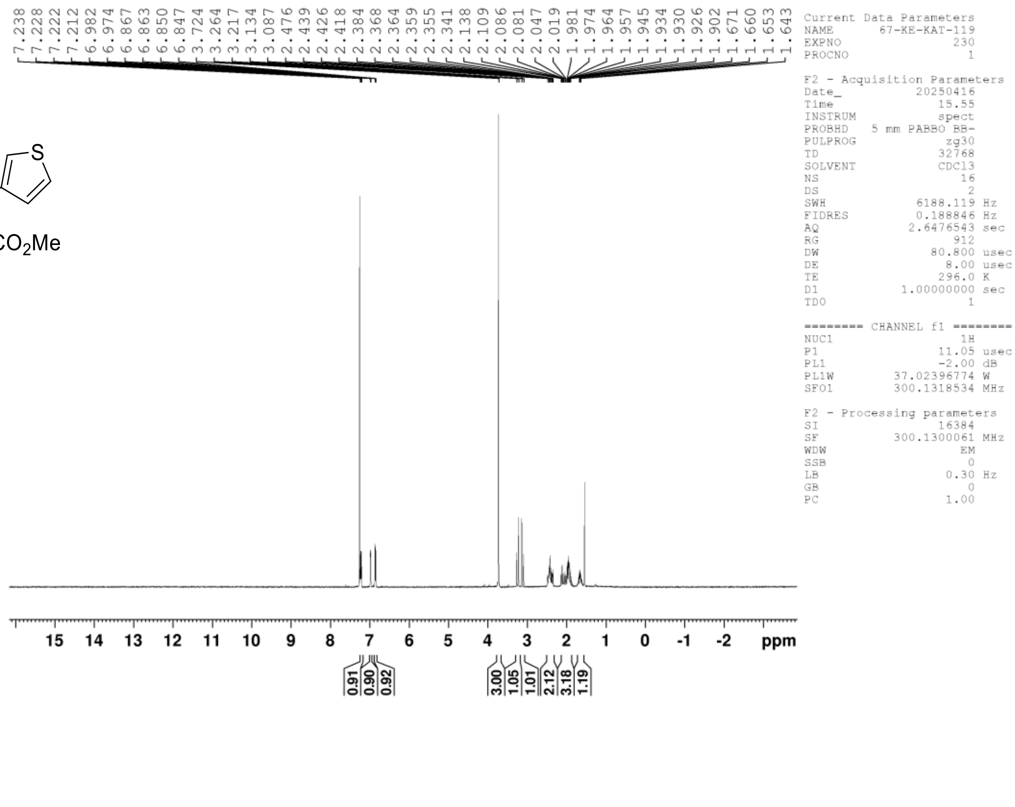

7.238
7.228
7.222
7.212
6.982
6.974
6.867
6.863
6.850
6.847
3.724
3.264
3.217
3.134
3.087
2.476
2.439
2.426
2.418
2.384
2.368
2.364
2.359
2.355
2.341
2.138
2.109
2.086
2.081
2.047
2.019
1.981
1.974
1.964
1.957
1.945
1.934
1.930
1.926
1.902
1.671
1.660
1.653
1.643
Current Data Parameters
NAME 67-KE-KAT-119
EXPNO 230
PROCNO 1
F2 - Acquisition Parameters
Date_ 20250416
Time 15.55
INSTRUM spect
PROBHD 5 mm PABBO BB-
PULPROG zg30
TD 32768
SOLVENT CDCl3
NS 16
DS 2
SWH 6188.119 Hz
FIDRES 0.188846 Hz
AQ 2.6476543 sec
RG 912
DW 80.800 usec
DE 8.00 usec
TE 296.0 K
D1 1.00000000 sec
TD0 1
======== CHANNEL f1 ========
NUC1 1H
P1 11.05 usec
PL1 -2.00 dB
PL1W 37.02396774 W
SFO1 300.1318534 MHz
F2 - Processing parameters
SI 16384
SF 300.1300061 MHz
WDW EM
SSB 0
LB 0.30 Hz
GB 0
PC 1.00
15 14 13 12 11 10 9 8 7 6 5 4 3 2 1 0 -1 -2 ppm
0.91
0.90
0.92
3.00
1.05
1.01
2.12
3.18
1.19

¹³C

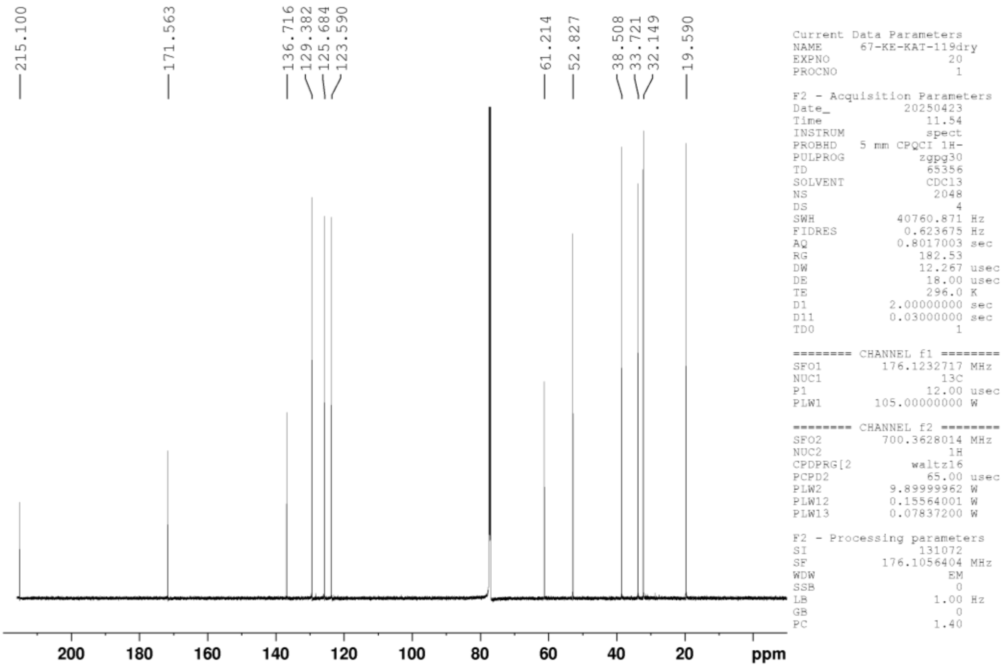
215.100
171.563
136.716
129.382
125.684
123.590
61.214
52.827
38.508
33.721
32.149
19.590
Current Data Parameters
NAME 67-KE-KAT-119dry
EXPNO 20
PROCNO 1
F2 - Acquisition Parameters
Date_ 20250423
Time 11.54
INSTRUM spect
PROBHD 5 mm CPQCI 1H-
PULPROG zgpg30
TD 65356
SOLVENT CDCl3
NS 2048
DS 4
SWH 40760.871 Hz
FIDRES 0.623675 Hz
AQ 0.8017003 sec
RG 182.53
DW 12.267 usec
DE 18.00 usec
TE 296.0 K
D1 2.00000000 sec
D11 0.03000000 sec
TD0 1
======== CHANNEL f1 ========
SFO1 176.1232717 MHz
NUC1 13C
P1 12.00 usec
PLW1 105.00000000 W
======== CHANNEL f2 ========
SFO2 700.3628014 MHz
NUC2 1H
CPDPRG[2 waltz16
PCPD2 65.00 usec
PLW2 9.89999962 W
PLW12 0.15564001 W
PLW13 0.07837200 W
F2 - Processing parameters
SI 131072
SF 176.1056404 MHz
WDW EM
SSB 0
LB 1.00 Hz
GB 0
PC 1.40
200 180 160 140 120 100 80 60 40 20 ppm

3aP

¹H

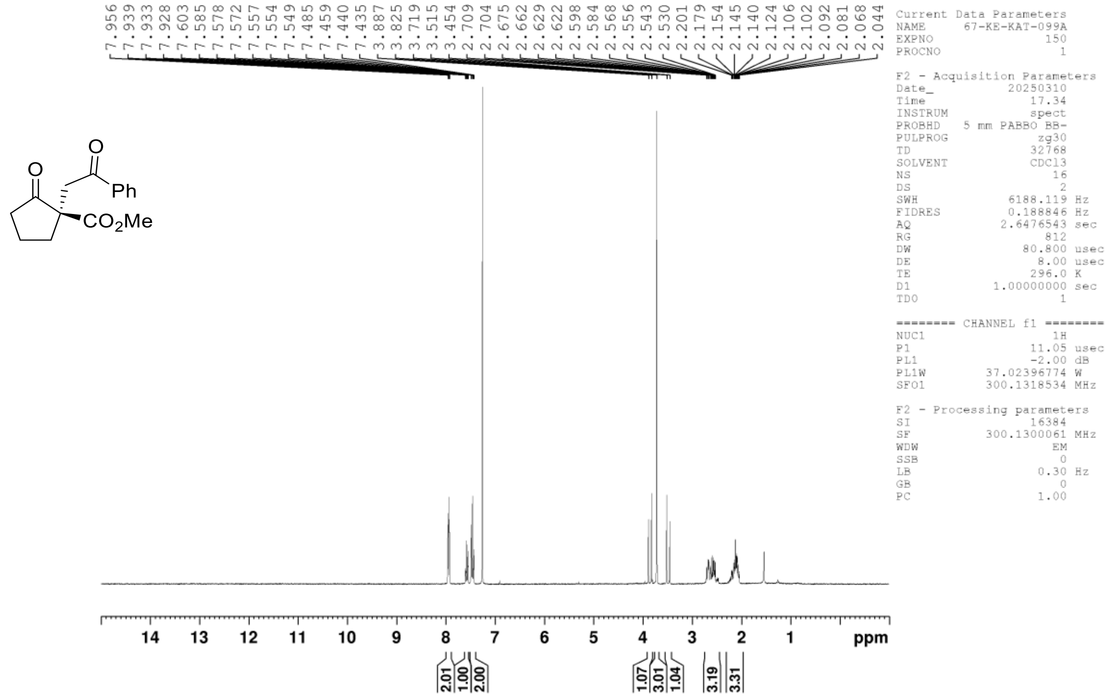
7.956
7.939
7.933
7.928
7.603
7.585
7.578
7.572
7.557
7.554
7.549
7.485
7.459
7.440
7.435
3.887
3.825
3.719
3.515
3.454
2.709
2.704
2.675
2.662
2.629
2.622
2.598
2.584
2.568
2.556
2.543
2.530
2.201
2.179
2.154
2.145
2.140
2.124
2.106
2.102
2.092
2.081
2.068
2.044
O
O
Ph
CO2Me
Current Data Parameters
NAME 67-KE-KAT-099A
EXPNO 150
PROCNO 1
F2 - Acquisition Parameters
Date_ 20250310
Time 17.34
INSTRUM spect
PROBHD 5 mm PABBO BB-
PULPROG zg30
TD 32768
SOLVENT CDCl3
NS 16
DS 2
SWH 6188.119 Hz
FIDRES 0.188846 Hz
AQ 2.6476543 sec
RG 812
DW 80.800 usec
DE 8.00 usec
TE 296.0 K
D1 1.00000000 sec
TD0 1
======== CHANNEL f1 ========
NUC1 1H
P1 11.05 usec
PL1 -2.00 dB
PL1W 37.02396774 W
SFO1 300.1318534 MHz
F2 - Processing parameters
SI 16384
SF 300.1300061 MHz
WDW EM
SSB 0
LB 0.30 Hz
GB 0
PC 1.00
14 13 12 11 10 9 8 7 6 5 4 3 2 1 ppm
2.01
1.00
2.00
1.07
3.01
1.04
3.19
3.31

13C

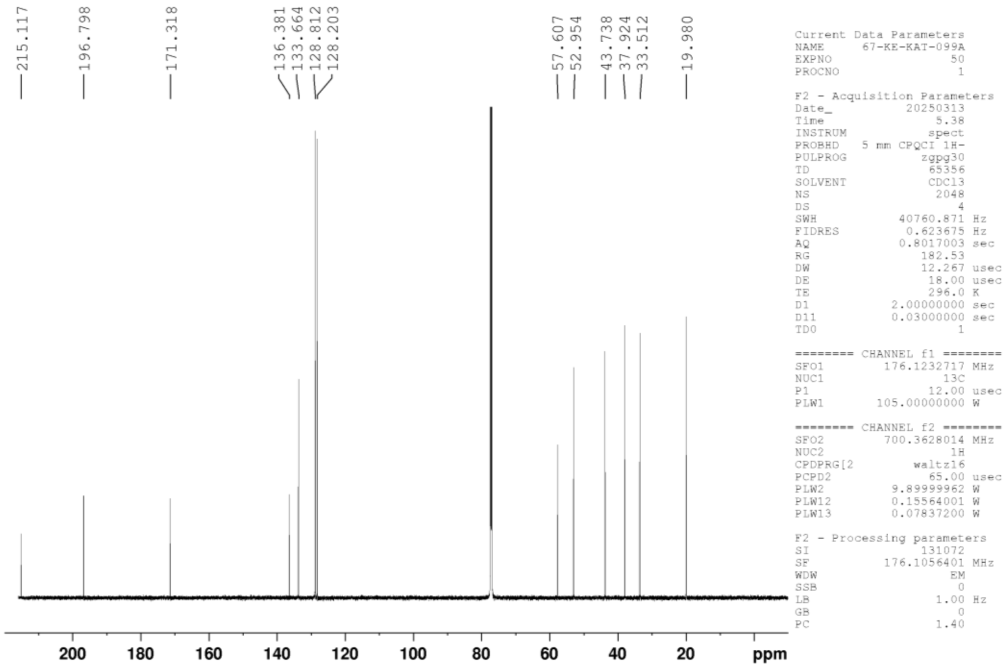
215.117
196.798
171.318
136.381
133.664
128.812
128.203
57.607
52.954
43.738
37.924
33.512
19.980
Current Data Parameters
NAME 67-KE-KAT-099A
EXPNO 50
PROCNO 1
F2 - Acquisition Parameters
Date_ 20250313
Time 5.38
INSTRUM spect
PROBHD 5 mm CPQCI 1H-
PULPROG zgpg30
TD 65356
SOLVENT CDCl3
NS 2048
DS 4
SWH 40760.871 Hz
FIDRES 0.623675 Hz
AQ 0.8017003 sec
RG 182.53
DW 12.267 usec
DE 18.00 usec
TE 296.0 K
D1 2.00000000 sec
D11 0.03000000 sec
TD0 1
======== CHANNEL f1 ========
SFO1 176.1232717 MHz
NUC1 13C
P1 12.00 usec
PLW1 105.00000000 W
======== CHANNEL f2 ========
SFO2 700.3628014 MHz
NUC2 1H
CPDPRG[2 waltz16
PCPD2 65.00 usec
PLW2 9.89999962 W
PLW12 0.15564001 W
PLW13 0.07837200 W
F2 - Processing parameters
SI 131072
SF 176.1056401 MHz
WDW EM
SSB 0
LB 1.00 Hz
GB 0
PC 1.40
200
180
160
140
120
100
80
60
40
20
ppm

3aS

1H

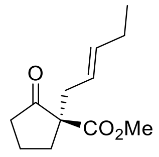
O
CO2Me

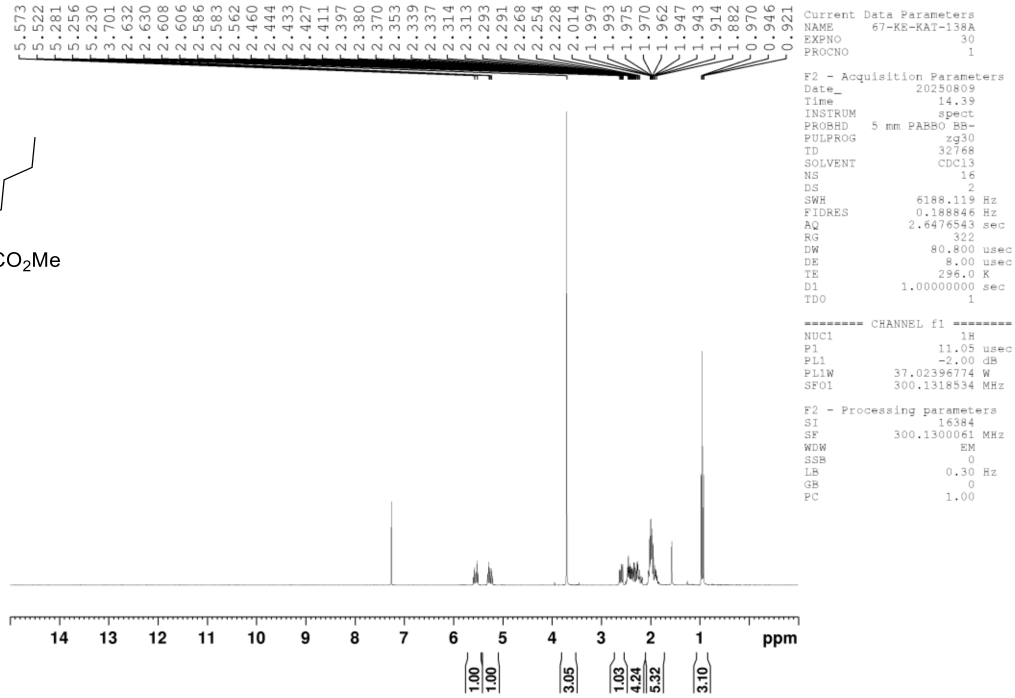
5.573
5.522
5.281
5.256
5.230
3.701
2.632
2.630
2.608
2.606
2.586
2.583
2.562
2.460
2.444
2.433
2.427
2.411
2.397
2.380
2.370
2.353
2.339
2.337
2.314
2.313
2.293
2.291
2.268
2.254
2.228
2.014
1.997
1.993
1.975
1.970
1.962
1.947
1.943
1.914
1.882
0.970
0.946
0.921
Current Data Parameters
NAME 67-KE-KAT-138A
EXPNO 30
PROCNO 1
F2 - Acquisition Parameters
Date_ 20250809
Time 14.39
INSTRUM spect
PROBHD 5 mm PABBO BB-
PULPROG zg30
TD 32768
SOLVENT CDCl3
NS 16
DS 2
SWH 6188.119 Hz
FIDRES 0.188846 Hz
AQ 2.6476543 sec
RG 322
DW 80.800 usec
DE 8.00 usec
TE 296.0 K
D1 1.00000000 sec
TD0 1
======== CHANNEL f1 ========
NUC1 1H
P1 11.05 usec
PL1 -2.00 dB
PL1W 37.02396774 W
SFO1 300.1318534 MHz
F2 - Processing parameters
SI 16384
SF 300.1300061 MHz
WDW EM
SSB 0
LB 0.30 Hz
GB 0
PC 1.00
14
13
12
11
10
9
8
7
6
5
4
3
2
1
ppm
1.00
1.00
3.05
1.03
4.24
5.32
3.10

[13]C

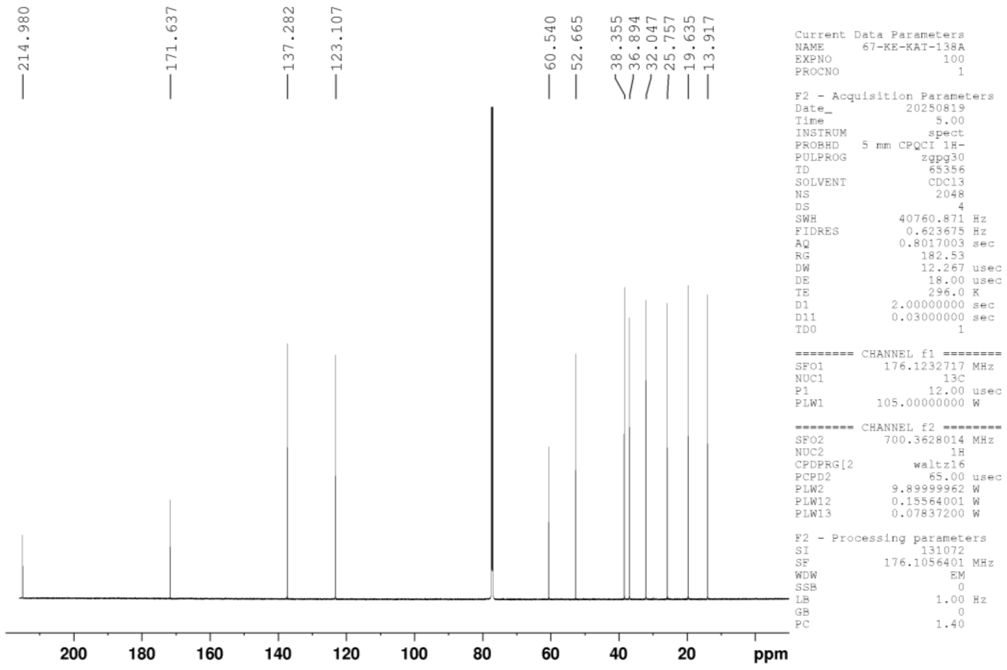
214.980
171.637
137.282
123.107
60.540
52.665
38.355
36.894
32.047
25.757
19.635
13.917
Current Data Parameters
NAME 67-KE-KAT-138A
EXPNO 100
PROCNO 1
F2 - Acquisition Parameters
Date_ 20250819
Time 5.00
INSTRUM spect
PROBHD 5 mm CPQCI 1H-
PULPROG zgpg30
TD 65356
SOLVENT CDCl3
NS 2048
DS 4
SWH 40760.871 Hz
FIDRES 0.623675 Hz
AQ 0.8017003 sec
RG 182.53
DW 12.267 usec
DE 18.00 usec
TE 296.0 K
D1 2.00000000 sec
D11 0.03000000 sec
TD0 1
======== CHANNEL f1 ========
SFO1 176.1232717 MHz
NUC1 13C
P1 12.00 usec
PLW1 105.00000000 W
======== CHANNEL f2 ========
SFO2 700.3628014 MHz
NUC2 1H
CPDPRG[2 waltz16
PCPD2 65.00 usec
PLW2 9.89999962 W
PLW12 0.15564001 W
PLW13 0.07837200 W
F2 - Processing parameters
SI 131072
SF 176.1056401 MHz
WDW EM
SSB 0
LB 1.00 Hz
GB 0
PC 1.40
200 180 160 140 120 100 80 60 40 20 ppm

3aW

[1]H

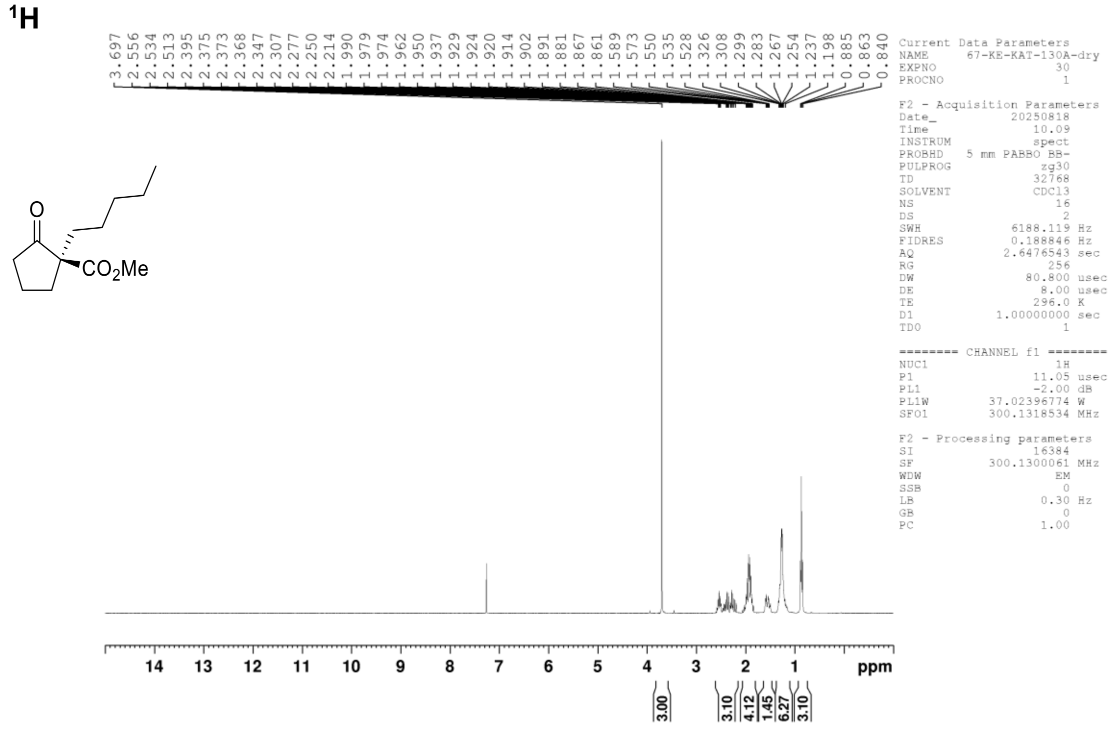
3.697
2.556
2.534
2.513
2.395
2.375
2.373
2.368
2.347
2.307
2.277
2.250
2.214
1.990
1.979
1.974
1.962
1.950
1.937
1.929
1.924
1.920
1.914
1.902
1.891
1.881
1.867
1.861
1.589
1.573
1.550
1.535
1.528
1.326
1.308
1.299
1.283
1.267
1.254
1.237
1.198
0.885
0.863
0.840
O
CO2Me
Current Data Parameters
NAME 67-KE-KAT-130A-dry
EXPNO 30
PROCNO 1
F2 - Acquisition Parameters
Date_ 20250818
Time 10.09
INSTRUM spect
PROBHD 5 mm PABBO BB-
PULPROG zg30
TD 32768
SOLVENT CDCl3
NS 16
DS 2
SWH 6188.119 Hz
FIDRES 0.188846 Hz
AQ 2.6476543 sec
RG 256
DW 80.800 usec
DE 8.00 usec
TE 296.0 K
D1 1.00000000 sec
TD0 1
======== CHANNEL f1 ========
NUC1 1H
P1 11.05 usec
PL1 -2.00 dB
PL1W 37.02396774 W
SFO1 300.1318534 MHz
F2 - Processing parameters
SI 16384
SF 300.1300061 MHz
WDW EM
SSB 0
LB 0.30 Hz
GB 0
PC 1.00
14 13 12 11 10 9 8 7 6 5 4 3 2 1 ppm
3.00
3.10
4.12
1.45
6.27
3.10

13C

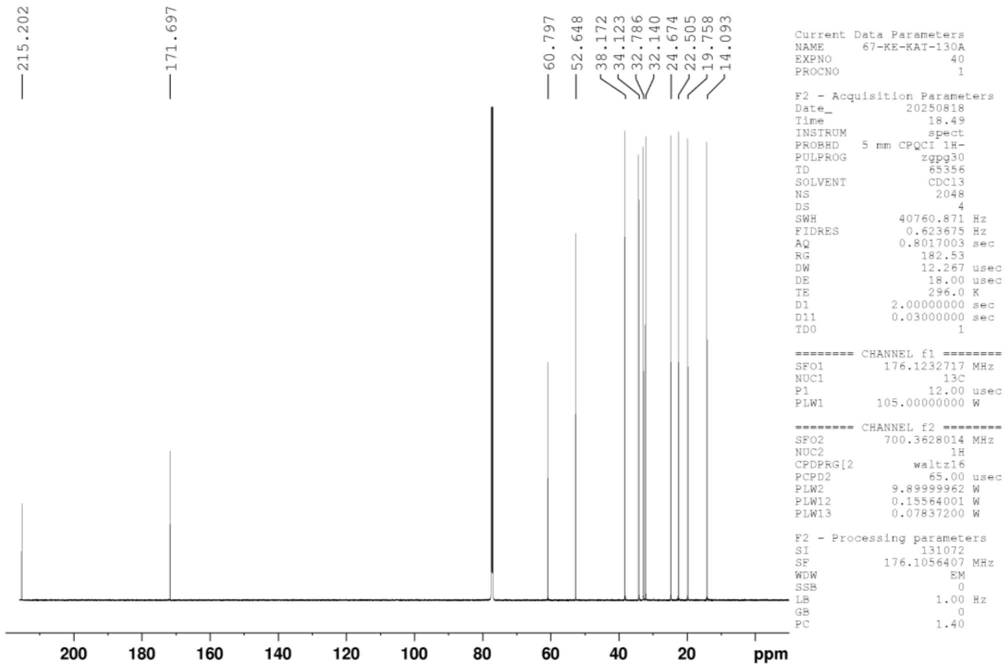

215.202
171.697
60.797
52.648
38.172
34.123
32.786
32.140
24.674
22.505
19.758
14.093
200 180 160 140 120 100 80 60 40 20 ppm
Current Data Parameters
NAME 67-KE-KAT-130A
EXPNO 40
PROCNO 1
F2 - Acquisition Parameters
Date_ 20250818
Time 18.49
INSTRUM spect
PROBHD 5 mm CPQCI 1H-
PULPROG zgpg30
TD 65356
SOLVENT CDCl3
NS 2048
DS 4
SWH 40760.871 Hz
FIDRES 0.623675 Hz
AQ 0.8017003 sec
RG 182.53
DW 12.267 usec
DE 18.00 usec
TE 296.0 K
D1 2.00000000 sec
D11 0.03000000 sec
TD0 1
======== CHANNEL f1 ========
SFO1 176.1232717 MHz
NUC1 13C
P1 12.00 usec
PLW1 105.00000000 W
======== CHANNEL f2 ========
SFO2 700.3628014 MHz
NUC2 1H
CPDPRG[2 waltz16
PCPD2 65.00 usec
PLW2 9.89999962 W
PLW12 0.15564001 W
PLW13 0.07837200 W
F2 - Processing parameters
SI 131072
SF 176.1056407 MHz
WDW EM
SSB 0
LB 1.00 Hz
GB 0
PC 1.40

3aY

1H

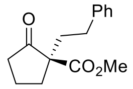

O
Ph
CO2Me

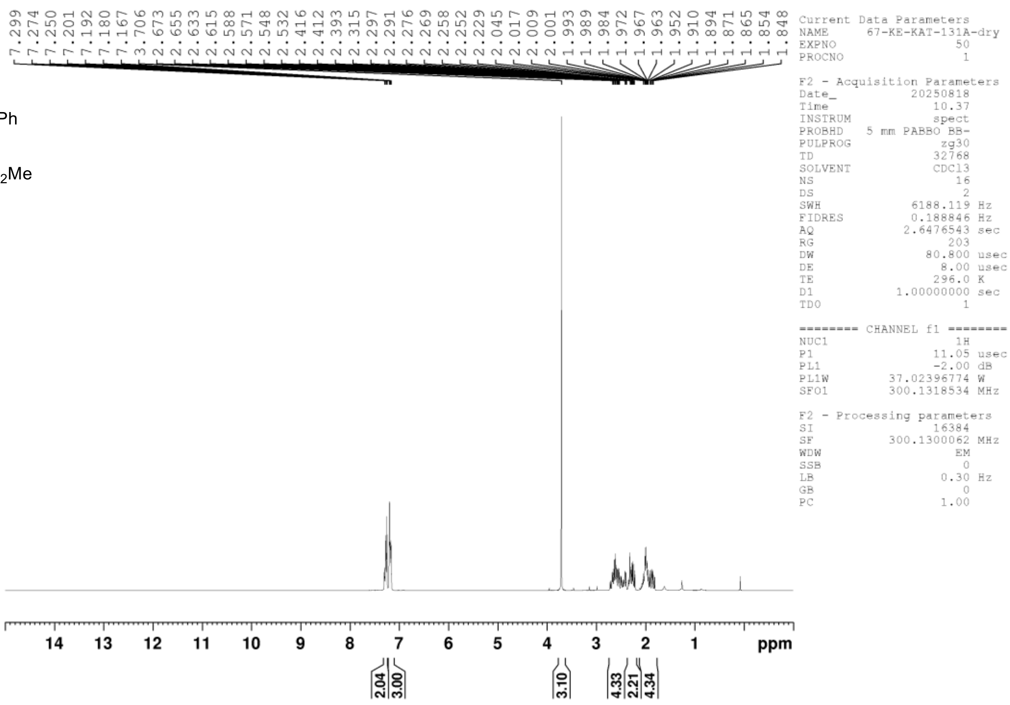

7.299
7.274
7.250
7.201
7.192
7.180
7.167
3.706
2.673
2.655
2.633
2.615
2.588
2.571
2.548
2.532
2.416
2.412
2.393
2.315
2.297
2.291
2.276
2.269
2.258
2.252
2.229
2.045
2.017
2.009
2.001
1.993
1.989
1.984
1.972
1.967
1.963
1.952
1.910
1.894
1.871
1.865
1.854
1.848
14 13 12 11 10 9 8 7 6 5 4 3 2 1 ppm
2.04
3.00
3.10
4.33
2.21
4.34
Current Data Parameters
NAME 67-KE-KAT-131A-dry
EXPNO 50
PROCNO 1
F2 - Acquisition Parameters
Date_ 20250818
Time 10.37
INSTRUM spect
PROBHD 5 mm PABBO BB-
PULPROG zg30
TD 32768
SOLVENT CDCl3
NS 16
DS 2
SWH 6188.119 Hz
FIDRES 0.188846 Hz
AQ 2.6476543 sec
RG 203
DW 80.800 usec
DE 8.00 usec
TE 296.0 K
D1 1.00000000 sec
TD0 1
======== CHANNEL f1 ========
NUC1 1H
P1 11.05 usec
PL1 -2.00 dB
PL1W 37.02396774 W
SFO1 300.1318534 MHz
F2 - Processing parameters
SI 16384
SF 300.1300062 MHz
WDW EM
SSB 0
LB 0.30 Hz
GB 0
PC 1.00

^{13}C

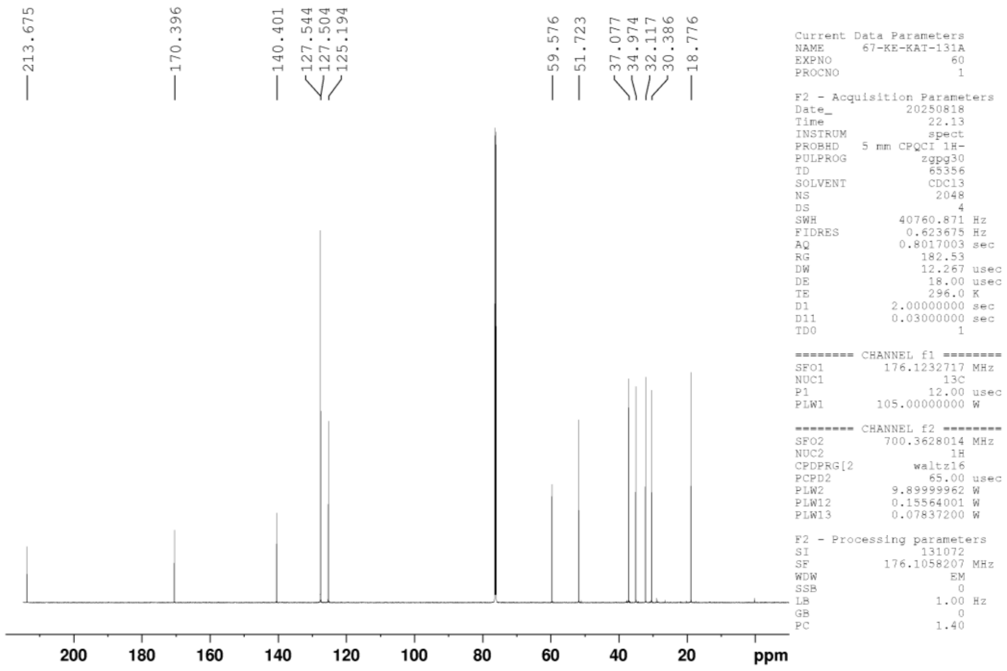
213.675
170.396
140.401
127.544
127.504
125.194
59.576
51.723
37.077
34.974
32.117
30.386
18.776
Current Data Parameters
NAME 67-KE-KAT-131A
EXPNO 60
PROCNO 1
F2 - Acquisition Parameters
Date_ 20250818
Time 22.13
INSTRUM spect
PROBHD 5 mm CPQCI 1H-
PULPROG zgpg30
TD 65356
SOLVENT CDCl3
NS 2048
DS 4
SWH 40760.871 Hz
FIDRES 0.623675 Hz
AQ 0.8017003 sec
RG 182.53
DW 12.267 usec
DE 18.00 usec
TE 296.0 K
D1 2.00000000 sec
D11 0.03000000 sec
TD0 1
======== CHANNEL f1 ========
SFO1 176.1232717 MHz
NUC1 13C
P1 12.00 usec
PLW1 105.00000000 W
======== CHANNEL f2 ========
SFO2 700.3628014 MHz
NUC2 1H
CPDPRG[2 waltz16
PCPD2 65.00 usec
PLW2 9.89999962 W
PLW12 0.15564001 W
PLW13 0.07837200 W
F2 - Processing parameters
SI 131072
SF 176.1058207 MHz
WDW EM
SSB 0
LB 1.00 Hz
GB 0
PC 1.40
200
180
160
140
120
100
80
60
40
20
ppm

3aZ

^{1}H

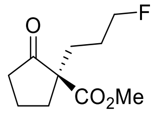
O
F
CO2Me

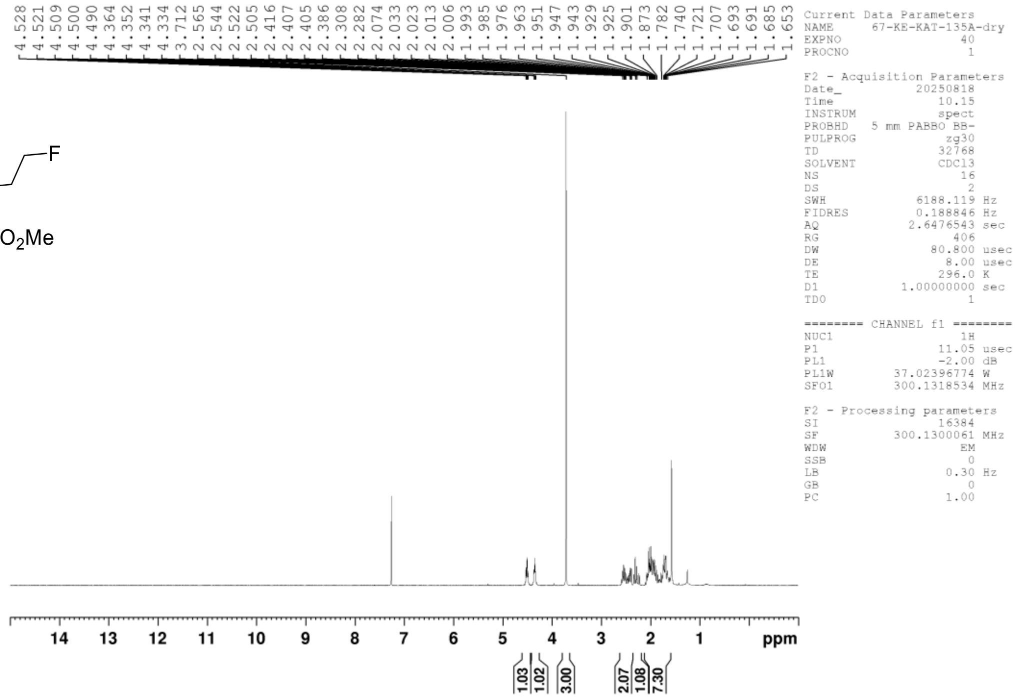
4.528
4.521
4.509
4.500
4.490
4.364
4.352
4.341
4.334
3.712
2.565
2.544
2.522
2.505
2.416
2.407
2.405
2.386
2.308
2.282
2.074
2.033
2.023
2.013
2.006
1.993
1.985
1.976
1.963
1.951
1.947
1.943
1.929
1.925
1.901
1.873
1.782
1.740
1.721
1.707
1.693
1.691
1.685
1.653
Current Data Parameters
NAME 67-KE-KAT-135A-dry
EXPNO 40
PROCNO 1
F2 - Acquisition Parameters
Date_ 20250818
Time 10.15
INSTRUM spect
PROBHD 5 mm PABBO BB-
PULPROG zg30
TD 32768
SOLVENT CDCl3
NS 16
DS 2
SWH 6188.119 Hz
FIDRES 0.188846 Hz
AQ 2.6476543 sec
RG 406
DW 80.800 usec
DE 8.00 usec
TE 296.0 K
D1 1.00000000 sec
TD0 1
======== CHANNEL f1 ========
NUC1 1H
P1 11.05 usec
PL1 -2.00 dB
PL1W 37.02396774 W
SFO1 300.1318534 MHz
F2 - Processing parameters
SI 16384
SF 300.1300061 MHz
WDW EM
SSB 0
LB 0.30 Hz
GB 0
PC 1.00
14
13
12
11
10
9
8
7
6
5
4
3
2
1
ppm
1.03
1.02
3.00
2.07
1.08
7.30

[13]C

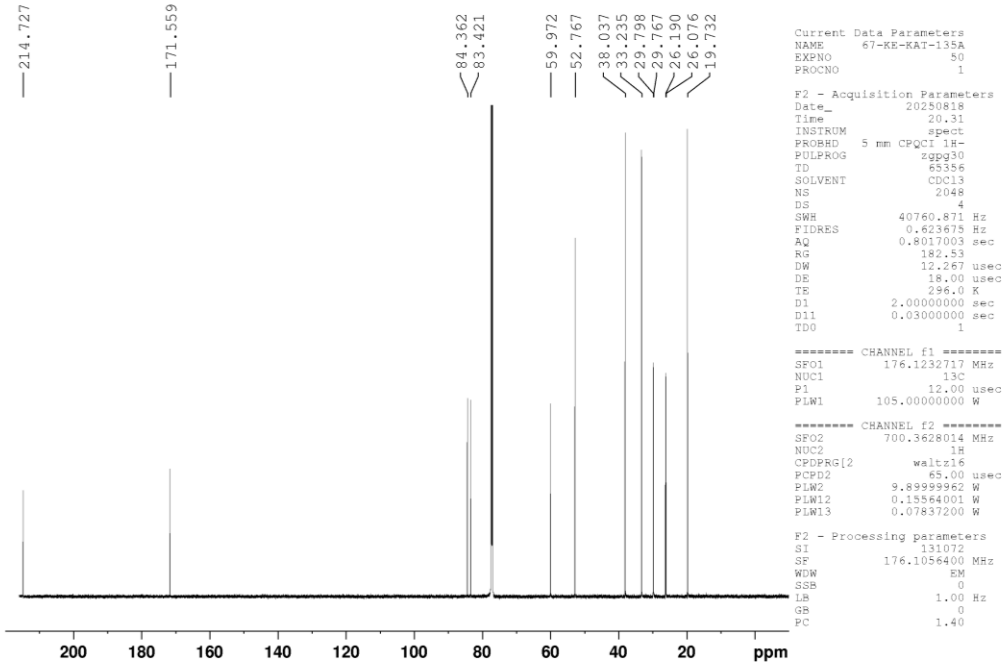
214.727
171.559
84.362
83.421
59.972
52.767
38.037
33.235
29.798
29.767
26.190
26.076
19.732
Current Data Parameters
NAME 67-KE-KAT-135A
EXPNO 50
PROCNO 1
F2 - Acquisition Parameters
Date_ 20250818
Time 20.31
INSTRUM spect
PROBHD 5 mm CPQCI 1H-
PULPROG zgpg30
TD 65356
SOLVENT CDCl3
NS 2048
DS 4
SWH 40760.871 Hz
FIDRES 0.623675 Hz
AQ 0.8017003 sec
RG 182.53
DW 12.267 usec
DE 18.00 usec
TE 296.0 K
D1 2.00000000 sec
D11 0.03000000 sec
TD0 1
======== CHANNEL f1 ========
SFO1 176.1232717 MHz
NUC1 13C
P1 12.00 usec
PLW1 105.00000000 W
======== CHANNEL f2 ========
SFO2 700.3628014 MHz
NUC2 1H
CPDPRG[2 waltz16
PCPD2 65.00 usec
PLW2 9.89999962 W
PLW12 0.15564001 W
PLW13 0.07837200 W
F2 - Processing parameters
SI 131072
SF 176.1056400 MHz
WDW EM
SSB 0
LB 1.00 Hz
GB 0
PC 1.40
200
180
160
140
120
100
80
60
40
20
ppm

3aAA

[1]H

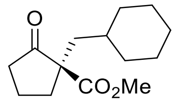
O
CO2Me

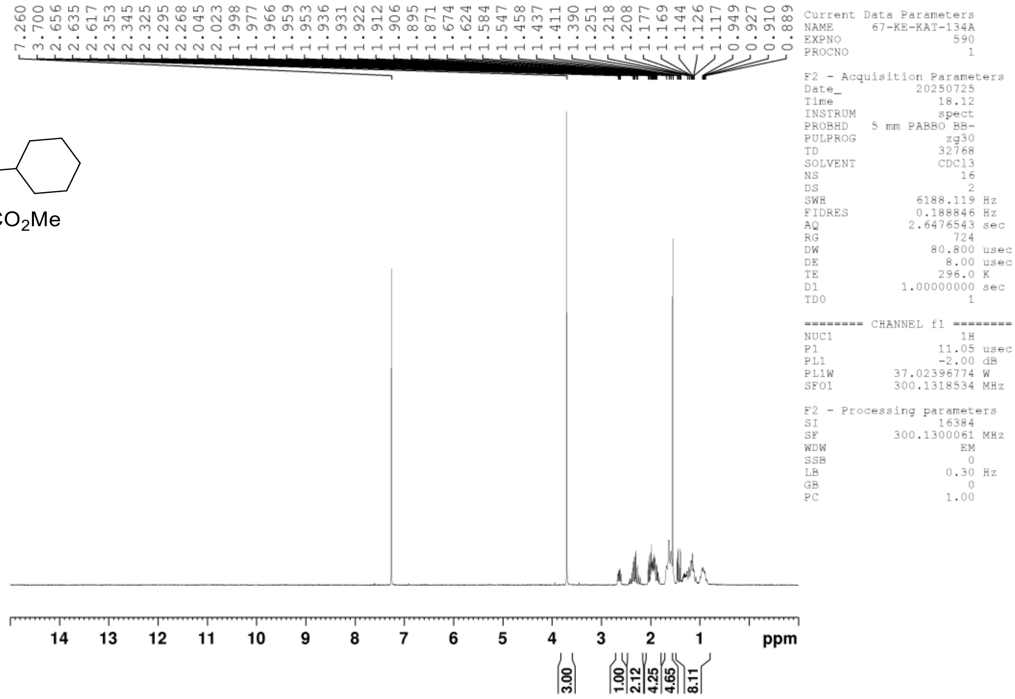
7.260
3.700
2.656
2.635
2.617
2.353
2.345
2.325
2.295
2.268
2.045
2.023
1.998
1.977
1.966
1.959
1.953
1.936
1.931
1.922
1.912
1.906
1.895
1.871
1.674
1.624
1.584
1.547
1.458
1.437
1.411
1.390
1.251
1.218
1.208
1.177
1.169
1.144
1.126
1.117
0.949
0.927
0.910
0.889
Current Data Parameters
NAME 67-KE-KAT-134A
EXPNO 590
PROCNO 1
F2 - Acquisition Parameters
Date_ 20250725
Time 18.12
INSTRUM spect
PROBHD 5 mm PABBO BB-
PULPROG zg30
TD 32768
SOLVENT CDCl3
NS 16
DS 2
SWH 6188.119 Hz
FIDRES 0.188846 Hz
AQ 2.6476543 sec
RG 724
DW 80.800 usec
DE 8.00 usec
TE 296.0 K
D1 1.00000000 sec
TD0 1
======== CHANNEL f1 ========
NUC1 1H
P1 11.05 usec
PL1 -2.00 dB
PL1W 37.02396774 W
SFO1 300.1318534 MHz
F2 - Processing parameters
SI 16384
SF 300.1300061 MHz
WDW EM
SSB 0
LB 0.30 Hz
GB 0
PC 1.00
14
13
12
11
10
9
8
7
6
5
4
3
2
1
ppm
3.00
1.00
2.12
4.25
4.65
8.11

[13]C

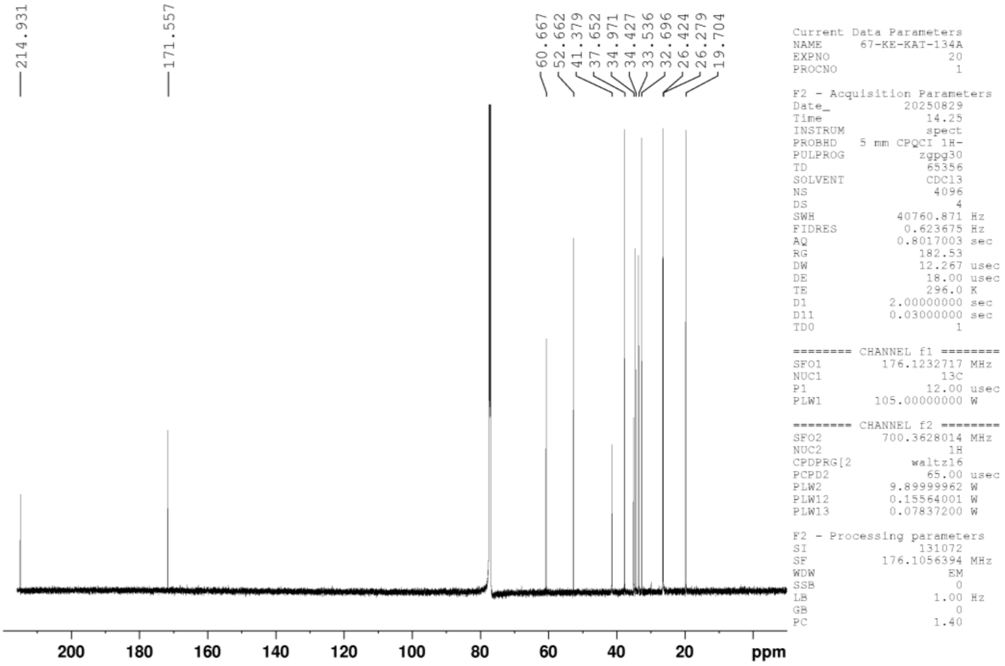

214.931
171.557
60.667
52.662
41.379
37.652
34.971
34.427
33.536
32.696
26.424
26.279
19.704
Current Data Parameters
NAME 67-KE-KAT-134A
EXPNO 20
PROCNO 1
F2 - Acquisition Parameters
Date_ 20250829
Time 14.25
INSTRUM spect
PROBHD 5 mm CPQCI 1H-
PULPROG zgpg30
TD 65356
SOLVENT CDCl3
NS 4096
DS 4
SWH 40760.871 Hz
FIDRES 0.623675 Hz
AQ 0.8017003 sec
RG 182.53
DW 12.267 usec
DE 18.00 usec
TE 296.0 K
D1 2.00000000 sec
D11 0.03000000 sec
TD0 1
======== CHANNEL f1 ========
SFO1 176.1232717 MHz
NUC1 13C
P1 12.00 usec
PLW1 105.00000000 W
======== CHANNEL f2 ========
SFO2 700.3628014 MHz
NUC2 1H
CPDPRG[2 waltz16
PCPD2 65.00 usec
PLW2 9.89999962 W
PLW12 0.15564001 W
PLW13 0.07837200 W
F2 - Processing parameters
SI 131072
SF 176.1056394 MHz
WDW EM
SSB 0
LB 1.00 Hz
GB 0
PC 1.40
200 180 160 140 120 100 80 60 40 20 ppm

3dA

[1]H

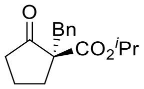

O
Bn
$CO_2{}^iPr$

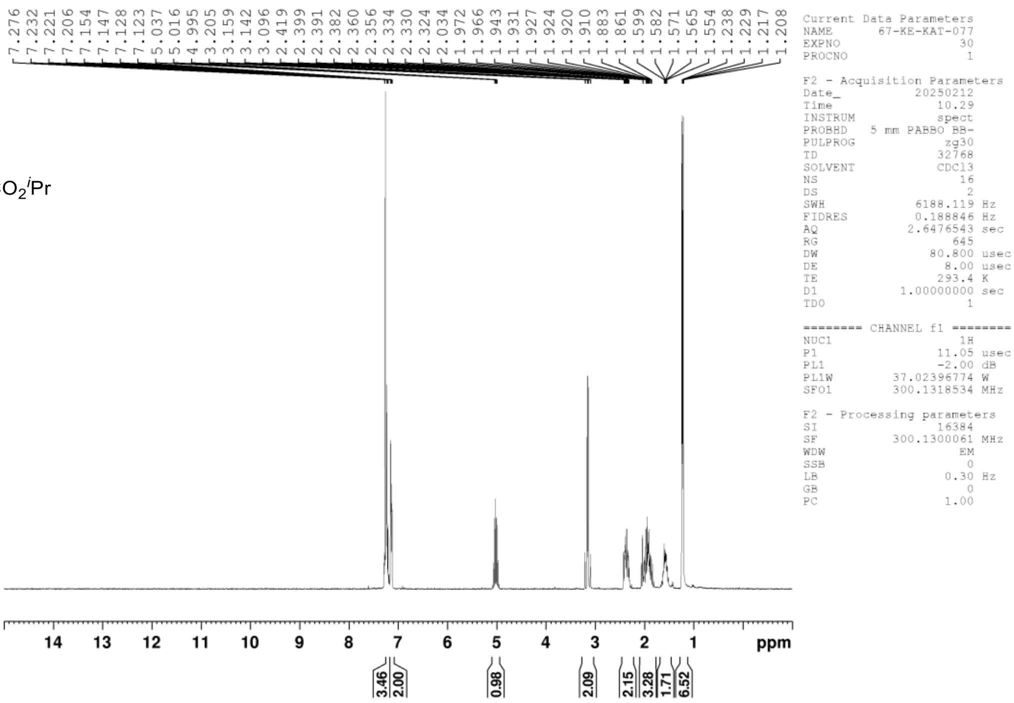

7.276
7.232
7.221
7.206
7.154
7.147
7.128
7.123
5.037
5.016
4.995
3.205
3.159
3.142
3.096
2.419
2.399
2.391
2.382
2.360
2.356
2.334
2.330
2.324
2.034
1.972
1.966
1.943
1.931
1.927
1.924
1.920
1.910
1.861
1.599
1.582
1.571
1.565
1.554
1.238
1.229
1.217
1.208
Current Data Parameters
NAME 67-KE-KAT-077
EXPNO 30
PROCNO 1
F2 - Acquisition Parameters
Date_ 20250212
Time 10.29
INSTRUM spect
PROBHD 5 mm PABBO BB-
PULPROG zg30
TD 32768
SOLVENT CDCl3
NS 16
DS 2
SWH 6188.119 Hz
FIDRES 0.188846 Hz
AQ 2.6476543 sec
RG 645
DW 80.800 usec
DE 8.00 usec
TE 293.4 K
D1 1.00000000 sec
TD0 1
======== CHANNEL f1 ========
NUC1 1H
P1 11.05 usec
PL1 -2.00 dB
PL1W 37.02396774 W
SFO1 300.1318534 MHz
F2 - Processing parameters
SI 16384
SF 300.1300061 MHz
WDW EM
SSB 0
LB 0.30 Hz
GB 0
PC 1.00
14 13 12 11 10 9 8 7 6 5 4 3 2 1 ppm
3.46
2.00
0.98
2.09
2.15
3.28
1.71
6.52

^{13}C

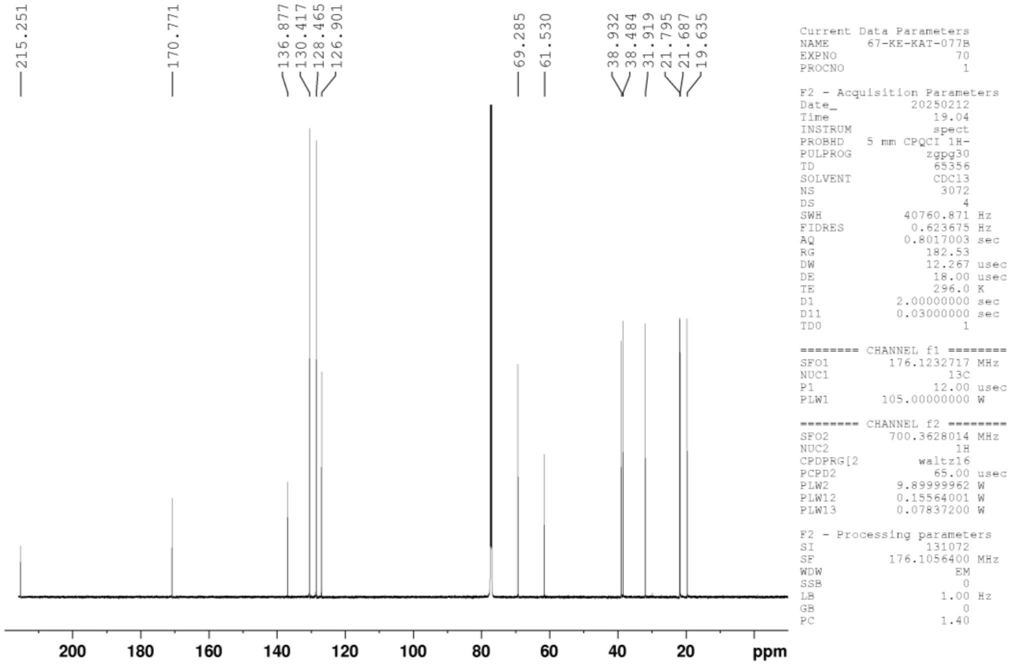

215.251
170.771
136.877
130.417
128.465
126.901
69.285
61.530
38.932
38.484
31.919
21.795
21.687
19.635
Current Data Parameters
NAME 67-KE-KAT-077B
EXPNO 70
PROCNO 1
F2 - Acquisition Parameters
Date_ 20250212
Time 19.04
INSTRUM spect
PROBHD 5 mm CPQCI 1H-
PULPROG zgpg30
TD 65356
SOLVENT CDCl3
NS 3072
DS 4
SWH 40760.871 Hz
FIDRES 0.623675 Hz
AQ 0.8017003 sec
RG 182.53
DW 12.267 usec
DE 18.00 usec
TE 296.0 K
D1 2.00000000 sec
D11 0.03000000 sec
TD0 1
======== CHANNEL f1 ========
SFO1 176.1232717 MHz
NUC1 13C
P1 12.00 usec
PLW1 105.00000000 W
======== CHANNEL f2 ========
SFO2 700.3628014 MHz
NUC2 1H
CPDPRG[2 waltz16
PCPD2 65.00 usec
PLW2 9.89999962 W
PLW12 0.15564001 W
PLW13 0.07837200 W
F2 - Processing parameters
SI 131072
SF 176.1056400 MHz
WDW EM
SSB 0
LB 1.00 Hz
GB 0
PC 1.40
200 180 160 140 120 100 80 60 40 20 ppm

3eA

^{1}H

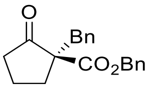

Bn
CO2Bn

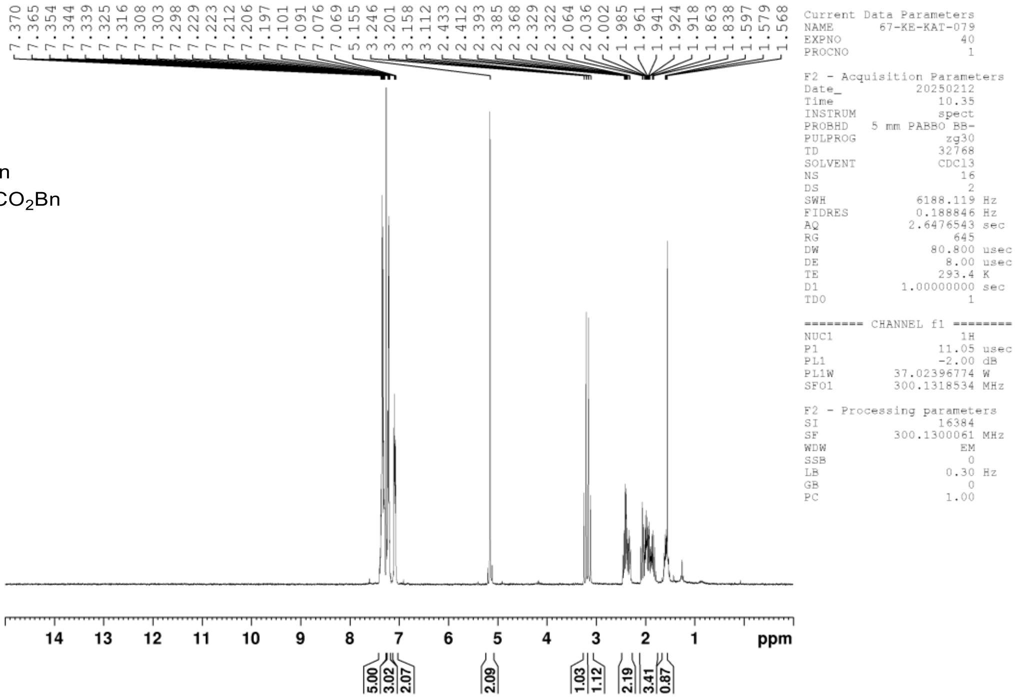

7.370
7.365
7.354
7.344
7.339
7.325
7.316
7.308
7.303
7.298
7.229
7.223
7.212
7.206
7.197
7.101
7.091
7.076
7.069
5.155
3.246
3.201
3.158
3.112
2.433
2.412
2.393
2.385
2.368
2.329
2.322
2.064
2.036
2.002
1.985
1.961
1.941
1.924
1.918
1.863
1.838
1.597
1.579
1.568
Current Data Parameters
NAME 67-KE-KAT-079
EXPNO 40
PROCNO 1
F2 - Acquisition Parameters
Date_ 20250212
Time 10.35
INSTRUM spect
PROBHD 5 mm PABBO BB-
PULPROG zg30
TD 32768
SOLVENT CDCl3
NS 16
DS 2
SWH 6188.119 Hz
FIDRES 0.188846 Hz
AQ 2.6476543 sec
RG 645
DW 80.800 usec
DE 8.00 usec
TE 293.4 K
D1 1.00000000 sec
TD0 1
======== CHANNEL f1 ========
NUC1 1H
P1 11.05 usec
PL1 -2.00 dB
PL1W 37.02396774 W
SFO1 300.1318534 MHz
F2 - Processing parameters
SI 16384
SF 300.1300061 MHz
WDW EM
SSB 0
LB 0.30 Hz
GB 0
PC 1.00
14 13 12 11 10 9 8 7 6 5 4 3 2 1 ppm
5.00
3.02
2.07
2.09
1.03
1.12
2.19
3.41
0.87

^{13}C

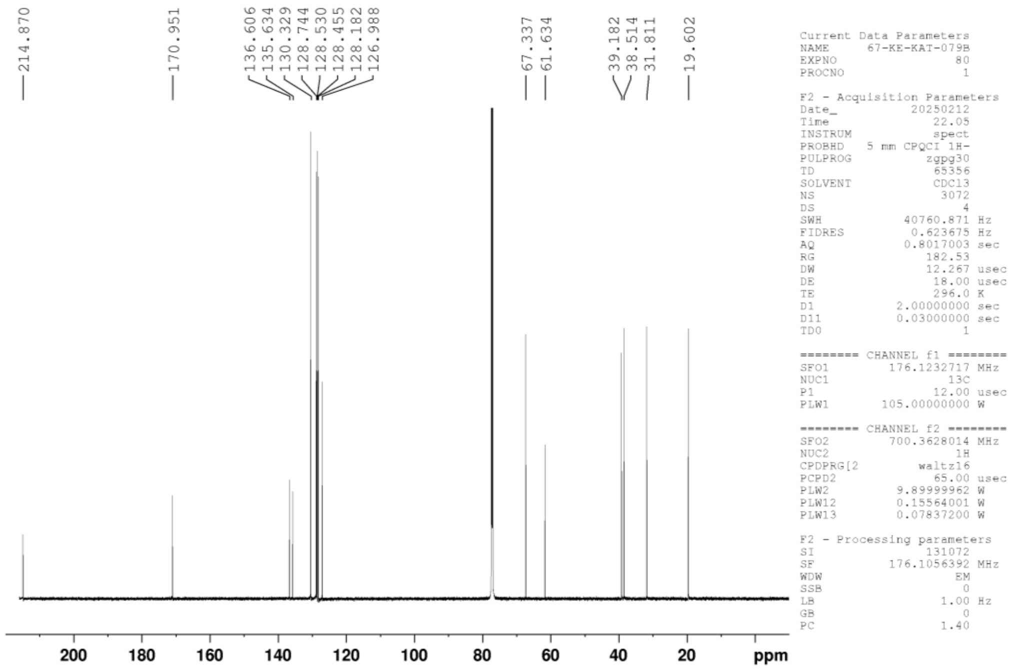
214.870
170.951
136.606
135.634
130.329
128.744
128.530
128.455
128.182
126.988
67.337
61.634
39.182
38.514
31.811
19.602
200 180 160 140 120 100 80 60 40 20 ppm
Current Data Parameters
NAME 67-KE-KAT-079B
EXPNO 80
PROCNO 1
F2 - Acquisition Parameters
Date_ 20250212
Time 22.05
INSTRUM spect
PROBHD 5 mm CPQCI 1H-
PULPROG zgpg30
TD 65356
SOLVENT CDCl3
NS 3072
DS 4
SWH 40760.871 Hz
FIDRES 0.623675 Hz
AQ 0.8017003 sec
RG 182.53
DW 12.267 usec
DE 18.00 usec
TE 296.0 K
D1 2.00000000 sec
D11 0.03000000 sec
TD0 1
======== CHANNEL f1 ========
SFO1 176.1232717 MHz
NUC1 13C
P1 12.00 usec
PLW1 105.00000000 W
======== CHANNEL f2 ========
SFO2 700.3628014 MHz
NUC2 1H
CPDPRG[2 waltz16
PCPD2 65.00 usec
PLW2 9.89999962 W
PLW12 0.15564001 W
PLW13 0.07837200 W
F2 - Processing parameters
SI 131072
SF 176.1056392 MHz
WDW EM
SSB 0
LB 1.00 Hz
GB 0
PC 1.40

3fA

^{1}H

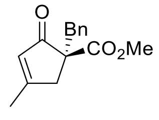
O
Bn
CO2Me

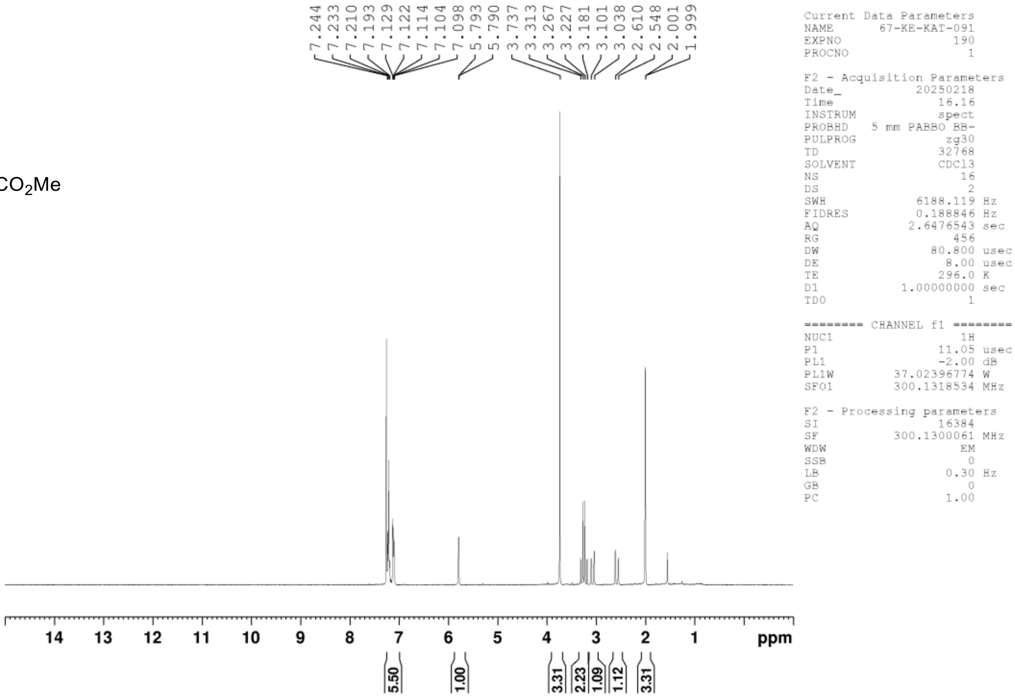
7.244
7.233
7.210
7.193
7.129
7.122
7.114
7.104
7.098
5.793
5.790
3.737
3.313
3.267
3.227
3.181
3.101
3.038
2.610
2.548
2.001
1.999
14 13 12 11 10 9 8 7 6 5 4 3 2 1 ppm
5.50
1.00
3.31
2.23
1.09
1.12
3.31
Current Data Parameters
NAME 67-KE-KAT-091
EXPNO 190
PROCNO 1
F2 - Acquisition Parameters
Date_ 20250218
Time 16.16
INSTRUM spect
PROBHD 5 mm PABBO BB-
PULPROG zg30
TD 32768
SOLVENT CDCl3
NS 16
DS 2
SWH 6188.119 Hz
FIDRES 0.188846 Hz
AQ 2.6476543 sec
RG 456
DW 80.800 usec
DE 8.00 usec
TE 296.0 K
D1 1.00000000 sec
TD0 1
======== CHANNEL f1 ========
NUC1 1H
P1 11.05 usec
PL1 -2.00 dB
PL1W 37.02396774 W
SFO1 300.1318534 MHz
F2 - Processing parameters
SI 16384
SF 300.1300061 MHz
WDW EM
SSB 0
LB 0.30 Hz
GB 0
PC 1.00

^{13}C

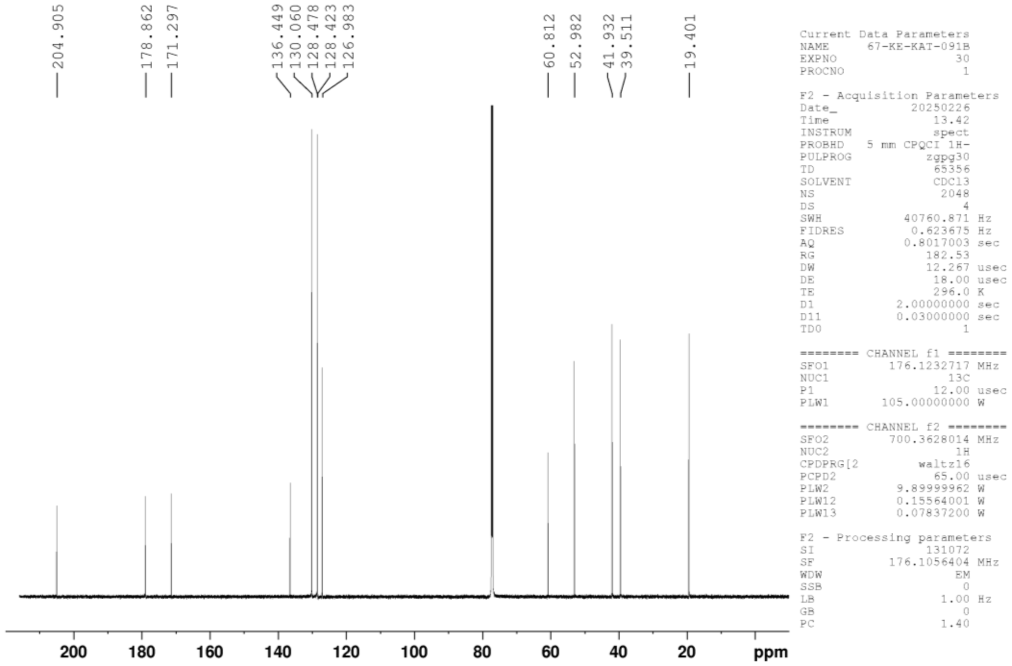
204.905
178.862
171.297
136.449
130.060
128.478
128.423
126.983
60.812
52.982
41.932
39.511
19.401
Current Data Parameters
NAME 67-KE-KAT-091B
EXPNO 30
PROCNO 1
F2 - Acquisition Parameters
Date_ 20250226
Time 13.42
INSTRUM spect
PROBHD 5 mm CPQCI 1H-
PULPROG zgpg30
TD 65356
SOLVENT CDCl3
NS 2048
DS 4
SWH 40760.871 Hz
FIDRES 0.623675 Hz
AQ 0.8017003 sec
RG 182.53
DW 12.267 usec
DE 18.00 usec
TE 296.0 K
D1 2.00000000 sec
D11 0.03000000 sec
TD0 1
======== CHANNEL f1 ========
SFO1 176.1232717 MHz
NUC1 13C
P1 12.00 usec
PLW1 105.00000000 W
======== CHANNEL f2 ========
SFO2 700.3628014 MHz
NUC2 1H
CPDPRG[2 waltz16
PCPD2 65.00 usec
PLW2 9.89999962 W
PLW12 0.15564001 W
PLW13 0.07837200 W
F2 - Processing parameters
SI 131072
SF 176.1056404 MHz
WDW EM
SSB 0
LB 1.00 Hz
GB 0
PC 1.40
200
180
160
140
120
100
80
60
40
20
ppm

3gA

^{1}H

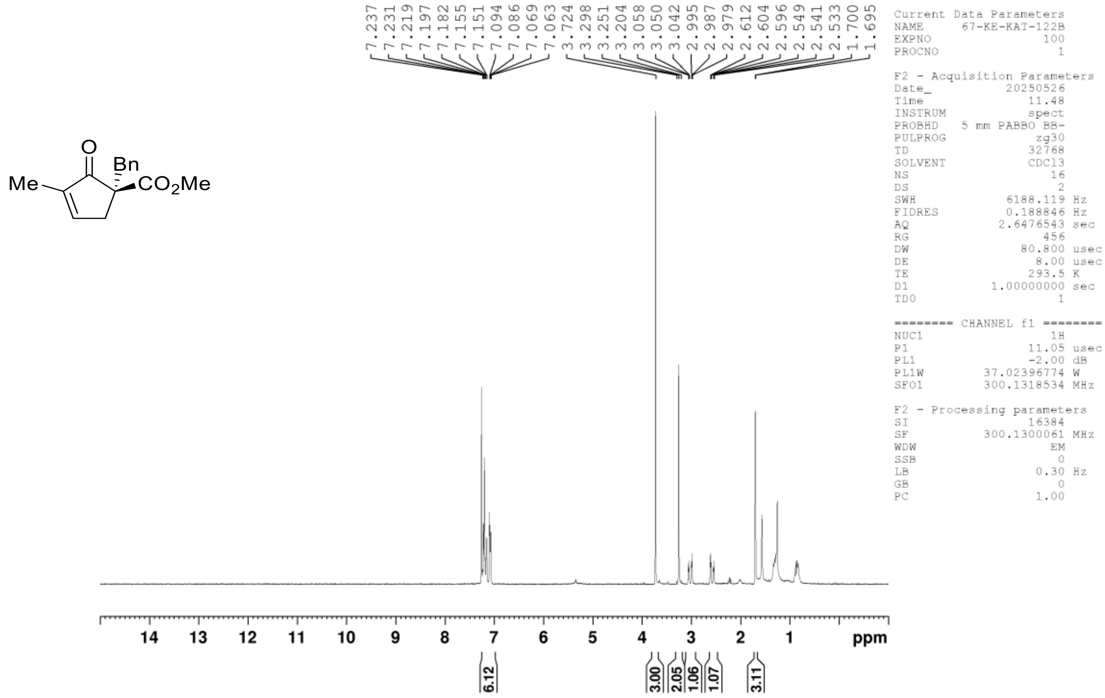
O
Bn
Me
CO2Me
7.237
7.231
7.219
7.197
7.182
7.155
7.151
7.094
7.086
7.069
7.063
3.724
3.298
3.251
3.204
3.058
3.050
3.042
2.995
2.987
2.979
2.612
2.604
2.596
2.549
2.541
2.533
1.700
1.695
Current Data Parameters
NAME 67-KE-KAT-122B
EXPNO 100
PROCNO 1
F2 - Acquisition Parameters
Date_ 20250526
Time 11.48
INSTRUM spect
PROBHD 5 mm PABBO BB-
PULPROG zg30
TD 32768
SOLVENT CDCl3
NS 16
DS 2
SWH 6188.119 Hz
FIDRES 0.188846 Hz
AQ 2.6476543 sec
RG 456
DW 80.800 usec
DE 8.00 usec
TE 293.5 K
D1 1.00000000 sec
TD0 1
======== CHANNEL f1 ========
NUC1 1H
P1 11.05 usec
PL1 -2.00 dB
PL1W 37.02396774 W
SFO1 300.1318534 MHz
F2 - Processing parameters
SI 16384
SF 300.1300061 MHz
WDW EM
SSB 0
LB 0.30 Hz
GB 0
PC 1.00
14
13
12
11
10
9
8
7
6
5
4
3
2
1
ppm
6.12
3.00
2.05
1.06
1.07
3.11

^{13}C

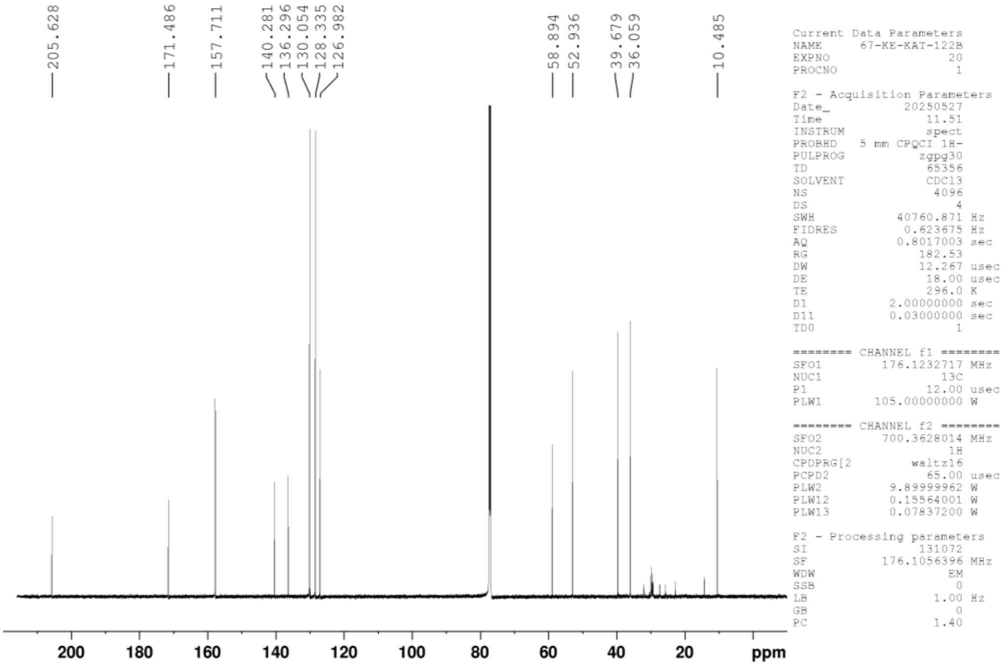
205.628
171.486
157.711
140.281
136.296
130.054
128.335
126.982
58.894
52.936
39.679
36.059
10.485
200 180 160 140 120 100 80 60 40 20 ppm
Current Data Parameters
NAME 67-KE-KAT-122B
EXPNO 20
PROCNO 1
F2 - Acquisition Parameters
Date_ 20250527
Time 11.51
INSTRUM spect
PROBHD 5 mm CPQCI 1H-
PULPROG zgpg30
TD 65356
SOLVENT CDCl3
NS 4096
DS 4
SWH 40760.871 Hz
FIDRES 0.623675 Hz
AQ 0.8017003 sec
RG 182.53
DW 12.267 usec
DE 18.00 usec
TE 296.0 K
D1 2.00000000 sec
D11 0.03000000 sec
TD0 1
======== CHANNEL f1 ========
SFO1 176.1232717 MHz
NUC1 13C
P1 12.00 usec
PLW1 105.00000000 W
======== CHANNEL f2 ========
SFO2 700.3628014 MHz
NUC2 1H
CPDPRG[2 waltz16
PCPD2 65.00 usec
PLW2 9.89999962 W
PLW12 0.15564001 W
PLW13 0.07837200 W
F2 - Processing parameters
SI 131072
SF 176.1056396 MHz
WDW EM
SSB 0
LB 1.00 Hz
GB 0
PC 1.40

3kA

^{1}H

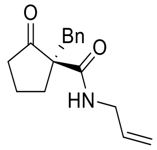
O
Bn
O
HN

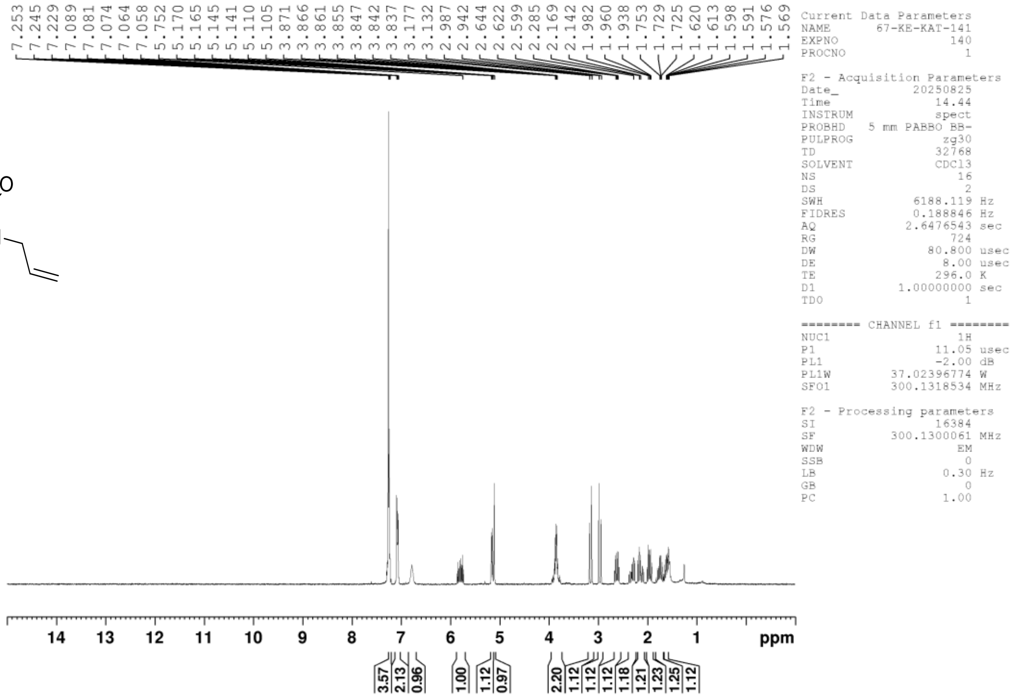
7.253
7.245
7.229
7.089
7.081
7.074
7.064
7.058
5.752
5.170
5.165
5.145
5.141
5.110
5.105
3.871
3.866
3.861
3.855
3.847
3.842
3.837
3.177
3.132
2.987
2.942
2.644
2.622
2.599
2.285
2.169
2.142
1.982
1.960
1.938
1.753
1.729
1.725
1.620
1.613
1.598
1.591
1.576
1.569
14 13 12 11 10 9 8 7 6 5 4 3 2 1 ppm
3.57 2.13 0.96 1.00 1.12 0.97 2.20 1.12 1.12 1.12 1.18 1.21 1.23 1.25 1.12
Current Data Parameters
NAME 67-KE-KAT-141
EXPNO 140
PROCNO 1
F2 - Acquisition Parameters
Date_ 20250825
Time 14.44
INSTRUM spect
PROBHD 5 mm PABBO BB-
PULPROG zg30
TD 32768
SOLVENT CDCl3
NS 16
DS 2
SWH 6188.119 Hz
FIDRES 0.188846 Hz
AQ 2.6476543 sec
RG 724
DW 80.800 usec
DE 8.00 usec
TE 296.0 K
D1 1.00000000 sec
TD0 1
======== CHANNEL f1 ========
NUC1 1H
P1 11.05 usec
PL1 -2.00 dB
PL1W 37.02396774 W
SFO1 300.1318534 MHz
F2 - Processing parameters
SI 16384
SF 300.1300061 MHz
WDW EM
SSB 0
LB 0.30 Hz
GB 0
PC 1.00

[13]C

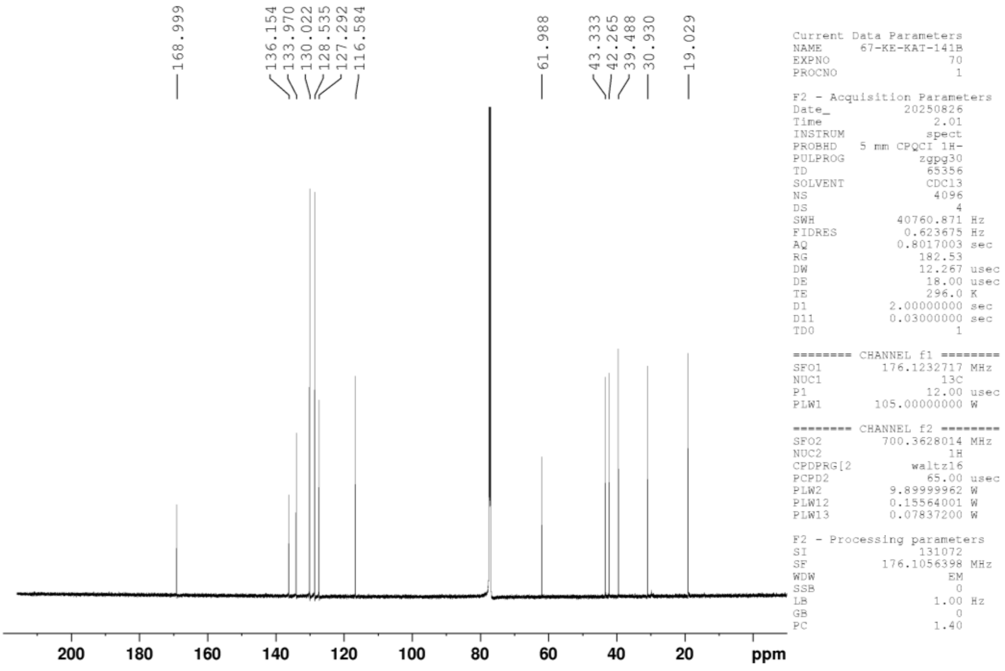
168.999
136.154
133.970
130.022
128.535
127.292
116.584
61.988
43.333
42.265
39.488
30.930
19.029
Current Data Parameters
NAME 67-KE-KAT-141B
EXPNO 70
PROCNO 1
F2 - Acquisition Parameters
Date_ 20250826
Time 2.01
INSTRUM spect
PROBHD 5 mm CPQCI 1H-
PULPROG zgpg30
TD 65356
SOLVENT CDCl3
NS 4096
DS 4
SWH 40760.871 Hz
FIDRES 0.623675 Hz
AQ 0.8017003 sec
RG 182.53
DW 12.267 usec
DE 18.00 usec
TE 296.0 K
D1 2.00000000 sec
D11 0.03000000 sec
TD0 1
======== CHANNEL f1 ========
SFO1 176.1232717 MHz
NUC1 13C
P1 12.00 usec
PLW1 105.00000000 W
======== CHANNEL f2 ========
SFO2 700.3628014 MHz
NUC2 1H
CPDPRG[2 waltz16
PCPD2 65.00 usec
PLW2 9.89999962 W
PLW12 0.15564001 W
PLW13 0.07837200 W
F2 - Processing parameters
SI 131072
SF 176.1056398 MHz
WDW EM
SSB 0
LB 1.00 Hz
GB 0
PC 1.40
200 180 160 140 120 100 80 60 40 20 ppm

3lA

[1]H

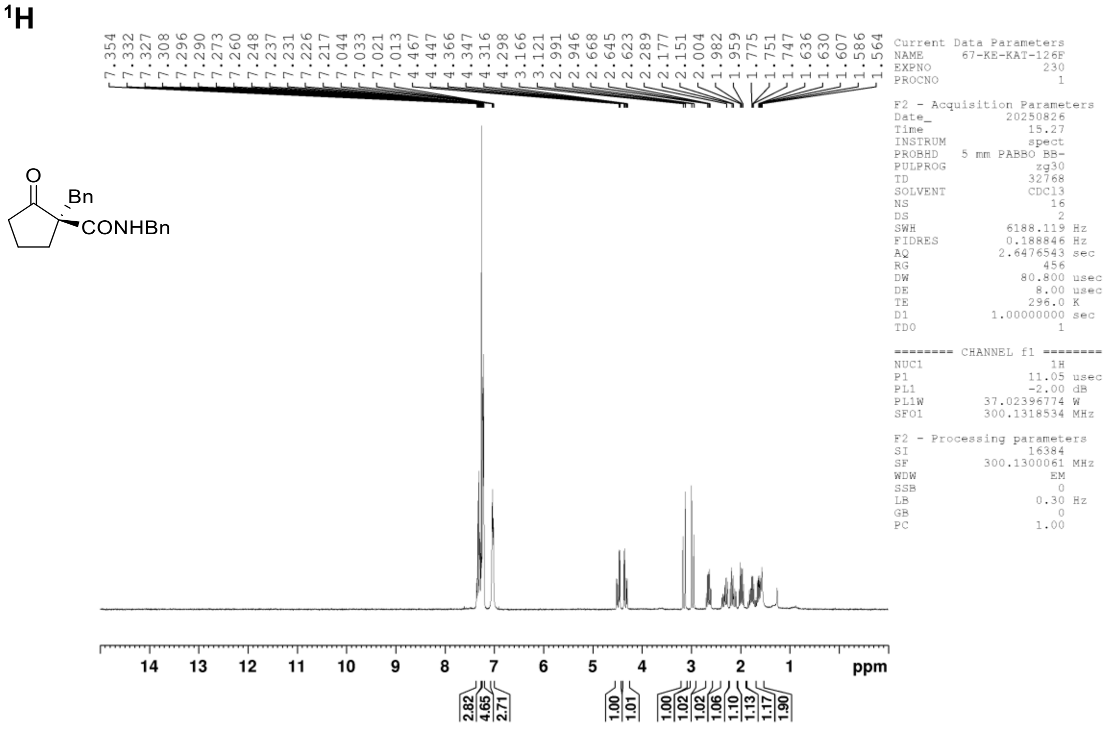
7.354
7.332
7.327
7.308
7.296
7.290
7.273
7.260
7.248
7.237
7.231
7.226
7.217
7.044
7.033
7.021
7.013
4.467
4.447
4.366
4.347
4.316
4.298
3.166
3.121
2.991
2.946
2.668
2.645
2.623
2.289
2.177
2.151
2.004
1.982
1.959
1.775
1.751
1.747
1.636
1.630
1.607
1.586
1.564
O
Bn
CONHBn
Current Data Parameters
NAME 67-KE-KAT-126F
EXPNO 230
PROCNO 1
F2 - Acquisition Parameters
Date_ 20250826
Time 15.27
INSTRUM spect
PROBHD 5 mm PABBO BB-
PULPROG zg30
TD 32768
SOLVENT CDCl3
NS 16
DS 2
SWH 6188.119 Hz
FIDRES 0.188846 Hz
AQ 2.6476543 sec
RG 456
DW 80.800 usec
DE 8.00 usec
TE 296.0 K
D1 1.00000000 sec
TD0 1
======== CHANNEL f1 ========
NUC1 1H
P1 11.05 usec
PL1 -2.00 dB
PL1W 37.02396774 W
SFO1 300.1318534 MHz
F2 - Processing parameters
SI 16384
SF 300.1300061 MHz
WDW EM
SSB 0
LB 0.30 Hz
GB 0
PC 1.00
14 13 12 11 10 9 8 7 6 5 4 3 2 1 ppm
2.82
4.65
2.71
1.00
1.01
1.00
1.02
1.02
1.06
1.10
1.13
1.17
1.90

^{13}C

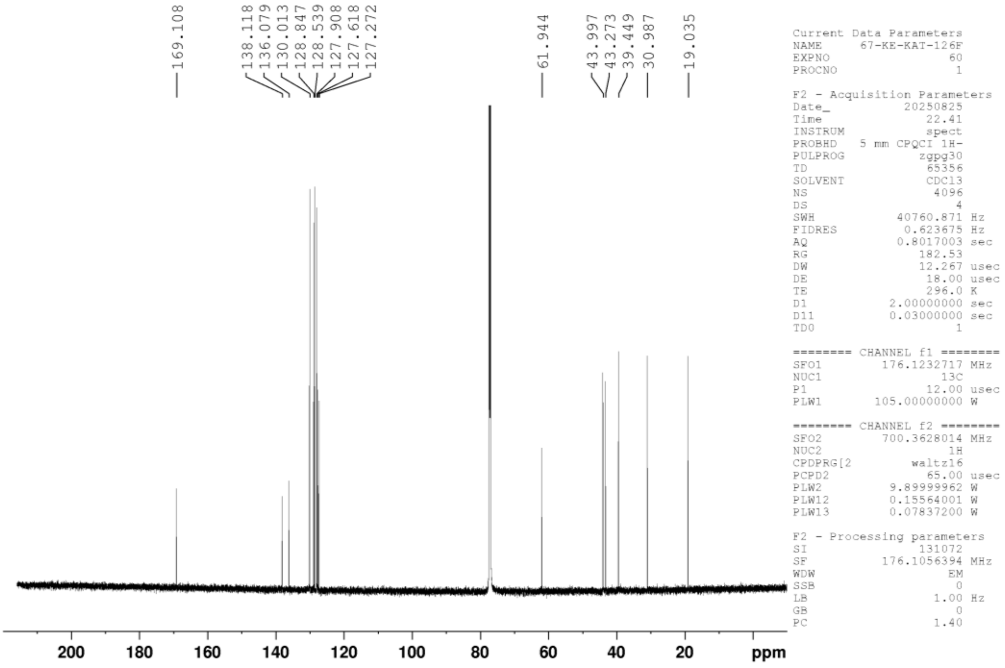
169.108
138.118
136.079
130.013
128.847
128.539
127.908
127.618
127.272
61.944
43.997
43.273
39.449
30.987
19.035
200
180
160
140
120
100
80
60
40
20
ppm
Current Data Parameters
NAME 67-KE-KAT-126F
EXPNO 60
PROCNO 1
F2 - Acquisition Parameters
Date_ 20250825
Time 22.41
INSTRUM spect
PROBHD 5 mm CPQCI 1H-
PULPROG zgpg30
TD 65356
SOLVENT CDCl3
NS 4096
DS 4
SWH 40760.871 Hz
FIDRES 0.623675 Hz
AQ 0.8017003 sec
RG 182.53
DW 12.267 usec
DE 18.00 usec
TE 296.0 K
D1 2.00000000 sec
D11 0.03000000 sec
TD0 1
======== CHANNEL f1 ========
SFO1 176.1232717 MHz
NUC1 13C
P1 12.00 usec
PLW1 105.00000000 W
======== CHANNEL f2 ========
SFO2 700.3628014 MHz
NUC2 1H
CPDPRG[2 waltz16
PCPD2 65.00 usec
PLW2 9.89999962 W
PLW12 0.15564001 W
PLW13 0.07837200 W
F2 - Processing parameters
SI 131072
SF 176.1056394 MHz
WDW EM
SSB 0
LB 1.00 Hz
GB 0
PC 1.40

14 HPLC Data

3aA

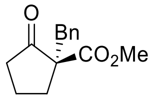

CHIRALPAK® *IA*, *n*-hexane/iPrOH = 97/3, 0.5 mL/min, λ = 215 nm

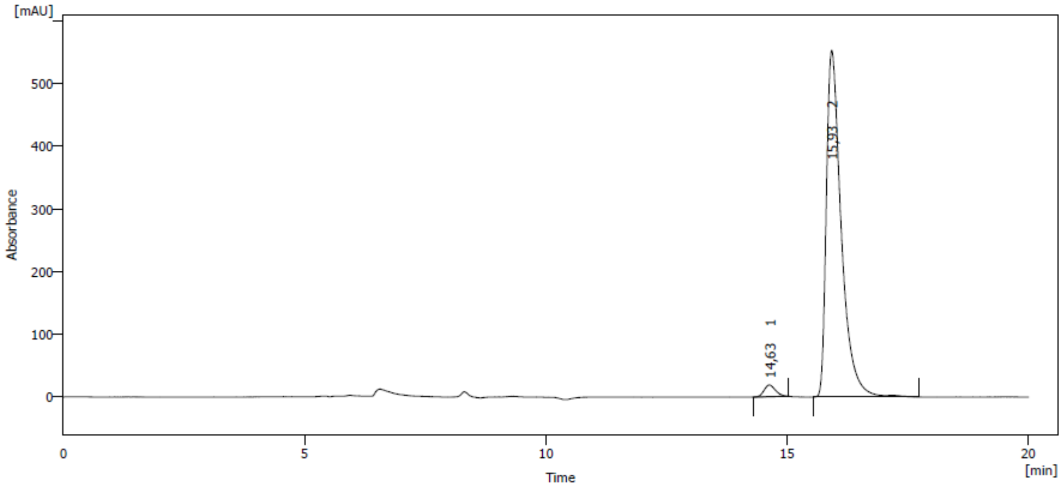

Result Table (Uncal - Data|67-KE-KAT-050 A_21_01_2025 11_43_48_452 - DAD 6.1L: Channel 1)

	Reten. Time [min]	Area [mAU.s]	Height [mAU]	Area [%]	Height [%]	W05 [min]	PDA Peak Purity
1	14,633	314,225	18,928	2,6	3,3	0,26	786
2	15,925	11877,988	553,357	97,4	96,7	0,31	748
	Total	12192,212	572,285	100,0	100,0		

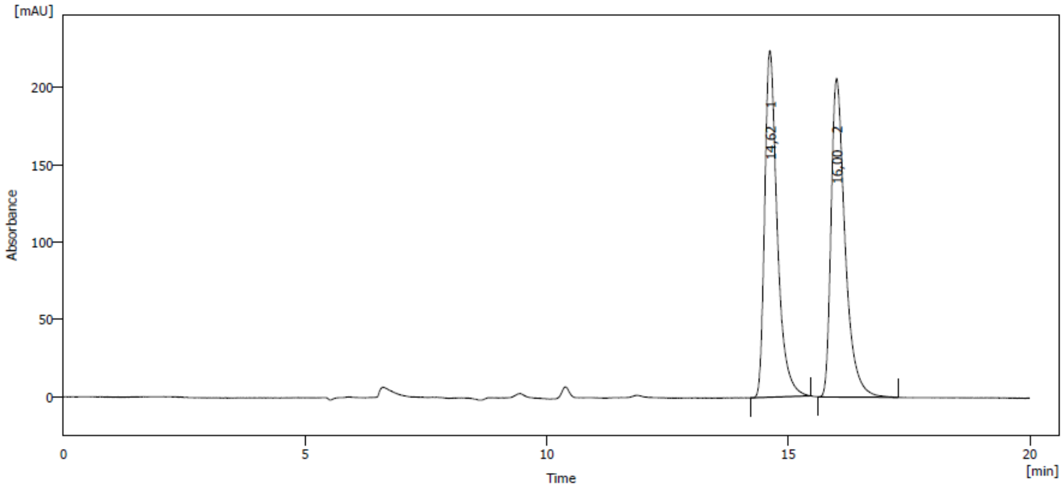

Result Table (Uncal - Data|67-MA-RAC-001_21_01_2025 11_23_12_451 - DAD 6.1L: Channel 1)

	Reten. Time [min]	Area [mAU.s]	Height [mAU]	Area [%]	Height [%]	W05 [min]	PDA Peak Purity
1	14,618	4053,802	224,534	49,3	52,1	0,27	791
2	15,998	4161,197	206,339	50,7	47,9	0,30	639
	Total	8214,999	430,874	100,0	100,0		

3aB

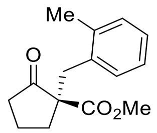

CHIRALPAK® *IJ*, cyclohexane/*i*PrOH = 97/3, 0.5 mL/min, λ = 215 nm

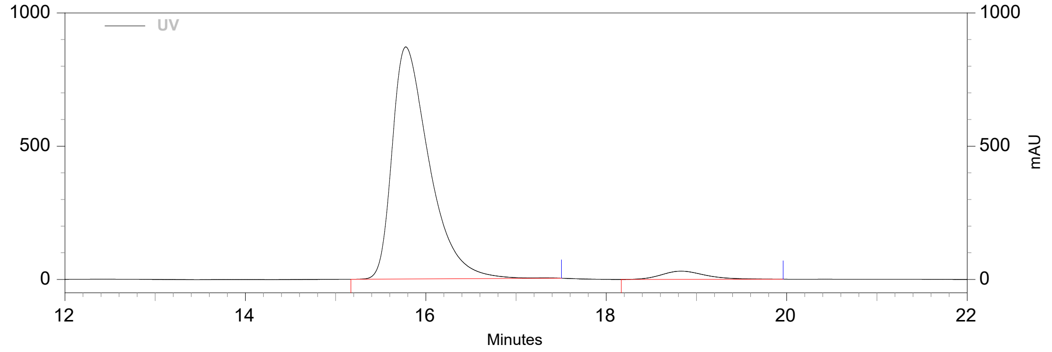

UV Results

Retention Time	Area	Area %	Height	Height %
15.777	99495235	95.97	3483174	96.58
18.830	4181869	4.03	123364	3.42
Totals	103677104	100.00	3606538	100.00

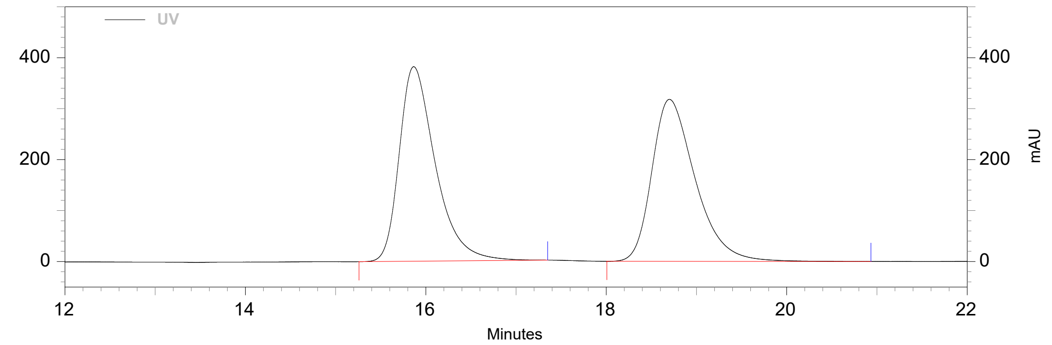

UV Results

Retention Time	Area	Area %	Height	Height %
15.867	42442639	50.01	1527486	54.57
18.700	42434018	49.99	1271897	45.43
Totals	84876657	100.00	2799383	100.00

3aC

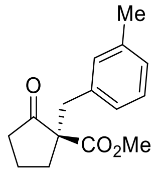

CHIRALPAK® *IJ*, cyclohexane/*i*PrOH = 97/3, 0.5 mL/min, *λ* = 215 nm

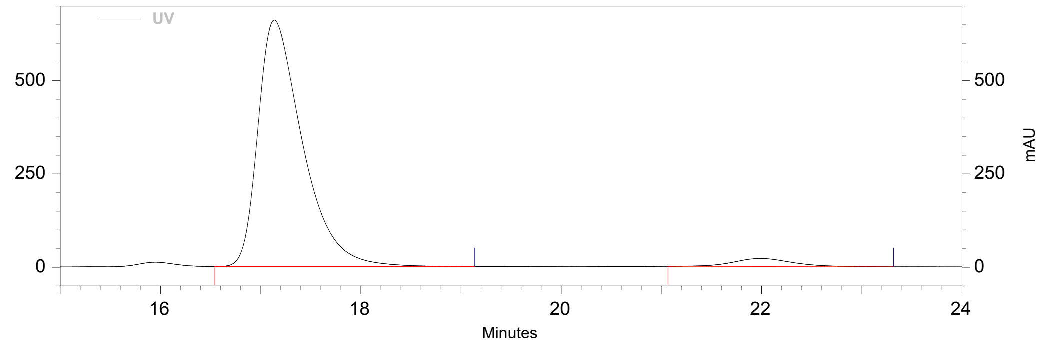

UV Results

Retention Time	Area	Area %	Height	Height %
17.137	81756116	95.85	2642280	96.84
21.987	3538179	4.15	86215	3.16
Totals	85294295	100.00	2728495	100.00

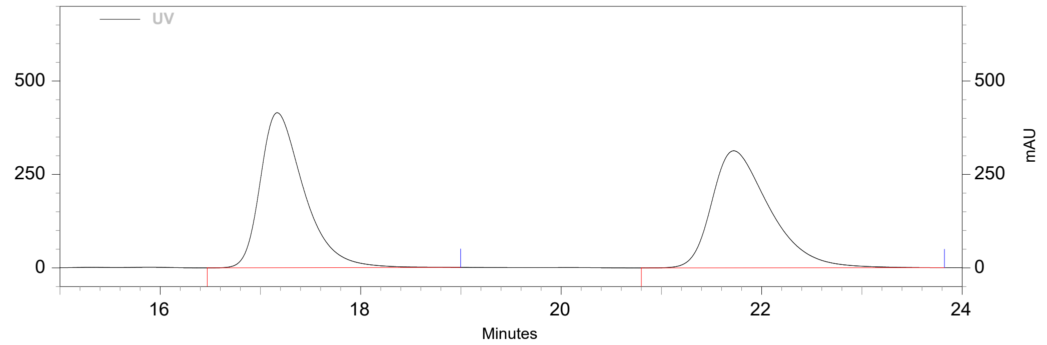

UV Results

Retention Time	Area	Area %	Height	Height %
17.167	50195361	49.96	1659191	56.97
21.720	50278842	50.04	1252983	43.03
Totals	100474203	100.00	2912174	100.00

3aD

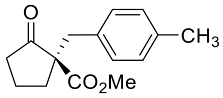

CHIRALPAK® *OD-H*, *n*-hexane/*i*PrOH = 97/3, 0.5 mL/min, *λ* = 215 nm

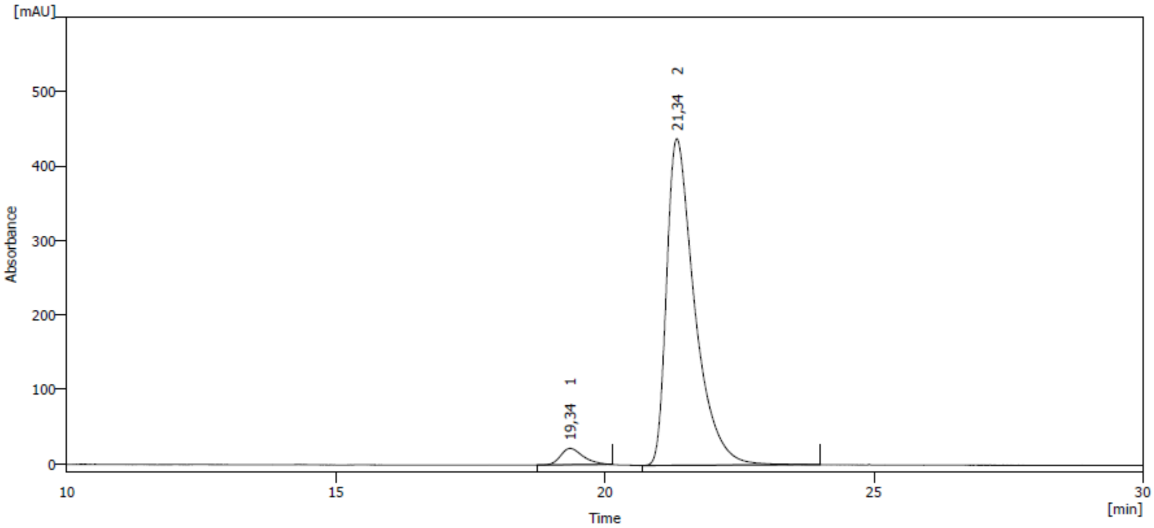

Result Table (Uncal - Data|67-KE-KAT-094 A sc_02_09_2025 12_11_11_790 - DAD 6.1L: Channel 1)

	Reten. Time [min]	Area [mAU.s]	Height [mAU]	Area [%]	Height [%]	W05 [min]	PDA Peak Purity
1	19,343	650,954	22,190	3,9	4,8	0,45	539
2	21,338	15920,042	439,026	96,1	95,2	0,53	545
	Total	16570,996	461,216	100,0	100,0		

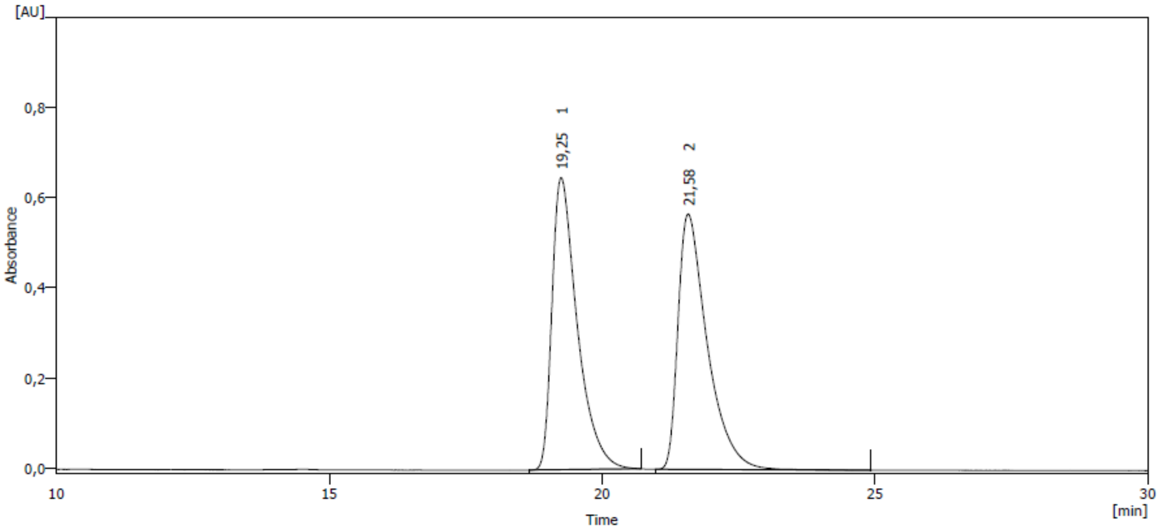

Result Table (Uncal - Data|67-KE-RAC-006 OD-H sc_02_09_2025 11_30_35_789 - DAD 6.1L: Channel 1)

	Reten. Time [min]	Area [mAU.s]	Height [mAU]	Area [%]	Height [%]	W05 [min]	PDA Peak Purity
1	19,253	20850,952	648,177	49,7	53,4	0,48	746
2	21,578	21116,808	566,747	50,3	46,6	0,55	429
	Total	41967,760	1214,924	100,0	100,0		

3aE

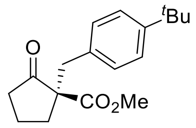

CHIRALPAK® *OD-H*, *n*-hexane/*i*PrOH = 97/3, 0.5 mL/min, λ = 215 nm

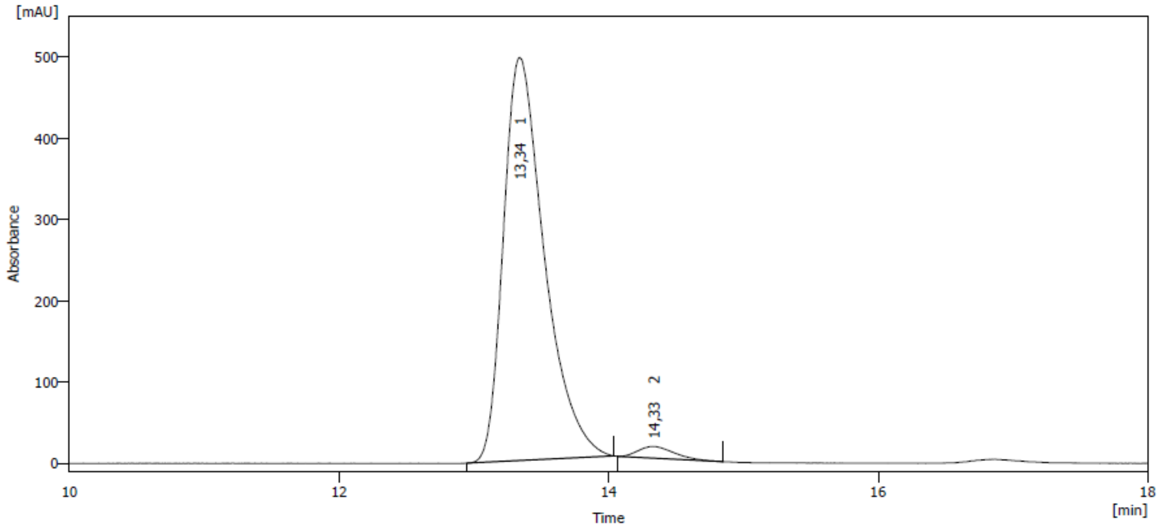

Result Table (Uncal - Data|67-KE-KAT-096A_06_03_2025 10_40_59_538 - DAD 6.1L: Channel 1)

	Reten. Time [min]	Area [mAU.s]	Height [mAU]	Area [%]	Height [%]	W05 [min]	PDA Peak Purity
1	13,338	10495,110	496,680	97,5	97,2	0,32	965
2	14,330	270,834	14,451	2,5	2,8	0,28	965
	Total	10765,944	511,131	100,0	100,0		

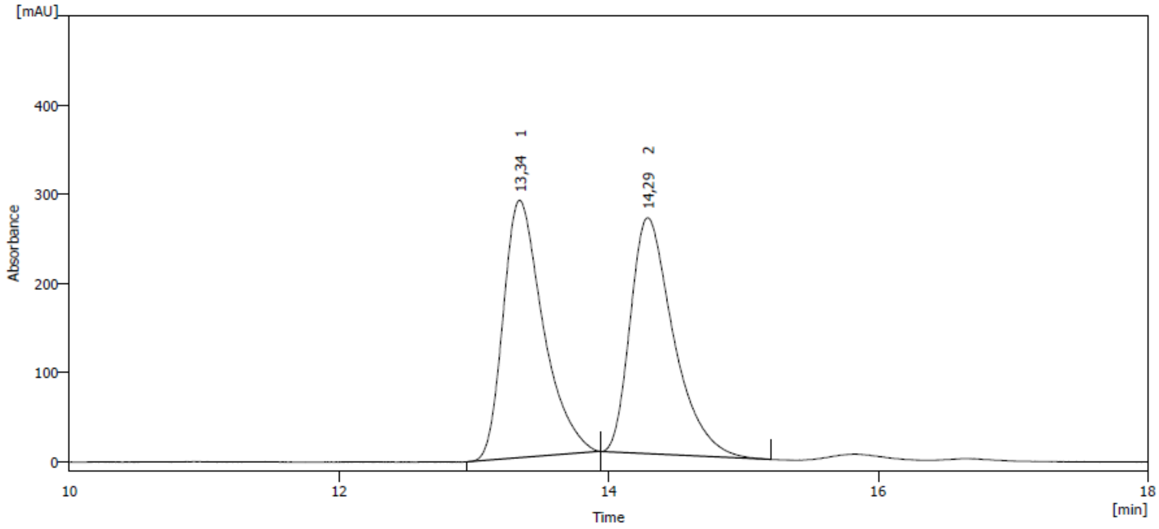

Result Table (Uncal - Data|67-KE-RAC-026_06_03_2025 09_58_17_537 - DAD 6.1L: Channel 1)

	Reten. Time [min]	Area [mAU.s]	Height [mAU]	Area [%]	Height [%]	W05 [min]	PDA Peak Purity
1	13,340	5940,433	289,097	50,2	52,2	0,31	961
2	14,288	5894,363	264,786	49,8	47,8	0,33	962
	Total	11834,796	553,883	100,0	100,0		

3aF

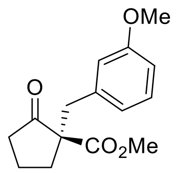

CHIRALPAK® *IJ*, cyclohexane/*i*PrOH = 97/3, 0.5 mL/min, *λ* = 215 nm

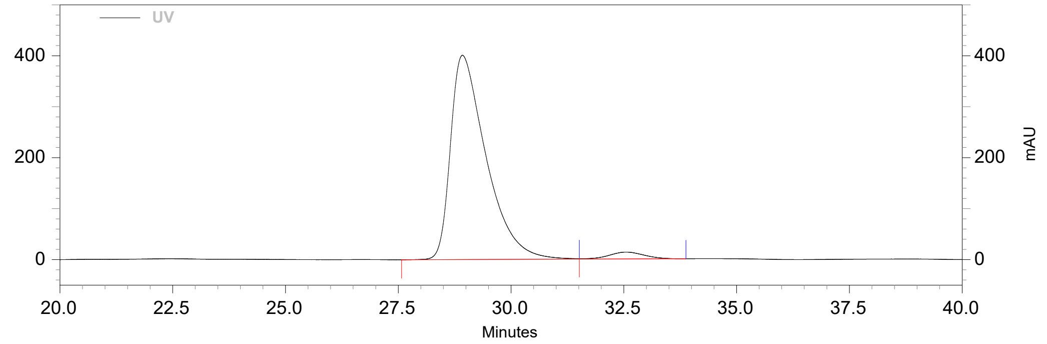

UV Results

Retention Time	Area	Area %	Height	Height %
28.920	87875490	96.80	1603737	96.75
32.547	2904743	3.20	53854	3.25

Totals	90780233	100.00	1657591	100.00

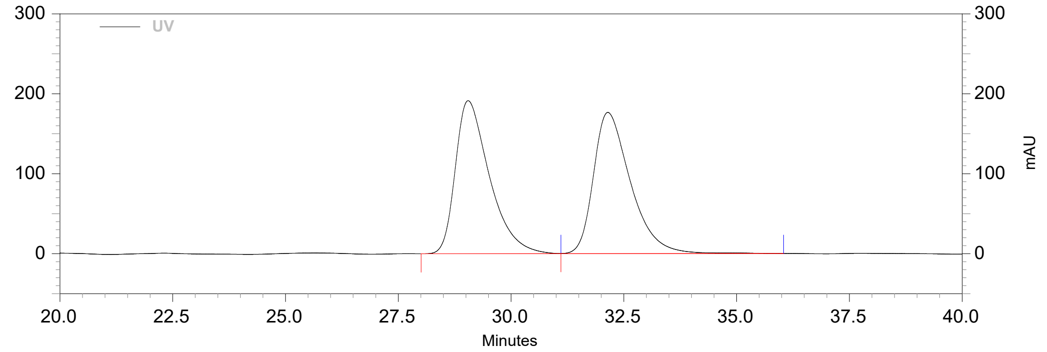

UV Results

Retention Time	Area	Area %	Height	Height %
29.047	40219294	49.94	765811	52.00
32.143	40310076	50.06	706768	48.00

Totals	80529370	100.00	1472579	100.00

3aG

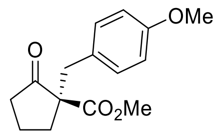

CHIRALPAK® *IJ*, *n*-hexane/*i*PrOH = 90/10, 0.5 mL/min, λ = 215 nm

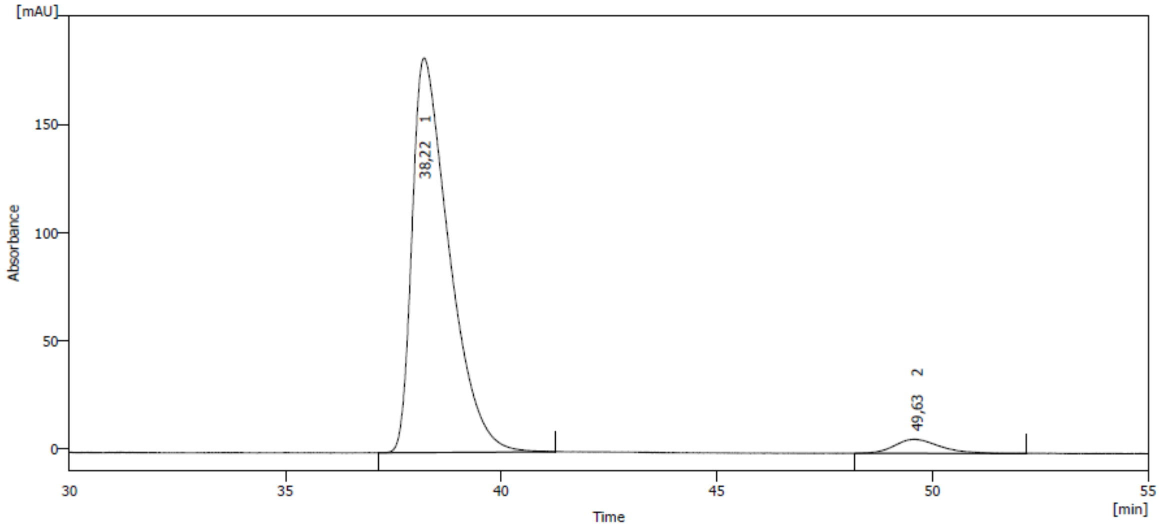

Result Table (Uncal - Data|67-KE-KAT-118A_09_04_2025 16_44_09_614 - DAD 6.1L: Channel 1)

	Reten. Time [min]	Area [mAU.s]	Height [mAU]	Area [%]	Height [%]	W05 [min]	PDA Peak Purity
1	38,217	10907,149	182,542	95,7	96,5	0,90	644
2	49,627	489,879	6,637	4,3	3,5	1,08	354
	Total	11397,028	189,179	100,0	100,0		

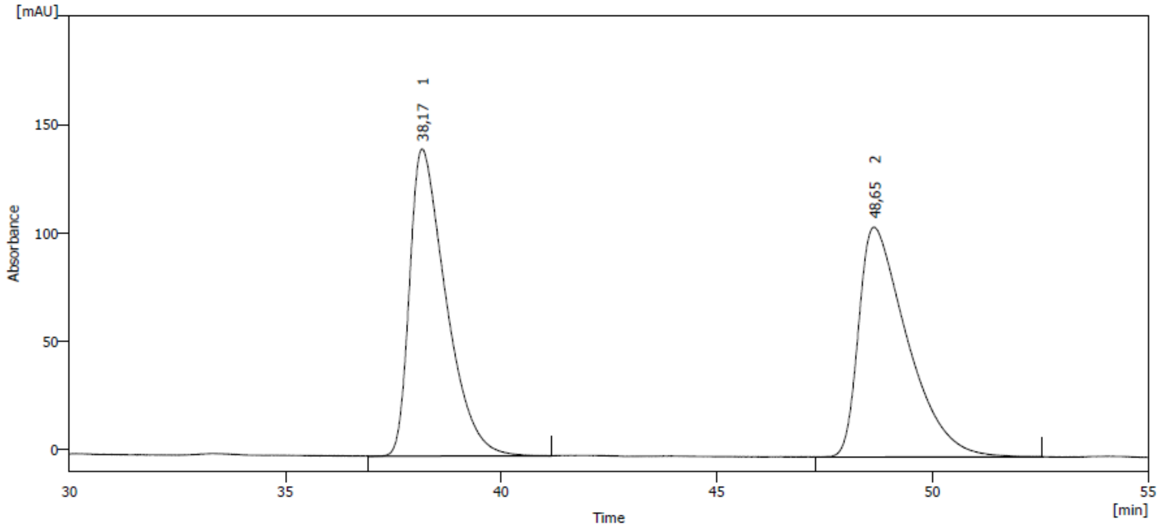

Result Table (Uncal - Data|67-KE-RAC-048_09_04_2025 15_45_35_613 - DAD 6.1L: Channel 1)

	Reten. Time [min]	Area [mAU.s]	Height [mAU]	Area [%]	Height [%]	W05 [min]	PDA Peak Purity
1	38,172	8330,353	141,891	50,0	57,2	0,89	529
2	48,652	8337,594	106,242	50,0	42,8	1,18	532
	Total	16667,947	248,134	100,0	100,0		

3aH

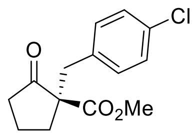

CHIRALPAK® *IC*, *n*-hexane/*i*PrOH = 97/3, 0.5 mL/min, *λ* = 215 nm

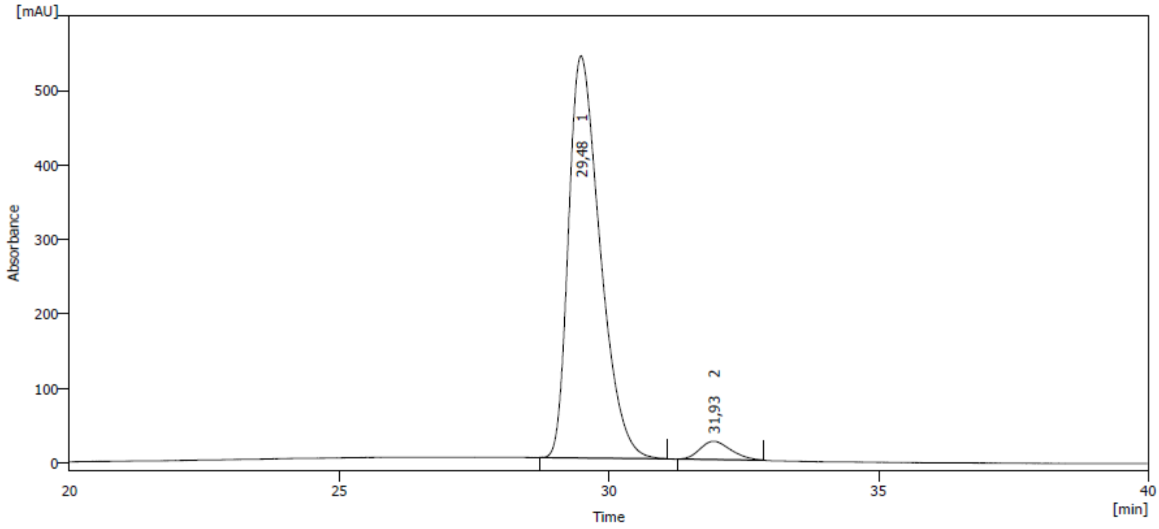

Result Table (Uncal - Data|67-KE-KAT-103 A_19_03_2025 10_29_22_573 - DAD 6.1L: Channel 1)

	Reten. Time [min]	Area [mAU.s]	Height [mAU]	Area [%]	Height [%]	W05 [min]	PDA Peak Purity
1	29,483	21991,417	540,336	95,7	95,6	0,63	909
2	31,933	977,965	24,590	4,3	4,4	0,62	931
	Total	22969,381	564,926	100,0	100,0		

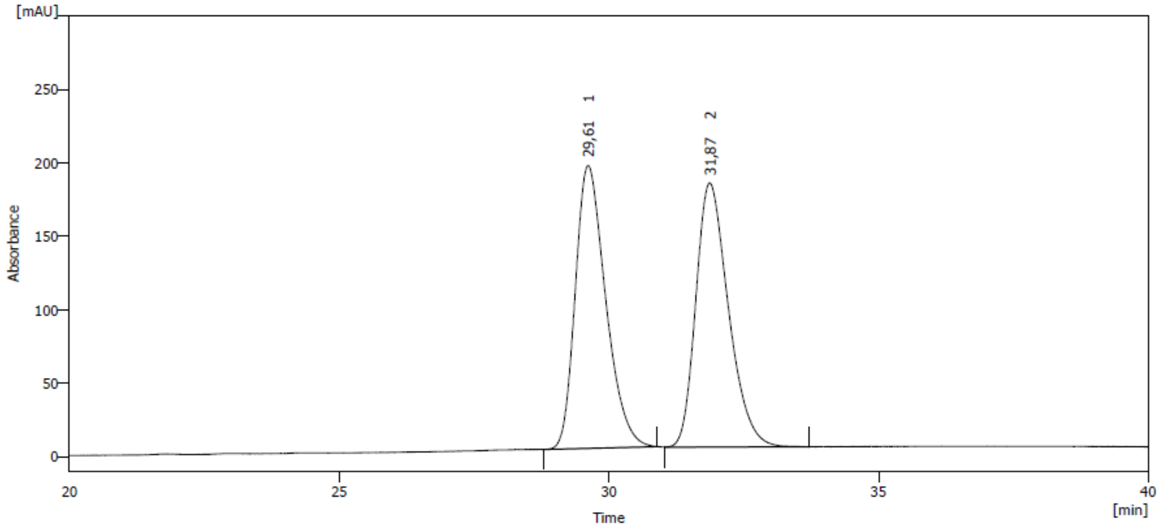

Result Table (Uncal - Data|67-KE-RAC-033 IC_18_03_2025 19_49_18_566 - DAD 6.1L: Channel 1)

	Reten. Time [min]	Area [mAU.s]	Height [mAU]	Area [%]	Height [%]	W05 [min]	PDA Peak Purity
1	29,610	7558,410	192,786	50,0	51,7	0,60	904
2	31,868	7566,819	180,147	50,0	48,3	0,65	877
	Total	15125,229	372,933	100,0	100,0		

3al

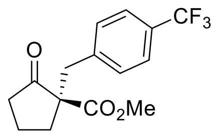

CHIRALPAK® *OD-H*, *n*-hexane/*i*PrOH = 99/1, 0.5 mL/min, *λ* = 215 nm

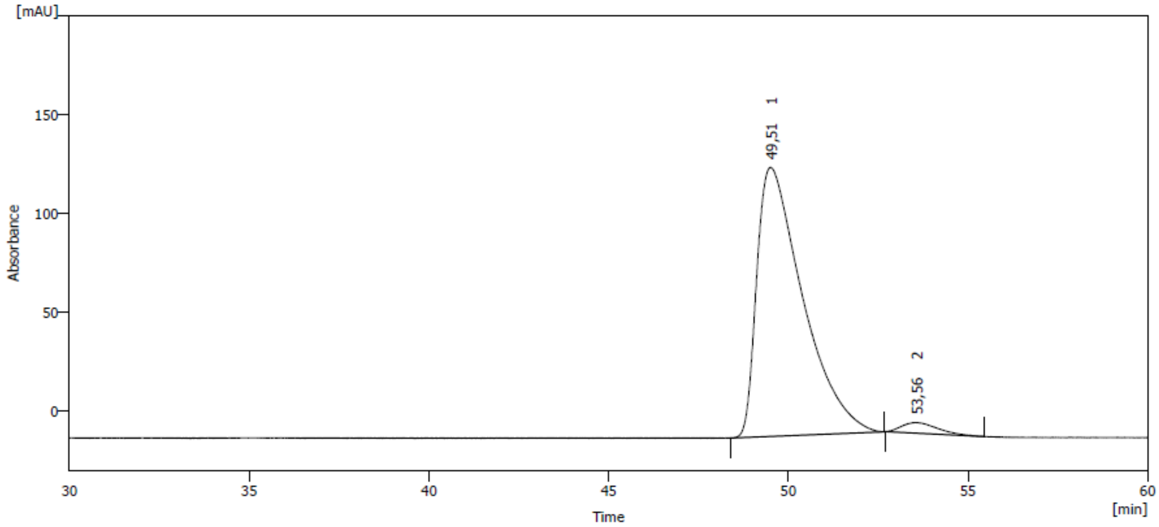

Result Table (Uncal - Data|67-KE-KAT-097A_06_03_2025 12_21_19_541 - DAD 6.1L: Channel 1)

	Reten. Time [min]	Area [mAU.s]	Height [mAU]	Area [%]	Height [%]	W05 [min]	PDA Peak Purity
1	49,512	12094,126	136,310	96,9	96,1	1,36	814
2	53,557	389,715	5,534	3,1	3,9	1,05	1000
	Total	12483,841	141,843	100,0	100,0		

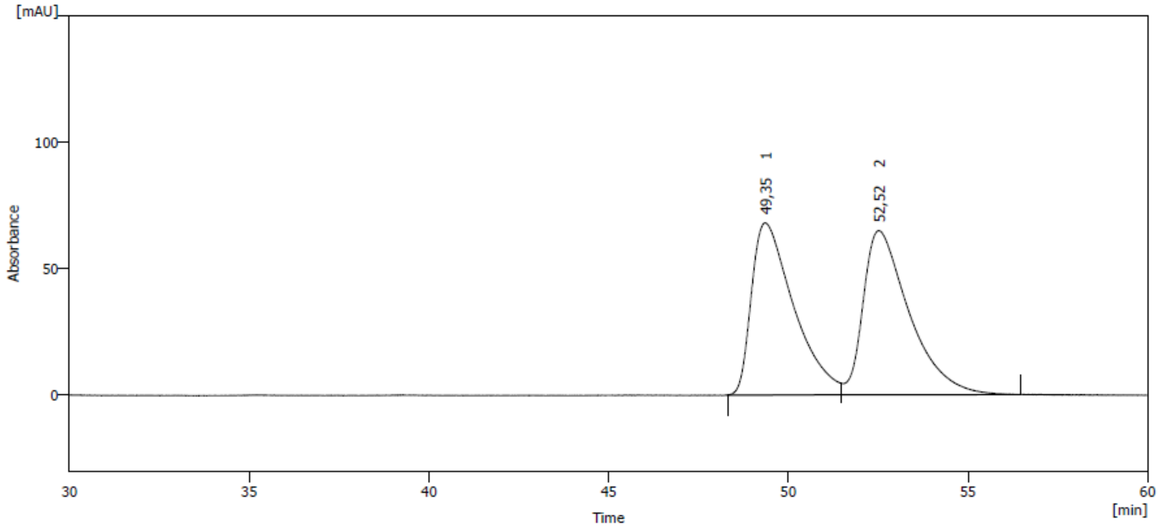

Result Table (Uncal - Data|67-KE-RAC-027_06_03_2025 13_24_34_542 - DAD 6.1L: Channel 1)

	Reten. Time [min]	Area [mAU.s]	Height [mAU]	Area [%]	Height [%]	W05 [min]	PDA Peak Purity
1	49,352	5581,891	68,265	48,9	51,2	1,25	901
2	52,518	5834,843	65,073	51,1	48,8	1,32	729
	Total	11416,734	133,338	100,0	100,0		

3aJ

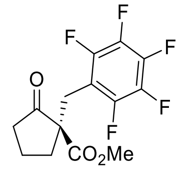

CHIRALPAK® *IC*, *n*-hexane/*i*PrOH = 97/3, 0.5 mL/min, λ = 208 nm

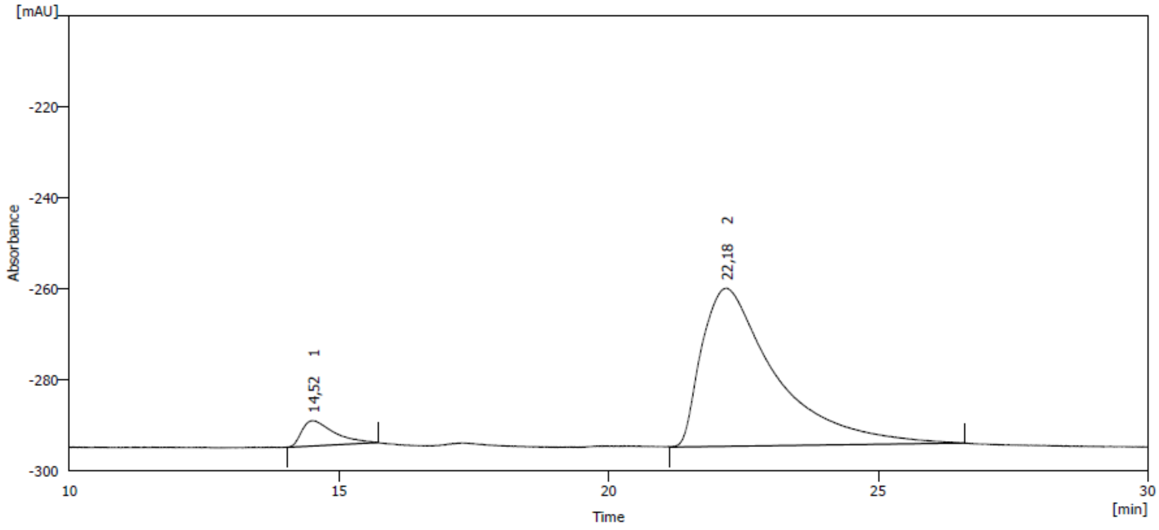

Result Table (Uncal - Data|67-KE-KAT-109A_03_04_2025 18_35_30_603 - DAD 6.1L: Channel 2)

	Reten. Time [min]	Area [mAU.s]	Height [mAU]	Area [%]	Height [%]	W05 [min]	PDA Peak Purity
1	14,517	235,204	5,651	6,6	13,9	0,66	973
2	22,185	3336,470	34,858	93,4	86,1	1,36	686
	Total	3571,674	40,509	100,0	100,0		

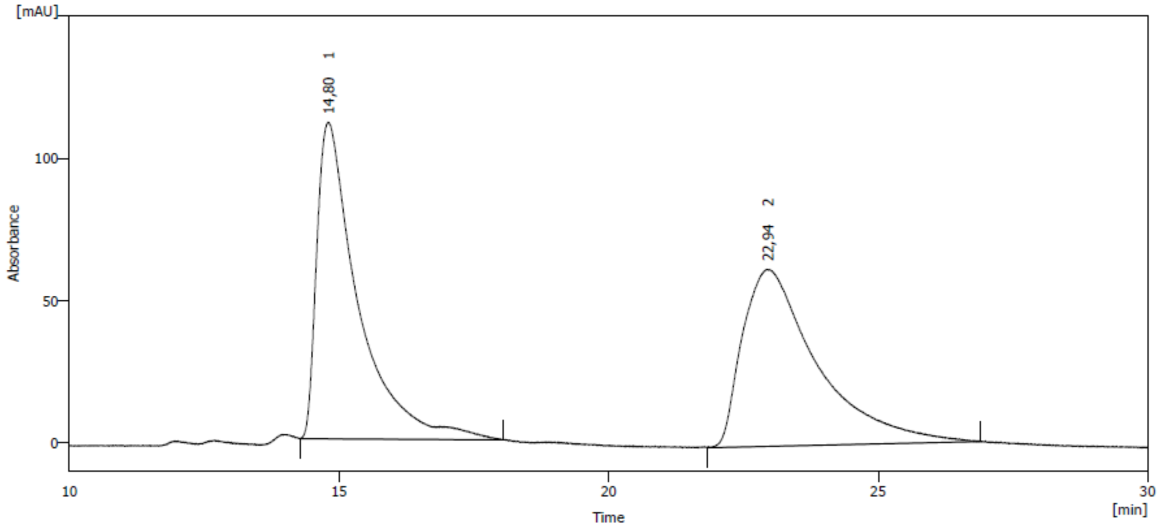

Result Table (Uncal - Data|67-KE-RAC-039_03_04_2025 17_56_22_602 - DAD 6.1L: Channel 2)

	Reten. Time [min]	Area [mAU.s]	Height [mAU]	Area [%]	Height [%]	W05 [min]	PDA Peak Purity
1	14,795	5937,329	111,522	49,7	64,1	0,71	689
2	22,938	6020,663	62,373	50,3	35,9	1,40	663
	Total	11957,992	173,895	100,0	100,0		

3aK

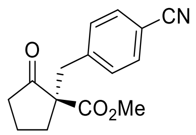

CHIRALPAK® *IJ*, cyclohexane/*i*PrOH = 90/10, 0.5 mL/min, *λ* = 215 nm

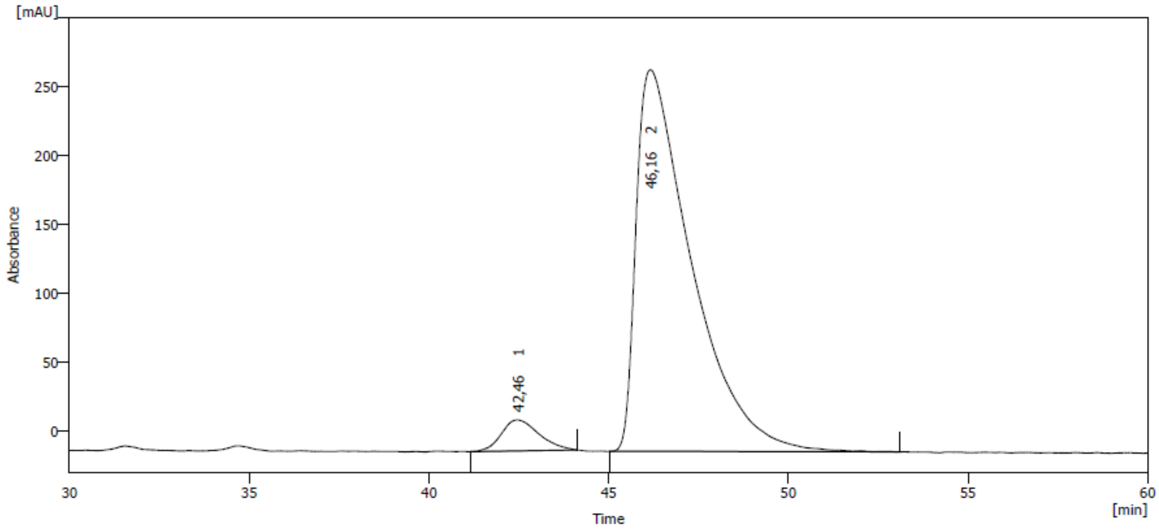

Result Table (Uncal - Data|67-KE-KAT-104A_31_03_2025 15_15_04_592 - DAD 6.1L: Channel 4)

	Reten. Time [min]	Area [mAU.s]	Height [mAU]	Area [%]	Height [%]	W05 [min]	PDA Peak Purity
1	42,463	1657,946	22,649	5,3	7,5	1,13	887
2	46,157	29774,762	277,393	94,7	92,5	1,58	377
	Total	31432.708	300.042	100.0	100.0		

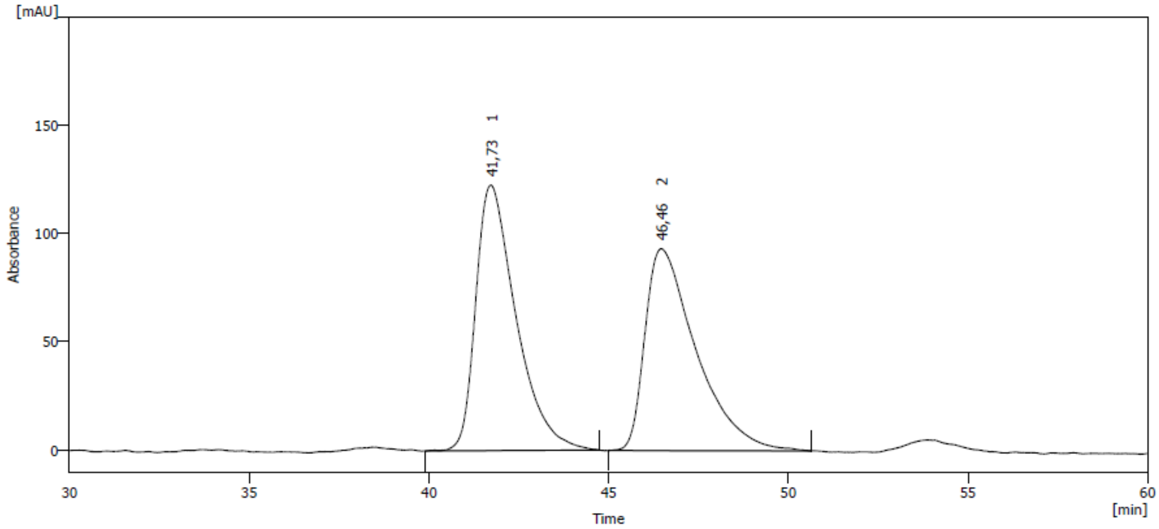

Result Table (Uncal - Data|67-KE-RAC-034 IJ_31_03_2025 16_15_43_593 - DAD 6.1L: Channel 4)

	Reten. Time [min]	Area [mAU.s]	Height [mAU]	Area [%]	Height [%]	W05 [min]	PDA Peak Purity
1	41,727	9570,289	122,761	50,8	56,8	1,16	576
2	46,460	9258,291	93,398	49,2	43,2	1,47	584
	Total	18828.579	216.159	100.0	100.0		

3aL

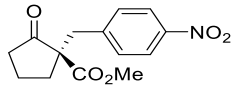

CHIRALPAK® *AS-H*, cyclohexane/*i*PrOH = 80/20, 0.2 mL/min, *λ* = 254 nm

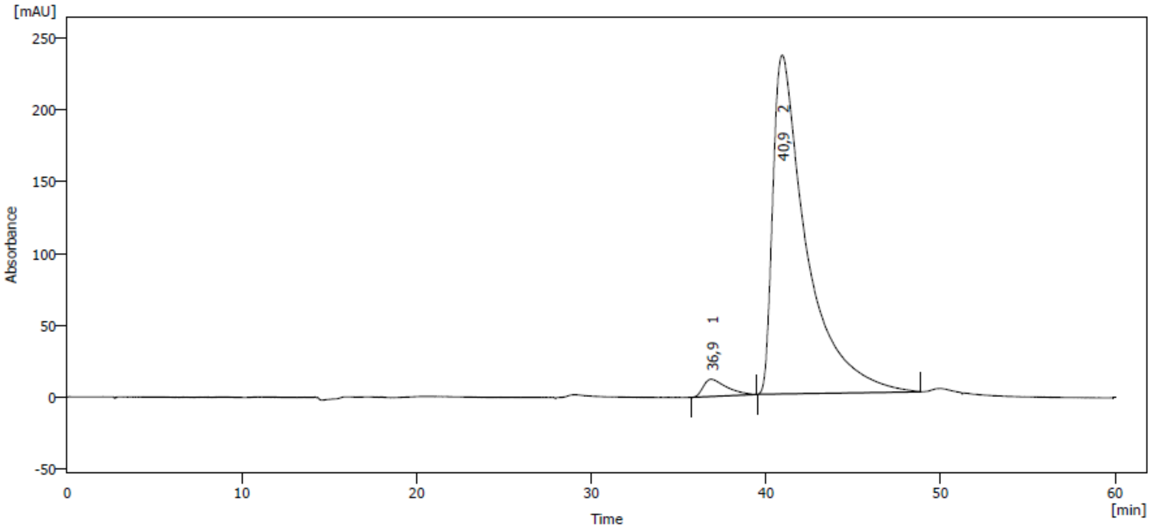

Result Table (Uncal - Data|67-KE-KAT-095B_03_04_2025 16_31_48_600 - DAD 6.1L: Channel 2)

	Reten. Time [min]	Area [mAU.s]	Height [mAU]	Area [%]	Height [%]	W05 [min]	PDA Peak Purity
1	36,920	1127,489	12,152	3,4	4,9	1,49	861
2	40,943	31657,199	236,164	96,6	95,1	1,79	759
	Total	32784,688	248,316	100,0	100,0		

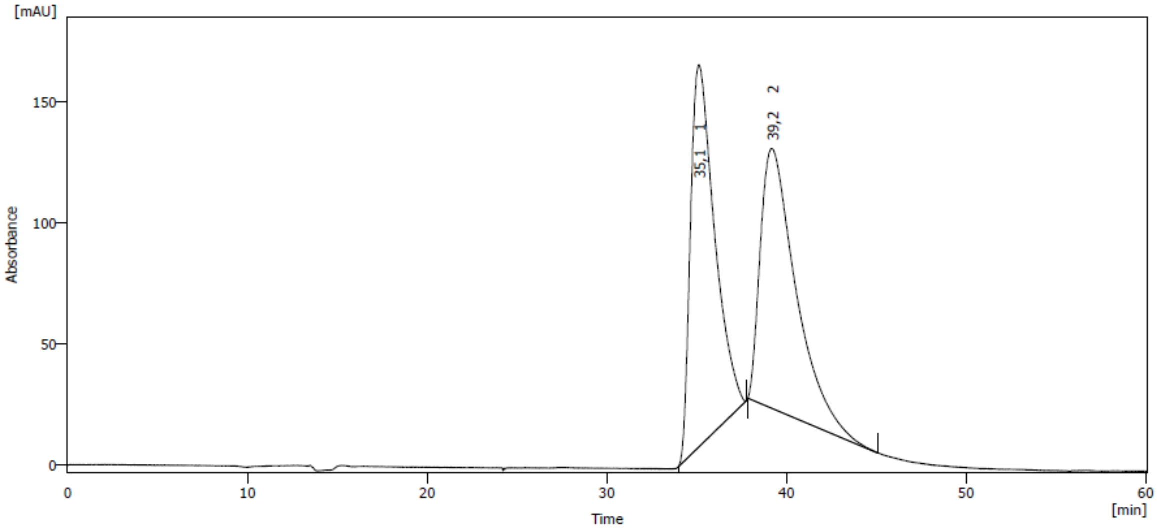

Result Table (Uncal - Data|67-KE-RAC-005 _06_12_2024 14_16_39_377 - DAD 6.1L: Channel 2)

	Reten. Time [min]	Area [mAU.s]	Height [mAU]	Area [%]	Height [%]	W05 [min]	PDA Peak Purity
1	35,128	14796,916	158,328	49,9	59,5	1,51	936
2	39,202	14884,067	107,672	50,1	40,5	1,99	883
	Total	29680,983	266,000	100,0	100,0		

3aM

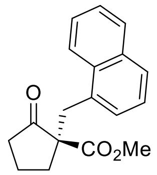

CHIRALPAK® *IJ*, cyclohexane/*i*PrOH = 97/3, 0.5 mL/min, λ = 215 nm

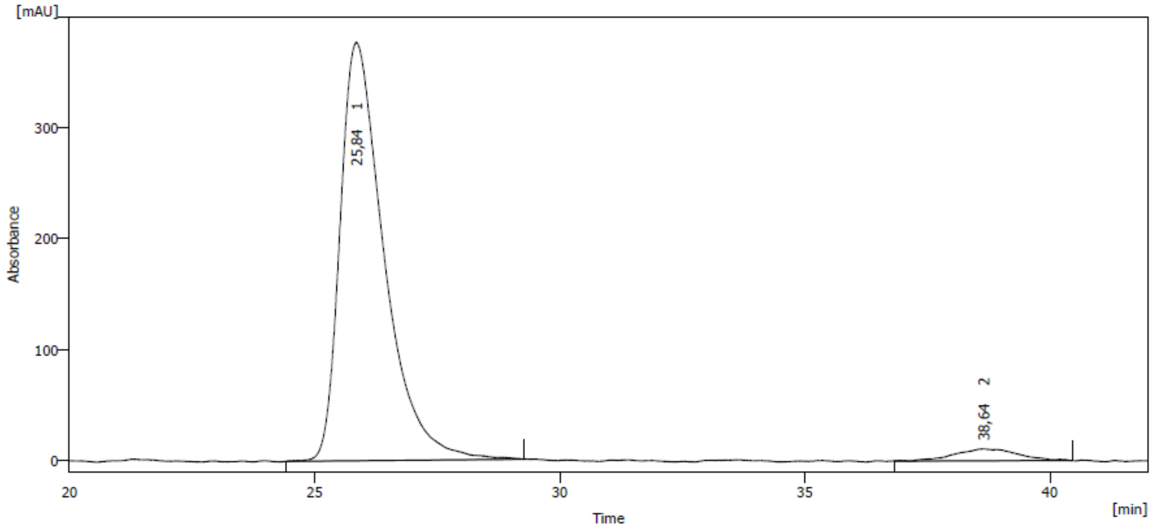

Result Table (Uncal - Data|67-KE-KAT-105A_31_03_2025 17_36_51_595 - DAD 6.1L: Channel 1)

	Reten. Time [min]	Area [mAU.s]	Height [mAU]	Area [%]	Height [%]	W05 [min]	PDA Peak Purity
1	25,843	23595,510	378,224	95,9	97,1	0,92	757
2	38,643	1000,348	11,100	4,1	2,9	1,40	795
	Total	24595.858	389.325	100.0	100.0		

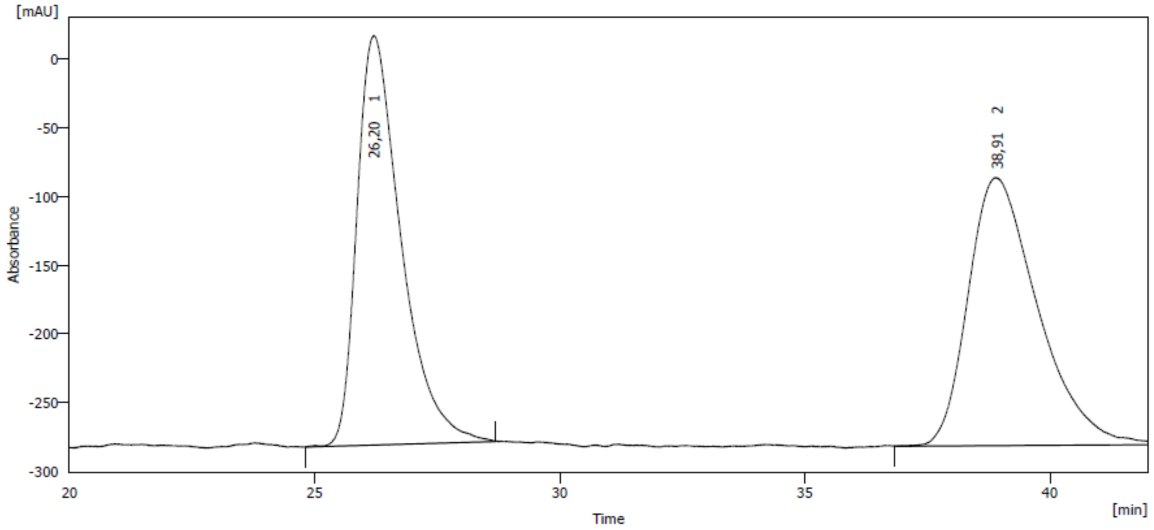

Result Table (Uncal - Data|67-KE-RAC-035 IJ_20_03_2025 10_25_31_576 - DAD 6.1L: Channel 1)

	Reten. Time [min]	Area [mAU.s]	Height [mAU]	Area [%]	Height [%]	W05 [min]	PDA Peak Purity
1	26,202	18624,773	297,442	49,2	60,4	0,94	519
2	38,913	19255,350	194,928	50,8	39,6	1,50	969
	Total	37880.123	492.370	100.0	100.0		

3aN

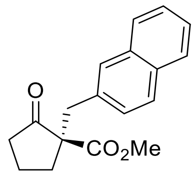

CHIRALPAK® *OD-H*, *n*-hexane/*i*PrOH = 99/1, 0.5 mL/min, *λ* = 215 nm

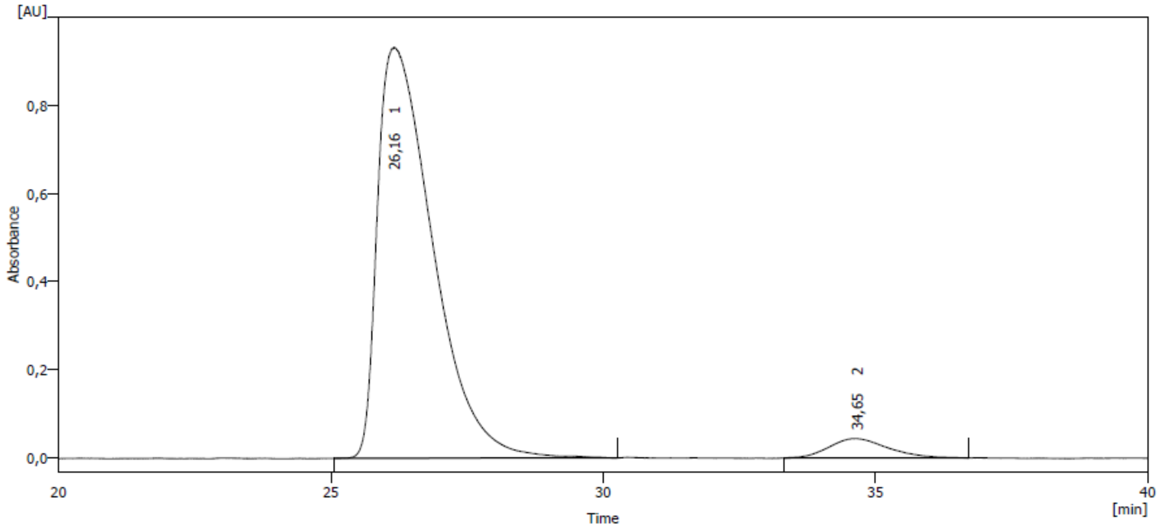

Result Table (Uncal - Data|67-KE-KAT-098A_20_03_2025 19_50_38_589 - DAD 6.1L: Channel 1)

	Reten. Time [min]	Area [mAU.s]	Height [mAU]	Area [%]	Height [%]	W05 [min]	PDA Peak Purity
1	26,155	65724,126	932,643	95,2	95,5	1,08	797
2	34,653	3318,713	44,288	4,8	4,5	1,15	796
	Total	69042.839	976.931	100.0	100.0		

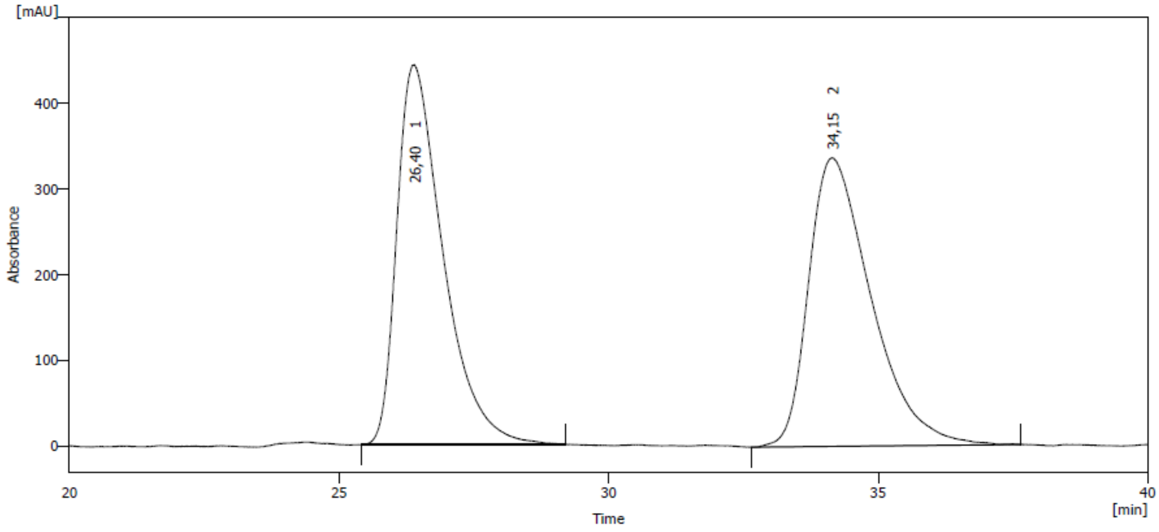

Result Table (Uncal - Data|67-KE-RAC-028 IJ_20_03_2025 18_50_02_588 - DAD 6.1L: Channel 1)
Noise (15,97-21,94 min): 2,3792 [mAU]

	Reten. Time [min]	Area [mAU.s]	Height [mAU]	Area [%]	Height [%]	W05 [min]	PDA Peak Purity
1	26,398	26451,246	443,274	49,3	56,8	0,89	881
2	34,148	27215,267	336,868	50,7	43,2	1,22	824

3aO

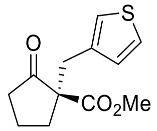

CHIRALPAK® *IA*, *n*-hexane/*i*PrOH = 97/3, 0.5 mL/min, *λ* = 215 nm

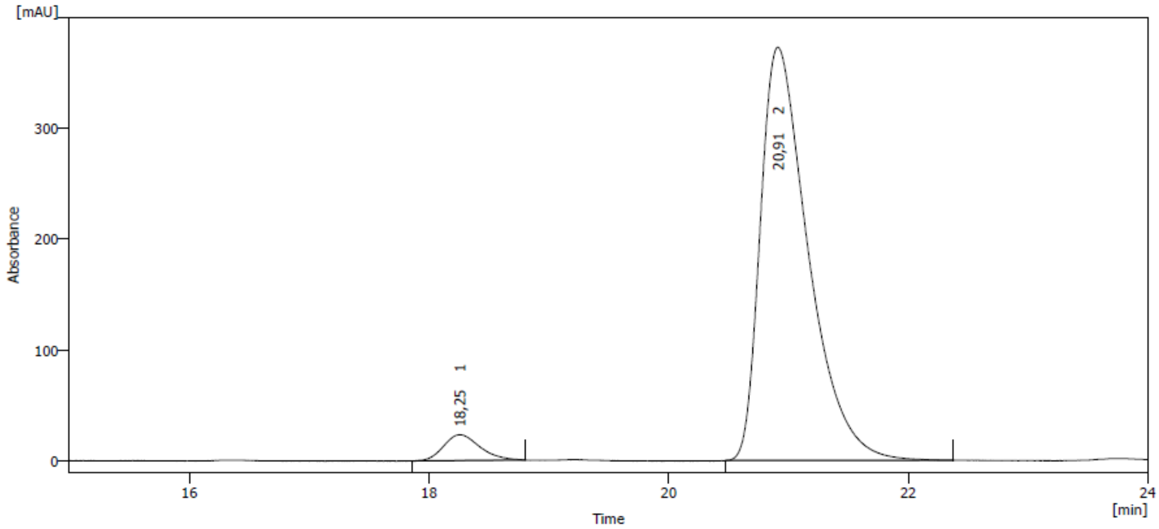

Result Table (Uncal - Data|67-KE-KAT-119A_16_04_2025 16_12_39_630 - DAD 6.1L: Channel 1)

	Reten. Time [min]	Area [mAU.s]	Height [mAU]	Area [%]	Height [%]	W05 [min]	PDA Peak Purity
1	18,253	493,794	23,392	4,5	5,9	0,33	793
2	20,912	10360,004	373,183	95,5	94,1	0,42	814
	Total	10853.797	396.575	100.0	100.0		

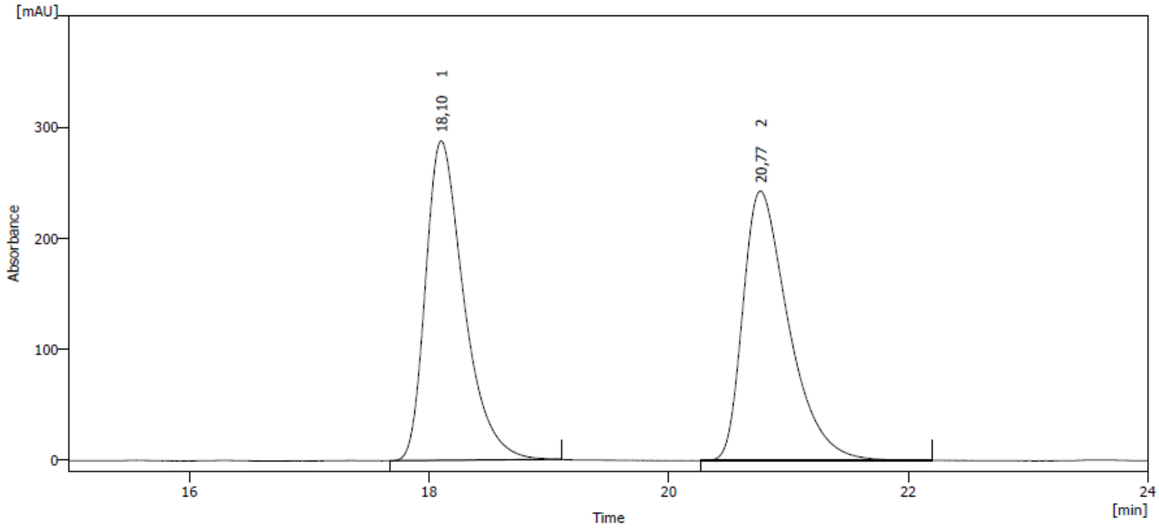

Result Table (Uncal - Data|67-KE-RAC-049_16_04_2025 15_19_30_628 - DAD 6.1L: Channel 1)

	Reten. Time [min]	Area [mAU.s]	Height [mAU]	Area [%]	Height [%]	W05 [min]	PDA Peak Purity
1	18,100	6413,093	288,149	49,9	54,2	0,34	770
2	20,765	6445,783	243,118	50,1	45,8	0,40	639
	Total	12858,876	531,266	100,0	100,0		

3aP

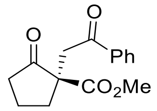

CHIRALPAK® *IJ*, *n*-hexane/*i*PrOH = 90/10, 0.5 mL/min, λ = 215 nm

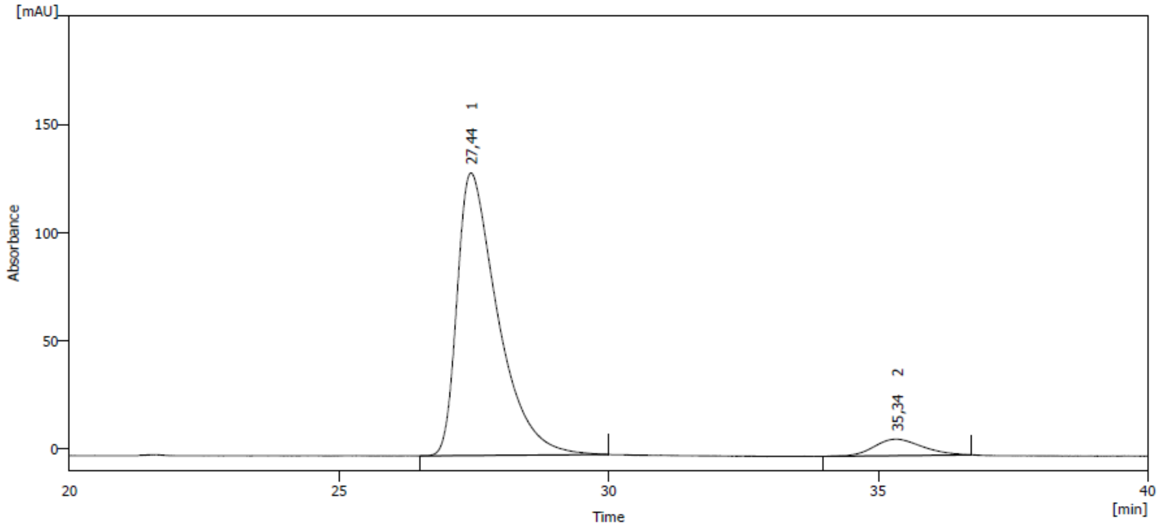

Result Table (Uncal - Data|67-KE-KAT-099B_25_04_2025 12_06_21_641 - DAD 6.1L: Channel 1)

	Reten. Time [min]	Area [mAU.s]	Height [mAU]	Area [%]	Height [%]	W05 [min]	PDA Peak Purity
1	27,443	6858,951	130,733	93,5	94,4	0,78	447
2	35,343	479,553	7,733	6,5	5,6	0,97	913
	Total	7338,504	138,465	100,0	100,0		

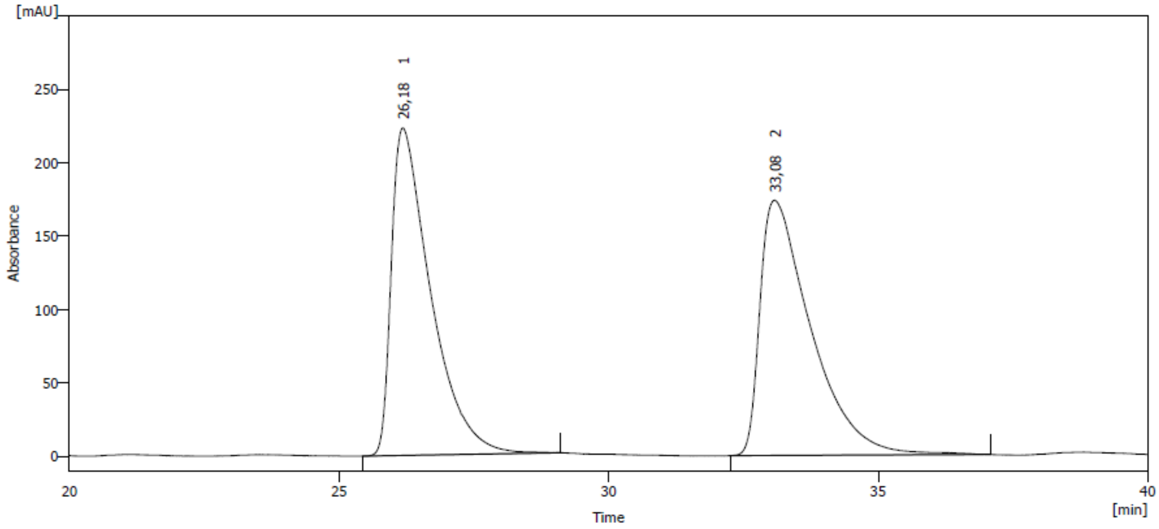

Result Table (Uncal - Data|67-KE-RAC-029 IJ_06_03_2025 17_55_56_547 - DAD 6.1L: Channel 1)

	Reten. Time [min]	Area [mAU.s]	Height [mAU]	Area [%]	Height [%]	W05 [min]	PDA Peak Purity
1	26,178	11307,137	223,366	49,7	56,2	0,75	863
2	33,078	11455,229	174,029	50,3	43,8	0,97	826
	Total	22762,366	397,394	100,0	100,0		

3aR

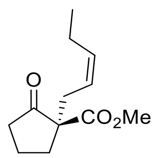

CHIRALPAK® *OD-H*, *n*-hexane/*i*PrOH = 97/3, 0.5 mL/min, *λ* = 202 nm

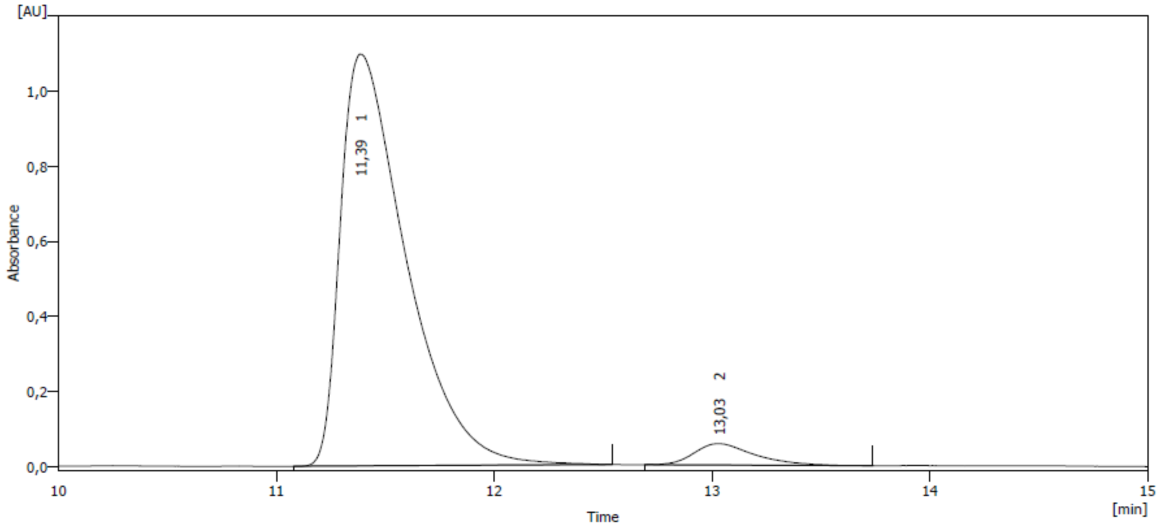

Result Table (Uncal - Data|67-KE-KAT-132A_25_07_2025 17_54_21_713 - DAD 6.1L: Channel 1)

	Reten. Time [min]	Area [mAU.s]	Height [mAU]	Area [%]	Height [%]	W05 [min]	PDA Peak Purity
1	11,385	23030,250	1097,401	95,4	95,0	0,32	918
2	13,032	1111,831	57,469	4,6	5,0	0,28	987
	Total	24142,080	1154,870	100,0	100,0		

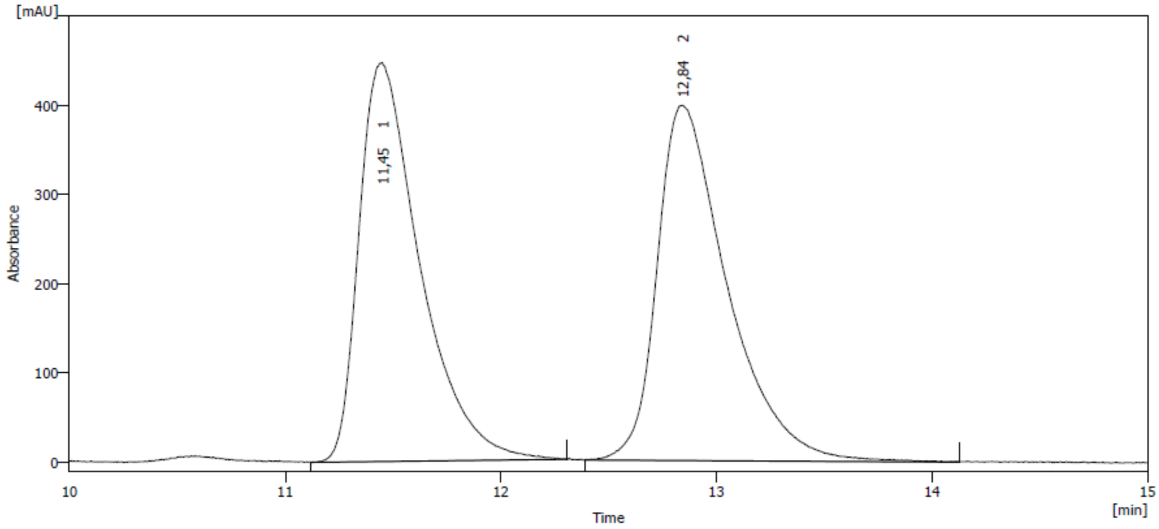

Result Table (Uncal - Data|67-KE-RAC-063 OD-H_25_07_2025 13_59_47_710 - DAD 6.1L: Channel 4)

	Reten. Time [min]	Area [mAU.s]	Height [mAU]	Area [%]	Height [%]	W05 [min]	PDA Peak Purity
1	11,450	8775,107	447,164	49,3	52,9	0,29	871
2	12,842	9034,203	398,545	50,7	47,1	0,33	744
	Total	17809,311	845,709	100,0	100,0		

3aS

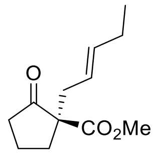

CHIRALPAK® *OD-H*, *n*-hexane/*i*PrOH = 97/3, 0.5 mL/min, *λ* = 215 nm

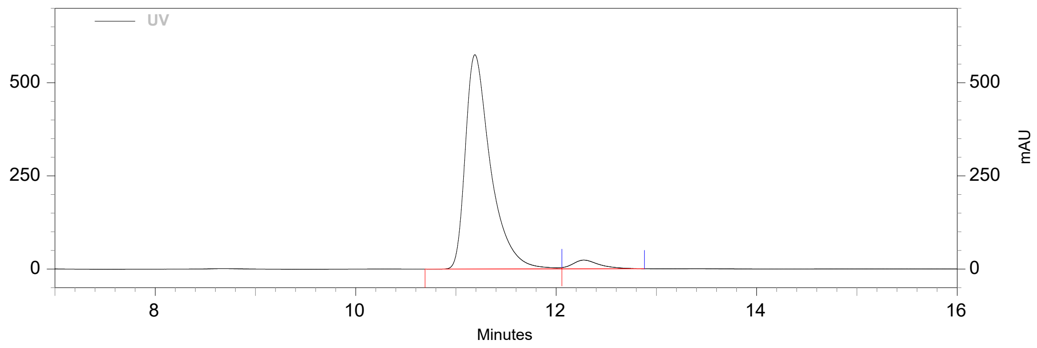

UV Results

Retention Time	Area	Area %	Height	Height %
11.190	40295342	95.87	2300955	96.09
12.277	1737484	4.13	93747	3.91
Totals	42032826	100.00	2394702	100.00

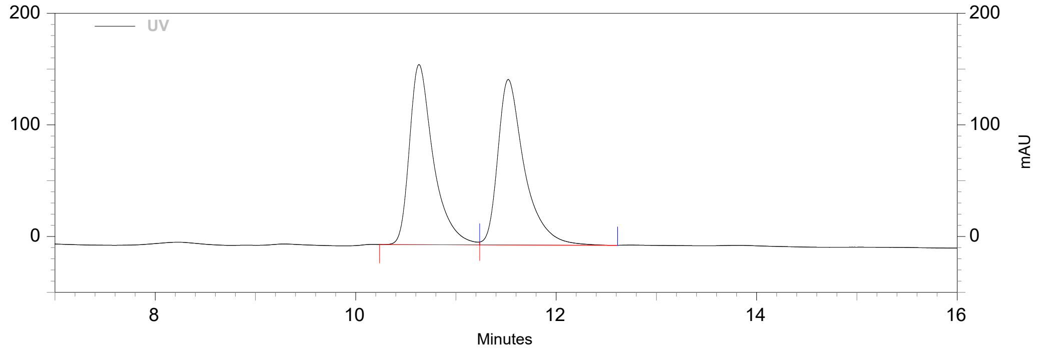

UV Results

Retention Time	Area	Area %	Height	Height %
10.630	10703561	49.56	645507	52.13
11.520	10892587	50.44	592752	47.87
Totals	21596148	100.00	1238259	100.00

3aT

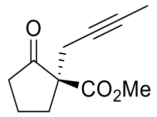

CHIRALPAK® *OD-H*, *n*-hexane/*i*PrOH = 97/3, 0.5 mL/min, *λ* = 204 nm

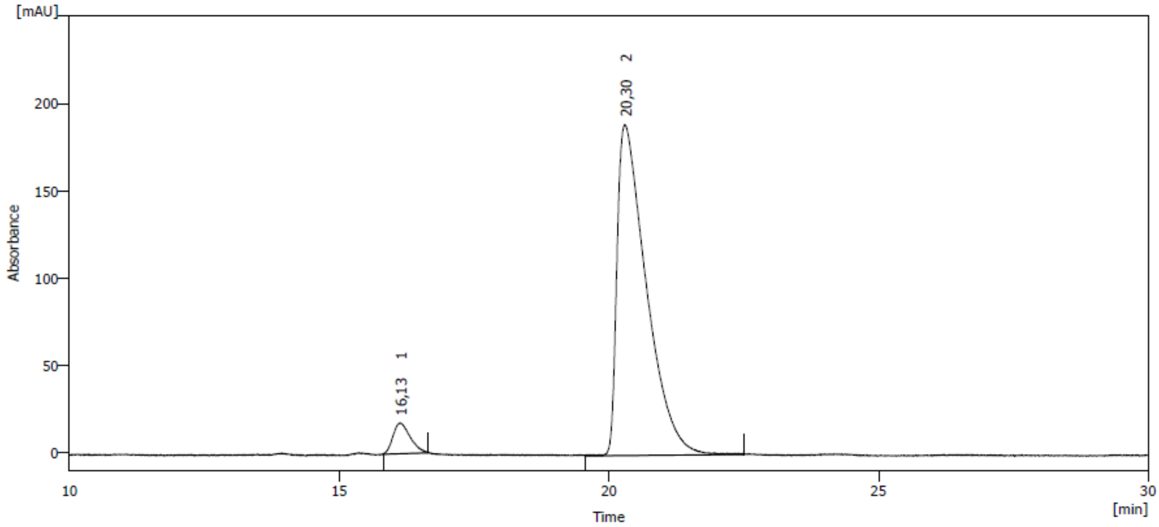

Result Table (Uncal - Data|67-KE-KAT-110 A_10_04_2025 10_40_22_621 - DAD 6.1L: Channel 1)

	Reten. Time [min]	Area [mAU.s]	Height [mAU]	Area [%]	Height [%]	W05 [min]	PDA Peak Purity
1	16,133	389,864	17,692	5,2	8,5	0,35	796
2	20,302	7170,937	189,521	94,8	91,5	0,57	469
	Total	7560,801	207,213	100,0	100,0		

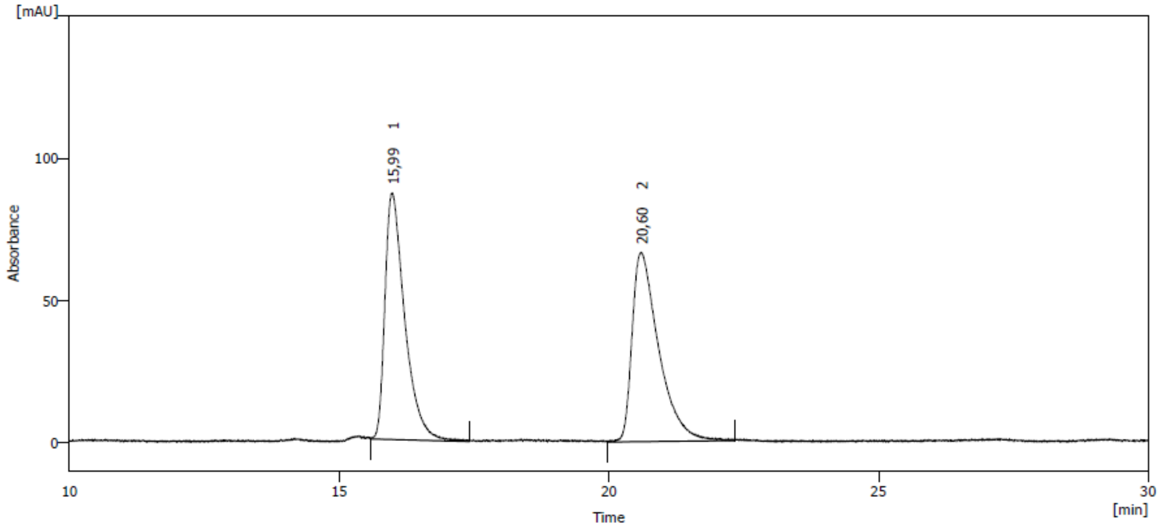

Result Table (Uncal - Data|67-KE-RAC-040 OD-H_10_04_2025 09_51_03_620 - DAD 6.1L: Channel 1)

	Reten. Time [min]	Area [mAU.s]	Height [mAU]	Area [%]	Height [%]	W05 [min]	PDA Peak Purity
1	15,992	2217,845	86,858	49,2	56,6	0,38	599
2	20,602	2292,274	66,674	50,8	43,4	0,51	599
	Total	4510,119	153,533	100,0	100,0		

3aAA

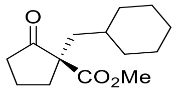

CHIRALPAK® *IA*, *n*-hexane/iPrOH = 97/3, 0.5 mL/min, λ = 215 nm

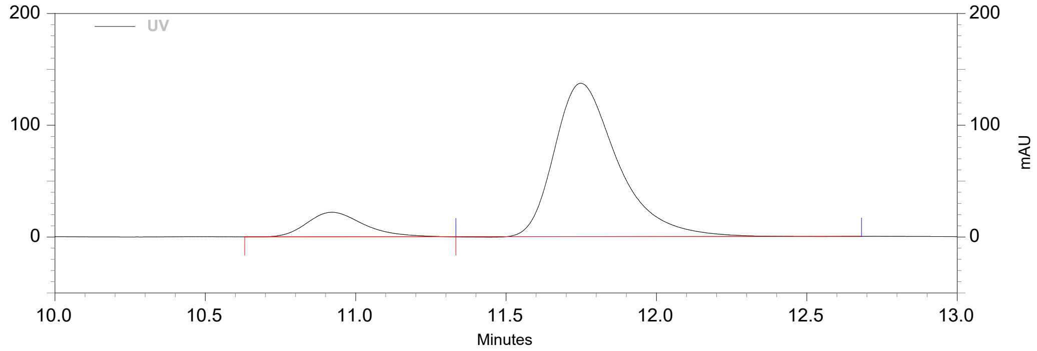

UV Results

Retention Time	Area	Area %	Height	Height %
10.920	1144289	12.50	87836	13.80
11.750	8008757	87.50	548826	86.20
Totals	9153046	100.00	636662	100.00

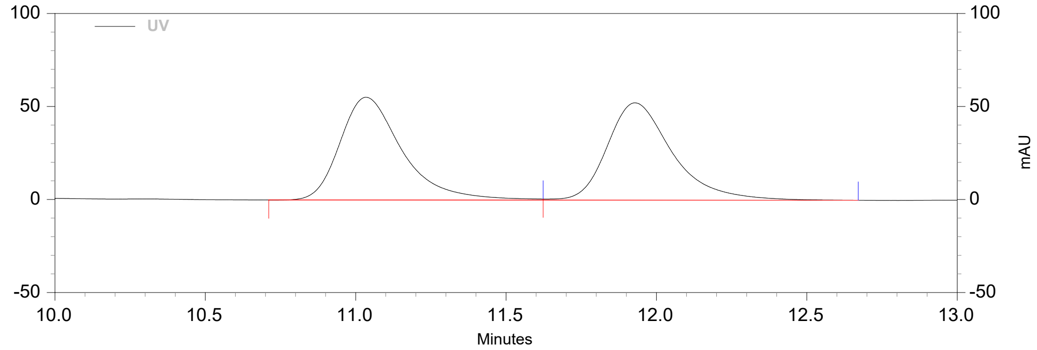

UV Results

Retention Time	Area	Area %	Height	Height %
11.033	3185317	50.21	221049	51.37
11.930	3159219	49.79	209257	48.63
Totals	6344536	100.00	430306	100.00

3bA

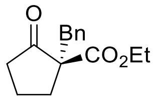

CHIRALPAK® *IA*, *n*-hexane/*i*PrOH = 98/2, 0.5 mL/min, *λ* = 215 nm

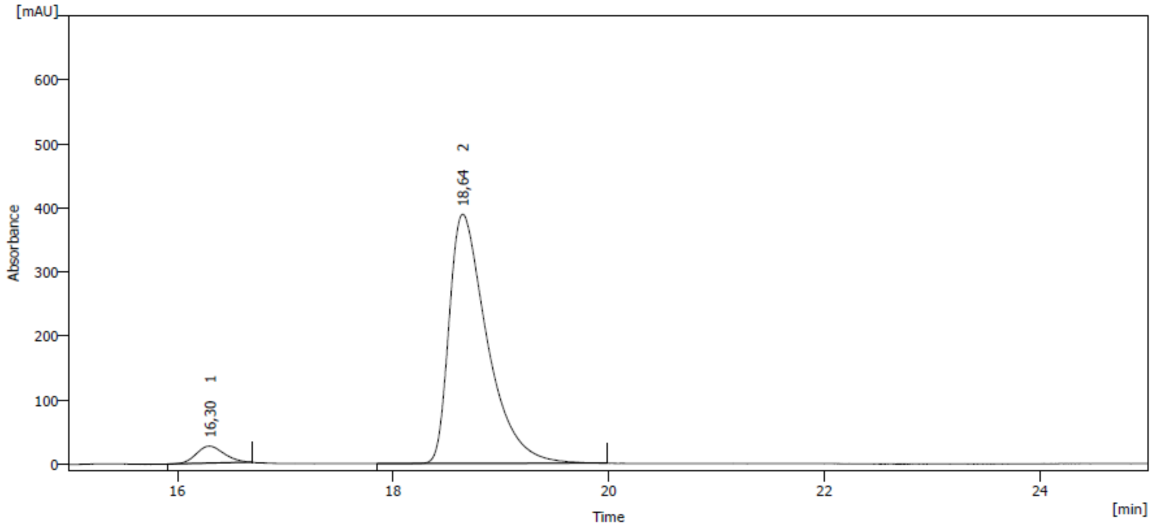

Result Table (Uncal - Data|67-KE-KAT-076 B_21_01_2025 13_05_32_455 - DAD 6.1L: Channel 1)

	Reten. Time [min]	Area [mAU.s]	Height [mAU]	Area [%]	Height [%]	W05 [min]	PDA Peak Purity
1	16,298	482,061	26,707	4,8	6,4	0,28	752
2	18,642	9559,832	389,485	95,2	93,6	0,36	650
	Total	10041,894	416,192	100,0	100,0		

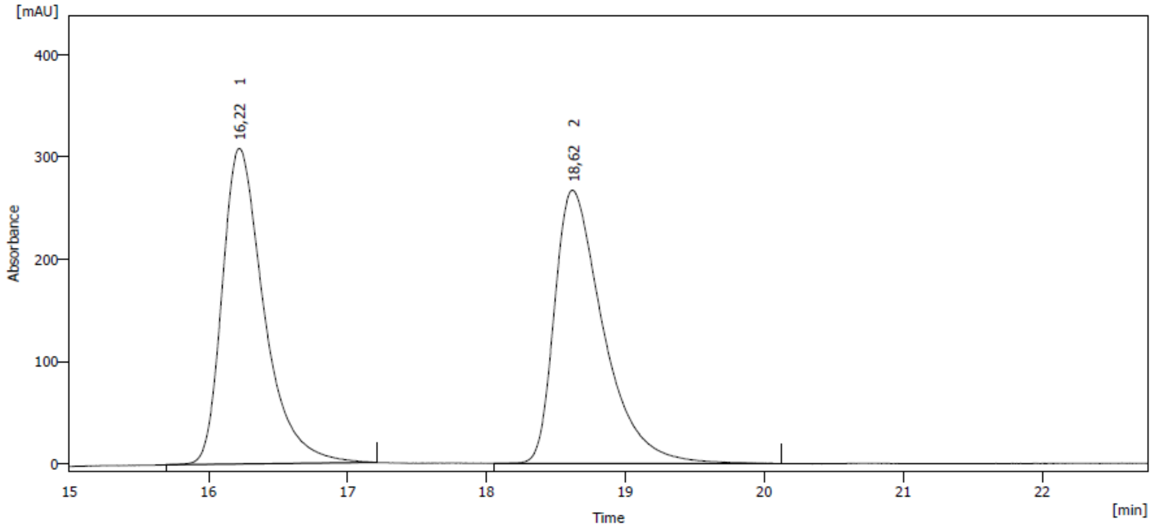

Result Table (Uncal - Data|67-KE-RAC-007_21_01_2025 12_34_56_454 - DAD 6.1L: Channel 1)

	Reten. Time [min]	Area [mAU.s]	Height [mAU]	Area [%]	Height [%]	W05 [min]	PDA Peak Purity
1	16,220	6444,585	308,589	49,7	53,6	0,31	803
2	18,623	6529,593	267,157	50,3	46,4	0,36	732
	Total	12974,178	575,746	100,0	100,0		

3cA

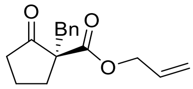

CHIRALPAK® *IA*, *n*-hexane/iPrOH = 99/1, 0.4 mL/min, λ = 215 nm

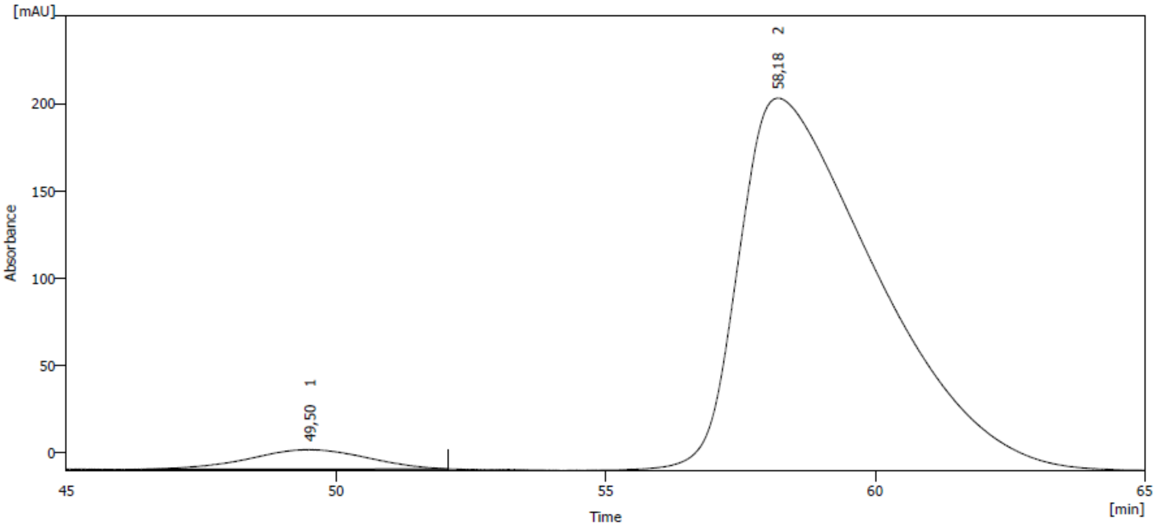

Result Table (Uncal - Data|67-KE-KAT-080 A_30_01_2025 10_12_14_459 - DAD 6.1L: Channel 1)

	Reten. Time [min]	Area [mAU.s]	Height [mAU]	Area [%]	Height [%]	W05 [min]	PDA Peak Purity
1	49,498	1717,410	11,288	4,3	5,0	2,39	996
2	58,182	38005,802	213,457	95,7	95,0	2,74	320
	Total	39723,213	224,745	100,0	100,0		

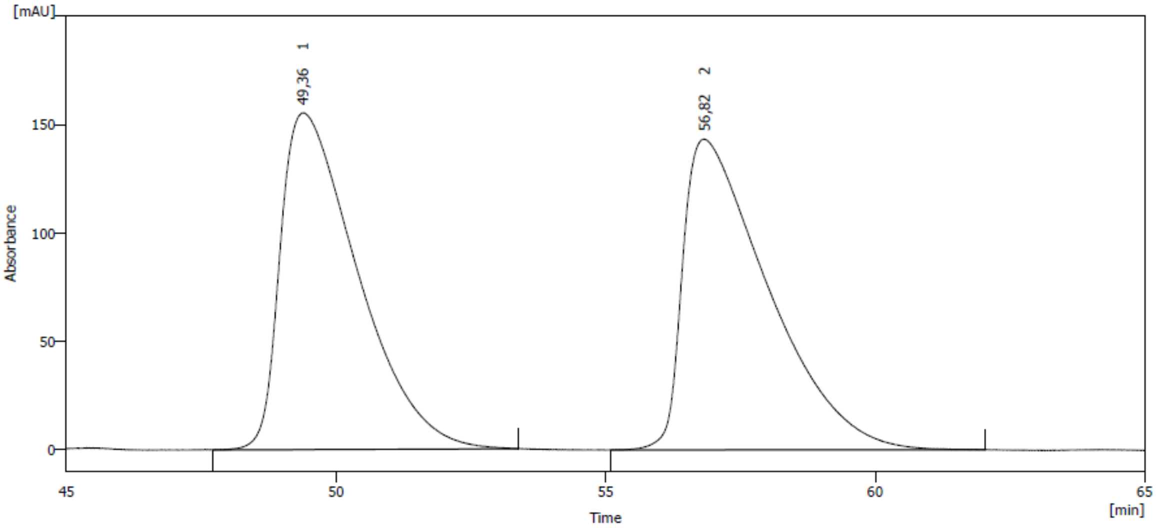

Result Table (Uncal - Data|67-KE-RAC-011_30_01_2025 11_27_31_460 - DAD 6.1L: Channel 1)

	Reten. Time [min]	Area [mAU.s]	Height [mAU]	Area [%]	Height [%]	W05 [min]	PDA Peak Purity
1	49,363	15547,156	155,486	49,6	52,0	1,54	888
2	56,822	15829,176	143,625	50,4	48,0	1,69	839
	Total	31376,331	299,111	100,0	100,0		

3dA

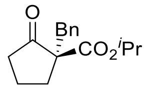

CHIRALPAK® *IA*, *n*-hexane/*i*PrOH = 98/2, 0.5 mL/min, *λ* = 215 nm

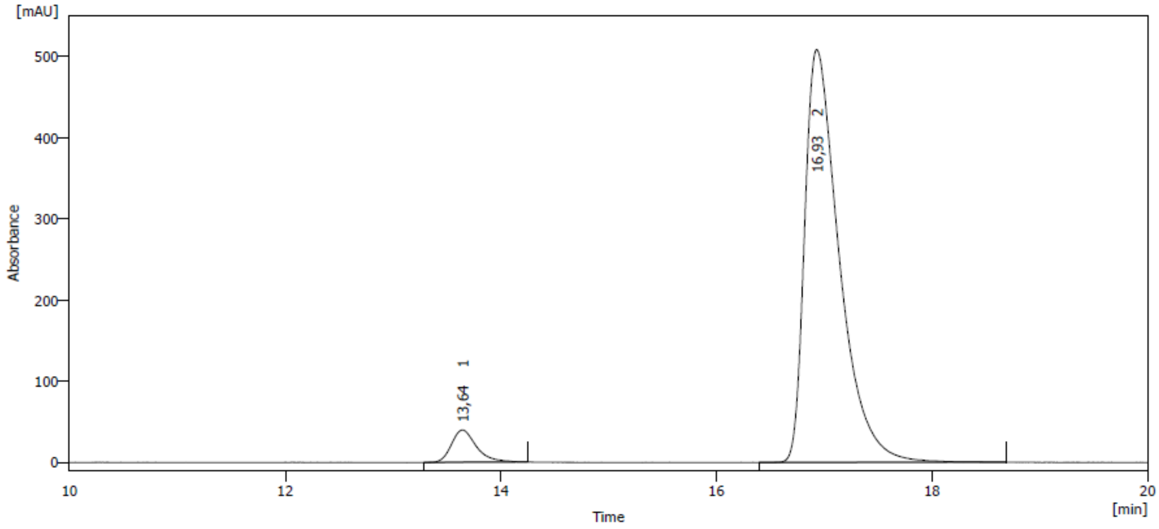

Result Table (Uncal - Data|67-KE-KAT-077 B_17_01_2025 17_12_39_445 - DAD 6.1L: Channel 1)

	Reten. Time [min]	Area [mAU.s]	Height [mAU]	Area [%]	Height [%]	W05 [min]	PDA Peak Purity
1	13,642	647,469	39,988	5,4	7,3	0,24	663
2	16,930	11355,346	509,221	94,6	92,7	0,33	615
	Total	12002,815	549,209	100,0	100,0		

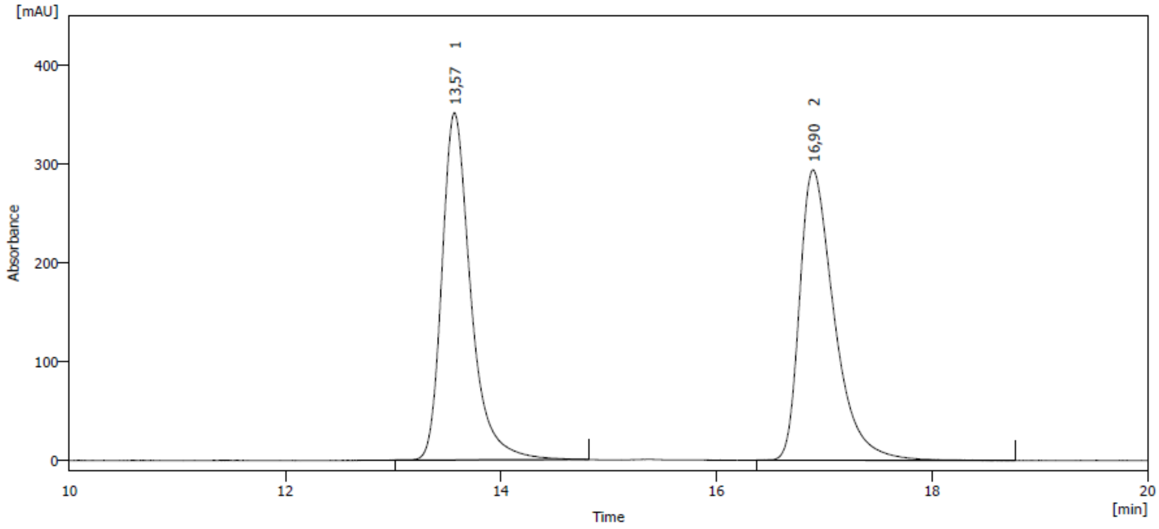

Result Table (Uncal - Data|67-KE-RAC-008_17_01_2025 16_24_26_443 - DAD 6.1L: Channel 1)

	Reten. Time [min]	Area [mAU.s]	Height [mAU]	Area [%]	Height [%]	W05 [min]	PDA Peak Purity
1	13,568	6529,986	352,049	49,8	54,5	0,27	896
2	16,895	6584,404	294,493	50,2	45,5	0,33	687
	Total	13114,390	646,542	100,0	100,0		

3eA

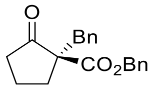

CHIRALPAK® *IA*, *n*-hexane/*i*PrOH = 97/3, 0.5 mL/min, *λ* = 215 nm

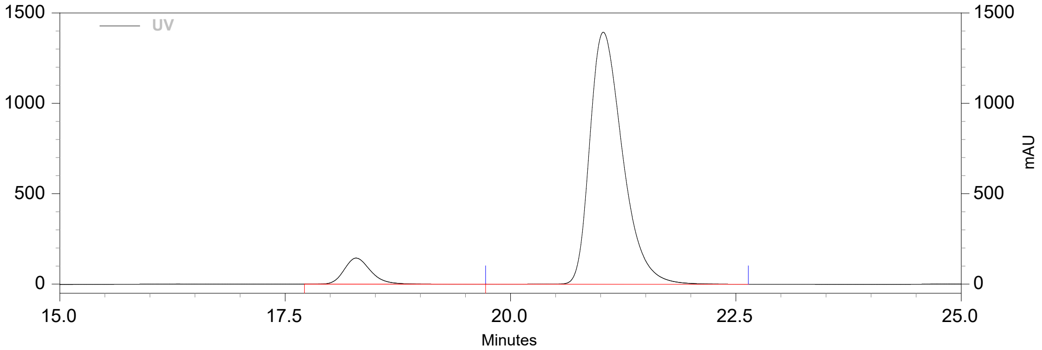

UV Results

Retention Time	Area	Area %	Height	Height %
18.287	11701905	7.66	577074	9.38
21.030	141071886	92.34	5575618	90.62
Totals	152773791	100.00	6152692	100.00

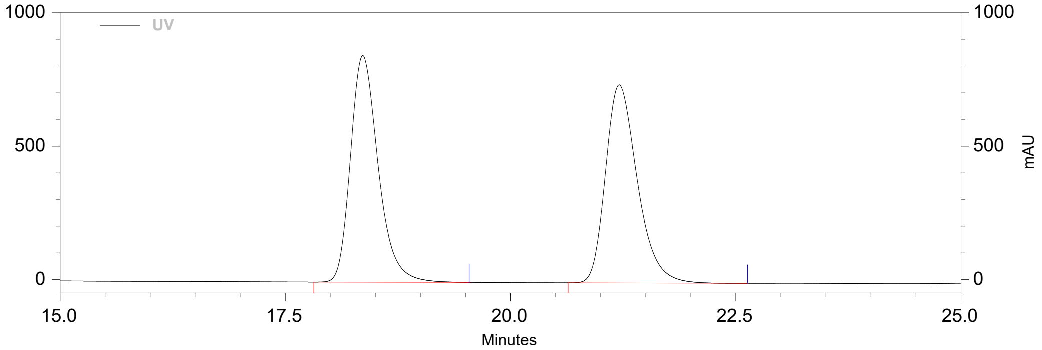

UV Results

Retention Time	Area	Area %	Height	Height %
18.360	72783216	49.94	3395931	53.35
21.207	72950638	50.06	2969385	46.65
Totals	145733854	100.00	6365316	100.00

3fA

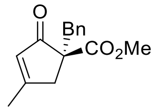

CHIRALPAK® *AD-H*, *n*-hexane/*i*PrOH = 97/3, 0.8 mL/min, *λ* = 215 nm

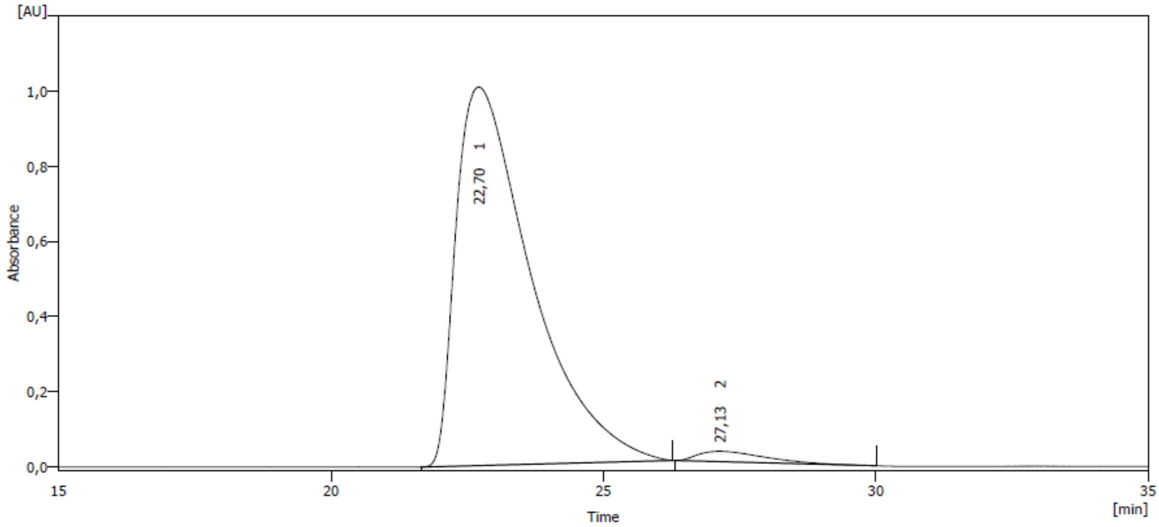

Result Table (Uncal - Data|67-KE-KAT-091 B_25_02_2025 12_30_09_534 - DAD 6.1L: Channel 1)

	Reten. Time [min]	Area [mAU.s]	Height [mAU]	Area [%]	Height [%]	W05 [min]	PDA Peak Purity
1	22,705	96829,230	1009,477	97,6	97,3	1,44	963
2	27,132	2430,800	27,932	2,4	2,7	1,25	956
	Total	99260,030	1037,409	100,0	100,0		

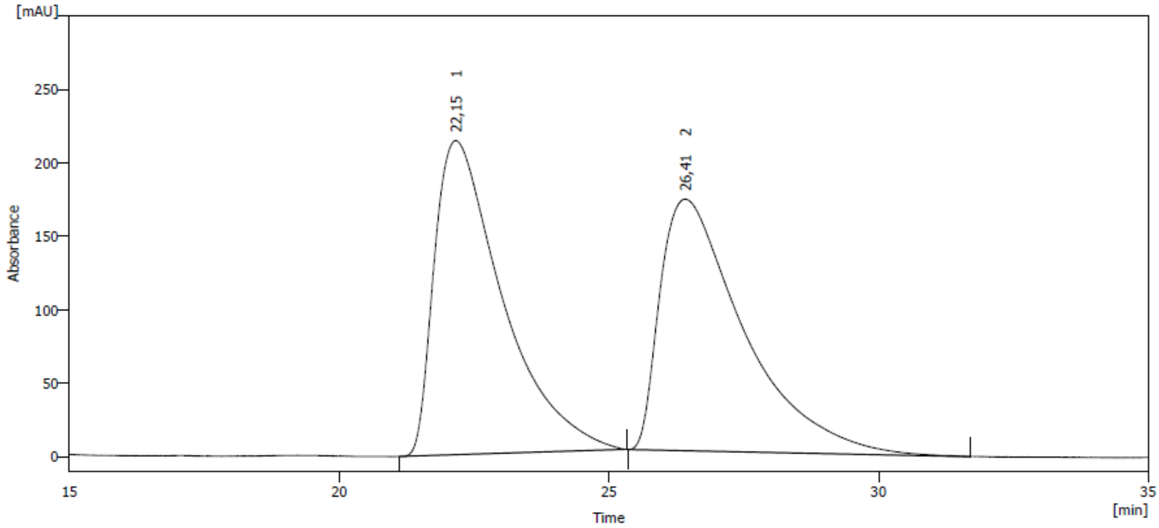

Result Table (Uncal - Data|67-KE-RAC-023 AD-H_25_02_2025 13_21_49_535 - DAD 6.1L: Channel 1)

	Reten. Time [min]	Area [mAU.s]	Height [mAU]	Area [%]	Height [%]	W05 [min]	PDA Peak Purity
1	22,153	18826,671	214,268	50,4	55,5	1,32	966
2	26,407	18531,474	171,591	49,6	44,5	1,59	897
	Total	37358,145	385,859	100,0	100,0		

3gA

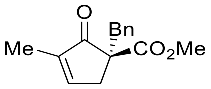

CHIRALPAK® *AD-H*, *n*-hexane/*i*PrOH = 97/3, 0.5 mL/min

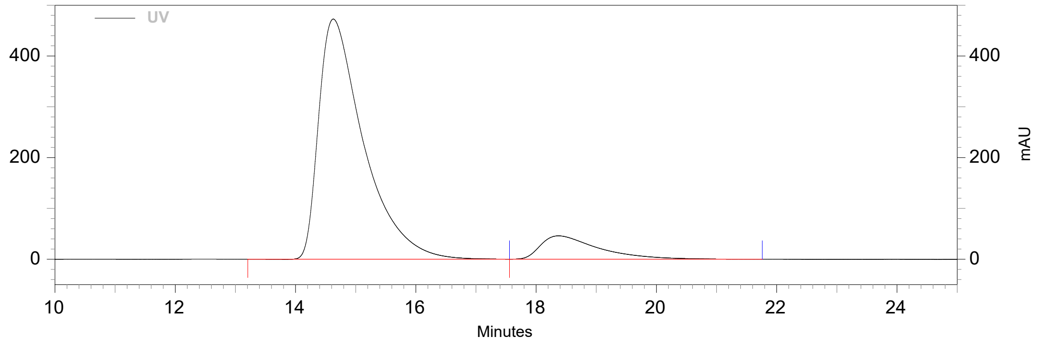

UV Results

Retention Time	Area	Area %	Height	Height %
14.627	97900774	88.99	1890091	91.15
18.373	12111779	11.01	183435	8.85
Totals	110012553	100.00	2073526	100.00

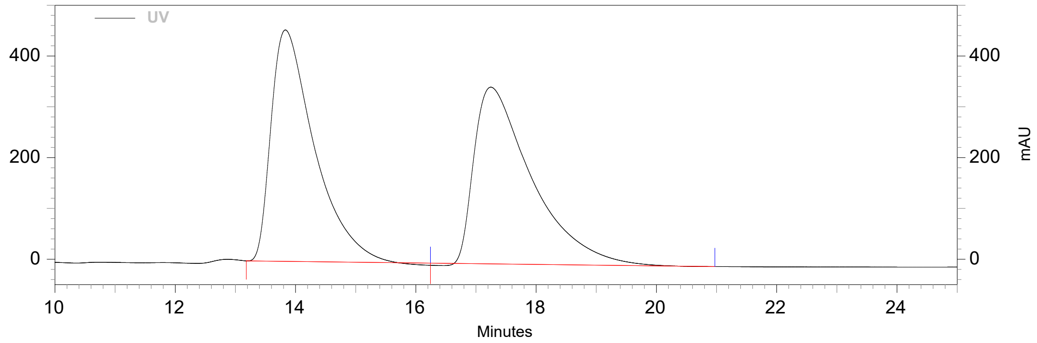

UV Results

Retention Time	Area	Area %	Height	Height %
13.830	91109273	49.64	1822030	56.70
17.247	92431394	50.36	1391180	43.30
Totals	183540667	100.00	3213210	100.00

3hA

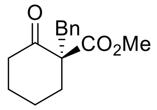

CHIRALPAK® *IC*, *n*-hexane/iPrOH = 97/3, 0.5 mL/min, λ = 215 nm

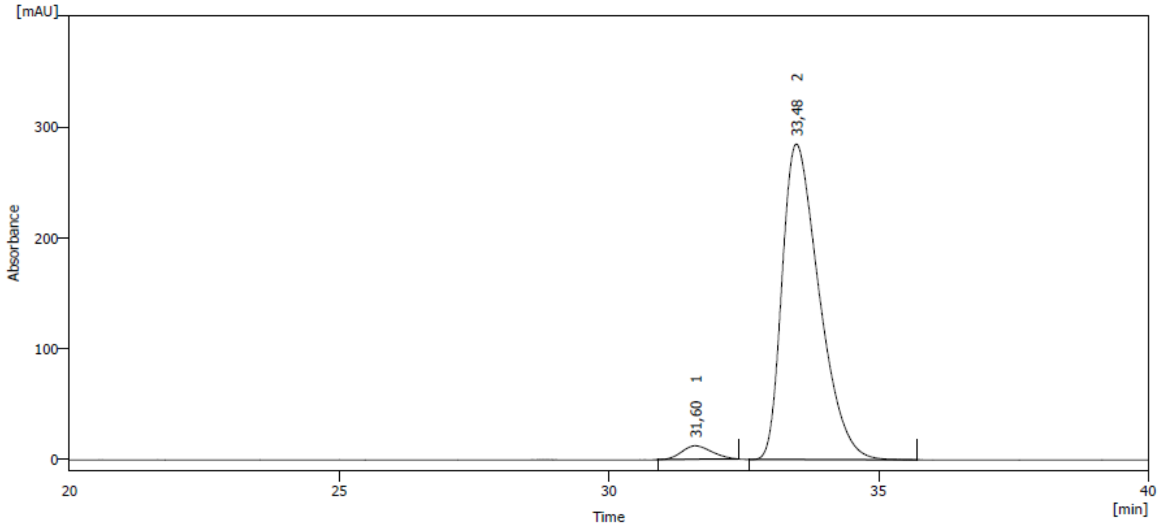

Result Table (Uncal - Data|67-KE-KAT-100 A_19_03_2025 09_48_47_572 - DAD 6.1L: Channel 1)

	Reten. Time [min]	Area [mAU.s]	Height [mAU]	Area [%]	Height [%]	W05 [min]	PDA Peak Purity
1	31,603	476,487	12,353	3,4	4,2	0,61	904
2	33,475	13618,635	284,960	96,6	95,8	0,74	670
	Total	14095,122	297,313	100,0	100,0		

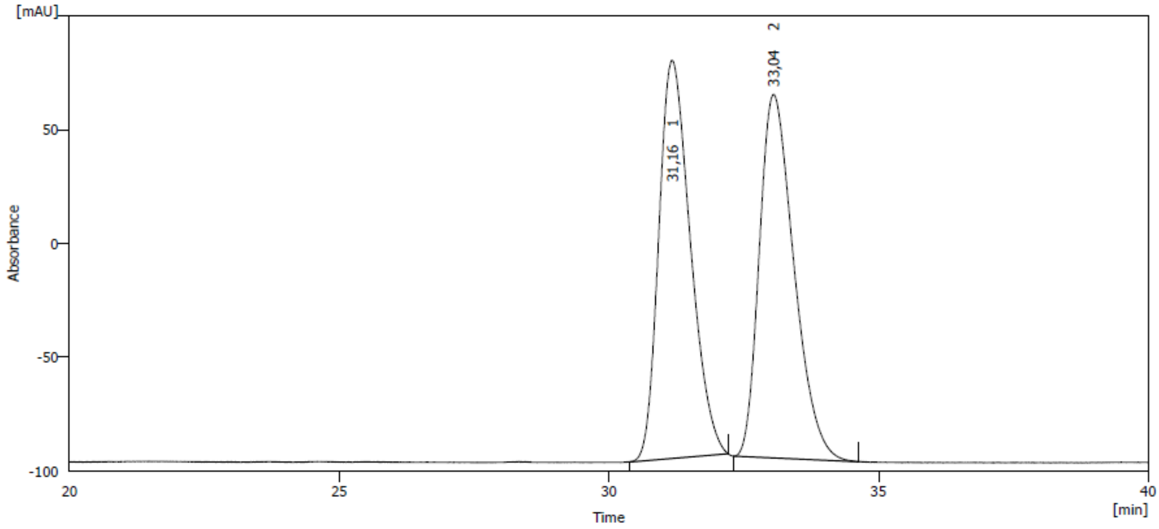

Result Table (Uncal - Data|67-KE-RAC-030 IC_18_03_2025 18_48_42_565 - DAD 6.1L: Channel 1)

	Reten. Time [min]	Area [mAU.s]	Height [mAU]	Area [%]	Height [%]	W05 [min]	PDA Peak Purity
1	31,163	7129,985	175,287	49,9	52,3	0,64	554
2	33,042	7147,512	159,971	50,1	47,7	0,69	523
	Total	14277,497	335,258	100,0	100,0		

3iA

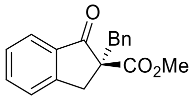

CHIRALPAK® *AD-H*, *n*-hexane/iPrOH = 98/2, 0.8 mL/min, *λ* = 215 nm

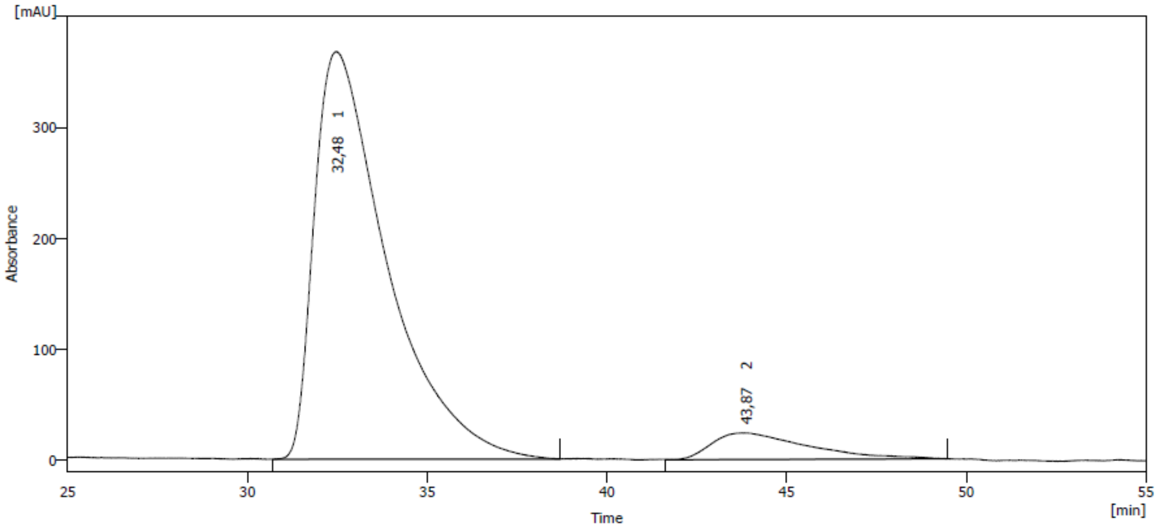

Result Table (Uncal - Data|67-KE-KAT-089I_29_05_2025 14_29_05_676 - DAD 6.1L: Channel 3)

	Reten. Time [min]	Area [mAU.s]	Height [mAU]	Area [%]	Height [%]	W05 [min]	PDA Peak Purity
1	32,483	51715,792	367,916	92,0	93,8	2,05	875
2	43,867	4503,221	24,266	8,0	6,2	2,82	812
	Total	56219,014	392,182	100,0	100,0		

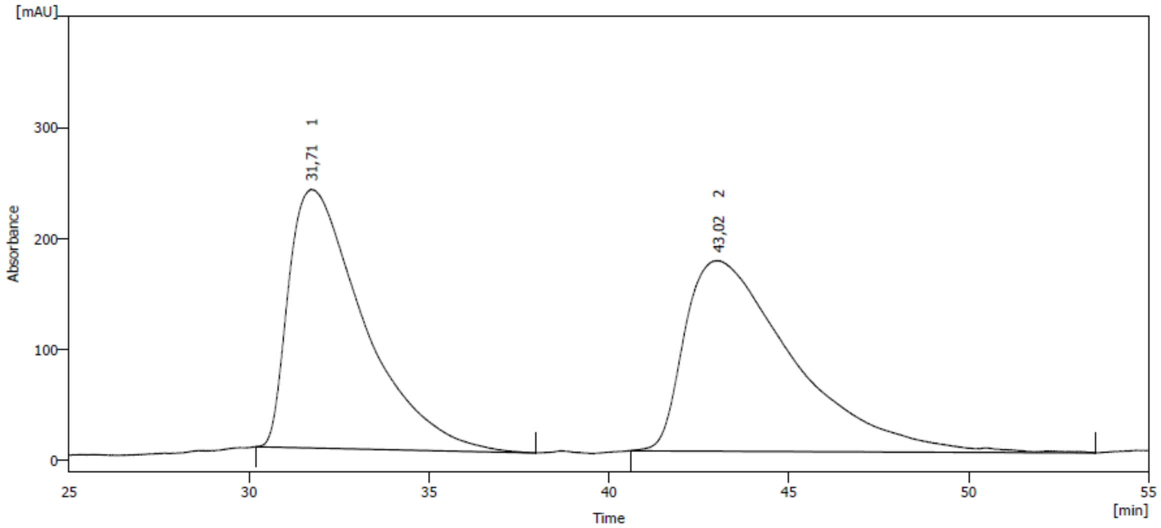

Result Table (Uncal - Data|67-KE-RAC-020 AD-H_14_02_2025 09_30_41_506 - DAD 6.1L: Channel 3)

	Reten. Time [min]	Area [mAU.s]	Height [mAU]	Area [%]	Height [%]	W05 [min]	PDA Peak Purity
1	31,712	34056,784	233,375	48,4	57,6	2,15	918
2	43,022	36304,630	172,084	51,6	42,4	3,09	858
	Total	70361,413	405,459	100,0	100,0		

3jA

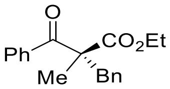

CHIRALPAK® *IJ*, *n*-hexane/iPrOH = 97/3, 0.5 mL/min, λ = 215 nm

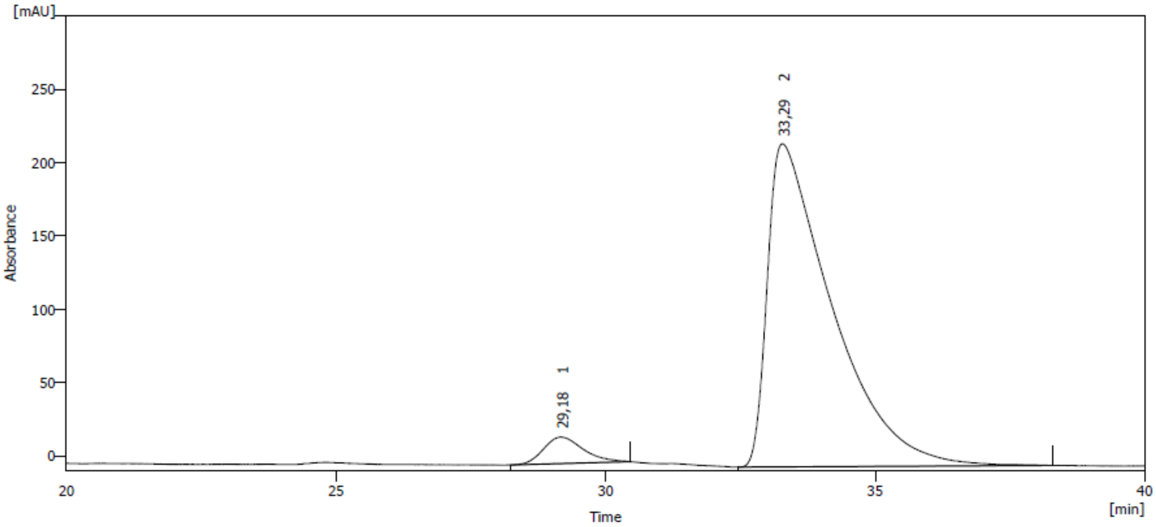

Result Table (Uncal - Data|67-KE-KAT-120-5A_26_08_2025 17_23_39_782 - DAD 6.1L: Channel 1)

	Reten. Time [min]	Area [mAU.s]	Height [mAU]	Area [%]	Height [%]	W05 [min]	PDA Peak Purity
1	29,185	936,429	18,210	4,8	7,6	0,80	803
2	33,290	18402,268	220,660	95,2	92,4	1,23	640
	Total	19338,697	238,870	100,0	100,0		

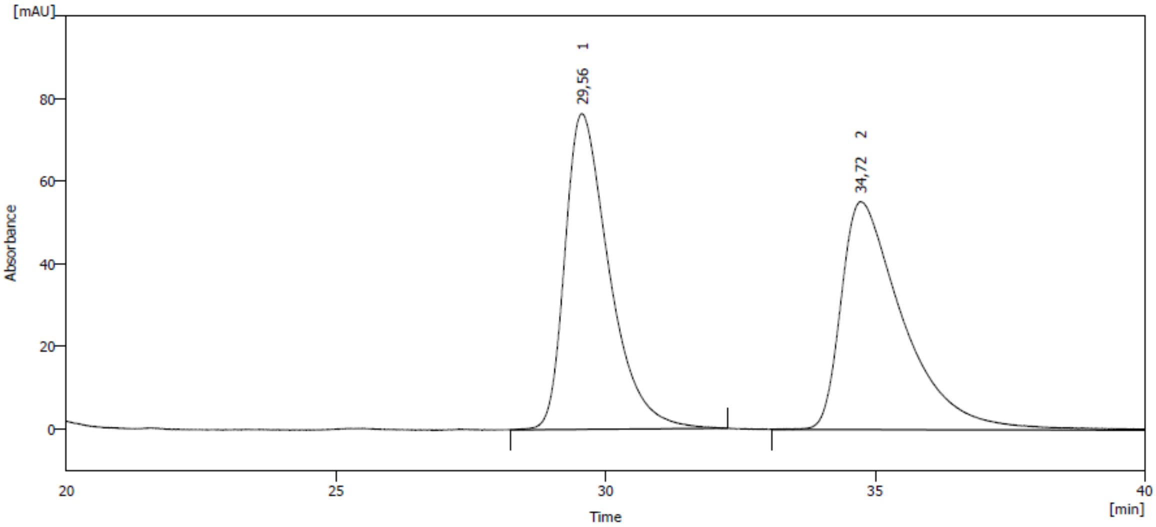

Result Table (Uncal - Data|67-KE-RAC-050-IJ_26_08_2025 19_55_31_785 - DAD 6.1L: Channel 1)

	Reten. Time [min]	Area [mAU.s]	Height [mAU]	Area [%]	Height [%]	W05 [min]	PDA Peak Purity
1	29,557	4375,040	76,550	49,6	58,1	0,85	632
2	34,715	4449,998	55,275	50,4	41,9	1,16	483
	Total	8825,038	131,826	100,0	100,0		

3kA

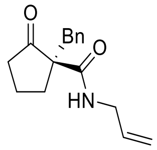

CHIRALPAK® *OD-H*, *n*-hexane/*i*PrOH = 90/10, 1.0 mL/min, λ = 215 nm

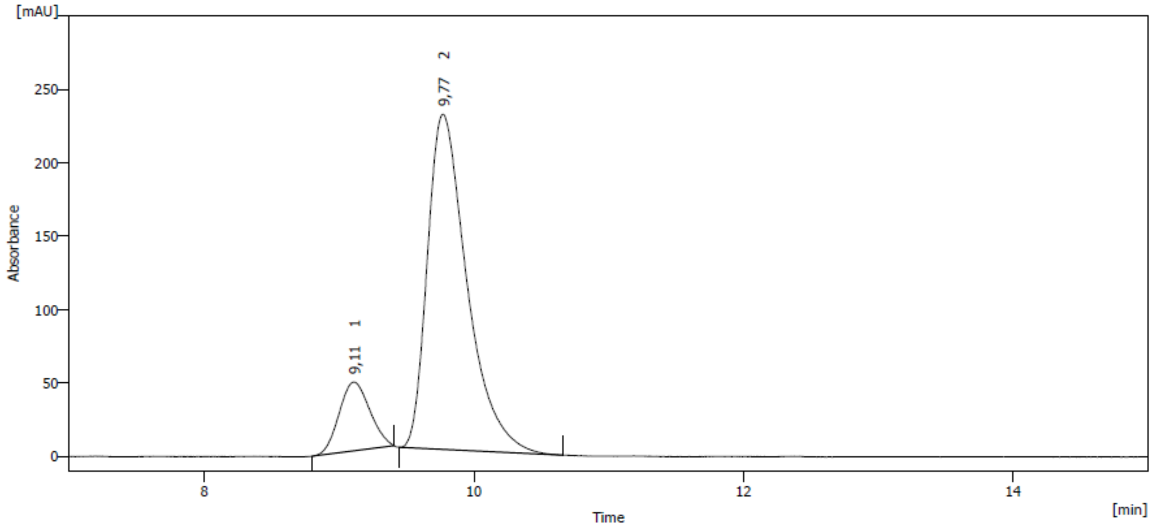

Result Table (Uncal - Data|67-KE-KAT-141 B_25_08_2025 13_49_06_774 - DAD 6.1L: Channel 1)

	Reten. Time [min]	Area [mAU.s]	Height [mAU]	Area [%]	Height [%]	W05 [min]	PDA Peak Purity
1	9,110	742,520	46,871	13,7	17,0	0,26	931
2	9,772	4678,676	228,751	86,3	83,0	0,31	913
	Total	5421,196	275,622	100,0	100,0		

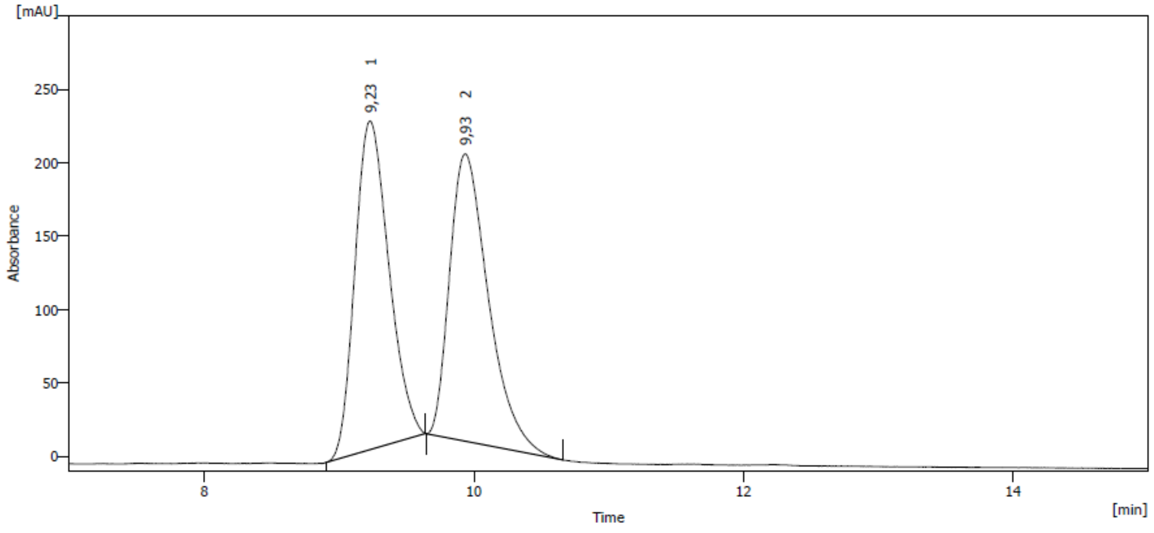

Result Table (Uncal - Data|67-KE-RAC-070 OD-H_25_08_2025 10_15_05_768 - DAD 6.1L: Channel 1)

	Reten. Time [min]	Area [mAU.s]	Height [mAU]	Area [%]	Height [%]	W05 [min]	PDA Peak Purity
1	9,227	3920,306	224,329	50,0	53,4	0,28	913
2	9,933	3920,789	196,016	50,0	46,6	0,30	847
	Total	7841,096	420,345	100,0	100,0		

3lA

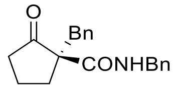

CHIRALPAK® *OD-H*, *n*-hexane/*i*PrOH = 90/10, 1.0 mL/min, *λ* = 215 nm

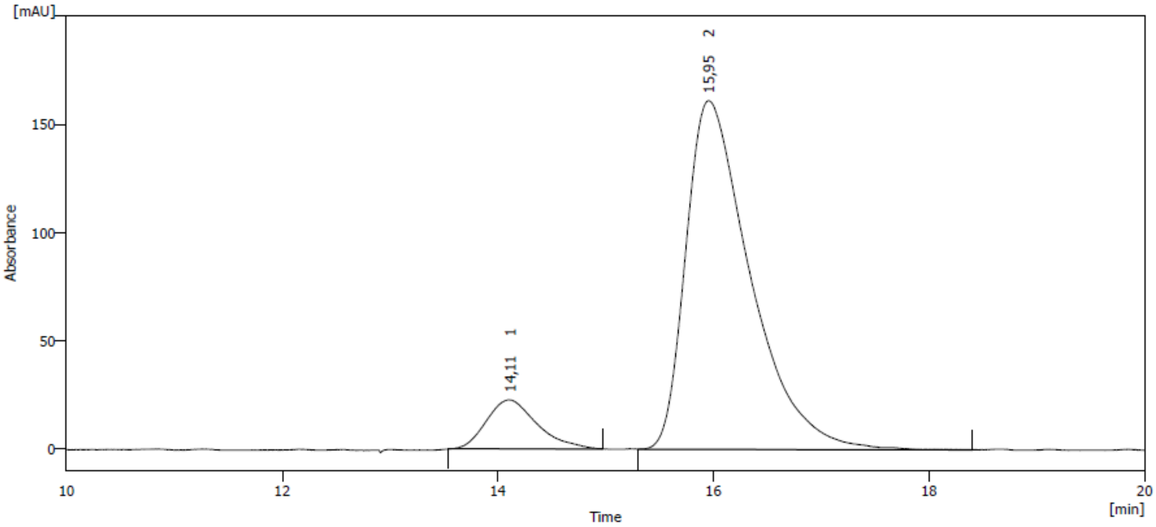

Result Table (Uncal - Data|67-KE-KAT-126 F_25_08_2025 14_30_16_776 - DAD 6.1L: Channel 1)

	Reten. Time [min]	Area [mAU.s]	Height [mAU]	Area [%]	Height [%]	W05 [min]	PDA Peak Purity
1	14,108	742,309	22,723	10,0	12,4	0,50	765
2	15,948	6712,333	161,251	90,0	87,6	0,62	619
	Total	7454,642	183,975	100,0	100,0		

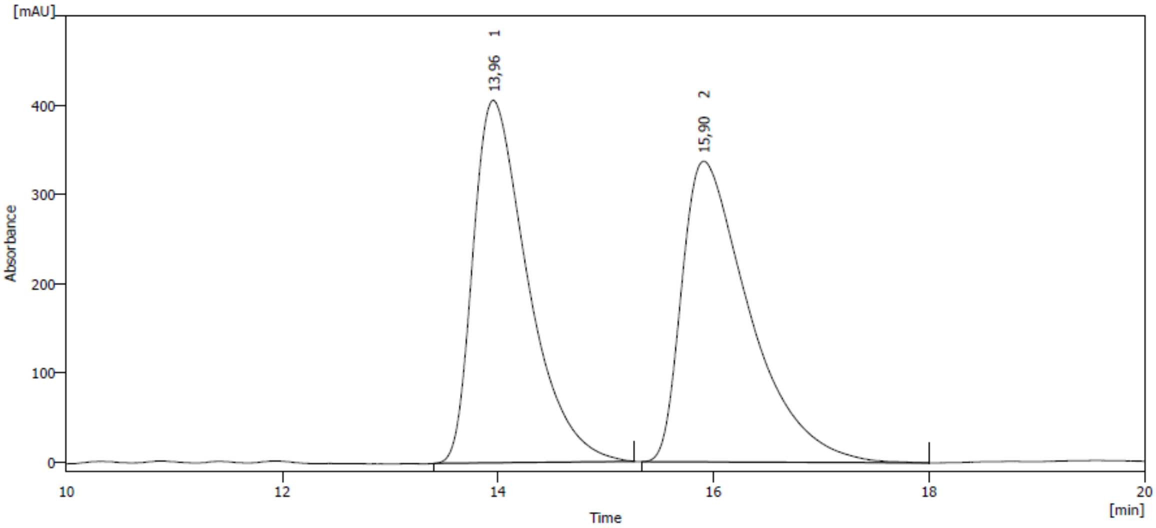

Result Table (Uncal - Data|67-KE-RAC-058 OD-H_25_08_2025 15_11_25_778 - DAD 6.1L: Channel 1)

	Reten. Time [min]	Area [mAU.s]	Height [mAU]	Area [%]	Height [%]	W05 [min]	PDA Peak Purity
1	13,958	14327,810	406,617	49,3	54,7	0,53	892
2	15,905	14751,328	337,181	50,7	45,3	0,65	772
	Total	29079,138	743,798	100,0	100,0		

15 GC Data

3aQ

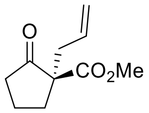

Bondex un ß column with H_2 carrier gas (40 °C for 1 min then with 0.5 °C/min to 80 °C)

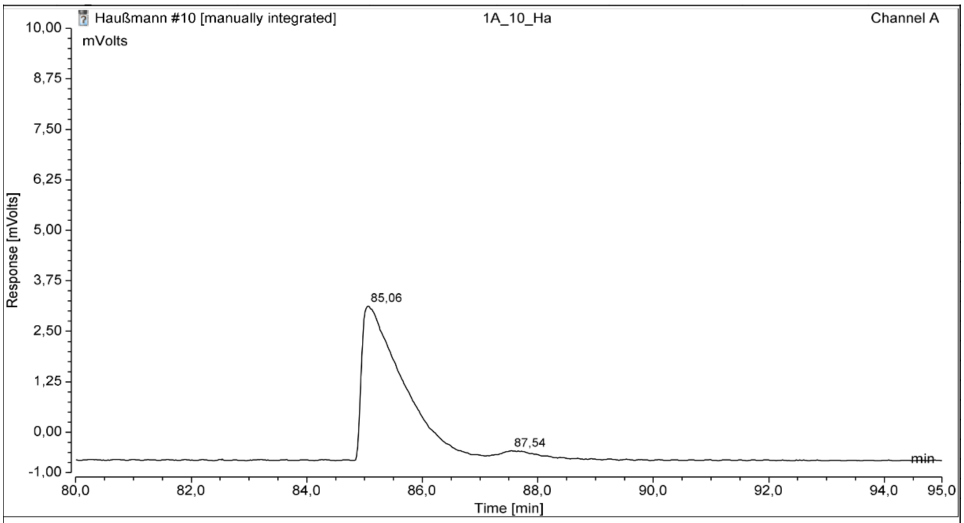

Integration Results

No.	Retention Time min	Area mVolts*min	Rel.Area %	Peak Type	Peak Width min	
1	85,061	3,061	97,90	BMB*	1,21	
2	87,545	0,066	2,10	BMB*	0,91	

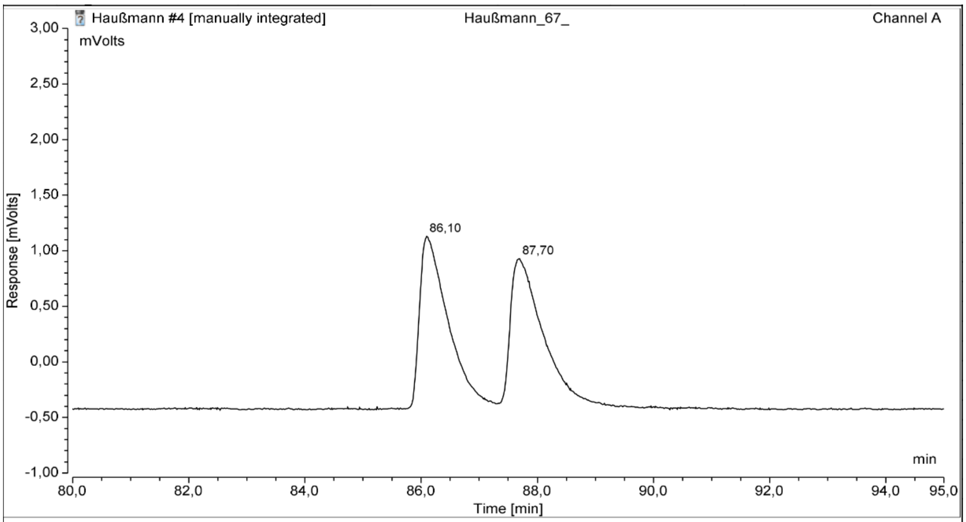

Integration Results

No.	Retention Time min	Area mVolts*min	Rel.Area %	Peak Type	Peak Width min	
1	86,098	0,854	51,04	BMb*	1,31	
2	87,698	0,819	48,96	bMB*	1,12	

3aU

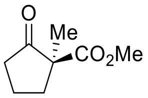

ChiralDex-B-DM column with H_2 carrier gas (40 °C for 1 min then with 2.5 °C/min to 200 °C)

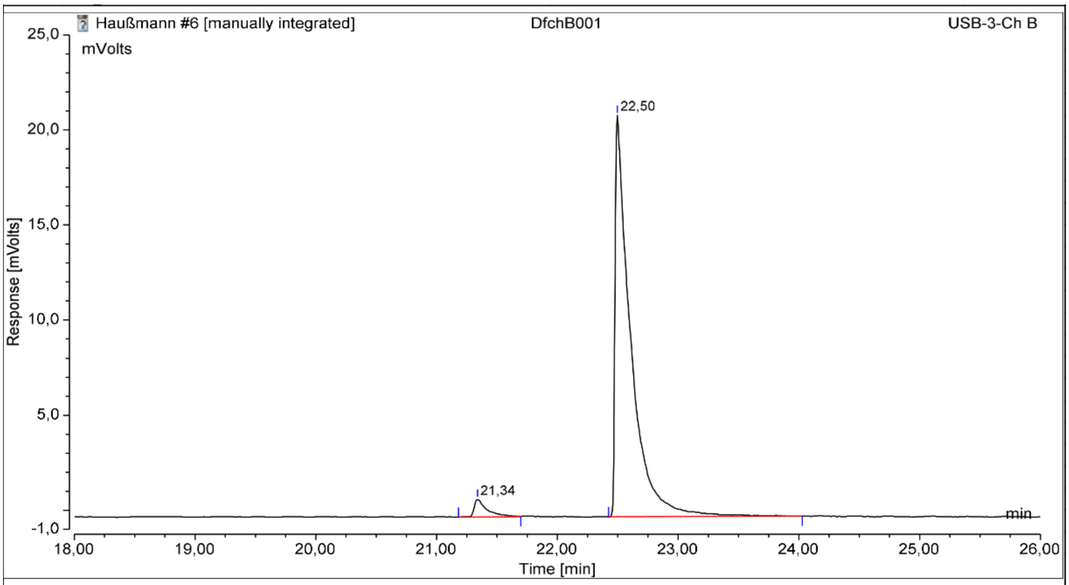

Integration Results

No.	Retention Time min	Area mVolts*min	Rel.Area %	Peak Type	Peak Width min	
1	21,338	0,114	3,50	BMB*	0,14	
2	22,498	3,150	96,50	BMB*	0,21	

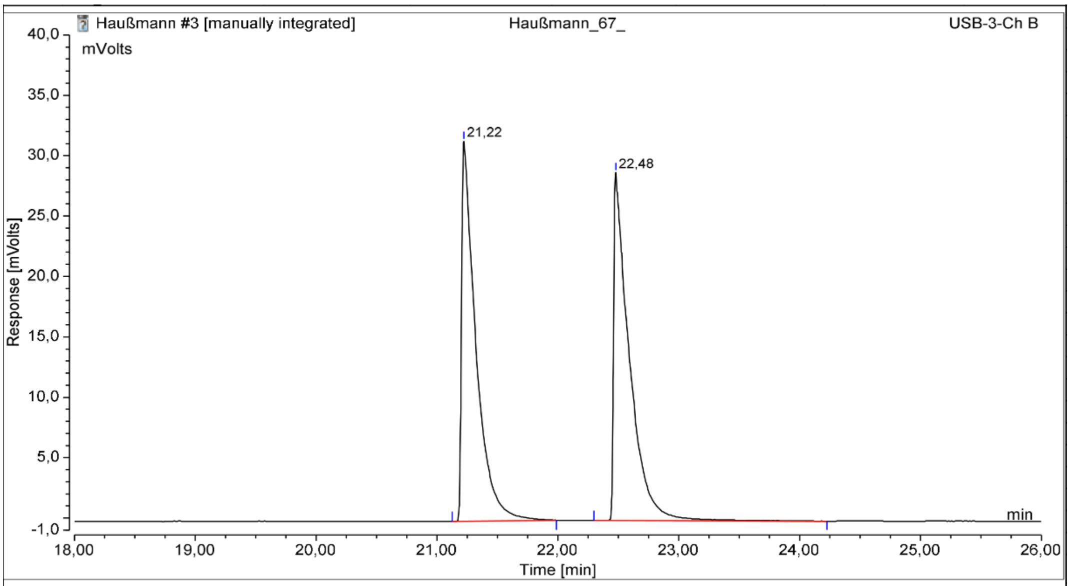

Integration Results

No.	Retention Time min	Area mVolts*min	Rel.Area %	Peak Type	Peak Width min	
1	21,223	4,281	49,58	BMB*	0,18	
2	22,480	4,354	50,42	BMB*	0,23	

3aV

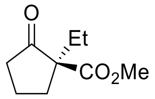

ChiralDex-B-DM column with H_2 carrier gas 40 °C for 1 min then with 0.5 °C/min to 200 °C

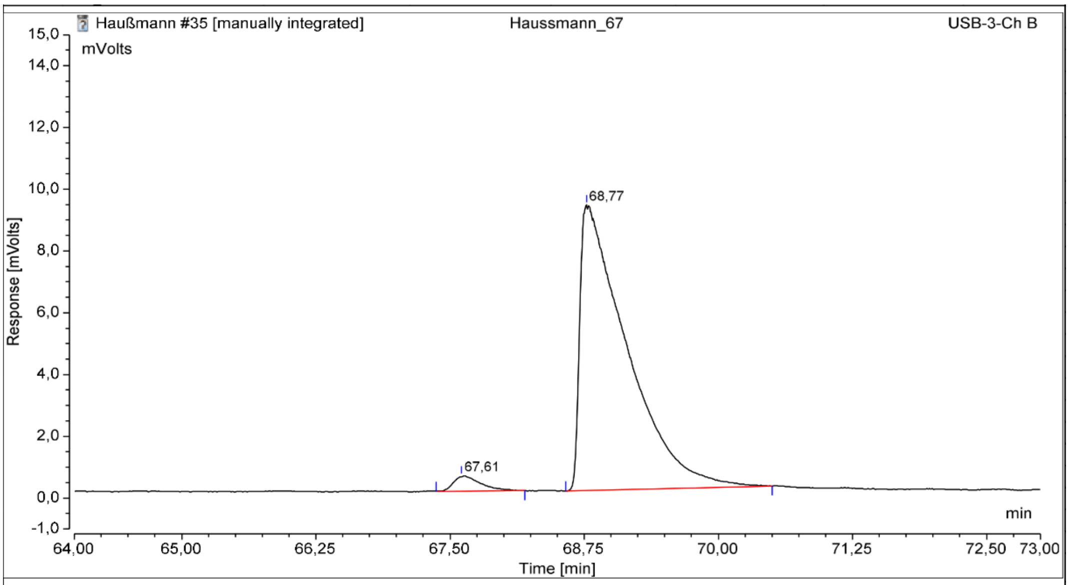

Integration Results

No.	Retention Time min	Area mVolts*min	Rel.Area %	Peak Type	Peak Width min	
1	67,608	0,142	2,96	BMB*	0,50	
2	68,770	4,668	97,04	BMB*	0,87	

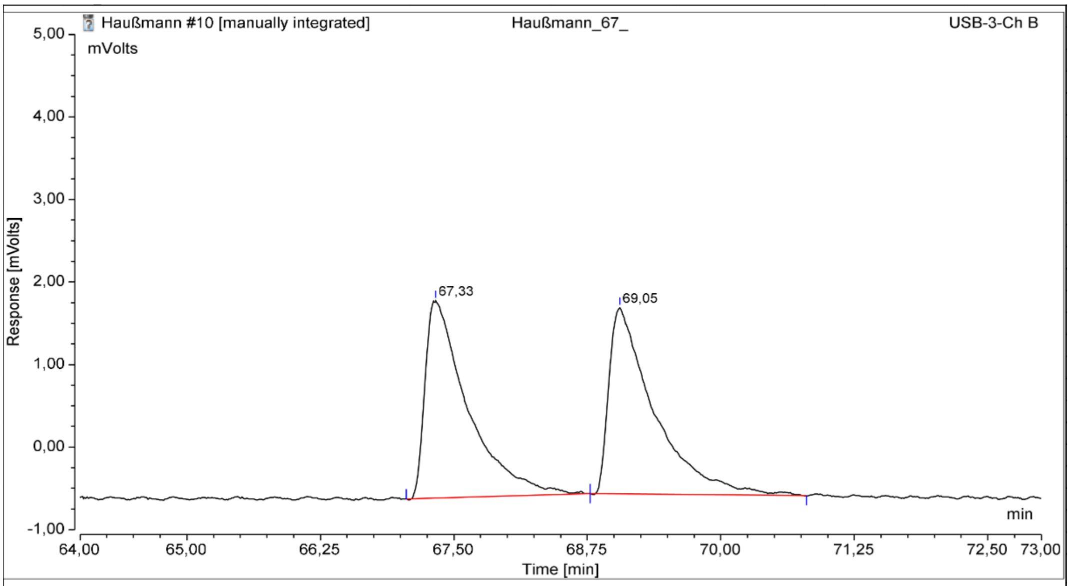

Integration Results

No.	Retention Time min	Area mVolts*min	Rel.Area %	Peak Type	Peak Width min	
1	67,330	1,105	50,07	BMB*	0,76	
2	69,053	1,102	49,93	BMB*	0,82	

3aW

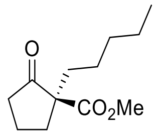

ChiralDex-B-DM column with H_2 carrier gas 40 °C for 1 min then with 1°C/min to 200 °C

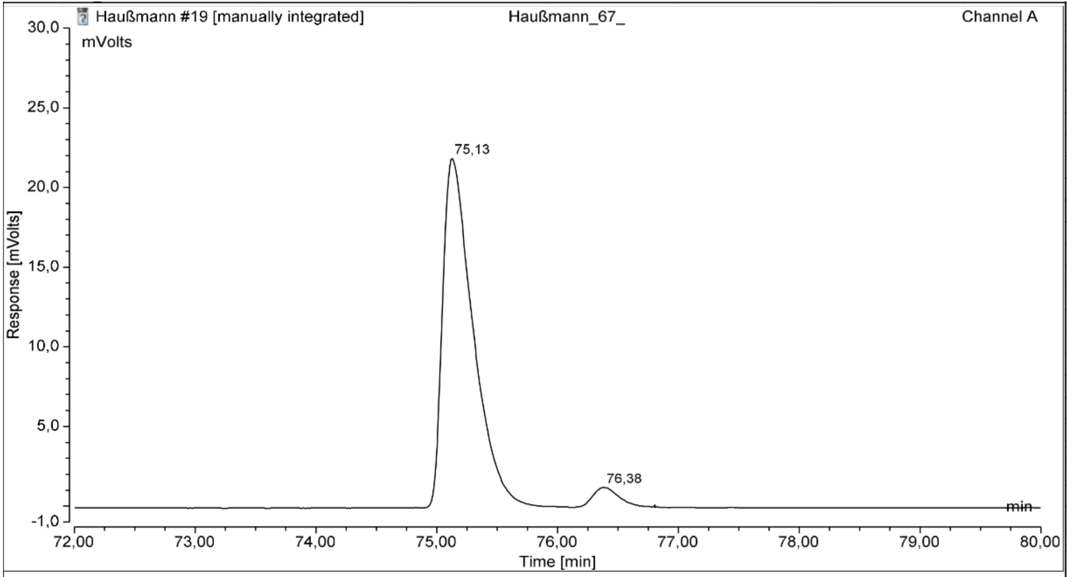

Integration Results

No.	Retention Time min	Area mVolts*min	Rel.Area %	Peak Type	Peak Width min	
1	75,125	6,402	95,21	BMB*	0,44	
2	76,383	0,322	4,79	BMB*	0,39	

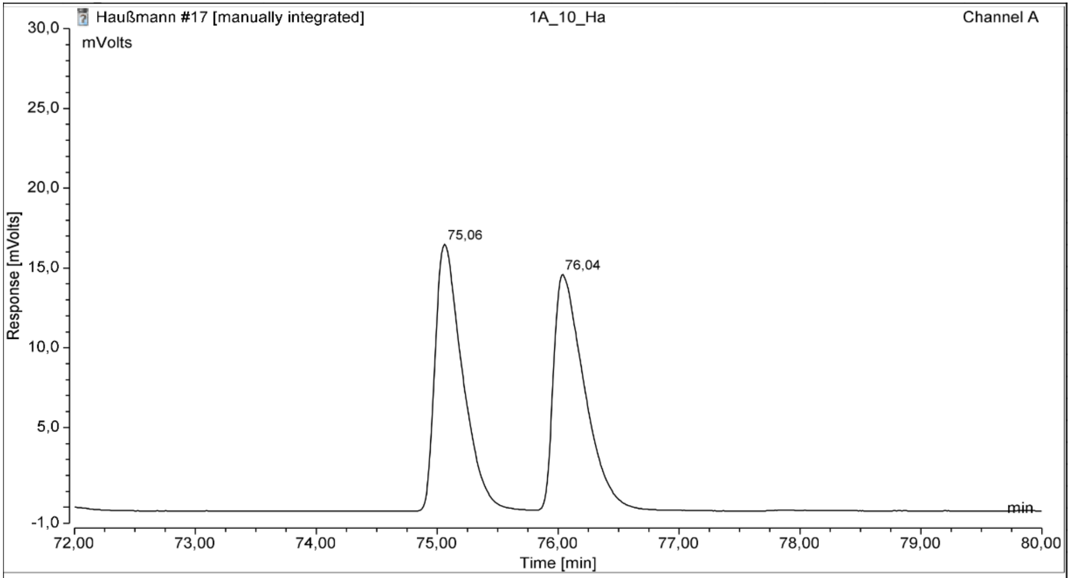

Integration Results

No.	Retention Time min	Area mVolts*min	Rel.Area %	Peak Type	Peak Width min	
1	75,062	4,371	50,07	BMB*	0,40	
2	76,035	4,359	49,93	BMB*	0,46	

3aX

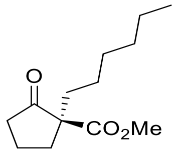

Bondex un Beta column with H_2 carrier gas 40 °C for 1 min then with 1 °C/min to 200 °C

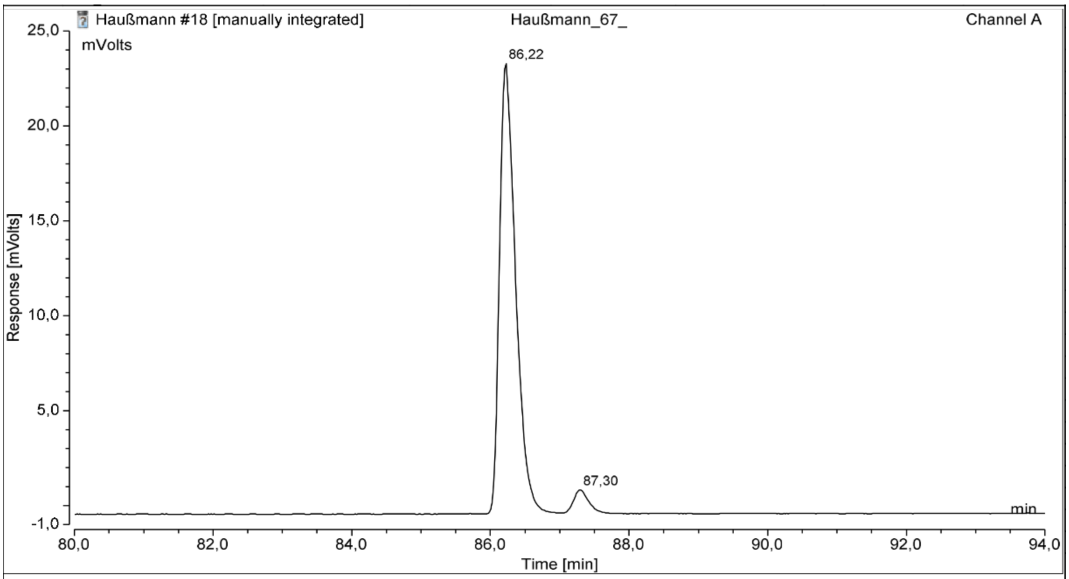

Integration Results

No.	Retention Time min	Area mVolts*min	Rel.Area %	Peak Type	Peak Width min	
1	86,222	6,332	95,51	BMB*	0,43	
2	87,297	0,298	4,49	BMB*	0,39	

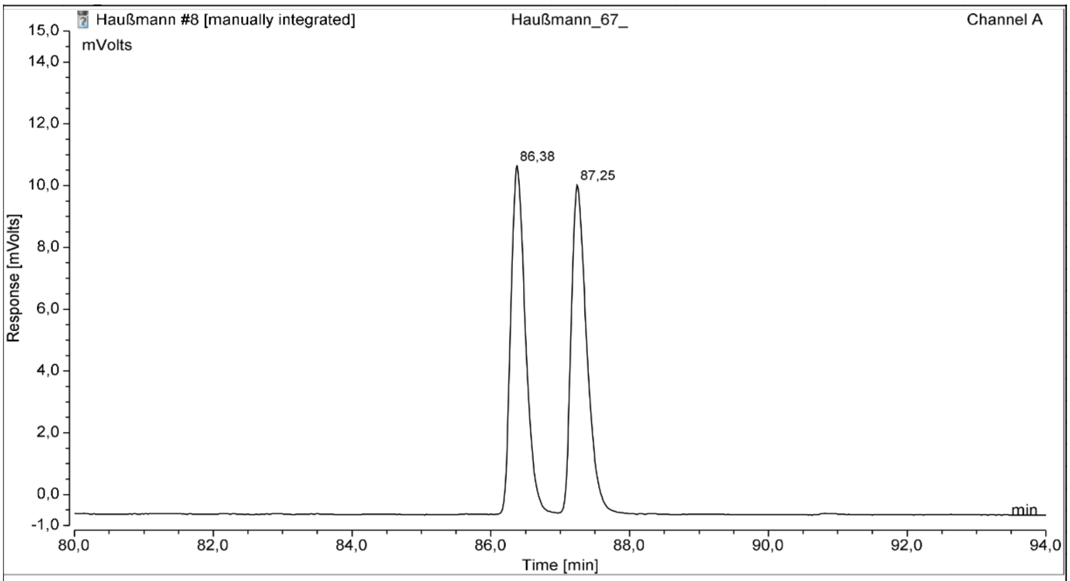

Integration Results

No.	Retention Time min	Area mVolts*min	Rel.Area %	Peak Type	Peak Width min	
1	86,375	2,748	49,99	BMB*	0,39	
2	87,245	2,749	50,01	BMB*	0,41	

3aY

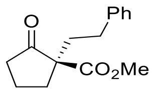

Bondex un beta column with H_2 carrier gas 40 °C for 1 min then with 1 °C/min to 100 °C for 3 min then with 1.0 °C min to 130 °C for 3 min then with 0.5 °C/min to 200 °C

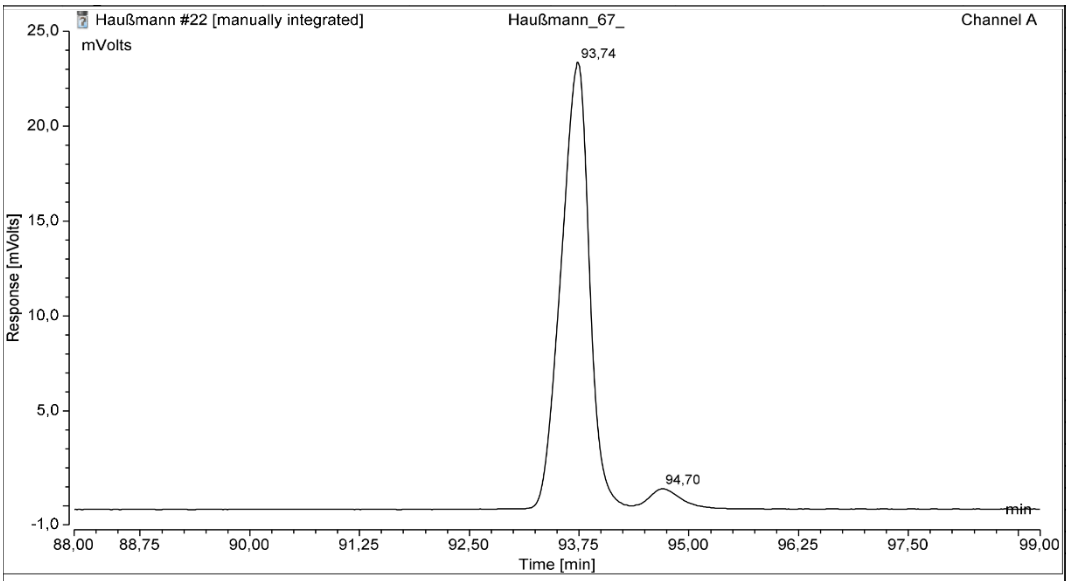

Integration Results

No.	Retention Time min	Area mVolts*min	Rel.Area %	Peak Type	Peak Width min	
1	93,738	8,659	96,03	BMB*	0,59	
2	94,701	0,358	3,97	BMB*	0,61	

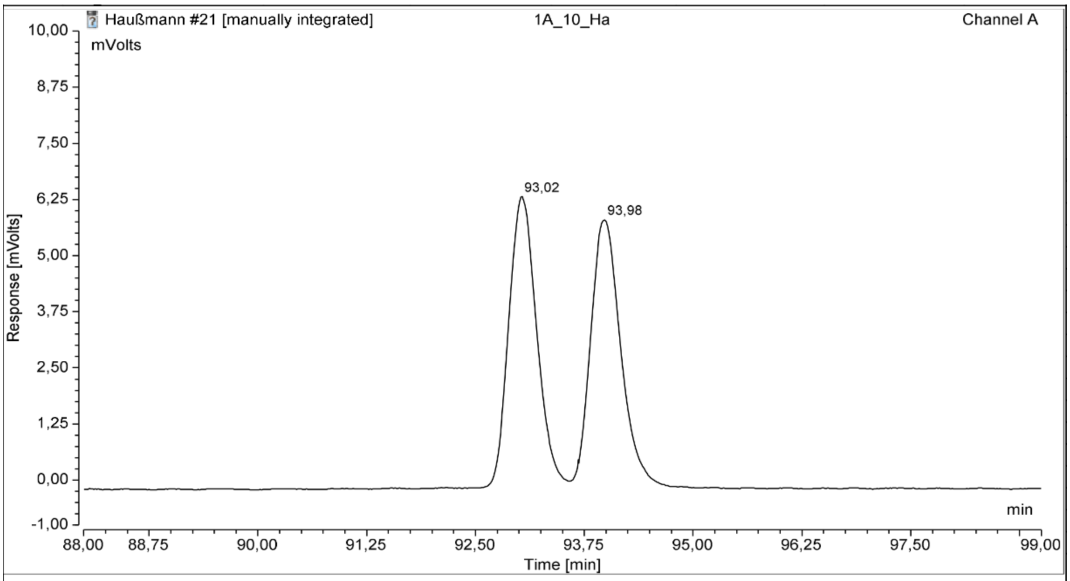

Integration Results

No.	Retention Time min	Area mVolts*min	Rel.Area %	Peak Type	Peak Width min	
1	93,025	2,319	50,36	BMB*	0,58	
2	93,979	2,286	49,64	BMB*	0,62	

3aZ

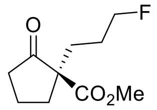

Beta-DexTM-225 column with H_2 carrier gas 40 °C for 1 min then with 2.5 °C/min to 200 °C

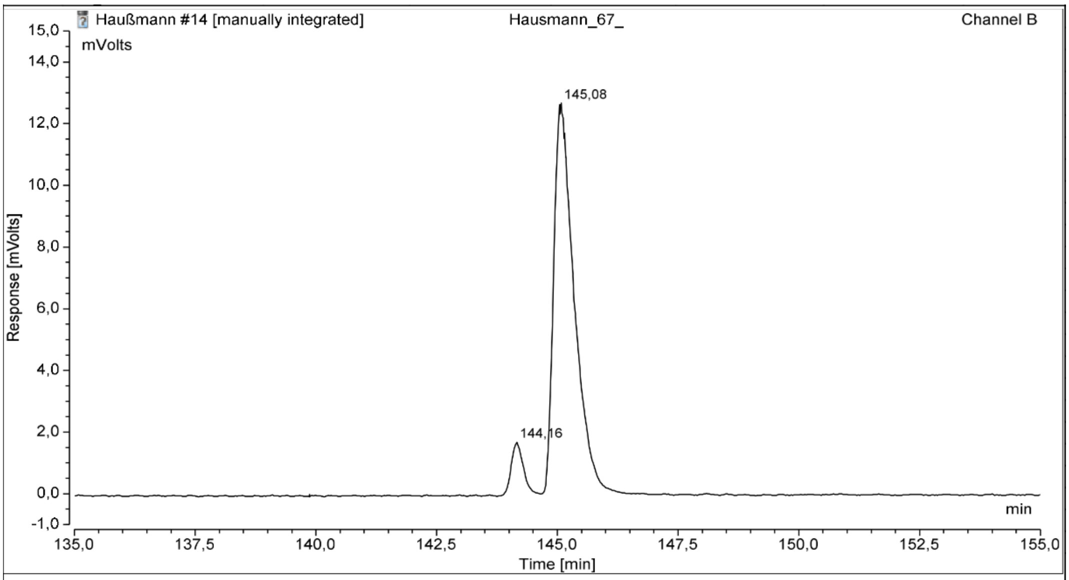

Integration Results

No.	Retention Time min	Area mVolts*min	Rel.Area %	Peak Type	Peak Width min
1	19,411	1,506	17,53	BMB*	1,48
2	124,897	0,414	4,82	BMB*	1,42

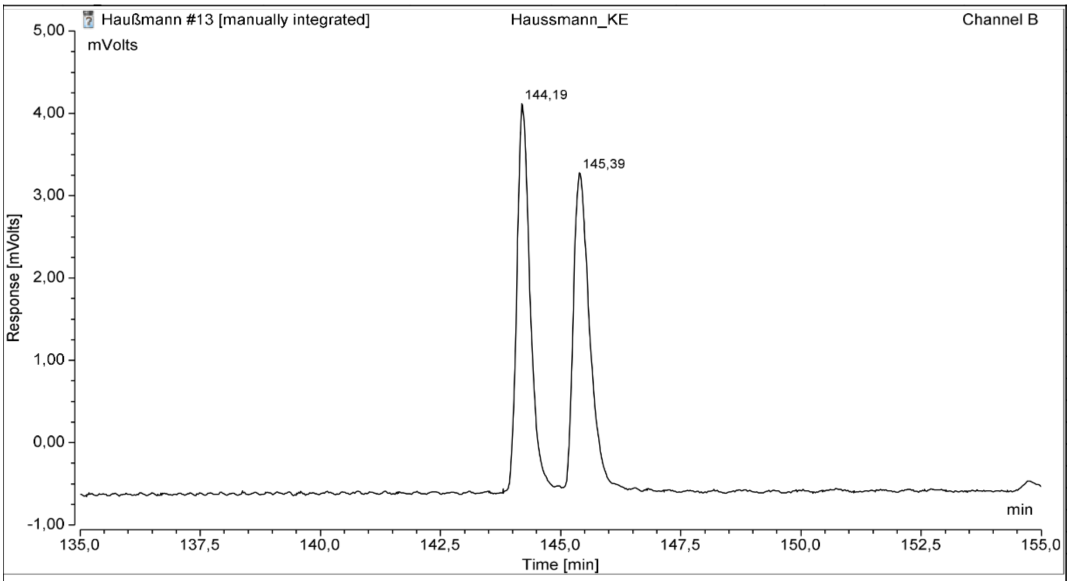

Integration Results

No.	Retention Time min	Area mVolts*min	Rel.Area %	Peak Type	Peak Width min
1	144,187	1,478	49,87	BMB*	0,54
2	145,391	1,485	50,13	BMB*	0,67

3mU

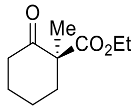

Bondex un alpha+beta column with H_2 carrier gas 40 °C for 1 min then with 0.5 °C/min to 200 °C

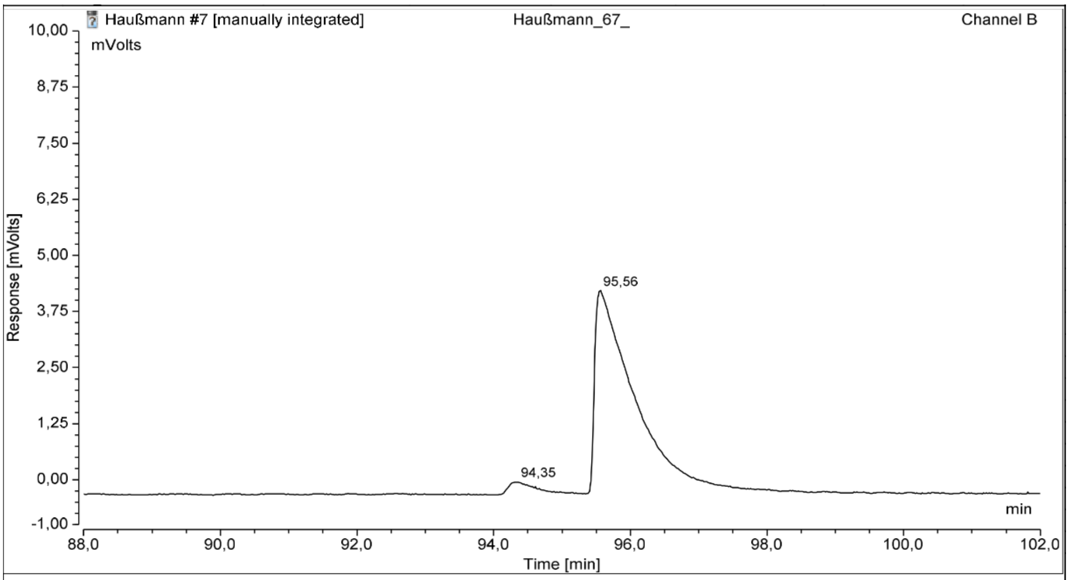

Integration Results

No.	Retention Time min	Area mVolts*min	Rel.Area %	Peak Type	Peak Width min	
1	94,354	0,095	3,01	BMB*	0,46	
2	95,558	3,077	96,99	BMB*	1,08	

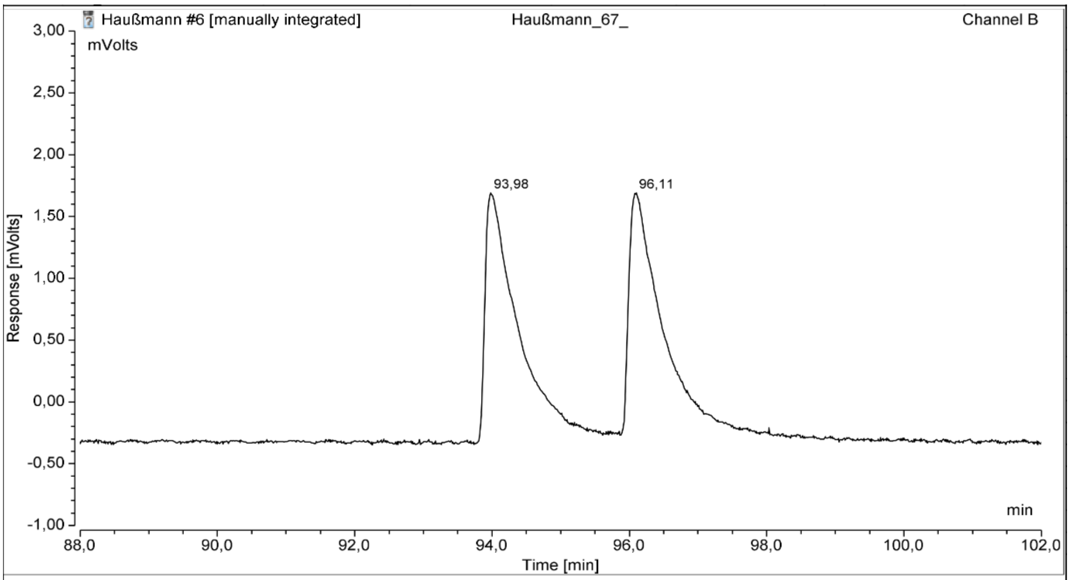

Integration Results

No.	Retention Time min	Area mVolts*min	Rel.Area %	Peak Type	Peak Width min	
1	93,983	1,098	50,50	BMB*	0,71	
2	96,105	1,077	49,50	BMB*	0,69	

3nU

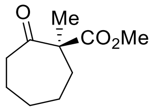

ChiralDex-B-DM column with H_2 carrier gas 40 °C for 1 min to 200 °C

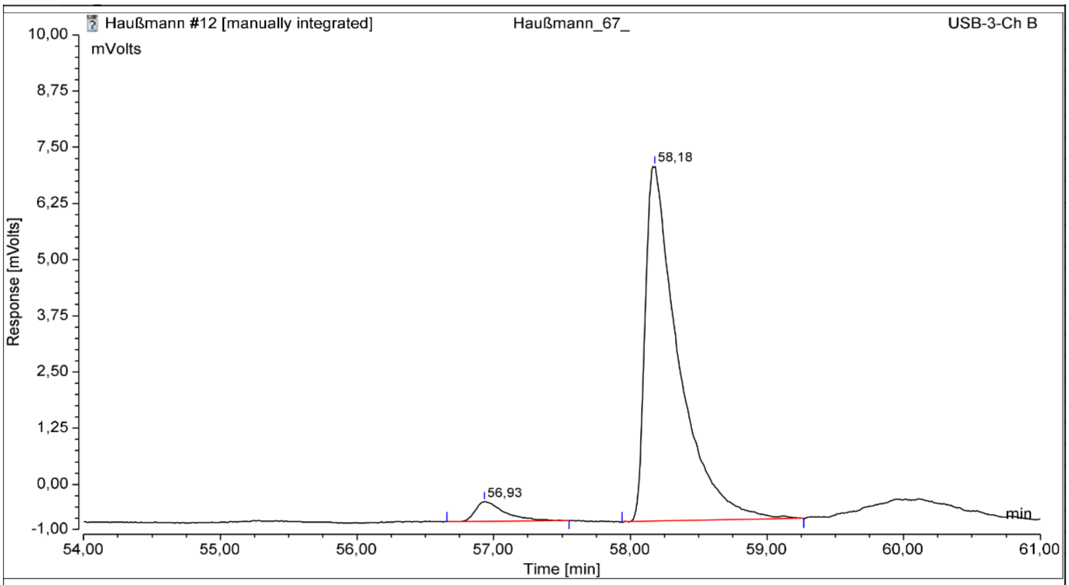

Integration Results

No.	Retention Time min	Area mVolts*min	Rel.Area %	Peak Type	Peak Width min	
1	56,932	0,105	4,51	BMB*	0,32	
2	58,179	2,224	95,49	BMB*	0,38	

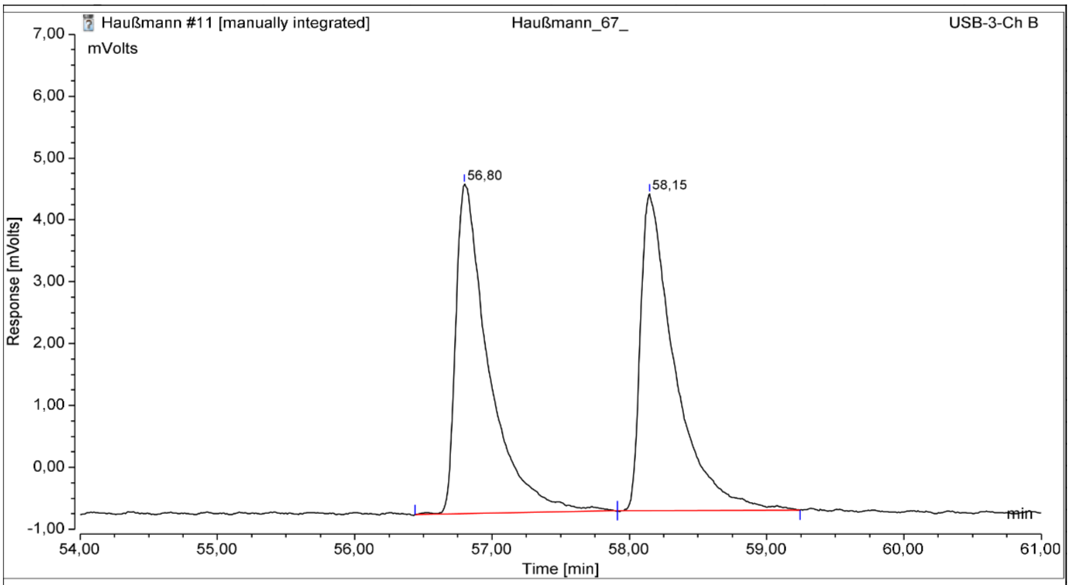

Integration Results

No.	Retention Time min	Area mVolts*min	Rel.Area %	Peak Type	Peak Width min	
1	56,799	1,482	50,12	BMB*	0,40	
2	58,146	1,475	49,88	BMB*	0,40	

3oU

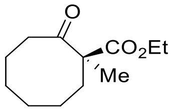

ChiralDex-B-DM column with H_2 carrier gas 40 °C for 1 min then with 1 °C/min to 80 °C for 3 min then with 0.5 °C/min 200 °C

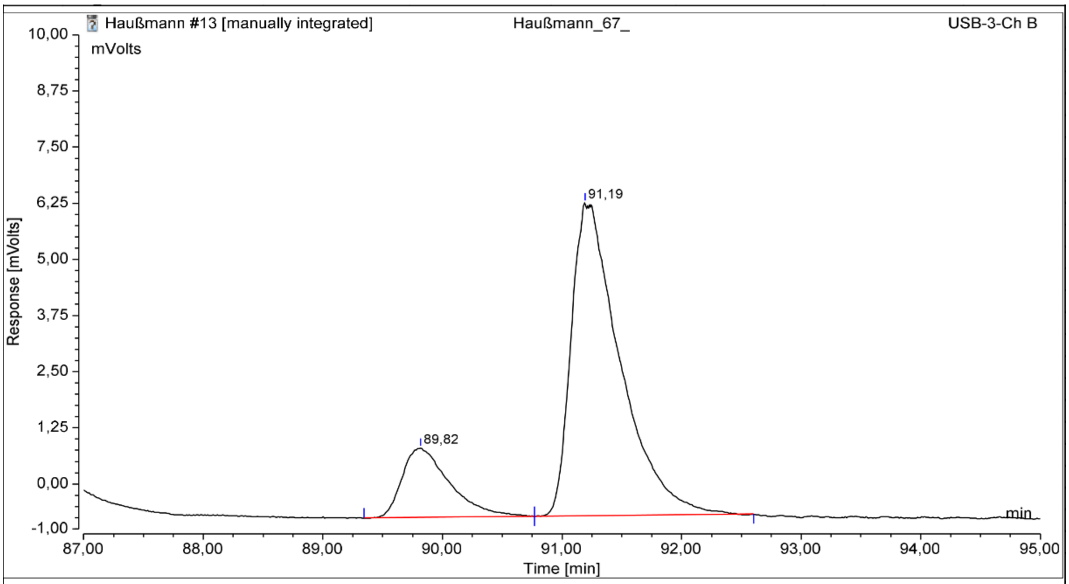

Integration Results

No.	Retention Time min	Area mVolts*min	Rel.Area %	Peak Type	Peak Width min	
1	89,818	0,705	17,61	BMb*	1,29	
2	91,194	3,297	82,39	bMB*	0,80	

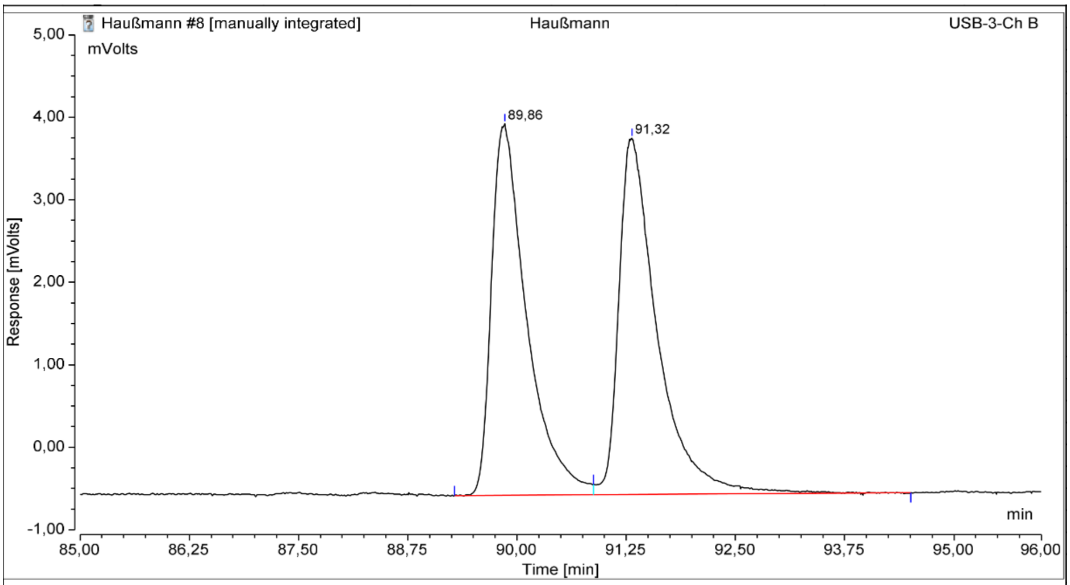

Integration Results

No.	Retention Time min	Area mVolts*min	Rel.Area %	Peak Type	Peak Width min	
1	89,861	2,097	48,75	BM *	0,70	
2	91,315	2,204	51,25	MB*	0,94	
